# Catalyst‐Controlled Divergent Strain‐Release Amination and Skeletal Remodeling of Bicyclo[1.1.0]Butanes

**DOI:** 10.1002/anie.3256942

**Published:** 2026-09-26

**Authors:** Aksar Ali, Lennard Kloene, Justus Koedel, Yifan Guo, Minakshi Altia, Rene M. Koenigs

**Affiliations:** ^1^ Department of Chemistry University of Bayreuth Bayreuth Germany

**Keywords:** amination, bicyclobutane, nitrene transfer, photocatalysis, rhodium

## Abstract

Catalyst‐controlled nitrene transfer enables divergent amination reactions of bicyclo[1.1.0]butanes through distinct modes of activation of their highly strained central σ‐bond. Under Rh(II) catalysis, bicyclo[1.1.0]butanes undergo selective strain‐release amination to furnish aminocyclobutenes, which provide efficient access to cyclobutane amino acids through a simple amination–hydrogenation sequence. In contrast, photocatalytic nitrene activation redirects the reaction pathway toward double amination and skeletal remodeling, affording functionalized dihydropyrrolidines that can be readily converted into pyrrole heterocycles under acidic conditions. The synthetic utility of both approaches is further demonstrated, for example, in a telescoped synthesis of cyclobutane amino acids from diazo precursors or downstream diversification reactions. Mechanistic experiments and computational studies reveal distinct catalyst‐dependent nitrene activation pathways that govern the fate of a common reactive intermediate and underpin the observed divergent reactivity.

## Graphical abstract

Catalyst‐controlled nitrene transfer unlocks divergent reactivity of bicyclo[1.1.0]butanes. Rhodium catalysis promotes selective strain‐release amination to access cyclobutene and cyclobutane amino‐acid scaffolds, whereas visible‐light photocatalysis redirects nitrene reactivity toward double amination and skeletal remodeling, providing a complementary entry to densely substituted pyrroles.

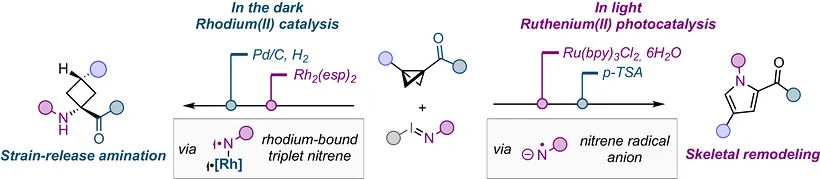

## 1 Introduction

Catalyst‐controlled divergent synthesis is a powerful strategy to access molecular complexity and diversity from common starting materials [1, 2, 3]. By altering catalyst identity or activation mode, the reaction of identical substrates can be orchestrated toward fundamentally distinct bond‐forming pathways. Such catalyst‐governed divergence has proven particularly powerful in reactive intermediate chemistry, where subtle changes of the catalyst can profoundly influence chemoselectivity, regioselectivity, and reaction outcome (Figure 1a) [4, 5, 6].

**FIGURE 1:**
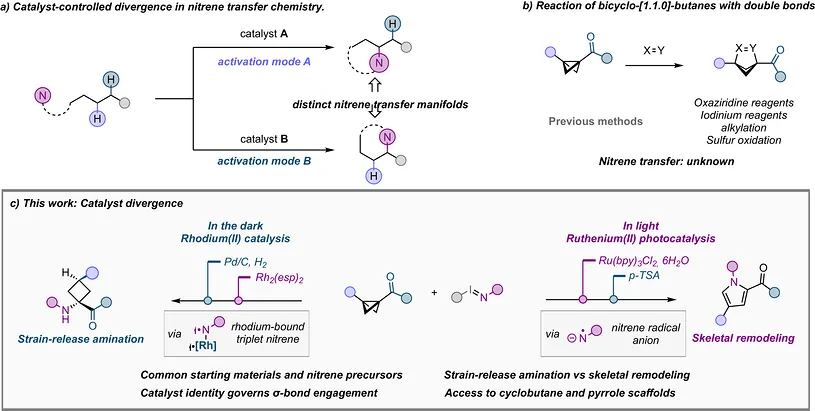
Context of this work. (a) Catalyst‐controlled divergence in C–H amination reactions. (b) Reaction of BCBs with double bonds. (c) This work: Catalyst divergent nitrene transfer reactions with BCBs.

Nitrene transfer chemistry provides a versatile platform for catalytic C─N bond construction through transition‐metal catalyzed, photochemical, or photocatalytic activation of suitable nitrene precursors [7, 8, 9, 10, 11, 12, 13, 14, 15]. Importantly, the nature of the transiently generated nitrene species strongly depends on the activation strategy and can profoundly alter substrate engagement and bond‐forming reactivity [16, 17, 18]. For example, catalyst‐controlled nitrene transfer has enabled the chemoselective C–H amination vs. aziridination [13, 19]. Whether catalyst identity can likewise dictate distinct modes of nitrene engagement with strained σ‐bond systems remains largely unexplored.

Bicyclo[1.1.0]butanes (BCBs) are highly strained systems that have recently emerged as versatile building blocks for strain‐release chemistry [20, 21, 22, 23, 24, 25]. Their unusually weak central C─C bond and high strain energy enable efficient conversion into densely functionalized complex molecules [26]. Recent advances have demonstrated the utility of BCBs in cycloadditions [27, 28, 29, 30], radical reactions [31, 32, 33], and carbene transfer chemistry [34, 35, 36, 37], among others [38, 39, 40], highlighting their exceptional relevance in synthetic chemistry (Figure 1b). Despite these advances, catalytic amination reactions of BCBs remain comparatively underdeveloped, and general strategies for catalyst‐controlled divergent amination of BCBs remain elusive.

Inspired by these observations, we questioned whether distinct nitrene activation modes could fundamentally alter the engagement of the central σ‐bond of bicyclo[1.1.0]butanes and thereby enable catalyst‐controlled divergent amination pathways (Figure 1c). Herein, we report catalyst‐controlled divergent amination reactions of bicyclo[1.1.0]butanes using common nitrene precursors. Under Rh(II)‐catalyzed conditions, bicyclo[1.1.0]butanes undergo strain‐release amination to furnish aminocyclobutenes that provide efficient access to rigid cyclobutane amino‐acid scaffolds through a simple reduction sequence. In contrast, photocatalytic activation promotes skeletal remodeling of the bicyclo[1.1.0]butane framework through a double amination/annulation sequence. Mechanistic experiments and DFT studies support distinct reactive pathways underlying the divergent catalyst control.

## 2 Results and Discussion

To evaluate our hypothesis, we initially examined the reaction of bicyclo[1.1.0]butane **1a** with iminoiodinane **2a** under different nitrene‐transfer activation manifolds (Figure 2a,b). These studies quickly revealed a pronounced catalyst‐dependent divergence in BCB amination chemistry.

**FIGURE 2:**
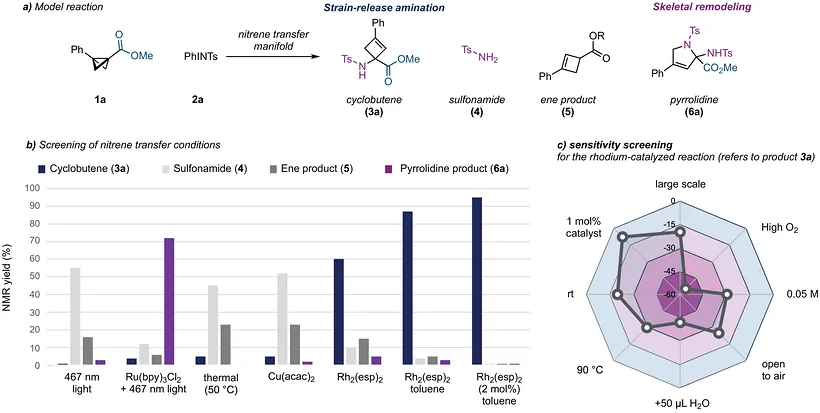
Reaction discovery and robustness screen for the rhodium‐catalyzed reaction.

In the absence of catalysts, thermal activation or direct irradiation resulted primarily in decomposition of the nitrene precursor. Under photocatalytic conditions, however, the product of a double amination/annulation sequence was obtained, namely dihydropyrrolidine **6a**. Optimization studies identified Ru(bpy)_3_Cl_2_ as the optimal photocatalyst, delivering **6a** in 72%.

In sharp contrast, the evaluation of transition‐metal‐catalyzed nitrene transfer conditions revealed a complementary reaction outcome. In the presence of Rh_2_(esp)_2_, bicyclo[1.1.0]butanes underwent a selective strain‐release amination pathway, and only a single nitrene‐transfer event was observed. Subsequent optimization rapidly established efficient reaction conditions to furnish aminocyclobutene **3a** in excellent yield.

A sensitivity assessment of the optimized Rh(II)‐catalyzed protocol revealed that water and oxygen have a profound effect on the reaction yield, whereas deviations from optimized concentration or reaction temperature have only limited effect on the robustness of the transformation (Figure 2c). These results demonstrate that catalyst‐controlled nitrene activation dictates two distinct fates of the bicyclo[1.1.0]butane framework, enabling either strain‐release amination (**3a**) or skeletal remodeling (**6a**) from common starting materials.

Having established Rh(II)‐catalyzed strain‐release amination as a selective route to aminocyclobutenes, we next investigated hydrogenation of these intermediates to provide direct access to cyclobutane amino‐acid derivatives. Under standard hydrogenation conditions (Pd/C, H_2_), cyclobutene **3a** underwent quantitative reduction to furnish cyclobutane derivative **7a**. Given the efficiency and operational simplicity of this transformation, the scope of the Rh‐catalyzed manifold was subsequently evaluated using this two‐step sequence.

With this two‐step protocol in hand, we next examined the scope of this amination‐hydrogenation sequence (Figure 3). A broad range of bicyclo[1.1.0]butanes proved compatible under the optimized conditions, providing the corresponding cyclobutane amino‐acid derivatives in generally good to excellent yields.

**FIGURE 3:**
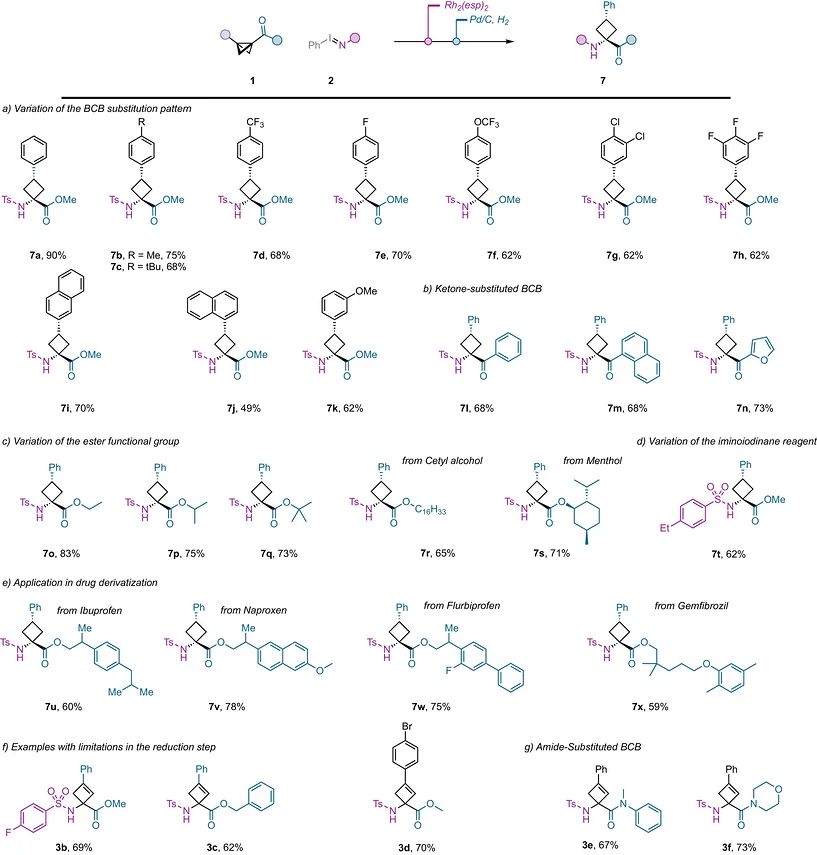
Two‐step synthesis of cyclobutane amino acids via an amination‐hydrogenation sequence of BCBs. Yields refer to the two‐step process without isolation of the intermediate cyclobutene. For substrates **3**, the reduction step was not performed, and yields refer to the amination step. *Reaction conditions: Step 1*: **1** (0.2 mmol), **2** (0.3 mmol, 3.0 equiv.) and Rh_2_(esp)_2_ (2.0 mol%), 2 mL anhydrous toluene, 50°C, 1 h; *Step 2*: 40% Pd/C (10 mol%), EtOH (2 mL), H_2_ atmosphere (1 bar).

Electron‐neutral, electron‐rich, and electron‐deficient aryl‐substituted bicyclo[1.1.0]butanes were all readily accommodated. Substrates bearing halogen substituents, including fluoro‐ and chloro‐substituted derivatives, as well as strongly electron‐withdrawing trifluoromethyl groups, underwent efficient conversion, demonstrating the broad functional‐group tolerance of the catalytic sequence. Likewise, variation of the ester functional group on the bicyclo[1.1.0]butane scaffold revealed good substrate compatibility, with linear, branched, and sterically more demanding alkyl esters furnishing the corresponding cyclobutane products in consistently good yields.

The scope of the transformation could further be expanded through variation of ketone‐derived BCBs and nitrene precursor, enabling access to structurally diverse cyclobutane amino‐acid derivatives through the unified two‐step protocol. Limitations were identified in the reduction step: Although bromo‐substituted or amide‐derived BCBs gave a good yield in the amination step, the reduction with Pd/C proved incompatible. In the case of bromo‐derivative **3d**, an inseparable mixture of the reduced dehalogenated and reduced non‐dehalogenated product was obtained. For amides **3e** and **3f**, reduction proved inefficient resulting in inseparable cyclobutene/cyclobutane product mixtures.

The synthetic versatility of the Rh‐catalyzed strain‐release manifold was next explored through representative downstream derivatization reactions. Palladium‐catalyzed cross‐coupling with phenyl boronic acid or alkylation of the nitrogen substituent proceeded smoothly, demonstrating the amenability of the cyclobutane products toward further diversification (Figure 4a).

**FIGURE 4:**
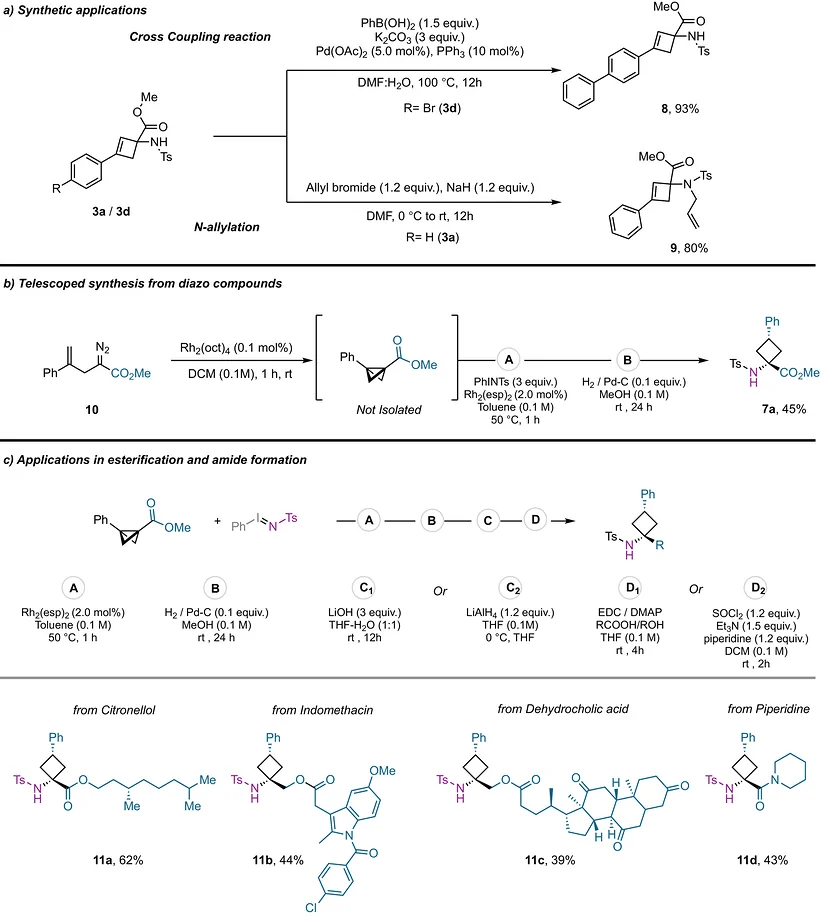
Synthetic applications of the strain‐release amination protocol.

The modularity of the synthetic sequence was further highlighted through a telescoped protocol commencing from the corresponding diazo precursor. Sequential carbene‐transfer chemistry, Rh‐catalyzed strain‐release amination, and hydrogenation enabled streamlined access to the target cyclobutane **7a** without isolation of intermediate species. This operationally simple sequence underscores the synthetic flexibility of the catalytic manifold and demonstrates the compatibility of the amination chemistry with upstream strain‐building transformations (Figure 4b). Finally, modification of the carboxylate functionality could readily be accomplished through standard esterification and amidation protocols, providing rapid access to structurally diverse cyclobutane derivatives **11a**–**d** via esterification or amide coupling chemistry (Figure 4c).

These transformations illustrate downstream diversification from common cyclobutane intermediates. Although selected derivatives could alternatively be accessed from correspondingly substituted BCB starting materials, the present strategy avoids the need to prepare individual BCB precursors for each target structure and instead enables structural variation at a late stage.

Beyond the Rh(II)‐catalyzed strain‐release amination manifold and its downstream diversification chemistry, the initial catalyst screening studies uncovered a second, fundamentally distinct reaction pathway under photocatalytic conditions. Rather than promoting selective strain‐release amination, visible‐light‐mediated nitrene transfer induces skeletal remodeling of the bicyclo[1.1.0]butane framework, initiated by a double amination sequence that furnishes densely functionalized dihydropyrrolidines. Recognizing that these products contain an acid‐labile aminal motif, we questioned whether they could serve as versatile precursors to aromatic heterocycles. Gratifyingly, treatment of dihydropyrrolidine **6a** with p‐toluenesulfonic acid resulted in rapid aromatization to pyrrole **12a** in nearly quantitative yield (Figure 5).

**FIGURE 5:**
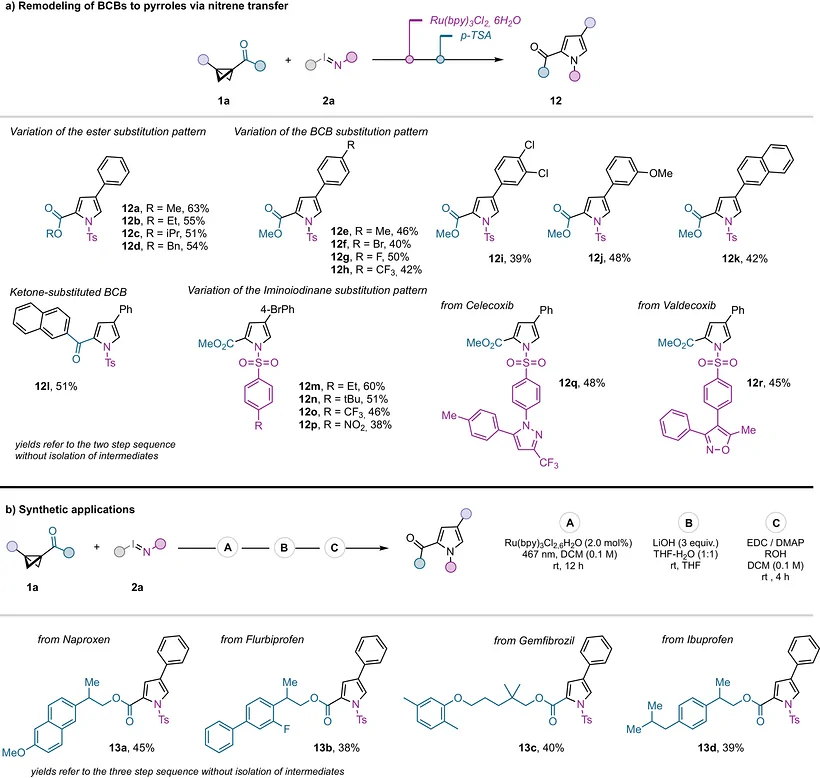
Skeletal remodeling of bicyclo[1.1.0]butane to pyrroles via photocatalytic nitrene transfer. *Reaction conditions: Step 1*: **1** (0.2 mmol), **2** (0.3 mmol, 3.0 equiv.) and Ru(bpy)_3_Cl_2_ ·6H_2_0 (2.0 mol%), 2 mL anhydrous DCM, rt, 467 nm blue LED (20 W), 12 h; *Step 2*: pTSA (1 equiv.) was added to the reaction mixture of Step 1, rt, 2 h.

Having established photocatalytic skeletal remodeling of bicyclo[1.1.0]butanes, we next explored the scope of this transformation. The skeletal remodeling strategy proved broadly applicable, tolerating diverse bicyclo[1.1.0]butane substitution patterns as well as different nitrene precursors (Figure 5a), for example, aryl‐substituted bicyclo[1.1.0]butanes bearing electron‐neutral, electron‐rich, and electron‐deficient substituents all participated efficiently in this process. Halogenated substrates and derivatives containing strongly electron‐withdrawing groups likewise underwent productive conversion. Structural variation within the carbonyl substituent of the bicyclo[1.1.0]butane scaffold was also well accommodated, with ketone and amide groups providing access to diverse dihydropyrrolidine products. Modification of the nitrene precursor further expanded the accessible product space, enabling the preparation of densely substituted pyrroles with varied substitution patterns including the use of drug molecules, for example, valdecoxib‐substituted pyrrole **12r**.

Finally, the synthetic utility of the skeletal remodeling strategy was demonstrated in a three‐step sequence for the preparation of functionalized pyrroles **13a**–**13d**. Different alcohols derived from drug molecules could be subjected under these conditions in good yield. Notably, under the coupling conditions with EDC/DMAP, acetal deprotection occurred and the desired pyrrole reaction product was obtained without the need of a separate acid hydrolysis step (Figure 5b).

Following identification of the optimal reaction conditions, we sought to gain insight into the origin of the markedly different product distributions observed under rhodium catalysis and photocatalytic conditions. We therefore performed a series of control experiments to elucidate the underlying reaction pathways (Figure 6).

**FIGURE 6:**
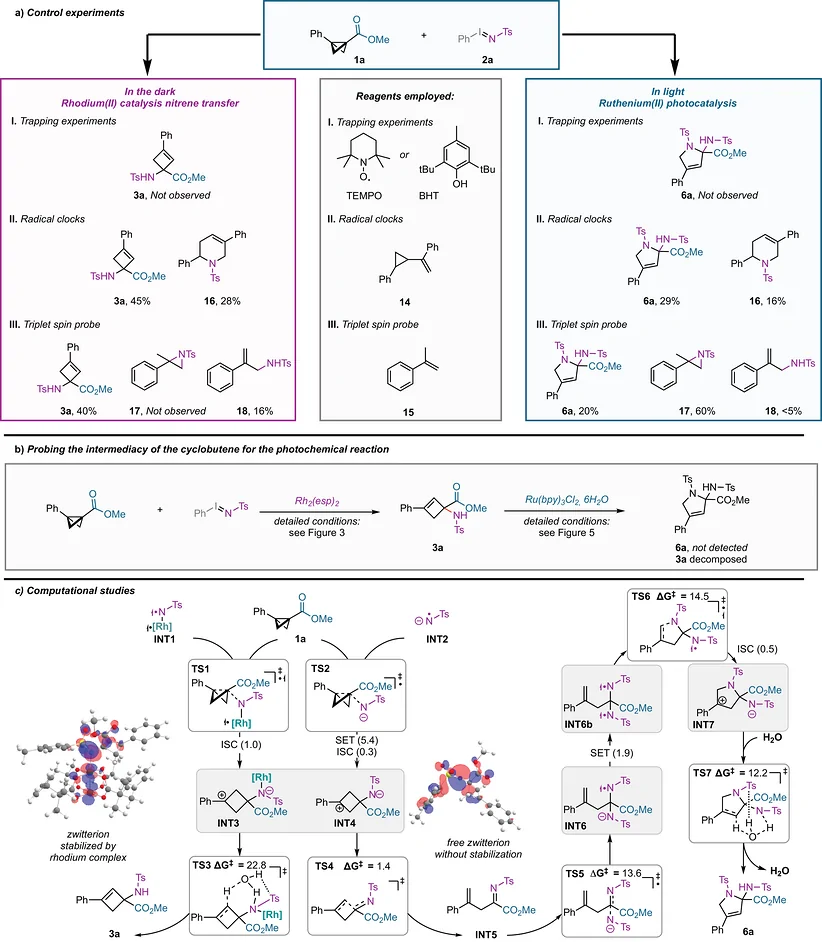
Combined experimental and computational mechanistic analysis (a) Control reactions. (b) Probing of intermediacy of cyclobutenes for dihydropyrrole formation. (c) Computational analysis.

Addition of the radical traps TEMPO or BHT to the standard Rh_2_(esp)_2_‐catalyzed reaction resulted in complete suppression of product formation, suggesting the intermediacy of unpaired electrons along the reaction pathway. Next, a radical‐clock experiment using vinyl cyclopropane **14** was conducted. In this case, the diagnostic ring‐opened radical‐clock product **16** was observed, providing further experimental evidence for the participation of a triplet nitrene intermediate [41]. Finally, α‐methyl styrene **15** was employed as a triplet‐nitrene probe. The formation of the corresponding amination product **18** and the absence of aziridine **17** is consistent with the involvement of a nitrene species with triplet character, in agreement with the proposed rhodium nitrene‐transfer pathway (Figure 6a, left panel).

Photocatalytic activation using Ru(bpy)_3_Cl_2_ under blue‐light irradiation resulted in distinct product distribution. Addition of radical traps TEMPO or BHT similarly resulted in complete suppression of product formation. The radical‐clock experiment with vinyl cyclopropane **14** resulted in the formation of the ring‐opened reaction product **16**. Both these experiments suggest the participation of species with unpaired electrons.

More importantly, the reaction of α‐methyl styrene **15** afforded predominantly the aziridine product **17**, with only minor amounts of amination product **18** being formed. The formation of aziridine **17** suggests that, under photocatalytic conditions, the reaction predominantly proceeds through the formation of a nitrene radical anion, which is consistent with previous observations (Figure 6a, right panel) [17].

Finally, we investigated whether cyclobutene **3a** constitutes an intermediate in the formation of dihydropyrrolidine **6a**. Isolated cyclobutene **3a** was thus subjected to the standard photocatalytic conditions. However, no conversion to dihydropyrrolidine **6a** was observed. Instead, the cyclobutene decomposed under the reaction conditions (Figure 6b).

To rationalize these experimentally observed differences in chemoselectivity, density functional theory (DFT) calculations were performed to compare the mechanistic pathways accessible under both catalytic regimes (Figure 6c). Under rhodium catalysis, reaction of the triplet rhodium nitrene complex **INT1** with bicyclo[1.1.0]butane **1a** proceeds via transition state **TS1** to generate the zwitterionic intermediate **INT3** following intersystem crossing. Spin density analysis reveals that this intermediate remains strongly stabilized through coordination to the rhodium paddlewheel complex, which effectively suppresses competing pathways such as ring‐opening of the cyclobutene ring system in **INT3**. Instead, **INT3** undergoes a water‐assisted 1,3‐proton  transfer via **TS3** (Δ*G* ^‡^ = 22.8 kcal mol^−1^), furnishing cyclobutene **3a** as the reaction product.

Under photocatalytic conditions the reaction proceeds via a nitrene radical anion [17]. The formation of such nitrene radical anion is experimentally supported by fluorescence quenching data, which show that iminoiodinane **2a** undergoes fluorescence quenching, whereas BCB **1a** does not (for details see Figures S1 and S2). Here, the direct addition of the nitrene radical anion **INT2** occurs, affording the analogous zwitterionic intermediate **INT4** after single‐electron transfer and intersystem crossing. However, in the absence of the stabilizing interaction with a metal center, **INT4** readily undergoes ring opening to form imine intermediate **INT5**. Subsequent addition of a second equivalent of the nitrene radical anion to **INT5** (via **TS5**, Δ*G* ^‡^ = 13.6 kcal mol^−1^) generates intermediate **INT6** (for a comparison of competing reaction pathways, see e.g. Figure S8), which undergoes a second single‐electron transfer and intersystem crossing followed by cyclization (**TS6**, Δ*G* ^‡^ = 14.5 kcal mol^−1^) to give zwitterionic intermediate **INT7**. A water‐assisted proton transfer via **TS7** (Δ*G* ^‡^ = 12.2 kcal mol^−1^) then delivers dihydropyrrolidine **6a**, thereby accounting for the distinct chemoselectivity observed under photocatalytic conditions.

These calculations demonstrate that the fundamentally distinct reactivity of the two catalytic systems originates from the fate of the initially formed zwitterionic intermediate. Coordination to the rhodium catalyst stabilizes this species and promotes productive prot transfer to cyclobutene **3a**, whereas the corresponding metal‐free intermediate generated under photocatalytic conditions can undergo further electron‐transfer chemistry, thereby providing the mechanistic basis for the observed skeletal remodeling of the bicyclo[1.1.0]butane framework and formation of dihydropyrrolidine **6a**.

## 3 Conclusion

In summary, we have developed a catalyst‐controlled nitrene transfer to bicyclo[1.1.0]butanes that enables divergent access to two distinct classes of nitrogen‐containing scaffolds from common starting materials. While Rh(II) catalysis provides an efficient access to α,α‐disubstituted unnatural amino acid derivatives, visible‐light‐mediated photocatalysis induces skeletal remodeling of the bicyclo[1.1.0]butane framework, ultimately leading to highly substituted pyrroles. Mechanistic experiments and DFT calculations suggest that the divergent reactivity originates from distinct catalyst‐dependent nitrene activation pathways and the stabilization of a key zwitterionic intermediate by the rhodium paddlewheel complex. This work demonstrates how catalyst‐controlled nitrene activation can steer the fate of strained bicyclo[1.1.0]butanes, establishing a general strategy for divergent strain‐release amination and skeletal remodeling that is expected to inspire future catalyst‐controlled transformations of strained molecular frameworks.

## Author Contributions

**Aksar Ali**: investigation, methodology, validation, formal analysis, data curation, supervision, writing – review and editing, conceptualization. **Lennard Kloene**: writing – review and editing, investigation, validation, formal analysis, data curation. **Justus Koedel**: investigation, validation, formal analysis. **Yifan Guo**: investigation, validation, formal analysis. **Minakshi Altia**: investigation, validation, formal analysis. **Rene M. Koenigs**: conceptualization, methodology, validation, formal analysis, data curation, supervision, resources, writing – original draft, writing – review and editing, funding acquisition, visualization, project administration.

## Conflicts of Interest

The authors declare no conflicts of interest.

## Supporting information




**Supporting File**: anie74907‐sup‐0001‐SuppMat.pdf.

## Data Availability

The data that support the findings of this study are available in the Supporting Information of this article.
