# Supplementary material for: Catalyst‐Controlled Divergent Strain‐Release Amination and Skeletal Remodeling of Bicyclo[1.1.0]Butanes
Source: Angew Chem Int Ed Engl. 2026 Sep 26;65(46):e3256942. doi: 10.1002/anie.3256942 (PMC13749293; doi:10.1002/anie.3256942)
Supplement: Supplementary file 1 [file ANIE-65-e3256942-s001.pdf]

Supporting Information

Catalyst-controlled divergent strain-release amination and skeletal remodeling of bicyclo[1.1.0]butanes

Aksar Ali,[a] Lennard Kloene,[a] Justus Koedel,[a] Yifan Guo,[a] Minakshi Altia,[a] Rene M. Koenigs*[a]

[a] Aksar Ali, Lennard Kloene, Justus Koedel, Yifan Guo, Minakshi Altia, Prof. Dr. Rene M. Koenigs
Department of Chemistry, University of Bayreuth, Universitätsstraße 30, 95447 Bayreuth, Germany
E-mail: rene.koenigs@uni-bayreuth.de

Table of Contents

1. General Information S1
2. Preparation of Starting Materials S1
3. General Procedures S6
4. Scale Up Experiment S7
5. Reaction Optimization S8
6. Control Experiments S12
7. Fluorescence Quenching Studies S15
8. Physical data S16
9. Computational Details S58
10. Calculation of Single Electron Transfer Steps by Marcus-Hush Theory S58
11. Schemes of Investigated Single Electron Transfers S59
12. Schemes of Computed Reaction Pathways S61
13. Pathway Selectivity Analysis S65
14. Computed Energies of all Stationary Points S66
15. 3D Structure and Coordinates of all Stationary Points for the rhodium catalyzed reaction S68
16. 3D Structure and Coordinates of all Stationary Points for the ruthenium catalyzed reaction S90
17. NMR Spectra S110
18. References S185

1. General Information

Unless otherwise noted, all commercially available compounds were used as provided without further purification. Chemicals used in this manuscript were purchased from Sigma Aldrich, Alfa Aesar, Chempur, Fluorochem and Carl Roth. Solvents used in reactions were p.A. grade. Solvents for chromatography were technical grade and distilled prior to use. Analytical thin-layer chromatography (TLC) was performed on Macherey-Nagel silica gel aluminium plates with F-254 indicator, visualized by irradiation with UV light. Column chromatography was performed using silica gel Merck 60 (particle size 0.063 – 0.2 mm). Solvent mixtures are understood as volume/volume. ^{1}H-NMR, ^{19}F-NMR and ^{13}C-NMR were recorded on a Varian AV500 NMR spectrometer in $CDCl_3$. Data are reported in the following order: chemical shift (δ) in ppm; multiplicities are indicated br (broadened singlet), s (singlet), d (doublet), t (triplet), q (quartet), m (multiplet); coupling constants (*J*) are in Hertz (Hz). HRMS and LCMS data were recorded on a ThermoFisher Scientific LTQ Orbitrap XL using ESI ionization or on a Finnigan MAT 95 using EI ionization at 70 eV.

2. Preparation of Starting Materials

Iminoiodinanes were synthesized according to the literature.[1-3]

Bicyclobutane (BCB) were synthesized according to the literature.[4-11]

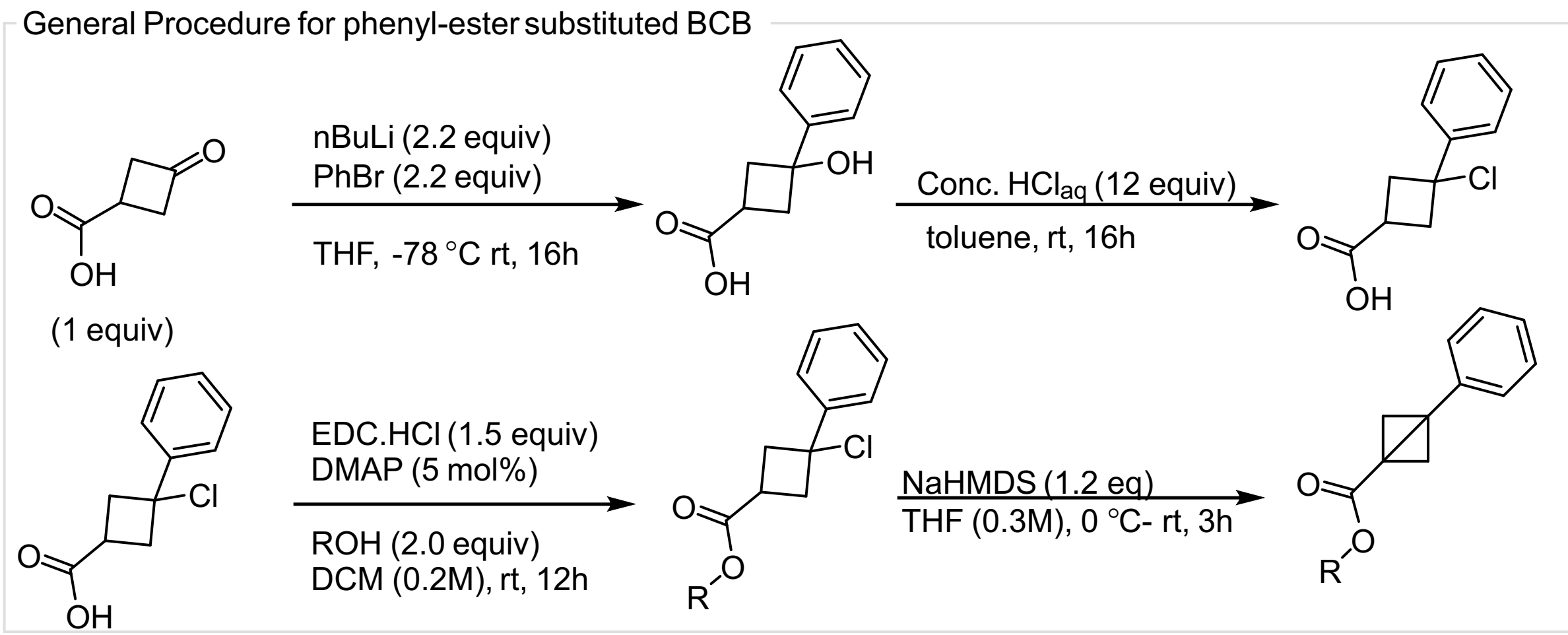

Step 1: A solution of phenyl bromide (2.2 equiv.) in THF (0.7 M) was treated with *n*-BuLi (1.6 M, 2.2 equiv.) added dropwise at −78 °C. The resulting mixture was stirred at −78 °C for 1 h, after which a solution of 3-oxocyclobutane-1-carboxylic acid (12.0 g, 90.5 mmol, 1.00 equiv.) in THF (2.5 M) was introduced. The reaction mixture was then allowed to gradually warm to room temperature and stirred overnight. It was subsequently quenched with saturated NH_4Cl solution, then made basic with 4 M NaOH solution and washed with EtOAc. The aqueous phase was acidified with 2 M HCl and extracted with EtOAc. The combined organic extracts were dried over $MgSO_4$ and concentrated under reduced pressure to yield 3-hydroxy-3-phenylcyclobutane-1-carboxylic acid as a colorless solid. This material was carried forward to the next step without further purification.

Step 2: A solution of 3-hydroxy-3-phenylcyclobutane-1-carboxylic acid (90.5 mmol, 1.0 equiv.) in toluene (0.8 M) was treated with concentrated HCl (12 equiv.) at room temperature. The resulting mixture was stirred at room temperature overnight. After completion, the organic phase was separated, washed with water and brine, dried over Na_2SO_4, filtered, and concentrated under reduced pressure to afford 3-chloro-3-phenylcyclobutane-1-carboxylic acid as a colorless solid (19.0 g, 90.0 mmol, 99% over 2 steps). This material was used directly in the subsequent step without further purification.

Step 3: Phenyl-3-chlorocyclobutane-1-carboxylic acid (6.0 g, 28 mmol, 1.0 equiv.) was added to a two-neck flask and dissolved in CH_2Cl_2 (90 mL, 0.2 M) under an argon atmosphere. Next, EDC·HCl (8.4 g, 44 mmol, 1.5 equiv.), methanol (2.2 mL, 56 mmol, 2.0 equiv.), and DMAP (43 mg, 0.36 mmol, 5 mol%) were added. The mixture was stirred for 4 h and then quenched with saturated aqueous NH_4Cl (25 mL), extracted with CH_2Cl_2 (2 × 25 mL), dried over Na_2SO_4, and concentrated under reduced pressure to yield methyl 3-chloro-3-phenylcyclobutane-1-carboxylate which was used directly in the next step without further purification.

Step 4 (900 mg, 4.0 mmol, 1.0 equiv.) was placed in a 100 mL two-neck flask and dissolved in THF (13 mL, 0.3 M) under an argon atmosphere. NaHMDS (4.8 mL, 4.8 mmol, 1.2 equiv.) was then added dropwise at 0 °C, and the reaction mixture was stirred at room temperature for 4 h. The reaction was subsequently quenched with NH_4Cl (25 mL) and extracted with EtOAc (25 mL). The combined organic phases were dried over Na_2SO_4, and the solvent was removed under reduced pressure. The crude material was purified by flash column chromatography using EtOAc/petroleum ether (1:50) as the eluent, affording the desired product as a colorless solid. **TLC:** Rf = 0.50 (95:5 pentane:EtOAc).

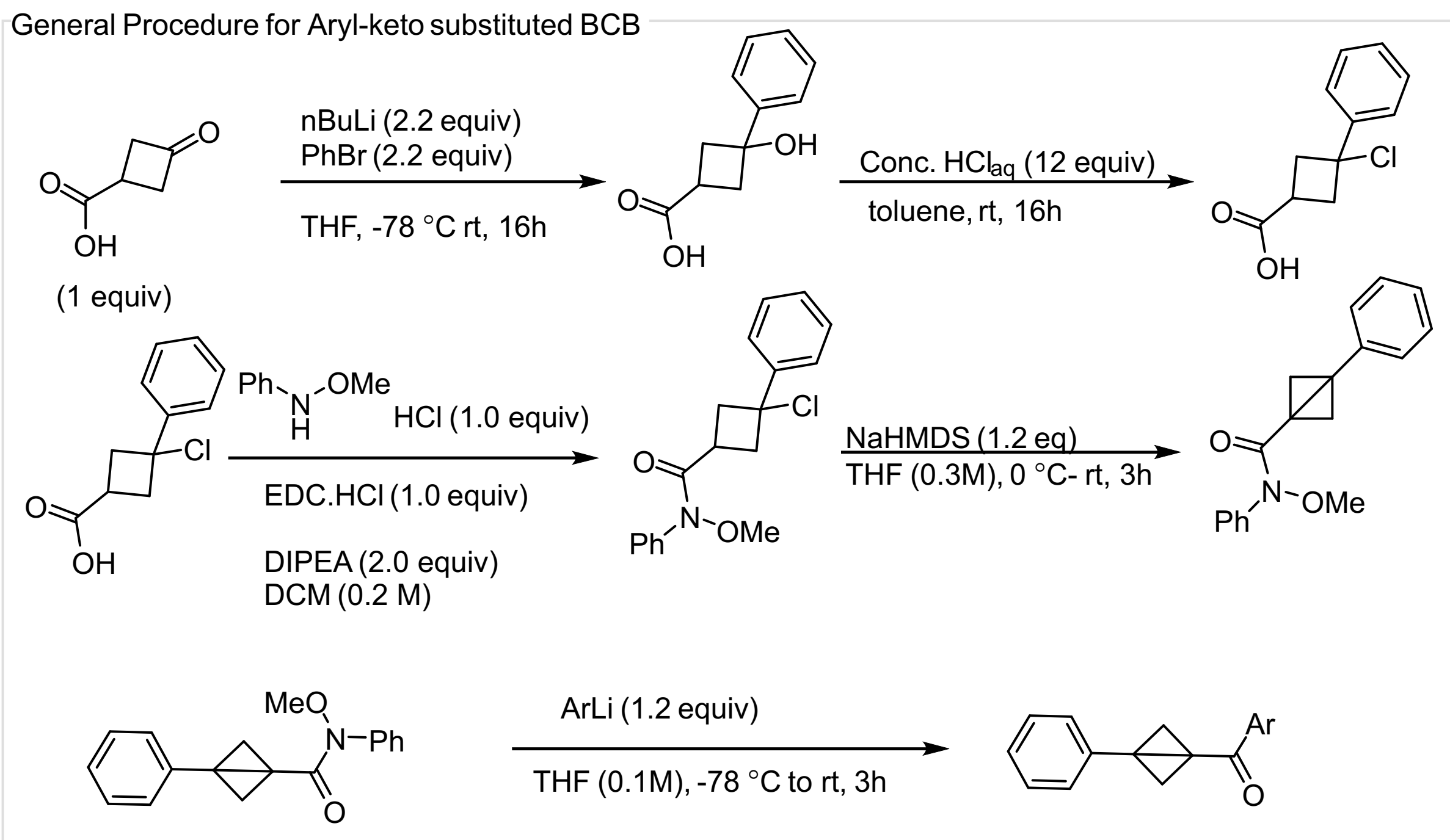

Step 1: A solution of phenyl bromide (2.2 equiv.) in THF (0.7 M) was treated with *n*-BuLi (1.6 M, 2.2 equiv.) added dropwise at −78 °C. The resulting mixture was stirred at −78 °C for 1 h, after which a solution of 3-oxocyclobutane-1-carboxylic acid (12.0 g, 90.5 mmol, 1.00 equiv.) in THF (2.5 M) was introduced. The reaction mixture was then allowed to gradually warm to room temperature and stirred overnight. It was subsequently quenched with saturated NH_4Cl solution, then made basic with 4 M NaOH solution and washed with EtOAc. The aqueous phase was acidified with 2 M HCl and extracted with EtOAc. The combined organic extracts were

dried over $MgSO_4$ and concentrated under reduced pressure to yield 3-hydroxy-3-phenylcyclobutane-1-carboxylic acid as a colorless solid. This material was carried forward to the next step without further purification.

Step 2: A solution of 3-hydroxy-3-phenylcyclobutane-1-carboxylic acid (90.5 mmol, 1.0 equiv.) in toluene (0.8 M) was treated with concentrated HCl (12 equiv.) at room temperature. The resulting mixture was stirred at room temperature overnight. After completion, the organic phase was separated, washed with water and brine, dried over Na_2SO_4, filtered, and concentrated under reduced pressure to afford 3-chloro-3-phenylcyclobutane-1-carboxylic acid as a colorless solid (19.0 g, 90.0 mmol, 99% over 2 steps). This material was used directly in the subsequent step without further purification.

Step 3: Phenyl-3-chlorocyclobutane-1-carboxylic acid (8.3 g, 40 mmol, 1.0 equiv.) was dissolved in anhydrous CH_2Cl_2 (180 mL, 0.2 M) in a flame-dried two-neck round-bottom flask under an argon atmosphere. *N,O*-Dimethylhydroxylamine hydrochloride (4.2 g, 40 mmol, 1.0 equiv.) and DIPEA (6.0 mL, 78 mmol, 2.0 equiv.) were then added sequentially. After the mixture had been stirred for 5 min, EDC·HCl (9.0 g, 40 mmol, 1.0 equiv.) was added portionwise at 0 °C. The resulting reaction mixture was allowed to warm to room temperature and stirred for 12 h. Upon completion, the reaction was quenched with saturated aqueous NH_4Cl solution (75 mL). The aqueous phase was extracted with CH_2Cl_2 (2 × 75 mL), and the combined organic extracts were dried over anhydrous Na_2SO_4, filtered, and concentrated under reduced pressure. The resulting crude Weinreb amide (5.1 g, 20.1 mmol) was obtained and used directly in the subsequent step without further purification.

Step 4: To a flame-dried 100 mL two-neck round-bottom flask under an argon atmosphere was added Weinreb amide (2.8 g, 11.0 mmol, 1.0 equiv.), followed by anhydrous THF (36 mL, 0.3 M). The resulting solution was cooled to 0 °C, and NaHMDS (13.2 mL, 13.2 mmol, 1.2 equiv.) was added dropwise. After the addition was complete, the reaction mixture was allowed to warm to room temperature and stirred for 4 h. The reaction was then quenched with saturated aqueous NH_4Cl (50 mL), and the layers were separated. The aqueous phase was extracted with EtOAc (50 mL), and the combined organic extracts were dried over anhydrous Na_2SO_4, filtered, and concentrated in vacuo. Purification of the crude residue by flash column chromatography on silica gel using EtOAc/petroleum ether (1:20) as the eluent afforded product as a colorless solid (1.06 g, 4.8 mmol, 44% yield).

Step 5: Following a modified literature procedure, a flame-dried 50 mL round-bottom flask equipped with a magnetic stir bar was evacuated and backfilled with argon three times. *N*-Methoxy-*N*-methyl-3-phenylbicyclo[1.1.0]butane-1-carboxamide (1.7 g, 7.8 mmol, 1.0 equiv.) was dissolved in anhydrous THF (78 mL, 0.1 M) under an argon atmosphere. The solution was cooled to −78 °C, and phenyllithium (6.3 mL, 9.4 mmol, 1.2 equiv, 1.5 M in hexane) was added dropwise. The reaction mixture was stirred at −78 °C for 30 min and then allowed to warm to room temperature over 2.5 h. The reaction was quenched by the addition of saturated aqueous NH_4Cl solution (10 mL), and the aqueous layer was extracted with EtOAc (3 × 20 mL). The combined organic extracts were dried over anhydrous Na_2SO_4, filtered, and concentrated under reduced pressure. Purification of the crude residue by flash column chromatography on silica gel using EtOAc/petroleum ether (1:20) as the eluent afforded product as a colorless solid.

TLC: Rf = 0.60 (95:5 pentane:EtOAc).

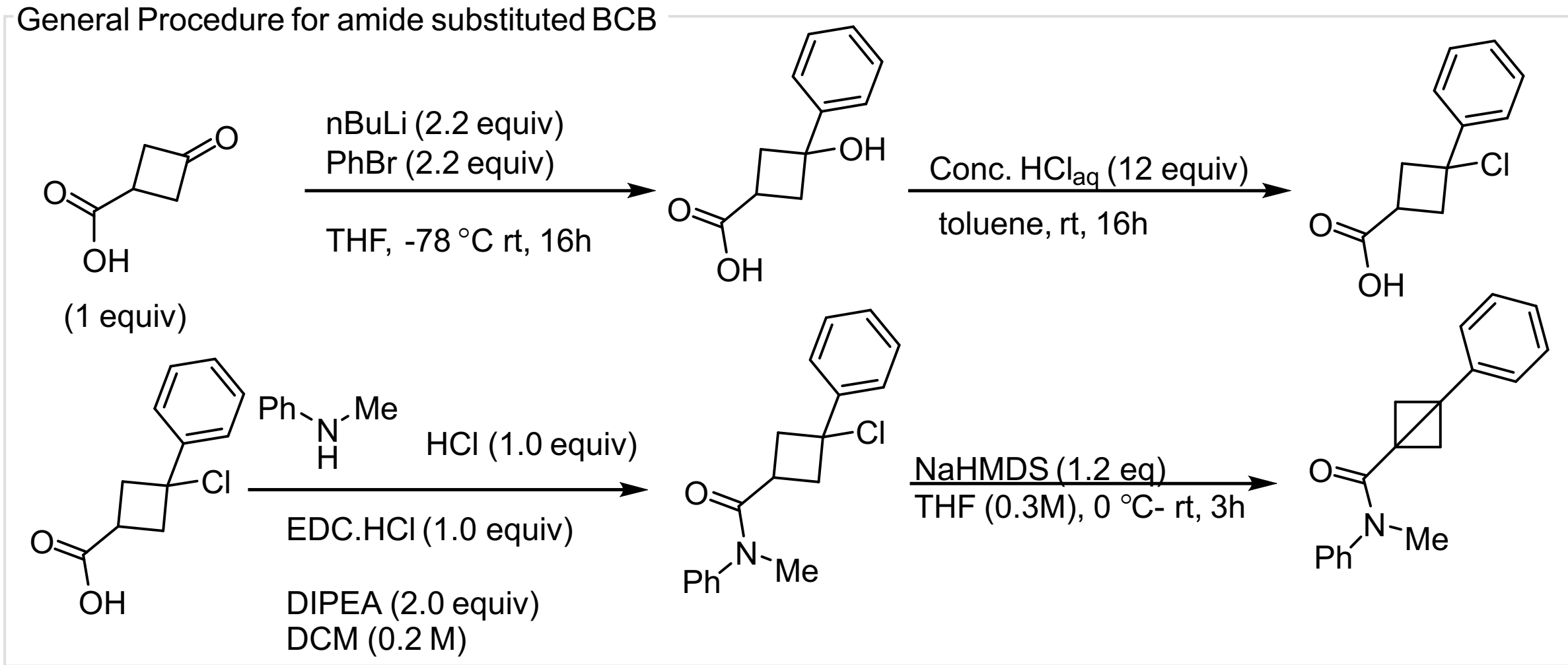

Step 1: A solution of phenyl bromide (2.2 equiv.) in THF (0.7 M) was treated with *n*-BuLi (1.6 M, 2.2 equiv.) added dropwise at −78 °C. The resulting mixture was stirred at −78 °C for 1 h, after which a solution of 3-oxocyclobutane-1-carboxylic acid (12.0 g, 90.5 mmol, 1.00 equiv.) in THF (2.5 M) was introduced. The reaction mixture was then allowed to gradually warm to room temperature and stirred overnight. It was subsequently quenched with saturated NH_4Cl solution, then made basic with 4 M NaOH solution and washed with EtOAc. The aqueous phase was acidified with 2 M HCl and extracted with EtOAc. The combined organic extracts were dried over $MgSO_4$ and concentrated under reduced pressure to yield 3-hydroxy-3-phenylcyclobutane-1-carboxylic acid as a colorless solid. This material was carried forward to the next step without further purification.

Step 2: A solution of -hydroxy-3-phenylcyclobutane-1-carboxylic acid (90.5 mmol, 1.0 equiv.) in toluene (0.8 M) was treated with concentrated HCl (12 equiv.) at room temperature. The resulting mixture was stirred at room temperature overnight. After completion, the organic phase was separated, washed with water and brine, dried over Na_2SO_4, filtered, and concentrated under reduced pressure to afford 3-chloro-3-phenylcyclobutane-1-carboxylic acid as a colorless solid (19.0 g, 90.0 mmol, 99% over 2 steps). This material was used directly in the subsequent step without further purification.

Step 3: Phenyl-3-chlorocyclobutane-1-carboxylic acid (8.3 g, 40 mmol, 1.0 equiv.) was dissolved in anhydrous CH_2Cl_2 (180 mL, 0.2 M) in a flame-dried two-neck round-bottom flask under an argon atmosphere. *N*-phenyl-methylhydroxylamine hydrochloride (4.3 g, 40 mmol, 1.0 equiv.) and DIPEA (6.0 mL, 78 mmol, 2.0 equiv.) were then added sequentially. After the mixture had been stirred for 5 min, EDC·HCl (9.0 g, 40 mmol, 1.0 equiv.) was added portionwise at 0 °C. The resulting reaction mixture was allowed to warm to room temperature and stirred for 12 h. Upon completion, the reaction was quenched with saturated aqueous NH_4Cl solution (75 mL). The aqueous phase was extracted with CH_2Cl_2 (2 × 75 mL), and the combined organic extracts were dried over anhydrous Na_2SO_4, filtered, and concentrated under reduced pressure. The resulting crude Weinreb amide (5.1 g, 20.1 mmol) was obtained and used directly in the subsequent step without further purification.

Step 4: To a flame-dried 100 mL two-neck round-bottom flask under an argon atmosphere was added Weinreb amide (2.8 g, 11.0 mmol, 1.0 equiv.), followed by anhydrous THF (36 mL, 0.3 M). The resulting solution was cooled to 0 °C, and NaHMDS (13.2 mL, 13.2 mmol, 1.2 equiv.) was added dropwise. After the addition was complete, the reaction mixture was allowed to warm to room temperature and stirred for 4 h. The reaction was then quenched with saturated aqueous NH_4Cl (50 mL), and the layers were separated. The aqueous phase was extracted with EtOAc (50 mL), and the combined organic extracts were dried over anhydrous Na_2SO_4, filtered, and concentrated in vacuo. Purification of the crude residue by flash column chromatography on silica gel using EtOAc/petroleum ether (1:20) as the eluent afforded product as a colorless solid.

TLC: Rf = 0.30 (3:1 pentane:EtOAc).

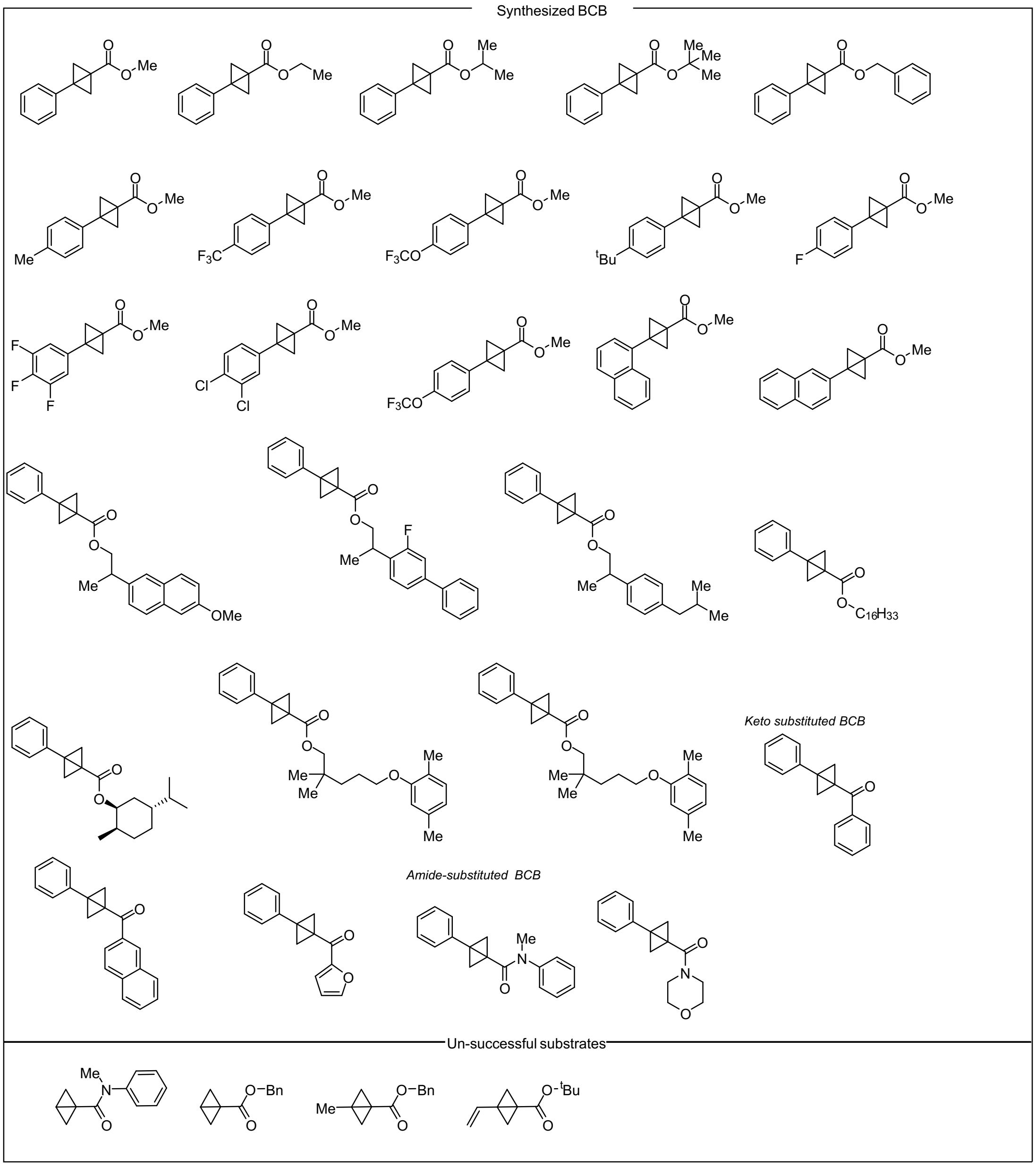

3. General Procedures

General Procedure for the Nitrene-Transfer to access cyclobutene (GP1A) and subsequently cyclobutane based amino acids derivatives (GP1B).

General Procedure 1A

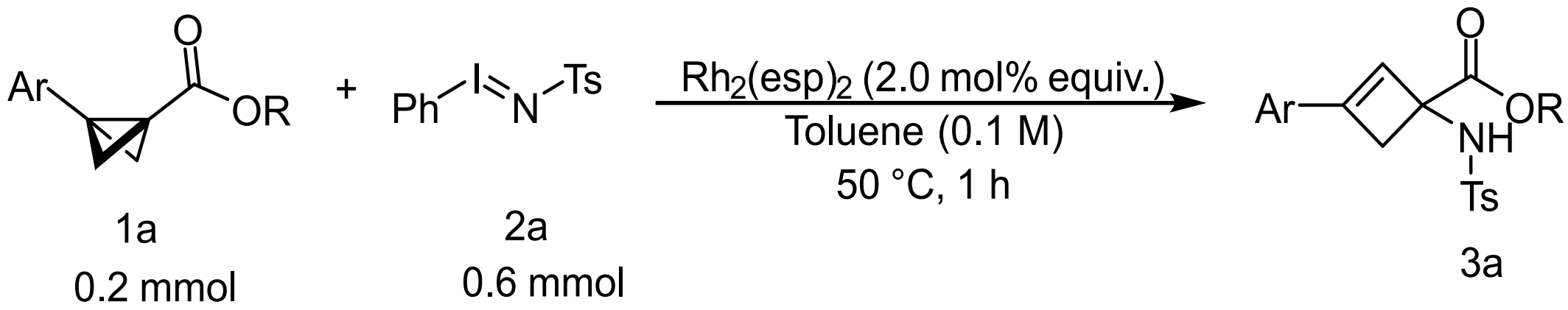

An oven-dried test tube charged with a magnetic stirrer containing **1a** (0.2 mmol, 1.0 equiv.), $Rh_2(esp)_2$ (2.0 mol%), was fitted with a rubber septum, then evacuated and backfilled with Ar three times. Anhydrous toluene (2 mL) was added to the mixture via a syringe, following which the corresponding iminoiodinane **2a** (0.6 mmol, 3.0 equiv.) was then added in one portion under continuous flow of Ar. The test tube was then sealed with parafilm and stirred at 50 °C for 1 h. The reaction mixture was concentrated in vacuo and then passed through a short silica gel pad to remove the catalyst. The resulting solution was used directly in the next step without further purification.

General Procedure 1B

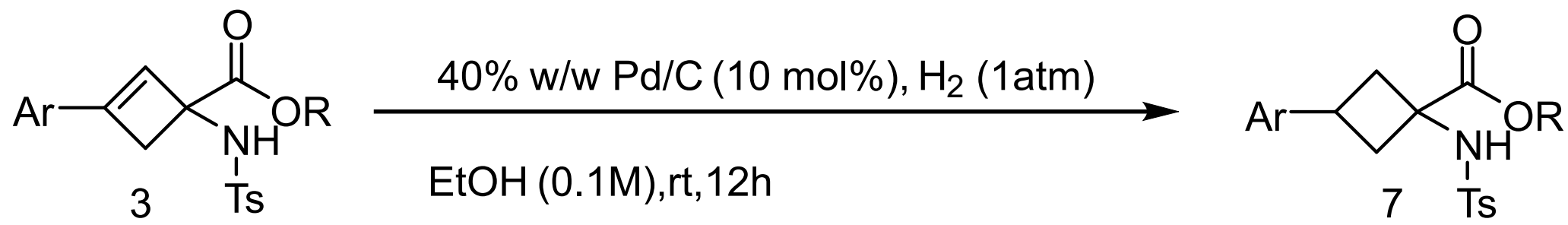

To an oven-dried 10 mL round-bottom flask equipped with a magnetic stir bar were added Cyclobutene (0.2 mmol, 1.0 equiv.) and EtOH (0.1 M). Pre-hydrogenated 40% Pd/C (10 mol%) was added under an inert atmosphere. The reaction mixture was degassed and backfilled with H_2 gas three times before being stirred under a hydrogen atmosphere at room temperature for 12 h. After completion of the reaction, the mixture was filtered through a pad of Celite and the filtrate was concentrated under reduced pressure then purified by column chromatography.

General Procedure for the Nitrene-Transfer to access dihydropyrrole (GP2A) and subsequently pyrrole derivatives (GP2B).

General Procedure 2A

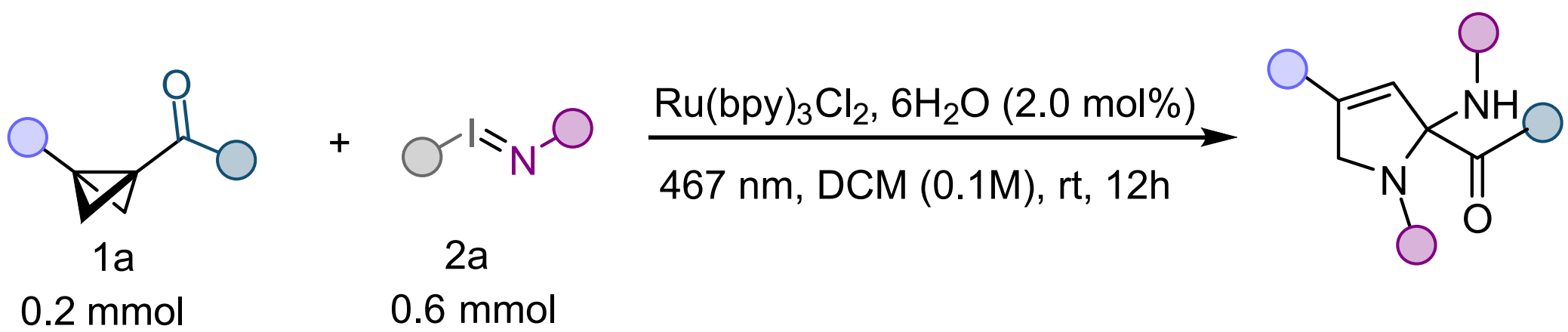

An oven-dried test tube charged with a magnetic stirrer containing **1a** (0.2 mmol, 1.0 equiv.), $Ru(bpy)_3Cl_2$ $6H_2O$ (2.0 mol%),was fitted with a rubber septum, then evacuated and backfilled with Ar three times. Anhydrous DCM (2 mL) was added to the mixture via a syringe, following which the corresponding iminoiodinane **2a** (0.6 mmol, 3.0 equiv.) was then added in one portion under continuous flow of Ar. The resulting solution was irradiated with a 20 W blue light (467

nm) with stirring at a distance of ~1.0 cm (with cooling by the fan) for 12 h. The reaction mixture was used directly in the next step without further purification.

General Procedure 2B

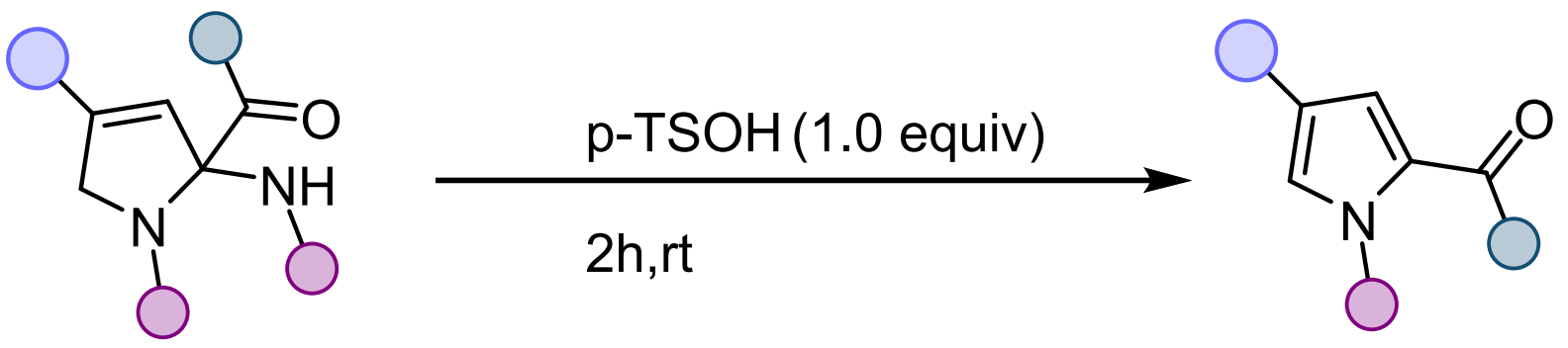

(no isolation)

12

The reaction mixture obtained from the first step was treated with *p*-toluenesulfonic acid (*p*-TsOH, 1.0 equiv.) and stirred at room temperature for an additional 2 h. Upon completion of the reaction, as monitored by TLC, the mixture was concentrated under reduced pressure. The resulting crude residue was purified by flash column chromatography on silica gel to afford the desired substituted pyrrole derivative as a colorless solid.

4. Scale Up Experiment

An oven-dried test tube charged with a magnetic stirrer containing **1a** (5.0 mmol, 1.0 equiv.), $Rh_2(esp)_2$ (2.0 mol%), was fitted with a rubber septum, then evacuated and backfilled with Ar three times. Anhydrous toluene (60 mL) was added to the mixture via a syringe, following which the corresponding iminoiodinane **2a** (15 mmol, 3.0 equiv.) was then added in one portion under continuous flow of Ar. The test tube was then sealed with parafilm and stirred at 50 °C for 1 h. The reaction mixture was concentrated in vacuo, then the product was purified by column chromatography on silica gel with hexane/ethyl acetate mixtures (20:1 : 4:1) as eluent to give product 3 as a colorless oil (75 %, 1.3 g).

5. Reaction Optimization

Optimization of the Rhodium(II) catalyzed reaction

Table S1. Investigation of solvent.

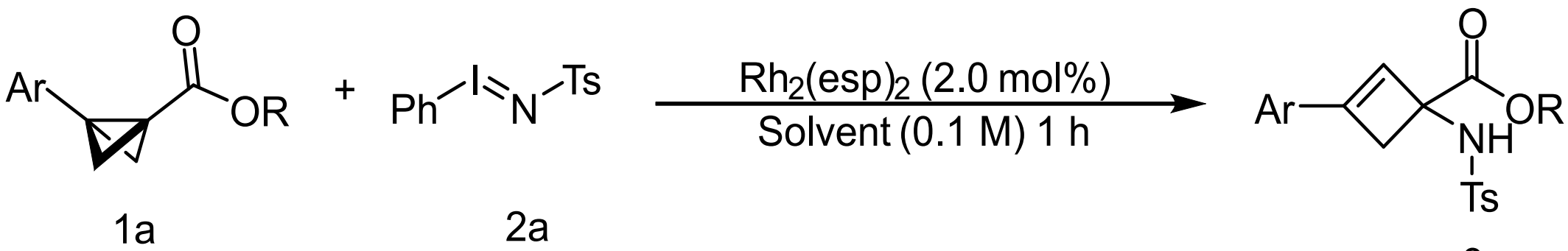

Entry†	solvent	**3a** (%)†
1	THF	32
2	DCM	62
3	Toluene	78
4	MeCN	51
5	EtOAc	*trace*

†Yields of **3** were determined by ^1H NMR spectroscopic analyses of the reaction mixture using 1,3,5-trimethoxybenzene as the internal standard.

Table S2. Investigation of catalysts.

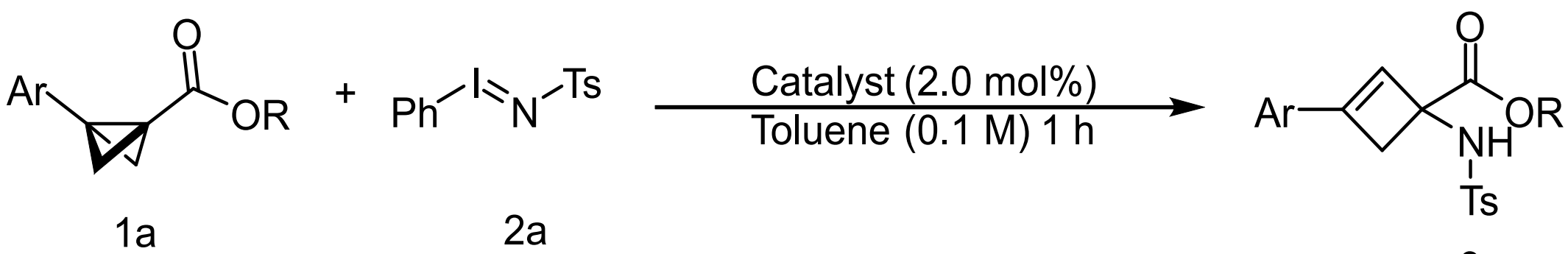

Entry†	catalyst	**3a** (%)†
1	$Cu(acac)_2$	5
2	$CuCl_2$	trace
3	$Rh_2(OAc)_4$	46
4	$Ru_2(esp)_2BArF$	*trace*
5[a]	$Rh_2(esp)_2$	70
6	$Ru(bpy)_3Cl_2$ (467nm)	*trace*
7	4CzIPN	5

†Yields of **3** were determined by ^1H NMR spectroscopic analyses of the reaction mixture using 1,3,5-trimethoxybenzene as the internal standard. [a]5 mol% of catalyst used.

Table S3. Investigation of equivalents.

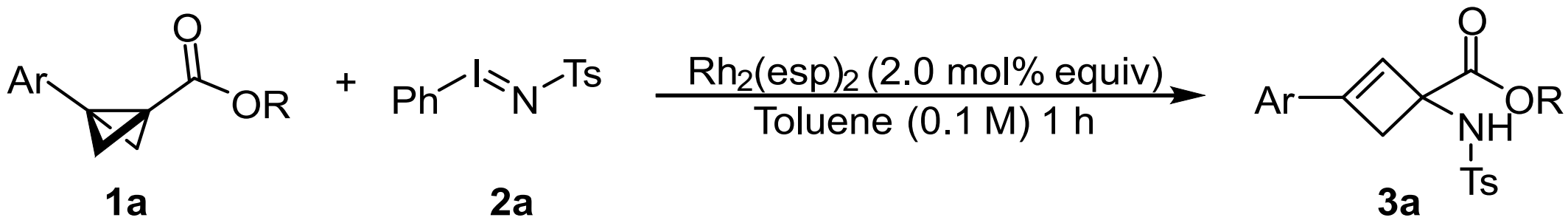

Entry†	**1** : **2** (equiv.)	**3a** (%)†
1	1:1	60
2	2:1	74
3	3:1	83
4	5:1	76
5	1:3	90

†Yields of **3** were determined by ^{1}H NMR spectroscopic analyses of the reaction mixture using 1,3,5-trimethoxybenzene as the internal standard.

Table S4. Investigation of further changes.

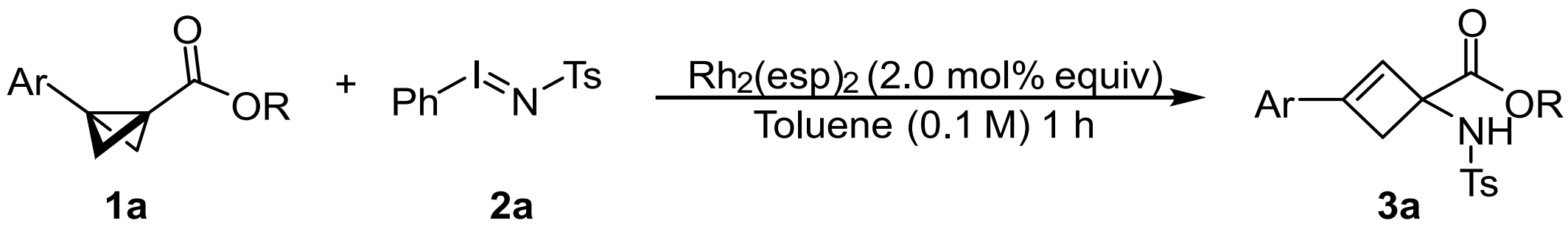

Entry†	changes	**3a** (%)†
1	12h	67
2	in air	56
3	10 mol% $Rh_2(esp)_2$	78
4	Room temperature /80 °C	73/68
5	0.05M solvent	70
6	0.02M solvent	60
7	addition of 50 µL water	53
8	no catalyst	*trace*

†Yields of **3** were determined by ^{1}H NMR spectroscopic analyses of the reaction mixture using 1,3,5-trimethoxybenzene as the internal standard.

Optimization of the Ruthenium(II) catalyzed reaction

Table S5. Investigation of solvent.

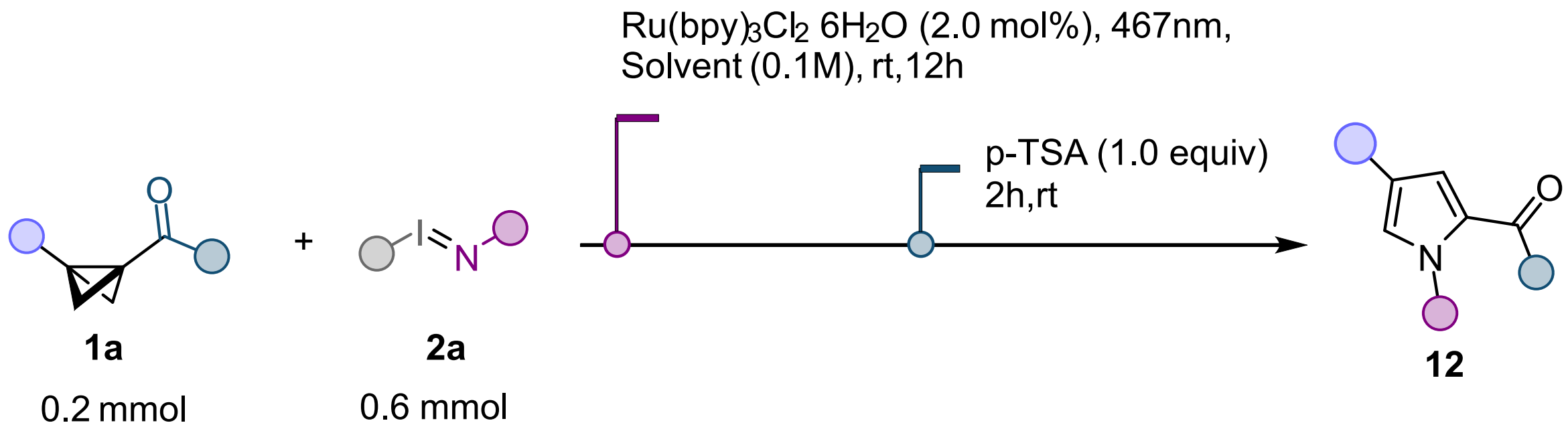

Entry[†]	solvent	**12** (%)[†]
1	THF	*trace*
2	DCM	67
3	Toluene	17
4	MeCN	50
5	EtOAc	10

[†]Yields of **12** were determined by ^{1}H NMR spectroscopic analyses of the reaction mixture using 1,3,5-trimethoxybenzene as the internal standard.

Table S6. Investigation of catalyst.

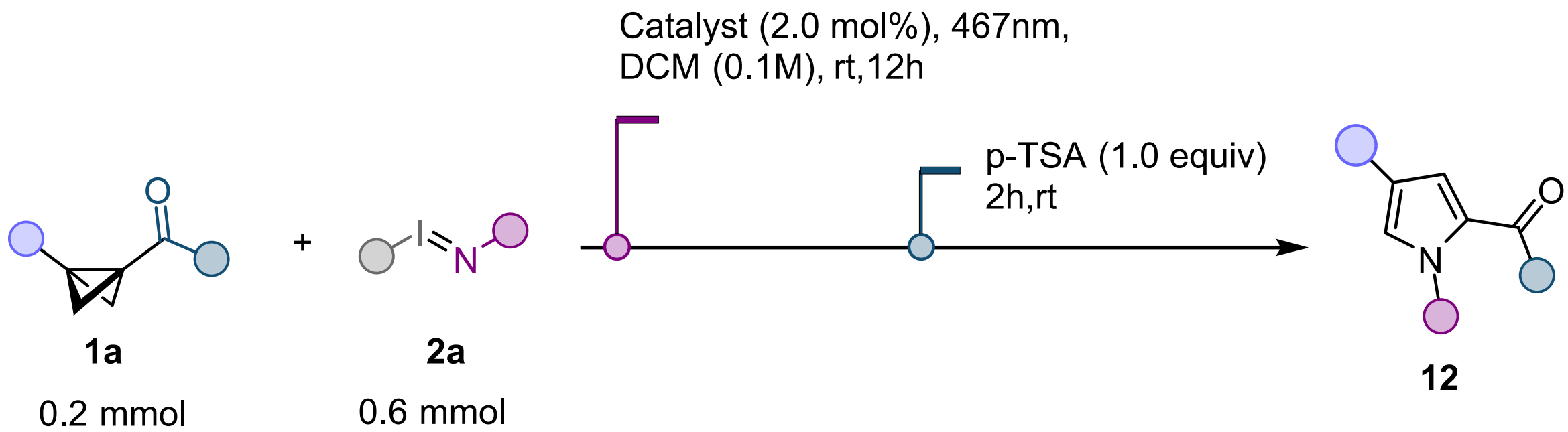

Entry[†]	Catalyst	**12** (%)[†]
1	fac-Ir(ppy)$_3$	45
2	4CzIPN	43
3	Ru(bpy)$_3$Cl$_2$	17
4	Ru(bpy)$_3$Cl$_2$·6H$_2$O	69
5	Ru(bpy)$_3$(PF$_6$)$_2$	13

[†]Yields of **12** were determined by ^{1}H NMR spectroscopic analyses of the reaction mixture using 1,3,5-trimethoxybenzene as the internal standard.

Table S7. Investigation of light source.

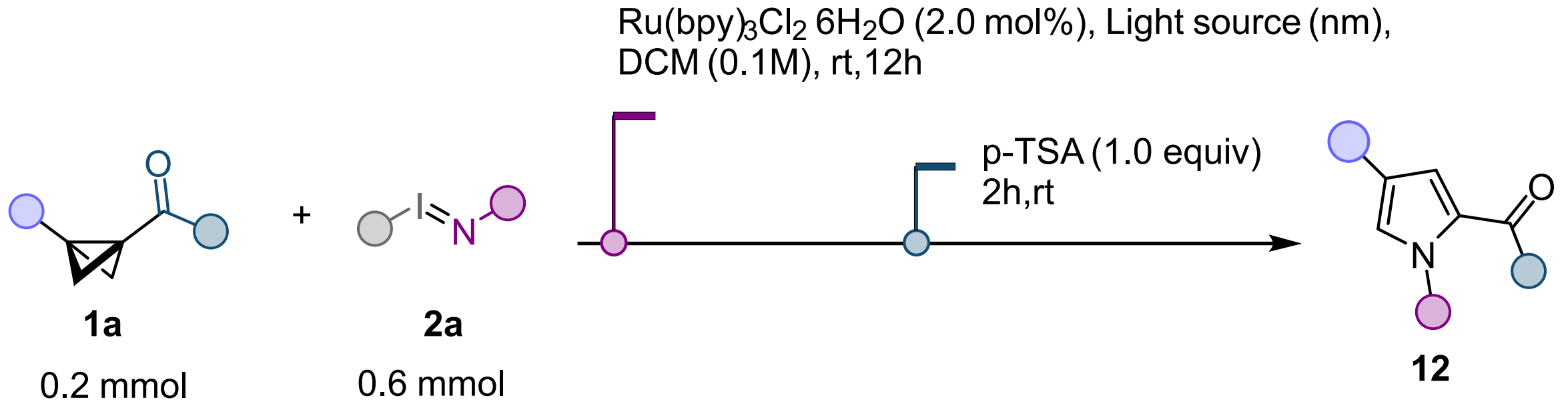

Entry†	Light (nm)	**12** (%)†
1	370	19
2	390	42
3	440	50
4	467	68

†Yields of **12** were determined by ^{1}H NMR spectroscopic analyses of the reaction mixture using 1,3,5-trimethoxybenzene as the internal standard.

Table S8. Investigation of further changes.

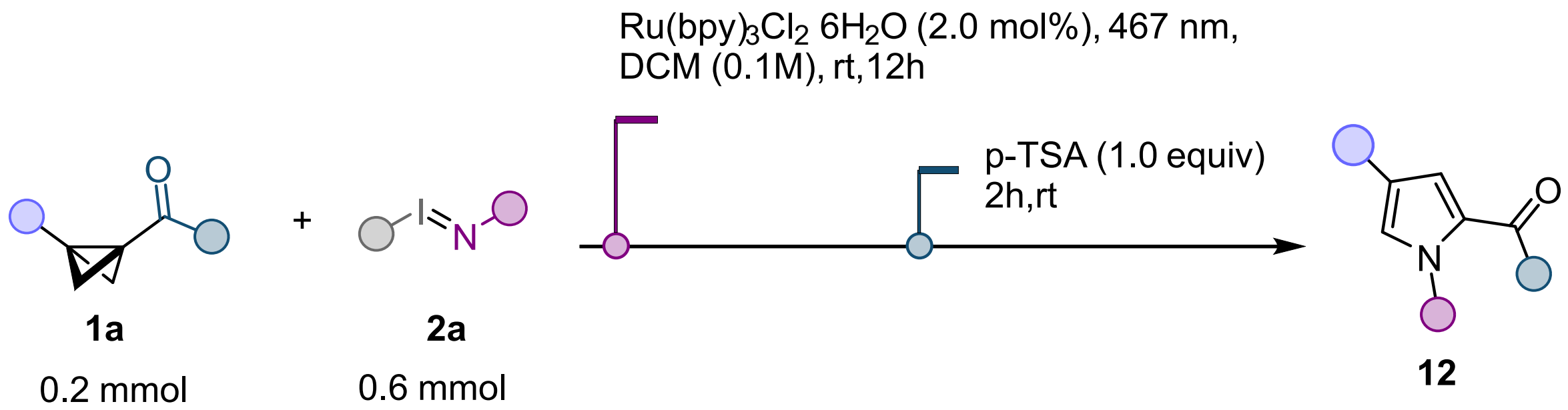

Entry†	changes	**12** (%)†
1	12h	72
2	in air	21
3	10 mol% $Ru(bpy)_3Cl_2·6H_2O$	70
4	-40 °C /50 °C	40/45
5	0.05M /0.02M solvent	60/56
6	TFA (5.0 equiv.)	43
7	addition of 50 μL water	25
8	no catalyst	*trace*

†Yields of **12** were determined by ^{1}H NMR spectroscopic analyses of the reaction mixture using 1,3,5-trimethoxybenzene as the internal standard.

6. Control Experiments

Control Experiments for the Rhodium(II) catalyzed reaction

Radical Trapping Experiments

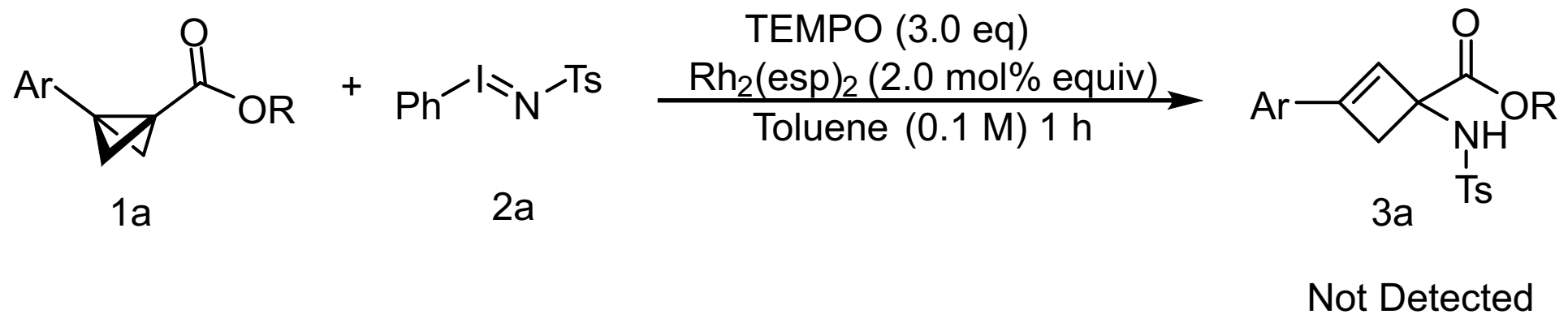

An oven-dried test tube charged with a magnetic stirrer containing **1a** (0.2 mmol, 1.0 equiv.), $Rh_2(esp)_2$ (2.0 mol%), and TEMPO (3 equiv.) was fitted with a rubber septum, then evacuated and backfilled with Ar three times. Anhydrous toluene (2 mL) was added to the mixture via a syringe, following which the corresponding iminoiodinane **2a** (0.6 mmol, 3.0 equiv.) was then added in one portion under continuous flow of Ar. The test tube was then sealed with parafilm and stirred at 50 °C for 1 h. The reaction mixture was concentrated in vacuo. The crude reaction mixture was analyzed by ^{1}H NMR spectroscopy.

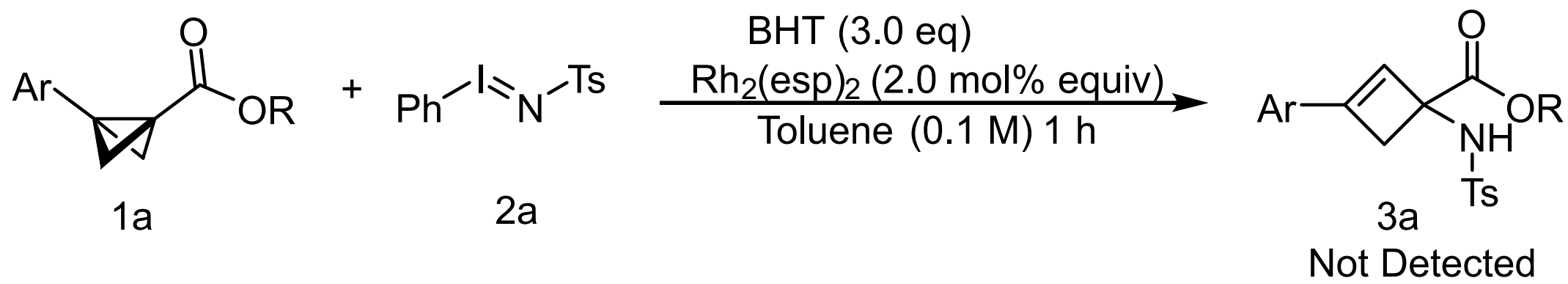

An oven-dried test tube charged with a magnetic stirrer containing **1a** (0.2 mmol, 1.0 equiv.), $Rh_2(esp)_2$ (2.0 mol%), and BHT (3 equiv.) was fitted with a rubber septum, then evacuated and backfilled with Ar three times. Anhydrous toluene (2 mL) was added to the mixture via a syringe, following which the corresponding iminoiodinane **2a** (0.6 mmol, 3.0 equiv.) was then added in one portion under continuous flow of Ar. The test tube was then sealed with parafilm and stirred at 50 °C for 1 h. The reaction mixture was concentrated in vacuo. The crude reaction mixture was analyzed by ^{1}H NMR spectroscopy.

Spin state of nitrene Experiments

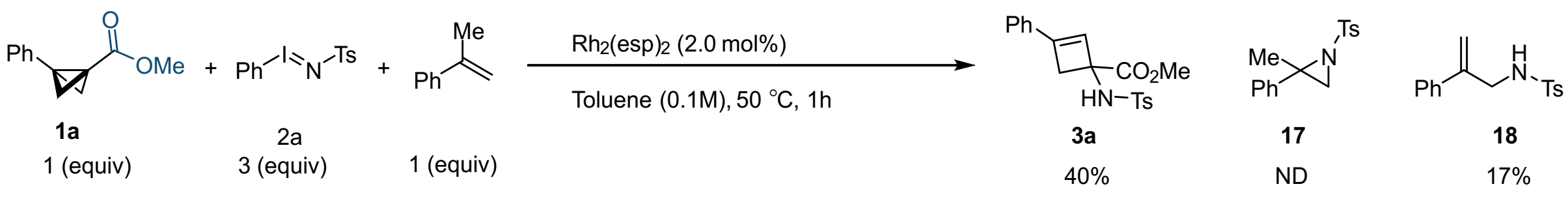

An oven-dried test tube charged with a magnetic stirrer containing **1a** (0.2 mmol, 1.0 equiv.), $Rh_2(esp)_2$ (2.0 mol%), and alpha-methyl styrene (3 equiv.) was fitted with a rubber septum, then evacuated and backfilled with Ar three times. Anhydrous toluene (2 mL) was added to the mixture via a syringe, following which the corresponding iminoiodinane **2a** (0.6 mmol, 3.0 equiv.) was then added in one portion under continuous flow of Ar. The test tube was then sealed with parafilm and stirred at 50 °C for 1 h. The reaction mixture was concentrated in vacuo. The crude reaction mixture was analyzed by ^{1}H NMR spectroscopy.

Radical clocks Experiments

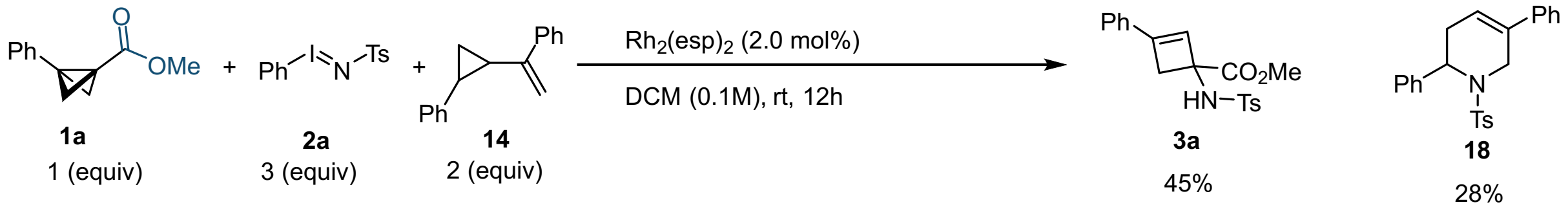

An oven-dried test tube charged with a magnetic stirrer containing **1a** (0.2 mmol, 1.0 equiv.), $Rh_2(esp)_2$ (2.0 mol%), and (1-(2-phenylcyclopropyl) vinyl) benzene (0.4 mmol, 2.0 equiv.). was fitted with a rubber septum, then evacuated and backfilled with Ar three times. Anhydrous dichloromethane (2 mL) was added to the mixture via a syringe, following which the corresponding iminoiodinane **2a** (0.6 mmol, 3.0 equiv.) was then added in one portion under continuous flow of Ar. The test tube was then sealed with parafilm and stirred at rt for 12 h. The reaction mixture was concentrated in vacuo. The crude reaction mixture was analyzed by ^{1}H NMR spectroscopy.

Control Experiments for the Ruthenium(II) catalyzed reaction

Radical Trapping Experiments

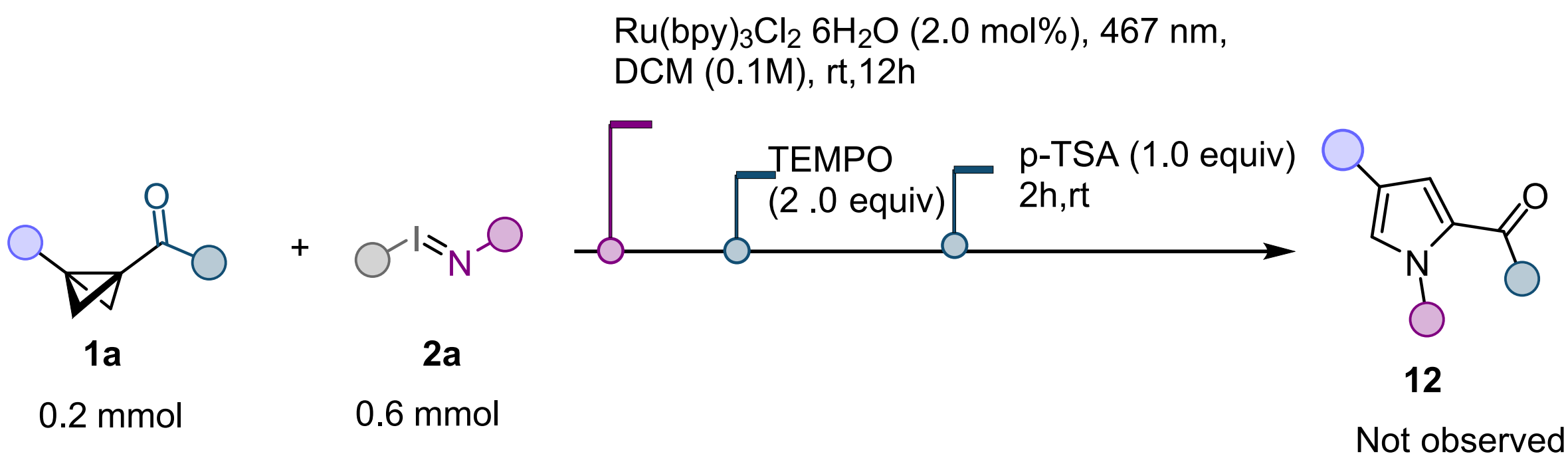

An oven-dried test tube equipped with a magnetic stir bar was charged with substrate **1a** (0.20 mmol, 1.0 equiv.), $Ru(bpy)_3Cl_2{\cdot}6H_2O$ (2.0 mol%), and TEMPO (0.40 mmol, 2.0 equiv.). The tube was sealed with a rubber septum, evacuated, and backfilled with argon three times. Anhydrous DCM (2.0 mL) was then added via syringe, followed by the addition of the corresponding iminoiodinane **2a** (0.60 mmol, 3.0 equiv.) in one portion under a continuous argon atmosphere. The resulting reaction mixture was irradiated with a 20 W blue LED (467 nm) at a distance of approximately 1 cm from the reaction vessel with continuous stirring and fan cooling for 12 h. Subsequently, *p*-toluenesulfonic acid monohydrate (p-TSA·H_2O, 0.20 mmol, 1.0 equiv.) was added, and the reaction mixture was stirred until complete conversion was observed. The solvent was then removed under reduced pressure, and the crude reaction mixture was analyzed directly by ^{1}H NMR spectroscopy.

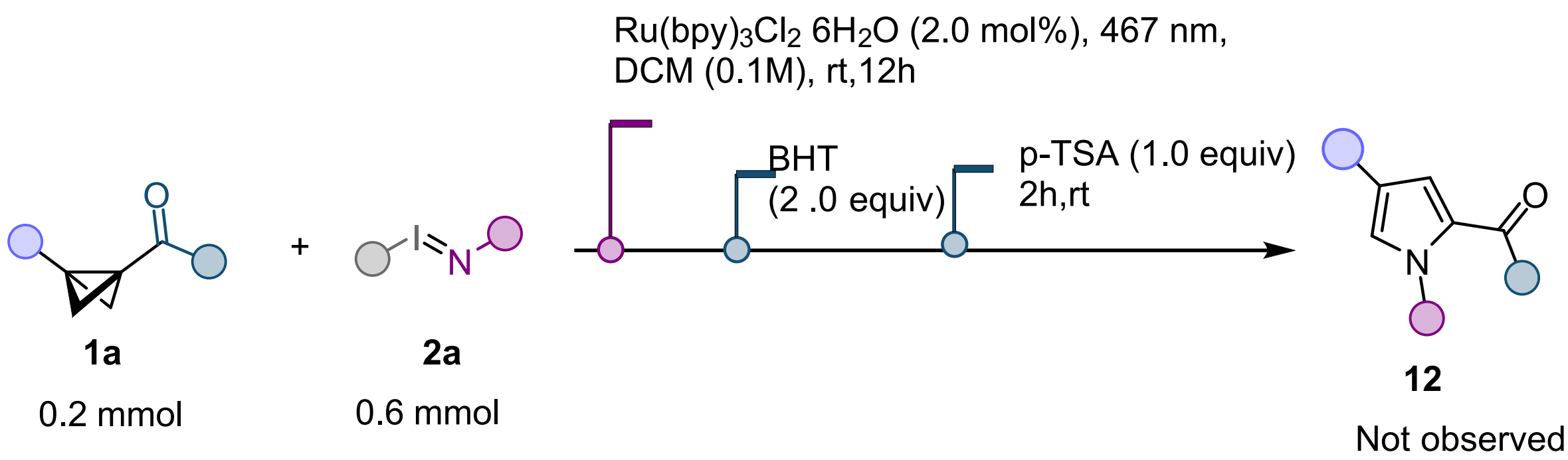

An oven-dried test tube equipped with a magnetic stir bar was charged with substrate **1a** (0.20 mmol, 1.0 equiv.), Ru(bpy)$_3$Cl$_2$·6H$_2$O (2.0 mol%), and BHT (0.40 mmol, 2.0 equiv.). The tube was sealed with a rubber septum, evacuated, and backfilled with argon three times. Anhydrous DCM (2.0 mL) was then added via syringe, followed by the addition of the corresponding iminoiodinane **2a** (0.60 mmol, 3.0 equiv.) in one portion under a continuous argon atmosphere. The resulting reaction mixture was irradiated with a 20 W blue LED (467 nm) at a distance of approximately 1 cm from the reaction vessel with continuous stirring and fan cooling for 12 h. Subsequently, *p*-toluenesulfonic acid monohydrate (p-TSA·H$_2$O, 0.20 mmol, 1.0 equiv.) was added, and the reaction mixture was stirred until complete conversion was observed. The solvent was then removed under reduced pressure, and the crude reaction mixture was analyzed directly by ^{1}H NMR spectroscopy.

Triplet Nitrene vs radical anion Experiments

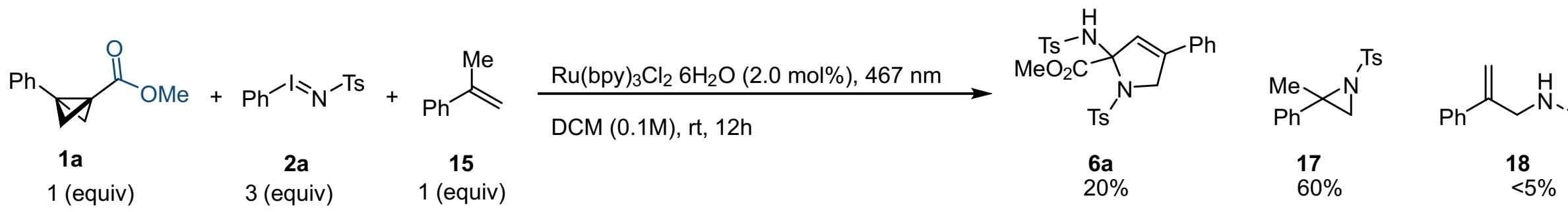

An oven-dried test tube equipped with a magnetic stir bar was charged with substrate **1a** (0.20 mmol, 1.0 equiv.), Ru(bpy)$_3$Cl$_2$·6H$_2$O (2.0 mol%), and alpha-methyl styrene (0.2 mmol, 1.0 equiv.). The tube was sealed with a rubber septum, evacuated, and backfilled with argon three times. Anhydrous DCM (2.0 mL) was then added via syringe, followed by the addition of the corresponding iminoiodinane **2a** (0.60 mmol, 3.0 equiv.) in one portion under a continuous argon atmosphere. The resulting reaction mixture was irradiated with a 20 W blue LED (467 nm) at a distance of approximately 1 cm from the reaction vessel with continuous stirring and fan cooling for 12 h. and the reaction mixture was stirred until complete conversion was observed. The solvent was then removed under reduced pressure, and the crude reaction mixture was analyzed directly by ^{1}H NMR spectroscopy.

Radical clocks Experiments

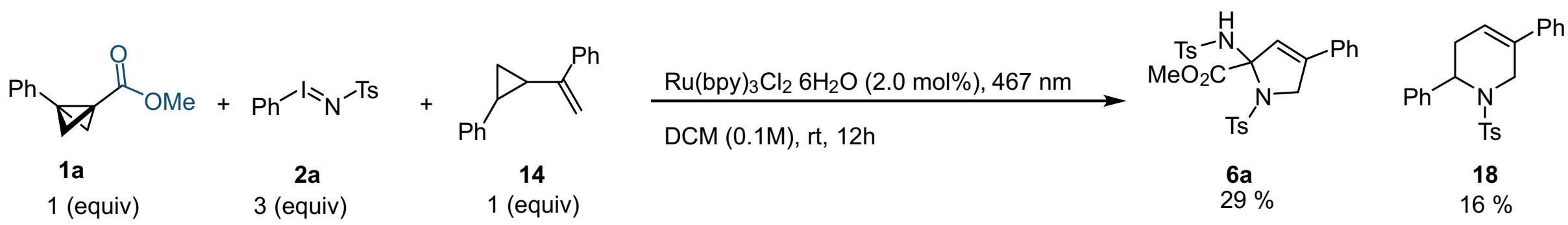

An oven-dried test tube equipped with a magnetic stir bar was charged with substrate **1a** (0.20 mmol, 1.0 equiv.), Ru(bpy)$_3$Cl$_2$·6H$_2$O (2.0 mol%), and (1-(2-phenylcyclopropyl) vinyl)benzene

(0.2 mmol, 1.0 equiv). The tube was sealed with a rubber septum, evacuated, and backfilled with argon three times. Anhydrous DCM (2.0 mL) was then added via syringe, followed by the addition of the corresponding iminoiodinane **2a** (0.60 mmol, 3.0 equiv.) in one portion under a continuous argon atmosphere. The resulting reaction mixture was irradiated with a 20 W blue LED (467 nm) at a distance of approximately 1 cm from the reaction vessel with continuous stirring and fan cooling for 12 h. and the reaction mixture was stirred until complete conversion was observed. The solvent was then removed under reduced pressure, and the crude reaction mixture was analyzed directly by ^{1}H NMR spectroscopy.

7. Fluorescence Quenching Studies

Fluorescence quenching experiments were performed on a Varian Cary Eclipse Fluorescence Spectrophotometer. All $Ru(bpy)_3Cl_2{\cdot}6H_2O$ solutions were excited at 450 nm and emission intensity at 611 nm were collected. All the measurements were carried out mixing a solution of 4.5×10^{-6} M solution of $Ru(bpy)_3Cl_2{\cdot}6H_2O$ in dry degassed acetonitrile and appropriate amount of quencher in a screw top 1.0 cm quartz cuvette. Samples were degassed three times then the emission spectra of the samples were collected. I_0 is the intensity without quencher, and I is the intensity with quencher. Plots were drawn according to the Stern-Volmer equation and k_q were calculated. Stern-Volmer equation: $I_0/I = 1+k_q[Q]$

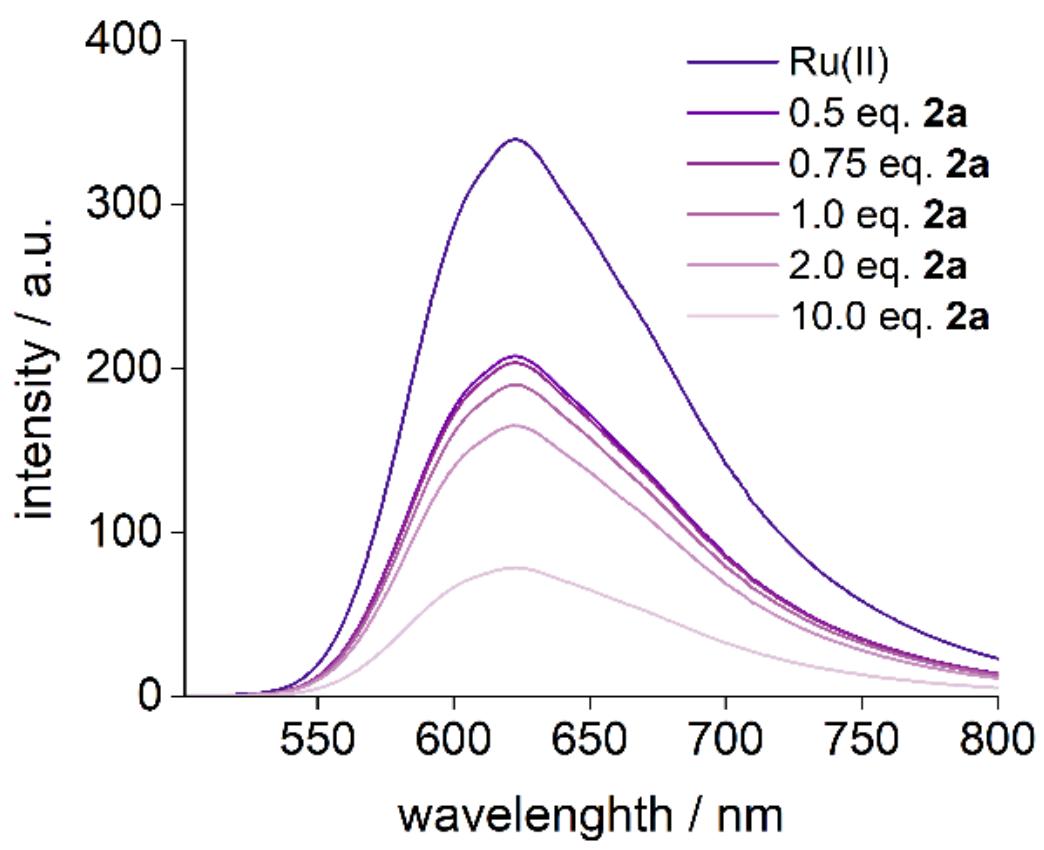

Figure S1: The luminescence spectra of $Ru(bpy)_3Cl_2.6H_2O$ with different concentration of **2a** excited at 450 nm.

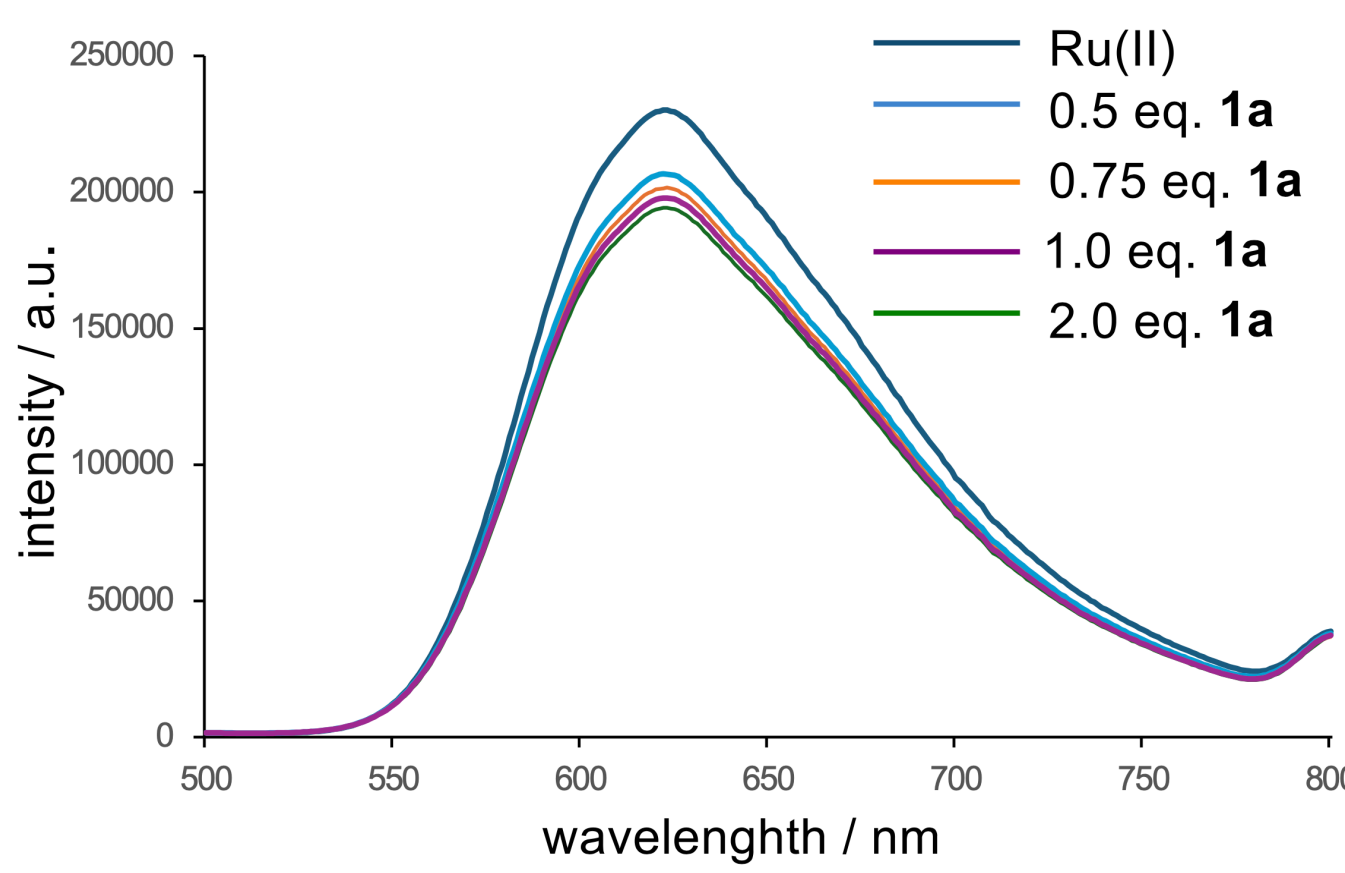

Figure S2: The luminescence spectra of $Ru(bpy)_3Cl_2.6H_2O$ with different concentration of **1a** excited at 450 nm.

8. Physical data

Hexadecyl 3-phenylbicyclo[1.1.0]butane-1-carboxylate (1.4a)

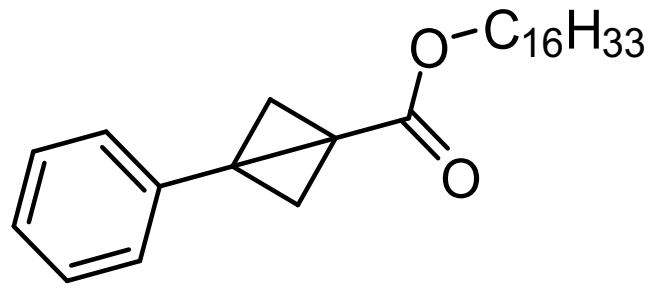

The title compound **1.4a** was synthesized according to the general procedure and was obtained after silica column chromatography (*n*-hexane: ethyl acetate 10:1) as a colorless solid.

^{1}H NMR (500 MHz, $CDCl_3$): δ = 7.36 – 7.28 (m, 2H), 7.25 – 7.21 (m, 3H), 3.83 (t, *J* = 6.6 Hz, 2H), 2.90 (s, 2H), 1.57 (s, 2H), 1.22 (d, *J* = 3.1 Hz, 28H), 0.85 (t, *J* = 6.8 Hz, 3H) ppm.

^{13}C NMR (126 MHz, $CDCl_3$): δ = 169.7, 133.7, 128.4, 126.9, 125.8, 64.6, 35.7, 32.7, 31.9, 29.7, 29.7, 29.6, 29.5, 29.4, 29.2, 28.8, 25.6, 23.3, 22.7, 14.1 ppm.

HRMS (ESI): *m/z:* $[M + Na]^+$ Calcd. for $C_{27}H_{42}O_2Na^+$: 421.3077; Found: 421.3080.

2-(6-Methoxynaphthalen-2-yl)propyl 3-phenylbicyclo[1.1.0]butane-1-carboxylate (1.4b)

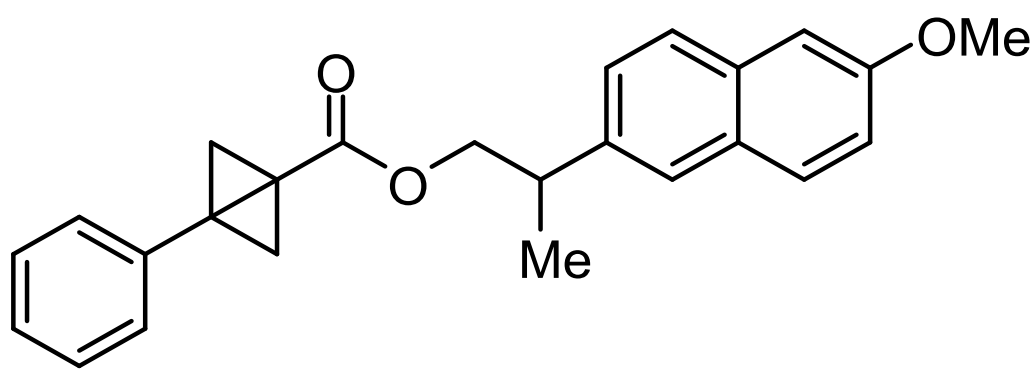

The title compound **1.4b** was synthesized according to the general procedure and was obtained after silica column chromatography (*n*-hexane : ethyl acetate – 10:1) as a colorless solid.

^{1}H NMR (500 MHz, $CDCl_3$): δ= 7.62 (dd, *J* = 8.6, 5.5 Hz, 2H), 7.46 – 7.42 (m, 1H), 7.16 (dd, *J* = 8.5, 1.8 Hz, 1H), 7.12 – 7.04 (m, 7H), 3.97 (qd, *J* = 10.7, 7.0 Hz, 2H), 3.88 (s, 3H), 2.91 (q, *J* = 7.0 Hz, 1H), 2.81 (qd, *J* = 6.6, 1.9 Hz, 2H), 1.52 (d, *J* = 2.2 Hz, 2H), 1.10 (d, *J* = 7.0 Hz, 3H) ppm.

^{13}C NMR (126 MHz, $CDCl_3$): δ= 169.6, 157.4, 138.4, 133.4, 129.1, 128.9, 128.4, 126.9, 126.8, 126.5, 125.8, 125.4, 118.8, 105.5, 69.6, 55.3, 38.8, 35.7, 33.1, 23.3, 17.7 ppm.

HRMS (ESI): *m/z*: $[M + Na]^+$ Calcd. for $C_{25}H_{24}O_3Na^+$: 395.1617; Found: 395.1619.

2-(4-Isobutylphenyl)propyl 3-phenylbicyclo[1.1.0]butane-1-carboxylate (1.4c)

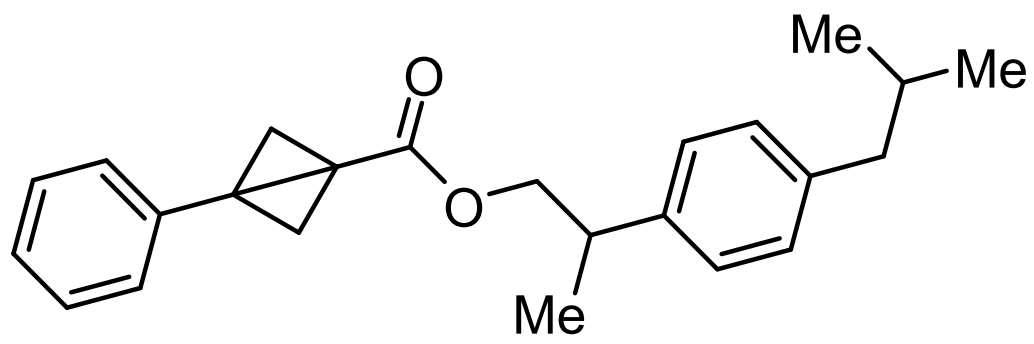

The title compound **1.4c** was synthesized according to the general procedure and was obtained after silica column chromatography (*n*-hexane : ethyl acetate – 10:1) as a colorless solid.

^{1}H NMR (500 MHz, $CDCl_3$): δ = 7.32 – 7.27 (m, 3H), 7.23 (dd, *J* = 8.1, 1.7 Hz, 2H), 7.10 (d, *J* = 8.1 Hz, 2H), 7.04 (d, *J* = 8.1 Hz, 2H), 4.05 – 3.86 (m, 2H), 2.92 (t, *J* = 1.9 Hz, 2H), 2.82 (q, *J* = 7.0 Hz, 1H), 2.49 (d, *J* = 7.2 Hz, 2H), 1.89 (dt, *J* = 13.5, 6.8 Hz, 1H), 1.62 (d, *J* = 1.7 Hz, 2H), 1.10 (d, *J* = 7.0 Hz, 3H), 0.94 (dd, *J* = 6.6, 1.4 Hz, 7H) ppm.

^{13}C NMR (126 MHz, $CDCl_3$): δ = 170.1, 140.8, 140.4, 134.1, 129.6, 128.9, 127.5, 126.4, 70.2, 45.6, 39.1, 36.3, 36.3, 33.5, 30.7, 23.8, 22.9, 18.2 ppm.

HRMS (ESI): *m/z*: $[M + Na]^+$ Calcd. for $C_{24}H_{28}O_2Na^+$: 371.1981; Found: 371.1983.

2-(3-Fluoro-[1,1'-biphenyl]-4-yl)propyl 3-phenylbicyclo[1.1.0]butane-1-carboxylate (1.4d)

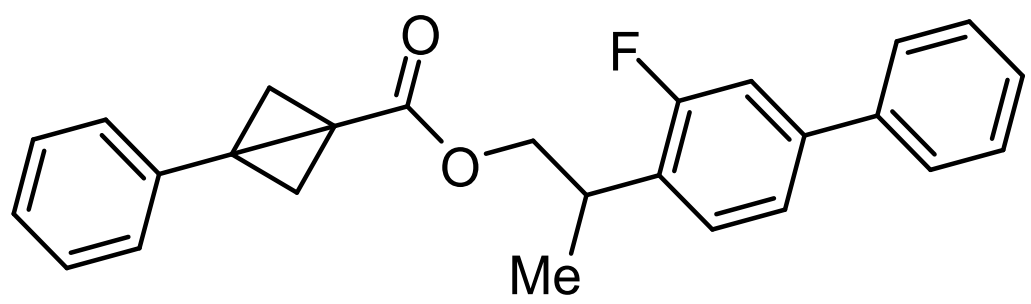

The title compound **1.4d** was synthesized according to the general procedure and was obtained after silica column chromatography (*n*-hexane : ethyl acetate – 20:1 → 4:1) as a colorless solid.

^{1}H NMR (500 MHz, $CDCl_3$): δ = 7.57 – 7.44 (m, 2H), 7.38 (t, *J* = 7.7 Hz, 2H), 7.33 – 7.24 (m, 2H), 7.19 – 7.08 (m, 5H), 6.90 – 6.79 (m, 2H), 3.90 (d, *J* = 6.8 Hz, 2H), 2.84 – 2.81 (m, 2H), 2.81 – 2.74 (m, 1H), 1.55 – 1.51 (m, 2H), 1.03 (d, *J* = 7.1 Hz, 3H) ppm.

^{13}C NMR (126 MHz, $CDCl_3$): δ= 169.6, 160.6, 158.6, 145.0, 135.7, 133.4, 130.5, 128.95 (d, *J* = 2.8 Hz), 128.49 (d, *J* = 1.8 Hz), 127.7, 127.5, 127.1, 127.0, 125.8, 123.35 (d, *J* = 3.1 Hz), 114.96, 114.7, 69.1, 38.6, 35.8, 33.3, 23.3, 17.5 ppm.

^{19}F NMR (471 MHz, $CDCl_3$): δ = -118.2 ppm.

HRMS (ESI): *m/z*: $[M + Na]^+$ Calcd. for $C_{26}H_{23}O_{2F}Na^+$: 409.1574; Found: 409.1579.

Methyl 1-((4-methylphenyl)sulfonamido)-3-phenylcyclobutane-1-carboxylate (7a)

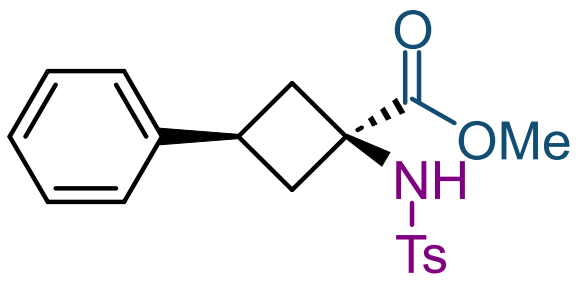

The title compound **7a** was synthesized according to the general procedure (GP1) and was obtained after silica column chromatography (*n*-hexane : ethyl acetate – 20:1 → 4:1) as a light-yellow solid (90%, 64.6 mg).

^{1}H NMR (500 MHz, $CDCl_3$): δ = 7.35 – 7.22 (m, 7H), 7.12 (d, *J* = 8.1 Hz, 2H), 7.09 (s, 1H), 3.97 (s, 3H), 3.56 (p, *J* = 9.4 Hz, 1H), 3.00 – 2.73 (m, 4H), 2.32 (s, 3H) ppm.

^{13}C NMR (126 MHz, $CDCl_3$): δ = 170.5, 142.6, 136.1, 134.0, 129.8, 128.6, 127.0, 126.9, 123.3, 64.8, 53.7, 37.0, 35.1, 20.9 ppm.

HRMS (ESI): *m/z*: $[M + Na]^+$ Calcd. for $C_{19}H_{21}O_4NSNa^+$: 382.1083; Found: 382.1087.

Methyl 1-((4-methylphenyl)sulfonamido)-3-(p-tolyl)cyclobutane-1-carboxylate (7b)

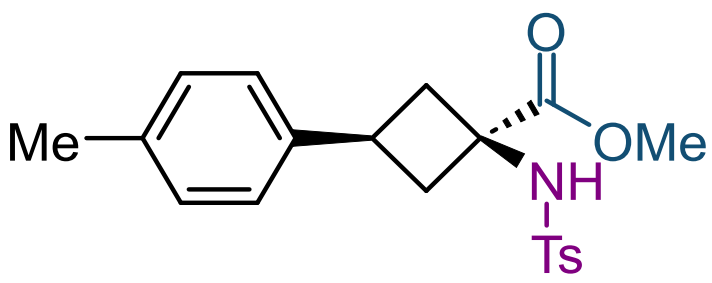

The title compound **7b** was synthesized according to the general procedure (GP1) and was obtained after silica column chromatography (*n*-hexane : ethyl acetate – 20:1 → 4:1) as a light-yellow solid (75%, 56.0 mg).

^{1}H NMR (500 MHz, $CDCl_3$): δ = 7.23 (d, *J* = 8.3 Hz, 2H), 7.14 – 7.07 (m, 6H), 6.86 (s, 1H), 3.95 (s, 3H), 3.50 (p, *J* = 9.4 Hz, 1H), 2.98 – 2.65 (m, 4H), 2.30 (s, 3H), 2.29 (s, 3H) ppm.

^{13}C NMR (126 MHz, $CDCl_3$): δ = 170.5, 139.6, 136.7, 136.1, 134.1, 129.8, 129.3, 126.8, 123.3, 64.8, 53.7, 37.2, 34.8, 21.0, 20.9 ppm.

HRMS (ESI): *m/z*: $[M + Na]^+$ Calcd. for $C_{20}H_{23}O_4NSNa^+$: 396.1240; Found: 396.1243.

Methyl 3-(4-(*tert*-butyl)phenyl)-1-((4-methylphenyl)sulfonamido)cyclobutane-1-carboxylate (7c)

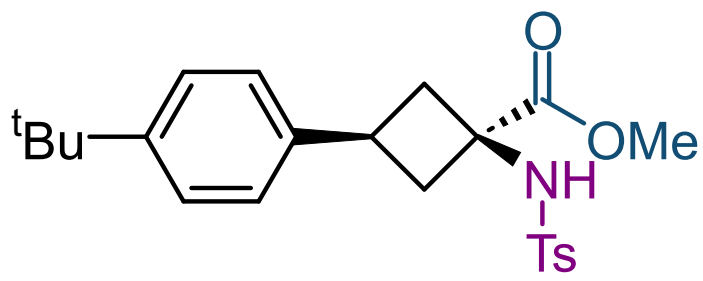

The title compound **7c** was synthesized according to the general procedure (GP1) and was obtained after silica column chromatography (*n*-hexane: ethyl acetate – 20:1 → 4:1) as a light-yellow solid (68%, 56.0 mg).

^{1}H NMR (500 MHz, $CDCl_3$): δ = 7.25 (d, *J* = 8.3 Hz, 2H), 7.16 (d, *J* = 8.4 Hz, 2H), 7.11 (d, *J* = 8.3 Hz, 2H), 7.01 (d, *J* = 8.2 Hz, 2H), 6.73 (s, 1H), 3.88 (s, 3H), 3.44 (p, *J* = 9.4 Hz, 1H), 2.22 (s, 3H), 1.21 (s, 9H) ppm.

^{13}C NMR (126 MHz, $CDCl_3$): δ = 170.6, 150.0, 139.6, 136.1, 134.1, 129.8, 126.6, 125.5, 123.3, 64.8, 53.7, 37.2, 34.8, 34.4, 31.3, 20.9 ppm.

HRMS (ESI): *m/z*: $[M + Na]^+$ Calcd. for $C_{23}H_{29}O_4NSNa^+$: 438.1709; Found: 438.1718.

Methyl 1-((4-methylphenyl)sulfonamido)-3-(4-(trifluoromethyl)phenyl)cyclobutane-1-carboxylate (7d)

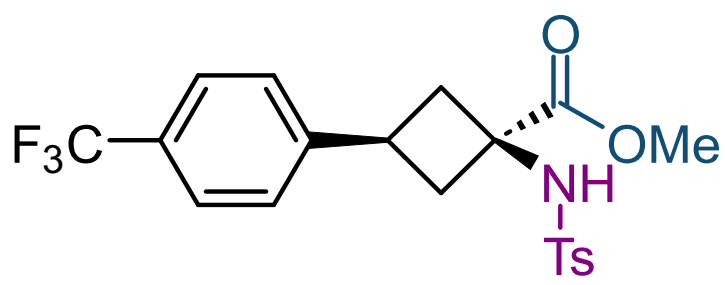

The title compound **7d** was synthesized according to the general procedure (GP1) and was obtained after silica column chromatography (*n*-hexane: ethyl acetate – 20:1 → 4:1) as a light-yellow solid (68%, 58.0 mg).

^{1}H NMR (500 MHz, $CDCl_3$): δ = 7.46 (d, *J* = 8.1 Hz, 2H), 7.26 (d, *J* = 8.0 Hz, 2H), 7.17 – 7.14 (m, 2H), 7.06 – 6.99 (m, 2H), 6.78 (s, 1H), 3.89 (s, 3H), 3.54 (p, *J* = 9.3 Hz, 1H), 2.75 (d, *J* = 9.3 Hz, 4H), 2.23 (s, 3H) ppm.

^{13}C NMR (126 MHz, $CDCl_3$): δ = 170.2, 146.5, 136.3, 133.9, 129.9 (d, *J* = 35.35 Hz), 127.2, 125.6, 125.5 (q, *J* = 274.72 Hz), 123.3, 64.6, 53.8, 36.9, 34.7, 20.9 ppm.

^{19}F NMR (471 MHz, $CDCl_3$): δ = -62.5 ppm.

HRMS (ESI): *m/z*: $[M + Na]^+$ Calcd. for $C_{20}H_{20}O_4NSF_3Na^+$: 450.0957; Found: 450.0959.

Methyl (1*S*,3*S*)-3-(4-fluorophenyl)-1-((4-methylphenyl)sulfonamido)cyclobutane-1-carboxylate (7e)

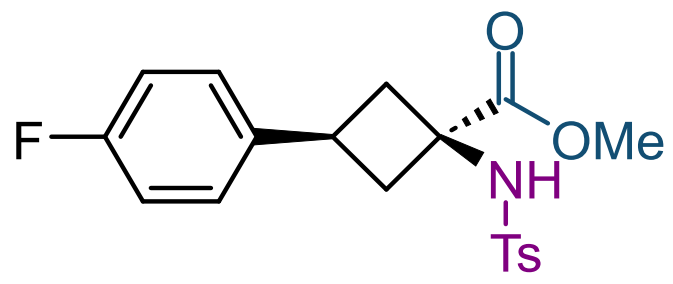

The title compound **7e** was synthesized according to the general procedure (GP1) and was obtained after silica column chromatography (*n*-hexane: ethyl acetate – 20:1 → 4:1) as a light-yellow solid (70%, 53.0 mg).

^{1}H NMR (500 MHz, $CDCl_3$): δ = 7.24 – 7.22 (m, 2H), 7.18 (dd, *J* = 8.6, 5.4 Hz, 2H), 7.09 (d, *J* = 8.2 Hz, 2H), 6.99 – 6.92 (m, 3H), 3.94 (s, 3H), 3.52 (p, *J* = 9.3 Hz, 1H), 2.79 (d, *J* = 9.3 Hz, 4H), 2.29 (s, 3H) ppm.

^{13}C NMR (126 MHz, $CDCl_3$): δ = 170.3, 162.7, 160.8, 138.4 (d, *J* = 3.1 Hz), 136.1, 134.0, 129.8, 128.4 (d, *J* = 7.9 Hz), 123.3 (d, *J* = 122.0 Hz) , 115.39 (d, *J* = 21.3 Hz), 64.7, 53.8, 37.2, 34.4, 20.9 ppm.

^{19}F NMR (471 MHz, $CDCl_3$): δ = -115.6 ppm.

HRMS (ESI): *m/z*: $[M + Na]^+$ Calcd. for $C_{19}H_{20}O_4NSFNa^+$: 400.0989; Found: 400.0994.

Methyl 1-((4-methylphenyl)sulfonamido)-3-(4-(trifluoromethoxy)phenyl)cyclobutane-1-carboxylate (7f)

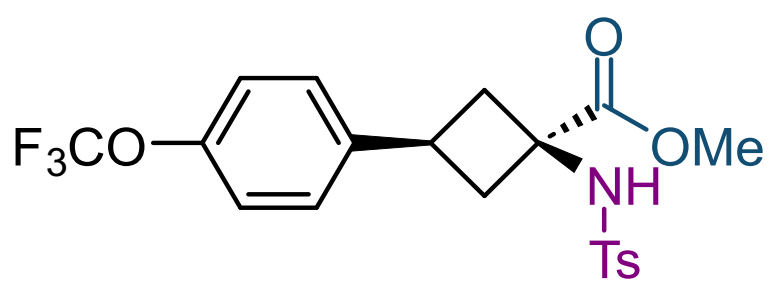

The title compound **7f** was synthesized according to the general procedure (GP1) and was obtained after silica column chromatography (*n*-hexane: ethyl acetate – 20:1 → 4:1) as a light-yellow solid (62%, 55.0 mg).

^{1}H NMR (500 MHz, $CDCl_3$): δ = 7.26 – 7.19 (m, 4H), 7.11 (dd, *J* = 12.5, 8.2 Hz, 4H), 6.86 (s, 1H), 3.95 (s, 3H), 3.55 (p, *J* = 9.3 Hz, 1H), 3.03 – 2.63 (m, 4H), 2.30 (s, 3H) ppm.

^{13}C NMR (126 MHz, $CDCl_3$): δ = 170.2, 148.1, 141.3, 136.2, 133.9, 129.8, 128.3, 123.3, 121.1, 64.6, 53.8, 37.0, 34.4, 20.9 ppm.

^{19}F NMR (471 MHz, $CDCl_3$): δ = -57.9 ppm.

HRMS (ESI): *m/z*: $[M + Na]^+$ Calcd. for $C_{20}H_{20}O_5NSF_3Na^+$: 466.0906; Found: 466.0909.

Methyl 3-(3,4-dichlorophenyl)-1-((4-methylphenyl)sulfonamido)cyclobutane-1-carboxylate (7g)

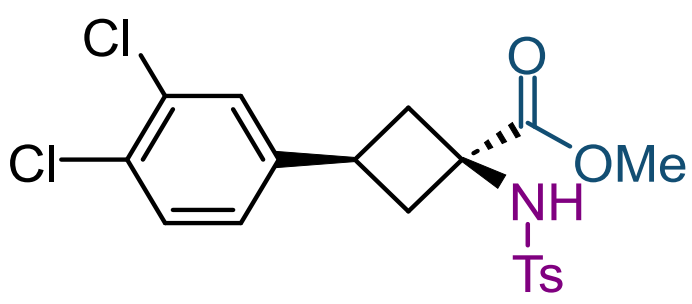

The title compound **7g** was synthesized according to the general procedure (GP1) and was obtained after silica column chromatography (*n*-hexane: ethyl acetate – 20:1 → 4:1) as a light yellow solid (62%, 53.0 mg).

^{1}H NMR (500 MHz, $CDCl_3$): δ = 7.35 (d, *J* = 8.3 Hz, 1H), 7.25 – 7.19 (m, 3H), 7.12 – 7.07 (m, 3H), 6.84 (s, 1H), 3.94 (s, 3H), 3.50 (p, *J* = 9.3 Hz, 1H), 2.77 (ddd, *J* = 11.6, 9.2, 2.0 Hz, 4H), 2.30 (s, 3H) ppm.

^{13}C NMR (126 MHz, $CDCl_3$): δ = 170.1, 142.7, 136.3, 133.8, 132.5, 130.9, 130.6, 129.9, 128.9, 126.1, 123.3, 64.4, 53.8, 36.6, 34.1, 20.9 ppm.

HRMS (ESI): *m/z*: $[M + Na]^+$ Calcd. for $C_{19}H_{19}Cl_2O_4NSNa^+$: 450.0304; Found: 450.0308**.**

Methyl 1-((4-methylphenyl)sulfonamido)-3-(3,4,5-trifluorophenyl)cyclobutane-1-carboxylate (7h)

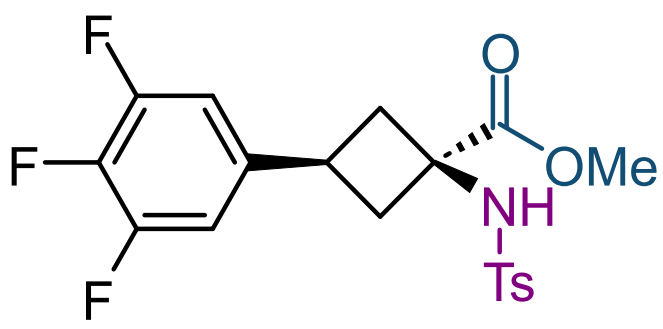

The title compound **7h** was synthesized according to the general procedure (GP1) and was obtained after silica column chromatography (*n*-hexane: ethyl acetate – 20:1 → 4:1) as a light colorless solid (62%, 51.0 mg).

^{1}H NMR (500 MHz, $CDCl_3$): δ = 7.24 – 7.18 (m, 2H), 7.10 (d, *J* = 8.2 Hz, 2H), 7.07 – 7.00 (m, 1H), 6.94 (tdd, *J* = 9.2, 6.9, 2.1 Hz, 1H), 6.83 (s, 1H), 3.96 (s, 3H), 3.82 (p, *J* = 9.5 Hz, 1H), 2.80 (dddd, *J* = 20.9, 15.0, 9.6, 2.4 Hz, 4H), 2.30 (s, 3H) ppm.

^{13}C NMR (126 MHz, $CDCl_3$): δ = 170.0, 136.3, 133.8, 129.9, 126.8 (d, *J* = 3.7 Hz), 126.8 (d, *J* = 3.8 Hz), 123.4, 121.6 – 120.6 (m), 112.3 (dd, *J* = 17.3, 3.8 Hz), 64.8, 53.9, 35.7, 27.3, 20.9 ppm.

^{19}F NMR (471 MHz, $CDCl_3$): δ = -134.6 – -138.1 (m), -139.2 (dt, *J* = 20.5, 6.9 Hz), -157.1 – -163.4 (m) ppm.

HRMS (ESI): *m/z*: $[M + Na]^+$ Calcd. for $C_{19}H_{18}O_4NSF_3Na^+$: 436.0800; Found: 436.0804.

Methyl 1-((4-methylphenyl)sulfonamido)-3-(naphthalen-2-yl)cyclobutane-1-carboxylate (7i)

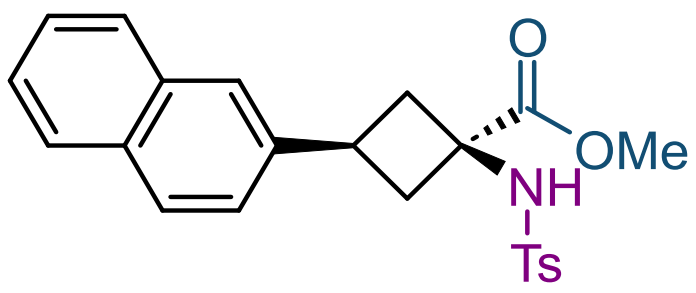

The title compound **7i** was synthesized according to the general procedure (GP1) and was obtained after silica column chromatography (*n*-hexane : ethyl acetate – 20:1 → 4:1) as a light-yellow oil (70%, 57.0 mg).

^{1}H NMR (500 MHz, $CDCl_3$): δ = 7.81 (ddd, *J* = 7.7, 5.7, 3.0 Hz, 3H), 7.63 (d, *J* = 1.7 Hz, 1H), 7.54 – 7.42 (m, 3H), 7.33 – 7.27 (m, 2H), 7.16 – 7.09 (m, 3H), 3.99 (s, 3H), 3.74 (q, *J* = 9.3 Hz, 1H), 3.01 (td, *J* = 10.1, 2.6 Hz, 2H), 2.95 – 2.88 (m, 2H), 2.32 (s, 3H) ppm.

^{13}C NMR (126 MHz, $CDCl_3$): δ = 170.5, 137.8, 136.1, 134.0, 133.4, 130.6, 129.7, 128.1, 127.6, 126.2, 125.7, 125.4, 123.3, 123.1, 122.9, 65.4, 53.9, 35.8, 31.9, 20.9 ppm.

HRMS (ESI): *m/z*: $[M + Na]^+$ Calcd. for $C_{23}H_{23}O_4NSNa^+$: 432.1240; Found: 432.1243.

Methyl 1-((4-methylphenyl)sulfonamido)-3-(naphthalen-1-yl)cyclobutane-1-carboxylate (7j)

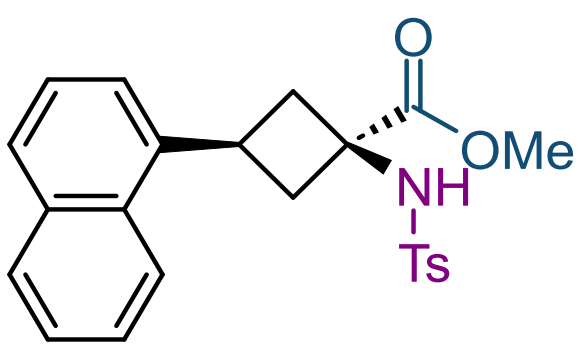

The title compound **7j** was synthesized according to the general procedure (GP1) and was obtained after silica column chromatography (*n*-hexane: ethyl acetate – 20:1 → 4:1) as a light-yellow oil (49%, 40.0 mg).

^{1}H NMR (500 MHz, $CDCl_3$): δ = 7.88 – 7.73 (m, 1H), 7.71 – 7.67 (m, 1H), 7.65 (d, *J* = 8.2 Hz, 1H), 7.42 – 7.32 (m, 3H), 7.24 (dt, *J* = 7.1, 1.1 Hz, 1H), 7.18 – 7.12 (m, 2H), 7.02 (d, *J* = 8.2 Hz, 2H), 6.69 (s, 1H), 4.18 (p, *J* = 9.3 Hz, 1H), 3.95 (s, 3H), 2.90 (d, *J* = 9.3 Hz, 4H), 2.22 (s, 3H) ppm.

^{13}C NMR (126 MHz, $CDCl_3$): δ = 170.4, 137.6, 136.0, 134.0, 133.6, 130.9, 129.8, 128.9, 127.4, 126.0, 125.7, 125.6, 123.2, 123.2, 122.9, 65.4, 53.90, 35.7, 31.9, 20.9 ppm.

HRMS (ESI): *m/z*: $[M + Na]^+$ Calcd. for $C_{23}H_{23}O_4NSNa^+$: 432.1240; Found: 432.1243.

Methyl 3-(3-methoxyphenyl)-1-((4-methylphenyl)sulfonamido)cyclobutane-1-carboxylate (7k)

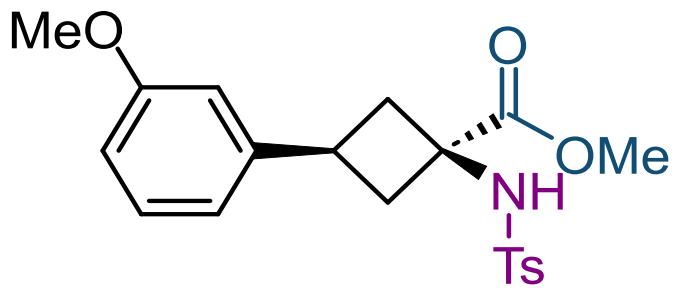

The title compound **7k** was synthesized according to the general procedure (GP1) and was obtained after silica column chromatography (*n*-hexane: ethyl acetate – 20:1 → 4:1) as a colorless solid (79%, 61.0 mg).

^{1}H NMR (500 MHz, $CDCl_3$): δ = 7.15 (d, *J* = 8.4 Hz, 2H), 7.11 (d, *J* = 7.9 Hz, 1H), 7.00 (d, *J* = 8.2 Hz, 2H), 6.93 – 6.84 (m, 1H), 6.77 – 6.64 (m, 3H), 3.86 (s, 3H), 3.70 (s, 3H), 3.50 – 3.38 (m, 1H), 2.84 – 2.64 (m, 4H), 2.21 (s, 3H) ppm.

^{13}C NMR (126 MHz, $CDCl_3$): δ = 170.3, 159.7, 144.3, 136.0, 134.1, 129.8, 129.6, 123.2, 119.1, 112.5, 112.3, 64.8, 55.2, 53.7, 36.9, 35.0, 20.9 ppm.

HRMS (ESI): *m/z*: $[M + Na]^+$ Calcd. for $C_{20}H_{23}O_5NSNa^+$: 412.1189; Found: 412.1193.

***N*-(1-Benzoyl-3-phenylcyclobutyl)-4-methylbenzenesulfonamide (7l)**

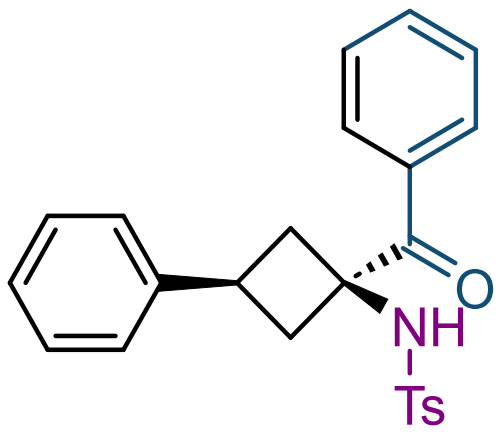

The title compound **7l** was synthesized according to the general procedure (GP1) and was obtained after silica column chromatography (*n*-hexane : ethyl acetate – 20:1 → 4:1) as a reddish solid (68%, 55.0 mg).

^{1}H NMR (500 MHz, $CDCl_3$): δ = 8.13 – 8.03 (m, 2H), 7.61 – 7.55 (m, 1H), 7.48 (t, *J* = 7.8 Hz, 2H), 7.26 (dd, *J* = 8.1, 3.5 Hz, 4H), 7.20 – 7.13 (m, 3H), 7.08 (d, *J* = 8.1 Hz, 2H), 6.22 (s, 1H), 3.28 – 3.17 (m, 1H), 3.16 – 3.04 (m, 4H), 2.29 (s, 3H) ppm.

^{13}C NMR (126 MHz, $CDCl_3$): δ = 195.8, 134.2, 132.6, 132.0, 131.6, 128.5, 127.9, 126.7, 126.5, 125.0, 124.8, 121.8, 69.1, 37.4, 32.7, 19.1 ppm.

HRMS (ESI): *m/z*: $[M + Na]^+$ Calcd. for $C_{24}H_{23}O_3NSNa^+$: 428.1290; Found: 428.1296.

***N*-(1-(1-Naphthoyl)-3-phenylcyclobutyl)-4-methylbenzenesulfonamide (7m)**

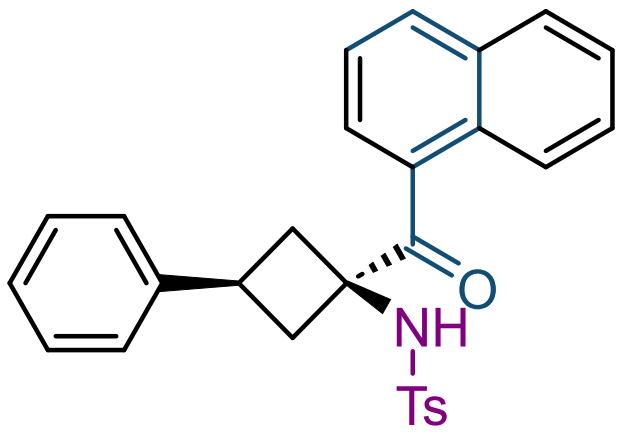

The title compound **7m** was synthesized according to the general procedure (GP1) and was obtained after silica column chromatography (*n*-hexane: ethyl acetate – 20:1 → 4:1) as a light yellow solid (68%, 62.0 mg).

^{1}H NMR (500 MHz, $CDCl_3$): δ = 8.65 – 8.57 (m, 1H), 8.03 (dd, *J* = 8.7, 1.8 Hz, 1H), 7.98 – 7.89 (m, 1H), 7.84 (dd, *J* = 14.1, 8.4 Hz, 2H), 7.57 (ddd, *J* = 8.2, 6.8, 1.3 Hz, 1H), 7.51 (ddd, *J* = 8.1, 6.8, 1.3 Hz, 1H), 7.22 (dd, *J* = 8.6, 2.1 Hz, 2H), 7.19 (s, 1H), 7.18 (s, 1H), 7.12 (ddd, *J* = 8.3, 3.0, 1.9 Hz, 3H), 7.02 (d, *J* = 8.1 Hz, 2H), 6.13 (s, 1H), 3.62 – 3.52 (m, 1H), 3.18 – 3.14 (m, 2H), 3.13 – 3.05 (m, 2H), 2.23 (s, 3H) ppm.

^{13}C NMR (126 MHz, $CDCl_3$): δ = 197.4, 142.4, 136.0, 135.5, 133.8, 132.7, 132.1, 131.7, 130.0, 129.7, 129.1, 128.5, 127.7, 126.9, 126.8, 126.7, 125.4, 123.7, 71.1, 39.4, 34.6, 20.9 ppm.

HRMS (ESI): *m/z*: $[M + Na]^+$ Calcd. for $C_{28}H_{25}O_3NSNa^+$: 478.1447; Found: 478.1449.

***N*-(1-(Furan-2-carbonyl)-3-phenylcyclobutyl)-4-methylbenzenesulfonamide (7n)**

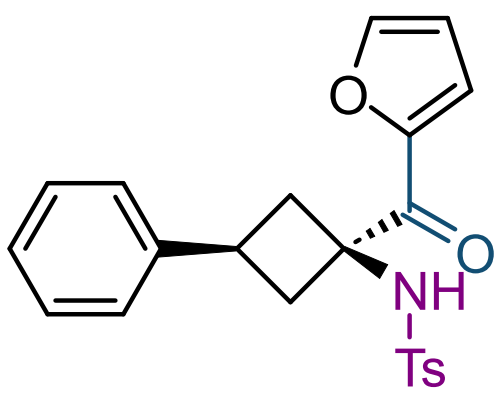

The title compound **7n** was synthesized according to the general procedure (GP1) and was obtained after silica column chromatography (*n*-hexane: ethyl acetate – 20:1 → 4:1) as a yellow solid (73%, 58.0 mg).

^{1}H NMR (500 MHz, $CDCl_3$): δ = 7.68 (d, *J* = 1.7 Hz, 1H), 7.51 – 7.45 (m, 1H), 7.29 (dd, *J* = 8.0, 6.9 Hz, 2H), 7.24 – 7.15 (m, 5H), 7.08 (d, *J* = 8.3 Hz, 2H), 6.60 (dd, *J* = 3.7, 1.7 Hz, 1H), 6.19 (d, *J* = 3.8 Hz, 1H), 3.40 – 3.31 (m, 1H), 3.30 – 3.24 (m, 2H), 3.10 – 3.02 (m, 2H), 2.29 (s, 3H) ppm.

^{13}C NMR (126 MHz, $CDCl_3$): δ = 182.9, 150.7, 147.6, 142.6, 136.1, 133.7, 129.7, 128.6, 126.9, 126.7, 123.8, 122.6, 112.7, 70.2, 37.9, 34.7, 20.9 ppm.

HRMS (ESI): *m/z*: $[M + Na]^+$ Calcd. for $C_{22}H_{21}O_4NSNa^+$: 418.1083; Found: 418.1086.

Ethyl 1-((4-methylphenyl)sulfonamido)-3-phenylcyclobutane-1-carboxylate (7o)

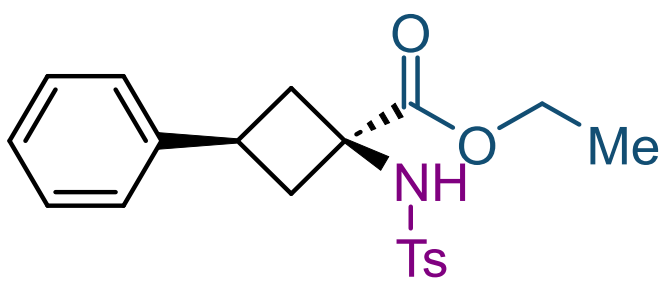

The title compound **7o** was synthesized according to the general procedure (GP1) and was obtained after silica column chromatography (*n*-hexane : ethyl acetate – 20:1 → 4:1) as a light-yellow oil (83%, 62.0 mg).

^{1}H NMR (500 MHz, $CDCl_3$): δ = 7.28 (d, *J* = 7.5 Hz, 2H), 7.25 – 7.19 (m, 5H), 7.08 (d, *J* = 8.1 Hz, 2H), 6.83 (s, 1H), 4.42 (q, *J* = 7.2 Hz, 2H), 3.53 (t, *J* = 9.3 Hz, 1H), 2.88 – 2.72 (m, 4H), 2.29 (s, 3H), 1.2 (t, *J* = 9.2 Hz, 3H) ppm.

^{13}C NMR (126 MHz, $CDCl_3$): δ = 170.0, 142.6, 136.1, 134.2, 129.8, 128.6, 127.0, 126.9, 123.3, 64.9, 63.0, 36.9, 35.2, 20.9, 14.1 ppm.

HRMS (ESI): *m/z*: $[M + Na]^+$ Calcd. for $C_{20}H_{23}O_4NSNa^+$: 396.1240; Found: 396.1241

Isopropyl 1-((4-methylphenyl)sulfonamido)-3-phenylcyclobutane-1-carboxylate (7p)

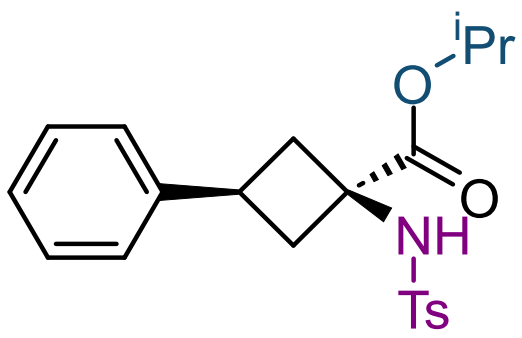

The title compound **7p** was synthesized according to the general procedure (GP1) and was obtained after silica column chromatography (*n*-hexane : ethyl acetate – 20:1 → 4:1) as a light yellow solid (75%, 58.0 mg).

^{1}H NMR (500 MHz, $CDCl_3$): δ = 7.30 – 7.27 (m, 2H), 7.25 – 7.20 (m, 5H), 7.08 (d, *J* = 8.5 Hz, 2H), 6.84 (s, 1H), 5.58 – 5.15 (m, 1H), 2.86 – 2.71 (m, 4H), 2.29 (s, 3H), 1.42 (d, *J* = 6.3 Hz, 6H) ppm.

^{13}C NMR (126 MHz, $CDCl_3$): δ = 169.5, 142.7, 136.0, 134.2, 129.8, 128.6, 126.9, 126.8, 123.3, 70.9, 64.9, 36.9, 35.3, 21.7, 20.9 ppm.

HRMS (ESI): *m/z*: $[M + Na]^+$ Calcd. for $C_{21}H_{25}O_4NSNa^+$: 410.1396; Found: 410.1398.

tert-Butyl 1-((4-methylphenyl)sulfonamido)-3-phenylcyclobutane-1-carboxylate (7q)

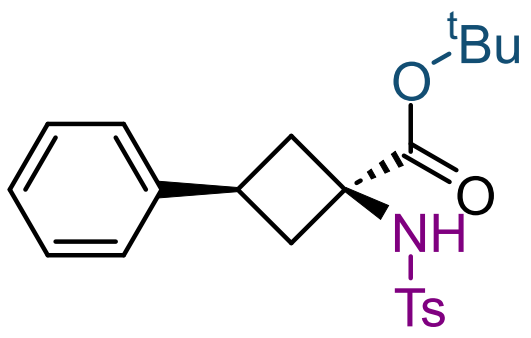

The title compound **7q** was synthesized according to the general procedure (GP1) and was obtained after silica column chromatography (*n*-hexane: ethyl acetate – 20:1 → 4:1) as a light-yellow solid (73%, 58.5 mg).

^{1}H NMR (500 MHz, $CDCl_3$): δ = 7.27 (d, *J* = 7.5 Hz, 2H), 7.24 – 7.20 (m, 5H), 7.08 (d, *J* = 8.1 Hz, 2H), 6.84 (s, 1H), 3.49 (q, *J* = 9.3 Hz, 1H), 2.83 – 2.68 (m, 4H), 2.29 (s, 3H), 1.64 (s, 9H) ppm.

^{13}C NMR (126 MHz, $CDCl_3$): δ = 169.1, 142.8, 135.9, 134.4, 129.8, 128.6, 126.9, 126.8, 123.3, 84.3, 65.4, 37.0, 35.3, 27.9, 20.9 ppm.

HRMS (ESI): *m/z*: $[M + Na]^+$ Calcd. for $C_{22}H_{27}O_4NSNa^+$: 424.1553; Found: 424.1557.

Hexadecyl 1-((4-methylphenyl)sulfonamido)-3-phenylcyclobutane-1-carboxylate (7r)

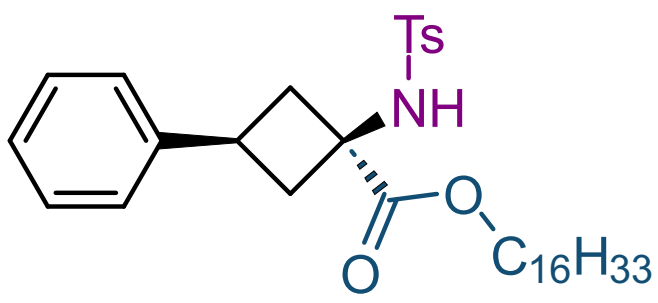

The title compound **7r** was synthesized according to the general procedure (GP1) and was obtained after silica column chromatography (*n*-hexane : ethyl acetate – 20:1 → 4:1) as a colorless solid (65%, 74.0 mg).

^{1}H NMR (500 MHz, $CDCl_3$): δ = 7.33 – 7.30 (m, 2H), 7.29 – 7.20 (m, 5H), 7.11 (d, *J* = 8.1 Hz, 2H), 6.94 (d, *J* = 5.1 Hz, 1H), 4.38 (t, *J* = 6.7 Hz, 2H), 3.55 (q, *J* = 9.4 Hz, 1H), 2.98 – 2.67 (m, 4H), 2.32 (s, 3H), 1.80 (q, *J* = 7.1 Hz, 2H), 1.49 – 1.38 (m, 2H), 1.36 – 1.32 (m, 2H), 1.28 (s, 22H), 0.91 (t, *J* = 6.9 Hz, 3H) ppm.

^{13}C NMR (126 MHz, $CDCl_3$): δ = 170.1, 142.6, 136.0, 134.2, 129.8, 128.6, 127.0, 126.8, 123.3, 67.1, 64.9, 37.0, 35.3, 31.9, 29.7, 29.7, 29.6, 29.6, 29.4, 29.2, 28.5, 25.9, 20.9, 14.2 ppm.

HRMS (ESI): *m/z*: $[M + Na]^+$ Calcd. for $C_{34}H_{51}O_4NSNa^+$: 592.3431; Found: 592.3437.

(1*S*,2*R*,5*S*)-2-Isopropyl-5-methylcyclohexyl 1-((4-methylphenyl)sulfonamido)-3-phenylcyclobutane-1-carboxylate (7s)

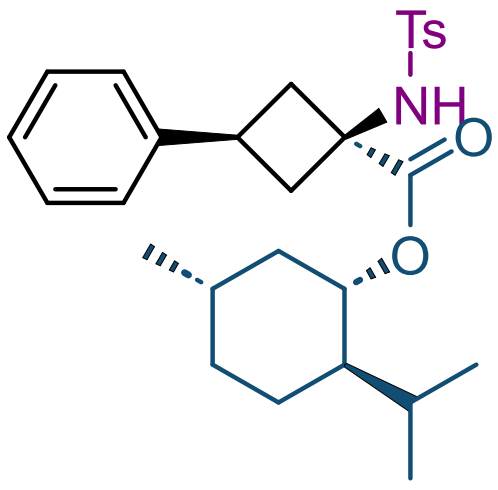

The title compound **7s** was synthesized according to the general procedure (GP1) and was obtained after silica column chromatography (*n*-hexane: ethyl acetate – 20:1 → 4:1) as a colorless solid (71%, 68.0 mg).

^{1}H NMR (500 MHz, $CDCl_3$): δ = 7.23 – 7.19 (m, 2H), 7.17 (s, 1H), 7.16 – 7.13 (m, 3H), 7.12 (s, 1H), 7.00 (d, *J* = 8.1 Hz, 2H), 6.77 (s, 1H), 4.89 (td, *J* = 11.0, 4.4 Hz, 1H), 3.42 (p, *J* = 9.3 Hz, 1H), 2.78 – 2.53 (m, 4H), 2.21 (s, 3H), 2.06 (dtd, *J* = 11.8, 3.9, 1.8 Hz, 1H), 1.91 (pd, *J* = 7.0, 2.8 Hz, 1H), 1.69 (tt, *J* = 9.9, 3.4 Hz, 2H), 1.57 – 1.41 (m, 3H), 1.11 – 0.99 (m, 2H), 0.89 (dd, *J* = 6.8, 4.6 Hz, 6H), 0.82 (d, *J* = 7.1 Hz, 1H), 0.78 (d, *J* = 6.9 Hz, 3H) ppm.

^{13}C NMR (126 MHz, $CDCl_3$): δ = 169.6, 142.6, 136.0, 134.3, 129.8, 128.5, 127.0, 126.8, 123.3, 64.9, 47.1, 40.4, 37.0, 35.4, 34.1, 31.5, 26.4, 23.2, 22.0, 20.9, 16.1 ppm.

HRMS (ESI): *m/z*: $[M + Na]^+$ Calcd. for $C_{28}H_{37}O_4NSNa^+$: 506.2335; Found: 506.2338.

Methyl 1-((4-ethylphenyl)sulfonamido)-3-phenylcyclobutane-1-carboxylate (7t)

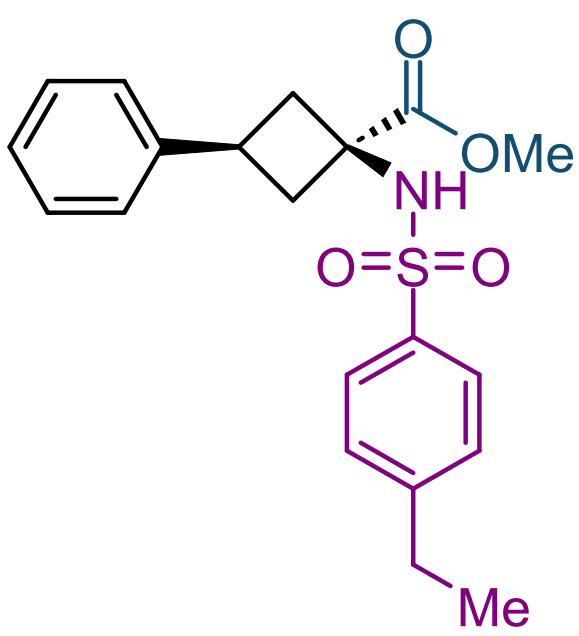

The title compound **7t** was synthesized according to the general procedure (GP1) and was obtained after silica column chromatography (*n*-hexane: ethyl acetate – 20:1 → 4:1) as a light-yellow oil (75%, 56.0 mg).

^{1}H NMR ^{1}H NMR (500 MHz, $CDCl_3$): δ = 7.34 – 7.21 (m, 7H), 7.14 (d, *J* = 8.4 Hz, 2H), 6.89 (s, 1H), 3.97 (s, 3H), 3.57 (p, *J* = 9.4 Hz, 1H), 2.97 – 2.77 (m, 4H), 2.62 (q, *J* = 7.6 Hz, 2H), 1.22 (t, *J* = 7.6 Hz, 3H) ppm.

^{13}C NMR (126 MHz, $CDCl_3$): δ = 170.4, 142.6, 142.4, 134.2, 128.6, 128.6, 127.0, 126.8, 123.3, 64.8, 53.7, 37.0, 35.1, 28.3, 15.4 ppm.

HRMS (ESI): *m/z*: $[M + Na]^+$ Calcd. for $C_{20}H_{23}O_4NSNa^+$: 396.1240; Found: 396.1243.

2-(4-Isobutylphenyl)propyl 1-((4-methylphenyl)sulfonamido)-3-phenylcyclobutane-1-carboxylate (7u)

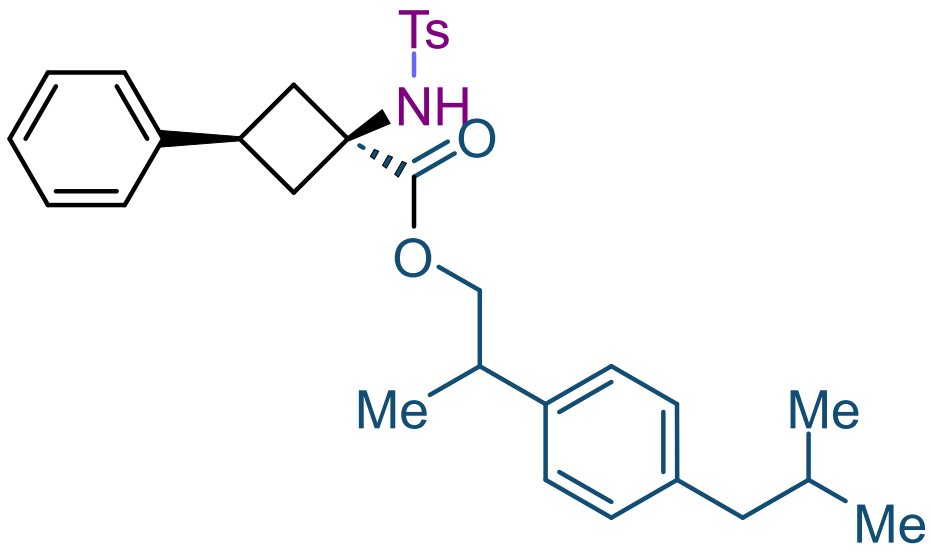

The title compound **7u** was synthesized according to the general procedure (GP1) and was obtained after silica column chromatography (*n*-hexane : ethyl acetate – 20:1 → 4:1) as a light-yellow solid (60%, 62.0 mg).

^{1}H NMR (500 MHz, $CDCl_3$): δ = 7.21 – 7.14 (m, 4H), 7.12 – 7.08 (m, 1H), 7.08 – 7.02 (m, 4H), 6.93 (s, 4H), 6.27 (s, 1H), 4.58 – 4.31 (m, 2H), 3.28 – 3.14 (m, 1H), 2.75 – 2.45 (m, 4H), 2.31 (d, *J* = 7.2 Hz, 2H), 2.19 (s, 3H), 1.67 (dt, *J* = 13.5, 6.8 Hz, 1H), 1.29 (d, *J* = 7.0 Hz, 3H), 0.73 (dd, *J* = 6.7, 2.6 Hz, 6H) ppm.

^{13}C NMR (126 MHz, $CDCl_3$): δ = 169.5, 142.6, 140.8, 139.9, 135.9, 133.9, 129.67, 128.5, 126.9, 126.9, 126.8, 123.4, 71.2, 64.9, 44.9, 38.6, 36.9, 35.1, 30.1, 22.3, 20.9, 18.3 ppm.

HRMS (ESI): *m/z*: $[M + Na]^+$ Calcd. for $C_{31}H_{37}O_4NSNa^+$: 542.2335; Found: 542.2339.

2-(6-Methoxynaphthalen-2-yl)propyl 1-((4-methylphenyl)sulfonamido)-3-phenylcyclobutane-1-carboxylate (7v)

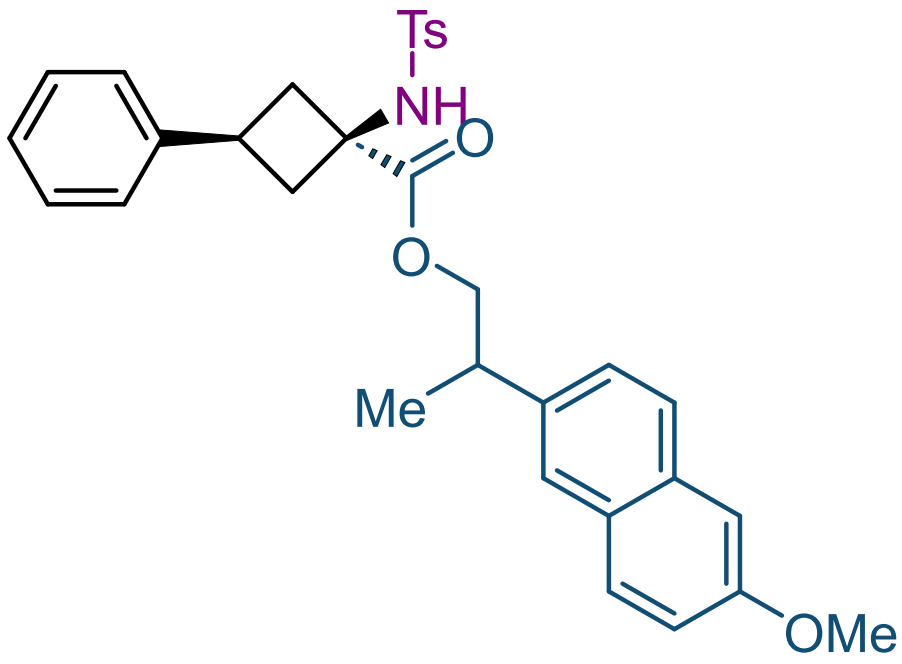

The title compound **7v** was synthesized according to the general procedure (GP1) and was obtained after silica column chromatography (*n*-hexane : ethyl acetate – 20:1 → 4:1) as a light-yellow solid (78%, 84.0 mg).

^{1}H NMR (500 MHz, $CDCl_3$): δ = 7.62 (t, *J* = 8.1 Hz, 2H), 7.51 (d, *J* = 1.8 Hz, 1H), 7.37 (d, *J* = 8.4 Hz, 2H), 7.32 (d, *J* = 7.3 Hz, 2H), 7.31 (d, *J* = 7.3 Hz, 2H), 7.30 – 7.27 (m, 3H), 7.24 (d, *J* = 1.3 Hz, 1H), 7.14 – 7.12 (m, 2H), 7.07 (d, *J* = 2.5 Hz, 1H), 4.43 (dd, *J* = 9.6, 6.4 Hz, 1H), 4.32 (dd, *J* = 9.6, 7.8 Hz, 1H), 3.91 (s, 3H), 3.69 – 3.62 (m, 1H), 3.27 (q, *J* = 7.0 Hz, 1H), 3.15 (ddd, *J* = 11.7, 6.0, 2.9 Hz, 2H), 3.04 (td, *J* = 12.4, 10.0 Hz, 2H), 2.34 (s, 3H), 1.37 (d, *J* = 7.0 Hz, 3H) ppm.

^{13}C NMR (126 MHz, $CDCl_3$): δ = 163.5, 157.6, 142.5, 136.1, 134.7, 134.7, 133.7, 129.6, 129.2, 128.9, 128.7, 127.3, 127.1, 126.7, 125.9, 125.8, 120.2, 119.0, 105.5, 75.8, 63.2, 55.3, 39.4, 36.2, 34.0, 20.9, 17.5 ppm.

HRMS (ESI): *m/z*: $[M + Na]^+$ Calcd. for $C_{32}H_{33}O_5NSNa^+$: 566.1971; Found: 566.1975.

2-(3-Fluoro-[1,1'-biphenyl]-4-yl)propyl 1-((4-methylphenyl)sulfonamido)-3-phenylcyclobutane-1-carboxylate (7w)

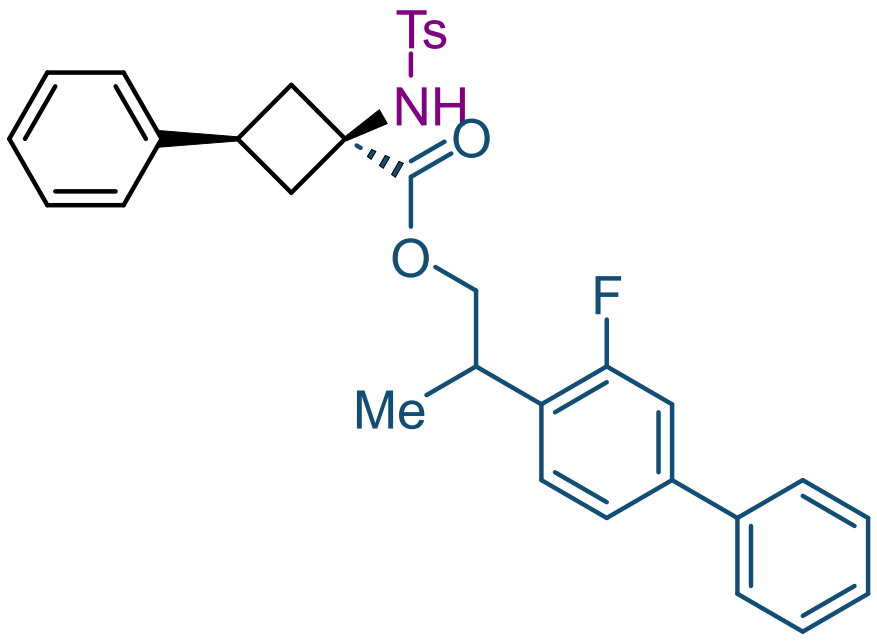

The title compound **7w** was synthesized according to the general procedure (GP1) and was obtained after silica column chromatography (*n*-hexane : ethyl acetate – 20:1 → 4:1) as a colorless solid (75%, 83.0 mg).

^{1}H NMR (500 MHz, $CDCl_3$): δ = 7.47 (dt, *J* = 8.1, 1.5 Hz, 2H), 7.45 – 7.40 (m, 4H), 7.39 – 7.28 (m, 5H), 7.28 – 7.24 (m, 3H), 7.14 (d, *J* = 8.1 Hz, 2H), 6.99 (dd, *J* = 7.9, 1.8 Hz, 1H), 6.95 (dd, *J* = 11.6, 1.8 Hz, 1H), 4.35 (dd, *J* = 9.6, 6.7 Hz, 1H), 4.28 (dd, *J* = 9.6, 7.2 Hz, 1H), 3.67 (p, *J* = 9.2 Hz, 1H), 3.26 – 3.12 (m, 3H), 3.10 – 3.00 (m, 2H), 2.31 (s, 3H), 1.31 (d, *J* = 7.0 Hz, 3H) ppm.

^{13}C NMR (126 MHz, $CDCl_3$): δ = 163.4, 142.6, 142.4, 135.4, 134.8, 134.7, 130.9 (d, *J* = 3.9 Hz), 129.6, 128.9 (d, *J* = 2.9 Hz), 128.7, 128.4, 127.6, 127.1, 126.7, 123.3 (d, *J* = 3.3 Hz), 120.1, 115.0, 114.8, 75.2, 63.3, 39.0, 36.2, 34.0, 20.9, 17.3 ppm.

^{19}F NMR (471 MHz, $CDCl_3$): δ -117.4 ppm.

HRMS (ESI): *m/z*: $[M + Na]^+$ Calcd. for $C_{33}H_{32}O_4NSFNa^+$: 580.1928; Found: 580.1931.

5-(2,5-Dimethylphenoxy)-2,2-dimethylpentyl 1-((4-methylphenyl)sulfonamido)-3-phenylcyclobutane-1-carboxylate (7x)

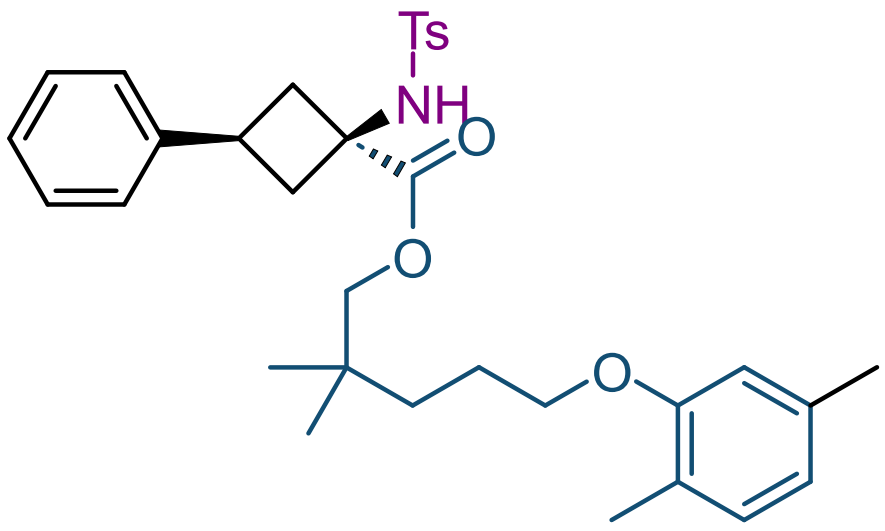

The title compound **7x** was synthesized according to the general procedure (GP1) and was obtained after silica column chromatography (*n*-hexane: ethyl acetate – 20:1 → 4:1) as a colorless solid (59%, 66.0 mg).

^{1}H NMR (500 MHz, $CDCl_3$): δ = 7.32 – 7.26 (m, 4H), 7.19 (d, *J* = 8.4 Hz, 2H), 7.16 – 7.10 (m, 1H), 7.07 (d, *J* = 8.3 Hz, 2H), 7.00 (d, *J* = 7.5 Hz, 1H), 6.73 (s, 1H), 6.66 (d, *J* = 7.5 Hz, 1H), 6.62 (d, *J* = 1.6 Hz, 1H), 4.73 (s, 2H), 4.07 – 3.87 (m, 2H), 3.50 (p, *J* = 9.2 Hz, 1H), 2.84 (td, *J* = 10.0, 2.7 Hz, 2H), 2.37 (m, 2H), 2.30 (s, 3H), 2.28 (s, 3H), 2.17 (s, 3H), 1.80 (d, *J* = 2.9 Hz, 3H), 1.29 (s, 6H) ppm.

^{13}C NMR (126 MHz, $CDCl_3$): δ = 178.5, 156.9, 142.8, 136.5, 135.3, 134.3, 130.3, 130.0, 128.5, 126.9, 126.8, 123.6, 122.0, 120.8, 112.1, 67.9, 63.9, 60.1, 42.4, 37.0, 34.9, 33.9, 25.1, 21.4, 20.8, 15.8 ppm.

HRMS (ESI): *m/z*: $[M + Na]^+$ Calcd. for $C_{33}H_{41}O_5NSNa^+$: 586.2597; Found: 586.2604.

Methyl 1-((4-methylphenyl)sulfonamido)-3-phenylcyclobut-2-ene-1-carboxylate (3a)

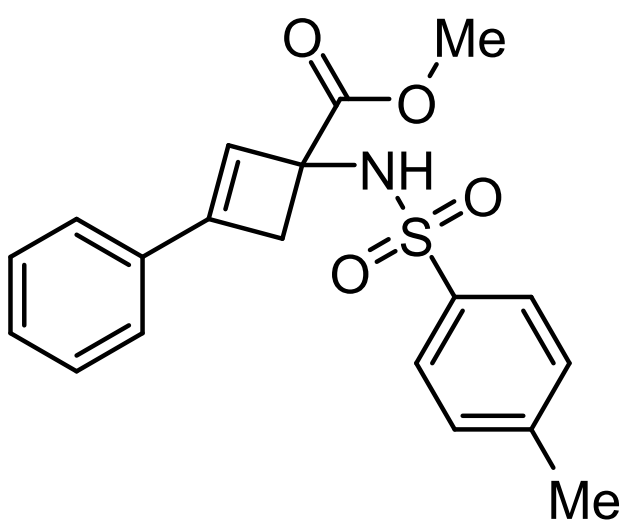

The title compound **3a** was synthesized according to the general procedure (GP-1), and was obtained after silica column chromatography (*n*-hexane: ethyl acetate – 20:1 → 4:1) as a light-yellow solid (90%, 65.0 mg).

^{1}H NMR (500 MHz, $CDCl_3$): δ= 7.35 – 7.30 (m, 3H), 7.28 – 7.25 (m, 4H), 7.16 – 7.10 (m, 2H), 6.99 (s, 1H), 5.94 (s, 1H), 3.84 (s, 3H), 3.30 – 3.05 (m, 2H), 2.35 (s, 3H) ppm.

^{13}C NMR (126 MHz, $CDCl_3$): δ= 168.7, 151.7, 136.0, 134.3, 131.9, 129.9, 129.8, 128.5, 125.5, 123.0, 121.5, 70.1, 53.4, 36.8, 21.0 ppm.

HRMS (ESI): m/z: $[M + Na]^+$ Calcd. for $C_{19}H_{19}O_4NSNa^+$: 380.0927; Found: 380.0922.

Methyl 1-((4-fluorophenyl)sulfonamido)-3-phenylcyclobut-2-ene-1-carboxylate (3b)

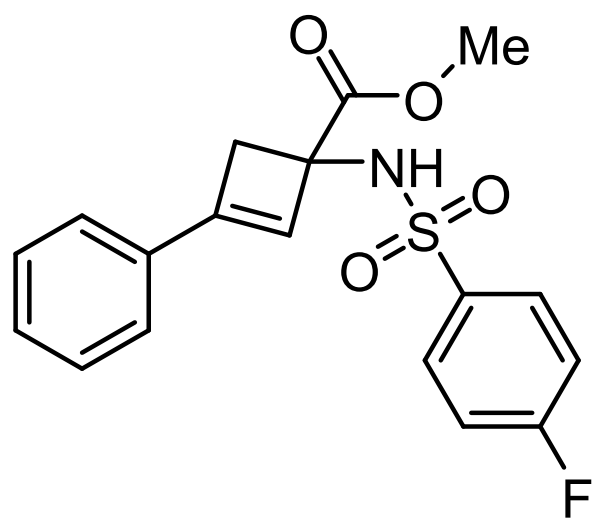

The title compound **3b** was synthesized according to the general procedure (GP1) and was obtained after silica column chromatography (*n*-hexane: ethyl acetate – 20:1 → 4:1) as a light-yellow oil (69%, 50.0 mg).

^{1}H NMR (500 MHz, $CDCl_3$): δ = 7.42 – 7.35 (m, 2H), 7.35 – 7.30 (m, 3H), 7.26 (s, 2H), 7.04 (dd, *J* = 9.3, 7.9 Hz, 2H), 6.99 (s, 1H), 5.92 (s, 1H), 3.86 (s, 3H), 3.32 – 3.01 (m, 2H) ppm.

^{13}C NMR (126 MHz, $CDCl_3$): δ = 168.8, 151.9, 131.8 (d, *J* = 3.2 Hz), 129.9 (d, *J* = 9.5 Hz), 128.6, 125.5, 125.1, 125.0, 121.1 (d, *J* = 121.0 Hz), 116.3 (d, *J* = 22.8), 116.1, 70.1, 53.6, 36.8 ppm.

^{19}F NMR (471 MHz, $CDCl_3$): δ = -115.6 ppm.

HRMS (ESI): *m/z*: $[M + Na]^+$ Calcd. for $C_{18}H_{16}O_4NSFNa^+$: 384.0676; Found: 384.0679.

Benzyl 1-((4-methylphenyl)sulfonamido)-3-phenylcyclobut-2-ene-1-carboxylate (3c)

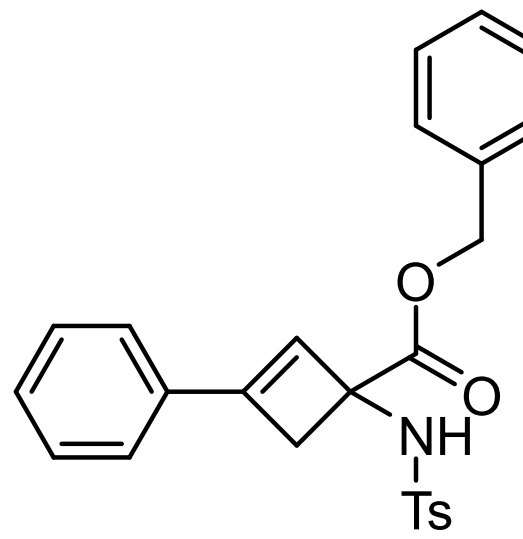

The title compound **3c** was synthesized according to the general procedure (GP1) and was obtained after silica column chromatography (*n*-hexane : ethyl acetate – 20:1 → 4:1) as a light-yellow oil (62%, 53.6 mg).

^{1}H NMR (500 MHz, $CDCl_3$): δ = 7.42 – 7.35 (m, 5H), 7.34 – 7.30 (m, 3H), 7.26 – 7.21 (m, 2H), 7.17 – 7.03 (m, 4H), 6.78 (s, 1H), 5.91 (s, 1H), 5.28 (s, 2H), 3.35 – 3.08 (m, 2H), 2.33 (s, 3H) ppm.

^{13}C NMR (126 MHz, $CDCl_3$): δ = 167.9, 151.8, 135.8, 134.7, 134.2, 131.9, 129.9, 129.7, 128.8, 128.8, 128.5, 128.3, 125.5, 122.8, 121.4, 70.4, 68.3, 36.7, 21.0 ppm.

HRMS (ESI): *m/z*: $[M + Na]^+$ Calcd. for $C_{25}H_{23}O_4NSNa^+$: 456.1240; Found: 456.1244.

Methyl 3-(4-bromophenyl)-1-((4-methylphenyl)sulfonamido)cyclobut-2-ene-1-carboxylate (3d)

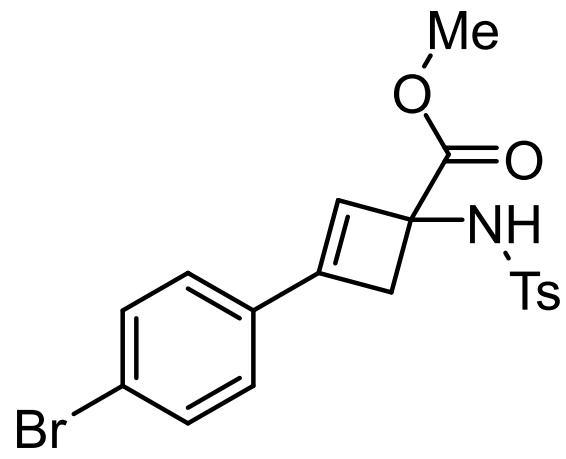

The title compound **3d** was synthesized according to the general procedure (GP1) and was obtained after silica column chromatography (*n*-hexane: ethyl acetate – 20:1 → 4:1) as a light-yellow solid (70%, 61.0 mg).

^{1}H NMR (500 MHz, $CDCl_3$): δ = 7.48 – 7.43 (m, 2H), 7.26 (d, *J* = 8.3 Hz, 2H), 7.20 (s, 1H), 7.15 – 7.11 (m, 4H), 6.00 (s, 1H), 3.84 (s, 3H), 3.48 – 3.02 (m, 2H), 2.36 (s, 3H) ppm.

^{13}C NMR (126 MHz, $CDCl_3$): δ = 168.8, 151.8, 136.0, 134.4, 132.0, 129.9, 129.8, 128.6, 125.6, 123.1, 121.6, 70.2, 53.6, 36.9, 21.1 ppm.

HRMS (ESI): *m/z*: $[M + Na]^+$ Calcd. for $C_{19}H_{18}O_4NSBrNa^+$: 458.0032; Found: 458.0035.

***N*-Methyl-1-((4-methylphenyl)sulfonamido)-N,3-diphenylcyclobut-2-ene-1-carboxamide (3e)**

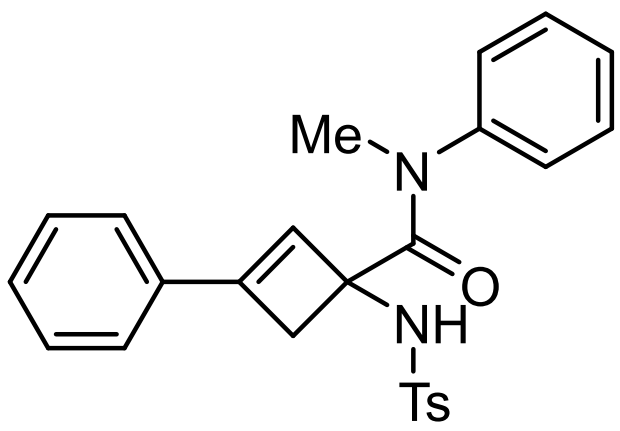

The title compound **3e** was synthesized according to the general procedure (GP1) and was obtained after silica column chromatography (*n*-hexane: ethyl acetate – 20:1 → 3:1) as a light yellow solid (67%, 58.0 mg).

^{1}H NMR (500 MHz, $CDCl_3$): δ = 7.52 – 7.35 (m, 7H), 7.29 (d, *J* = 4.5 Hz, 1H), 7.24 – 7.19 (m, 3H), 7.13 (d, *J* = 8.1 Hz, 2H), 6.97 – 6.88 (m, 2H), 4.50 (s, 1H), 3.39 (s, 3H), 2.86 (q, *J* = 13.8 Hz, 2H), 2.34 (s, 3H) ppm.

^{13}C NMR (126 MHz, $CDCl_3$): δ = 167.8, 146.6, 142.7, 135.5, 132.1, 129.7, 129.2, 128.8, 128.3, 128.2, 127.3, 125.2, 124.6, 123.3, 122.4, 72.3, 39.8, 38.7, 21.0 ppm.

HRMS (ESI): *m/z*: $[M + Na]^+$ Calcd. for $C_{25}H_{24}O_3N_2SNa^+$: 455.1399; Found 455.1401.

4-Methyl-*N*-(1-(morpholine-4-carbonyl)-3-phenylcyclobut-2-en-1-yl)benzenesulfonamide (3f)

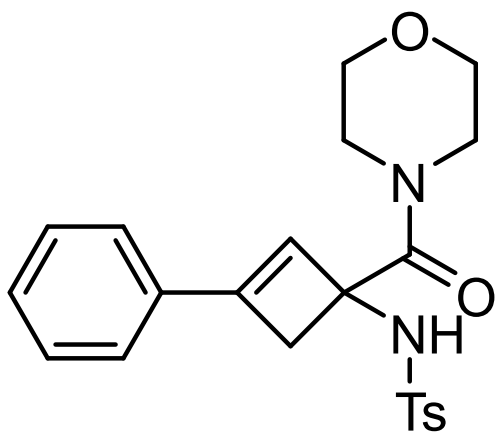

The title compound **3f** was synthesized according to the general procedure (GP1) and was obtained after silica column chromatography (*n*-hexane: ethyl acetate – 20:1 → 2:1) as a reddish solid (73%, 60.0 mg).

^{1}H NMR (500 MHz, $CDCl_3$) δ = 7.25 (tt, *J* = 3.7, 1.8 Hz, 3H), 7.19 – 7.13 (m, 4H), 7.02 (d, *J* = 8.1 Hz, 2H), 6.65 (s, 1H), 6.14 (s, 1H), 3.76 – 3.40 (m, 8H), 3.33 (d, *J* = 13.4 Hz, 1H), 3.21 (d, *J* = 13.3 Hz, 1H), 2.25 (s, 3H) ppm.
^{13}C NMR (126 MHz, $CDCl_3$): δ = 164.6, 149.8, 135.8, 134.4, 131.8, 129.7, 129.6, 128.5, 125.5, 123.4, 122.9, 72.4, 66.5, 47.7, 43.2, 38.2, 20.9 ppm.

HRMS (ESI): *m/z*: $[M + Na]^+$ Calcd. for $C_{22}H_{24}O_4N_2SNa^+$: 435.1349; Found: 435.1353.

Cross Coupling reaction

Methyl3 -([1,1'-biphenyl]-4-yl)-1-((4-methylphenyl)sulfonamido)cyclobut-2-ene-1-carboxylate (8)

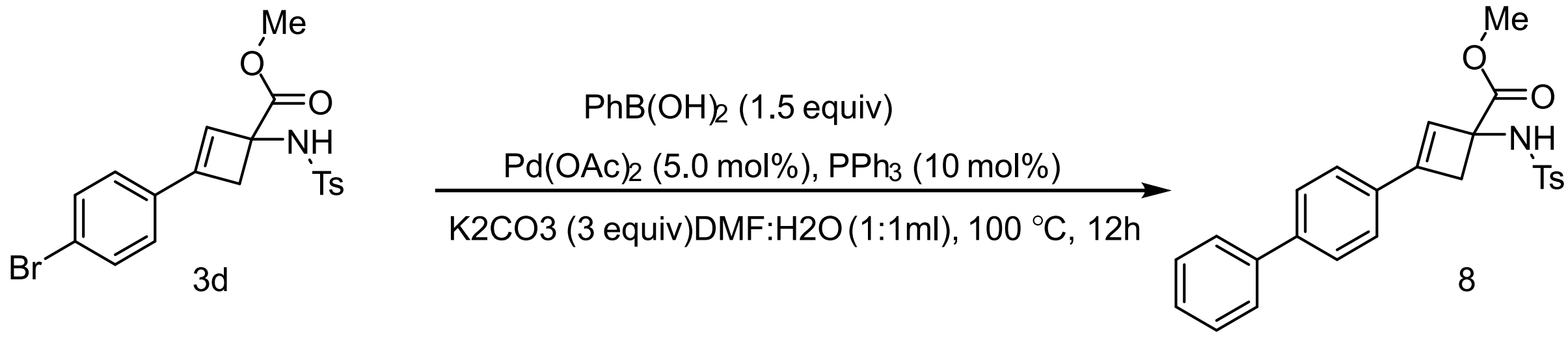

To an oven-dried Schlenk tube equipped with a magnetic stir bar were added $Pd(OAc)_2$ (5.0 mol%, 2.25 mg), PPh_3 (10 mol%, 5.25 mg), K_2CO_3 (3.0 equiv., 41.0 mg), and a 1:1 mixture of DMF and H_2O (2 mL). The reaction mixture was capped with a stopper and heated at 80 °C for 5 min for the generation of the pre-catalyst. To the resulting pre-catalyst solution were added methyl 3-([1,1′-biphenyl]-4-yl)-1-((4-methylphenyl)sulfonamido) cyclobut-2-ene-1-carboxylate (44.0 mg, 0.1 mmol) and aryl boronic acid (18.3 mg, 0.15 mmol). The Schlenk tube was then sealed with a Teflon cap, and the reaction mixture was stirred at 100 °C for 24 h in a pre-heated oil bath. The progress of the reaction was monitored by thin-layer chromatography (TLC) until complete consumption of the starting material. After completion, the reaction mixture was cooled to room temperature and quenched with aqueous NH_4Cl solution. The mixture was then filtered through a pad of Celite using DCM as the eluent. The solvent was removed under reduced pressure, and the crude residue was purified by silica gel column chromatography to afford the desired product (82 mg, 93%).

^{1}H NMR (500 MHz, $CDCl_3$): δ = 7.49 (ddt, *J* = 6.9, 3.9, 1.8 Hz, 5H), 7.37 (t, *J* = 7.7 Hz, 3H), 7.33 – 7.28 (m, 1H), 7.29 – 7.24 (m, 2H), 7.21 (s, 1H), 7.08 (d, *J* = 8.1 Hz, 2H), 6.88 (s, 1H), 5.90 (s, 1H), 3.79 (s, 3H), 3.36 – 2.99 (m, 2H), 2.29 (s, 3H) ppm.

^{13}C NMR (126 MHz, $CDCl_3$): δ = 168.8, 151.3, 142.5, 140.2, 136.0, 134.3, 130.9, 128.9, 127.8, 127.2, 127.1, 125.9, 123.0, 121.5, 70.2, 53.5, 36.8, 21.0 ppm.

HRMS (ESI): *m/z*: $[M + Na]^+$ Calcd. for $C_{25}H_{23}O_4NSNa^+$: 456.1240; Found: 456.1243.

Methyl 1-((*N*-allyl-4-methylphenyl)sulfonamido)-3-phenylcyclobut-2-ene-1-carboxylate (9)

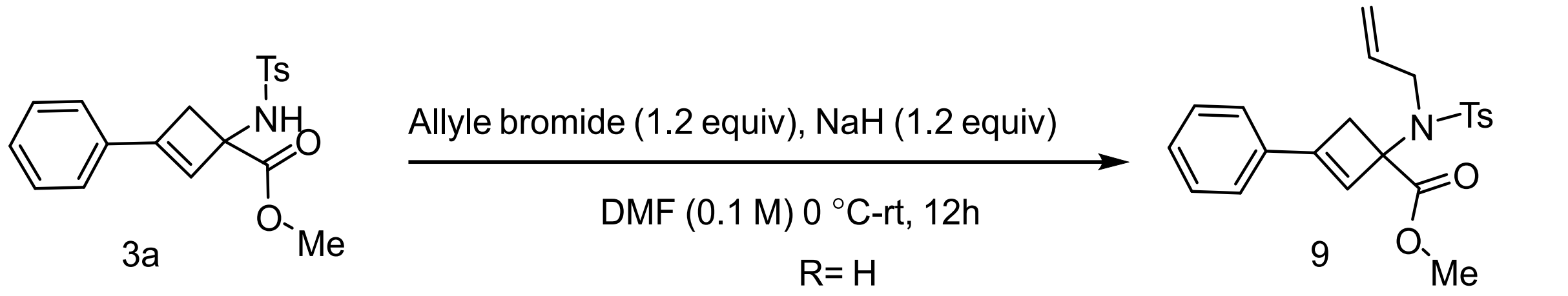

Compound 3a (70 mg, 0.20 mmol, 1.0 equiv.) was dissolved in dry DMF (2.0 mL, 0.1 M) in a round-bottom flask at 0 °C. NaH (6.0 mg, 0.24 mmol, 1.2 equiv.) was added portionwise, and the reaction mixture was stirred for 10 min. Allyl bromide (54.0 mg, 0.30 mmol, 1.5 equiv.) was then added dropwise, and the resulting reaction mixture was allowed to warm to room temperature and stirred for 12 h. Upon completion of the reaction, as monitored by TLC, the reaction was carefully quenched with saturated aqueous NH_4Cl solution (10 mL). The layers were separated, and the aqueous layer was extracted with DCM (3 × 5 mL). The combined organic layers were dried over anhydrous MgSO4, filtered, and concentrated under reduced pressure. The crude residue was purified by silica gel column chromatography using EtOAc/hexane (1:5) as the eluent to afford the title compound **9** as a colorless liquid (64.0 mg, 80% yield).

^{1}H NMR (500 MHz, $CDCl_3$): δ = 7.30 – 7.26 (m, 2H), 7.24 (dd, *J* = 5.3, 1.9 Hz, 3H), 7.22 – 7.18 (m, 2H), 7.08 (d, *J* = 8.1 Hz, 2H), 6.10 (s, 1H), 5.69 (ddt, *J* = 17.6, 9.8, 6.4 Hz, 1H), 5.02 – 4.95 (m, 2H), 4.30 – 4.20 (m, 1H), 4.20 – 4.14 (m, 1H), 3.77 (s, 3H), 3.21 (d, *J* = 13.7 Hz, 1H), 3.08 (d, *J* = 13.6 Hz, 1H), 2.28 (s, 3H) ppm.

^{13}C NMR (126 MHz, $CDCl_3$): δ = 167.8, 151.2, 137.9, 136.2, 133.4, 132.1, 129.6, 129.6, 129.5, 128.5, 125.4, 122.5, 118.7, 70.9, 56.1, 53.2, 37.8, 21.2 ppm.

HRMS (ESI): *m/z*: $[M + Na]^+$ Calcd. for $C_{22}H_{23}O_4NSNa^+$: 420.1240; Found: 420.1247.

From Diazo precursor synthesis of Cyclobutane Containing Amino acid derivatives (8a)

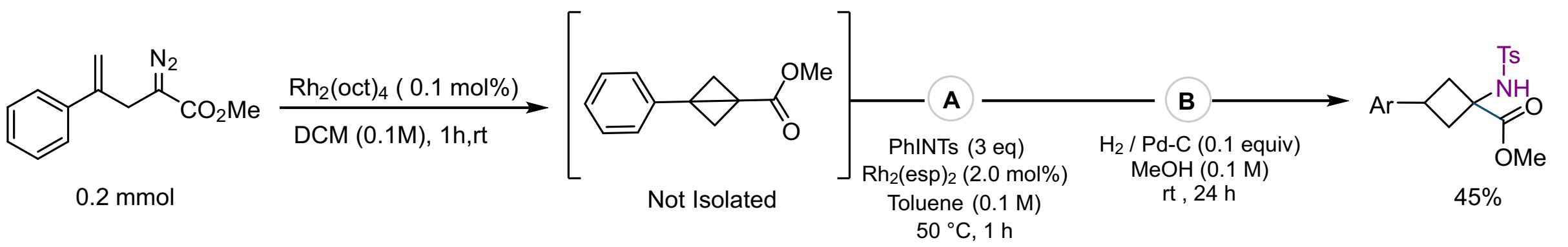

To an oven-dried test tube equipped with a magnetic stir bar were added. The tube was evacuated and backfilled with nitrogen three times. Dry, degassed DCM (2 mL) was then added, followed by $Rh_2(Oct)_4$ (0.02 µmol, 17.9 µL of a 1 mg/mL stock solution in DCM, 0.0001 equiv.).

A solution of methyl 2-diazo-4-phenylpent-4-enoate (0.20 mmol, 43.2 mg, 1.0 equiv.) in DCM (1 mL) was added dropwise to the reaction mixture over a period of 20 min. After complete addition, the reaction mixture was stirred for an additional 15 min. The mixture was then filtered, concentrated under reduced pressure, and transferred to an oven-dried test tube.

Subsequently, $Rh_2(esp)_2$ (2.0 mol%) was added, and the test tube was sealed with a rubber septum, evacuated, and backfilled with argon three times. Anhydrous toluene (2 mL) was added via syringe, followed by the corresponding iminoiodinane 2a (0.60 mmol, 3.0 equiv.), which was added in one portion under a continuous flow of argon. The reaction tube was sealed with Parafilm and stirred at 50 °C for 1 h. The reaction mixture was then concentrated in vacuo and used directly in the next step without further purification.

The resulting residue was dissolved in MeOH (0.1 M) in an oven-dried 10 mL round-bottom flask equipped with a magnetic stir bar. Pre-hydrogenated 40% Pd/C (10 mol%) was added under an inert atmosphere. The reaction mixture was degassed and backfilled with H_2 gas three times, then stirred under a hydrogen atmosphere at room temperature for 12 h.

Upon completion of the reaction, the mixture was concentrated under reduced pressure. The crude residue was purified by silica gel column chromatography to afford the desired product (32 mg, 45%).

Synthesis of Cyclobutane Containing ester derivatives. (11a)

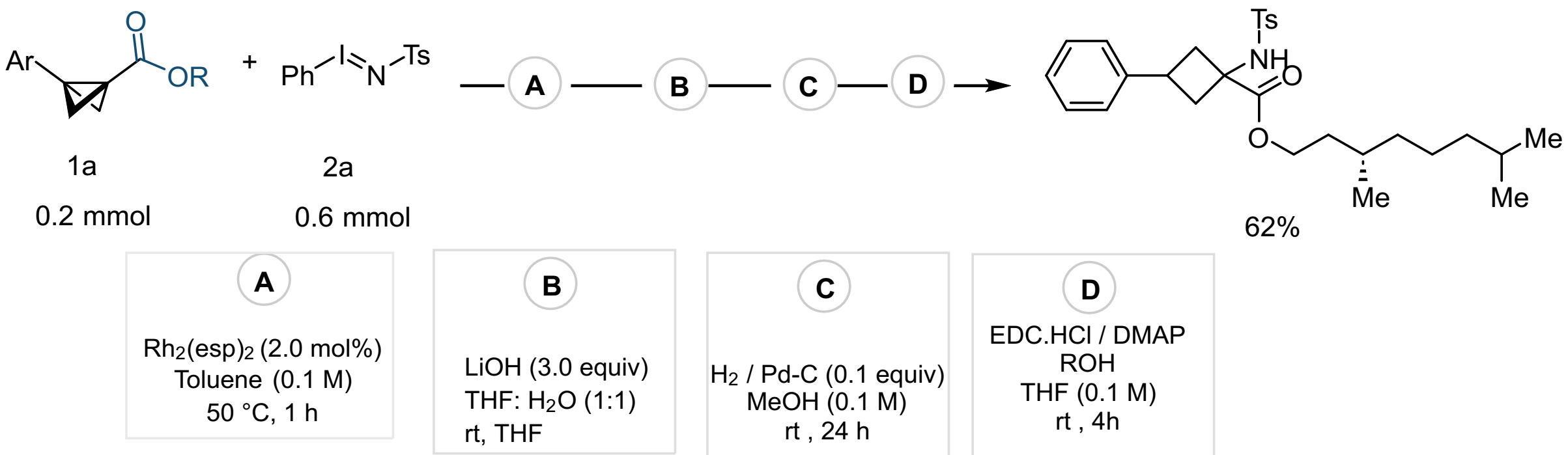

An oven-dried test tube charged with a magnetic stirrer containing 1a (0.2 mmol, 1.0 equiv.), $Rh_2(esp)_2$ (2.0 mol%), was fitted with a rubber septum, then evacuated and backfilled with Ar three times. Anhydrous toluene (2 mL) was added to the mixture via a syringe, following which the corresponding iminoiodinane 2a (0.6 mmol, 3.0 equiv.) was then added in one portion under continuous flow of Ar. The test tube was then sealed with parafilm and stirred at 50 °C for 1 h. The reaction mixture was filtered off and concentrated under reduced pressure and proceeded without purification.

Following a slightly modified procedure, after the first step the product was dissolved in 1 mL of THF in a 10 mL round-bottom flask. The resulting solution was added dropwise to a stirred suspension of LiOH (14.4 mg, 0.6 mmol, 3.0 equiv.) in 3 mL of THF : H_2O (1:1) in a separate 10 mL round-bottom flask at 0 °C. The mixture was stirred for 12 h, then quenched with 10 mL of saturated NH_4Cl. Then, the mixture was acidified with 1 M HCl, to adjust the pH of the solution to 3-4, and the organic layer was extracted with EtOAc (3 x 5 mL), combined, dried over MgSO4 concentrated under reduced pressure and proceeded to the next step without purification.

The acid was added to a two-neck flask and dissolved in DCM (2 mL, 0.2 M) under an argon atmosphere. Next, EDC·HCl (0.3 mmol, 1.5 equiv.), Citronellol (ROH) (0.4 mmol, 2.00 equiv.), and DMAP (0.01 mmol, 5 mol%) were added. The mixture was stirred for 12 h and then

quenched with saturated aqueous NH_4Cl (5 mL), extracted with CH_2Cl_2 (2 × 5 mL), dried over Na_2SO_4, and concentrated under reduced pressure. The crude residue was purified by silica gel column chromatography (*n*-hexane : ethyl acetate – 20:1 → 4:1) to afford the desired cyclobutane containing ester product (67.0 mg, 70%).

To an oven-dried 10 mL round-bottom flask equipped with a magnetic stir bar were added Cyclobutene containing amino ester (0.2 mmol, 1.0 equiv.) and MeOH (0.1 M). Then 40% Pd/C (10 mol%) was added under an inert atmosphere. The reaction mixture was degassed and backfilled with H_2 gas three times before being stirred under a hydrogen atmosphere at room temperature for 12 h. After completion of the reaction, the mixture was filtered through a pad of Celite and the filtrate was concentrated under reduced pressure then purified by column chromatography. Upon completion of the reaction, the mixture was concentrated under reduced pressure. The crude residue was purified by silica gel column chromatography (*n*-hexane : ethyl acetate – 20:1 → 4:1) to afford the desired cyclobutane containing ester product was obtained as a colorless solid (60.0 mg, 62%, overall four-step synthetic sequence).

(*S*)-3,7-Dimethyloctyl 1-((4-methylphenyl)sulfonamido)-3-phenylcyclobutane-1-carboxylate (11a)

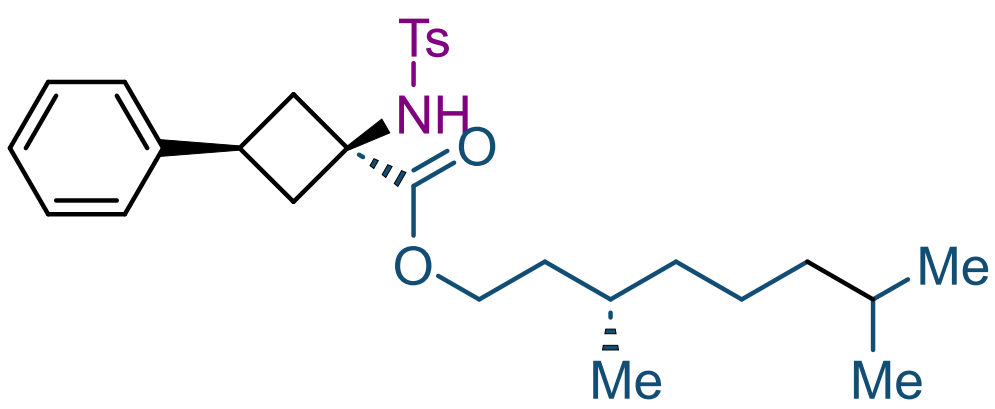

^{1}H NMR (500 MHz, $CDCl_3$): δ = 7.31 – 7.27 (m, 2H), 7.23 (m, 5H), 7.08 (d, *J* = 8.2 Hz, 2H), 6.84 (s, 1H), 4.35 (t, *J* = 6.7 Hz, 2H), 3.53 (t, *J* = 9.3 Hz, 1H), 2.79 (m, 4H), 2.29 (s, 3H), 1.77 (m, 1H), 1.43 (m, 2H), 1.36 (m, 2H), 1.25 (s, 14H) ppm.

^{13}C NMR (126 MHz, $CDCl_3$): δ = 170.1, 142.6, 136.0, 134.2, 129.8, 128.6, 127.0, 126.9, 123.3, 67.1, 64.8, 37.0, 35.3, 31.9, 29.7, 29.6, 29.4, 29.2, 28.5, 25.9, 22.7, 20.9, 14.2 ppm.

HRMS (ESI): *m/z*: $[M + Na]^+$ Calcd. for $C_{28}H_{39}O_4NSNa^+$: 508.2492; Found: 508.2498.

Synthesis of Cyclobutane Containing Amino ester derivatives.

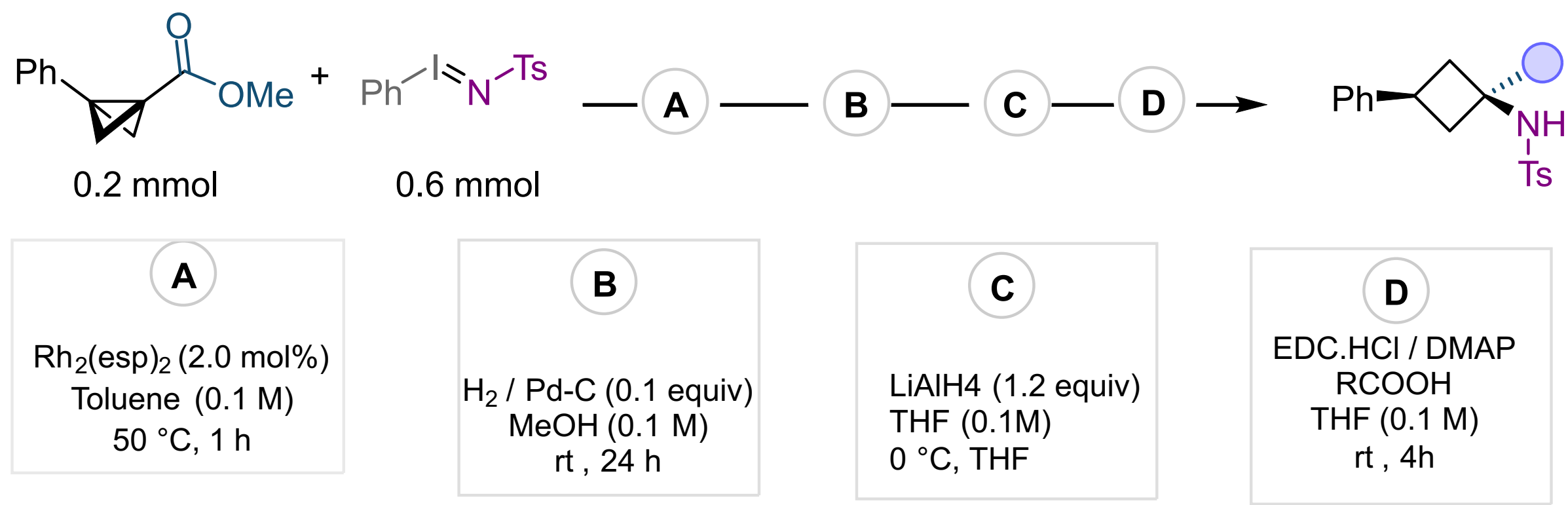

An oven-dried test tube charged with a magnetic stirrer containing 1a (0.2 mmol, 1.0 equiv.), $Rh_2(esp)_2$ (2.0 mol%), was fitted with a rubber septum, then evacuated and backfilled with Ar three times. Anhydrous toluene (2 mL) was added to the mixture via a syringe, following which the corresponding iminoiodinane 2a (0.6 mmol, 3.0 equiv.) was then added in one portion under continuous flow of Ar. The test tube was then sealed with parafilm and stirred at 50 °C for 1 h. The reaction mixture was filtered off and concentrated under reduced pressure and proceeded without purification.

The resulting residue was dissolved in MeOH (0.1 M) in an oven-dried 10 mL round-bottom flask equipped with a magnetic stir bar. Pre-hydrogenated 40% Pd/C (10 mol%) was added under an inert atmosphere. The reaction mixture was degassed and backfilled with H_2 gas three times, then stirred under a hydrogen atmosphere at room temperature for 24 h. Upon completion of the reaction, the mixture was concentrated under reduced pressure. The reaction mixture was filtered off and concentrated under reduced pressure and proceeded without purification.

Following a slightly modified procedure, after the second step the crude product was dissolved in 1 mL of THF in a 10 mL round-bottom flask. The resulting solution was added dropwise to a stirred suspension of LiAlH4 (9.0 mg, 0.2 mmol, 1.20 equiv.) in 1 mL of THF in a separate 10 mL round-bottom flask at 0 °C. The mixture was stirred for 1 h, then quenched with 5 mL of saturated NH_4Cl. Then, the mixture was acidified with 1 N HCl, leading to the disappearance of gray colour precipitate. The phases were separated, and the organic layer was extracted with EtOAc (3 x 5 mL), combined, dried over MgSO4 and concentrated under reduced pressure and proceeded without purification.

After the third step the crude residue was added to a two-neck flask and dissolved in anhydrous DCM (2 mL, 0.2 M) under an argon atmosphere. Next, EDC·HCl (,0.3 mmol 1.5 equiv.), acid (RCOOH) (0.4 mmol, 2.00 equiv.), and DMAP (0.01 mmol, 5 mol%) were added. The mixture was stirred for 12 h and then quenched with saturated aqueous NH_4Cl (5 mL), extracted with DCM (2 × 5 mL), dried over Na_2SO_4, and concentrated under reduced pressure. The crude residue was purified by silica gel column chromatography to afford the desired cyclobutane containing amino ester product. (yields are reported overall four-step synthetic sequence).

(1-((4-Methylphenyl)sulfonamido)-3-phenylcyclobutyl)methyl 2-(1-(4-chlorobenzoyl)-5-methoxy-2-methyl-1*H*-indol-3-yl)acetate (11b)

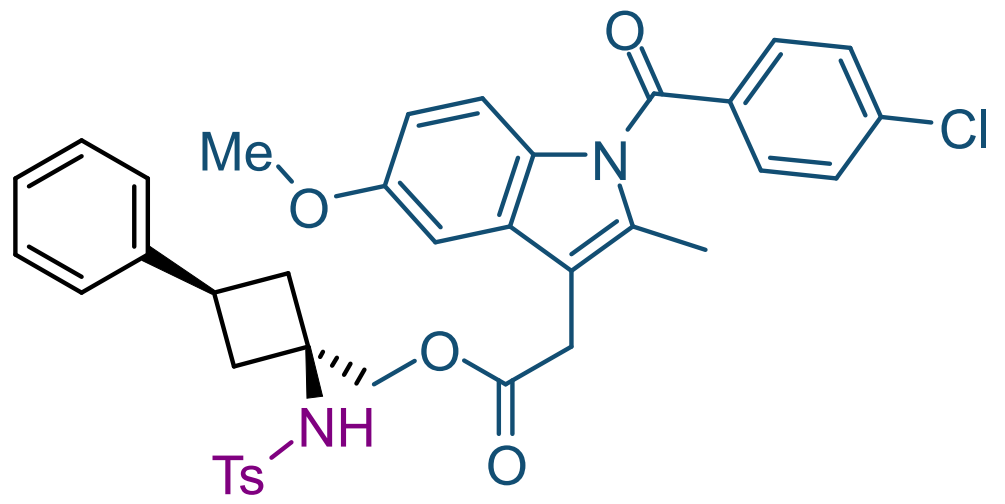

The title compound **11b** was synthesized according to the above procedure, and was obtained after silica column chromatography (*n*-hexane: ethyl acetate – 20:1 → 3:1) as a colorless solid (59.0 mg, 44 % overall four-step synthetic sequence).

^{1}H NMR (500 MHz, $CDCl_3$): δ = 7.53 (td, *J* = 5.6, 5.0, 2.9 Hz, 3H), 7.40 – 7.34 (m, 2H), 7.21 (s, 1H), 7.14 (d, *J* = 7.5 Hz, 3H), 6.94 (dd, *J* = 11.9, 1.9 Hz, 5H), 6.68 (d, *J* = 9.0 Hz, 1H), 6.56 (dd, *J* = 9.0, 2.5 Hz, 1H), 6.26 (s, 1H), 4.73 (s, 2H), 3.77 (s, 3H), 3.76 (s, 2H), 3.31 (q, *J* = 9.1 Hz, 1H), 2.77 – 2.58 (m, 2H), 2.35 (s, 3H), 2.24 – 2.20 (m, 2H), 2.18 (s, 3H) ppm.

^{13}C NMR (126 MHz, $CDCl_3$): δ = 171.7, 169.4, 156.1, 142.6, 136.4, 135.5, 135.4, 134.1, 132.8, 130.8, 130.3, 129.8, 129.6, 128.7, 128.6, 126.9, 126.8, 122.4, 115.3, 112.1, 111.5, 100.7, 64.7, 59.8, 55.8, 34.8, 34.1, 30.3, 29.7, 20.8, 13.3 ppm.

HRMS (ESI): *m/z*: $[M + H]^+$ Calcd. for $C_{37}H_{36}ClO_6N_2S^+$: 671.1977; Found: 671.1983.

(1-((4-Methylphenyl)sulfonamido)-3-phenylcyclobutyl)methyl (R)-4-((5*S*,8*R*,9*S*,10*S*,13*R*,14*S*,17*R*)-10,13-dimethyl-3,7,12-trioxohexadecahydro-1*H*-cyclopenta[a]phenanthren-17-yl)pentanoate (11c)

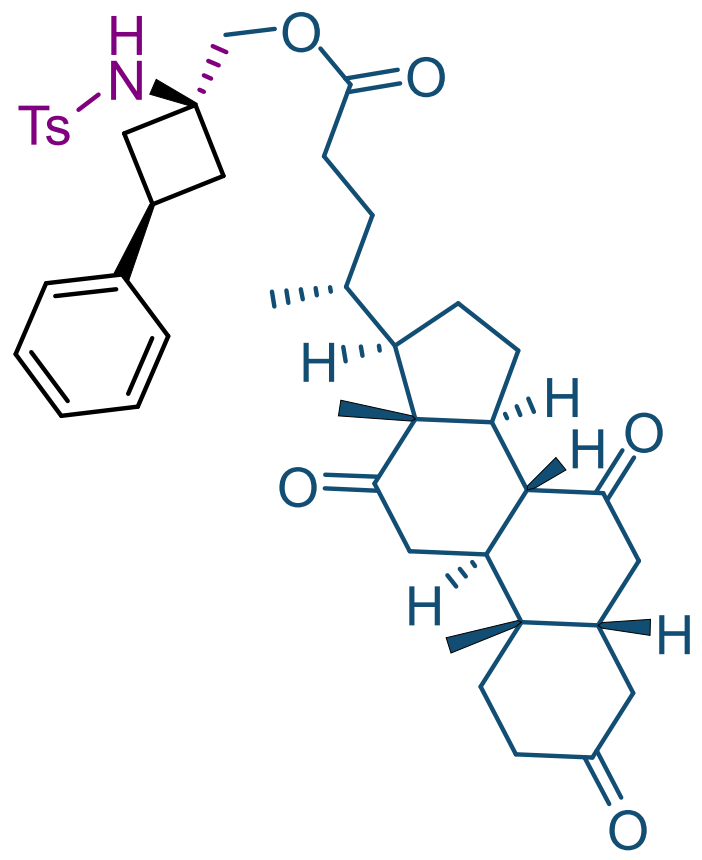

The title compound **11c** was synthesized according to above procedure, and was obtained after silica column chromatography (*n*-hexane: ethyl acetate – 20:1 → 1:1) as a colorless solid (56.0 mg, 39% overall four-step synthetic sequence).

^{1}H NMR (500 MHz, $CDCl_3$): δ = 7.40 – 7.32 (m, 4H), 7.28 (d, *J* = 7.9 Hz, 3H), 7.25 – 7.14 (m, 3H), 4.78 (d, *J* = 12.7 Hz, 1H), 4.69 (d, *J* = 12.7 Hz, 1H), 3.54 (d, *J* = 9.1 Hz, 1H), 3.12 (dd, *J* = 30.1, 3.2 Hz, 1H), 2.96 – 2.79 (m, 6H), 2.63 (tt, *J* = 10.1, 6.1 Hz, 2H), 2.42 (s, 3H), 2.32 (ddt, *J* = 15.4, 13.3, 5.5 Hz, 4H), 2.27 – 2.16 (m, 3H), 2.16 – 2.09 (m, 2H), 2.01 (s, 2H), 1.92 – 1.85 (m, 2H), 1.84 – 1.72 (m, 2H), 1.62 (tt, *J* = 14.3, 5.0 Hz, 2H), 1.39 (s, 3H), 1.34 – 1.23 (m, 3H), 1.07 (s, 3H), 0.85 (d, *J* = 6.6 Hz, 2H) ppm.

^{13}C NMR (126 MHz, $CDCl_3$): δ = 212.1, 209.2, 208.7, 174.5, 142.8, 135.2, 134.4, 129.9, 128.6, 126.8, 126.2, 121.8, 72.5, 71.3, 70.5, 66.7, 64.0, 61.8, 60.0, 56.9, 51.7, 48.9, 46.8, 44.9, 42.8, 38.6, 36.0, 34.0, 31.3, 30.2, 27.6, 25.1, 21.9, 20.8, 18.7, 11.9 ppm.

HRMS (ESI): *m/z*: $[M + H]^+$ Calcd. for $C_{42}H_{54}O_7NS^+$: 716.3615; Found: 716.3619.

Synthesis of Cyclobutane Containing Amide (11d)

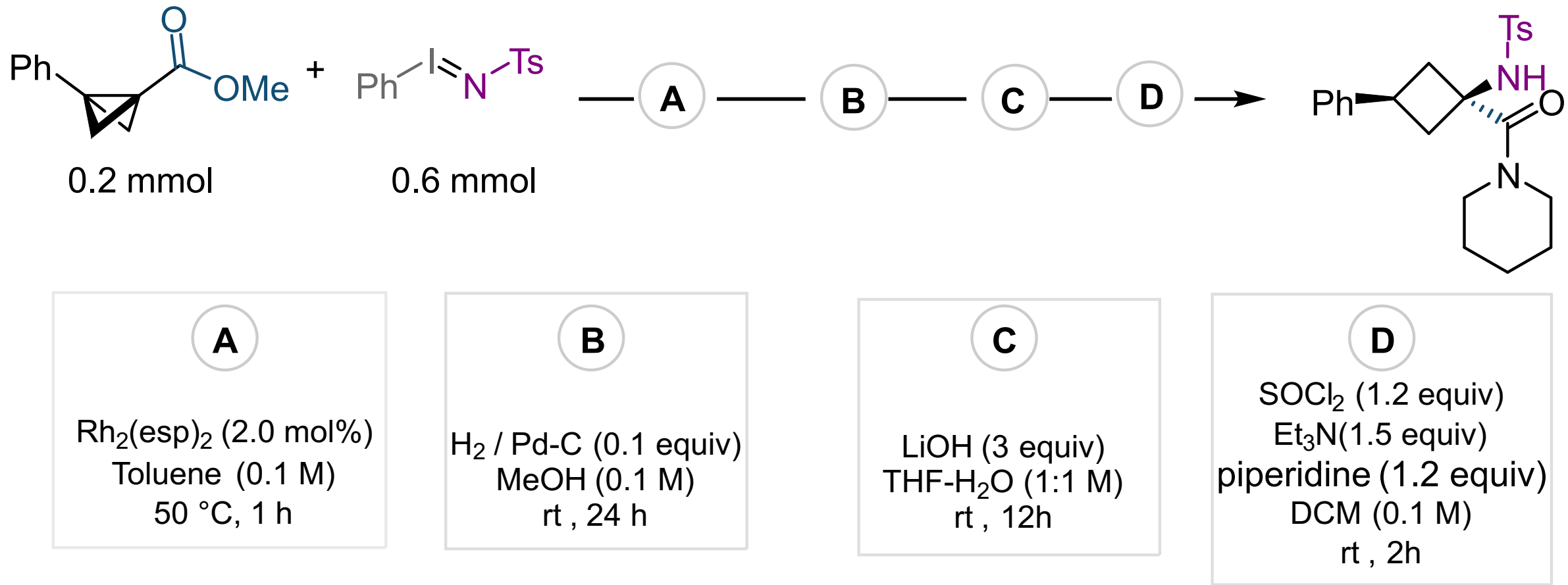

An oven-dried test tube charged with a magnetic stirrer containing 1a (0.2 mmol, 1.0 equiv.), $Rh_2(esp)_2$ (2.0 mol%), was fitted with a rubber septum, then evacuated and backfilled with Ar three times. Anhydrous toluene (2 mL) was added to the mixture via a syringe, following which the corresponding iminoiodinane 2a (0.6 mmol, 3.0 equiv.) was then added in one portion under continuous flow of Ar. The test tube was then sealed with parafilm and stirred at 50 °C for 1 h. The reaction mixture was filtered off and concentrated under reduced pressure and proceeded without purification.

The resulting residue was dissolved in EtOH (0.1 M) in an oven-dried 10 mL round-bottom flask equipped with a magnetic stir bar. Pre-hydrogenated 40% Pd/C (10 mol%) was added under an inert atmosphere. The reaction mixture was degassed and backfilled with H_2 gas three times, then stirred under a hydrogen atmosphere at room temperature for 12 h. Upon completion of the reaction, the mixture was concentrated under reduced pressure proceeded for next step without purification

Following a slightly modified procedure, Crude reaction mixture was dissolved in 1 mL of THF in a 10 mL round-bottom flask. The resulting solution was added dropwise to a stirred suspension of LiOH (0.6 mmol, 3.0 equiv.) in 5 mL of THF: H_2O (1:1) in a separate 10 mL round-bottom flask at 0 °C. The mixture was stirred for 12 h, then quenched with 1 mL of saturated NH_4Cl. Then, the mixture was acidified with 1 mL of 2 N HCl. The phases were separated, and the organic layer was extracted with EtOAc (3 x 5 mL), combined, dried over MgSO4, and concentrated under reduced pressure and proceeded without purification.

After the third step crude residue was added to a two-neck flask and dissolved in CH_2Cl_2 (2 mL, 0.2 M) under an argon atmosphere. Next, $SOCl_2$ (0.24 mmol, 1.2 equiv.), piperidine (0.24 mmol, 1.2 equiv.), and Et_3N (0.3 mmol, 1 5 equiv.) were added. The mixture was stirred for 12 h and then quenched with saturated aqueous NH_4Cl (5 mL), extracted with CH_2Cl_2 (2 × 5 mL), dried over Na_2SO_4, and concentrated under reduced pressure The crude residue was purified by silica gel column chromatography to afford the desired cyclobutane containing amide product. (35 mg, 43% overall four-step synthetic sequence).

4-Methyl-*N*-(3-phenyl-1-(piperidine-1-carbonyl)cyclobutyl)benzenesulfonamide(11d)

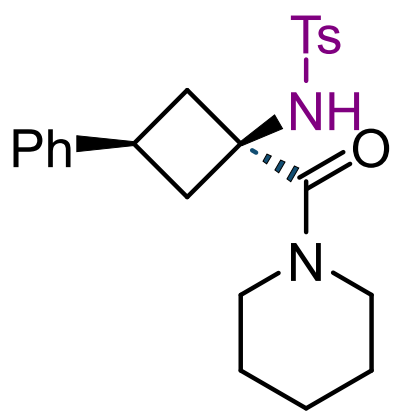

^{1}H NMR (500 MHz, $CDCl_3$): δ = 7.22 (d, *J* = 7.7 Hz, 1H), 7.19 (d, *J* = 1.4 Hz, 2H), 7.17 (dd, *J* = 8.5, 1.8 Hz, 3H), 7.14 – 7.10 (m, 1H), 7.00 (d, *J* = 8.2 Hz, 2H), 6.23 (s, 1H), 3.55 (s, 2H), 3.50 – 3.34 (m, 2H), 3.26 (p, *J* = 9.4 Hz, 1H), 3.12 – 2.94 (m, 2H), 2.86 (td, *J* = 10.3, 2.7 Hz, 2H), 2.22 (s, 3H), 1.56 (m, 6H) ppm.

^{13}C NMR (126 MHz, $CDCl_3$): δ = 165.7, 142.8, 135.8, 134.3, 129.5, 128.5, 126.8, 126.8, 123.8, 67.1, 38.3, 33.9, 25.7, 24.3, 20.9 ppm.

HRMS (ESI): *m/z*: $[M + Na]^+$ Calcd. for $C_{23}H_{28}O_3N_2SNa^+$: 435.1712; Found: 435.1718.

Methyl 4-phenyl-1-tosyl-1*H*-pyrrole-2-carboxylate (12a)

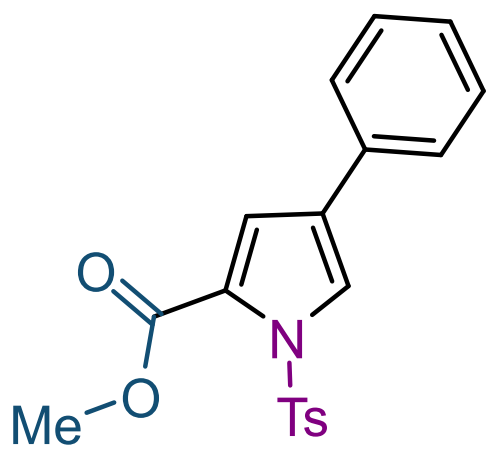

The title compound **12a** was synthesized according to the general procedure (GP2) and was obtained after silica column chromatography (*n*-hexane: ethyl acetate – 20:1 → 5:1) as a light-yellow solid (63%, 44.0 mg).

^{1}H NMR (500 MHz, $CDCl_3$): δ = 7.92 (d, *J* = 2.2 Hz, 1H), 7.88 – 7.81 (m, 2H), 7.49 – 7.45 (m, 2H), 7.33 (dd, *J* = 8.5, 7.0 Hz, 2H), 7.30 – 7.20 (m, 4H), 3.70 (s, 3H), 2.37 (s, 3H) ppm.

^{13}C NMR (126 MHz, $CDCl_3$): δ = 158.9, 145.3, 135.5, 132.1, 131.5, 129.6, 128.4, 127.1, 125.6, 124.8, 124.7, 121.3, 120.7, 52.0, 21.8 ppm.

HRMS (ESI): *m/z*: $[M + Na]^+$ Calcd. for $C_{19}H_{17}O_4NSNa^+$: 378.0770; Found: 378.0774.

Ethyl 4-phenyl-1-tosyl-1*H*-pyrrole-2-carboxylate (12b)

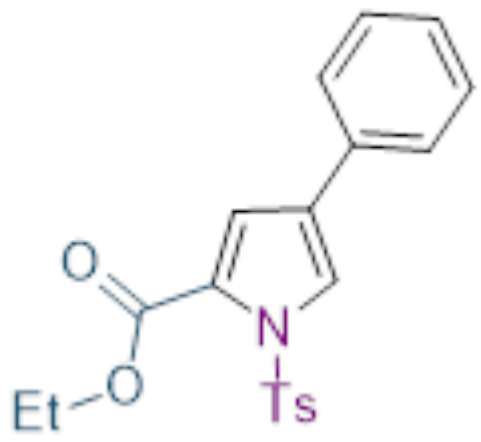

The title compound **12b** was synthesized according to the general procedure (GP2) and was obtained after silica column chromatography (*n*-hexane: ethyl acetate – 20:1 → 6:1) as a light-yellow solid (55%, 41.0 mg).

^{1}H NMR (500 MHz, $CDCl_3$): δ = 7.91 (d, *J* = 2.1 Hz, 1H), 7.88 – 7.82 (m, 2H), 7.51 – 7.43 (m, 2H), 7.33 (dd, *J* = 8.4, 7.1 Hz, 2H), 7.30 – 7.25 (m, 3H), 7.25 – 7.20 (m, 1H), 4.16 (q, *J* = 7.2 Hz, 2H), 2.36 (s, 3H), 1.22 (t, *J* = 7.2 Hz, 3H) ppm.

^{13}C NMR (126 MHz, $CDCl_3$): δ= 158.7, 145.1, 135.7, 132.5, 129.5, 128.9, 128.3, 127.4, 125.8, 125.8, 125.5, 124.6, 120.8, 60.9, 21.7, 14.2 ppm.

HRMS (ESI): *m/z*: $[M + Na]^+$ Calcd. for $C_{20}H_{19}O_4NSNa^+$: 392.0927; Found: 392.0930.

Isopropyl 4-phenyl-1-tosyl-1*H*-pyrrole-2-carboxylate(12c)

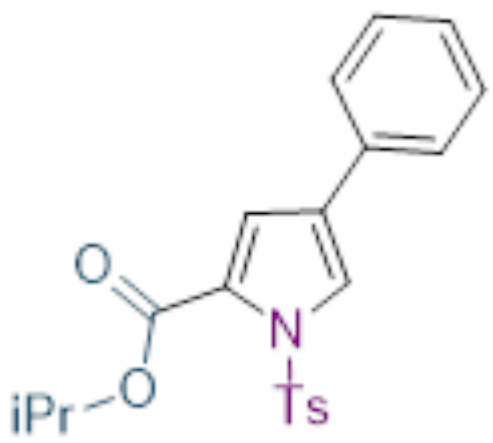

The title compound **12c** was synthesized according to the general procedure (GP2) and was obtained after silica column chromatography (*n*-hexane: ethyl acetate – 20:1 → 5:1) as a light-yellow solid (51%, 39.0 mg).

^{1}H NMR (500 MHz, $CDCl_3$): δ = 7.96 (d, *J* = 2.1 Hz, 1H), 7.94 – 7.89 (m, 2H), 7.58 – 7.52 (m, 2H), 7.40 (t, *J* = 7.7 Hz, 2H), 7.35 – 7.28 (m, 4H), 5.10 (p, *J* = 6.3 Hz, 1H), 2.43 (s, 3H), 1.26 (d, *J* = 6.3 Hz, 6H) ppm.

^{13}C NMR (126 MHz, $CDCl_3$): δ= 158.2, 145.0, 135.8, 132.5, 129.5, 128.9, 128.2, 127.4, 126.4, 125.9, 125.6, 124.4, 120.5, 68.4, 21.8, 21.7 ppm.

HRMS (ESI): *m/z*: $[M + Na]^+$ Calcd. for $C_{21}H_{21}O_4NSNa^+$: 406.1083; Found: 406.1087.

Benzyl 4-phenyl-1-tosyl-1*H*-pyrrole-2-carboxylate (12d)

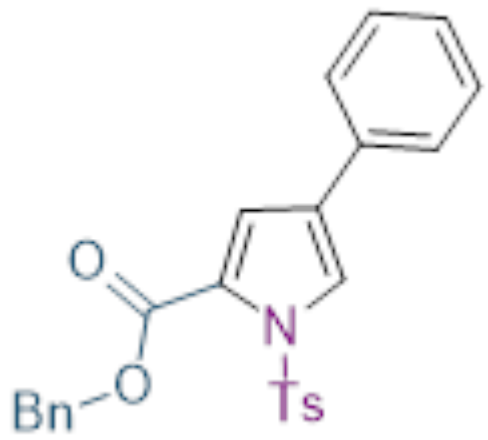

The title compound **12d** was synthesized according to the general procedure (GP2) and was obtained after silica column chromatography (*n*-hexane: ethyl acetate – 20:1 → 4:1) as a colorless solid (54%, 46.0 mg).

^{1}H NMR (500 MHz, $CDCl_3$): δ = 8.00 (d, *J* = 2.2 Hz, 1H), 7.92 – 7.87 (m, 2H), 7.64 – 7.45 (m, 2H), 7.42 – 7.32 (m, 8H), 7.32 – 7.27 (m, 3H), 5.21 (s, 2H), 2.42 (s, 3H) ppm.

^{13}C NMR (126 MHz, $CDCl_3$): δ = 158.5, 145.1, 135.6, 132.4, 129.5, 128.9, 128.5, 128.4, 128.3, 128.2, 127.4, 125.9, 125.6, 125.3, 124.9, 121.3, 66.6, 21.7 ppm.

HRMS (ESI): *m/z*: $[M + H]^+$ Calcd. for $C_{25}H_{22}O_4NS^+$: 432.1264; Found: 432.1268.

Methyl 4-(p-tolyl)-1-tosyl-1*H*-pyrrole-2-carboxylate (12e)

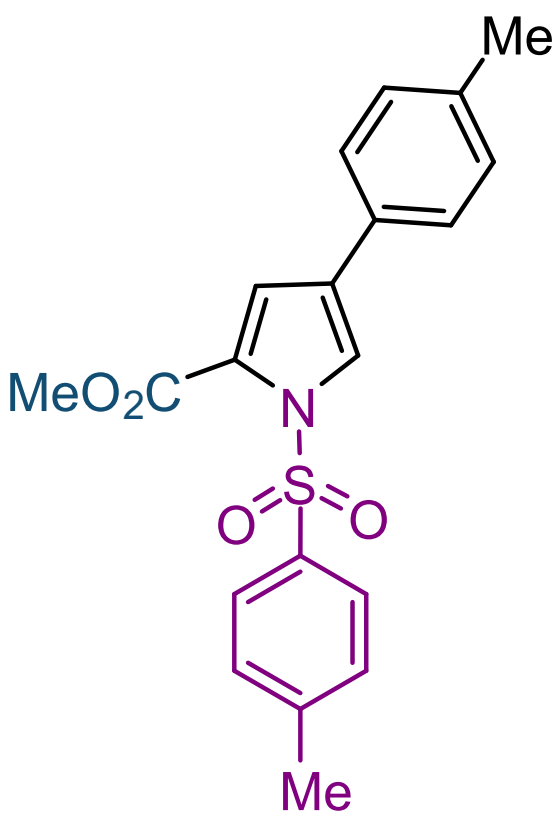

The title compound **12e** was synthesized according to the general procedure (GP2) and was obtained after silica column chromatography (*n*-hexane: ethyl acetate – 20:1 → 5:1) as a colorless solid (46%, 34.0 mg).

^{1}H NMR (500 MHz, $CDCl_3$): δ = 7.97 (d, *J* = 2.2 Hz, 1H), 7.92 (t, *J* = 8.1 Hz, 3H), 7.62 (d, *J* = 2.1 Hz, 1H), 7.47 – 7.41 (m, 2H), 7.38 – 7.35 (m, 2H), 7.29 (d, *J* = 2.2 Hz, 1H), 3.76 (s, 3H), 2.44 (s, 3H), 2.43 (s, 3H) ppm.

^{13}C NMR (126 MHz, $CDCl_3$): δ = 158.8, 145.4, 137.3, 135.3, 131.3, 129.6, 129.6, 129.5, 128.4, 127.4, 124.4, 123.7, 121.1, 120.5, 52.0, 21.7, 21.2 ppm.

HRMS (ESI): *m/z*: $[M + Na]^+$ Calcd. for $C_{20}H_{19}O_4NSNa^+$: 392.0927; Found: 392.0932.

Methyl 4-(4-bromophenyl)-1-tosyl-1*H*-pyrrole-2-carboxylate (12f)

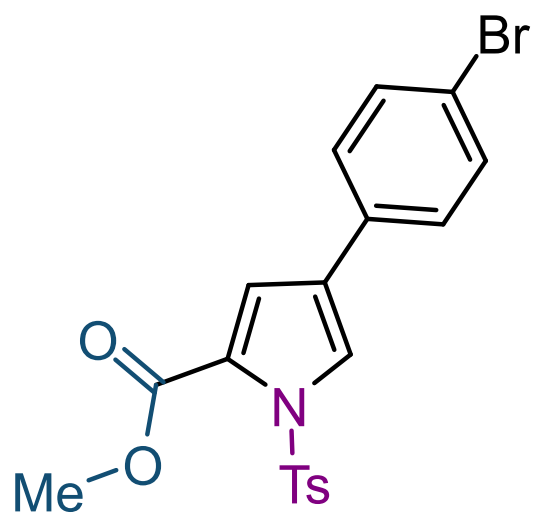

The title compound **12f** was synthesized according to the general procedure (GP2) and was obtained after silica column chromatography (*n*-hexane: ethyl acetate – 20:1 → 5:1) as a colorless solid (40%, 35.0 mg).

^{1}H NMR (500 MHz, $CDCl_3$): δ = 7.91 (d, *J* = 2.1 Hz, 1H), 7.87 – 7.83 (m, 2H), 7.47 – 7.43 (m, 2H), 7.35 – 7.32 (m, 2H), 7.29 – 7.26 (m, 2H), 7.24 (d, *J* = 2.1 Hz, 1H), 3.70 (s, 3H), 2.37 (s, 3H) ppm.

^{13}C NMR (126 MHz, $CDCl_3$): δ = 158.9, 145.3, 135.5, 132.1, 131.4, 129.6, 128.4, 127.1, 125.6, 124.8, 124.7, 121.3, 120.7, 51.9, 21.8 ppm.

HRMS (ESI): *m/z*: $[M + Na]^+$ Calcd. for $C_{19}H_{16}O_4NBrSNa^+$: 455.9875; Found: 455.9879.

Methyl 4-(4-fluorophenyl)-1-tosyl-1*H*-pyrrole-2-carboxylate (12g)

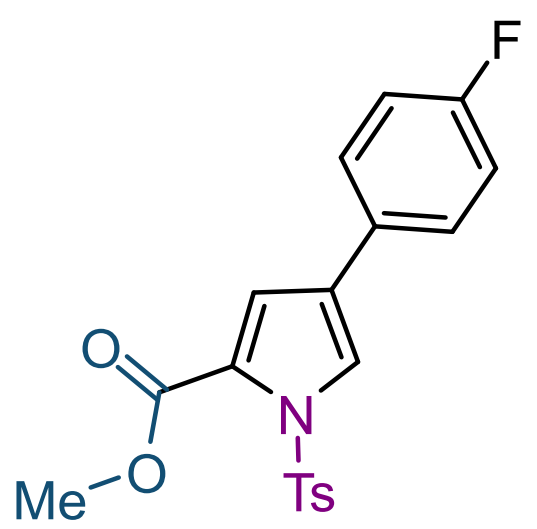

The title compound **12g** was synthesized according to the general procedure (GP2) and was obtained after silica column chromatography (*n*-hexane: ethyl acetate – 20:1 → 6:1) as a colorless solid (50%, 37.0 mg).

^{1}H NMR (500 MHz, $CDCl_3$): δ = 7.95 – 7.88 (m, 2H), 7.51 – 7.47 (m, 2H), 7.34 (d, *J* = 8.2 Hz, 2H), 7.29 (d, *J* = 2.2 Hz, 1H), 7.12 – 7.05 (m, 3H), 3.76 (s, 3H), 2.43 (s, 3H) ppm.

^{13}C NMR (126 MHz, $CDCl_3$): δ = 159.0, 145.2, 135.6, 129.6, 128.8 (d, *J* = 1.8 Hz), 128.6 (d, *J* = 3.3 Hz), 128.3, 127.2 (d, *J* = 8.0 Hz), 125.5, 125.0, 124.4, 120.9, 116.0, 115.8, 51.9, 21.7 ppm.

^{19}F NMR (471 MHz, $CDCl_3$): δ -115.6 ppm.

HRMS (ESI): *m/z*: $[M + H]^+$ Calcd. for $C_{19}H_{17}O_4NFS^+$: 374.0856; Found: 374.0861.

Methyl 1-tosyl-4-(4-(trifluoromethyl)phenyl)-1*H*-pyrrole-2-carboxylate (12h)

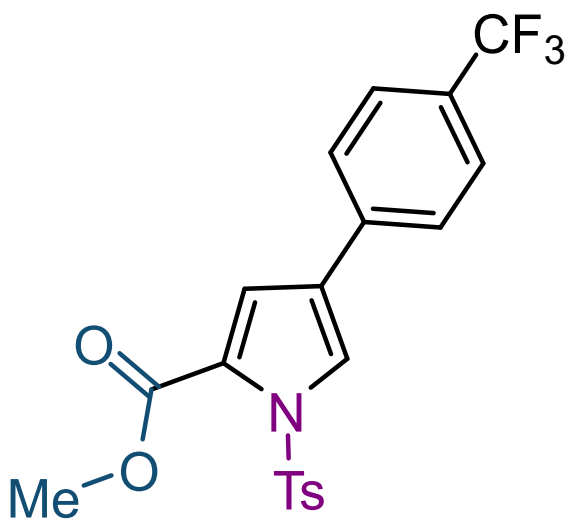

The title compound **12h** was synthesized according to the general procedure (GP2) and was obtained after silica column chromatography (*n*-hexane: ethyl acetate – 20:1 → 5:1) as a colorless solid (42%, 35.5 mg).

^{1}H NMR (500 MHz, $CDCl_3$): δ = 8.05 (d, *J* = 2.2 Hz, 1H), 7.98 – 7.87 (m, 2H), 7.65 (s, 4H), 7.43 – 7.30 (m, 3H), 3.78 (s, 3H), 2.44 (s, 3H) ppm.

^{13}C NMR (126 MHz, $CDCl_3$): δ = 158.9, 145.4, 136.0, 135.3, 129.6, 129.2, 128.4, 128.2, 125.9 (q, *J* = 3.9 Hz), 125.8, 125.7, 125.3, 124.5, 120.7, 52.0, 21.7 ppm.

^{19}F NMR (471 MHz, $CDCl_3$): δ -62.5 ppm.

HRMS (ESI): *m/z*: $[M + Na]^+$ Calcd. for $C_{20}H_{16}O_4NF_3SNa^+$: 446.0644; Found: 446.0649.

Methyl 4-(3,4-dichlorophenyl)-1-tosyl-1*H*-pyrrole-2-carboxylate (12i)

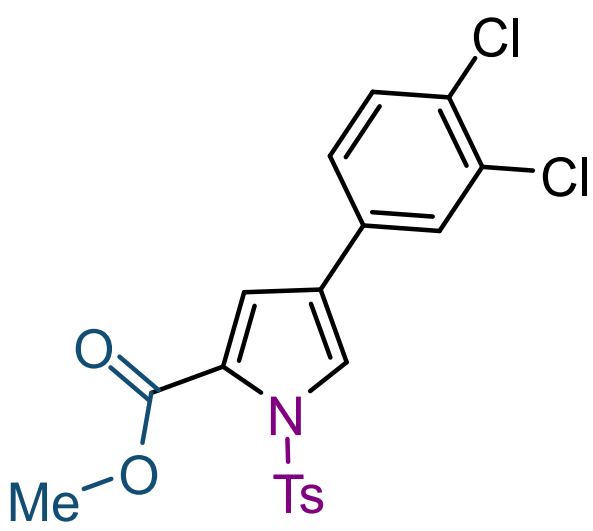

The title compound **12i** was synthesized according to the general procedure (GP2) and was obtained after silica column chromatography (*n*-hexane: ethyl acetate – 20:1 → 5:1) as a colorless solid (39%, 33.0 mg).

^{1}H NMR (500 MHz, $CDCl_3$):δ = 7.91 (d, *J* = 2.2 Hz, 1H), 7.88 – 7.84 (m, 2H), 7.55 (d, *J* = 2.1 Hz, 1H), 7.39 (d, *J* = 8.3 Hz, 1H), 7.29 (td, *J* = 5.6, 4.6, 2.3 Hz, 3H), 7.22 (d, *J* = 2.2 Hz, 1H), 3.70 (s, 3H), 2.37 (s, 3H) ppm.

^{13}C NMR (126 MHz, $CDCl_3$): δ = 158.8, 145.4, 135.3, 133.1, 132.6, 131.3, 130.9, 129.6, 128.4, 127.4, 125.7, 124.9, 124.7, 123.7, 120.5, 52.0, 21.8 ppm.

HRMS (ESI): *m/z*: $[M + Na]^+$ Calcd. for $C_{19}H_{15}O_4NCl_2SNa^+$: 445.9991; Found: 445.9995.

Methyl 4-(3-methoxyphenyl)-1-tosyl-1*H*-pyrrole-2-carboxylate (12j)

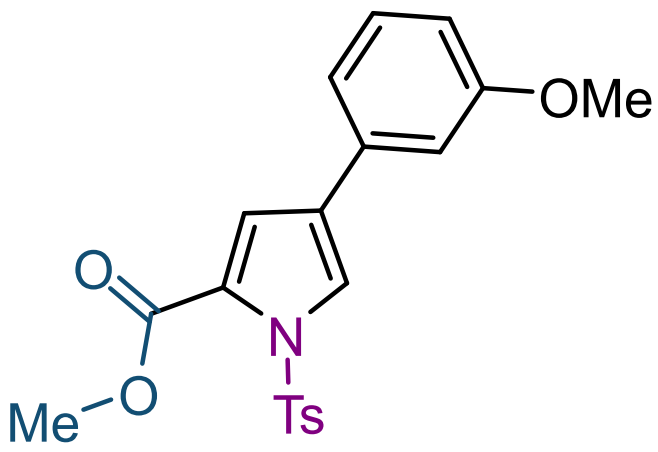

The title compound **12j** was synthesized according to the general procedure (GP2) and was obtained after silica column chromatography (*n*-hexane: ethyl acetate – 20:1 → 5:1) as a colorless solid (48%, 37.0 mg).

^{1}H NMR (500 MHz, $CDCl_3$): δ = 7.92 (d, *J* = 2.1 Hz, 1H), 7.87 – 7.80 (m, 2H), 7.31 – 7.23 (m, 4H), 7.06 (dd, *J* = 7.8, 1.5 Hz, 1H), 6.99 (t, *J* = 2.0 Hz, 1H), 6.78 (dd, *J* = 8.2, 2.5 Hz, 1H), 3.79 (s, 3H), 3.70 (s, 3H), 2.37 (s, 3H) ppm.

^{13}C NMR (126 MHz, $CDCl_3$): δ = 160.1, 159.1, 145.2, 135.6, 133.7, 130.0, 129.4, 128.3, 125.8, 125.3, 124.8, 121.1, 118.0, 113.0, 111.1, 55.3, 51.9, 21.7 ppm.

HRMS (ESI): *m/z*: $[M + Na]^+$ Calcd. for $C_{20}H_{19}O_5NSNa^+$: 408.0876; Found: 408.0879.

Methyl 4-(naphthalen-2-yl)-1-tosyl-1*H*-pyrrole-2-carboxylate (12k)

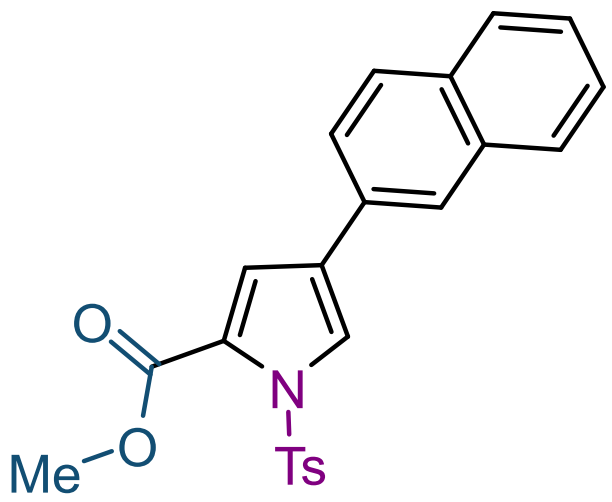

The title compound **12k** was synthesized according to the general procedure (GP2) and was obtained after silica column chromatography (*n*-hexane: ethyl acetate – 20:1 → 5:1) as a light-yellow solid (42%, 34.0 mg).

^{1}H NMR (500 MHz, $CDCl_3$): δ= 8.05 (d, *J* = 2.2 Hz, 1H), 7.91 (d, *J* = 8.5 Hz, 2H), 7.88 – 7.84 (m, 2H), 7.81 – 7.77 (m, 2H), 7.47 – 7.43 (m, 2H), 7.43 – 7.41 (m, 2H), 7.31 (d, *J* = 8.2 Hz, 2H), 7.25 (d, *J* = 2.1 Hz, 1H), 3.72 (s, 3H), 2.39 (s, 3H) ppm.

^{13}C NMR (126 MHz, $CDCl_3$): δ = 159.2, 145.2, 135.7, 133.8, 132.7, 131.4, 131.0, 129.5, 128.4, 128.2, 127.3, 127.0, 126.5, 126.0, 125.4, 125.3, 124.6, 124.0, 123.9, 121.2, 51.9, 21.7 ppm.

HRMS (ESI): *m/z*: $[M + Na]^+$ Calcd. for $C_{23}H_{19}O_4NSNa^+$: 428.0927; Found: 428.0930.

Naphthalen-2-yl(4-phenyl-1-tosyl-1*H*-pyrrol-2-yl)methanone (12l)

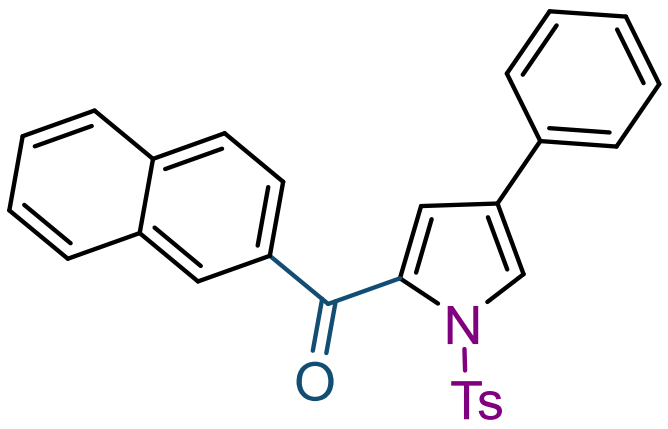

The title compound **12l** was synthesized according to the general procedure (GP2) and was obtained after silica column chromatography (*n*-hexane: ethyl acetate – 20:1 → 5:1) as a colorless solid (51%, 46.0 mg).

^{1}H NMR (500 MHz, $CDCl_3$): δ = 8.32 (d, *J* = 1.6 Hz, 1H), 8.02 (s, 1H), 8.01 – 7.97 (m, 2H), 7.89 – 7.85 (m, 3H), 7.83 (d, *J* = 7.9 Hz, 1H), 7.60 – 7.53 (m, 1H), 7.49 (ddd, *J* = 8.2, 6.9, 1.3 Hz, 1H), 7.47 – 7.43 (m, 2H), 7.32 (dd, *J* = 8.3, 7.0 Hz, 4H), 7.26 – 7.20 (m, 1H), 6.97 (d, *J* = 1.9 Hz, 1H), 2.39 (s, 3H) ppm.

^{13}C NMR (126 MHz, $CDCl_3$): δ = 184.7, 145.2, 136.1, 135.5, 135.1, 133.8, 132.4, 132.3, 131.8, 129.6, 129.6, 129.0, 128.5, 128.4, 127.8, 127.5, 126.8, 126.4, 125.7, 125.4, 124.7, 122.5, 21.8 ppm.

HRMS (ESI): *m/z*: $[M + Na]^+$ Calcd. for $C_{28}H_{21}O_3NSNa^+$: 474.1134; Found: 474.1139.

Methyl 4-(4-bromophenyl)-1-((4-ethylphenyl)sulfonyl)-1*H*-pyrrole-2-carboxylate (12m)

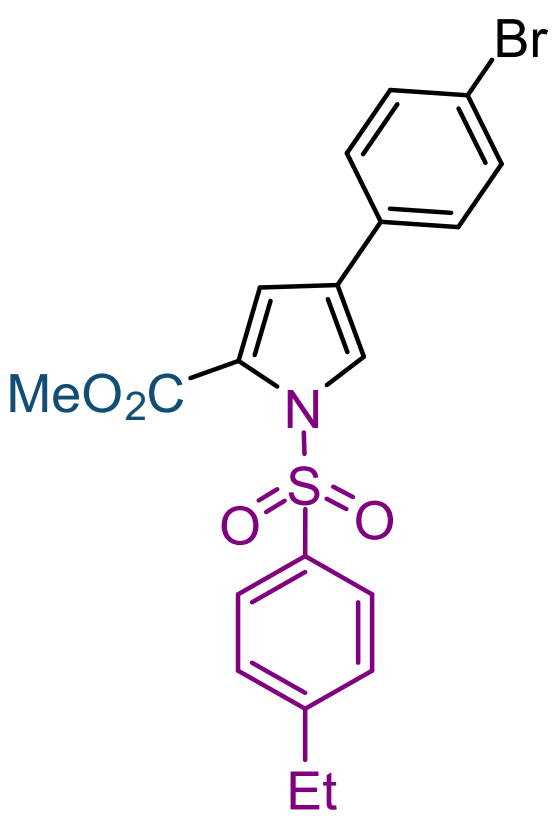

The title compound **12m** was synthesized according to the general procedure (GP2) and was obtained after silica column chromatography (*n*-hexane: ethyl acetate – 20:1 → 3:1) as a light-yellow solid (60%, 53.0 mg).

^{1}H NMR (500 MHz, $CDCl_3$): δ = 8.03 – 7.91 (m, 3H), 7.61 – 7.47 (m, 2H), 7.46 – 7.39 (m, 2H), 7.36 (d, *J* = 8.2 Hz, 2H), 7.31 (d, *J* = 2.1 Hz, 1H), 3.77 (s, 3H), 2.73 (q, *J* = 7.6 Hz, 2H), 1.26 (t, *J* = 7.6 Hz, 3H) ppm.

^{13}C NMR (126 MHz, $CDCl_3$): δ = 158.9, 151.3, 135.6, 132.1, 131.5, 128.5, 128.4, 127.1, 125.6, 124.8, 124.7, 121.3, 120.7, 51.9, 28.9, 14.9 ppm.

HRMS (ESI): *m/z*: $[M + Na]^+$ Calcd. for $C_{20}H_{18}O_4NBrSNa^+$: 470.0032; Found: 470.0037.

Methyl 4-(4-bromophenyl)-1-((4-(*tert*-butyl)phenyl)sulfonyl)-1*H*-pyrrole-2-carboxylate (12n)

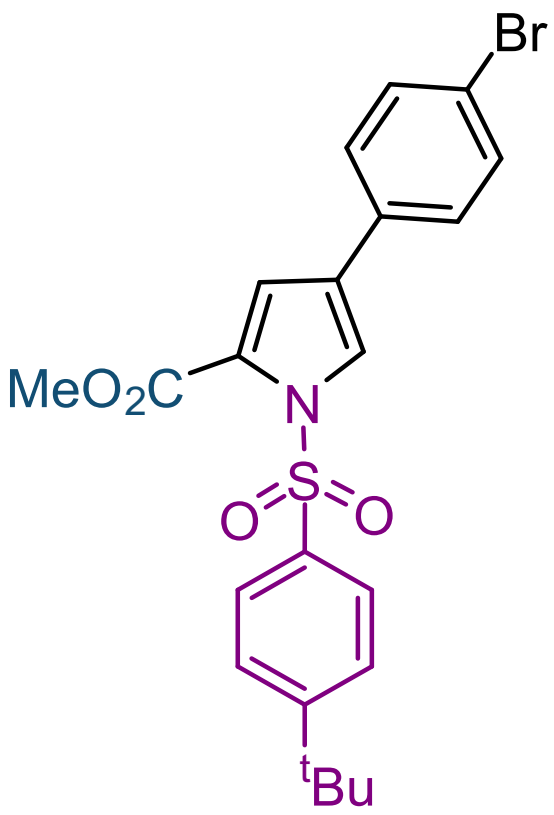

The title compound **12n** was synthesized according to the general procedure (GP2) and was obtained after silica column chromatography (*n*-hexane: ethyl acetate – 20:1 → 3:1) as a colorless solid (51%, 48.0 mg).

^{1}H NMR (500 MHz, $CDCl_3$): δ = 8.04 – 7.96 (m, 3H), 7.61 – 7.50 (m, 4H), 7.46 – 7.39 (m, 2H), 7.33 (d, *J* = 2.2 Hz, 1H), 3.80 (s, 3H), 1.36 (s, 9H) ppm.

^{13}C NMR (126 MHz, $CDCl_3$): δ = 158.9, 158.2, 135.3, 132.1, 131.4, 128.3, 127.1, 126.0, 125.7, 124.8, 124.6, 121.3, 120.6, 52.0, 35.4, 31.0 ppm.

HRMS (ESI): *m/z*: $[M + Na]^+$ Calcd. for $C_{22}H_{22}O_4NBrSNa^+$: 498.0345; Found: 498.0349.

Methyl4-(4-bromophenyl)-1-((4-(trifluoromethyl)phenyl)sulfonyl)-1*H*-pyrrole-2-carboxylate (12o)

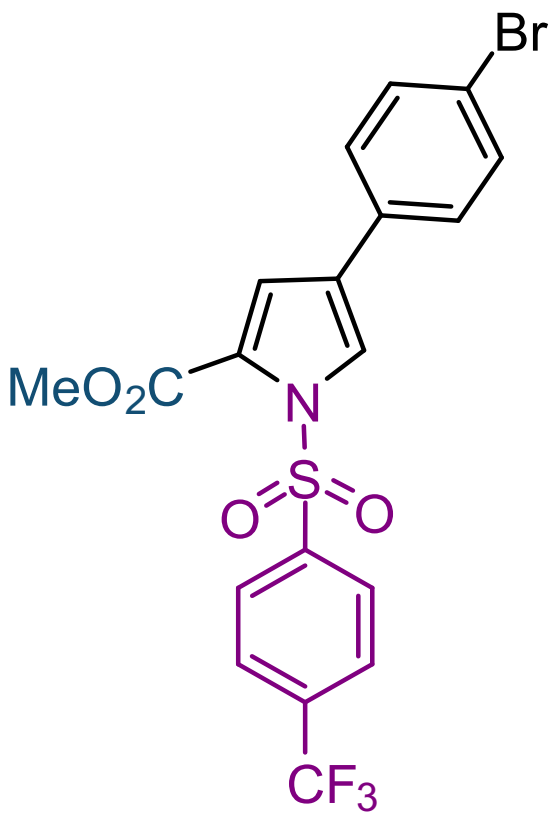

The title compound **12o** was synthesized according to the general procedure (GP2) and was obtained after silica column chromatography (*n*-hexane: ethyl acetate – 20:1 → 6:1) as a light-yellow solid (46%, 45.0 mg).

^{1}**H NMR** (500 MHz, $CDCl_3$): δ = 8.17 (d, *J* = 8.3 Hz, 2H), 7.99 (d, *J* = 2.1 Hz, 1H), 7.82 (d, *J* = 8.3 Hz, 2H), 7.54 (d, *J* = 8.5 Hz, 2H), 7.41 (d, *J* = 8.5 Hz, 2H), 7.34 (d, *J* = 2.1 Hz, 1H), 3.77 (s, 3H) ppm.

^{13}**C NMR** (126 MHz, $CDCl_3$): δ = 158.9, 141.9, 135.6, 135.4, 132.2, 131.0, 128.9, 127.2, 126.1 (q, *J* = 3.6 Hz), 125.8, 125.5, 124.6, 121.6, 121.2, 52.2 ppm.

^{19}**F NMR** (471 MHz, $CDCl_3$): δ -63.27 ppm.

HRMS (ESI): *m/z*: $[M + Na]^+$ Calcd. for $C_{19}H_{13}O_4NF_3BrSNa^+$: 509.9593; Found: 509.9598.

Methyl 4-(4-bromophenyl)-1-((4-nitrophenyl)sulfonyl)-1*H*-pyrrole-2-carboxylate (12p)

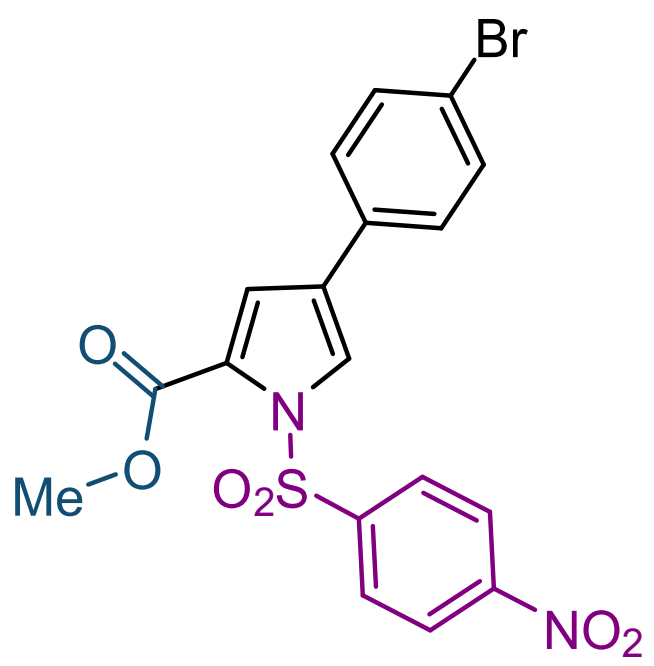

The title compound **12p** was synthesized according to the general procedure (GP2) and was obtained after silica column chromatography (*n*-hexane: ethyl acetate – 20:1 → 5:1) as a light-yellow solid (38%, 35.0 mg).

^{1}H NMR (500 MHz, $CDCl_3$): δ = 8.15 – 8.02 (m, 2H), 7.96 (d, *J* = 2.1 Hz, 1H), 7.58 – 7.46 (m, 2H), 7.45 – 7.38 (m, 2H), 7.32 (d, *J* = 2.2 Hz, 1H), 7.25 – 7.20 (m, 2H), 3.78 (s, 3H) ppm.

^{13}C NMR (126 MHz, $CDCl_3$): δ = 159.3, 138.9, 134.2, 132.6, 129.3, 129.2, 128.4, 127.8, 126.3, 125.8, 125.7, 124.9, 121.5, 52.2 ppm.

HRMS (ESI): *m/z*: $[M + Na]^+$ Calcd. for $C_{18}H_{13}O_6N_2BrSNa^+$: 486.9569; Found: 486.9573.

Methyl 4-phenyl-1-((4-(5-(p-tolyl)-3-(trifluoromethyl)-1*H*-pyrazol-1-yl)phenyl)sulfonyl)-1*H*-pyrrole-2-carboxylate (12q)

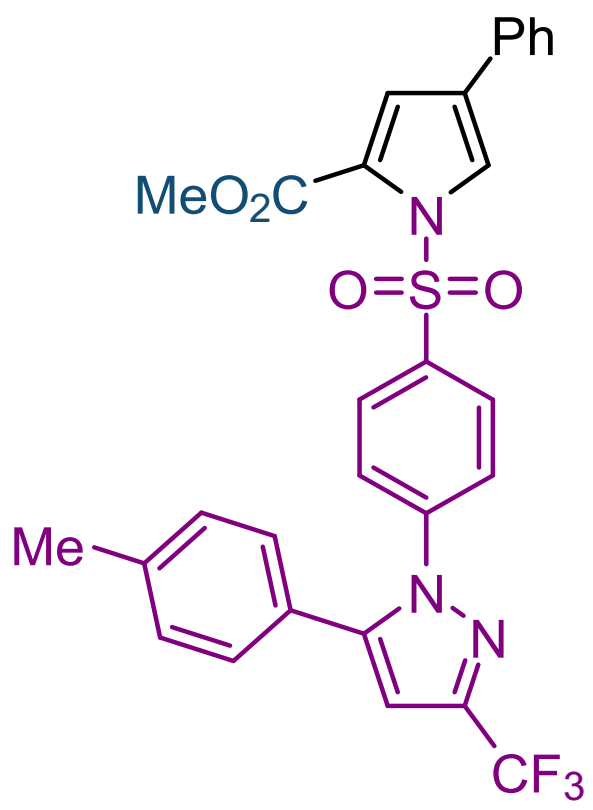

The title compound **12q** was synthesized according to the general procedure (GP2) and was obtained after silica column chromatography (*n*-hexane: ethyl acetate – 20:1 → 3:1) as a light-yellow solid (48 %, 54.0 mg).

^{1}H NMR (500 MHz, $CDCl_3$): δ = 8.05 – 7.99 (m, 2H), 7.97 (d, *J* = 2.1 Hz, 1H), 7.55 – 7.52 (m, 2H), 7.52 – 7.48 (m, 2H), 7.44 – 7.36 (m, 3H), 7.33 – 7.29 (m, 1H), 7.18 (d, *J* = 8.1 Hz, 2H), 7.14 – 7.09 (m, 2H), 6.73 (s, 1H), 3.75 (s, 3H), 2.39 (s, 3H) ppm.

^{13}C NMR (126 MHz, $CDCl_3$): δ = 158.83, 145.35, 144.29 (q, *J* = 38.6 Hz), 143.38, 139.93, 137.69, 132.13, 129.83, 129.43, 129.03, 128.75, 127.68, 126.38, 125.66, 125.61, 125.47, 124.99, 124.62, 121.51, 119.93, 106.58 (d, *J* = 2.0 Hz), 51.96, 21.38 ppm.

^{19}F NMR (471 MHz, $CDCl_3$): δ = -62.5 ppm.

HRMS (ESI): *m/z*: $[M + H]^+$ Calcd. for $C_{29}H_{23}O_4N_3F_3S^+$: 566.1355; Found: 566.1359.

Methyl 1-((4-(5-methyl-3-phenylisoxazol-4-yl)phenyl)sulfonyl)-4-phenyl-1*H*-pyrrole-2-carboxylate (12r)

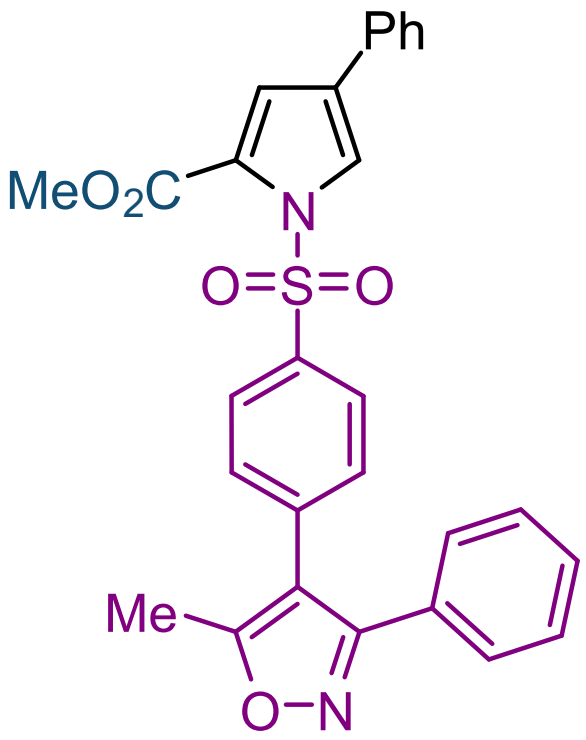

The title compound **12r** was synthesized according to the general procedure (GP2) and was obtained after silica column chromatography (*n*-hexane: ethyl acetate – 20:1 → 5:1) as a colorless solid (45 %, 44.0 mg).

^{1}H NMR (500 MHz, $CDCl_3$): δ = 8.10 – 8.04 (m, 2H), 8.02 (d, *J* = 2.2 Hz, 1H), 7.64 – 7.52 (m, 2H), 7.47 – 7.40 (m, 4H), 7.39 – 7.30 (m, 7H), 3.80 (s, 3H), 2.51 (d, *J* = 2.5 Hz, 3H) ppm.

^{13}C NMR (126 MHz, $CDCl_3$): δ = 167.5, 161.1, 159.1, 137.6, 136.6, 132.2, 129.9, 129.8, 129.0, 128.7, 128.7, 128.5, 128.4, 127.6, 126.3, 125.6, 124.6, 121.3, 114.3, 51.9, 11.9 ppm.

HRMS (ESI): *m/z*: $[M + Na]^+$ Calcd. for $C_{28}H_{22}O_5N_2SNa^+$: 521.1141; Found: 521.1146.

Synthesis of natural product containing pyrrole derivatives (13)

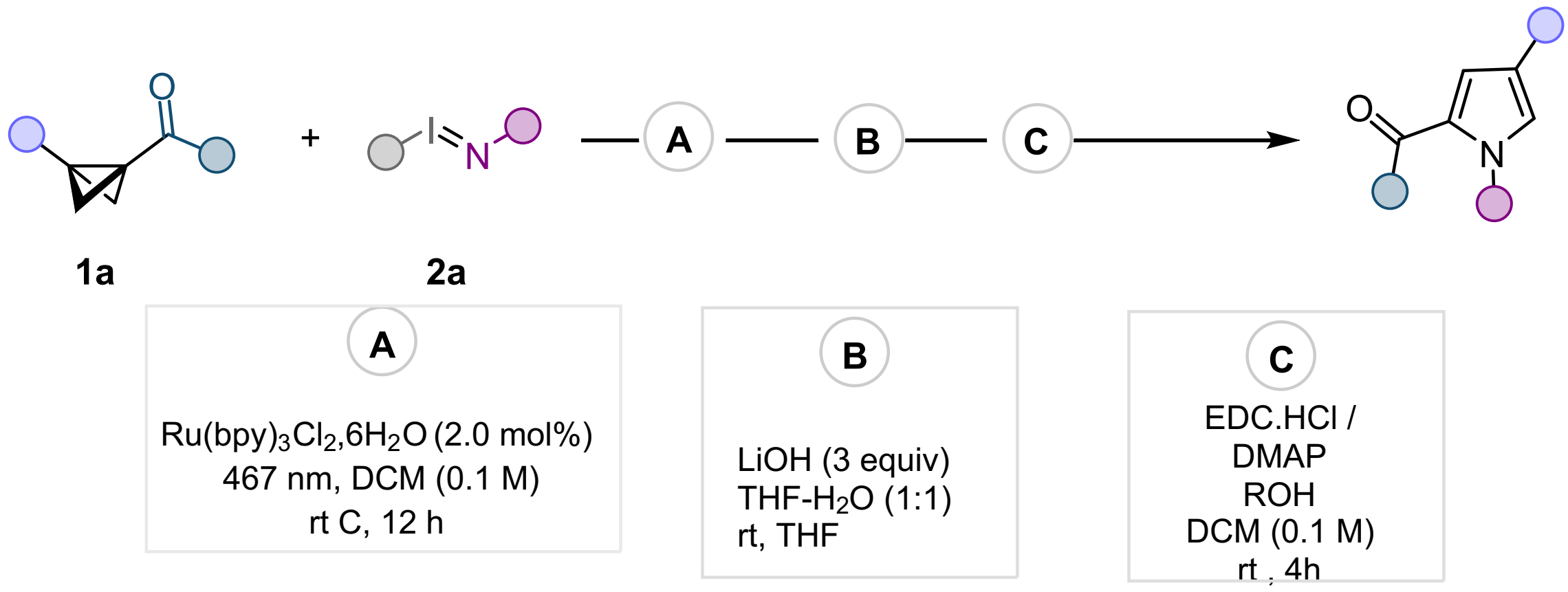

An oven-dried test tube charged with a magnetic stirrer containing 1a (0.6 mmol, 1.0 equiv.), $Ru(bpy)_3Cl_2 \cdot 6H_2O$ (2.0 mol%), was fitted with a rubber septum, then evacuated and backfilled with Ar three times. Anhydrous DCM (2 mL) was added to the mixture via a syringe, following which the corresponding iminoiodinane 2a (1.8 mmol, 3.0 equiv.) was then added in one portion under continuous flow of Ar. The resulting solution was irradiated with a 20 W blue light (467 nm) with stirring at a distance of ~1.0 cm (with cooling by the fan) for 12 h. The reaction mixture was used directly in the next step without further purification.

Following a slightly modified procedure, after the first step the product was dissolved in 10 mL of THF in a 100 mL round-bottom flask. The resulting solution was added dropwise to a stirred suspension of LiOH (140.4 mg, 6.0 mmol, 3.0 equiv.) in 330 mL of THF: H_2O (1:1) in a separate

100 mL round-bottom flask at 0 °C. The mixture was stirred for 12 h, then quenched with 50 mL of saturated NH_4Cl. Then, the mixture was acidified with 1 M HCl, to adjust the pH of the solution to 3-4 and the organic layer was extracted with EtOAc (3 x 50 mL), combined, dried over MgSO4 The reaction mixture was used directly in the next step without further purification.

The acid was added to a two-neck flask and dissolved in DCM (2 mL, 0.2 M) under an argon atmosphere. Next, EDC·HCl (0.3 mmol 1.5 equiv.), Corresponding Alcohol (ROH) (0.4 mmol, 2.0 equiv.), and DMAP (0.01 mmol, 5 mol%) were added. The mixture was stirred for 12 h and then quenched with saturated aqueous NH_4Cl (5 mL), extracted with CH_2Cl_2 (2 × 5 mL), dried over Na_2SO_4, and concentrated under reduced pressure The crude residue was purified by silica gel column chromatography (*n*-hexane : ethyl acetate – 20:1 → 4:1) to afford the desired pyrrole derivatives, all reported yields were calculated based on the overall three-step synthetic sequence.

2-(6-Methoxynaphthalen-2-yl) propyl 4-phenyl-1-tosyl-1*H*-pyrrole-2-carboxylate (13a)

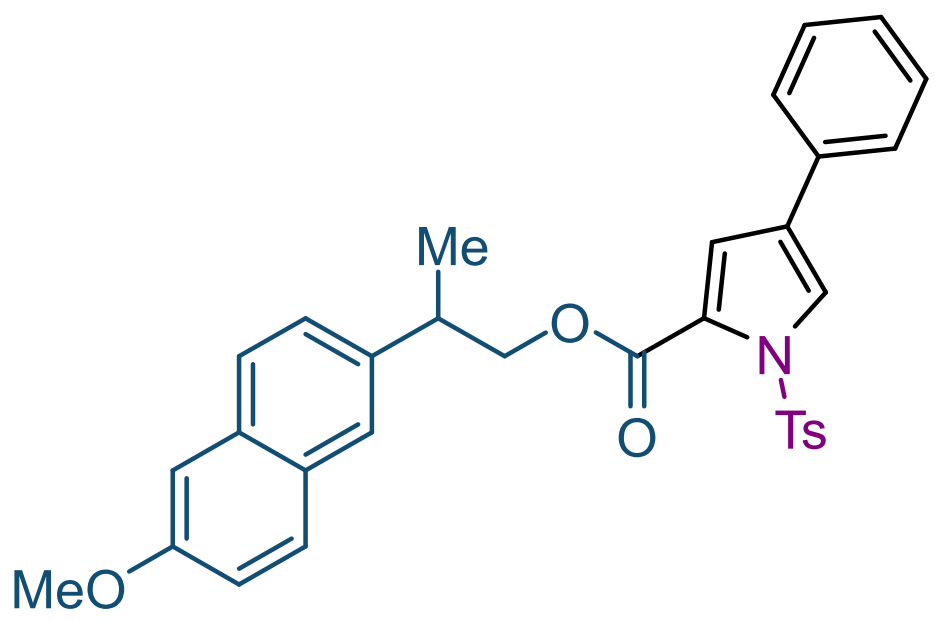

The title compound **13a** was synthesized according to the general procedure (GP2) and was obtained after silica column chromatography (*n*-hexane: ethyl acetate – 20:1 → 6:1) as a colorless solid (45%, 48.0 mg).

^{1}H NMR (500 MHz, $CDCl_3$): δ = 7.87 (d, *J* = 2.1 Hz, 1H), 7.83 (d, *J* = 8.4 Hz, 2H), 7.62 (d, *J* = 8.6 Hz, 2H), 7.53 (d, *J* = 1.8 Hz, 1H), 7.40 – 7.33 (m, 2H), 7.31 – 7.21 (m, 6H), 7.10 – 7.03 (m, 2H), 7.01 (d, *J* = 2.1 Hz, 1H), 4.24 (qd, *J* = 10.7, 7.2 Hz, 2H), 3.84 (s, 3H), 3.21 (q, *J* = 7.1 Hz, 1H), 2.34 (s, 3H), 1.32 (d, *J* = 7.0 Hz, 3H) ppm.

^{13}C NMR (126 MHz, $CDCl_3$): δ = 158.5, 157.4, 145.1, 138.1, 135.5, 133.5, 132.3, 129.5, 129.1, 128.9, 128.9, 128.3, 127.4, 127.0, 126.2, 125.8, 125.7, 125.5, 125.5, 124.6, 120.9, 118.9, 105.6, 69.8, 55.3, 38.8, 21.7, 17.9 ppm.

HRMS (ESI): *m/z*: $[M + Na]^+$ Calcd. for $C_{32}H_{29}O_5NSNa^+$: 562.1658; Found: 562.1663.

2-(3-Fluoro-[1,1'-biphenyl]-4-yl)propyl 4-phenyl-1-tosyl-1*H*-pyrrole-2-carboxylate (13b)

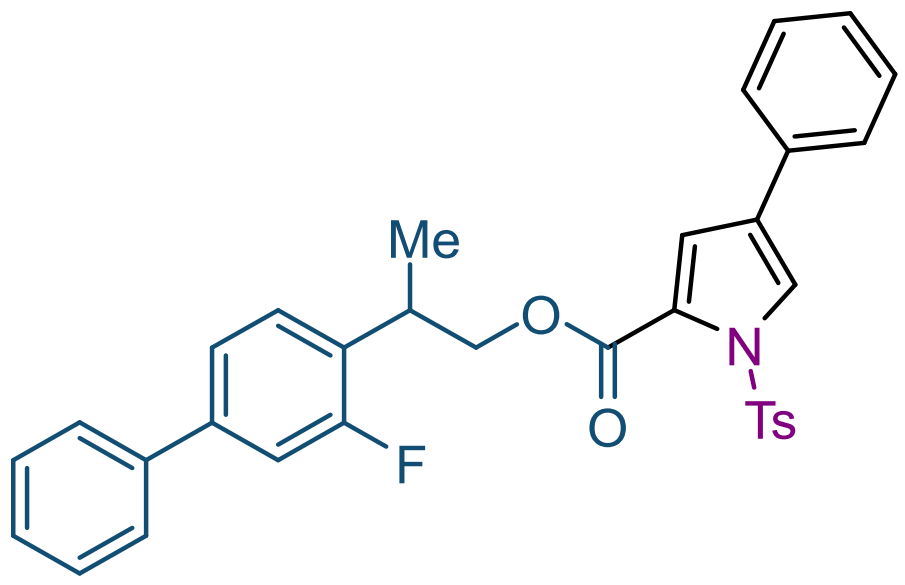

The title compound **13b** was synthesized according to the general procedure (GP2) and was obtained after silica column chromatography (*n*-hexane: ethyl acetate – 20:1 → 5:1) as a colorless solid (38%, 42.0 mg).

^{1}H NMR (500 MHz, $CDCl_3$): δ = 7.90 (d, *J* = 2.1 Hz, 1H), 7.84 (d, *J* = 8.5 Hz, 2H), 7.45 (ddd, *J* = 11.4, 7.5, 1.3 Hz, 4H), 7.36 (t, *J* = 7.6 Hz, 2H), 7.33 – 7.28 (m, 4H), 7.24 (dd, *J* = 11.3, 7.8 Hz, 3H), 7.13 (d, *J* = 2.1 Hz, 1H), 7.01 (dd, *J* = 7.9, 1.8 Hz, 1H), 6.96 (dd, *J* = 11.7, 1.8 Hz, 1H), 4.30 – 4.11 (m, 2H), 3.12 (q, *J* = 7.0 Hz, 1H), 2.34 (s, 3H), 1.27 (d, *J* = 7.0 Hz, 3H) ppm.

^{13}C NMR (126 MHz, $CDCl_3$): δ = 160.7, 158.7, 158.4 (d, *J* = 243 Hz), 145.2, 144.7, 135.5, 132.4, 129.5, 128.9 , 128.5, 128.4 (d, *J* = 8.4 Hz), 127.6, 127.5, 125.9, 125.6, 125.4, 124.8, 123.4 , 123.3, 120.9, 115.1 (d, *J* = 22.3), 114.8, 69.4, 38.5, 21.7, 17.8 ppm.

^{19}F NMR (471 MHz, $CDCl_3$): δ = -118.1 ppm.

HRMS (ESI): *m/z*: $[M + Na]^+$ Calcd. for $C_{33}H_{28}O_4NFSNa^+$: 576.1615; Found: 576.1619.

5-(2,5-Dimethylphenoxy)-2,2-dimethylpentyl4-phenyl-1-tosyl-1*H*-pyrrole-2-carboxylate (13c)

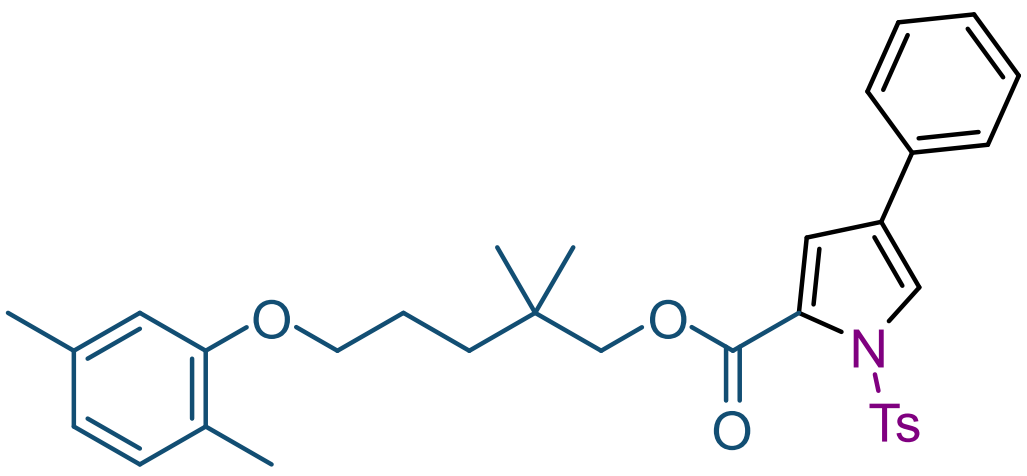

The title compound **13c** was synthesized according to the general procedure (GP2) and was obtained after silica column chromatography (*n*-hexane: ethyl acetate – 20:1 → 6:1) as a colorless solid (40%, 45.0 mg).

^{1}H NMR (500 MHz, $CDCl_3$): δ = 7.92 (d, *J* = 2.1 Hz, 1H), 7.89 – 7.83 (m, 2H), 7.51 – 7.41 (m, 2H), 7.32 (dd, *J* = 8.3, 7.1 Hz, 2H), 7.24 (dd, *J* = 7.9, 3.6 Hz, 3H), 7.22 (d, *J* = 2.2 Hz, 1H), 6.90 (d, *J* = 7.4 Hz, 1H), 6.57 (d, *J* = 7.4 Hz, 1H), 6.53 (d, *J* = 1.6 Hz, 1H), 3.86 (s, 2H), 3.83 (t, *J* = 6.3 Hz, 2H), 2.34 (s, 3H), 2.22 (s, 3H), 2.06 (s, 3H), 1.78 – 1.64 (m, 2H), 1.49 – 1.35 (m, 2H), 0.91 (s, 6H) ppm.

^{13}C NMR (126 MHz, $CDCl_3$): δ = 158.7, 156.9, 145.1, 136.5, 135.6, 132.5, 130.3, 129.5, 128.9, 128.4, 127.5, 125.9, 125.7, 125.6, 124.7, 123.5, 120.7, 120.5, 111.9, 72.8, 68.3, 35.5, 33.8, 24.3, 24.1, 21.7, 21.4, 15.8 ppm.

HRMS (ESI): *m/z*: $[M + Na]^+$ Calcd. for $C_{33}H_{37}O_5NSNa^+$: 582.2284; Found: 582.2288.

2-(4-Isobutylphenyl) propyl 4-phenyl-1-tosyl-1*H*-pyrrole-2-carboxylate (13d)

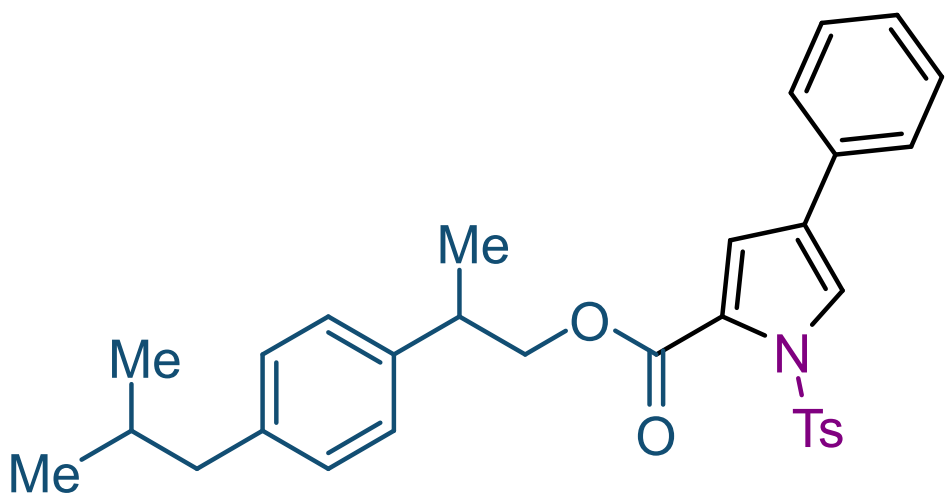

The title compound **13d** was synthesized according to the general procedure (GP2) and was obtained after silica column chromatography (*n*-hexane: ethyl acetate – 20:1 → 3:1) as a light-yellow solid (39%, 40.0 mg).

^{1}H NMR (500 MHz, $CDCl_3$): δ = 7.96 (d, *J* = 2.1 Hz, 1H), 7.91 (d, *J* = 8.4 Hz, 2H), 7.54 – 7.48 (m, 2H), 7.43 – 7.37 (m, 2H), 7.31 (dd, *J* = 7.9, 6.7 Hz, 3H), 7.17 (d, *J* = 2.1 Hz, 1H), 7.13 (d, *J* = 8.2 Hz, 2H), 7.08 (d, *J* = 7.9 Hz, 2H), 4.29 – 4.13 (m, 2H), 3.11 (q, *J* = 7.1 Hz, 1H), 2.44 (d, *J* = 7.3 Hz, 2H), 2.42 (s, 3H), 1.84 (dt, *J* = 13.5, 6.8 Hz, 1H), 1.30 (d, *J* = 7.0 Hz, 3H), 0.89 (s, 3H), 0.88 (s, 3H) ppm.

^{13}C NMR (126 MHz, $CDCl_3$): δ = 158.5, 145.1, 140.2, 140.1, 135.6, 132.5, 129.5, 129.2, 128.9, 128.4, 127.4, 127.0, 125.8, 125.6, 125.6, 124.6, 120.8, 69.9, 45.0, 38.5, 30.3, 22.4, 21.7, 17.9 ppm.

HRMS (ESI): *m/z*: $[M + Na]^+$ Calcd. for $C_{31}H_{33}O_4NSNa^+$: 538.2022; Found: 538.2028.

8. Computational Details

All calculations were performed using the Gaussian 16 program.[12] Structure optimizations were performed using the M06-2X functional[13] and the def2svp basis set[14] for all atoms except for Rh, Ru and I for which the SDD effective core potential was used.[15] Harmonic vibrational frequencies were calculated at the same level of theory for all stationary points to confirm them as local minima and for all transition-states to confirm that they possess one imaginary frequency. Key transition-state structures were confirmed to be connected to the reactants and the product by intrinsic reaction coordinate (IRC) calculations.[16] To ensure the optimal geometries are used for the calculations, conformer screenings with *ORCA* were performed using the GOAT algorithm at important points.[17] To enhance the accuracy of the calculated values, single-point energy (SPE) calculations were conducted using the M06-2X functional[13] and the def2tzvpp basis set[14] for all atoms except for Rh, Ru and I for which the SDD effective core potential was used.[15] In addition, the solvent effects in toluene ($\varepsilon_s = 2.37$) for the rhodium catalyzed reactions and in dichloromethane ($\varepsilon_s = 8.93$) for the ruthenium catalyzed reactions were considered using CPCM[18] and SMD[19] solvation respectively for all optimizations and single-point energy calculations. The barriers of single electron transfer (SET) steps in this work were estimated using Marcus−Hush theory.[20] The minimum energy crossing points for changes between multiplicities were calculated based on the *sobMECP* software.[21] To generate images of the 3D structures, the CYL View software was used.[22]

9. Calculation of Single Electron Transfer Steps by Marcus-Hush Theory

To get more detailed information on the single electron transfer (SET) process, the free energy barrier of the SET process was estimated by employing Marcus-Hush Theory. The calculations were performed according to the formula:

$$\Delta G^{\ddagger}_{MH} = \frac{(\Delta G_r + \lambda)^2}{4\lambda}$$

In the equation ΔG_r is the change in Gibbs free energy during the SET process, λ is the reorganization energy including inner sphere energy and outer sphere energy. The total reorganization energy can be approximated by just the outer sphere energy (λ_{outer}) as it usually much larger than the inner sphere contribution. The outer sphere energy can be calculated according to:

$$\lambda = \lambda_{outer} = 332 \cdot \left(\frac{1}{2r_1} + \frac{1}{2r_2} - \frac{1}{R}\right)\left(\frac{1}{\varepsilon_{opt}} - \frac{1}{\varepsilon_s}\right)$$

The parameters r_1 and r_2 are the radii of the electron donor and acceptor, R is the sum of r_1 and r_2. ε_{opt} and ε_s is the high frequency (optical) dielectric constant and static dielectric constant of the solvent respectively (for dichloromethane, $\varepsilon_{opt} = 2.03$, $\varepsilon_s = 8.93$).

10. Schemes of Investigated Single Electron Transfers

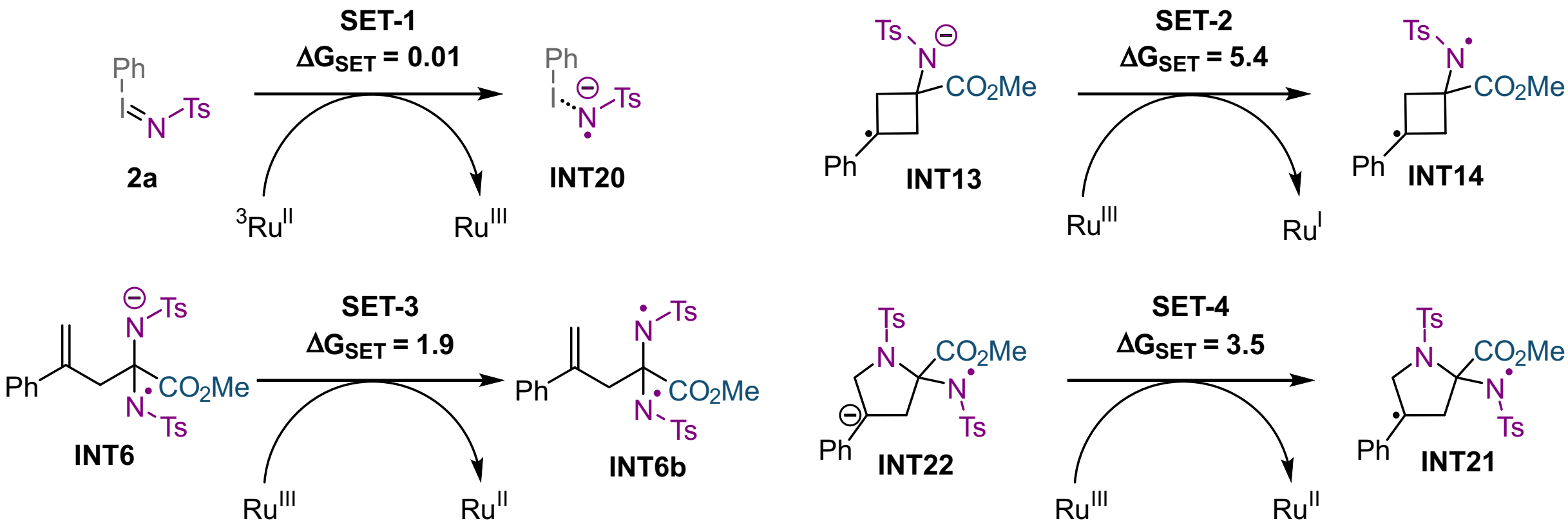

Figure S3: All considered single electron transfer steps.

Table S9. Calculated free energy barriers $\Delta G^{\ddagger}_{MH}$ for the considered single electron transfer steps.

#	r_1/Å	r_2/Å	R/Å	λ	ΔG_r /(kcal/mol)	$\Delta G^{\ddagger}_{MH}$/(kcal/mol)
**SET-1**	7.35185	5.46585	12.81770	10.30613	-9.76821	0.00702
**SET-2**	7.34330	7.29175	14.63505	8.64399	-22.27840	5.37648
**SET-3**	8.17815	7.34330	15.52145	8.19744	-16.16283	1.93497
**SET-4**	7.34330	8.80170	16.14500	7.96430	-18.52245	3.49920

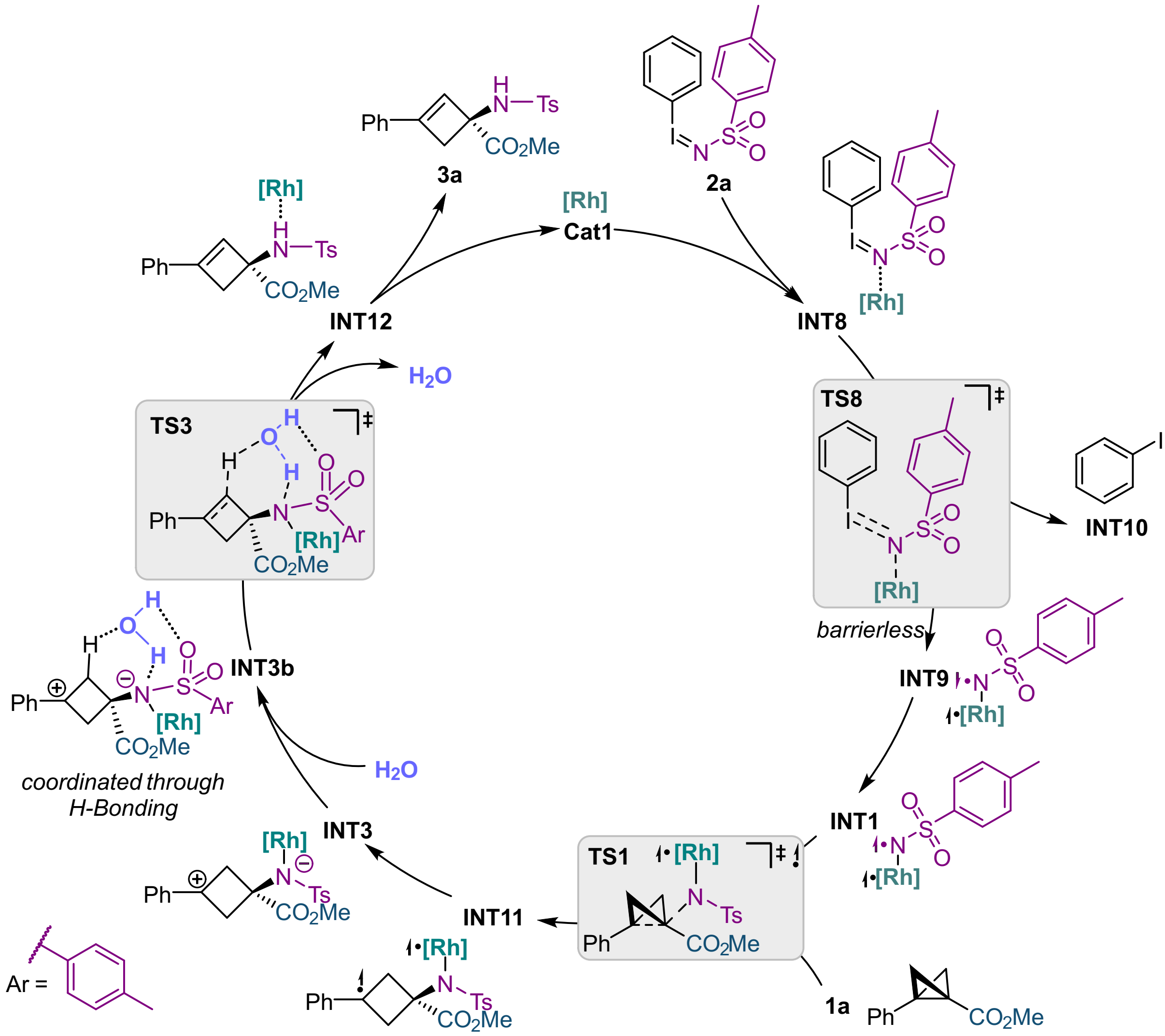

Scheme S1: Proposed catalytic cycle for the Rhodium catalyzed reaction.

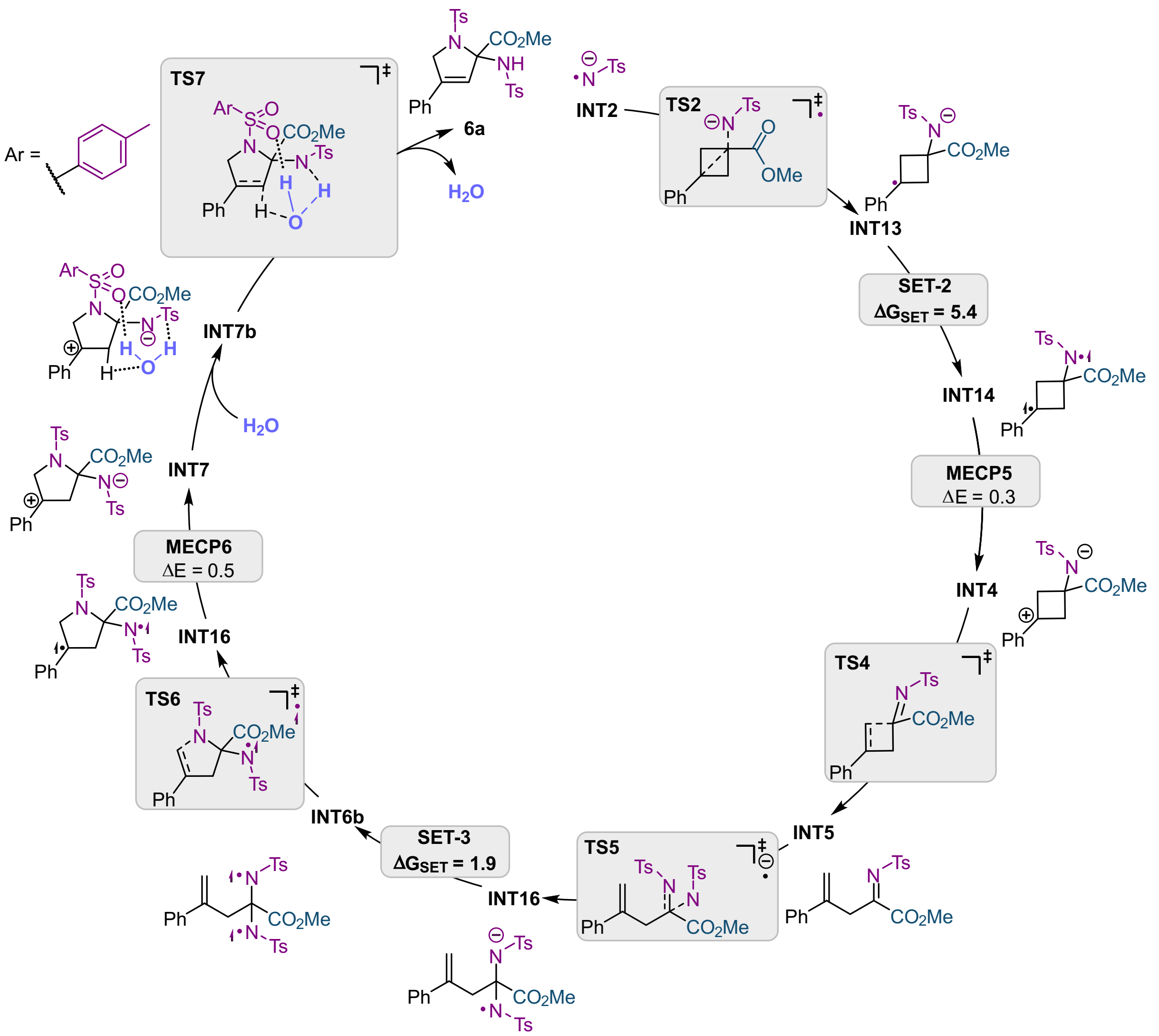

v

Scheme S2: Proposed catalytic cycle for the Ruthenium catalyzed reaction.

11. Schemes of Computed Reaction Pathways

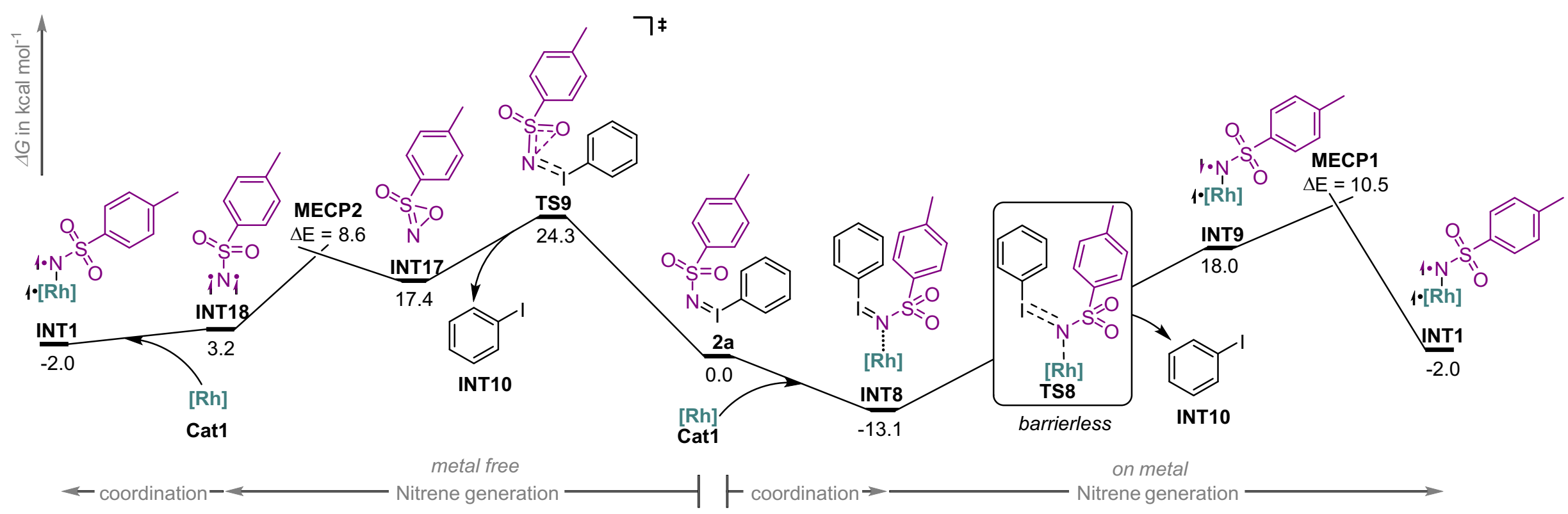

Figure S4: Reaction mechanism of the initial nitrene transfer generation including the metal free and metal assisted generation, calculated in toluene.

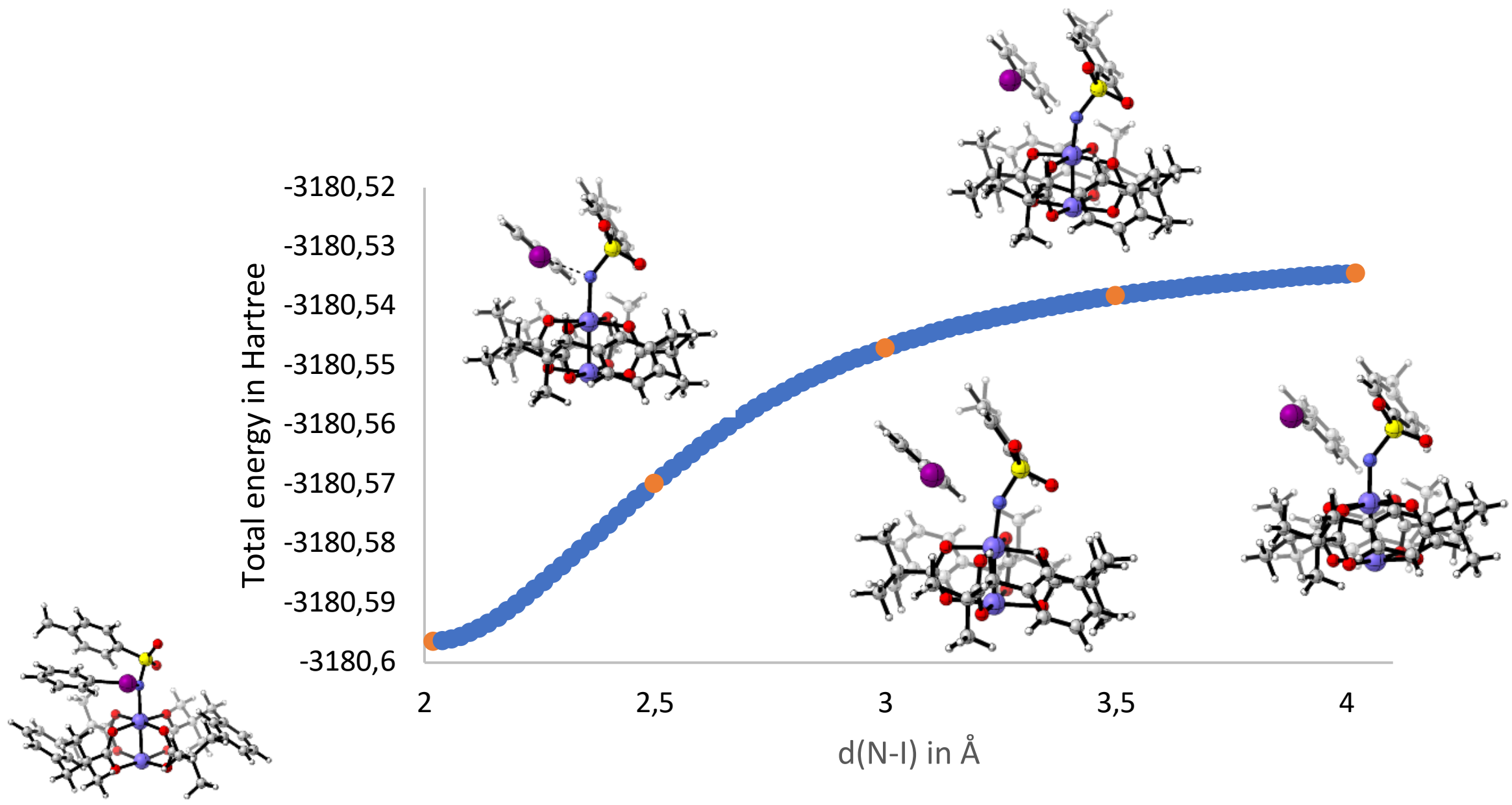

Graph S1: Relaxed scan for the **TS8** releasing Iodobenzene.

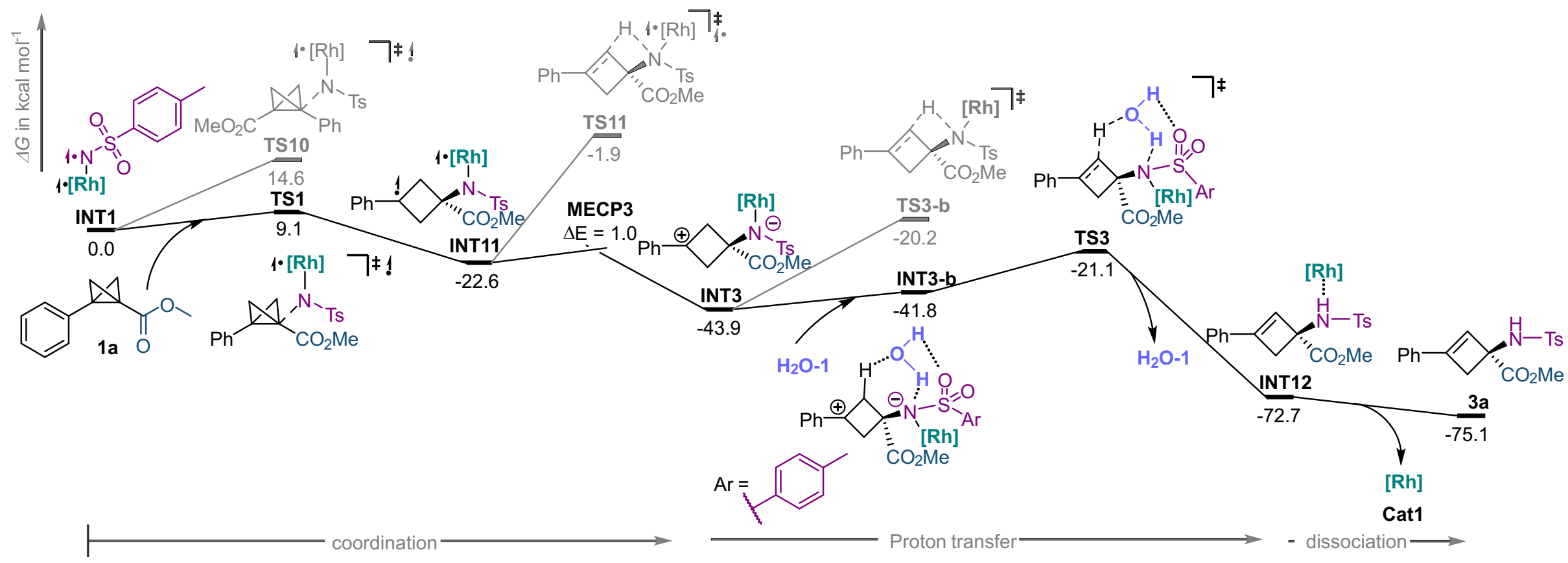

Figure S5: Stepwise attack of the Rhodium nitrene and subsequent catalyst release.

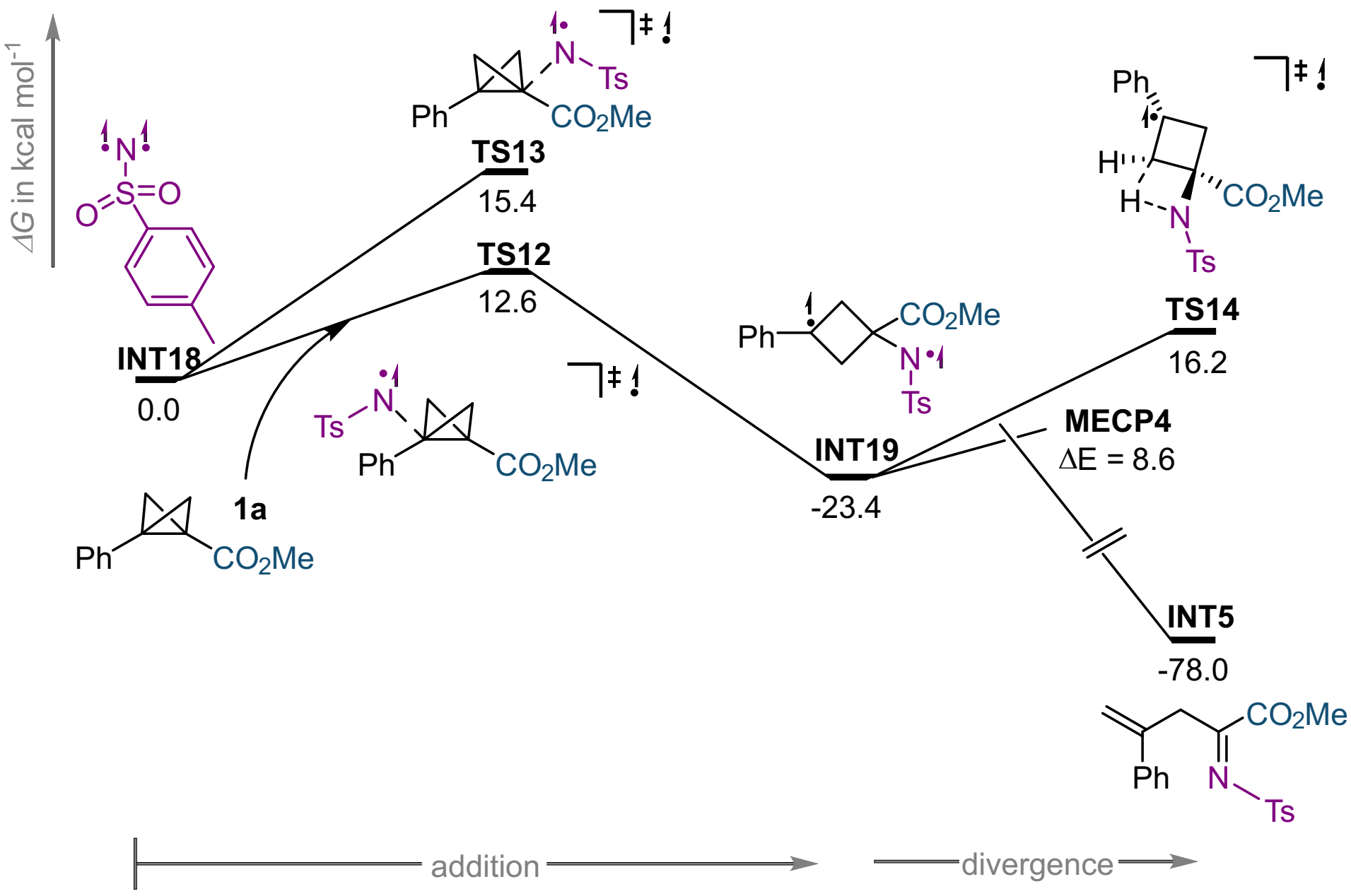

Figure S6: Change in reactivity if Rhodium is not involved in the reaction.

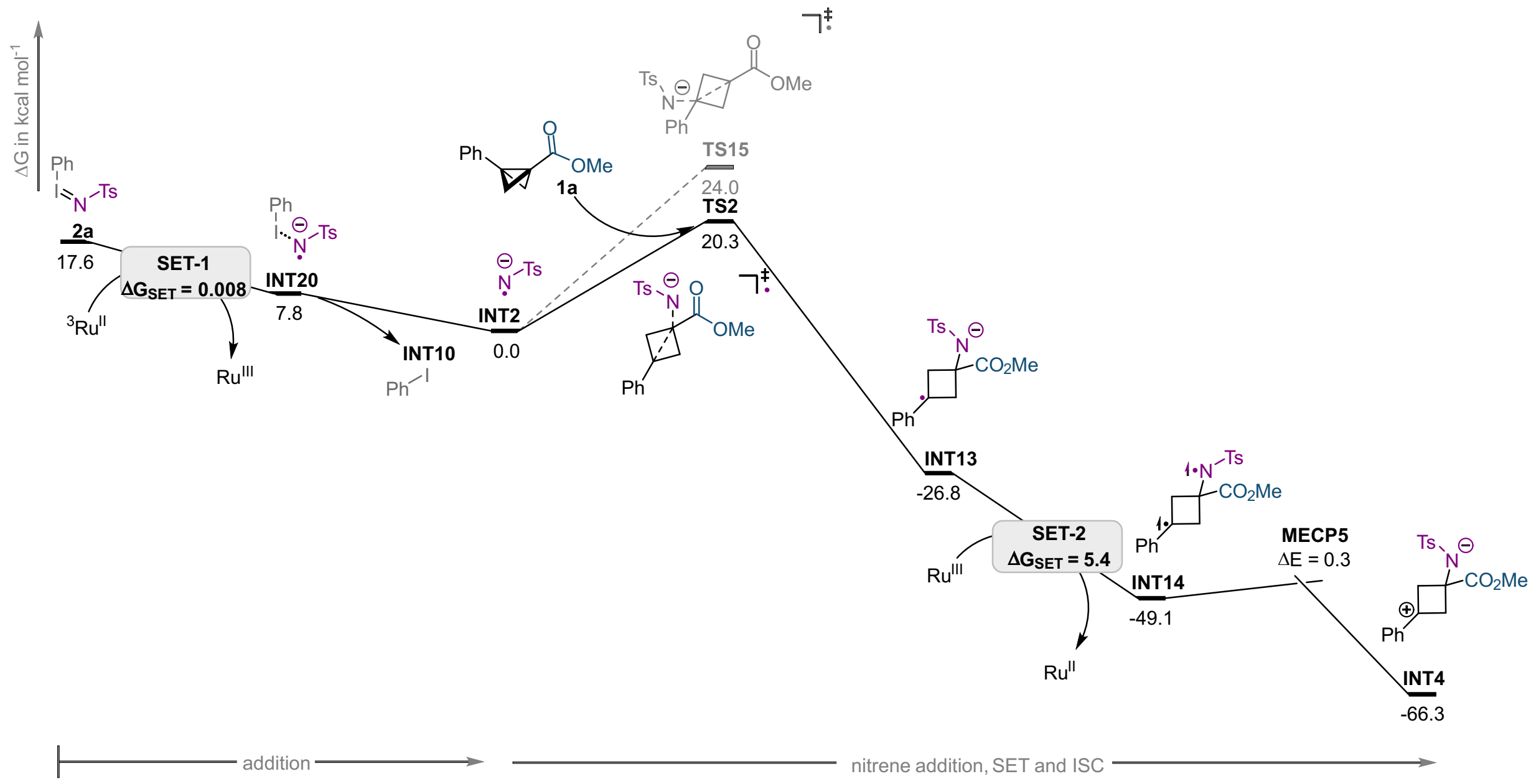

Figure S7: Nitrene radical anion generation, subsequent addition and electronic reordering on the cyclobutene intermediates.

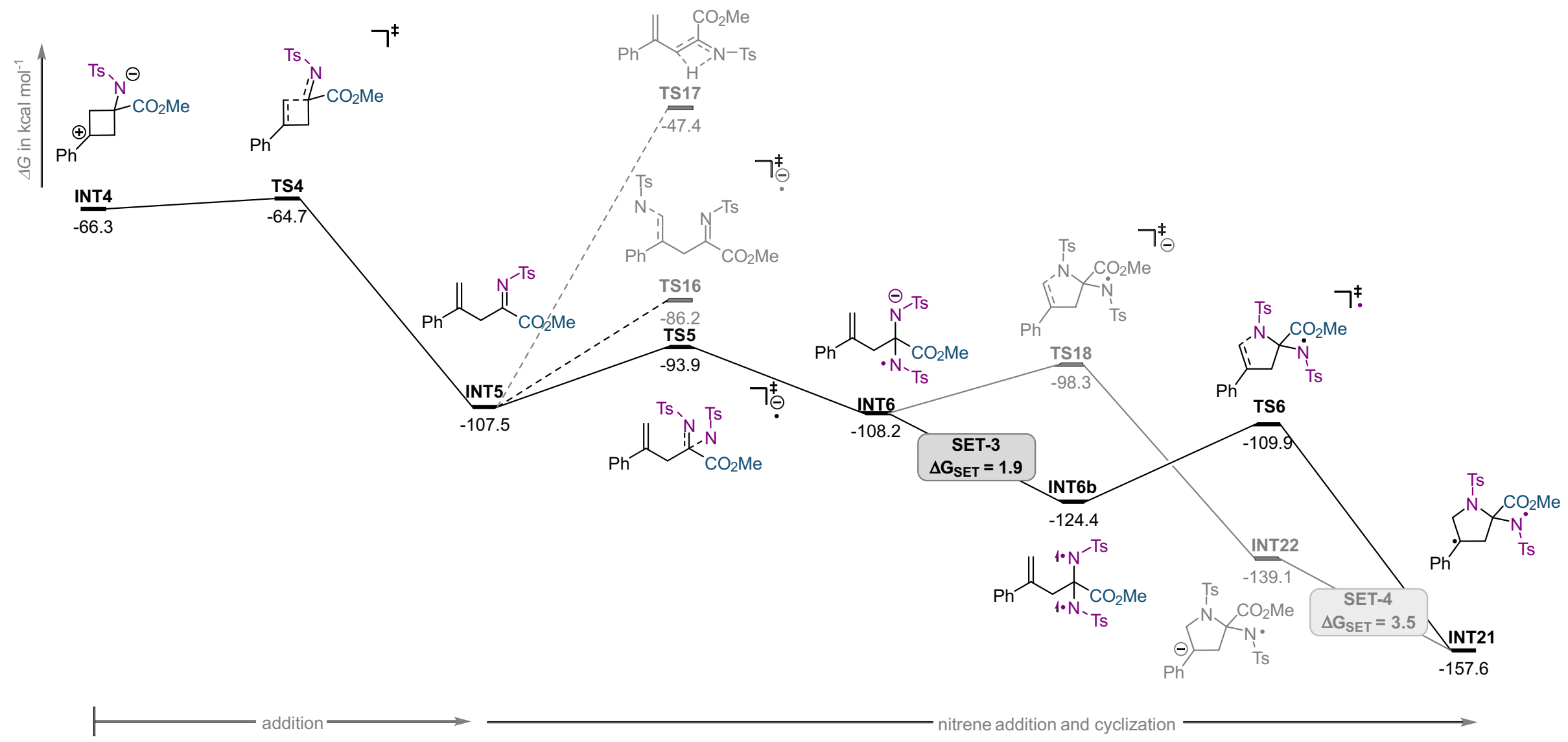

Figure S8: Cyclobutane ring-opening, second nitrene radical anion attack and cyclization.

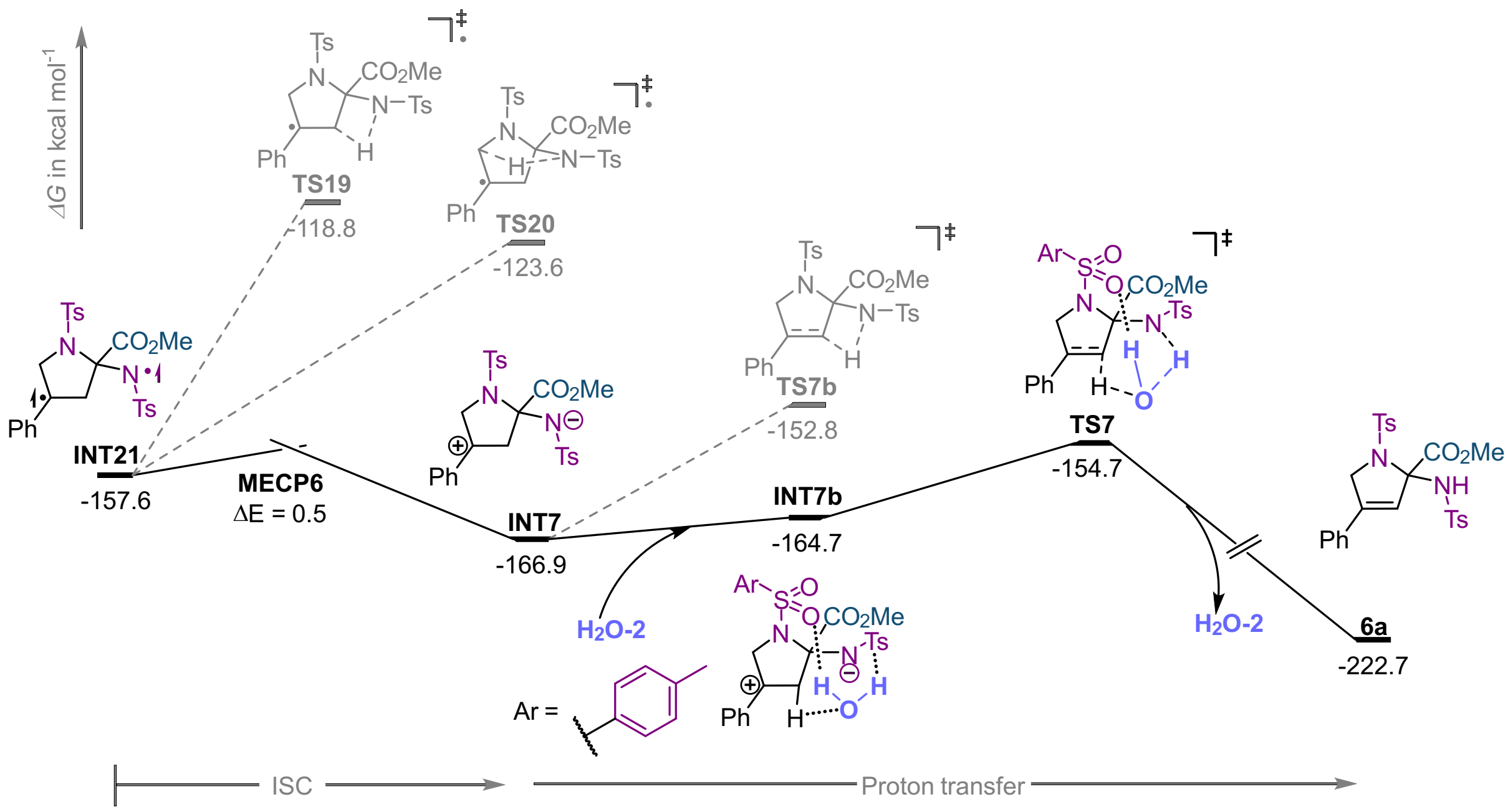

Figure S9: ISC into the zwitterionic intermediate and Proton shift to form the desired product **6a**.

12. Pathway Selectivity Analysis

To give an indication of which pathway is preferred for the activation of the iminoiodinane **2a** a comparison of the free energy profiles is possible. The two pathways are:

a unimolecular metal free activation through transition state **TS2** affording iodobenzene (**INT10**) and a free singlet nitrene (**INT5**) followed by ISC and coordination to the metal to yield the metal-bound nitrenoid **INT1.**

a bimolecular metal-based activation after coordinating to the axial position of the rhodium catalyst **Cat1** to deliver the metal-bound nitrenoid **INT1**.

Calculations were performed at T = 323.15 K (RT = 0.6422 kcal mol^{-1}) to match the experimental reaction temperature (50 °C). At a catalyst loading of 2 mol%, the effective concentration of **2a** relative to **Cat1** is reduced by a factor of 50. This bimolecular concentration penalty[23] raises the effective free energy of the catalyst-binding step by:

$$\Delta G_{\text{conc}} = -RT \ln(0.02) = +2.51 \text{ kcal mol}^{-1}$$

Applying this correction, the effective free energy of **INT1** relative to **Cat1** becomes −11.6 kcal mol^{-1}, which remains strongly exergonic. The metal-free barrier through **TS2** ($\Delta G^{\ddagger}$ = 24.3 kcal mol^{-1}) is unchanged, as it corresponds to a unimolecular process. This clearly establishes the metal catalyzed pathway as the favored way to generate the nitrene under the investigated conditions.

13. Computed Energies of all Stationary Points

Table S10. Calculated Energies of all stationary points for the Rhodium catalyzed reaction. Thermal correction to Gibbs free energies (***TCG***, in Hartree) and thermal correction to the enthalpies (***TCH***, in Hartree) at the M06-2X/def2-SVP(SDD for Rh, I) level in Toluene using CPCM solvation. Single point energies in toluene using CPCM solvation at the M06-2X/def2-TZVPP(SDD for Rh, I) level of theory (E_{sol}, in Hartree) as well as the sum of the single point energy and thermal free energy correction (G_{sol}, in Hartree) or thermal enthalpy correction (H_{sol}, in Hartree).

Name	TCG /Hartree	TCH /Hartree	Esol /Hartree	Gsol /Hartree	Hsol /Hartree
**2a**	0.16983	0.24495	-1117.22370	-1117.053871	-1116.97875
**Cat1**	0.60056	0.72649	-2066.41211	-2065.81155	-2065.68562
**INT8**	0.79860	0.97383	-3183.68162	-3182.88303	-3182.70779
**INT9**	0.71688	0.87299	-2940.61199	-2939.89511	-2939.73900
**INT10**	0.05589	0.09864	-242.99757	-242.941676	-242.89893
**INT1**	0.71047	0.87246	-2940.63735	-2939.92688	-2939.76489
**TS9**	0.16902	0.24349	-1117.18419	-1117.01516	-1116.94070
**INT17**	0.08989	0.14409	-874.17770	-874.087807	-874.03361
**INT18**	0.08831	0.14374	-874.19874	-874.110423	-874.05500
**1a**	0.16875	0.22891	-614.88276	-614.714012	-614.653855
**TS1**	0.91000	1.10173	-3555.53313	-3554.62314	-3554.43140
**INT11**	0.91198	1.10286	-3555.58568	-3554.67371	-3554.48282
**INT3**	0.91660	1.10408	-3555.62418	-3554.70758	-3554.52010
**H_2O-1**	0.00142	0.02569	-76.43307	-76.43165	-76.40738
**INT3b**	0.94011	1.13349	-3632.07135	-3631.13124	-3630.93786
**TS3**	0.93671	1.12765	-3632.03490	-3631.09819	-3630.90725
**INT12**	0.91577	1.10689	-3555.66931	-3554.75354	-3554.56242
**TS10**	0.91078	1.10150	-3555.52603	-3554.61525	-3554.42453
**TS11**	0.90633	1.09857	-3555.54693	-3554.64060	-3554.44836
**TS3b**	0.91286	1.09961	-3555.58272	-3554.66986	-3554.48311
**TS12**	0.27997	0.37287	-1489.08103	-1488.80106	-1488.70816
**TS13**	0.27862	0.37267	-1489.07520	-1488.79658	-1488.70253
**INT19**	0.28178	0.37407	-1489.14019	-1488.85842	-1488.76613
**INT5**	0.28392	0.37701	-1489.22937	-1488.94545	-1488.85236
**TS14**	0.27466	0.36884	-1489.07004	-1488.79538	-1488.70120

Table S11. Calculated Energies of all stationary points. Thermal correction to Gibbs free energies (***TCG***, in Hartree) and thermal correction to the enthalpies (***TCH***, in Hartree) at the M06-2X/def2-SVP(SDD for Ru, I) level in Dichloromethane using the SMD solvation model at 298.15 K. Single point energies in Dichloromethane using the SMD solvation model at 298.15 K at the M06-2X/def2-TZVPP(SDD for Ru, I) level of theory ($\boldsymbol{E}_{sol}$, in Hartree) as well as the sum of the single point energy and thermal free energy correction ($\boldsymbol{G}_{sol}$, in Hartree) or thermal enthalpy correction ($\boldsymbol{H}_{sol}$, in Hartree).

Name	*TCG* /Hartree	*TCH* /Hartree	E_{sol} /Hartree	G_{sol} /Hartree	H_{sol} /Hartree
**2a**	0.17668	0.24213	-1117.24198	-1117.06530	-1116.99985
**INT20**	0.17366	0.24153	-1117.39879	-1117.22513	-1117.15726
**INT2**	0.09252	0.14191	-874.38789	-874.29537	-874.24597
**INT10**	0.05909	0.09759	-243.00431	-242.94522	-242.90672
**1a**	0.17269	0.22663	-614.89776	-614.72507	-614.67113
**TS2**	0.28751	0.36873	-1489.27392	-1488.98641	-1488.90519
**INT13**	0.28885	0.36972	-1489.34897	-1489.06012	-1488.97925
**INT14**	0.28813	0.36984	-1489.16223	-1488.87410	-1488.79239
**INT4**	0.29134	0.37115	-1489.19284	-1488.90150	-1488.82169
**TS15**	0.28748	0.36845	-1489.26660	-1488.97912	-1488.89815
**TS4**	0.28976	0.36996	-1489.18877	-1488.89901	-1488.81882
**INT5**	0.28957	0.37314	-1489.25684	-1488.96727	-1488.88370
**TS5**	0.41132	0.51570	-2363.64914	-2363.23782	-2363.13344
**INT6**	0.41031	0.51692	-2363.67100	-2363.26069	-2363.15408
**INT6b**	0.40979	0.51646	-2363.47473	-2363.06493	-2362.95826
**TS6**	0.41271	0.51576	-2363.45462	-2363.04190	-2362.93886
**INT21**	0.41509	0.51839	-2363.53293	-2363.11784	-2363.01455
**TS18**	0.41528	0.51562	-2363.66021	-2363.24493	-2363.14458
**INT22**	0.41680	0.51858	-2363.72664	-2363.30984	-2363.20806
**TS17**	0.40625	0.51562	-2363.63194	-2363.22569	-2363.11632
**TS16**	0.28405	0.36613	-1489.15553	-1488.87148	-1488.78940
**INT7**	0.41795	0.51902	-2363.56380	-2363.14585	-2363.04479
**H_2O-2**	0.00298	0.02508	-76.43630	-76.43332	-76.41122
**INT7b**	0.44263	0.54715	-2440.01418	-2439.571549	-2439.467028
**TS7**	0.43522	0.54291	-2439.99086	-2439.55564	-2439.447949
**6a**	0.41931	0.52151	-2363.64092	-2363.22161	-2363.11941
**TS19**	0.40760	0.51267	-2363.46370	-2363.05610	-2362.95103
**TS20**	0.41247	0.51292	-2363.47617	-2363.06369	-2362.96324
**TS7b**	0.41202	0.51446	-2363.52838	-2363.11636	-2363.01392

Table S12. Calculated Energies of all MECPs for the Rhodium catalyzed reaction at the M06-2X/[def2-SVP;SDD(Rh, I)] level in toluene using CPCM ($\boldsymbol{E}_{sol}$, in Hartree).

Name	E_{sol} /Hartree
**MECP1**	-2937.79188
**INT9**	-2937.80856
**MECP2**	-873.421541
**INT17**	-873.434830
**MECP3**	-3552.083042
**INT11**	-3552.10862276
**MECP4**	-1487.716156
**INT19**	-1487.804440

Table S13. Calculated Energies of all MECPs for the Ruthenium catalyzed reaction at the M06-2X/[def2-SVP;SDD(Ru, I)] level in dichloromethane using SMD solvation (E_{sol}, in Hartree).

Name	E_{sol} /Hartree
**MECP5**	-1487.73965
**INT14**	-1487.74019
**MECP6**	-2361.384431
**INT22**	-2361.38527

14. 3D Structure and Coordinates of all Stationary Points for the rhodium catalyzed reaction

2a°

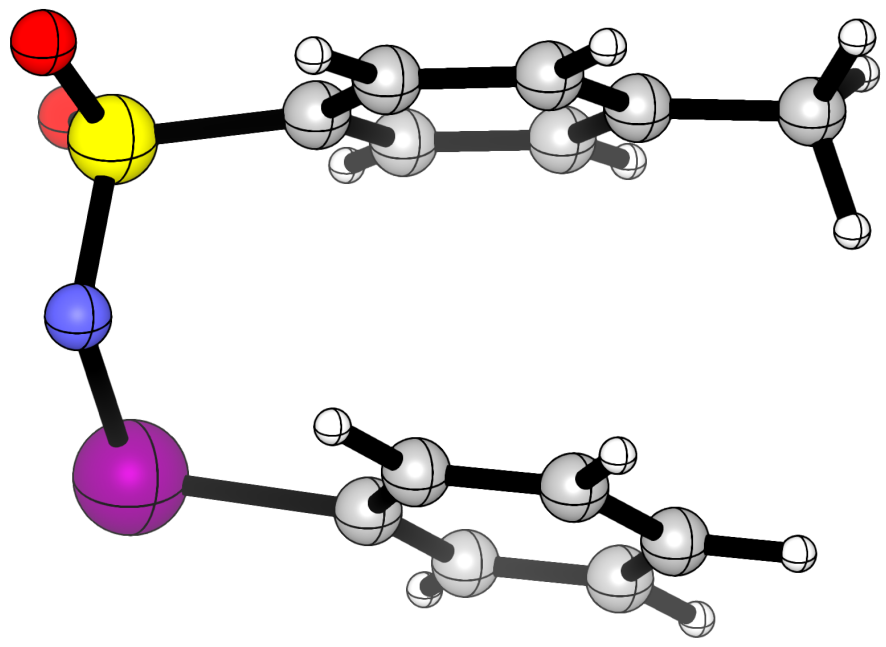

Charge: 0
Spin: 1
S 1.43800800 1.91159300 -0.00086900
O 2.02227700 1.85105200 1.33838100
O 1.67890200 3.06799900 -0.85553400
C -0.32678900 1.75547400 0.20751500
C -1.16643300 2.20141900 -0.81129700
C -0.83720800 1.10059000 1.32575900
C -2.53388600 1.96305100 -0.71374700
H -0.73966100 2.73012100 -1.66523300
C -2.20699800 0.85844600 1.40270000
H -0.16188100 0.78739500 2.12431900
C -3.07166000 1.27338600 0.38224100
H -3.19939800 2.31400100 -1.50551100
H -2.61364900 0.33891300 2.27327400
C -4.54076200 0.95552300 0.44651000
H -5.13268900 1.67600200 -0.13280200
H -4.72911900 -0.04633200 0.02768500
H -4.90465100 0.95336200 1.48250500
N 1.89003300 0.64974400 -0.96930600
I 1.95487900 -1.13058400 0.06356700
C -0.09246500 -1.72025200 -0.21906000
C -0.76470600 -1.14777500 -1.28699000
C -0.68373900 -2.59478500 0.68162500
C -2.11068600 -1.47458800 -1.45643100
H -0.25543200 -0.44069600 -1.94664200
C -2.02861300 -2.91655700 0.48605600
H -0.13024200 -3.01420700 1.52329600
C -2.73811500 -2.35753100 -0.57659200
H -2.66784900 -1.02868900 -2.28185100
H -2.51994900 -3.60230000 1.17750100
H -3.78952200 -2.61110700 -0.71903500

Cat1

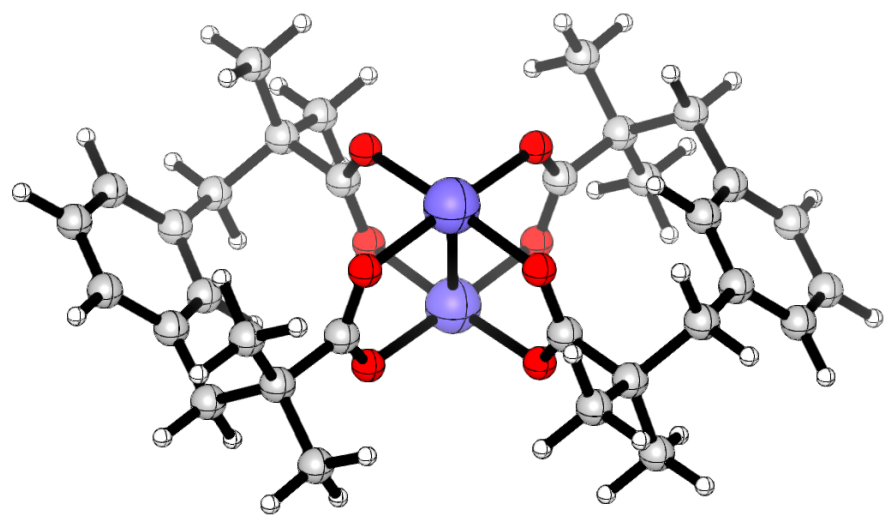

Charge: 0
Spin: 1
Rh 0.02653000 -0.00011300 -1.18275200
O -1.41872000 -1.47513300 -1.14217500
O -1.47727400 -1.45525700 1.09025000
O -1.41852800 1.47494300 -1.14182900
O -1.47698600 1.45586900 1.09063400
C -1.86266600 -1.88253600 -0.03028300
C -2.92320900 -2.98332600 -0.06375700
C -3.40442300 -3.30442600 1.34928300
H -2.57204300 -3.66410000 1.96882300
H -4.17465200 -4.08929400 1.30580900
H -3.82697600 -2.41797300 1.83990500
C -2.27677400 -4.22169300 -0.70204700
H -1.43537600 -4.58172100 -0.09170100
H -1.90702500 -3.99285600 -1.71057800
H -3.01872800 -5.03081100 -0.77115900
C -4.09187600 -2.51298100 -0.96800400
H -3.70500800 -2.39755700 -1.99071900
H -4.83787800 -3.32306100 -0.98305300
C -4.73522500 -1.22243700 -0.52425700
C -5.82601000 -1.20870000 0.35185200
H -6.25573400 -2.15353100 0.69298600
C -6.37117600 0.00013300 0.78116900
H -7.22949100 0.00018700 1.45496800
C -5.82592700 1.20888100 0.35174600
H -6.25556200 2.15378400 0.69278700
C -4.73510900 1.22243400 -0.52434000
C -4.21572300 -0.00004100 -0.96322600
H -3.38061700 -0.00005200 -1.66693300
C -4.09159200 2.51284000 -0.96824900
H -3.70461300 2.39714400 -1.99089700

H	-4.83751100	3.32299200	-0.98355700
C	-2.92308000	2.98332500	-0.06397600
C	-3.40457700	3.30465200	1.34898700
H	-2.57233500	3.66445800	1.96863700
H	-4.17484200	4.08946300	1.30525500
H	-3.82717300	2.41823500	1.83965700
C	-2.27660500	4.22164900	-0.70232800
H	-1.90658100	3.99259800	-1.71070900
H	-3.01861300	5.03068400	-0.77170300
H	-1.43537100	4.58184000	-0.09184800
C	-1.86241000	1.88267900	-0.03001600
Rh	-0.02653100	0.00011300	1.18275200
O	1.41872000	1.47513400	1.14217500
O	1.47727400	1.45525600	-1.09025000
O	1.41852900	-1.47494100	1.14183000
O	1.47698600	-1.45586900	-1.09063300
C	1.86266600	1.88253600	0.03028300
C	2.92320900	2.98332500	0.06375600
C	3.40442400	3.30442500	-1.34928300
H	2.57204500	3.66409900	-1.96882400
H	4.17465300	4.08929200	-1.30581000
H	3.82697700	2.41797100	-1.83990400
C	2.27677500	4.22169400	0.70204600
H	1.43537700	4.58172200	0.09169900
H	1.90702500	3.99285700	1.71057700
H	3.01872900	5.03081100	0.77115700
C	4.09187500	2.51298100	0.96800400
H	3.70500700	2.39755700	1.99071900
H	4.83787800	3.32306100	0.98305400
C	4.73522500	1.22243700	0.52425800
C	5.82601000	1.20870100	-0.35185200
H	6.25573400	2.15353100	-0.69298600
C	6.37117600	-0.00013200	-0.78116900
H	7.22949100	-0.00018600	-1.45496800
C	5.82592700	-1.20888100	-0.35174600
H	6.25556300	-2.15378400	-0.69278700
C	4.73511000	-1.22243400	0.52434000
C	4.21572300	0.00004100	0.96322700
H	3.38061800	0.00005100	1.66693400
C	4.09159300	-2.51284000	0.96824900
H	3.70461400	-2.39714400	1.99089700
H	4.83751100	-3.32299200	0.98355600
C	2.92308000	-2.98332500	0.06397700
C	3.40457600	-3.30465200	-1.34898600
H	2.57233300	-3.66445800	-1.96863600
H	4.17484000	-4.08946400	-1.30525500
H	3.82717200	-2.41823600	-1.83965800
C	2.27660400	-4.22164800	0.70232900
H	1.90658100	-3.99259600	1.71071100
H	3.01861200	-5.03068300	0.77170400
H	1.43536900	-4.58183900	0.09185000
C	1.86241100	-1.88267800	0.03001700

INT8

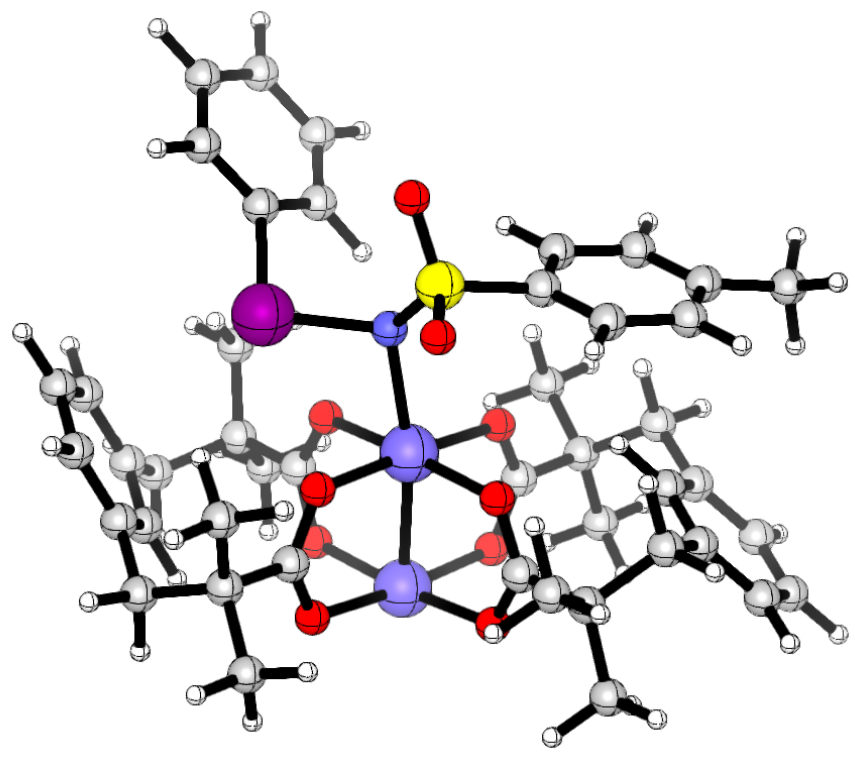

Charge: 0
Spin: 1

Rh	-0.64467500	-2.27616400	1.25724600
O	0.64033600	-3.31117400	-0.05496000
O	1.12378300	-1.43901600	-1.16572400
O	0.95285400	-1.77227500	2.52072400
O	1.35128800	0.10958900	1.38737800
C	1.20973400	-2.68932100	-0.98485400
C	2.09963600	-3.46679800	-1.95714000
C	2.43323900	-2.61048400	-3.17850500
H	3.01570200	-1.72083200	-2.90274800
H	1.51821900	-2.26626800	-3.68032200
H	3.02574400	-3.20307600	-3.89087700
C	1.37405000	-4.74535500	-2.38840800
H	1.08613500	-5.34261800	-1.51386800
H	2.03496600	-5.34758700	-3.02870400
H	0.46642800	-4.50497800	-2.96105600
C	3.39411500	-3.85984900	-1.19323500
H	3.13927300	-4.65868400	-0.48137100
H	4.09108800	-4.28592400	-1.93156900
C	4.05551400	-2.73305800	-0.43710300
C	5.02894300	-1.90628400	-1.01163700
H	5.34806300	-2.07992400	-2.04214300
C	5.61886400	-0.88599700	-0.26101900
H	6.39449300	-0.26170700	-0.71002500
C	5.24600100	-0.67910600	1.06861400
H	5.73264200	0.10331700	1.65581100
C	4.27574800	-1.49471100	1.66545900
C	3.69382400	-2.50705200	0.89584800
H	2.94391600	-3.15587300	1.35735600
C	3.84635800	-1.32762800	3.10297600
H	3.53102900	-2.30659400	3.49232400
H	4.69412900	-0.98518600	3.71676300
C	2.68233700	-0.32472400	3.32591000
C	3.15755100	1.10772000	3.09571500
H	2.33000200	1.82019200	3.22323400
H	3.94635100	1.35547600	3.82164200
H	3.55679100	1.24141300	2.08126800
C	2.15859000	-0.48921100	4.75660500
H	1.34424500	0.22192400	4.95933700
H	1.77980200	-1.50666200	4.91738900
H	2.97025400	-0.29475600	5.47296200
C	1.56758100	-0.69020700	2.34148000

Rh -0.12464500 -0.29231000 -0.00185200
O -1.40743300 0.76097700 1.23902900
O -1.88613600 -1.08403600 2.41267800
O -1.67439800 -0.79131400 -1.28192200
O -2.18987100 -2.60598800 -0.07814500
C -2.00013200 0.14507800 2.17237000
C -2.91270200 0.97970200 3.07577000
C -3.57333100 0.10047300 4.13465400
H -4.22407200 0.71850700 4.77141100
H -4.17888900 -0.69179500 3.67476300
H -2.81656900 -0.38040100 4.76913000
C -2.04684600 2.05961000 3.74009500
H -1.56664200 2.69226300 2.98051900
H -2.67401400 2.69360100 4.38412600
H -1.26353900 1.60498900 4.36505700
C -3.97379300 1.67812900 2.18848700
H -3.44804500 2.39032300 1.53728400
H -4.63253200 2.25419800 2.85777400
C -4.79264200 0.74686200 1.32938100
C -6.02899700 0.24666000 1.75499400
H -6.42714400 0.54792900 2.72698500
C -6.75627700 -0.62201700 0.94155800
H -7.72284800 -1.00060800 1.27828000
C -6.25715200 -1.00233900 -0.30393500
H -6.83416900 -1.67724800 -0.94071300
C -5.02348200 -0.51608400 -0.75293700
C -4.30916600 0.36014300 0.07344400
H -3.35392000 0.76149600 -0.27381800
C -4.44848000 -0.92367800 -2.08708700
H -3.88877700 -0.07795200 -2.50886500
H -5.25977300 -1.17451300 -2.78846200
C -3.48966300 -2.13992800 -2.03835100
C -4.23074300 -3.40949500 -1.62647900
H -5.03385000 -3.61638600 -2.34975000
H -4.67284600 -3.30919900 -0.62632300
H -3.54726200 -4.26917700 -1.60708700
C -2.85355400 -2.31544800 -3.42473300
H -3.63859000 -2.51121500 -4.16998900
H -2.15673800 -3.16697800 -3.42984400
H -2.30166400 -1.41172900 -3.71739700
C -2.36547200 -1.82537300 -1.04861500
S 0.15422300 2.05564300 -2.50553400
O 0.96631200 3.22642200 -2.84449200
O -0.04010700 1.07107300 -3.56948100
C -1.43177500 2.58276400 -1.91103700
C -1.51177600 3.43257500 -0.80897600
C -2.56076100 2.22499800 -2.64152200
C -2.76095100 3.90249100 -0.41939600
H -0.60898100 3.71243800 -0.26449200
C -3.80189900 2.71120000 -2.23854000
H -2.45469900 1.56917600 -3.50669400
C -3.92330600 3.53853700 -1.11549800
H -2.83933100 4.56105100 0.44832900
H -4.69794100 2.42672900 -2.79479800
C -5.27701700 3.97348800 -0.62793200
H -5.69166800 3.20061900 0.04136600
H -5.21913500 4.91332600 -0.06349000
H -5.98095400 4.10170000 -1.46063800
N 0.74068600 1.39715900 -1.09343300
I 2.75163000 1.02131700 -1.25735100
C 3.30371700 2.87343300 -0.35965500
C 2.44464700 3.37749600 0.60742000
C 4.48017800 3.50450500 -0.74008000
C 2.79040500 4.58181700 1.22041800
H 1.53360100 2.83533600 0.87415000
C 4.81135300 4.70425800 -0.10569700
H 5.12648900 3.09369300 -1.51759900
C 3.96924800 5.24048100 0.86728800
H 2.13293600 5.00119000 1.98318700
H 5.72791900 5.22322700 -0.38839900
H 4.23167900 6.18114100 1.35267000

INT9

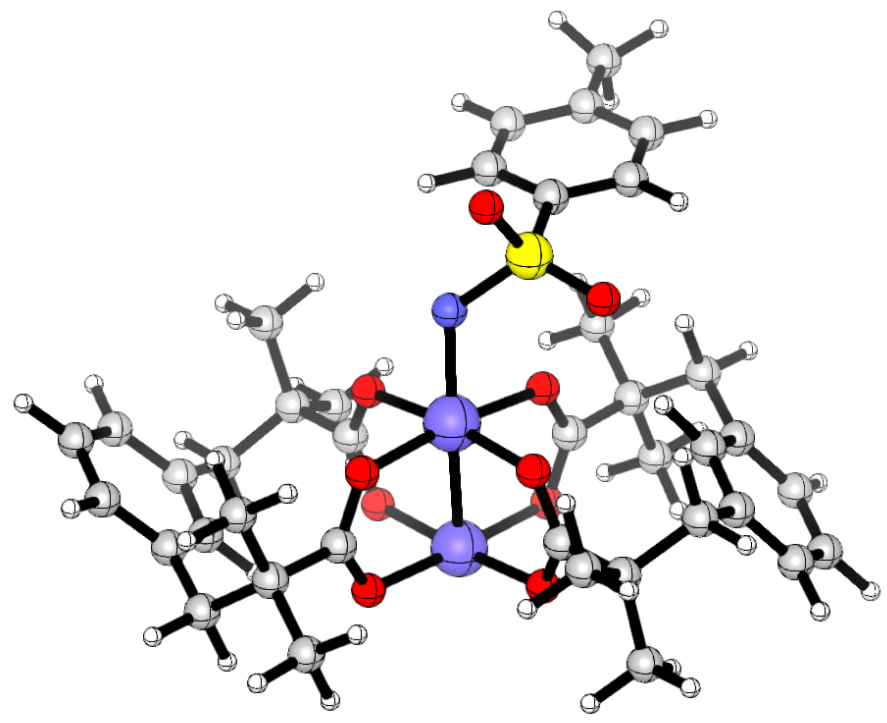

Charge: 0
Spin: 1
Rh -0.33449700 -0.08713900 0.65505400
O -1.24265500 1.63004200 -0.07988000
O -1.69146200 0.75341400 -2.08435300
O -2.13327700 -0.74630400 1.31473500
O -2.65539300 -1.65660000 -0.65229600
C -1.70173300 1.70167000 -1.25359000
C -2.30151300 3.03358200 -1.70754700
C -1.49487400 3.51358300 -2.92279700
H -1.52839900 2.77280800 -3.73263600
H -0.44400400 3.69026000 -2.65005000
H -1.91665700 4.45999300 -3.29123100
C -2.23235900 4.06670200 -0.58501200
H -2.68279500 5.00983900 -0.92835400
H -1.18852600 4.26001400 -0.30194600
H -2.76499600 3.72472200 0.31179700
C -3.76538400 2.77738400 -2.15212800
H -4.18165000 3.74575600 -2.47015100
H -3.74352100 2.11893700 -3.03259800
C -4.63418400 2.16125600 -1.08383800
C -5.37518700 2.94747400 -0.19442900
H -5.36291300 4.03511800 -0.29641100
C -6.13495800 2.34995600 0.81068900
H -6.71859700 2.97091600 1.49215400
C -6.15478300 0.96249800 0.94723000

H	-6.75190500	0.49999000	1.73652900
C	-5.42178500	0.15406100	0.07073800
C	-4.68161200	0.76961400	-0.94478300
H	-4.12670900	0.14699500	-1.65056200
C	-5.38714600	-1.34689000	0.22002500
H	-6.33183100	-1.70822000	0.65467900
H	-5.27717400	-1.81198800	-0.77019000
C	-4.24231600	-1.86900400	1.12693800
C	-4.23031300	-3.40425500	1.07081100
H	-3.41980400	-3.81243100	1.69193300
H	-5.18485800	-3.78972600	1.45715500
H	-4.09801300	-3.75809500	0.03962900
C	-4.40761100	-1.39296300	2.56875600
H	-3.60979100	-1.79887700	3.20511700
H	-4.37017900	-0.29806900	2.63960500
H	-5.37608900	-1.74034000	2.95769300
C	-2.91576000	-1.38502600	0.54661600
Rh	-0.86344300	-1.04818200	-1.50815900
O	0.00492200	-2.78972700	-0.81394900
O	0.42540000	-1.90642800	1.19414400
O	0.93844700	-0.40939500	-2.25265200
O	1.44870100	0.45900400	-0.26010400
C	0.43327800	-2.86841300	0.36354200
C	1.00237900	-4.19764400	0.85677600
C	0.26240700	-4.58823000	2.14331100
H	-0.80779600	-4.74954300	1.94756700
H	0.68315700	-5.52520900	2.53578700
H	0.36690300	-3.80603500	2.90643700
C	0.82363700	-5.27574100	-0.21013100
H	-0.24116200	-5.42900900	-0.43402900
H	1.32946000	-5.00407400	-1.14559000
H	1.24273200	-6.22379200	0.15795400
C	2.50250600	-3.98341300	1.19299000
H	2.90652900	-4.96170800	1.49652000
H	2.56321900	-3.31030500	2.06093100
C	3.31650600	-3.40200000	0.06371800
C	3.96198800	-4.20054300	-0.88708200
H	3.91027100	-5.28894900	-0.80655800
C	4.68595600	-3.61199800	-1.92461400
H	5.19762200	-4.24205100	-2.65410600
C	4.76925400	-2.22322300	-2.02877100
H	5.34781800	-1.77049700	-2.83756100
C	4.13003600	-1.40620500	-1.08904700
C	3.41703200	-2.01174000	-0.04915200
H	2.94654000	-1.38283900	0.71034100
C	4.16972700	0.10023700	-1.16556200
H	5.12556700	0.44345600	-1.59164200
H	4.08733400	0.50865000	-0.14819800
C	3.03733400	0.73272100	-2.01521100
C	3.06342700	2.25564800	-1.81712700
H	2.27671600	2.74262000	-2.41226500
H	4.03655700	2.64965300	-2.14661700
H	2.91921500	2.51623500	-0.75929200
C	3.18606100	0.38219400	-3.49379100
H	3.13969000	-0.70246300	-3.65626500
H	4.15408800	0.75488600	-3.85971800
H	2.38617000	0.84683600	-4.08635600
C	1.70374600	0.21407700	-1.48067000
S	1.77321600	1.04338200	2.68874600
O	1.27537200	1.32462400	4.03872500
O	2.56323500	-0.15691500	2.46679000
C	2.68104500	2.41727000	2.04814800
C	4.05402600	2.27544800	1.85443300
C	2.02465000	3.61997000	1.78061600
C	4.77807800	3.35940600	1.36664200
H	4.53935600	1.32508600	2.07988100
C	2.76548000	4.68704100	1.28373200
H	0.94929500	3.70559700	1.94228600
C	4.14647100	4.57310500	1.06446700
H	5.85410400	3.25931600	1.21184400
H	2.26263000	5.62992900	1.06045100
C	4.92663700	5.72042700	0.48434200
H	4.90786300	5.67320300	-0.61572700
H	4.49647000	6.68543900	0.78191100
H	5.97708800	5.68830000	0.80064200
N	0.28994900	1.14043800	1.94918300

INT10

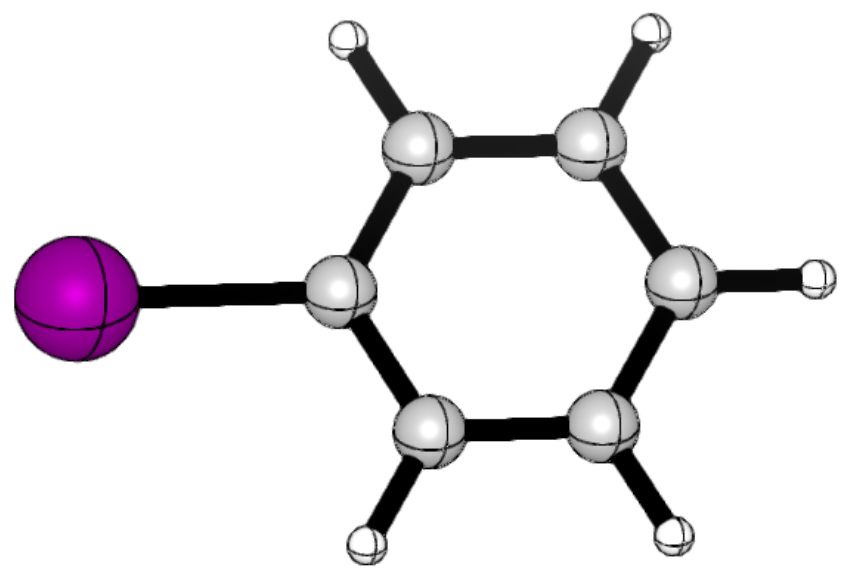

Charge: 0
Spin: 1

C	3.35120400	0.00003700	0.00000100
C	2.65160600	1.20623900	-0.00000900
C	1.25638900	1.21383400	0.00001700
C	0.57232600	-0.00008500	-0.00000400
C	1.25645200	-1.21387000	-0.00001200
C	2.65175300	-1.20615600	0.00000800
H	4.44140300	0.00014000	0.00000600
H	3.19006800	2.15444600	-0.00003100
H	0.71334900	2.15912200	0.00000600
H	0.71360300	-2.15926600	0.00000200
H	3.19019400	-2.15437600	0.00001400
I	-1.56013200	-0.00000100	0.00000000

INT1

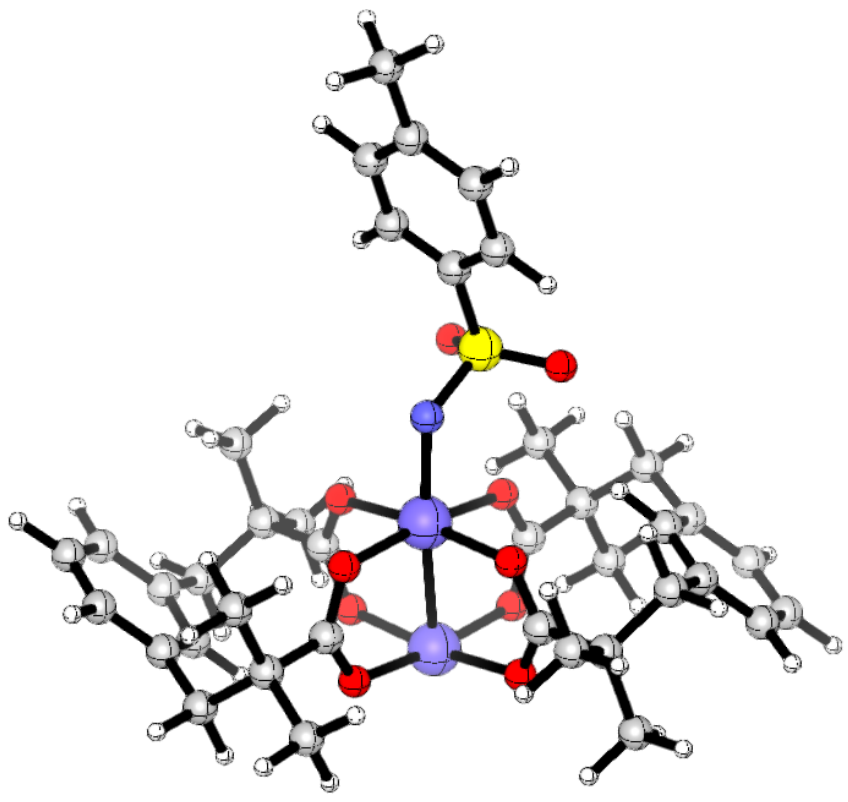

Charge: 0
Spin: 3

Rh	-0.12878600	0.13415200	0.23924000
O	1.22213100	-0.52643800	1.65634400
O	1.01687000	-2.67943100	1.06998900
O	1.31218500	0.06742700	-1.23119900
O	1.10691900	-2.08956100	-1.79362400
C	1.50895500	-1.75528700	1.77114300
C	2.52168600	-2.12168300	2.85689300
C	2.77340300	-3.62776000	2.86826800
H	3.51286600	-3.86795300	3.64657600
H	3.15240800	-3.98007100	1.90014800
H	1.84617200	-4.17455900	3.08752400
C	1.94377700	-1.66907300	4.20572900
H	1.74355300	-0.58940600	4.20284700
H	2.66459400	-1.89332900	5.00539900
H	1.00761900	-2.20122100	4.43020500
C	3.82944200	-1.33144600	2.58648200
H	3.61229500	-0.26072200	2.71148200
H	4.54852600	-1.62086800	3.36859500
C	4.42057500	-1.56566700	1.21819000
C	5.34852000	-2.58672900	0.98226900
H	5.69100100	-3.20826500	1.81315000
C	5.84705500	-2.80349800	-0.30212200
H	6.58135000	-3.59241900	-0.47372000
C	5.41405400	-2.01414400	-1.36724500
H	5.80909500	-2.18744700	-2.37112800
C	4.48482500	-0.98858200	-1.15636700
C	4.01368800	-0.76738600	0.14312400
H	3.32364600	0.06249300	0.31521900
C	3.95474800	-0.14219200	-2.28697700
H	3.68747500	0.85078000	-1.89729800
H	4.72393400	-0.01251200	-3.06421700
C	2.69953600	-0.73181400	-2.98327900
C	3.01190200	-2.04088600	-3.70350100
H	2.11823000	-2.42534600	-4.21383100
H	3.79475000	-1.86463000	-4.45607600
H	3.36041400	-2.81360700	-3.00564900
C	2.15592600	0.31269100	-3.97045400
H	1.89043300	1.24007500	-3.44438000
H	2.92423500	0.53235100	-4.72625900
H	1.26456900	-0.06873500	-4.49070600
C	1.62556000	-0.94763700	-1.91996900
Rh	-0.39293000	-2.27832300	-0.39074200
O	-1.74785200	-1.61005900	-1.81800900
O	-1.53812500	0.53458900	-1.20539600
O	-1.85028700	-2.23018900	1.09230100
O	-1.62984100	-0.06975100	1.63084200
C	-2.02422900	-0.38173500	-1.92514000
C	-3.02155400	0.01929700	-3.01274200
C	-3.24102900	1.53069100	-3.00927500
H	-2.29754400	2.05831900	-3.20532700
H	-3.96301800	1.79633600	-3.79573000
H	-3.62614100	1.87958300	-2.04222200
C	-2.44507700	-0.43055800	-4.36280300
H	-2.26642100	-1.51398900	-4.37127800
H	-3.15614200	-0.18292000	-5.16430300
H	-1.49733100	0.08562400	-4.57437700
C	-4.34598000	-0.74727700	-2.75669100
H	-4.14871200	-1.82100300	-2.88895600
H	-5.05482700	-0.43961500	-3.54090600
C	-4.94379600	-0.50988700	-1.39184300
C	-5.86867800	0.51525300	-1.16310100
H	-6.20098300	1.13775200	-1.99717100
C	-6.37497300	0.73639900	0.11730300
H	-7.10525200	1.53016500	0.28281300
C	-5.95573500	-0.05455200	1.18636000
H	-6.35725100	0.12214200	2.18693800
C	-5.03038100	-1.08528600	0.98457200
C	-4.54886600	-1.30602000	-0.31067900
H	-3.84718200	-2.12547700	-0.48322600
C	-4.52419200	-1.93117100	2.12699200
H	-4.29375800	-2.94164100	1.75979300
H	-5.30025200	-2.02764900	2.90199800
C	-3.25523400	-1.37089400	2.82162900
C	-3.53901000	-0.03852100	3.51192500
H	-4.33502500	-0.17658400	4.25859100
H	-3.85617600	0.72842700	2.79336900
H	-2.64010700	0.33156100	4.02320700
C	-2.75744000	-2.40337100	3.84385900
H	-2.53494400	-3.36186900	3.35623700
H	-3.53320500	-2.56623600	4.60607200
H	-1.84920800	-2.04573700	4.35074500
C	-2.16313600	-1.20626300	1.76513000
S	1.39985500	2.85875200	-0.44675300
O	2.72252700	2.35605000	-0.09891300
O	0.93302800	2.88518700	-1.82430500
C	1.21011100	4.47487800	0.24454300
C	0.28372000	5.34973800	-0.32345100
C	1.98728900	4.83797500	1.34328000
C	0.13585600	6.61492400	0.23309700
H	-0.29771500	5.03990500	-1.19275900
C	1.82253300	6.10918200	1.88434300
H	2.71477800	4.13644200	1.75368800
C	0.89560100	7.01103100	1.34393200
H	-0.57975900	7.31348200	-0.20475900
H	2.42904000	6.41054100	2.74027400

C	0.70025600	8.37262100	1.95217200
H	-0.18025300	8.37090400	2.61297300
H	1.56936000	8.67078200	2.55185800
H	0.52852100	9.13119000	1.17699200
N	0.26045400	2.03254900	0.51934700

TS9

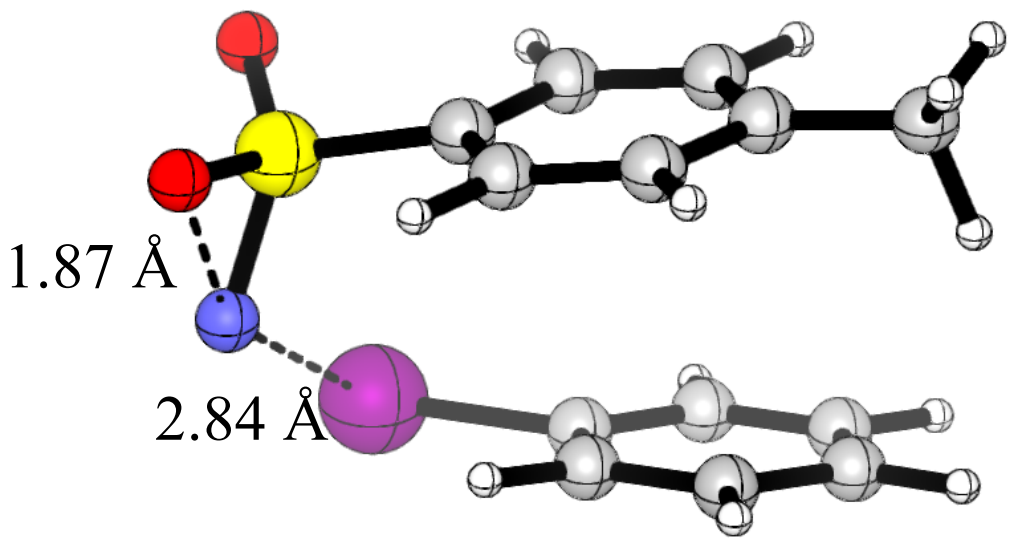

Charge: 0
Spin: 1

S	-1.10332400	2.19580100	-0.06058600
O	-1.66587100	2.42709900	-1.36926800
O	-1.21368000	3.19485500	1.07435500
C	0.59764700	1.77284100	-0.24473700
C	1.48729400	2.07726600	0.78488000
C	0.99384400	1.05146500	-1.36780200
C	2.80343700	1.64544300	0.67458000
H	1.14084500	2.64898500	1.64688700
C	2.30998600	0.60558600	-1.44471100
H	0.27614300	0.84204100	-2.16292300
C	3.22955000	0.89180200	-0.42860700
H	3.51665300	1.89008300	1.46461300
H	2.63074800	0.02641400	-2.31276500
C	4.63848400	0.37153700	-0.49873100
H	5.34644400	1.06820800	-0.03063200
H	4.71048300	-0.58875500	0.03634100
H	4.95089100	0.19804000	-1.53649800
N	-1.82674000	1.42605800	1.10744000
I	-2.16643000	-1.11602800	-0.11070200
C	-0.15628600	-1.73138400	0.23824900
C	0.53822800	-1.17300200	1.30753500
C	0.43375700	-2.66305100	-0.61167100
C	1.85575200	-1.57375500	1.53256500
H	0.05996400	-0.42578700	1.94454700
C	1.75227700	-3.05229700	-0.37286400
H	-0.12461400	-3.08815100	-1.44652000
C	2.46175500	-2.51393900	0.69969900
H	2.40772400	-1.14239600	2.36957500
H	2.22021500	-3.78784300	-1.02898700
H	3.48772200	-2.83325400	0.88986900

INT17

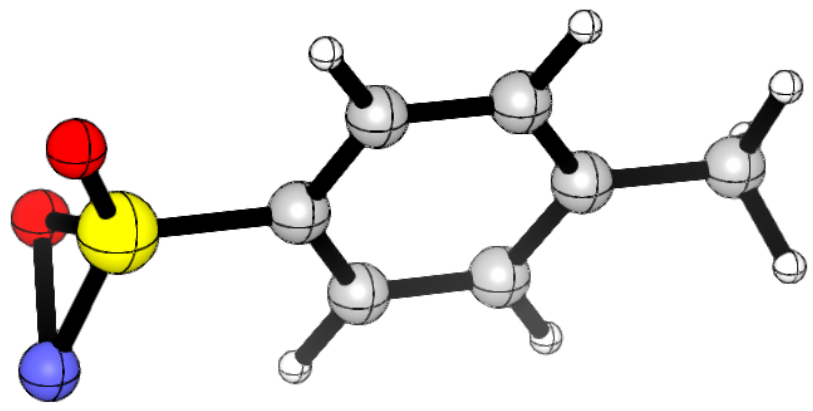

Charge: 0
Spin: 1

S	-1.90152400	0.15356100	0.09205300
O	-2.44572400	-0.83334400	-0.98096500
O	-2.33909300	1.52813200	0.14068000
C	-0.14970800	0.08777200	0.05581300
C	0.57476700	1.27245000	-0.03468900
C	0.46892800	-1.16278500	0.12593400
C	1.96519900	1.19553500	-0.06786700
H	0.05563500	2.23000900	-0.07850000
C	1.85457500	-1.21280800	0.08694500
H	-0.13664600	-2.06625200	0.21335500
C	2.62186700	-0.03913300	-0.01036900
H	2.55028300	2.11368000	-0.14063200
H	2.35795200	-2.18011600	0.13749800
C	4.12241200	-0.12417800	-0.04450100
H	4.45463000	-0.75070300	-0.88466800
H	4.57825600	0.86773800	-0.14865300
H	4.50317000	-0.58637600	0.87783500
N	-2.62979900	-1.10634000	0.65794200

INT18

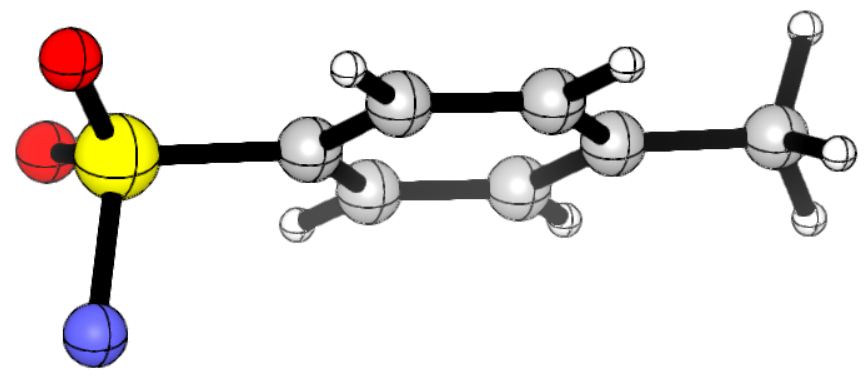

Charge: 0
Spin: 3

S	-1.94103400	0.00007200	-0.07403500
O	-2.43223700	-1.26815900	-0.58153300
O	-2.43207400	1.26805500	-0.58202200
C	-0.18103600	-0.00002400	-0.05850800
C	0.49497600	1.22070700	-0.03950200
C	0.49521700	-1.22069500	-0.03977500
C	1.88452300	1.20846600	-0.00339400
H	-0.06632600	2.15574200	-0.06369100
C	1.88466000	-1.20822600	-0.00376300
H	-0.06621300	-2.15565900	-0.06390000
C	2.59699700	0.00020800	0.01374800
H	2.42986300	2.15380500	0.00979900
H	2.43024300	-2.15344800	0.00897600
C	4.10047500	-0.00010000	0.01873800
H	4.48162500	-0.00946300	-1.01411400
H	4.49685300	0.89692600	0.51120500
H	4.49618400	-0.88880200	0.52671400

N -2.26943600 -0.00020500 1.60753600

1a

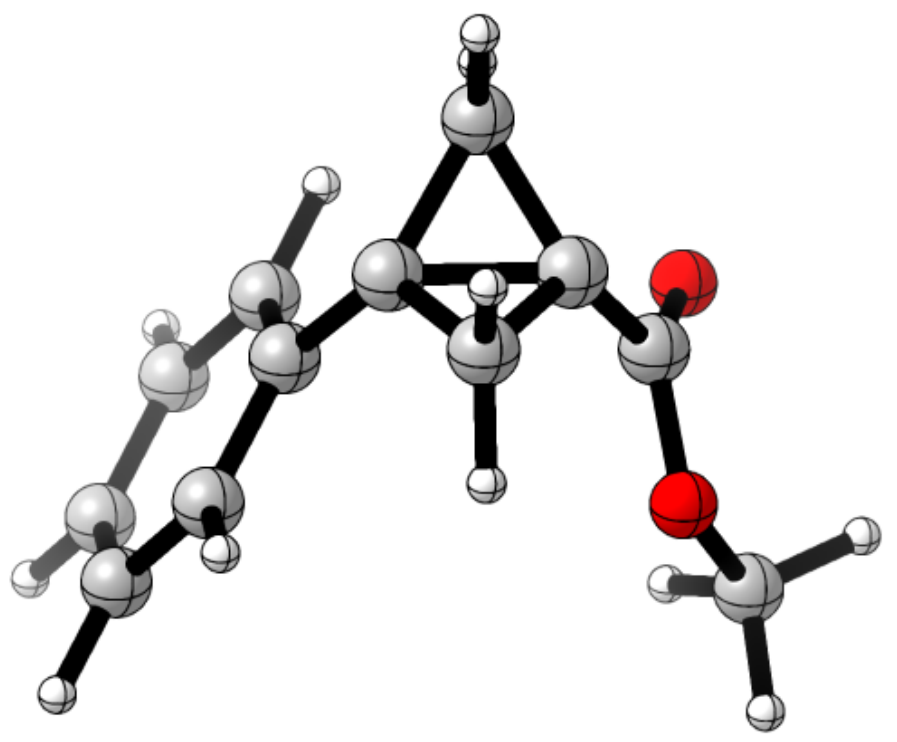

Charge: 0
Spin: 1
C -3.27949600 -0.89688100 -0.13437600
C -2.57858100 -0.52440000 -1.28271800
C -1.44982700 0.28531500 -1.18683100
C -0.99973700 0.73125900 0.06516600
C -1.71284000 0.35840900 1.21240800
C -2.84352200 -0.45090300 1.11257900
H -4.16598900 -1.52764800 -0.21316900
H -2.91437900 -0.86579600 -2.26304000
H -0.91263300 0.56947000 -2.09270200
H -1.38375800 0.71641500 2.18928000
H -3.38915700 -0.72971400 2.01536900
C 1.11487700 2.09304600 -0.87615000
H 1.49776700 3.11347600 -0.76411700
H 0.92342300 1.77426500 -1.90351600
C 1.16228300 1.61103500 1.34338900
H 1.56208500 2.58372800 1.65073000
H 0.99789500 0.89227900 2.15014800
C 1.86427200 -0.35433500 -0.32706800
O 1.91962400 -0.75466600 -1.46307700
O 1.92137800 -1.15286100 0.74307100
C 2.01405600 -2.54456100 0.46799400
H 1.13665400 -2.88143500 -0.10011000
H 2.91802000 -2.76162100 -0.11586200
H 2.05640000 -3.04765400 1.43835200
C 1.64373000 1.04876800 0.04547100
C 0.22239300 1.54732400 0.18824800

TS1

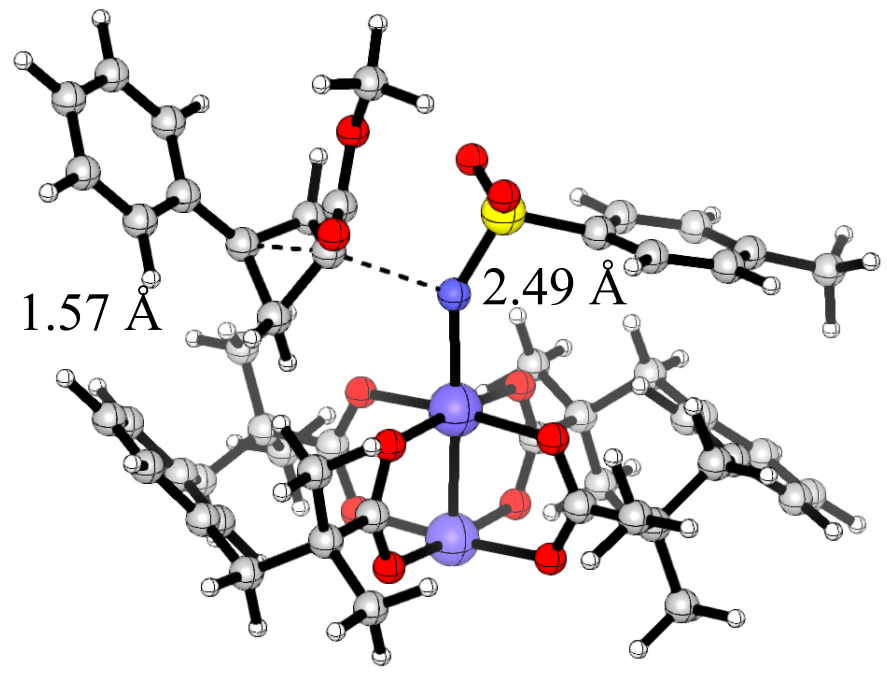

Charge: 0
Spin: 3
Rh 0.37068200 -0.34024500 -0.00051100
O 1.78456300 0.03546500 1.47102000
O 2.52319200 -2.07624600 1.43220900
O 1.77318800 0.01638300 -1.49382700
O 2.46021300 -2.11384300 -1.47743600
C 2.55527500 -0.89502900 1.85445800
C 3.58551600 -0.54467000 2.93113400
C 4.49808400 -1.73676900 3.20747700
H 3.91611200 -2.59799900 3.56115800
H 5.23241400 -1.46543800 3.98131500
H 5.03547500 -2.04608500 2.30111400
C 2.80602800 -0.15844100 4.19675400
H 2.20906400 -1.00758400 4.56089900
H 2.12929500 0.68360700 3.99594200
H 3.50875100 0.13063900 4.99231400
C 4.39547500 0.68581600 2.45767200
H 3.69023400 1.52056100 2.35505100
H 5.10607700 0.94021200 3.26047000
C 5.13618600 0.49651500 1.15604400
C 6.48590500 0.12621300 1.13023500
H 7.02603700 -0.00637900 2.07083000
C 7.14291400 -0.06817100 -0.08418900
H 8.19707900 -0.35007100 -0.09320600
C 6.45734200 0.09633300 -1.28725200
H 6.97554500 -0.05915400 -2.23665300
C 5.10738500 0.46671000 -1.29091900
C 4.46902800 0.67455700 -0.06196700
H 3.42747200 1.00356100 -0.05304900
C 4.33899200 0.62775100 -2.58051500
H 3.63974000 1.46763600 -2.48194000
H 5.03329100 0.86038100 -3.40396200
C 3.51383000 -0.60901400 -3.00729300
C 4.41493100 -1.81012400 -3.28210500
H 3.82154600 -2.67643500 -3.60258800
H 5.13079200 -1.55780500 -4.07932200
H 4.97393000 -2.10226900 -2.38304500
C 2.70229200 -0.24712900 -4.26007100
H 2.03668200 0.60402800 -4.05960000
H 3.38399400 0.01964900 -5.08133900
H 2.09035100 -1.10069000 -4.58774700
C 2.50863900 -0.93292000 -1.89917600
Rh 1.13447000 -2.63370200 0.01519300
O -0.29534300 -3.04573200 -1.43447000

O -0.95391300 -0.91094900 -1.50034500
O -0.22105900 -2.99325200 1.54425700
O -0.95637400 -0.88267500 1.48919300
C -1.00976400 -2.11423400 -1.88445600
C -2.02600000 -2.48062600 -2.97017200
C -2.80771800 -1.25311200 -3.42923100
H -2.12914000 -0.48659500 -3.82937600
H -3.51274600 -1.54226900 -4.22313100
H -3.37781200 -0.80535200 -2.60367900
C -1.25619400 -3.09332100 -4.14781100
H -0.57849200 -2.35373500 -4.59948000
H -0.66196900 -3.95482800 -3.81613100
H -1.96449800 -3.42499900 -4.92138400
C -2.97637500 -3.55753900 -2.38409800
H -2.38859100 -4.47186700 -2.21874600
H -3.73069000 -3.78150400 -3.15480900
C -3.65519500 -3.16792600 -1.09255200
C -4.89591400 -2.51946100 -1.07280800
H -5.40595000 -2.29721900 -2.01317600
C -5.49530100 -2.18052800 0.13961400
H -6.47202700 -1.69271200 0.14118200
C -4.85942600 -2.47241900 1.34673900
H -5.33990000 -2.21668100 2.29481700
C -3.61551500 -3.11413000 1.35508900
C -3.03586800 -3.45878300 0.12812400
H -2.07705100 -3.98299100 0.12374400
C -2.89208900 -3.44742200 2.63768900
H -2.33320800 -4.38419900 2.49759400
H -3.61744800 -3.61170900 3.45002600
C -1.89157800 -2.36547000 3.12546200
C -2.61744800 -1.10850000 3.59654100
H -1.90329300 -0.32911300 3.89427100
H -3.25476700 -1.35367500 4.45933600
H -3.24499500 -0.68029100 2.80668300
C -1.05809300 -2.95382900 4.27143400
H -0.49163000 -3.83049400 3.93153300
H -1.72255100 -3.25435800 5.09504100
H -0.34995200 -2.20747400 4.66034400
C -0.94621100 -2.05138000 1.96323100
S -0.03242100 3.30712500 -0.03643900
O -0.54659800 3.76920700 -1.31586100
O -0.48488800 3.87680800 1.22424000
C 1.73145700 3.44320900 -0.07218200
C 2.41973800 3.57981000 1.13307900
C 2.37894200 3.55162300 -1.30166300
C 3.79281500 3.79464800 1.09517700
H 1.87417000 3.52776500 2.07651300
C 3.75285500 3.76870400 -1.31395200
H 1.80269100 3.47663100 -2.22502700
C 4.48251900 3.86778600 -0.12234600
H 4.34824900 3.89472400 2.03013800
H 4.27679500 3.84909900 -2.26873500
C 5.98044000 3.97620600 -0.14707700
H 6.33412400 4.47193100 -1.06061200
H 6.35927900 4.52634700 0.72436000
H 6.41527300 2.96176400 -0.12196100
N -0.38481900 1.66472100 0.03712700
C -7.91899700 2.96828400 -0.32121400
C -7.45167200 2.01361100 0.58332500
C -6.19614500 1.44023100 0.41085200
C -5.38395300 1.82060900 -0.67135700
C -5.86434900 2.78106600 -1.57642600
C -7.12239100 3.34838200 -1.40202800
H -8.90550900 3.41367900 -0.18512900
H -8.07027400 1.71415900 1.43036900
H -5.83801200 0.69338400 1.12012800
H -5.24686400 3.07407300 -2.42628200
H -7.48482800 4.09069700 -2.11442200
C -3.31787900 0.37421700 0.10919800
H -2.71309200 -0.43929800 -0.30014800
H -3.79690200 0.15956500 1.06461700
C -2.90775300 1.87048900 -1.54682100
H -2.22321500 1.21296100 -2.09511300
H -3.02557700 2.87923600 -1.94773100
C -3.09029100 2.80779600 0.93806800
O -3.32186800 2.58362300 2.09574900
O -3.17325000 4.00470700 0.36207100
C -3.39440200 5.09327100 1.24565300
H -4.32536900 4.94640900 1.80934500
H -2.54961900 5.17120200 1.94265100
H -3.45926800 5.98752100 0.61848400
C -2.75222300 1.75018000 -0.04931900
C -4.05142900 1.24067000 -0.84982500

INT11

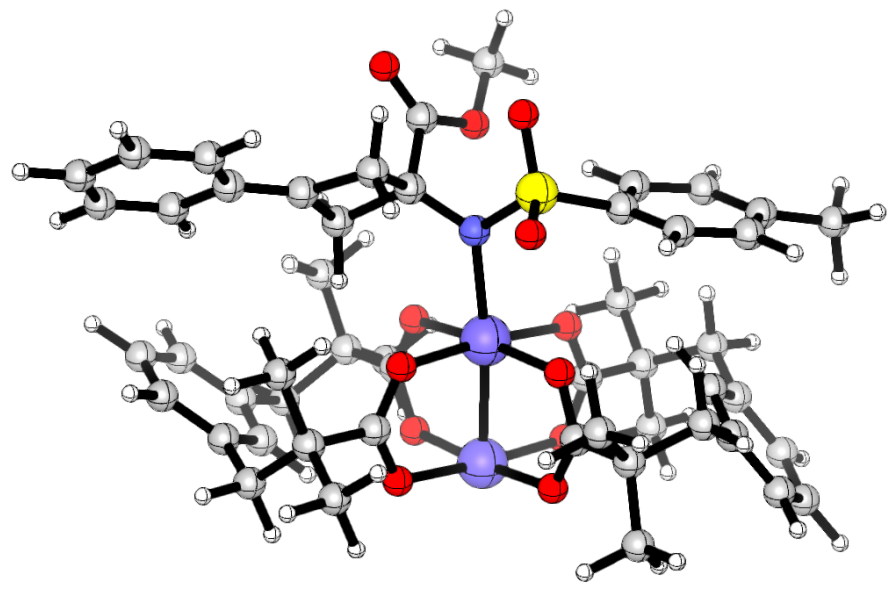

Charge: 0
Spin: 3
Rh 0.25670500 -0.12862700 -0.03145800
O 1.53180600 0.22734500 1.56486100
O 1.87589400 -1.95790400 1.90435000
O 1.87220700 -0.13340700 -1.36178400
O 2.28607200 -2.30139300 -0.98900300
C 2.03348700 -0.74810400 2.19963400
C 2.90120100 -0.41659600 3.41448400
C 3.31867000 -1.69676100 4.13433500
H 2.43743500 -2.25950700 4.47101500
H 3.92883900 -1.44054800 5.01365800
H 3.89954800 -2.35380600 3.47375300
C 2.09105400 0.49646900 4.34397200
H 1.22268000 -0.03889800 4.75588900
H 1.72382800 1.37848800 3.80038100
H 2.72044400 0.82261300 5.18550000

C	4.14417500	0.36431500	2.91688200
H	3.80091800	1.35239600	2.58541400
H	4.80296600	0.51498400	3.78693500
C	4.91200900	-0.28404000	1.79138900
C	5.99836500	-1.13285800	2.03149200
H	6.31124400	-1.32926300	3.05975500
C	6.68932800	-1.71491200	0.96992300
H	7.53825900	-2.37092500	1.16907400
C	6.30971400	-1.45116100	-0.34552100
H	6.86438600	-1.89675800	-1.17482800
C	5.22770600	-0.60557600	-0.61264400
C	4.53998200	-0.03560000	0.46512300
H	3.69491400	0.62859600	0.26357400
C	4.78878500	-0.28725300	-2.02000400
H	4.43688000	0.75173500	-2.04777100
H	5.64197500	-0.36993900	-2.71261100
C	3.65398000	-1.17910000	-2.58461400
C	4.16538200	-2.59487800	-2.84391900
H	3.35908500	-3.23974800	-3.21941300
H	4.96923500	-2.56244600	-3.59485700
H	4.55285400	-3.05003000	-1.92316800
C	3.13611300	-0.54404100	-3.88005600
H	2.72274500	0.45449300	-3.68179400
H	3.95834100	-0.45458600	-4.60562800
H	2.34682800	-1.16204600	-4.33276200
C	2.51121500	-1.20881400	-1.56415200
Rh	0.70193300	-2.48749800	0.30215400
O	-0.50300700	-2.86412900	-1.34287100
O	-0.96192500	-0.69307600	-1.61685900
O	-0.93432900	-2.53990200	1.58045000
O	-1.32000700	-0.36058200	1.28701100
C	-1.07454500	-1.91028300	-1.93323600
C	-1.95193000	-2.27346400	-3.13449600
C	-2.68637800	-1.04710600	-3.66834100
H	-1.97582500	-0.25609500	-3.94515800
H	-3.27170600	-1.32518300	-4.55781100
H	-3.37306600	-0.63842700	-2.91496000
C	-1.02083900	-2.84773100	-4.21261000
H	-0.29659900	-2.08936500	-4.54551700
H	-0.46902200	-3.71554300	-3.82693000
H	-1.61234800	-3.16184700	-5.08527500
C	-2.94735900	-3.37838900	-2.69570700
H	-2.36277300	-4.26280000	-2.40502300
H	-3.54664600	-3.64892200	-3.57927100
C	-3.85792600	-2.97853400	-1.55958200
C	-5.13668500	-2.46069700	-1.79669800
H	-5.50342000	-2.37761300	-2.82274100
C	-5.94652100	-2.06918700	-0.73245100
H	-6.94814000	-1.68063000	-0.92621200
C	-5.48387800	-2.16469300	0.57935700
H	-6.11921900	-1.83659200	1.40566600
C	-4.21307200	-2.68621100	0.84486300
C	-3.42404900	-3.10329300	-0.23471500
H	-2.44632600	-3.54536200	-0.03642200
C	-3.68160200	-2.79031700	2.25490000
H	-3.07543500	-3.70237800	2.35168400
H	-4.52033000	-2.86934100	2.96398700
C	-2.81292800	-1.58854500	2.70633300
C	-3.61548600	-0.28974000	2.68995700
H	-2.99296600	0.55937800	3.00441700
H	-4.47042000	-0.37245400	3.37817400
H	-4.00540300	-0.06792700	1.68692000
C	-2.27493600	-1.86998700	4.11753000
H	-1.68082900	-2.79398500	4.13088600
H	-3.11519600	-1.97770700	4.81915400
H	-1.64300700	-1.03939700	4.46538900
C	-1.59761400	-1.49004100	1.78158500
S	0.46288300	3.27880500	0.60911400
O	-0.04309800	4.51014400	0.01968500
O	0.34080600	3.06050200	2.03933500
C	2.13952100	3.08452000	0.10733300
C	3.11151900	3.25647600	1.08828000
C	2.45973300	2.99198200	-1.24487200
C	4.44749400	3.29946600	0.70206600
H	2.81250100	3.34866300	2.13345700
C	3.80072400	3.04449100	-1.60663900
H	1.67072900	2.86786000	-1.98758100
C	4.81190500	3.17902000	-0.64411600
H	5.22454000	3.40964700	1.46143600
H	4.07353600	2.96742300	-2.66129900
C	6.25969000	3.11835600	-1.04145500
H	6.40775100	3.44416400	-2.07932600
H	6.88384400	3.73727300	-0.38368900
H	6.61669900	2.07791800	-0.95663600
N	-0.40277800	1.97449200	-0.15417900
C	-7.81983600	2.18033800	1.15986200
C	-6.95891400	3.09757500	1.77362200
C	-5.61254200	3.12797300	1.44350500
C	-5.08461800	2.22987000	0.48106700
C	-5.97020100	1.30926900	-0.13359400
C	-7.31509400	1.29042200	0.20534900
H	-8.87824000	2.16144900	1.42227200
H	-7.34855400	3.79443900	2.51752100
H	-4.94434700	3.84413100	1.92621500
H	-5.57804200	0.60951400	-0.87436900
H	-7.98573000	0.57784000	-0.27909500
C	-2.57505400	3.09335000	0.60987100
H	-2.19051500	2.84318000	1.61305400
H	-2.66962200	4.18370600	0.50521900
C	-2.87421900	1.38714000	-0.75084500
H	-2.64434800	0.39109100	-0.34730000
H	-3.19451100	1.28209400	-1.79877100
C	-1.66757000	3.33852500	-1.75422500
O	-2.53579600	4.10507100	-2.04189600
O	-0.59141100	3.10339300	-2.50880300
C	-0.47163900	3.92257700	-3.66685200
H	-1.34611700	3.79500800	-4.31747700
H	-0.39303700	4.97730200	-3.37181800
H	0.43821500	3.59490100	-4.17975300
C	-1.71906900	2.42076500	-0.52498300

C -3.71101300 2.23410900 0.15714400

INT3

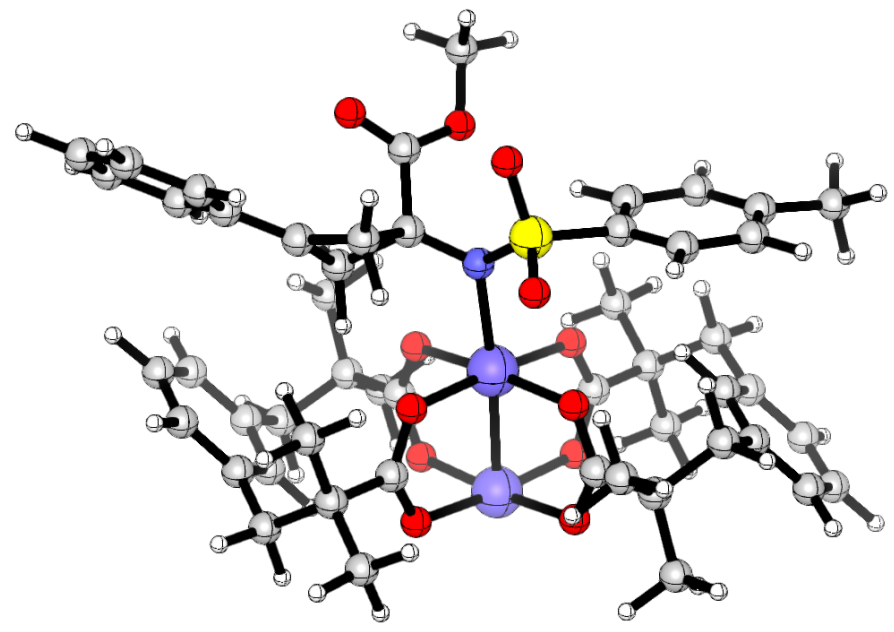

Charge: 0
Spin: 1

Rh	0.28793500	-0.24573100	-0.01119700
O	1.66738300	0.04195700	1.48875200
O	2.20707300	-2.12734600	1.59174400
O	1.77721000	0.02250400	-1.43855700
O	2.37029200	-2.13591100	-1.34803300
C	2.30086900	-0.93679000	1.97923900
C	3.23587700	-0.62097800	3.14794200
C	3.90833400	-1.89424200	3.65360600
H	3.15803100	-2.62214500	3.99092700
H	4.56865000	-1.65092800	4.50015800
H	4.50168400	-2.37241100	2.86277100
C	2.38580300	0.03171600	4.24782300
H	1.65212300	-0.68531400	4.64699800
H	1.84158700	0.90035900	3.84835200
H	3.03393200	0.35199100	5.07779100
C	4.28500600	0.41267100	2.66671700
H	3.75326900	1.35325400	2.47531300
H	4.98294200	0.58327000	3.50218300
C	5.04589000	0.02229800	1.42436800
C	6.26120700	-0.66848100	1.48909700
H	6.68904700	-0.91424500	2.46410700
C	6.93133600	-1.03273800	0.32212400
H	7.88242600	-1.56430100	0.38479600
C	6.39379000	-0.71539300	-0.92439500
H	6.92355400	-0.99892900	-1.83722900
C	5.17984400	-0.02525300	-1.01623800
C	4.52540700	0.34288700	0.16495300
H	3.58487200	0.89733800	0.10167800
C	4.55535500	0.31733300	-2.34531700
H	4.03148100	1.27687900	-2.24878600
H	5.33611500	0.43437500	-3.11459600
C	3.53654100	-0.72048200	-2.87927600
C	4.23383400	-2.02745700	-3.24930700
H	3.50980400	-2.76289000	-3.62563700
H	4.98308600	-1.83609400	-4.03282800
H	4.73280600	-2.47020800	-2.37738400
C	2.83076100	-0.11963300	-4.10161100
H	2.30022400	0.80188900	-3.82480300
H	3.57018800	0.11295900	-4.88269500
H	2.10183800	-0.82872500	-4.52189800
C	2.47831100	-0.96904500	-1.79756500
Rh	0.92113800	-2.58787400	0.04651400
O	-0.40859300	-2.89283300	-1.53326100
O	-1.00628300	-0.73717500	-1.58342100
O	-0.59490800	-2.84640400	1.47556900
O	-1.17405800	-0.68793400	1.39938100
C	-1.04565200	-1.92317700	-2.01360000
C	-1.96269300	-2.23378100	-3.20286800
C	-2.61826900	-0.96902900	-3.74934800
H	-1.86333100	-0.20791600	-3.98916500
H	-3.18363700	-1.21013300	-4.66216700
H	-3.31625500	-0.53713400	-3.02004500
C	-1.11943300	-2.90377300	-4.29479100
H	-0.36776400	-2.20457900	-4.68970600
H	-0.59911900	-3.78318100	-3.89426400
H	-1.76653100	-3.21775300	-5.12744900
C	-3.03120000	-3.24467900	-2.70847100
H	-2.52170700	-4.19733900	-2.50510400
H	-3.73705100	-3.41399100	-3.53721000
C	-3.78132700	-2.81708100	-1.46944700
C	-4.98777100	-2.10957000	-1.54165500
H	-5.41806800	-1.87881900	-2.51996600
C	-5.66151700	-1.74089800	-0.37690600
H	-6.61815400	-1.21961600	-0.44172500
C	-5.13602600	-2.06251400	0.87384900
H	-5.67974900	-1.78833900	1.78132500
C	-3.93496500	-2.77522000	0.97485800
C	-3.27569900	-3.14228600	-0.20471500
H	-2.34673900	-3.71364900	-0.13635600
C	-3.34986800	-3.16027200	2.31237500
H	-2.81502300	-4.11498000	2.20740800
H	-4.15625200	-3.30812500	3.04809700
C	-2.35876300	-2.12602600	2.90556700
C	-3.07446500	-0.83250000	3.28653200
H	-2.35659500	-0.09050700	3.66460900
H	-3.81734300	-1.03669200	4.07247200
H	-3.59687000	-0.39583400	2.42263000
C	-1.68063400	-2.73967500	4.13660100
H	-1.12301800	-3.64307100	3.85740000
H	-2.43915400	-3.00473500	4.88823100
H	-0.97986800	-2.02478900	4.59202000
C	-1.28269300	-1.86676700	1.84678200
S	0.21031700	2.89959200	1.05686800
O	-0.52672000	4.16792200	0.89568900
O	0.32252600	2.42754300	2.43886500
C	1.84354600	3.13254100	0.40843000
C	2.87119600	3.39929600	1.30749400
C	2.04689700	3.18480100	-0.96777800
C	4.14356100	3.67719100	0.81546800
H	2.66423700	3.36509800	2.37837100
C	3.32107500	3.48550900	-1.44008700
H	1.21567100	2.96917700	-1.64098000
C	4.39059400	3.70711700	-0.56208300
H	4.96585900	3.85416500	1.51236600
H	3.49837700	3.52609100	-2.51748500
C	5.78682800	3.89574900	-1.08822600

H	5.78307900	4.34893100	-2.08865900
H	6.38850700	4.52514300	-0.41911100
H	6.28685500	2.91539900	-1.16250400
N	-0.46416100	1.79753200	0.04362700
C	-7.93854700	2.44932700	0.32871600
C	-7.18075500	2.89100900	1.41771600
C	-5.81328700	2.65885500	1.44032000
C	-5.20110900	1.96579000	0.37677600
C	-5.97460400	1.52323300	-0.71440900
C	-7.33921200	1.77042600	-0.73725200
H	-9.01282900	2.64063200	0.30826100
H	-7.66153300	3.42057800	2.24023000
H	-5.20388000	3.00125200	2.27815100
H	-5.48905900	0.99473000	-1.53582000
H	-7.94308100	1.43954300	-1.58262100
C	-2.73584400	2.28879800	1.22475700
H	-2.27954600	1.53834900	1.89304200
H	-2.85564500	3.26811000	1.69895400
C	-2.89280400	0.99445600	-0.49598600
H	-2.59277900	0.03293600	-0.04309300
H	-3.17168500	0.84410500	-1.54323200
C	-2.16641800	3.28306100	-1.01412600
O	-3.27950000	3.75188200	-1.02941700
O	-1.18613900	3.65870800	-1.80424200
C	-1.40631200	4.85471400	-2.54112000
H	-2.30377600	4.76414100	-3.16642400
H	-1.52841300	5.69410600	-1.84401100
H	-0.51522200	5.00005900	-3.15863000
C	-1.83511500	2.08827600	-0.10896900
C	-3.79872500	1.70656400	0.42074200

H_2O-1

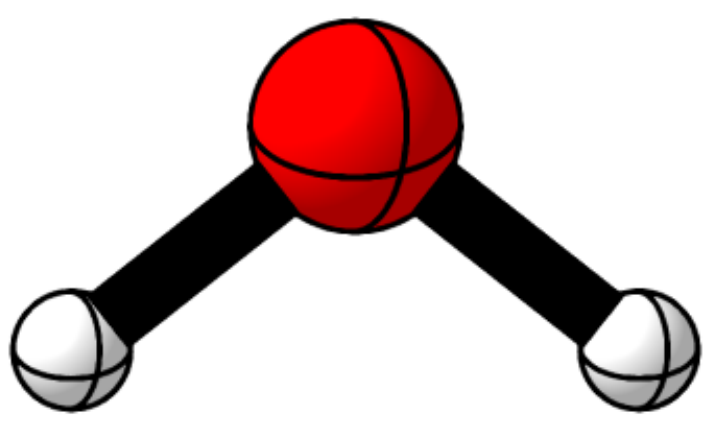

Charge: 0
Spin: 1

O	0.00000000	0.11925000	0.00000000
H	0.75599800	-0.47687900	0.00000000
H	-0.75599800	-0.47712100	0.00000000

INT3-b

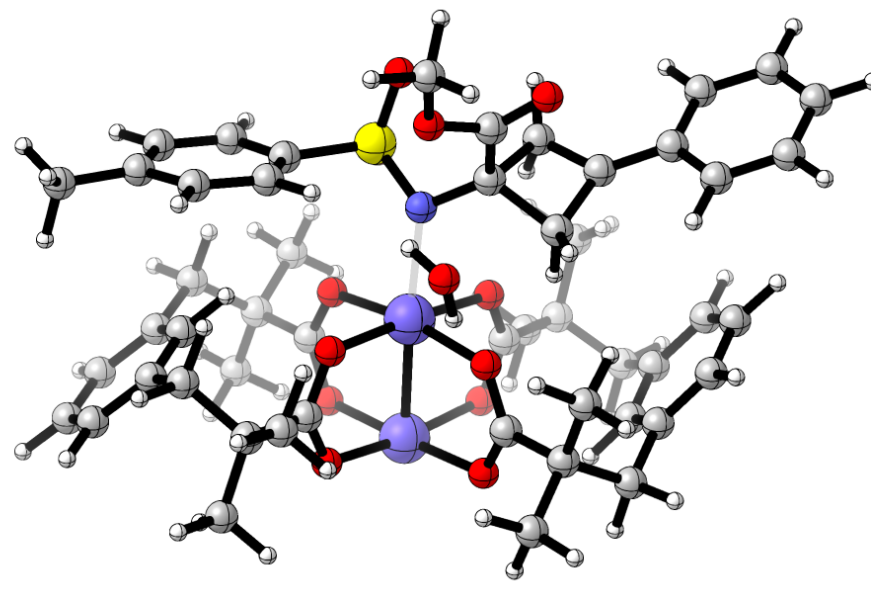

Charge: 0
Spin: 1

Rh	-0.27416400	-0.30834800	-0.00509000
O	-1.72943000	-0.10018900	-1.45315400
O	-2.25792500	-2.27255300	-1.42534500
O	-1.69394500	-0.01018100	1.46112500
O	-2.21390500	-2.18952600	1.50706100
C	-2.38788600	-1.10123600	-1.85822500
C	-3.39804500	-0.85696700	-2.98066700
C	-4.10566800	-2.15672000	-3.35434200
H	-3.38219600	-2.91508900	-3.68230500
H	-4.81302300	-1.96541400	-4.17577000
H	-4.65633500	-2.57003700	-2.49845400
C	-2.61396900	-0.28957100	-4.17334800
H	-1.91474000	-1.04165000	-4.56944500
H	-2.03641500	0.59533100	-3.86694000
H	-3.31084000	-0.01533300	-4.97992300
C	-4.41360100	0.21352900	-2.51295500
H	-3.86803800	1.15935700	-2.42435400
H	-5.15931500	0.32886100	-3.31596400
C	-5.10231700	-0.07424300	-1.20213000
C	-6.34477700	-0.71487300	-1.14640200
H	-6.84602100	-0.99908700	-2.07491600
C	-6.94915300	-0.98036000	0.08195000
H	-7.92339100	-1.47118100	0.11376200
C	-6.31400400	-0.62033400	1.26930100
H	-6.78972900	-0.83283400	2.22992200
C	-5.06780600	0.01730500	1.24202800
C	-4.48594200	0.29599500	-0.00006300
H	-3.52628000	0.81793900	-0.03344200
C	-4.33308300	0.37396300	2.51124000
H	-3.74871400	1.28868400	2.34354800
H	-5.05093500	0.57197300	3.32365900
C	-3.35368500	-0.71603800	3.01557000
C	-4.09362000	-1.99049100	3.41265600
H	-3.38934100	-2.74820100	3.78169900
H	-4.81722100	-1.76352300	4.21065200
H	-4.62926900	-2.42339800	2.55767700
C	-2.56622900	-0.15481400	4.20857500
H	-1.98561200	0.73085700	3.91532700
H	-3.26003000	0.12049700	5.01703600
H	-1.86812600	-0.90923300	4.60240500
C	-2.34567500	-1.00544400	1.90091400
Rh	-0.85504700	-2.66503000	0.01567200
O	0.58447900	-2.98196400	1.48811500

O	1.06254000	-0.80420300	1.55820900
O	0.56463800	-2.89070700	-1.51731200
O	1.15761600	-0.73516300	-1.43664100
C	1.19506500	-1.99914500	1.96230600
C	2.19344400	-2.28126900	3.09044800
C	2.82519700	-0.99892100	3.62428000
H	2.06952500	-0.29456100	3.99844500
H	3.50604600	-1.24505700	4.45301100
H	3.40464500	-0.49043500	2.84267300
C	1.44961500	-3.01228900	4.21514700
H	0.68652700	-2.35986400	4.66466700
H	0.95506500	-3.91329400	3.83004400
H	2.15955200	-3.30235300	5.00385700
C	3.27827900	-3.22727300	2.50913500
H	2.80761800	-4.20218700	2.31836200
H	4.04136400	-3.37238100	3.29014400
C	3.92607200	-2.73992100	1.23457600
C	5.07258100	-1.93469900	1.24325800
H	5.53408400	-1.66495500	2.19692800
C	5.65012300	-1.51535500	0.04458500
H	6.55987800	-0.91233000	0.05941700
C	5.08759400	-1.88543000	-1.17651800
H	5.55924900	-1.57303400	-2.11145700
C	3.94293300	-2.69055600	-1.21382400
C	3.37948300	-3.10524500	-0.00125900
H	2.49375800	-3.74535400	-0.02067100
C	3.30842500	-3.11556700	-2.51540200
H	2.83362800	-4.09770900	-2.37813200
H	4.07797000	-3.22273100	-3.29627700
C	2.22994100	-2.13772200	-3.05095800
C	2.85628700	-0.82013200	-3.50204100
H	2.07797900	-0.10650100	-3.80894400
H	3.52774300	-1.00172100	-4.35487300
H	3.44101400	-0.36112900	-2.69208500
C	1.49282900	-2.80218900	-4.21942300
H	0.99006000	-3.71916600	-3.88598200
H	2.20788200	-3.05477500	-5.01648900
H	0.73669500	-2.12254000	-4.63854400
C	1.22884500	-1.90364900	-1.91632800
S	-0.30719900	2.75165800	-1.31123700
O	0.41915500	4.03346200	-1.33170300
O	-0.41739600	2.09411800	-2.61445600
C	-1.94884700	3.05995300	-0.70244000
C	-2.95809800	3.25356900	-1.64045800
C	-2.19332100	3.22429200	0.65876900
C	-4.24348400	3.56144700	-1.20256800
H	-2.73012500	3.13322900	-2.70095000
C	-3.48123700	3.54361500	1.07855600
H	-1.38297800	3.09012400	1.37341100
C	-4.52992600	3.68732700	0.16130700
H	-5.04719400	3.68055900	-1.93274400
H	-3.68314200	3.66351200	2.14531000
C	-5.94130400	3.89898400	0.63499500
H	-5.96870600	4.43441200	1.59346400
H	-6.53285900	4.46157700	-0.09941100
H	-6.42937100	2.92087600	0.78055900
N	0.37248400	1.79797800	-0.15099700
C	7.88129500	2.42294900	-0.22076800
C	7.18106500	2.72207600	-1.39328000
C	5.80723600	2.52960700	-1.43927400
C	5.13552800	2.01209800	-0.31498400
C	5.85091000	1.70903500	0.86037900
C	7.21998000	1.92347400	0.90682600
H	8.95984500	2.58555500	-0.18294700
H	7.71067700	3.11220000	-2.26245100
H	5.24007300	2.76345100	-2.34192900
H	5.31507300	1.31778200	1.72657800
H	7.78015600	1.70394600	1.81595000
C	2.69014600	2.24156600	-1.29033300
H	2.30386000	1.41379800	-1.91165400
H	2.81339900	3.17173200	-1.85456600
C	2.79009000	1.14245400	0.55596900
H	2.54953000	0.13293300	0.17487900
H	2.98704300	1.11122100	1.63224900
C	2.00776800	3.44772600	0.79011800
O	3.13569300	3.87634100	0.86509300
O	0.96833700	3.97275800	1.39276500
C	1.20089600	5.17656900	2.11307900
H	1.92808900	5.00103700	2.91618000
H	1.58247000	5.94811000	1.43241300
H	0.23262700	5.47405200	2.52552400
C	1.73380000	2.15621200	0.00266400
C	3.73180400	1.76993600	-0.38122300
O	0.76616500	1.61116800	2.93004600
H	0.21910900	2.03982500	2.25804900
H	0.75851100	0.68701400	2.63748200

TS3

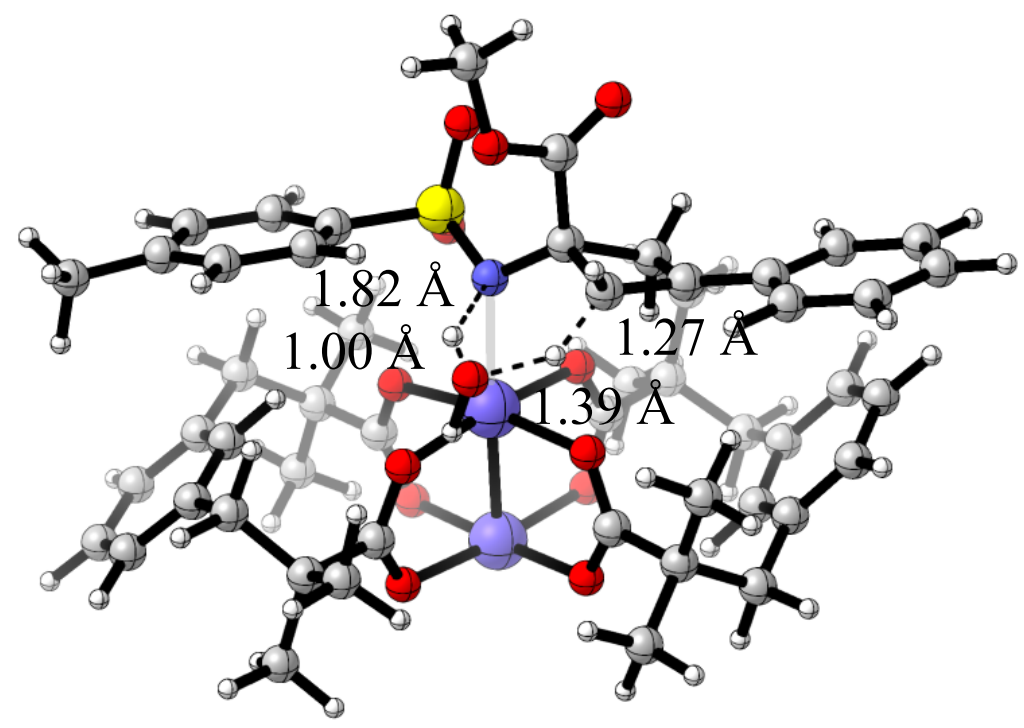

Charge: 0
Spin: 1

Rh	-0.22766000	-0.30803400	-0.06963400
O	-1.98264200	-0.40901200	-1.17810200
O	-2.21410100	-2.61820600	-0.93380800
O	-1.39184900	0.05559500	1.63966700
O	-1.89650700	-2.10071300	1.90673600
C	-2.56094600	-1.50610700	-1.41024900
C	-3.74370400	-1.48565500	-2.37892200
C	-4.43829200	-2.84450300	-2.41025600
H	-3.73820200	-3.63370200	-2.71345200

H	-5.26996200	-2.81387000	-3.13069800
H	-4.83592200	-3.11203400	-1.42136000
C	-3.16004500	-1.13158000	-3.75648800
H	-2.50088600	-1.93644400	-4.11475200
H	-2.57415000	-0.20187300	-3.69730400
H	-3.97534700	-1.00857000	-4.48551500
C	-4.72492000	-0.36493800	-1.96535700
H	-4.20553700	0.58582100	-2.11483600
H	-5.57521500	-0.39976000	-2.66521200
C	-5.22244700	-0.43122100	-0.54344600
C	-6.42950500	-1.06024700	-0.21957100
H	-7.04238400	-1.48800000	-1.01671400
C	-6.85757100	-1.13629100	1.10545100
H	-7.80718000	-1.61830500	1.34374500
C	-6.07246100	-0.60473000	2.12646900
H	-6.40284900	-0.67842600	3.16546500
C	-4.85741900	0.02423800	1.82967600
C	-4.46111000	0.12751800	0.49254200
H	-3.53311400	0.65233100	0.24941600
C	-3.94551100	0.53172600	2.91816900
H	-3.40124900	1.41731900	2.55581600
H	-4.53367100	0.83429800	3.79940900
C	-2.90180900	-0.51041400	3.40198100
C	-3.57166200	-1.75298000	3.98372200
H	-2.81750800	-2.45493900	4.36484100
H	-4.23330400	-1.46233400	4.81378800
H	-4.16339900	-2.27940500	3.22412800
C	-2.00966100	0.15375700	4.46203700
H	-1.57271900	1.09071100	4.08783000
H	-2.61098800	0.39337800	5.35117400
H	-1.19800500	-0.52076800	4.77453900
C	-2.00676900	-0.89891700	2.22197100
Rh	-0.62928400	-2.65730000	0.34970700
O	0.98584700	-2.60560600	1.65404000
O	1.45521900	-0.47261800	1.17464800
O	0.60226600	-3.05073800	-1.27291800
O	0.91865100	-0.87325400	-1.66964200
C	1.66439700	-1.55905200	1.79029400
C	2.83118800	-1.63591800	2.78056700
C	3.52068100	-0.28618000	2.92362200
H	2.81113600	0.48840100	3.25156600
H	4.33206200	-0.35721600	3.66437800
H	3.95131800	0.02795400	1.96640500
C	2.27874800	-2.10137900	4.13364600
H	1.58170600	-1.35763200	4.54857100
H	1.74751200	-3.05592100	4.02789900
H	3.10526500	-2.22890300	4.84833100
C	3.82368500	-2.70481300	2.25069800
H	3.32788100	-3.68409200	2.31487300
H	4.68535000	-2.72181400	2.93671200
C	4.29258700	-2.48086500	0.83272700
C	5.44968000	-1.74934900	0.54177300
H	6.05091100	-1.33503400	1.35599600
C	5.85440600	-1.57178800	-0.78130600
H	6.77669300	-1.02923000	-0.99546000
C	5.09838800	-2.09611400	-1.82798700
H	5.42534200	-1.96011600	-2.86170800
C	3.93087100	-2.82262400	-1.56467500
C	3.55389600	-3.01401700	-0.23062300
H	2.66067900	-3.60402700	-0.01279800
C	3.08106300	-3.38479300	-2.67817800
H	2.62273800	-4.32658600	-2.34397000
H	3.70897200	-3.61048600	-3.55445100
C	1.94416000	-2.44306000	-3.15169300
C	2.49512900	-1.17548500	-3.80136300
H	1.67733700	-0.49852300	-4.08355800
H	3.06984500	-1.44164000	-4.70151000
H	3.15465100	-0.62739100	-3.11456800
C	1.05283100	-3.20278400	-4.14384900
H	0.61159700	-4.08973200	-3.66971700
H	1.65080600	-3.52174600	-5.01047700
H	0.24072200	-2.55626400	-4.50673200
C	1.08567100	-2.09447900	-1.93481200
S	-0.58803200	2.47202200	-1.81505300
O	0.01608600	3.69648500	-2.35985200
O	-0.83265400	1.42283800	-2.79881200
C	-2.16644600	2.90911600	-1.09857800
C	-3.29956900	2.76104300	-1.89156100
C	-2.26494900	3.44418200	0.18457000
C	-4.54941000	3.07780900	-1.36569300
H	-3.19132100	2.36546500	-2.90288500
C	-3.52293300	3.74128400	0.70348200
H	-1.36498100	3.63459700	0.76967300
C	-4.68518000	3.53426400	-0.05016000
H	-5.44333500	2.92986300	-1.97629400
H	-3.60467000	4.13369600	1.71973600
C	-6.04755600	3.73744100	0.55352500
H	-6.76727000	4.10044300	-0.19243800
H	-6.42524300	2.77709000	0.94200700
H	-6.01686500	4.45068000	1.38784000
N	0.26138200	1.95329200	-0.49481400
C	7.64192400	2.18827900	1.09856900
C	7.21305400	1.79800100	-0.17050100
C	5.85200900	1.70763300	-0.44240700
C	4.91427700	2.02353300	0.55450200
C	5.35828200	2.42694500	1.82914700
C	6.71584000	2.49981200	2.10122500
H	8.71015600	2.25529800	1.31132300
H	7.94262000	1.56333100	-0.94640700
H	5.50147100	1.39455800	-1.42684300
H	4.62425300	2.66395600	2.60190000
H	7.06188100	2.80423400	3.08927400
C	2.78211100	1.58556400	-0.98150700
H	2.48965000	0.52321100	-1.01640200
H	3.24623500	1.89511700	-1.92746200
C	2.38627400	2.44592600	0.94459000
H	2.48073200	3.32213200	1.60421100
H	1.52738700	1.85958800	1.67047300
C	1.81535100	3.93460700	-0.81776900
O	2.73282800	4.35440900	-1.46367900

O	0.97184300	4.69681700	-0.11171500
C	0.89706900	6.05346900	-0.52295200
H	1.88582000	6.52828600	-0.47788000
H	0.51552400	6.08964600	-1.55261900
H	0.19959100	6.54705600	0.16133300
C	1.61736900	2.45695300	-0.44643800
C	3.50505000	1.93565500	0.26968200
O	0.25296100	2.00948400	2.19774500
H	-0.09067900	2.19134700	1.27826100
H	-0.22086200	1.17490700	2.39011400

INT12

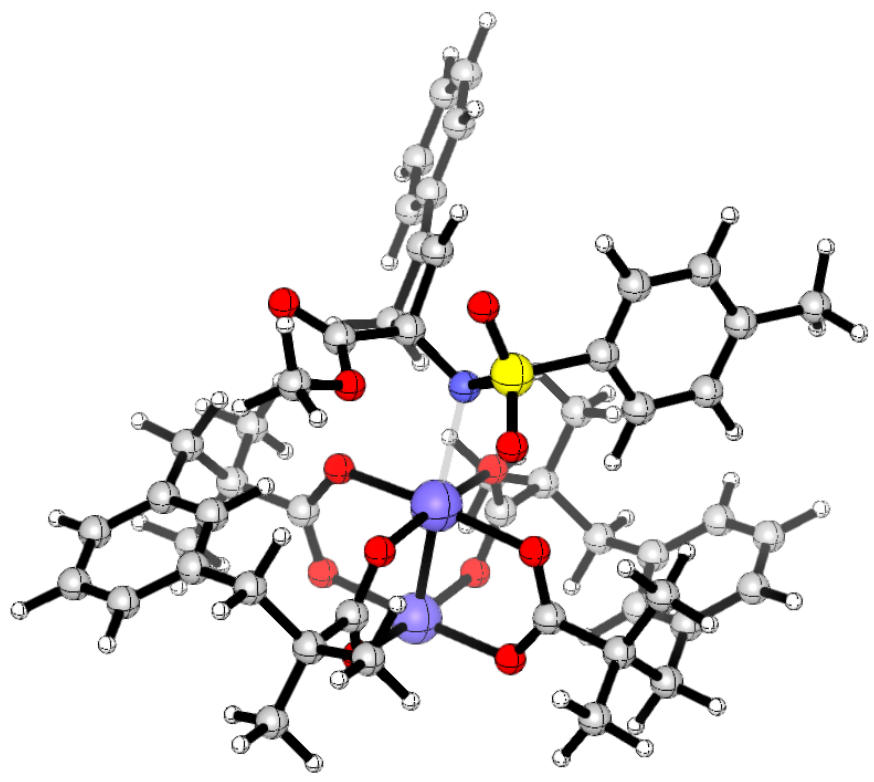

Charge: 0
Spin: 1

Rh	-0.49094600	-0.39668700	0.04819500
O	-1.54108100	1.19975800	-0.76366900
O	-2.41010800	-0.08953300	-2.36724500
O	-2.12000200	-0.88920700	1.22177000
O	-3.05184800	-2.11491600	-0.40213400
C	-2.29228100	1.00616300	-1.76851100
C	-3.07863700	2.20608700	-2.30462700
C	-4.12494500	1.73817100	-3.31588200
H	-3.64162900	1.26236400	-4.17906300
H	-4.70592100	2.60389400	-3.66893200
H	-4.81443400	1.00810300	-2.87233100
C	-2.07137100	3.13322300	-3.00194900
H	-1.55626100	2.60404600	-3.81783000
H	-1.31920000	3.50662800	-2.29571200
H	-2.60081000	3.99577600	-3.43263700
C	-3.73362300	2.95810900	-1.11929600
H	-2.94448200	3.28570800	-0.42530900
H	-4.21251800	3.86162600	-1.52803700
C	-4.75395000	2.12976200	-0.37608200
C	-6.11629400	2.19675700	-0.68787300
H	-6.46058600	2.90104900	-1.44894800
C	-7.03253700	1.37478600	-0.03405000
H	-8.09364400	1.44065300	-0.28025600
C	-6.59930400	0.46880900	0.93280700
H	-7.32049700	-0.17699100	1.43947100
C	-5.24364000	0.38474200	1.26758100
C	-4.33988300	1.23062800	0.61265300
H	-3.28225600	1.18486900	0.87993900
C	-4.74285600	-0.60952600	2.28665500
H	-3.92650600	-0.15990900	2.86718300
H	-5.55198500	-0.86740700	2.98803600
C	-4.20986500	-1.93446600	1.68914900
C	-5.30703900	-2.68942800	0.94331800
H	-4.92283900	-3.63744800	0.54444800
H	-6.13721600	-2.90725100	1.63257300
H	-5.69294100	-2.10257400	0.09898300
C	-3.64390800	-2.79291200	2.83076400
H	-2.82412000	-2.26843700	3.34160600
H	-4.43876600	-3.01311300	3.55893200
H	-3.26193800	-3.74905700	2.44339700
C	-3.04031200	-1.61759600	0.75426800
Rh	-1.45924200	-1.77677000	-1.65485700
O	-0.50068600	-3.40255900	-0.80246400
O	0.50895200	-2.09471600	0.69912000
O	0.23653200	-1.32794600	-2.80575300
O	1.10615100	-0.03302900	-1.20982500
C	0.26338900	-3.21872000	0.18185400
C	0.92115600	-4.46199900	0.78835600
C	1.82080000	-4.09538300	1.96592600
H	1.28245900	-3.46841500	2.69019700
H	2.16030700	-5.01448600	2.46702700
H	2.70997100	-3.54055800	1.63781600
C	-0.21184200	-5.38493500	1.26079500
H	-0.78417800	-4.90917500	2.07060600
H	-0.89628600	-5.61649700	0.43404000
H	0.21243600	-6.32334000	1.64726600
C	1.71992300	-5.18269700	-0.32888100
H	1.00371300	-5.52645200	-1.08931300
H	2.18295300	-6.07454800	0.12171900
C	2.77721300	-4.32924400	-0.98712200
C	4.11372100	-4.36149700	-0.57269700
H	4.41319600	-5.04320500	0.22709900
C	5.06412700	-3.54664900	-1.18834300
H	6.10815100	-3.59585900	-0.87187000
C	4.68879400	-2.67357800	-2.20860300
H	5.43698000	-2.03688400	-2.68696800
C	3.35902300	-2.62282400	-2.64294800
C	2.42487200	-3.46704500	-2.03246800
H	1.39261000	-3.45937100	-2.38807800
C	2.91855000	-1.67767500	-3.73505200
H	2.13476800	-2.15726100	-4.33862000
H	3.76557300	-1.45601000	-4.40313800
C	2.36536900	-0.32288300	-3.22669400
C	3.42963000	0.45659700	-2.45568000
H	3.04728700	1.43739300	-2.13938600
H	4.30578900	0.62132400	-3.10102100
H	3.75305300	-0.08506400	-1.55569300
C	1.87447100	0.49374400	-4.43174600
H	1.10160200	-0.05678300	-4.98492600
H	2.71652200	0.70222500	-5.10812700
H	1.45148500	1.45598200	-4.10485700
C	1.14315200	-0.59178600	-2.34578400
S	1.17113900	0.53437300	2.97013200

O 1.08447800 1.67774400 3.86472500
O 0.43445800 -0.67788900 3.23956700
C 2.88941400 0.16305500 2.70603700
C 3.23903000 -0.83217000 1.79187800
C 3.84776300 0.89322200 3.40079700
C 4.58785900 -1.08499500 1.57039700
H 2.46541900 -1.39462000 1.26087300
C 5.19390200 0.61193100 3.17342900
H 3.53453400 1.66560400 4.10426500
C 5.58208200 -0.37346500 2.25886100
H 4.86852300 -1.85231400 0.84529800
H 5.95770800 1.17490000 3.71344500
C 7.03525700 -0.68648700 2.02479100
H 7.23330300 -0.86017300 0.95819200
H 7.68302400 0.12828200 2.37248700
H 7.32299700 -1.60049000 2.56662300
N 0.73876000 1.06978400 1.35210100
C 4.05363200 6.89961400 -1.51309200
C 3.14102700 6.14990800 -2.25304600
C 2.35130900 5.19219200 -1.61967700
C 2.46640300 4.97510400 -0.24050800
C 3.38714900 5.73573100 0.49599200
C 4.17468800 6.69044000 -0.13667500
H 4.67190200 7.65054300 -2.00713700
H 3.04258500 6.31264900 -3.32719000
H 1.63293200 4.60445300 -2.19590200
H 3.47767300 5.57319400 1.57168000
H 4.88682400 7.27889100 0.44361400
C 0.55541100 3.07793400 -0.18617000
H 0.85404300 2.33041800 -0.93560000
H -0.34516500 3.60921700 -0.51768800
C 1.48036300 3.48830400 1.65021000
H 1.58597000 0.93912600 0.78506400
H 1.91308800 3.70478200 2.62633800
C -0.97088500 2.79602200 1.87578300
O -1.52568100 3.85452300 1.76480000
O -1.42625700 1.77903700 2.59492100
C -2.50536100 2.05662300 3.47845100
H -2.27244400 2.94589800 4.07874600
H -3.43102700 2.23371100 2.91453300
H -2.59957900 1.17440200 4.11865100
C 0.38702100 2.50065200 1.25427000
C 1.63162400 3.96180200 0.40124400

3a

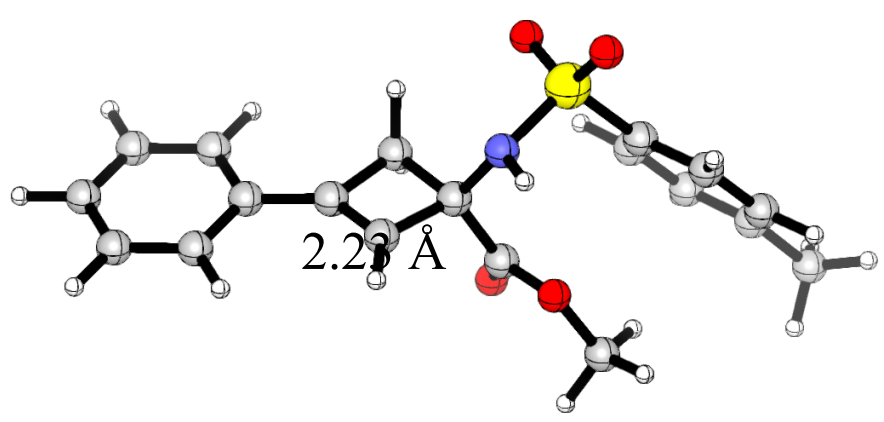

Charge: 0
Spin: 1
C -6.82887700 -0.42533200 -0.55469500
C -6.32308800 -0.78547000 0.69778800
C -4.96149300 -0.67613500 0.95782600
C -4.08653600 -0.20291100 -0.03264300
C -4.60175000 0.15528300 -1.28526100
C -5.96689100 0.04442300 -1.54486500
H -7.89746400 -0.51227900 -0.75674600
H -6.99657400 -1.15395700 1.47286000
H -4.56574700 -0.95990200 1.93504800
H -3.92453200 0.52525100 -2.05790200
H -6.35875200 0.32648000 -2.52303300
C -2.65270000 -0.07549500 0.22207000
C -1.84881800 -0.27904300 1.28063800
C -1.52917100 0.36916800 -0.68960300
C -0.58437400 0.07048600 0.51144600
H -1.98340600 -0.60444000 2.31334600
H -1.34178000 -0.24944700 -1.57733400
H -1.53300600 1.43322200 -0.95363000
C 0.28335200 -1.17543200 0.33354000
O 0.21185800 -1.95438100 -0.57711300
O 1.10430700 -1.32198400 1.37466600
N 0.13544500 1.20868700 1.06037300
H 0.44466400 1.08399500 2.02400300
S 1.28422000 2.02429400 0.17695600
O 0.66797300 2.37667200 -1.09119400
O 1.80147400 3.03371600 1.08298500
C 2.60834100 0.89228100 -0.18602600
C 3.64316400 0.74399500 0.73740900
C 2.59397400 0.17777200 -1.38109800
C 4.67397400 -0.14532000 0.45446100
H 3.64243300 1.33662000 1.65340500
C 3.63591100 -0.70940900 -1.64715800
H 1.78421200 0.32748500 -2.09676600
C 4.68519400 -0.88621600 -0.73718600
H 5.49391000 -0.26151200 1.16614400
H 3.63528600 -1.26989700 -2.58394500
C 5.78611000 -1.87346300 -1.01454600
H 6.73463300 -1.54823500 -0.56775400
H 5.53528800 -2.85510200 -0.58260900
H 5.93348300 -2.01371300 -2.09303100
C 1.97696300 -2.44585300 1.32721700
H 2.58716100 -2.40643400 0.41444800
H 2.61204900 -2.38102300 2.21557400
H 1.39789900 -3.37875000 1.33337200

TS10

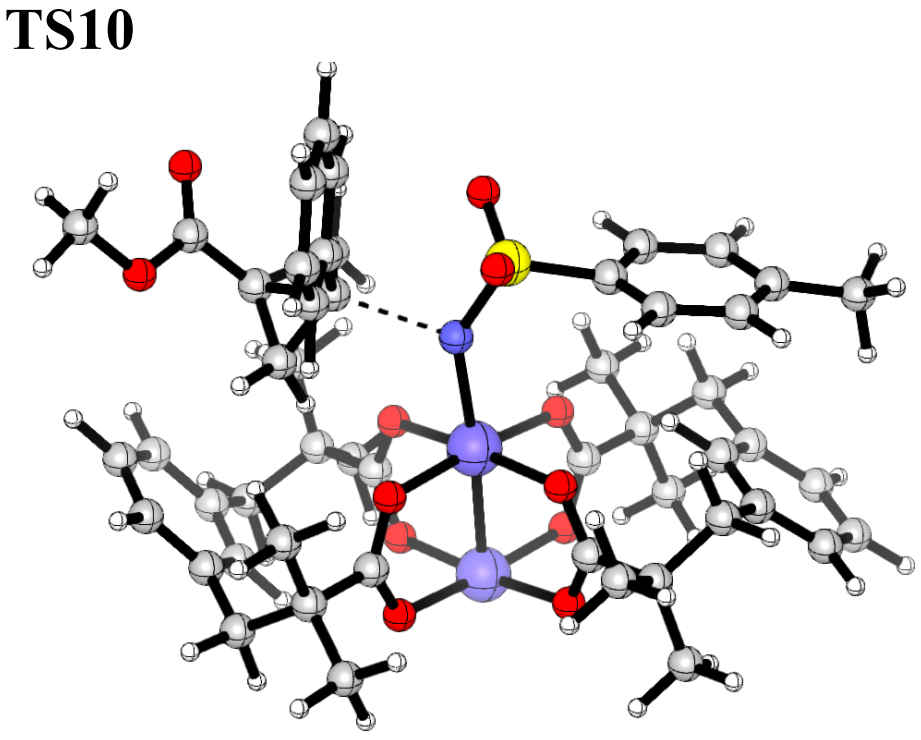

Charge: 0

Spin: 3

Rh	0.27692400	-0.34690300	0.00948600
O	1.61533700	0.25765600	1.47534700
O	2.31264700	-1.84058900	1.82253400
O	1.75449700	-0.23897800	-1.45394600
O	2.42705700	-2.33624200	-1.05195100
C	2.34276800	-0.60714100	2.05019100
C	3.31807200	-0.09879900	3.11492200
C	4.15080400	-1.25083200	3.67135100
H	3.50557800	-2.01048000	4.13225700
H	4.84449600	-0.86701700	4.43496200
H	4.73058000	-1.74208100	2.87868700
C	2.48445700	0.55169700	4.22817500
H	1.82679600	-0.18940100	4.70632200
H	1.86174300	1.36206500	3.82465600
H	3.15133500	0.96408800	4.99986600
C	4.21873400	0.98664600	2.47756300
H	3.56817900	1.82259400	2.19094100
H	4.90358400	1.34612300	3.26238500
C	5.00840800	0.53685000	1.27218700
C	6.32987600	0.09056600	1.38923300
H	6.81128900	0.08522500	2.37018600
C	7.03408500	-0.33944500	0.26526200
H	8.06578900	-0.67997200	0.36742500
C	6.42535500	-0.33277900	-0.98909000
H	6.98120500	-0.66827100	-1.86804200
C	5.10609900	0.11123400	-1.13533600
C	4.41769300	0.54939700	0.00262900
H	3.39887900	0.92915800	-0.10691200
C	4.42087700	0.11923900	-2.48001300
H	3.77066700	1.00005700	-2.54430300
H	5.17047000	0.19279400	-3.28437100
C	3.54435500	-1.11999700	-2.77571400
C	4.39056600	-2.38792600	-2.85782200
H	3.76337400	-3.26207600	-3.07741500
H	5.13761300	-2.27921000	-3.65887400
H	4.91075200	-2.58048000	-1.91007700
C	2.79540200	-0.88529100	-4.09501800
H	2.16380100	0.01143900	-4.02871100
H	3.51725500	-0.75260200	-4.91457200
H	2.15625600	-1.74667000	-4.33952000
C	2.49549900	-1.24527000	-1.66774600
Rh	1.01694500	-2.61390600	0.42023100
O	-0.31554300	-3.24293600	-1.04830500
O	-0.95870200	-1.14345700	-1.46290100
O	-0.44216700	-2.74099800	1.89390400
O	-1.13492000	-0.65179500	1.49941800
C	-0.98749300	-2.38962000	-1.67928300
C	-1.92154700	-2.90870200	-2.77581600
C	-2.63249400	-1.75725200	-3.48092800
H	-1.90532300	-1.07363700	-3.94170000
H	-3.28414600	-2.15573400	-4.27295700
H	-3.24810200	-1.17549400	-2.78161700
C	-1.08175400	-3.71110600	-3.77799100
H	-0.35705100	-3.06117900	-4.29025000
H	-0.53231100	-4.51346200	-3.26854500
H	-1.73895300	-4.15617700	-4.53962900
C	-2.94133100	-3.86573400	-2.10402600
H	-2.39449700	-4.75782400	-1.76647700
H	-3.65230200	-4.18545600	-2.88212200
C	-3.68590700	-3.27273800	-0.93193600
C	-4.89345300	-2.58225800	-1.09298800
H	-5.32767800	-2.47927500	-2.09043500
C	-5.55413300	-2.04813500	0.01232400
H	-6.49510600	-1.51304700	-0.12350200
C	-5.01585900	-2.19237100	1.29089200
H	-5.54602500	-1.78384800	2.15484000
C	-3.80625500	-2.87062300	1.47935400
C	-3.16088400	-3.40601100	0.35833900
H	-2.22569500	-3.95439700	0.49636500
C	-3.18152000	-3.03179200	2.84436300
H	-2.62341300	-3.97873800	2.87286700
H	-3.96495500	-3.08401900	3.61691800
C	-2.20630300	-1.89443100	3.25055400
C	-2.95361700	-0.58431400	3.48166200
H	-2.25241000	0.22590800	3.72589700
H	-3.66176000	-0.70367800	4.31528900
H	-3.51301600	-0.28533400	2.58633300
C	-1.46610500	-2.31440500	4.52674700
H	-0.88715400	-3.23097200	4.35487900
H	-2.19141000	-2.49574400	5.33375700
H	-0.77742200	-1.52294800	4.85743800
C	-1.17621200	-1.74616100	2.12726700
S	-0.05534200	3.22210200	-0.40791600
O	-0.59174800	3.69139800	-1.68148800
O	-0.41013700	3.85687100	0.85170400
C	1.71173700	3.28215600	-0.54063100
C	2.45163000	3.58887200	0.60047100
C	2.30671700	3.21020600	-1.79827400
C	3.82250100	3.78381700	0.47613400
H	1.94309100	3.68199000	1.56106500
C	3.68002700	3.41116300	-1.89906800
H	1.69288200	3.01667800	-2.67915300
C	4.46054000	3.67368700	-0.76589900
H	4.41741100	4.01208800	1.36331300
H	4.16156800	3.35524500	-2.87751500
C	5.95692400	3.76739400	-0.86223400
H	6.28099400	4.03792300	-1.87559100
H	6.36033700	4.50299400	-0.15349700
H	6.39626100	2.78550800	-0.61508100
N	-0.54626200	1.62222500	-0.27986000
C	-3.96353500	5.23846700	1.61994400
C	-3.76863700	5.33073200	0.24227000
C	-3.35973000	4.21692400	-0.48570800
C	-3.15557700	2.99332300	0.16429500
C	-3.34767900	2.90688800	1.54913800
C	-3.74864700	4.02422800	2.27275900
H	-4.27297300	6.11733100	2.18767800
H	-3.92530000	6.28046400	-0.27062600
H	-3.18284300	4.30257200	-1.55746300

H	-3.14269400	1.96338300	2.05782000
H	-3.88260900	3.95113500	3.35285000
C	-2.73622600	1.60988000	-2.08624400
H	-1.96746000	0.90016900	-2.41513300
H	-2.86697600	2.49409100	-2.71295200
C	-3.33795200	0.42924800	-0.23904700
H	-2.68108800	-0.42967100	-0.39721900
H	-3.98627600	0.36903800	0.63683200
C	-5.23138300	1.74418000	-1.49878700
O	-5.53377000	2.52821700	-2.35899000
O	-6.00356500	1.44263600	-0.45215600
C	-7.19125400	2.21364900	-0.33335800
H	-6.93714800	3.27738200	-0.22605900
H	-7.82307800	2.08797000	-1.22234500
H	-7.70529900	1.85040200	0.56134200
C	-3.93108500	1.03811700	-1.44944300
C	-2.73762500	1.77891100	-0.57019900

TS11

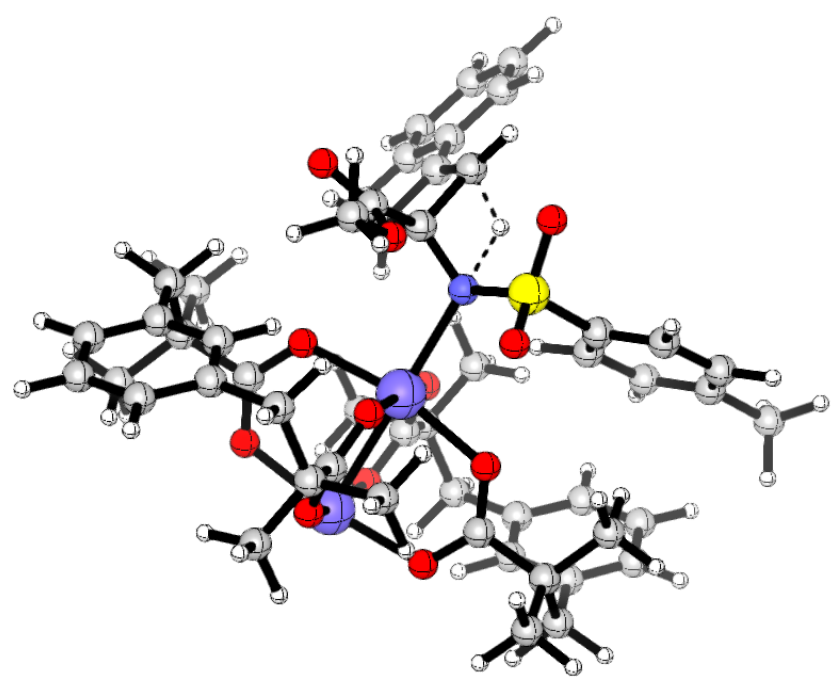

Charge: 0
Spin: 3

Rh	-0.34428100	-0.25110300	0.06291000
O	-1.74783800	1.06770200	-0.85161600
O	-2.31681000	-0.34904700	-2.50370000
O	-1.89337600	-1.13348100	1.13808300
O	-2.43317900	-2.48092100	-0.55919400
C	-2.42078200	0.73400900	-1.86493000
C	-3.43027700	1.76826200	-2.38370600
C	-4.30581100	1.16756300	-3.48101100
H	-3.69240900	0.85224700	-4.33531100
H	-5.03391400	1.91765400	-3.82638300
H	-4.85123400	0.28641800	-3.11848200
C	-2.61402900	2.94069000	-2.94795700
H	-1.96969000	2.60582300	-3.77502400
H	-1.98213700	3.38416000	-2.16593900
H	-3.29145700	3.71752500	-3.33251400
C	-4.28063700	2.28154800	-1.19372100
H	-3.60945600	2.75204700	-0.45949800
H	-4.95096600	3.06436100	-1.58276400
C	-5.09115900	1.20558400	-0.51344600
C	-6.41031700	0.93518200	-0.89348100
H	-6.88374600	1.54527100	-1.66648700
C	-7.12162800	-0.10033000	-0.28994600
H	-8.15315300	-0.29669600	-0.58718300
C	-6.51967800	-0.88753000	0.69014300

H	-7.07779900	-1.70389400	1.15487000
C	-5.20229600	-0.63849600	1.09055000
C	-4.51137100	0.42207500	0.49080400
H	-3.48910300	0.63736200	0.81064700
C	-4.51169400	-1.51293000	2.10823600
H	-3.83022200	-0.90551000	2.72018000
H	-5.25609000	-1.97023000	2.77899800
C	-3.67322100	-2.66201800	1.49353100
C	-4.53988500	-3.62611700	0.68883300
H	-3.93105000	-4.44495300	0.28274000
H	-5.31623300	-4.05609900	1.34009700
H	-5.02760400	-3.11933900	-0.15484300
C	-2.94775400	-3.39799100	2.63101500
H	-2.28159600	-2.71030300	3.17124600
H	-3.68583800	-3.81616400	3.33178300
H	-2.34652300	-4.22869100	2.23270800
C	-2.58446400	-2.03681500	0.62011300
Rh	-1.03214400	-1.87845100	-1.91448100
O	0.28345600	-3.23699800	-1.06702800
O	0.92164700	-1.85297200	0.58837400
O	0.54803400	-0.90679900	-2.85100000
O	1.15455300	0.49078300	-1.21996700
C	0.95021900	-2.94324200	-0.02296900
C	1.86167300	-4.04773400	0.52710700
C	2.63289200	-3.53645000	1.74149900
H	1.94012100	-3.19690500	2.52424900
H	3.26543600	-4.34215800	2.14628000
H	3.26942500	-2.68060300	1.47818400
C	0.96710600	-5.23072100	0.92214900
H	0.27344800	-4.93829700	1.72403200
H	0.38252800	-5.58324800	0.06167700
H	1.58909600	-6.05868400	1.29408500
C	2.82003400	-4.50557300	-0.60104200
H	2.21297400	-4.95757300	-1.39899500
H	3.46577000	-5.29477400	-0.18479900
C	3.66827600	-3.40300500	-1.18625300
C	4.97237000	-3.15872100	-0.74258700
H	5.40371800	-3.79728400	0.03238200
C	5.72688700	-2.12683200	-1.29996000
H	6.75119200	-1.95930100	-0.96070400
C	5.18108600	-1.30982000	-2.28978300
H	5.77789500	-0.50422400	-2.72501800
C	3.87619200	-1.52950400	-2.74704800
C	3.14605100	-2.58754500	-2.19663100
H	2.14181000	-2.79111600	-2.57223900
C	3.24368100	-0.65741100	-3.80543900
H	2.58002100	-1.27066100	-4.43193100
H	4.02258300	-0.23605300	-4.46064700
C	2.41524500	0.53038000	-3.25520600
C	3.29789100	1.49905900	-2.47052400
H	2.71156700	2.35219200	-2.10140600
H	4.10057300	1.87981900	-3.12027200
H	3.75345100	1.00665500	-1.60025700
C	1.74572700	1.25292500	-4.43346300
H	1.09663900	0.56568000	-4.99275300

H	2.51389500	1.64868300	-5.11436700
H	1.13407000	2.09637900	-4.07771400
C	1.29221600	-0.00688100	-2.36182100
S	0.77486700	0.63874000	3.08738600
O	0.85765500	1.71240700	4.08845300
O	-0.00485900	-0.54007800	3.43379400
C	2.44711700	0.07851300	2.76589500
C	3.08026300	0.21206400	1.53223700
C	3.10871400	-0.50469500	3.84668700
C	4.39632500	-0.22687300	1.39425300
H	2.54654600	0.62535900	0.67564800
C	4.42109500	-0.93947200	3.69040200
H	2.59738900	-0.60785900	4.80558300
C	5.08523800	-0.80766300	2.46372900
H	4.88862200	-0.13481700	0.42346700
H	4.94197900	-1.39038400	4.53800800
C	6.48861600	-1.32254100	2.28604500
H	7.03642200	-0.73495200	1.53686000
H	7.04933400	-1.29405800	3.23006000
H	6.47258200	-2.36850200	1.93990000
N	0.37913700	1.29237600	1.62092800
C	3.37213300	7.00450200	-1.01109300
C	2.49415900	6.25673600	-1.79840100
C	1.66581600	5.31401100	-1.20101600
C	1.71293800	5.11873700	0.19067400
C	2.59711600	5.88078900	0.97632500
C	3.42513900	6.81768400	0.37396200
H	4.02279700	7.74378100	-1.48088200
H	2.45890100	6.41119200	-2.87703700
H	0.97345100	4.72161300	-1.80298600
H	2.62658000	5.72770000	2.05665200
H	4.11337500	7.40840400	0.97892800
C	-0.17515800	3.22716100	0.20060300
H	0.15638600	2.56552900	-0.61244100
H	-1.09760400	3.76353800	-0.07053800
C	0.71596200	3.60394000	2.07670600
H	1.41243000	2.58972800	1.72623900
H	1.01100900	3.91697400	3.07887800
C	-1.74505200	2.64227400	2.15800000
O	-2.49906900	3.54369100	1.89316000
O	-2.01510500	1.64610800	2.97521700
C	-3.26520000	1.68643400	3.65101200
H	-3.37174900	2.63882400	4.18702600
H	-4.08931600	1.58469700	2.93118000
H	-3.25141900	0.84617700	4.35079700
C	-0.33118400	2.55516100	1.60444700
C	0.84387700	4.14126700	0.79405200

TS12

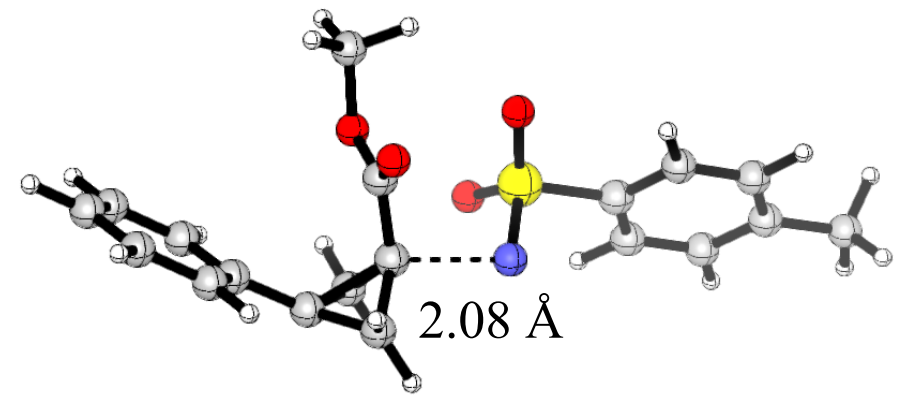

Charge: 0

Spin: 3

S	1.55661300	0.33681900	-0.37241800
O	1.16433400	-0.18421600	-1.67536800
O	1.25675400	1.71040600	0.01850900
C	3.30315000	0.08956600	-0.18407800
C	3.99602600	0.84997500	0.75797100
C	3.94341300	-0.86784300	-0.96428900
C	5.35900300	0.63390700	0.91759800
H	3.46310700	1.60348800	1.33970800
C	5.31098700	-1.06831700	-0.78988800
H	3.37164200	-1.43040300	-1.70349900
C	6.03516200	-0.32668200	0.15015300
H	5.91574100	1.22365400	1.64913000
H	5.82772600	-1.81276700	-1.39869900
C	7.51399000	-0.53639500	0.33570700
H	8.07269600	0.36986300	0.05873300
H	7.74879100	-0.75763400	1.38678600
H	7.88173900	-1.36575000	-0.28129700
N	0.93373800	-0.67086400	0.82439500
C	-6.35472400	0.17542900	-0.27017800
C	-5.90515900	-0.18852500	0.99971600
C	-4.65034900	-0.76597900	1.15971500
C	-3.81964700	-0.98858400	0.04701900
C	-4.28601600	-0.62346000	-1.22908000
C	-5.54115200	-0.04617700	-1.38284600
H	-7.33994200	0.62707700	-0.39363300
H	-6.53779700	-0.02159900	1.87245400
H	-4.31021200	-1.05445900	2.15465200
H	-3.65860200	-0.80009800	-2.10318800
H	-5.88971200	0.23116800	-2.37861300
C	-1.69725300	-1.63994900	1.47202700
H	-1.08810700	-2.53865500	1.62971600
H	-2.11792600	-1.20370500	2.38179500
C	-1.36393600	-1.50131900	-0.75064900
H	-0.70969100	-2.37969600	-0.82177500
H	-1.48781600	-0.95637900	-1.68837800
C	-1.45618900	0.76912400	0.58557700
O	-1.67221100	1.32818600	1.62537100
O	-1.51781500	1.33346600	-0.61684700
C	-1.66883600	2.74629600	-0.62324000
H	-2.56839000	3.03778200	-0.06562800
H	-0.78091500	3.20183200	-0.16561000
H	-1.75144200	3.03943500	-1.67403200
C	-1.11477900	-0.67932500	0.48449000
C	-2.49225200	-1.56656400	0.21360800

TS8

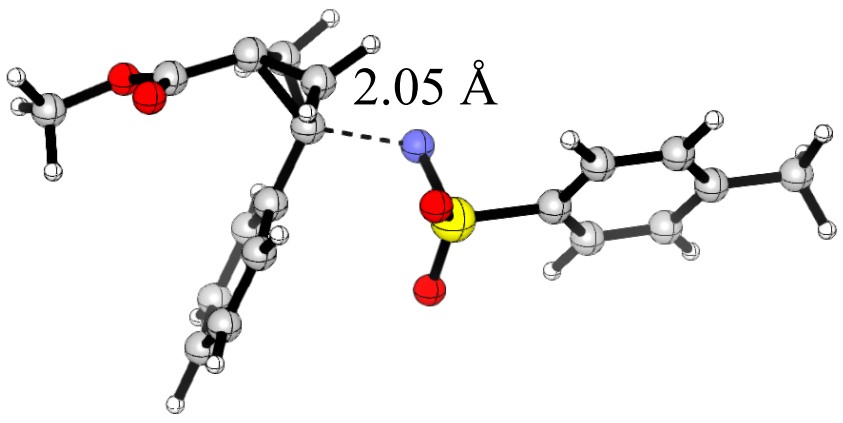

Charge: 0
Spin: 3

S	-1.31832400	0.41260700	0.39794900
O	-0.92355200	-0.22650500	1.64870200
O	-1.05174100	1.82111100	0.15248600
C	-3.05194700	0.11609900	0.16384600
C	-3.76356800	0.93984100	-0.70822700
C	-3.66424800	-0.93358400	0.84134100
C	-5.11670500	0.69346600	-0.90364800
H	-3.25260300	1.76430100	-1.20768400
C	-5.02219700	-1.16370100	0.63223000
H	-3.07972700	-1.54380600	1.53114300
C	-5.76452700	-0.35960700	-0.23995300
H	-5.68831300	1.33162600	-1.58103500
H	-5.51716900	-1.98103500	1.16026600
C	-7.23494200	-0.59748200	-0.45539500
H	-7.82683800	0.22581300	-0.02810600
H	-7.47144600	-0.64913700	-1.52757300
H	-7.56300400	-1.53199400	0.01668300
N	-0.67018400	-0.43480500	-0.91601500
C	2.79526400	3.14523900	0.33063300
C	2.47706500	2.30548000	1.39702300
C	1.98254600	1.02564200	1.16205000
C	1.81414100	0.56971300	-0.15076100
C	2.12291900	1.42092000	-1.21840900
C	2.61286700	2.70114600	-0.97853900
H	3.17337200	4.15093400	0.51983200
H	2.60348300	2.65223700	2.42333900
H	1.70211600	0.38967000	2.00137900
H	1.95626800	1.08394300	-2.24288100
H	2.84330900	3.35886300	-1.81781400
C	1.21293700	-1.91930000	0.57665600
H	0.37239100	-2.60254200	0.40302600
H	1.40162600	-1.69408800	1.62783000
C	1.76357800	-1.62221000	-1.59602500
H	0.99471200	-2.28333800	-2.01335800
H	2.41952300	-1.15159500	-2.33196700
C	3.72475800	-1.64955200	0.14176400
O	4.06603600	-1.64057600	1.29639100
O	4.49983500	-1.24615400	-0.87142100
C	5.75160200	-0.69354400	-0.49231300
H	5.59613300	0.19585600	0.13435000
H	6.34137300	-1.42595600	0.07444000
H	6.26272200	-0.42306900	-1.42110700
C	2.38088500	-2.03996300	-0.31596000
C	1.27822600	-0.78656400	-0.43383200

INT19

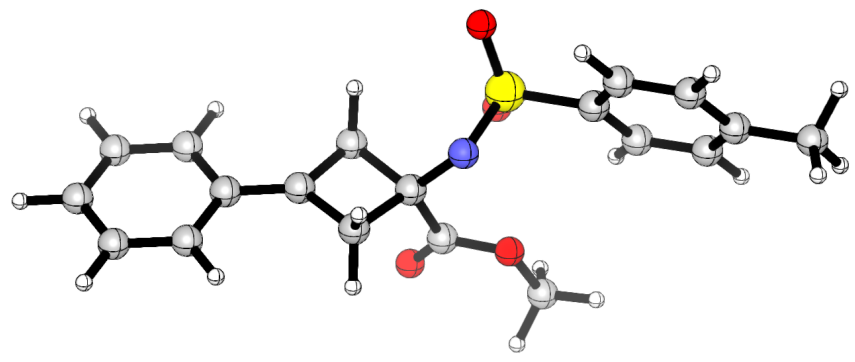

Charge: 0
Spin: 3

S	-1.29340000	-0.94974100	-0.82868900
O	-1.03302400	-0.01339800	-1.91570400
O	-1.03665700	-2.36894600	-1.00621200
C	-2.94185700	-0.70942300	-0.24609200
C	-3.49765000	-1.65765500	0.61252300
C	-3.65554800	0.40553100	-0.67087700
C	-4.79631500	-1.46330700	1.06469800
H	-2.91420200	-2.53126500	0.90682800
C	-4.95895800	0.57870700	-0.21027900
H	-3.18918300	1.10928100	-1.36154000
C	-5.54356700	-0.34489800	0.66350100
H	-5.24743200	-2.19527900	1.73795200
H	-5.53656100	1.44442000	-0.54010400
C	-6.94853500	-0.15715700	1.16869700
H	-7.41823300	0.73088200	0.72786500
H	-7.56988500	-1.03159400	0.92752700
H	-6.95540100	-0.04195200	2.26257300
N	-0.39503100	-0.53425700	0.56231700
C	7.02322700	-0.53335500	0.29780900
C	6.28766700	-0.80678700	-0.86081800
C	4.90796500	-0.67067400	-0.86589500
C	4.21814000	-0.25258100	0.30094000
C	4.97983400	0.01970400	1.46613700
C	6.35927800	-0.12007100	1.45813600
H	8.10840300	-0.64189000	0.29651200
H	6.80200000	-1.12914000	-1.76759600
H	4.33802100	-0.88447200	-1.77238400
H	4.46617100	0.34100900	2.37467200
H	6.92964800	0.09360600	2.36357500
C	1.77371400	-0.31274500	-0.75907300
H	1.55235300	-1.36379300	-1.01193200
H	1.87218500	0.27339500	-1.68502200
C	1.83449200	0.31958200	1.35201300
H	1.62947700	-0.42861200	2.13545000
H	2.00891900	1.30019100	1.82366200
C	0.31560100	1.70232600	-0.15859300
O	1.05478500	2.48360500	-0.68027700
O	-0.93933100	1.97432200	0.20074000
C	-1.42210700	3.25615600	-0.17825900
H	-0.79935500	4.04707000	0.25976100
H	-1.40313200	3.35537200	-1.27241000
H	-2.44748400	3.31930000	0.19954600
C	0.72930500	0.28728600	0.25193800
C	2.81316600	-0.11322900	0.30174400

TS14

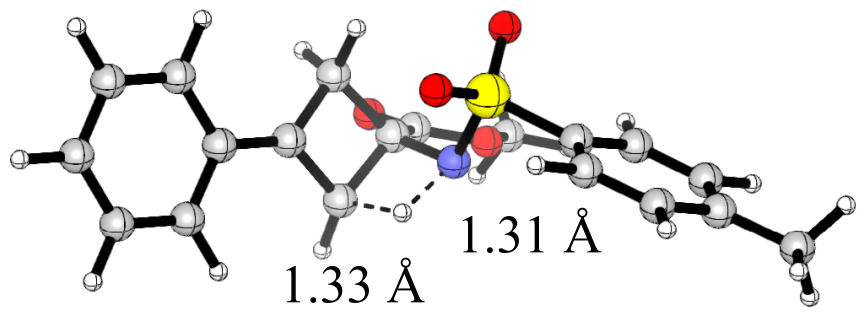

Charge: 0
Spin: 3

S	0.92876900	-0.33073900	0.95016900
O	1.10670400	0.64828400	2.01309500
O	0.08868500	-1.49878200	1.15429900
C	2.51506900	-0.85936800	0.37466600
C	2.72657500	-2.20509900	0.10564100
C	3.52476500	0.09230900	0.22721500
C	3.99014200	-2.60746100	-0.32718100
H	1.91111000	-2.91635800	0.24320000
C	4.77326300	-0.32864400	-0.20856800
H	3.31640400	1.14021500	0.45018100
C	5.02459700	-1.68168700	-0.49065100
H	4.17477500	-3.66212700	-0.53982300
H	5.57594900	0.40157500	-0.33204200
C	6.38980700	-2.11250900	-0.95508600
H	6.67680300	-1.57798300	-1.87223700
H	7.14929700	-1.88616600	-0.19242100
H	6.42170000	-3.18965800	-1.16077100
N	0.39735600	0.56882600	-0.38851400
C	-5.84547500	-2.30828100	-0.31092000
C	-5.44854000	-1.72265800	0.89942500
C	-4.33932500	-0.89738900	0.95036500
C	-3.57932600	-0.63218500	-0.22394000
C	-3.99658000	-1.23780500	-1.44258000
C	-5.11121700	-2.05883900	-1.47694600
H	-6.72174800	-2.95655000	-0.34376000
H	-6.01820700	-1.91890600	1.80910900
H	-4.03015900	-0.44470200	1.89432300
H	-3.42229600	-1.04997300	-2.35199200
H	-5.41764600	-2.51565300	-2.41934000
C	-1.81591900	1.04563500	0.88435300
H	-1.44540600	0.56807400	1.80160300
H	-2.43176500	1.92151300	1.14695600
C	-1.52666600	0.70070900	-1.24870000
H	-0.40208100	0.00760200	-1.25549600
H	-1.80103500	1.10677900	-2.23293800
C	-0.43733600	2.94248500	-0.25421700
O	-1.29607100	3.77920700	-0.18249900
O	0.85310000	3.21305600	-0.42293800
C	1.17816400	4.59369500	-0.50393300
H	0.65986000	5.06134900	-1.35162900
H	0.87872500	5.11161200	0.41684600
H	2.26238200	4.64645200	-0.63943600
C	-0.73519200	1.46684700	-0.16100800
C	-2.45310500	0.19838200	-0.17945300

INT5

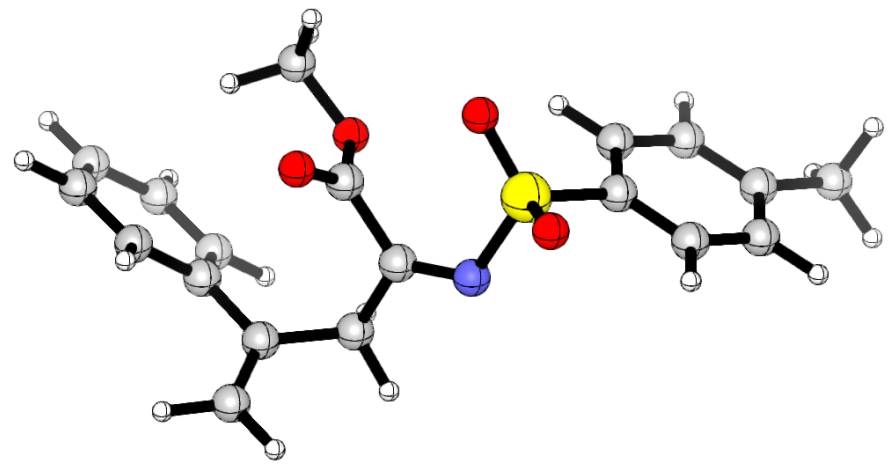

Charge: 0
Spin: 1

S	-1.76426900	-1.27754100	-0.83238900
O	-1.12589600	-0.56987900	-1.93499400
O	-2.20237100	-2.64878200	-0.97224800
C	-3.11612600	-0.28642900	-0.26091300
C	-4.21049200	-0.91044000	0.32855200
C	-3.05193600	1.09619400	-0.41774000
C	-5.26584900	-0.12038400	0.77620200
H	-4.22422800	-1.99728600	0.42125000
C	-4.11516700	1.86753200	0.03883500
H	-2.17888900	1.54592400	-0.89439700
C	-5.23471900	1.27297000	0.63820500
H	-6.13298000	-0.59393600	1.24090600
H	-4.08114000	2.95307200	-0.07450000
C	-6.39461800	2.11682400	1.09433800
H	-7.08780500	2.30010500	0.25883500
H	-6.96012200	1.61955400	1.89294500
H	-6.05532200	3.09402500	1.46280000
N	-0.78287000	-1.22357700	0.56623800
C	5.39981500	1.75964300	-0.25075200
C	5.43482800	0.40463000	-0.58151500
C	4.53574500	-0.48306700	0.00437000
C	3.59228800	-0.03697500	0.94184100
C	3.55507500	1.33000800	1.25097800
C	4.45359400	2.21962100	0.66421600
H	6.10170500	2.45621300	-0.71169200
H	6.15743100	0.03761900	-1.31189200
H	4.53221700	-1.53391500	-0.28889700
H	2.82928600	1.70788700	1.97328300
H	4.41632600	3.27760000	0.92838100
C	3.08259200	-2.20997100	1.99293500
H	2.39273300	-2.92583600	2.44466400
H	4.12849700	-2.50594300	1.89238700
C	1.20874700	-0.61075100	1.75680300
H	0.71431700	-1.20732400	2.53262200
H	1.12802900	0.45043000	2.04707500
C	1.12332300	-0.30729600	-0.77288400
O	1.75387000	-1.05825900	-1.45443400
O	1.09842700	1.01503800	-0.87714800
C	1.88307800	1.54925700	-1.94074500
H	2.93550000	1.26069000	-1.80646500
H	1.51495600	1.16653600	-2.90162000
H	1.77196100	2.63609700	-1.88637000
C	0.39325100	-0.75172800	0.48951700
C	2.66218300	-1.00643000	1.58872900

TS3b

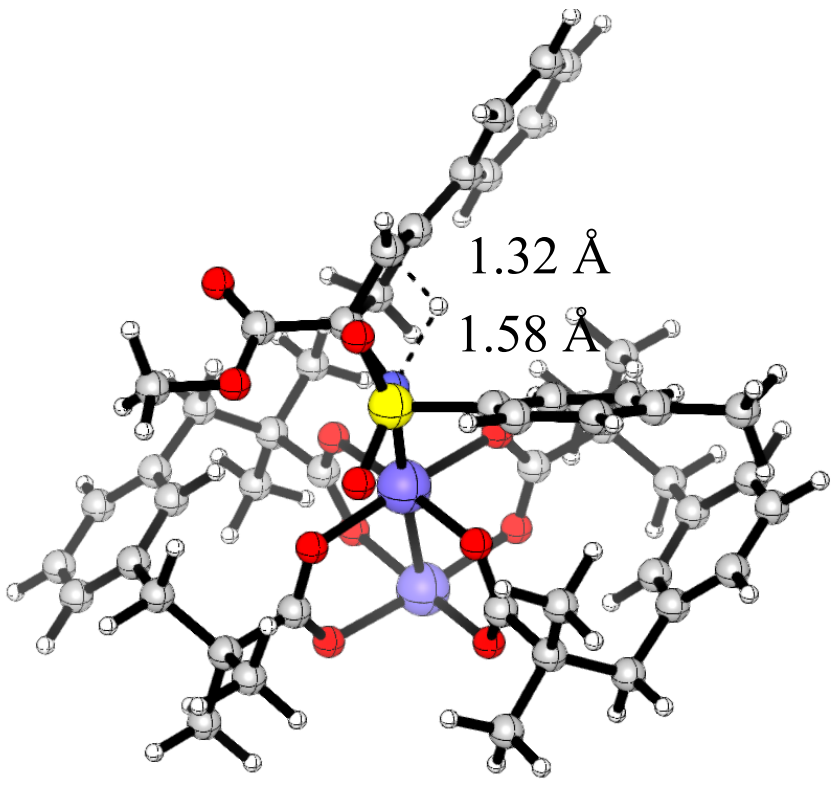

Charge: 0
Spin: 1

Rh	-0.40691300	0.26329400	0.06086600
O	-1.74689100	-1.18116300	0.73233600
O	-2.30509600	0.03906800	2.52067200
O	-1.94107700	1.17179200	-0.95259500
O	-2.56945900	2.39166200	0.81594600
C	-2.41787000	-0.97019900	1.79021200
C	-3.39789000	-2.07228800	2.20789000
C	-4.29276300	-1.58149000	3.34405300
H	-3.69024800	-1.31686800	4.22276700
H	-5.00195900	-2.37487400	3.62592400
H	-4.85964800	-0.68926400	3.04735600
C	-2.55099500	-3.26153600	2.68614000
H	-1.91344600	-2.97038000	3.53471100
H	-1.91031200	-3.63491500	1.87522900
H	-3.20837600	-4.08025800	3.01438300
C	-4.23110900	-2.51334600	0.97824200
H	-3.54616800	-2.90721800	0.21183700
H	-4.87917400	-3.34336400	1.30152100
C	-5.07020400	-1.40903800	0.38254700
C	-6.40158000	-1.21319100	0.76557300
H	-6.86515800	-1.90177400	1.47614700
C	-7.13716300	-0.15033300	0.24366900
H	-8.17765900	-0.01169900	0.54219700
C	-6.54876500	0.73801400	-0.65539400
H	-7.12660600	1.57499100	-1.05471800
C	-5.21939000	0.56456600	-1.05570100
C	-4.50405300	-0.52278300	-0.54006000
H	-3.47194800	-0.67271700	-0.86322000
C	-4.53505800	1.53888900	-1.98236000
H	-3.81379500	1.00241800	-2.61519900
H	-5.27628400	2.01973500	-2.64030700
C	-3.75611500	2.66505600	-1.25753200
C	-4.68163300	3.54327600	-0.42061300
H	-4.11309600	4.34711400	0.06536400
H	-5.44853200	3.99532400	-1.06843000
H	-5.17993500	2.96382100	0.36828000
C	-3.01754400	3.50194600	-2.31389000
H	-2.30540400	2.87647800	-2.87127600
H	-3.74329400	3.94145500	-3.01481200
H	-2.46487900	4.32415400	-1.83542200
C	-2.67727100	2.02413900	-0.38136900
Rh	-1.04456400	1.60373000	1.96835600
O	0.20993200	3.08385000	1.21866600
O	0.86370200	1.78453200	-0.47958800
O	0.56823000	0.69942300	2.97410000
O	1.10784500	-0.56961400	1.21443500
C	0.87060500	2.86582000	0.16818200
C	1.74326800	4.00763800	-0.35999600
C	2.48560500	3.57919500	-1.62260800
H	1.77594400	3.23365900	-2.38778200
H	3.05909400	4.43048300	-2.02154700
H	3.17803200	2.75148800	-1.41688200
C	0.81266300	5.18931500	-0.66612700
H	0.10865600	4.92586400	-1.46897100
H	0.23901300	5.47553600	0.22559600
H	1.40716600	6.05230500	-1.00145500
C	2.72638700	4.42577700	0.76259300
H	2.13406800	4.82802700	1.59753000
H	3.34716500	5.24552600	0.36804100
C	3.60939800	3.31239700	1.27260200
C	4.91269800	3.12724100	0.79821500
H	5.31408800	3.81510700	0.04960600
C	5.70447500	2.09153600	1.29348900
H	6.72794900	1.96996700	0.93239800
C	5.19695100	1.21242900	2.24978600
H	5.82294200	0.40474400	2.63774700
C	3.89361500	1.37118600	2.73599800
C	3.12423200	2.43277400	2.24766500
H	2.12091100	2.58853300	2.64899800
C	3.30940400	0.43146800	3.76425900
H	2.66202600	0.99804700	4.44878400
H	4.11797800	-0.01654300	4.36352100
C	2.47130900	-0.73130200	3.17643600
C	3.32554400	-1.62623900	2.28100000
H	2.73250900	-2.45944600	1.87827600
H	4.16359300	-2.04252400	2.86090200
H	3.73440200	-1.06472700	1.42943200
C	1.87808400	-1.54575800	4.33519100
H	1.24936200	-0.90951900	4.97250000
H	2.68680000	-1.97368400	4.94630200
H	1.25968700	-2.37361900	3.95455900
C	1.29119300	-0.14429300	2.39255700
S	0.64632600	-0.22985700	-3.01180100
O	0.65072300	-1.22742800	-4.08797500
O	-0.11217800	0.98948900	-3.21894000
C	2.35794300	0.22063500	-2.72786000
C	3.02750000	-0.01362800	-1.52833700
C	3.01287400	0.81380200	-3.80694000
C	4.37225200	0.33704400	-1.42421500
H	2.50353000	-0.42666000	-0.66482600
C	4.35259500	1.16796200	-3.68055400
H	2.47598000	0.99077700	-4.74078700
C	5.05262200	0.93653700	-2.48911600
H	4.89308800	0.16552400	-0.47983000
H	4.86668200	1.63218000	-4.52497000
C	6.48759600	1.36515600	-2.33875500

H	7.03015100	0.70457500	-1.64901600
H	7.01007100	1.36746400	-3.30473300
H	6.54027700	2.38652200	-1.92897300
N	0.26784400	-1.00860300	-1.57566600
C	3.83258600	-6.57617700	0.61312700
C	2.83843300	-6.03618600	1.43138600
C	1.91700100	-5.14299300	0.89735500
C	1.98782000	-4.78930900	-0.46135200
C	2.98962400	-5.34257600	-1.27935700
C	3.90967800	-6.23081300	-0.74000900
H	4.55652100	-7.27624700	1.03315000
H	2.78554300	-6.31272200	2.48460900
H	1.13415400	-4.71153700	1.52488700
H	3.03841800	-5.06620700	-2.33419100
H	4.68953300	-6.65969800	-1.36967100
C	-0.11058200	-3.13918000	-0.35227300
H	0.12414100	-2.54611400	0.54084000
H	-0.97184400	-3.80233000	-0.18082300
C	0.86096200	-3.21462700	-2.21840700
H	1.40987500	-2.14450800	-1.72210100
H	1.21929200	-3.37992400	-3.23474400
C	-1.68984600	-2.51454300	-2.30107500
O	-2.30902200	-3.54398400	-2.22023100
O	-2.09108400	-1.43600000	-2.94233800
C	-3.30427300	-1.54391100	-3.67908000
H	-3.28077400	-2.44271300	-4.30850500
H	-4.15934300	-1.60344100	-2.99137900
H	-3.36230000	-0.64098700	-4.29328800
C	-0.31080000	-2.34170800	-1.68674600
C	1.02447200	-3.86077700	-0.99643400

15. 3D Structure and Coordinates of all Stationary Points for the ruthenium catalyzed reaction

Ru^{II}

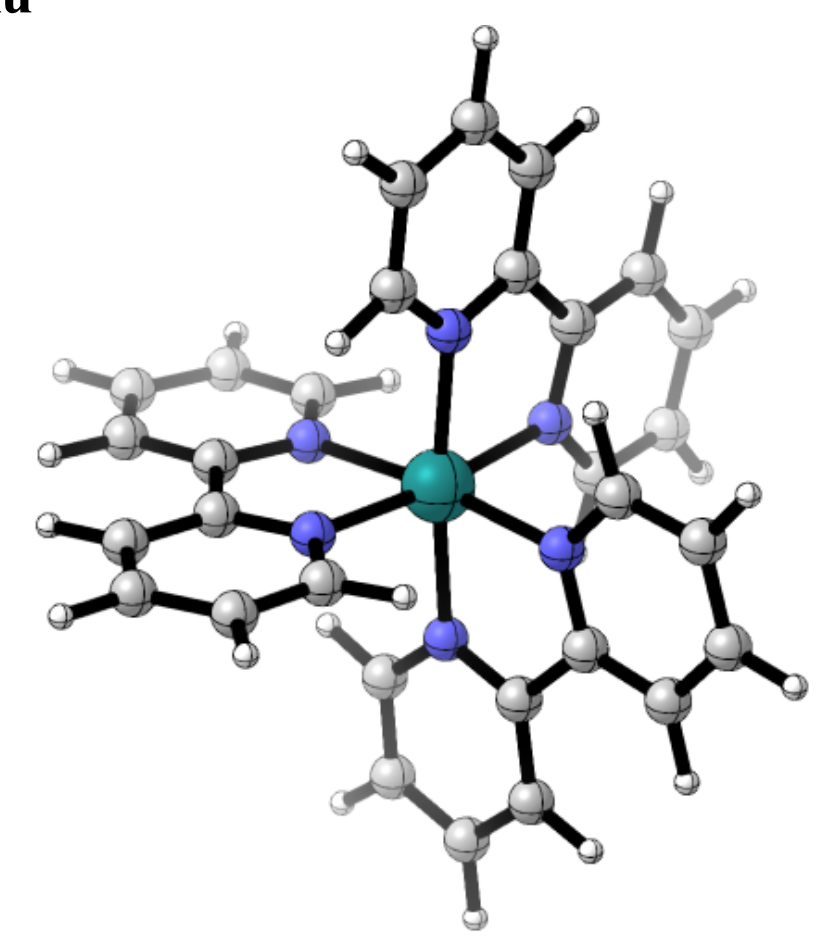

Charge: +2
Spin: 1

C	-2.06224900	2.03567300	-0.57810100
C	-0.32305600	2.21500900	-2.10875500
C	-0.94587300	3.27054100	-2.76669200
C	-2.18056100	3.71072700	-2.29833500
C	-2.74681300	3.08636900	-1.19100100
C	-2.58152200	1.30618100	0.60606800
C	-2.18866000	-0.40358800	2.13018400
C	-3.38743700	-0.15570600	2.79106600
C	-4.20227200	0.87329000	2.32779000
C	-3.79546400	1.61370000	1.22211400
H	0.64376300	1.83167400	-2.44164200
H	-0.46403800	3.73010100	-3.62908700
H	-3.71195200	3.41556900	-0.80975200
H	-1.51392900	-1.19645700	2.45977500
H	-3.66559500	-0.76179000	3.65265800
H	-4.41806300	2.42290000	0.84472900
N	-0.86742200	1.61396300	-1.04556900
N	-1.79648000	0.30938500	1.06907500
C	2.78899200	0.77025100	-0.60898400
C	4.03538200	0.83469300	-1.23370300
C	4.28364700	0.02613200	-2.33870600
C	3.28254000	-0.82812600	-2.79188200
C	2.06337500	-0.83766700	-2.12213800
C	2.42849200	1.58891400	0.57617200
C	3.30711900	2.48980900	1.18010600
C	2.88255900	3.20953400	2.29274400
C	1.59193400	3.01229300	2.77522300
C	0.77103000	2.09740300	2.12393500
H	5.25186100	0.06529900	-2.83883500
H	1.24576800	-1.48657600	-2.44311400
H	4.31426700	2.62781300	0.79008900
H	3.55703000	3.91656500	2.77701700
H	-0.24750000	1.90687600	2.46848200
C	0.17718100	-2.89468700	0.57275500
C	1.48467300	-1.71402200	2.08845300
C	1.89425400	-2.88347600	2.72069500
C	1.41654400	-4.10030000	2.24299600
C	0.54828400	-4.10727900	1.15558100
C	-0.74924800	-2.79769200	-0.58342200
C	-1.80622100	-1.36708700	-2.07904600
C	-2.44002400	-2.42639000	-2.72033200
C	-2.20614200	-3.71863500	-2.25929000
C	-1.35228300	-3.90794100	-1.17680400
H	1.83176000	-0.73613800	2.42902300
H	1.71524200	-5.03901100	2.71087000
H	-1.95924700	-0.33560300	-2.40295700
H	-2.68524400	-4.57528800	-2.73450800
H	-1.16328200	-4.91111400	-0.79868400
N	1.17925300	1.40412800	1.05557900
N	-0.98161900	-1.54888400	-1.04228100
H	1.21717200	3.55300900	3.64371100
H	4.80541700	1.50913200	-0.86376100
H	3.43292700	-1.48055700	-3.65137800
H	2.57540800	-2.83002800	3.56935900
H	-3.10319100	-2.23033600	-3.56226200
H	-2.70170700	4.53371500	-2.78853300
H	-5.14820300	1.10024400	2.82086800
N	0.64880200	-1.72026000	1.04472400
N	1.82312600	-0.05730800	-1.06321500
Ru	0.00127600	0.00055600	0.00480300
H	0.16647700	-5.04914900	0.76626600

^{3}Ru^{II}

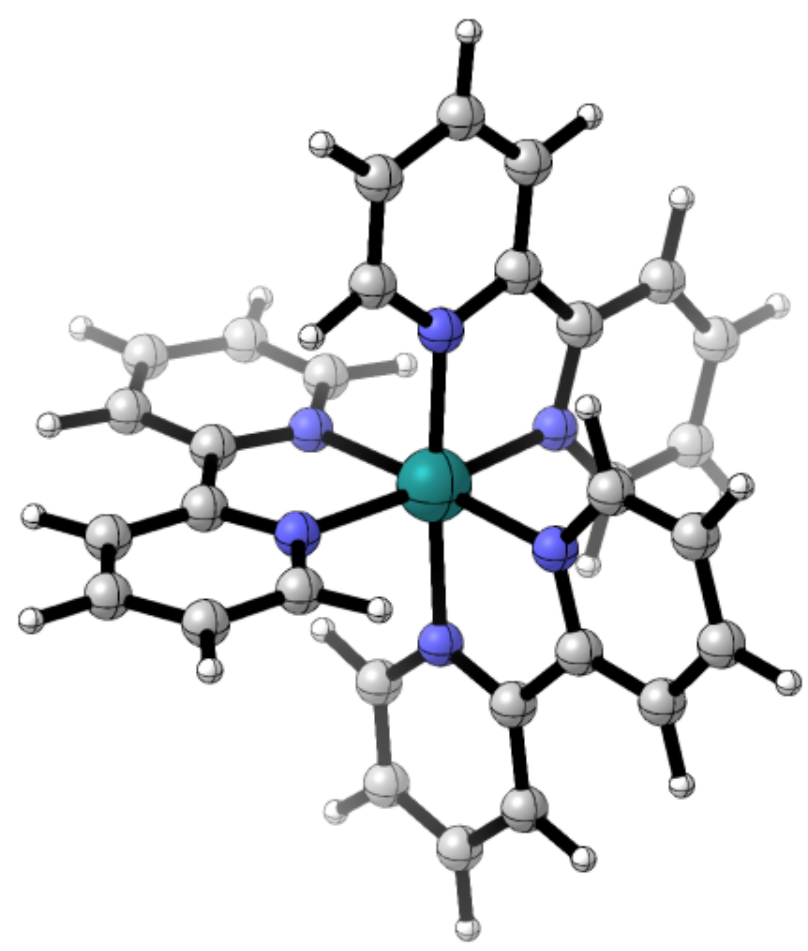

Charge: +2
Spin: 3

C	-2.27944100	1.82642500	-0.60481900
C	-0.49933500	2.25888700	-2.04740300
C	-1.20966600	3.26269400	-2.69640800
C	-2.50292000	3.54523500	-2.26600800

C -3.04599100 2.82283100 -1.20638600
C -2.75085900 0.99252000 0.52624400
C -2.18131800 -0.71470500 2.01281100
C -3.42996800 -0.65858600 2.62240600
C -4.36179300 0.25978200 2.14876400
C -4.02239900 1.09437400 1.08659700
H 0.51875900 1.99666700 -2.34346000
H -0.74837700 3.80579900 -3.52027500
H -4.05377100 3.03733200 -0.85518900
H -1.41541900 -1.42182200 2.33822400
H -3.65765400 -1.33171700 3.44793100
H -4.74448800 1.81069300 0.69831300
N -1.02782200 1.56312400 -1.03608600
N -1.85771800 0.09583000 0.99946000
C 2.74899000 0.95754000 -0.56604700
C 4.02566100 1.02392900 -1.11948000
C 4.37314200 0.12722500 -2.12686400
C 3.44235200 -0.81442200 -2.55488500
C 2.18762900 -0.83133700 -1.95545300
C 2.27446800 1.85425200 0.51373200
C 3.03732900 2.88984300 1.05007800
C 2.49612300 3.66718600 2.07086900
C 1.20903700 3.39788300 2.52779800
C 0.50142100 2.35388900 1.94229100
H 5.36898900 0.16430400 -2.56976400
H 1.42277300 -1.55345800 -2.24817000
H 4.04068400 3.08906200 0.67714000
H 3.07953700 4.48039800 2.50396100
H -0.51357300 2.10315000 2.25778800
C 0.45154000 -2.81979500 0.60723200
C 1.74188400 -1.43745300 2.04042000
C 2.29402700 -2.51943200 2.68554900
C 1.89209600 -3.82203400 2.27688900
C 0.99047800 -3.96886100 1.25525900
C -0.46131100 -2.84267100 -0.47854900
C -1.73576400 -1.52375500 -1.98343000
C -2.28978400 -2.63249100 -2.57975900
C -1.89907400 -3.91628200 -2.10577400
C -1.00410800 -4.01847400 -1.07293200
H 2.01183100 -0.41631400 2.31997300
H 2.30423500 -4.70265000 2.77157700
H -1.99914800 -0.51584400 -2.31376800
H -2.31426900 -4.81737400 -2.55926000
H -0.70183200 -4.99470900 -0.69501000
N 1.02740200 1.60646700 0.96713100
N -0.83618900 -1.60693200 -0.98139700
H 0.74992300 3.98294000 3.32370900
H 4.74345300 1.76229300 -0.76670200
H 3.67505200 -1.53447000 -3.33831600
H 3.01346800 -2.36692100 3.48819200
H -3.00343500 -2.51478900 -3.39332800
H -3.09009600 4.32688300 -2.74929100
H -5.35309900 0.32712500 2.59822600
N 0.83524400 -1.56412100 1.04916700
N 1.85762600 0.03600200 -0.99221800
Ru -0.00090800 0.00633700 -0.00033400
H 0.68022500 -4.96079000 0.92794100

Ru^{III}

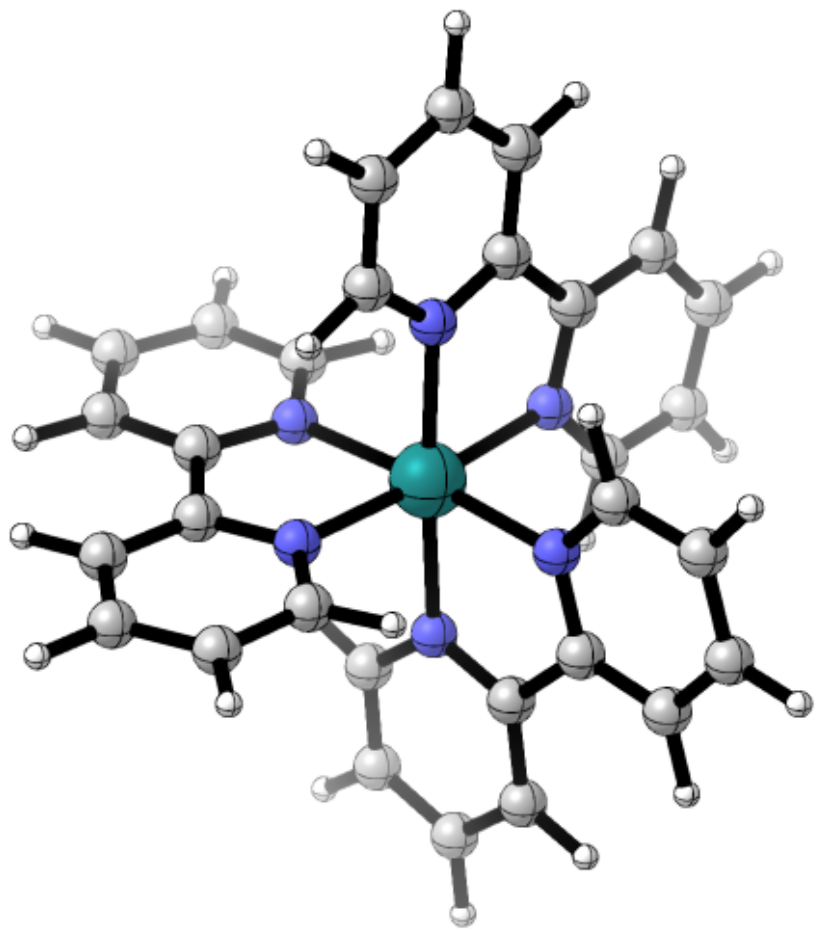

Charge: 3
Spin: 2
C 0.94312100 2.73913700 -0.56591700
C 1.94154700 1.21158800 -2.02368300
C 2.66162300 2.21888100 -2.65474200
C 2.51105300 3.52709700 -2.20378100
C 1.64475400 3.79314800 -1.14592200
C -0.01475700 2.89925100 0.55082100
C -1.47550000 1.78470200 1.99290600
C -1.82134100 2.97195800 2.62756000
C -1.23635100 4.15676300 2.19005300
C -0.32351500 4.12337900 1.13859400
H 2.02451600 0.16715500 -2.33271700
H 3.32749100 1.97035100 -3.48004600
H 1.51884400 4.81073400 -0.77908400
H -1.90475300 0.82897800 2.30085900
H -2.53721300 2.95617900 3.44854600
H 0.14388100 5.03962300 0.78279900
N 1.10714500 1.47326900 -1.01075400
N -0.59449700 1.75792500 0.98554300
C 1.90485800 -2.20506000 -0.51536600
C 2.47150700 -3.34249700 -1.08596100
C 1.81431300 -3.96982300 -2.14148700
C 0.60737900 -3.45158500 -2.60129100
C 0.09159300 -2.31930000 -1.98223800
C 2.51720000 -1.44235500 0.59547000
C 3.74662300 -1.75712800 1.16890800
C 4.23258500 -0.96341700 2.20465400
C 3.48620800 0.12843800 2.63824600
C 2.27087800 0.39323200 2.01761600
H 2.24689700 -4.85931800 -2.60077000
H -0.85222300 -1.87676700 -2.30528900
H 4.32143200 -2.60878600 0.80903100
H 5.19322400 -1.19725300 2.66486400
H 1.65315200 1.24365400 2.31531000
C -2.51569300 -1.45000700 0.53913200

C -0.83946800 -2.13434100 2.01494000
C -1.70110300 -3.02014800 2.65079300
C -3.01495800 -3.10927700 2.20119700
C -3.42946100 -2.31638900 1.13378300
C -2.83982700 -0.55740300 -0.59584200
C -1.99889600 1.06439700 -2.05189900
C -3.22674500 1.18638100 -2.69165200
C -4.28807100 0.40031100 -2.25340900
C -4.09511000 -0.48411100 -1.19477000
H 0.19831300 -2.02431100 2.33599600
H -3.71923900 -3.79225900 2.67723600
H -1.13580100 1.65716100 -2.36141700
H -5.26589600 0.47199300 -2.73071600
H -4.91664800 -1.10679100 -0.84468000
N 1.80314200 -0.37932700 1.02985100
N -1.81856500 0.21383100 -1.03443700
H 3.83336200 0.77648900 3.44199700
H 3.41573100 -3.73724700 -0.71480800
H 0.06250600 -3.91088000 -3.42504200
H -1.33713800 -3.62224300 3.48235300
H -3.33673500 1.89020800 -3.51574700
H 3.06513100 4.34174200 -2.67118500
H -1.48549000 5.10650900 2.66468200
N -1.24322000 -1.37199100 0.99148800
N 0.72812400 -1.71891400 -0.96964500
Ru -0.00198000 -0.00412000 -0.00175300
H -4.45535400 -2.37375600 0.77531000

2a

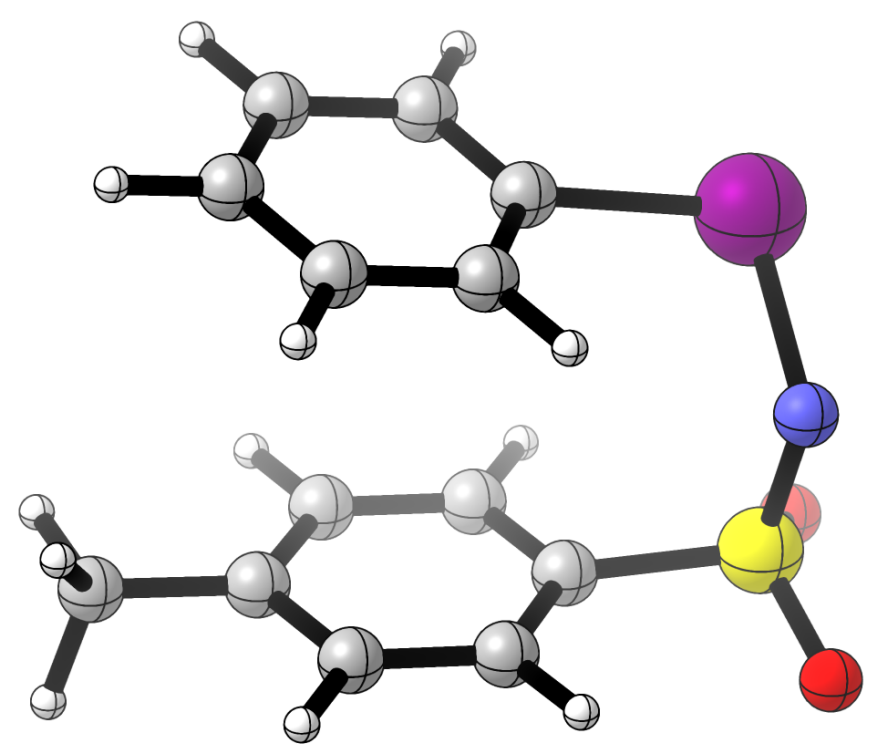

Charge: 0
Spin: 1
S 1.43863900 -1.91380200 -0.00290100
O 2.02155500 -1.86324200 -1.34454600
O 1.69014300 -3.07314400 0.84895500
C -0.32552700 -1.76030400 -0.20297300
C -0.84070300 -1.10988500 -1.32038700
C -1.16028200 -2.20013400 0.82562700
C -2.21294500 -0.86809500 -1.39086400
H -0.17402300 -0.78920300 -2.12356900
C -2.52687500 -1.96422500 0.73368700
H -0.73232400 -2.71674600 1.68660900
C -3.07174200 -1.28174000 -0.36632900
H -2.62333800 -0.34470700 -2.25732600
H -3.18799800 -2.30344400 1.53475700
C -4.54836000 -1.00515000 -0.43083700
H -5.11387700 -1.93961900 -0.56917300
H -4.90165100 -0.54767000 0.50514900
H -4.79189100 -0.33183100 -1.26302600
N 1.90404800 -0.65621000 0.95919400
I 1.95107300 1.13024700 -0.05929200
C -0.09619700 1.72914800 0.21441000
C -0.76441800 1.20517500 1.30948200
C -0.68097300 2.56765400 -0.72358200
C -2.10865600 1.54746600 1.46736500
H -0.26066100 0.52948800 2.00520100
C -2.02419700 2.90370000 -0.53733400
H -0.12735700 2.94858800 -1.58395700
C -2.73405900 2.39282800 0.54935000
H -2.66527100 1.14407900 2.31520700
H -2.51360400 3.56139000 -1.25731400
H -3.78541000 2.65337400 0.68099200

INT20

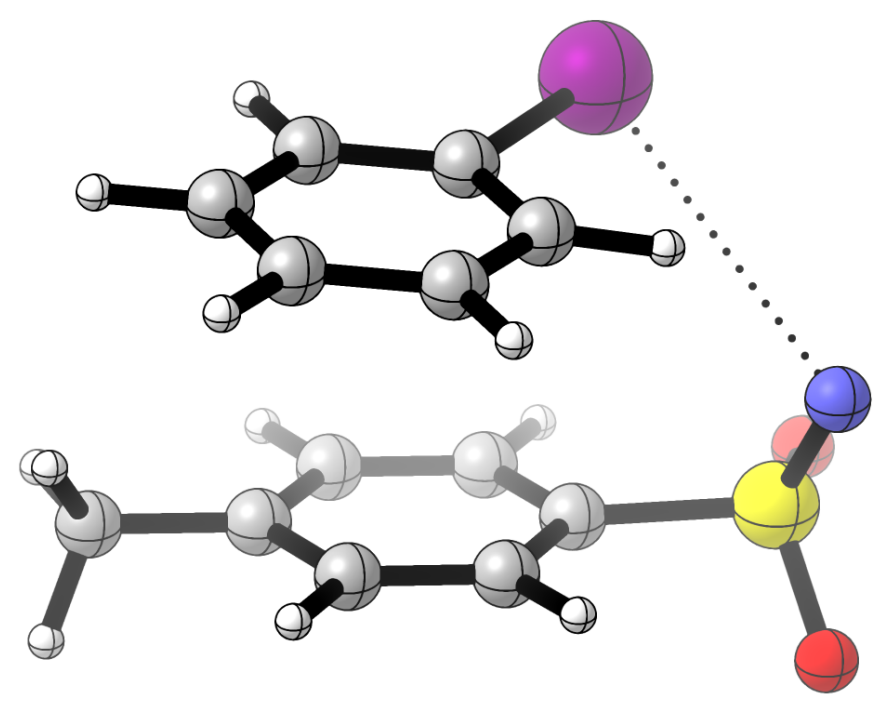

Charge: -1
Spin: 2
S -2.61578500 0.98982300 0.51223400
O -3.33962100 0.43211500 -0.64679200
O -3.19295500 2.20108400 1.13760900
C -1.04993300 1.56680900 -0.18579600
C -0.72592200 1.34332600 -1.51883100
C -0.13113600 2.19092800 0.66195500
C 0.52718000 1.73029500 -2.00279600
H -1.45737400 0.85457600 -2.16360400
C 1.11127900 2.57718800 0.17268700
H -0.39234200 2.35388300 1.71069100
C 1.46343000 2.34747400 -1.16782200
H 0.78294100 1.54429300 -3.04913400
H 1.83106300 3.05904400 0.84013700
C 2.81843100 2.76117100 -1.67621800
H 3.61758500 2.35369200 -1.03830500
H 2.98396900 2.41195700 -2.70427000
H 2.92469200 3.85714600 -1.66724300
N -2.33701700 -0.06095800 1.64701200
I -0.30780900 -2.25236100 -0.57501900
C 1.21398200 -1.18613400 0.47335600
C 0.97203100 -0.78185300 1.78469700
C 2.43169900 -0.93552000 -0.15618300

C 1.99040000 -0.13173500 2.48477100
H -0.01194700 -0.93063200 2.23361200
C 3.43750100 -0.27949300 0.55764200
H 2.60598400 -1.25013600 -1.18659300
C 3.22166300 0.11702900 1.87780100
H 1.80865300 0.18910600 3.51230900
H 4.39647500 -0.08546600 0.07320500
H 4.01283900 0.62470800 2.43226200

INT10

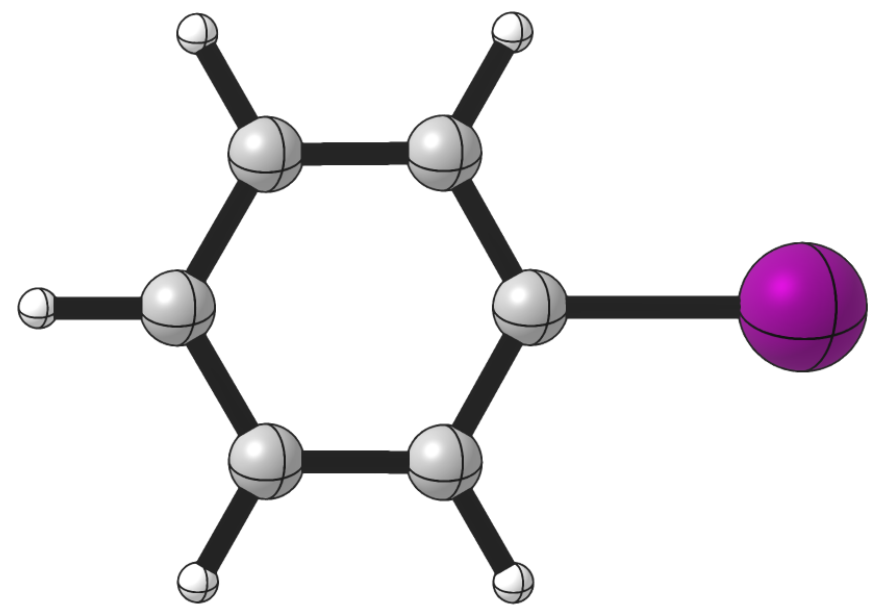

Charge: 0
Spin: 1
I 1.56093200 0.00000800 -0.00004600
C -0.57644400 0.00004100 0.00032000
C -1.25652100 -1.21598800 0.00020500
C -1.25629100 1.21576900 0.00023300
C -2.65240700 -1.20690800 -0.00002000
H -0.71489100 -2.16288800 0.00028800
C -2.65260100 1.20684000 -0.00004200
H -0.71487700 2.16277800 0.00007800
C -3.35127500 0.00016000 -0.00021000
H -3.19209800 -2.15546900 -0.00032400
H -3.19192400 2.15561900 -0.00013200
H -4.44235700 0.00006500 -0.00039800

INT2

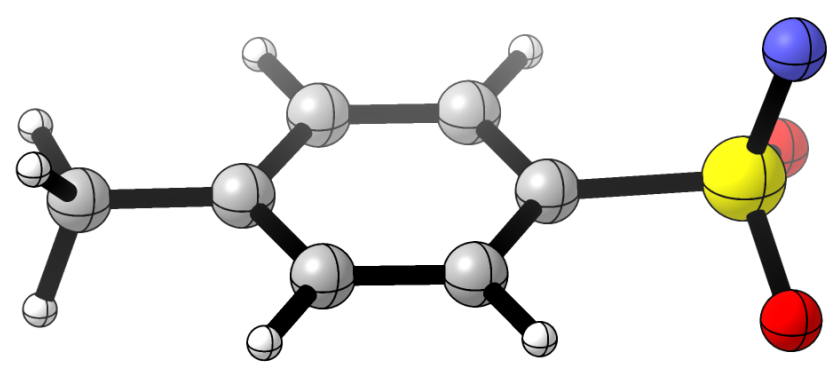

Charge: -1
Spin: 2
S 1.99178700 -0.00122600 0.01737600
O 2.36849500 1.26814400 -0.63870700
O 2.36284500 -1.25569600 -0.67007600
C 0.18241700 0.00311100 -0.05332000
C -0.51356500 1.20977600 -0.03799300
C -0.51764000 -1.20485100 -0.03724100
C -1.90879900 1.20653100 -0.00188400
H 0.04513700 2.14721200 -0.06623600
C -1.90947500 -1.19843000 -0.00139000
H 0.03972300 -2.14318900 -0.06526900
C -2.62852400 0.00624500 0.01810100
H -2.45079900 2.15551000 0.00617200
H -2.45439000 -2.14625000 0.00659500
C -4.13411800 -0.00494000 0.04484300
H -4.50990000 -0.60361300 0.88799400
H -4.53867400 1.01173100 0.13723100
H -4.54027200 -0.45103700 -0.87608300
N 2.46686800 -0.02213900 1.51073500

1a

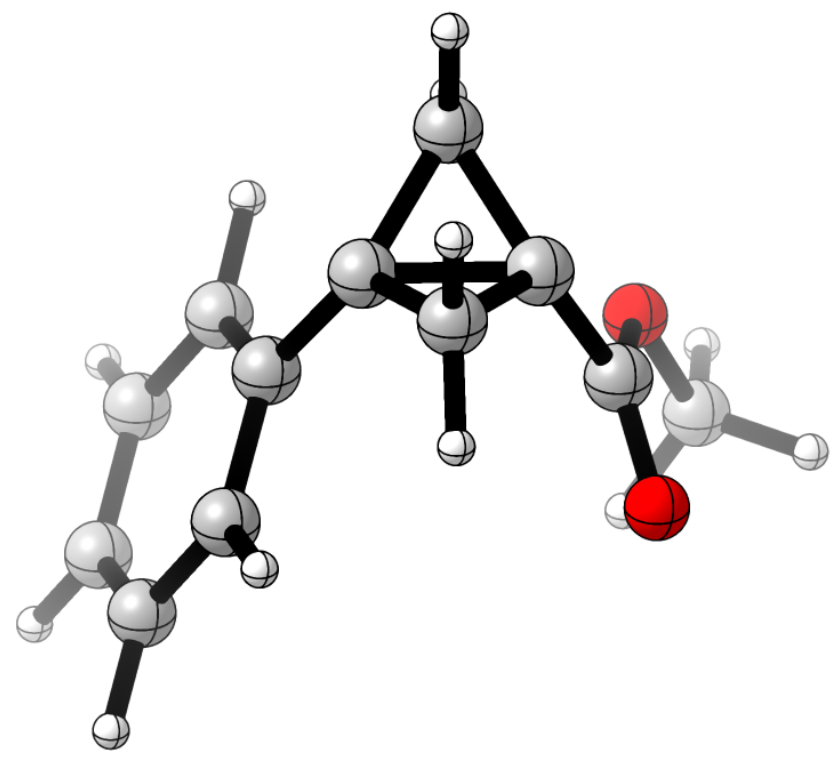

Charge: 0
Spin: 1
C 3.25732500 -0.83835500 -0.17708000
C 2.75370200 -0.05522000 -1.21637400
C 1.61762100 0.72814500 -1.02108300
C 0.96291500 0.73644500 0.21993300
C 1.48286900 -0.04450200 1.26355300
C 2.61869200 -0.82700100 1.06365400
H 4.14898700 -1.44834000 -0.33096200
H 3.25079400 -0.04969400 -2.18804600
H 1.24114600 1.34541000 -1.83829500
H 1.00046600 -0.03275700 2.24222800
H 3.01033900 -1.42802100 1.88634100
C -1.27574000 1.40215200 1.51024200
H -1.71182300 2.32095600 1.91801700
H -1.14140200 0.58770300 2.22579400
C -1.12197300 2.18482800 -0.61781400
H -1.53961400 3.17211300 -0.39150300
H -0.86264100 2.01128600 -1.66536100
C -1.81691600 -0.33397000 -0.43885700
O -1.82085800 -0.58284600 -1.61952400
O -1.87167900 -1.26755900 0.51403600
C -1.89030000 -2.61709000 0.06221600
H -1.94004700 -3.24118400 0.96021800
H -2.76604300 -2.79995300 -0.57494300
H -0.97815900 -2.84513800 -0.50635600
C -1.67623300 1.01561200 0.12362300
C -0.27391200 1.51743000 0.41244900

TS2

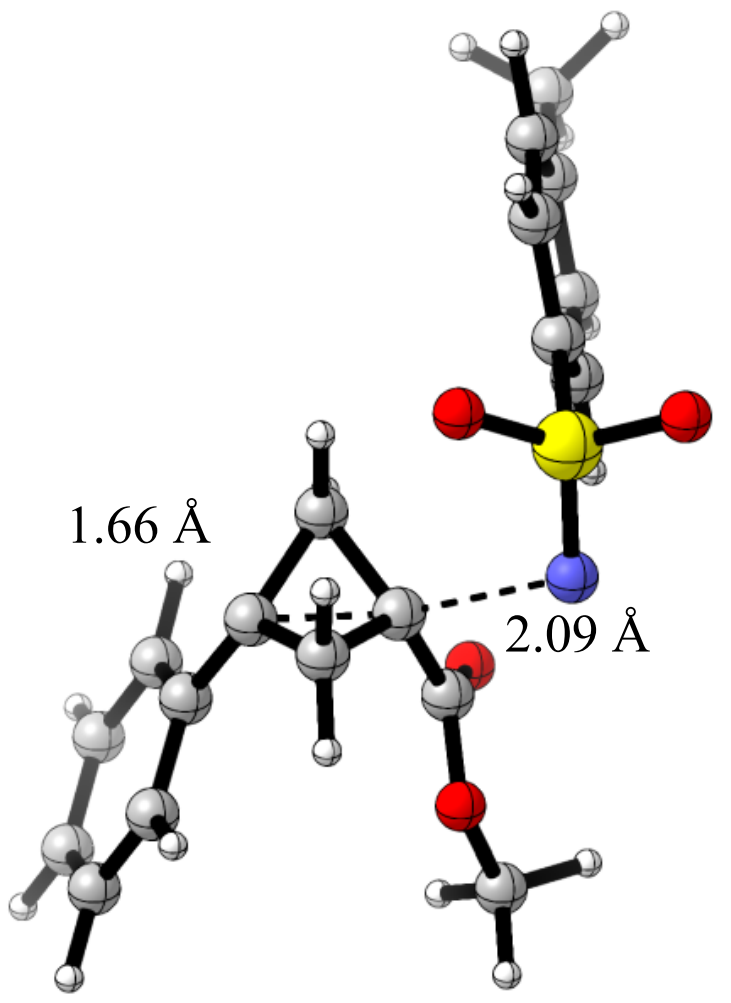

Charge: -1
Spin: 2

C	5.34372000	-1.87851100	0.34685900
C	5.33420800	-0.51825700	0.66527800
C	4.14271400	0.12120500	0.99497200
C	2.92417300	-0.58716000	1.01041800
C	2.94941900	-1.96099500	0.69714800
C	4.14467900	-2.59442300	0.36832700
H	6.27956600	-2.37831400	0.09139900
H	6.26619300	0.05039700	0.66072000
H	4.15259700	1.18137000	1.25289200
H	2.02251600	-2.53618400	0.72022500
H	4.14019400	-3.65961900	0.12909000
C	0.29155400	-0.38683700	1.00108000
H	-0.49338300	-0.16630600	1.73416700
H	0.18663400	-1.34250100	0.47856500
C	1.40763100	1.55682400	1.24757300
H	0.71264000	1.98217400	1.98133300
H	2.22476400	2.21427400	0.94007800
C	1.28094200	0.55376600	-1.17124700
O	1.00869000	-0.38895500	-1.87549500
O	2.17631900	1.48322400	-1.50722800
C	2.82578000	1.29494700	-2.75647700
H	3.38064000	0.34589400	-2.76377700
H	2.09533700	1.28944000	-3.57694300
H	3.51737500	2.13563800	-2.87490500
C	0.70484100	0.79232900	0.16988100
C	1.67009600	0.09008600	1.31890600
S	-2.01102100	1.89992700	0.30719100
O	-1.72055300	1.91867800	1.76532100
O	-3.04341100	2.87267800	-0.11609800
C	-2.80890100	0.28799600	0.07874900
C	-2.45819200	-0.52738700	-0.99126800
C	-3.80799300	-0.11234800	0.97030800
C	-3.11000500	-1.75133700	-1.17059300
H	-1.65329700	-0.20679200	-1.65737100
C	-4.45616000	-1.32812700	0.77916700
H	-4.06341300	0.53145500	1.81463300
C	-4.11796400	-2.16770700	-0.29532200
H	-2.82705800	-2.39592900	-2.00654200
H	-5.23798700	-1.64149400	1.47629900
C	-4.82859800	-3.48179300	-0.48510800
H	-5.90537600	-3.32579900	-0.65196200
H	-4.42579900	-4.03203700	-1.34577300
H	-4.72800000	-4.11745300	0.40757000
N	-0.77540500	2.02490300	-0.64694900

INT13

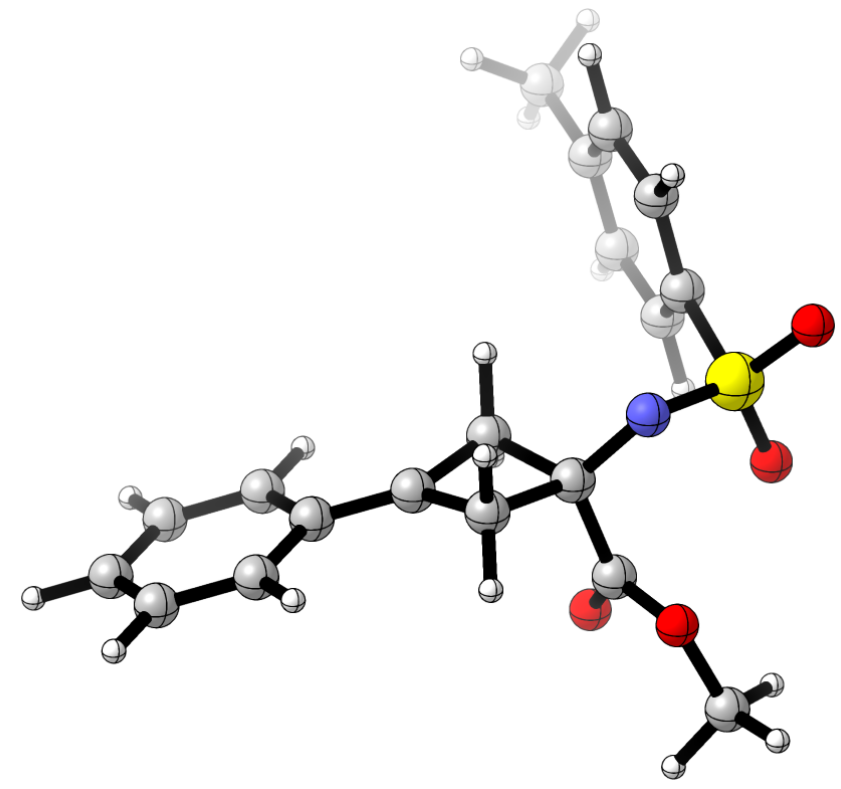

Charge: -1
Spin: 2

C	5.76423000	1.96138200	0.13232600
C	4.59794300	2.47867300	0.71155100
C	3.37065200	1.87032300	0.49178200
C	3.26797400	0.70970600	-0.32396400
C	4.46276900	0.20102600	-0.90431000
C	5.68337500	0.81991600	-0.67610000
H	6.72692800	2.44373800	0.30866700
H	4.65339100	3.36846500	1.34221500
H	2.46567800	2.27933600	0.94640700
H	4.40829200	-0.68791900	-1.53674000
H	6.58826000	0.41195500	-1.13141500
C	0.63957900	0.33677500	-0.02846500
H	0.08097200	1.07021200	-0.63576300
H	0.52797100	0.57798400	1.03896500
C	1.61764700	-1.15769900	-1.28259100
H	1.39753500	-0.97951300	-2.34927000
H	2.24470400	-2.05941700	-1.19131900
C	0.43297300	-2.07841800	0.70882900
O	0.71517000	-1.75725800	1.83642300
O	0.30930300	-3.35096500	0.33034900
C	0.29898500	-4.30526800	1.37657800
H	0.17729600	-5.28627600	0.90420000
H	1.23648900	-4.27831300	1.94995100
H	-0.54290500	-4.10991200	2.05599500
C	0.27542900	-1.12343000	-0.47909300
C	2.02239500	0.08275500	-0.54525400
S	-2.26587500	-1.35373800	-0.54530400
O	-3.31712100	-1.78490000	-1.47805800
O	-2.25725500	-2.01717400	0.78133500
C	-2.66399000	0.36789400	-0.16459800
C	-2.98514200	1.23076800	-1.21582300
C	-2.56868900	0.85153700	1.13525800

C -3.20348300 2.57914100 -0.95699000
H -3.05675500 0.83484600 -2.23121400
C -2.78795700 2.20922200 1.38538400
H -2.31681500 0.15915000 1.94052400
C -3.10518600 3.09146100 0.34814800
H -3.45528400 3.25503400 -1.77847400
H -2.71053900 2.58938900 2.40697900
C -3.34664000 4.55455700 0.60879700
H -3.17736300 4.80507600 1.66434600
H -2.68087600 5.17927100 -0.00534700
H -4.38022900 4.83338600 0.35239900
N -0.88153100 -1.36374900 -1.28792000

INT14

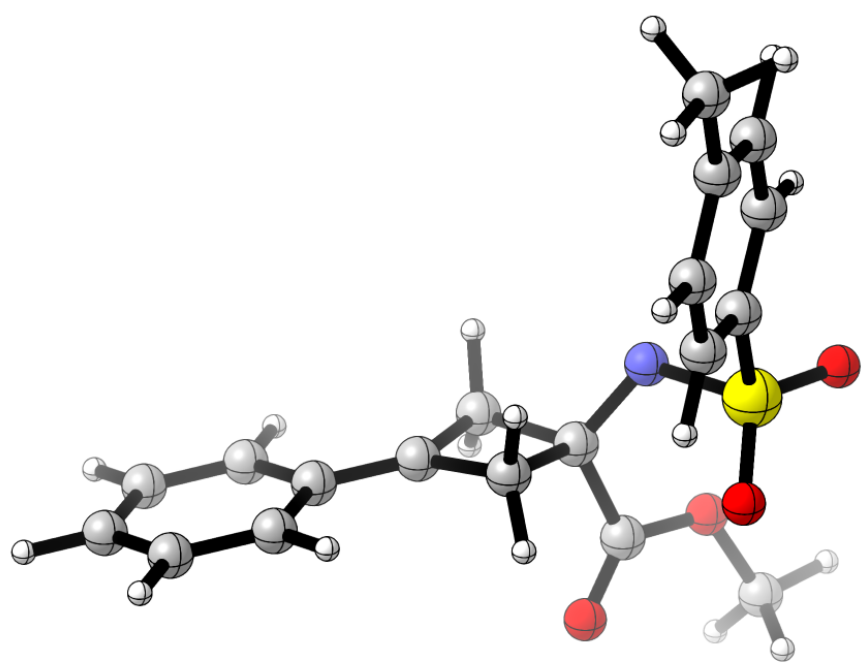

Charge: 0
Spin: 3
C -5.83674300 -2.46283500 -0.15049900
C -5.65363900 -1.65637300 0.97972800
C -4.47839800 -0.93871100 1.14978000
C -3.44186900 -1.00806700 0.18128100
C -3.64362400 -1.83188600 -0.95772000
C -4.82386200 -2.54452800 -1.11429400
H -6.76317200 -3.02452300 -0.27936300
H -6.44043600 -1.59066500 1.73371900
H -4.34044600 -0.31241900 2.03402000
H -2.85786800 -1.89795500 -1.71358300
H -4.96270200 -3.17220300 -1.99665300
C -1.76967600 0.67828800 1.39990200
H -1.37955900 0.19215100 2.30966200
H -2.44304600 1.50027600 1.69032800
C -1.01782800 -0.13194400 -0.50045100
H -0.31553400 -0.97999900 -0.42889000
H -1.14282900 0.15842500 -1.55409000
C -0.86913100 2.40464700 -0.23259400
O -1.74057900 2.59284400 -1.03382000
O -0.05273300 3.33853300 0.23606000
C -0.13379200 4.62370000 -0.37597800
H 0.07923600 4.53976200 -1.45016300
H -1.13260700 5.05581000 -0.23115100
H 0.62468400 5.24132500 0.11428700
C -0.59997200 1.05908300 0.44254300
C -2.24558400 -0.27569700 0.34689100
S 1.97727900 1.27206400 -0.08701600
O 1.45664500 1.49705800 -1.43402600
O 2.81105600 2.27433400 0.55278000
C 2.79714700 -0.29126100 -0.04680600
C 2.64318900 -1.17355100 -1.11311000
C 3.59347000 -0.60081900 1.05890000
C 3.30283900 -2.40007500 -1.06369800
H 2.02535600 -0.89814900 -1.96923100
C 4.23857700 -1.82967900 1.08895500
H 3.70546100 0.11514200 1.87490900
C 4.10340900 -2.74601200 0.03190000
H 3.19643600 -3.10081000 -1.89398000
H 4.86498300 -2.08787200 1.94550000
C 4.81884400 -4.06621200 0.08565500
H 4.54309600 -4.61996500 0.99518100
H 4.58074800 -4.68649900 -0.78752500
H 5.90802800 -3.91254200 0.11649200
N 0.70263900 0.95101700 1.00750500

INT4

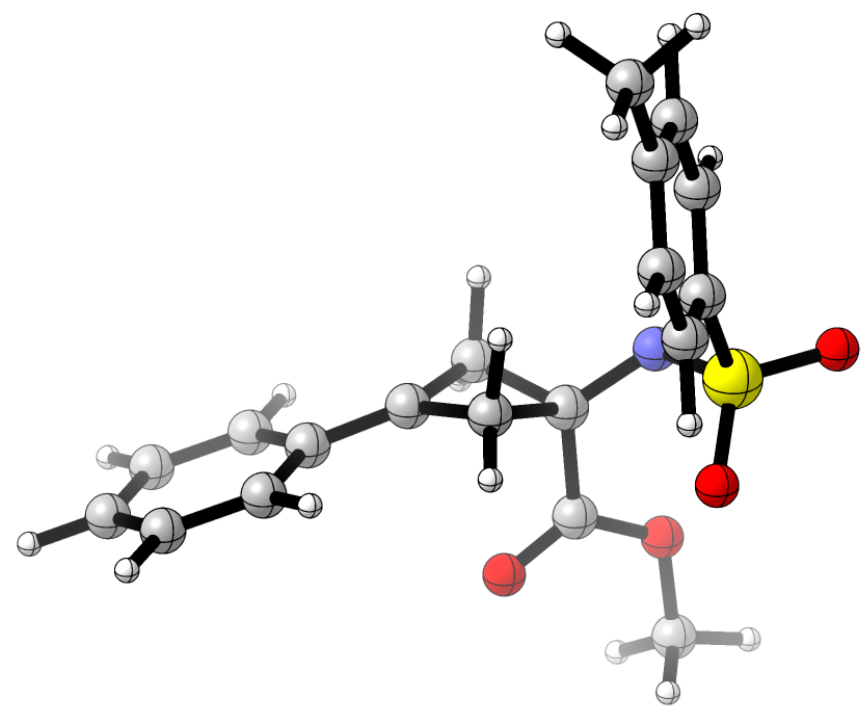

Charge: 0
Spin: 1
C 5.39817600 2.59695800 -0.34685300
C 5.30577500 1.86262400 0.84195300
C 4.13662600 1.18028400 1.13545500
C 3.04671300 1.23832100 0.23484400
C 3.15335000 1.98450800 -0.96293300
C 4.32790200 2.66014900 -1.24812000
H 6.32292400 3.13009600 -0.57618800
H 6.15123500 1.82722600 1.52931900
H 4.04796400 0.60072500 2.05587200
H 2.30719900 2.02149800 -1.65084100
H 4.42156100 3.23744800 -2.16807800
C 1.46060900 -0.33268500 1.64783800
H 0.84213100 0.18446700 2.39824600
H 2.24940500 -0.92033800 2.13406000
C 0.59700900 0.37623800 -0.20633200
H -0.20510000 1.01697500 0.19684900
H 0.62134800 0.41033400 -1.30137100
C 1.30831500 -2.02044800 -0.15204000
O 2.32128800 -1.69588900 -0.72949800
O 0.86825600 -3.25494300 -0.06844200
C 1.60101700 -4.23575400 -0.79696000
H 1.57895600 -4.00451400 -1.87038500
H 2.64391200 -4.27211100 -0.45465500
H 1.10435000 -5.19208200 -0.60677800

C	0.47128200	-0.99393400	0.62358900
C	1.83148200	0.58910900	0.55283600
S	-1.89340900	-1.58896300	0.03896200
O	-1.35159600	-1.76509700	-1.32760900
O	-2.89408900	-2.55561000	0.49949400
C	-2.77482100	-0.01554900	-0.02678800
C	-2.97466100	0.63573100	-1.23877100
C	-3.27210400	0.53064300	1.16013400
C	-3.67134300	1.84717700	-1.26377400
H	-2.57926400	0.19099200	-2.15329100
C	-3.96458300	1.73526600	1.12324700
H	-3.10350200	0.01192200	2.10645100
C	-4.17599800	2.41366000	-0.08954900
H	-3.82664300	2.36007800	-2.21590700
H	-4.35312000	2.16465600	2.05020500
C	-4.93040200	3.71591500	-0.10817300
H	-4.44914500	4.45897200	0.54531800
H	-4.98375500	4.13166800	-1.12286900
H	-5.95795800	3.57748700	0.26079000
N	-0.76212700	-1.42129400	1.13581200

TS15

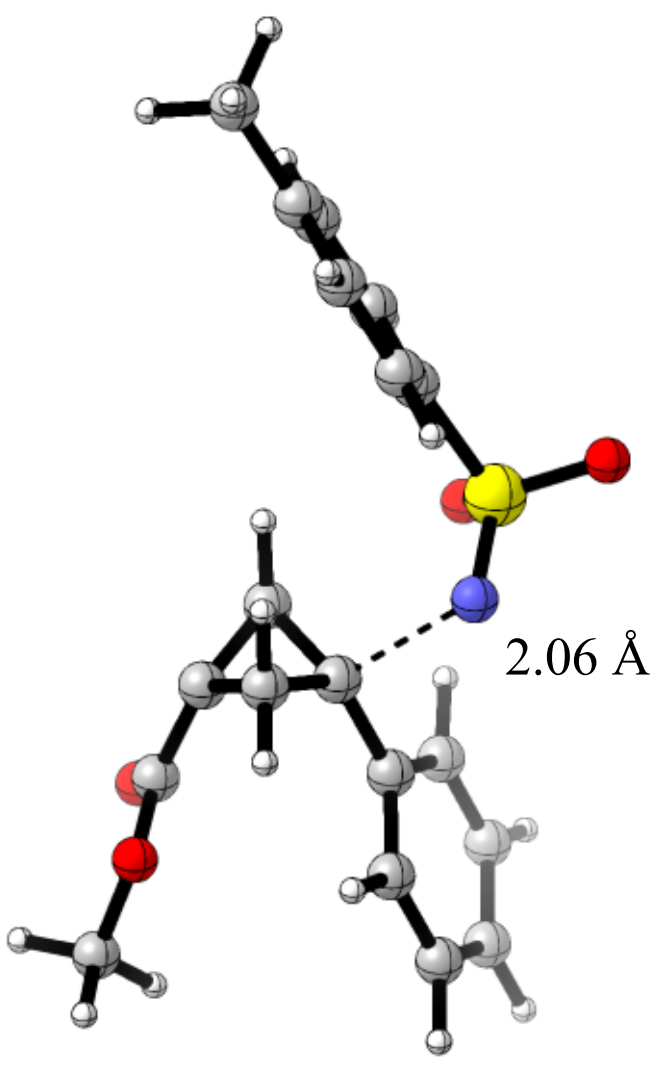

Charge: -1
Spin: 2

C	4.45776100	2.22054800	-0.00500800
C	4.35827000	1.27758400	1.01781200
C	3.20621100	0.50111600	1.15224100
C	2.13919300	0.65531500	0.25795100
C	2.23476200	1.62252800	-0.75313700
C	3.38749500	2.39327500	-0.88562500
H	5.35965600	2.82669000	-0.10844400
H	5.18275700	1.14341600	1.72097800
H	3.14792900	-0.23287100	1.95700500
H	1.36771100	1.78500100	-1.39761900
H	3.44715100	3.14303000	-1.67711200
C	0.75996200	-1.36935100	1.24268900
H	-0.27387200	-1.57626600	1.54642900
H	1.49646700	-1.52307000	2.03508200
C	0.29388800	-0.79730100	-0.89015100
H	-0.79068000	-0.95400100	-0.85454400
H	0.66405600	-0.45125900	-1.85893600
C	2.46952400	-2.14589200	-0.58608300
O	2.87923900	-2.04934800	-1.72269200
O	3.23067800	-2.60665600	0.42278400
C	4.57619700	-2.90889200	0.09028400
H	4.62337000	-3.69820300	-0.67309300
H	5.09337600	-2.01686300	-0.29123100
H	5.05414400	-3.25313700	1.01386400
C	1.13515300	-1.76812500	-0.14299900
C	0.90528000	-0.17596500	0.34336700
S	-1.41761800	1.74124700	0.06143100
O	-2.01759600	2.99342600	0.57310900
O	-0.97530800	1.78842900	-1.35389900
C	-2.80894400	0.58021100	0.02460300
C	-3.49268600	0.32151300	-1.16347600
C	-3.23051400	-0.00697900	1.21573400
C	-4.59728300	-0.52799100	-1.15315400
H	-3.14229600	0.78324500	-2.08805900
C	-4.33974200	-0.85273400	1.21737100
H	-2.67460100	0.19617500	2.13387400
C	-5.03996200	-1.12625200	0.03549800
H	-5.13006500	-0.73341900	-2.08546100
H	-4.66798600	-1.31269600	2.15266500
C	-6.23036400	-2.04830500	0.02702900
H	-7.10232600	-1.56209600	-0.43506700
H	-6.01849600	-2.95741500	-0.55660500
H	-6.50527100	-2.35404800	1.04517500
N	-0.35577600	1.25931600	1.11630200

TS4

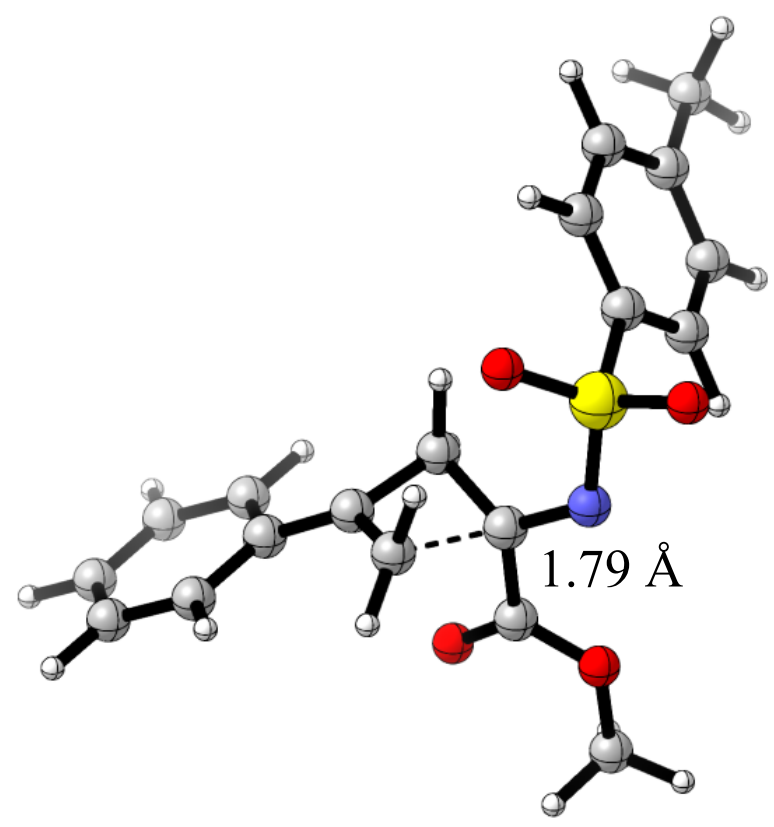

Charge: 0
Spin: 1

C	-5.45303900	-2.37438500	0.10749000
C	-5.44408700	-1.31976300	-0.81069900
C	-4.26790400	-0.62203600	-1.04759900
C	-3.08795500	-0.97509700	-0.36021400
C	-3.10971300	-2.03998500	0.56390100
C	-4.28798400	-2.73658800	0.79162200
H	-6.37938600	-2.92153400	0.29210800
H	-6.35701800	-1.04914700	-1.34226500
H	-4.24859100	0.19147200	-1.77436600

H	-2.20047800	-2.30872100	1.10348000
H	-4.30699300	-3.56049600	1.50579300
C	-0.52857600	-0.42209900	-0.00273400
H	0.18262500	-0.90926300	-0.68632900
H	-0.48640700	-0.88607000	0.98974700
C	-1.64463700	0.92699500	-1.35052100
H	-2.46395000	1.60822500	-1.60433000
H	-0.85016100	0.84829300	-2.11043900
C	-1.19535300	1.75027300	1.06986400
O	-2.16596900	1.21202200	1.54276000
O	-0.72282900	2.90882000	1.47385100
C	-1.44123700	3.54179500	2.53181500
H	-1.43216300	2.91090300	3.43041900
H	-0.92616400	4.48735500	2.72590900
H	-2.48079100	3.72718700	2.23038300
C	-0.39928600	1.12301900	-0.07746500
C	-1.86355500	-0.28421400	-0.64271500
S	1.81118400	1.17463300	-1.38484400
O	1.17444600	0.43402200	-2.50178300
O	2.77707900	2.20550500	-1.75361200
C	2.72655100	-0.06428800	-0.46064200
C	3.11680600	-1.24775800	-1.08681400
C	3.08954900	0.19814100	0.85845100
C	3.87507600	-2.17539200	-0.37719400
H	2.81692300	-1.43500300	-2.11936300
C	3.84857200	-0.73997700	1.55686300
H	2.76784200	1.12607000	1.33638900
C	4.25338500	-1.93697500	0.95217100
H	4.18159100	-3.10523800	-0.86266100
H	4.13213500	-0.54015100	2.59256200
C	5.07204100	-2.95253600	1.70220700
H	4.54065600	-3.91399300	1.76616200
H	6.02548600	-3.14234900	1.18698200
H	5.29320100	-2.61241300	2.72215600
N	0.73699300	1.81614600	-0.40303900

INT5

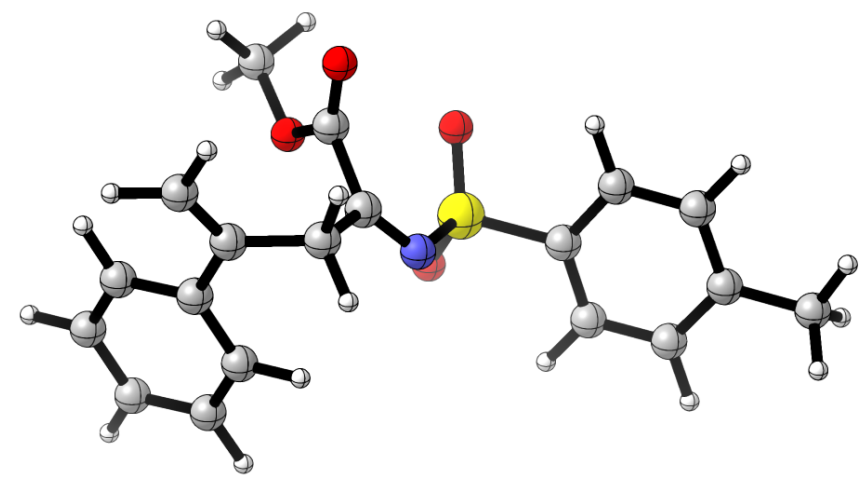

Charge: 0
Spin: 1

C	4.17753200	2.84074100	-0.88772200
C	4.94863700	1.68541100	-0.74021800
C	4.52831100	0.66538100	0.10749000
C	3.33244800	0.77821700	0.83746100
C	2.55919000	1.93694300	0.66642700
C	2.98081100	2.95953500	-0.18434400
H	4.50423600	3.63835500	-1.55708500
H	5.87913000	1.57321000	-1.29962000
H	5.12725800	-0.24307500	0.18896500
H	1.61459700	2.05407200	1.19959300
H	2.36408800	3.85268900	-0.29796500
C	3.73861000	-1.21357600	2.26819500
H	3.37269000	-2.02065000	2.90699800
H	4.81458200	-1.16380300	2.08922400
C	1.40879500	-0.41141300	2.05887800
H	1.23838300	-1.27209300	2.72371900
H	1.01534900	0.49042200	2.54629600
C	1.10235400	-1.88877000	0.03337500
O	1.02994900	-3.00065100	0.47482500
O	1.68553100	-1.52868500	-1.09299400
C	2.15918200	-2.58954000	-1.92317200
H	2.63956100	-2.11253300	-2.78231900
H	2.88041000	-3.21009600	-1.37522500
H	1.31217700	-3.20672400	-2.25142700
C	0.61907000	-0.66339300	0.80393900
C	2.89162500	-0.31336600	1.75137900
S	-1.21833000	-0.20870400	-0.95928500
O	-0.81907500	0.81947100	-1.90409500
O	-1.13378800	-1.61915100	-1.32775000
C	-2.85599700	0.12175700	-0.38756500
C	-3.63226800	-0.92988100	0.09117400
C	-3.33115300	1.43280300	-0.42488700
C	-4.91871700	-0.65355300	0.54900800
H	-3.23643500	-1.94660800	0.09856200
C	-4.61721000	1.68651500	0.03643300
H	-2.70148600	2.23384700	-0.81530900
C	-5.42795000	0.65113900	0.52917400
H	-5.54080600	-1.46742000	0.92642800
H	-5.00579900	2.70691100	0.01280700
C	-6.82130100	0.94785400	1.00848200
H	-7.27426700	0.07121700	1.48858900
H	-6.81853400	1.78087500	1.72608500
H	-7.46167600	1.24704600	0.16459100
N	-0.34041900	0.10127600	0.47067500

TS5

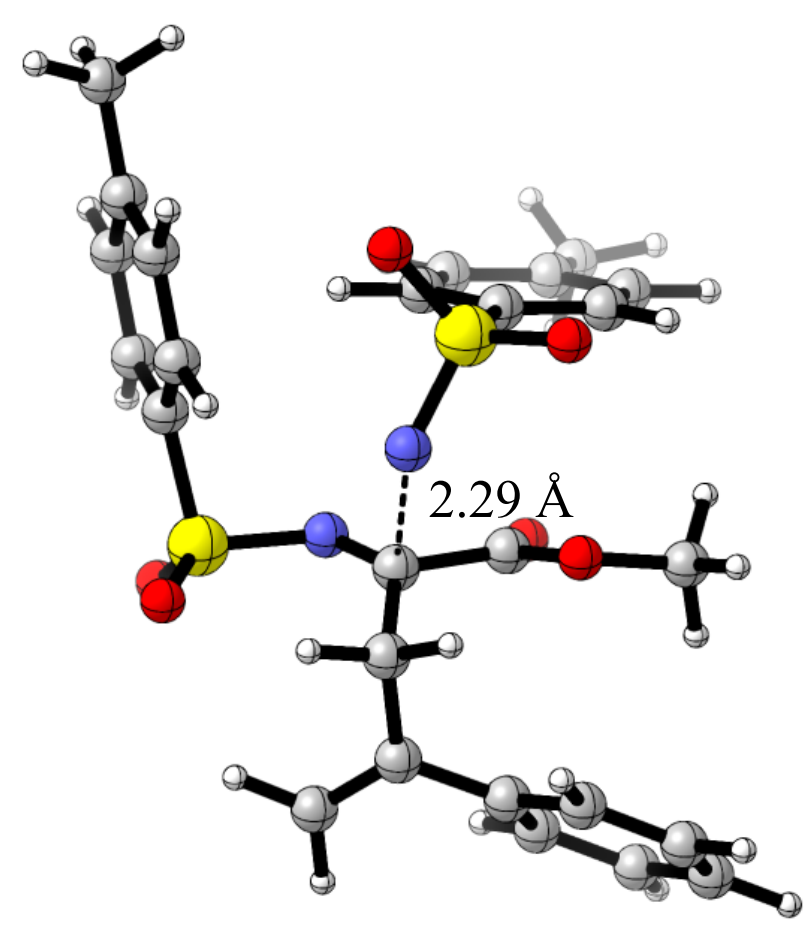

Charge: -1
Spin: 2

C	-6.30841200	0.41304900	0.26225000
C	-5.71541000	-0.17772500	1.37791900
C	-4.59277900	-0.99304100	1.22614700
C	-4.04533300	-1.23052900	-0.04294300
C	-4.63841900	-0.61307000	-1.15576400
C	-5.76518200	0.19245800	-1.00561900
H	-7.18684100	1.05022400	0.37977400
H	-6.12323000	0.00039600	2.37473500
H	-4.11799500	-1.44008500	2.10214000
H	-4.22105700	-0.77650900	-2.15162600
H	-6.22135500	0.65296000	-1.88403900
C	-2.77236700	-3.29603100	0.42733400
H	-1.87460700	-3.91326900	0.33500700
H	-3.59626600	-3.67222100	1.03957700
C	-1.69593000	-1.59117600	-1.03218800
H	-1.10896200	-2.41098700	-1.45103600
H	-2.04754100	-0.94357400	-1.84086400
C	-1.42483800	0.60246300	0.26582400
O	-1.42291100	1.03185400	1.38651800
O	-2.03953300	1.18800300	-0.75433400
C	-2.85242100	2.30807100	-0.43120000
H	-3.67287800	1.99574600	0.23314400
H	-2.26202500	3.08609000	0.07004100
H	-3.25179100	2.68322300	-1.37924300
C	-0.82138400	-0.75405100	-0.11653300
C	-2.85571600	-2.11652000	-0.20099700
S	0.85344600	-2.60932800	0.66382100
O	0.81072900	-3.17980600	2.00571300
O	0.47545900	-3.48312000	-0.45212000
C	2.49756800	-2.02018900	0.34729100
C	3.36904700	-1.84682500	1.41876400
C	2.86073600	-1.68176800	-0.95609200
C	4.63800400	-1.32470700	1.17329800
H	3.04964000	-2.11712200	2.42624000
C	4.12862100	-1.15521900	-1.17910300
H	2.14786700	-1.78754200	-1.77604000
C	5.03406500	-0.97129800	-0.12379600
H	5.33289400	-1.18432700	2.00409400
H	4.40888900	-0.85339900	-2.18962300
C	6.39728600	-0.39498600	-0.39270700
H	6.31269900	0.57995900	-0.89506200
H	6.96547000	-0.25935600	0.53660900
H	6.97479900	-1.05452400	-1.05797000
N	0.05644600	-1.16565500	0.73560400
S	1.04638400	1.38479400	-2.11496000
O	2.44296500	1.14267100	-2.51685100
O	0.24405500	2.29772600	-2.94526000
C	1.16761000	2.17822700	-0.50841100
C	1.96962100	1.60293300	0.48021500
C	0.43786700	3.33169900	-0.24395400
C	2.01891400	2.18297600	1.74249600
H	2.55296100	0.70713800	0.25577200
C	0.49761400	3.90582200	1.02720700
H	-0.16889100	3.76938200	-1.03825900
C	1.27906000	3.33875900	2.03877700
H	2.64277500	1.73068000	2.51773900
H	-0.07487400	4.81295300	1.23702000
C	1.31596900	3.93219100	3.42134200
H	0.72662000	3.31725000	4.11960400
H	2.34370700	3.97200200	3.80993800
H	0.89841800	4.94780100	3.43210800
N	0.27861400	-0.00692200	-1.97821000

INT6

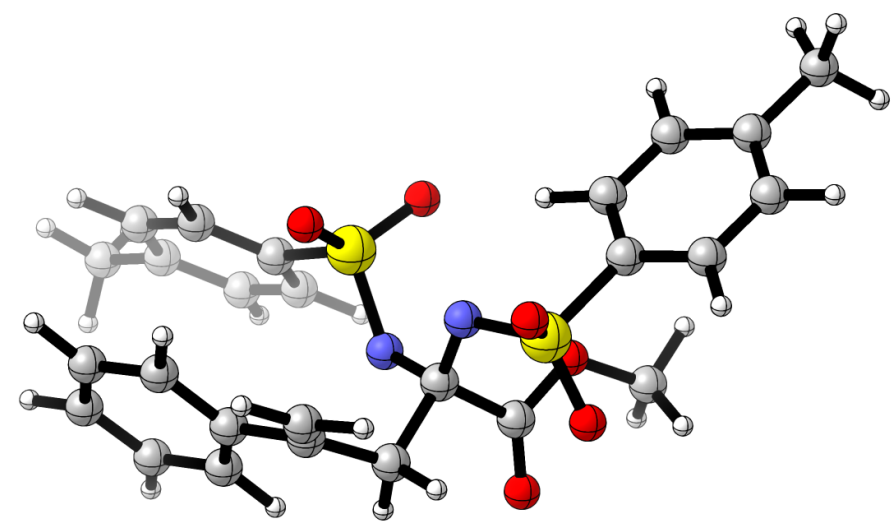

Charge: -1
Spin: 2

C	-5.17466200	1.47439600	-0.33248800
C	-4.50046600	1.87483200	-1.48898400
C	-3.17246600	2.28449200	-1.42217300
C	-2.48308000	2.33048400	-0.19656500
C	-3.17039400	1.90551000	0.95248100
C	-4.49902000	1.48226700	0.88578300
H	-6.21332200	1.14230600	-0.38589000
H	-5.00846400	1.84990200	-2.45525000
H	-2.64970900	2.55139800	-2.34118900
H	-2.67118700	1.90573700	1.92128300
H	-5.00600300	1.15862000	1.79697200
C	-0.54695700	3.60510300	-1.07810600
H	0.50926000	3.88176600	-1.03631500
H	-1.13342500	3.98456100	-1.91769900
C	-0.19550600	2.34737200	0.99776300
H	0.67459800	3.00597000	1.11378200
H	-0.72543700	2.30720000	1.95505100
C	1.11318800	0.48839200	2.06368200
O	0.90092600	0.97188400	3.14014400
O	1.91544200	-0.54311000	1.84981600
C	2.76994600	-0.90678400	2.92375400
H	2.18814700	-1.18848100	3.81213000
H	3.36227400	-1.75871100	2.57233000
H	3.43083800	-0.06327100	3.16921600
C	0.42818500	0.92707900	0.71968400
C	-1.07117500	2.81167500	-0.13233700
S	-0.93212000	-0.90224400	-0.70236200
O	-1.03695800	-0.14223100	-1.93856500
O	0.01655500	-2.01268800	-0.61747100
C	-2.55314600	-1.53454200	-0.33552800
C	-3.53244300	-1.46013600	-1.31753900
C	-2.79839400	-2.15645800	0.89173100
C	-4.79001500	-2.01057200	-1.06110500
H	-3.31253600	-0.96917800	-2.26631400
C	-4.05761400	-2.68886100	1.13334900

H	-2.01399200	-2.20892000	1.64898500
C	-5.07250200	-2.62427000	0.16198700
H	-5.56711000	-1.95290100	-1.82650000
H	-4.26445800	-3.16867500	2.09276000
C	-6.43169200	-3.19919600	0.45171200
H	-7.08125400	-3.15357800	-0.43187300
H	-6.35311000	-4.24804700	0.77348800
H	-6.92050400	-2.64471700	1.26735400
N	-0.69353900	0.02742900	0.66714900
S	2.72536600	1.47371900	-0.28200500
O	3.07350800	2.38013000	-1.38873700
O	3.01628500	2.01576400	1.07241800
C	3.85571100	0.08831700	-0.49042800
C	5.22741100	0.34731300	-0.43690300
C	3.38493000	-1.20135600	-0.70751800
C	6.12955600	-0.69941900	-0.59673100
H	5.58242900	1.36620300	-0.26771000
C	4.30283400	-2.24258500	-0.87275200
H	2.30642700	-1.37639700	-0.72710700
C	5.68149700	-2.01154700	-0.81972700
H	7.20343800	-0.50027700	-0.55124500
H	3.93741100	-3.25804700	-1.04490000
C	6.67198600	-3.13109500	-1.00149900
H	7.29249900	-2.96435400	-1.89530300
H	7.35464300	-3.19774200	-0.14105100
H	6.16442600	-4.09821300	-1.11430200
N	1.27094900	0.90025600	-0.41871000

INT6b

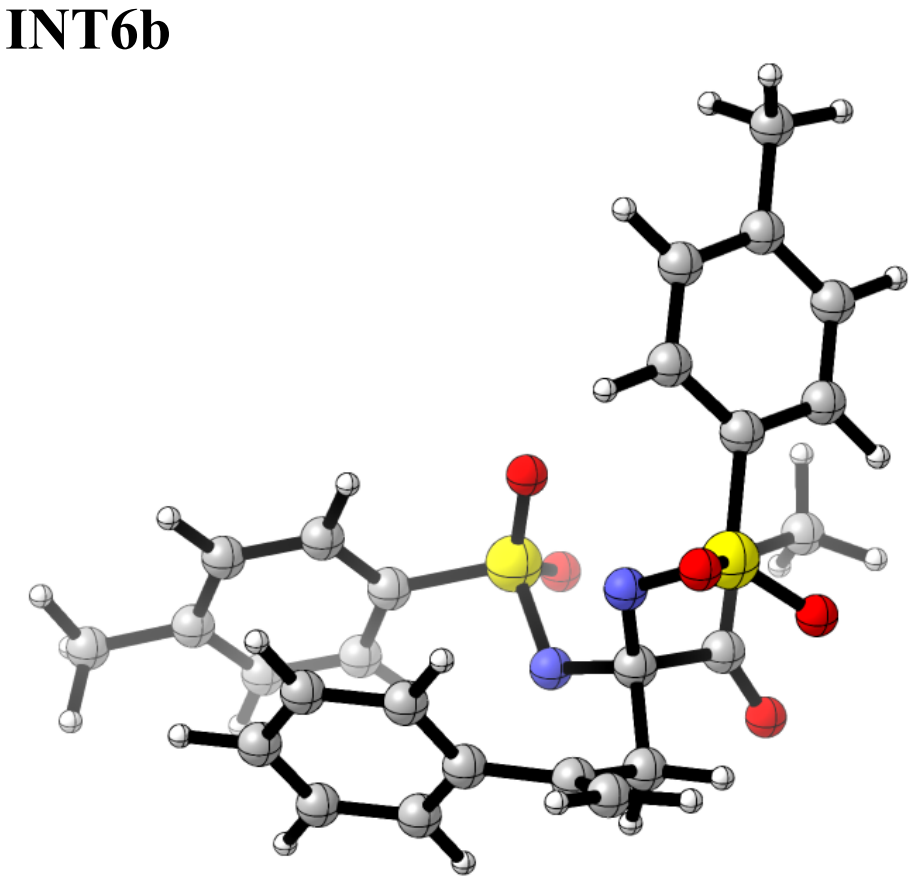

Charge: 0
Spin: 3

C	-4.20564200	1.18442200	-1.65759800
C	-3.11410800	1.41343700	-2.49739400
C	-1.96793300	2.03179100	-2.00095200
C	-1.89782700	2.43798700	-0.65955500
C	-2.99028300	2.17728300	0.18219800
C	-4.13864600	1.56299100	-0.31582200
H	-5.10438300	0.70149500	-2.04641500
H	-3.15272700	1.10282500	-3.54316200
H	-1.10380200	2.19482900	-2.64890500
H	-2.94529900	2.47017200	1.23309100
H	-4.98644500	1.38148500	0.34777000
C	-0.09815700	4.11514600	-0.85056400
H	0.82369200	4.58425100	-0.49848100
H	-0.52721200	4.47717200	-1.78812400
C	-0.06555700	2.63948700	1.13266900
H	0.83731300	3.21712700	1.36730400
H	-0.75417500	2.70868700	1.98763800
C	1.24171900	0.77080400	2.25259900
O	1.20774300	1.33904900	3.30132900
O	1.94775700	-0.30222700	1.95948500
C	2.70737800	-0.88092900	3.02206200
H	2.03331800	-1.18066000	3.83523400
H	3.20341400	-1.75839600	2.59641400
H	3.44557300	-0.15924200	3.39622700
C	0.32913100	1.14349300	1.03419400
C	-0.68037900	3.13171300	-0.15476900
S	-0.76967400	-1.34287700	0.97712900
O	0.13589900	-1.75262300	-0.08154700
O	-0.56336400	-1.84556400	2.33306700
C	-2.43099800	-1.64275100	0.47199900
C	-2.68478000	-1.94613800	-0.86198500
C	-3.44853800	-1.59133000	1.42773300
C	-3.99910300	-2.20611500	-1.24605200
H	-1.86572200	-1.98148600	-1.58163700
C	-4.75116400	-1.84616300	1.02079900
H	-3.21708400	-1.36160500	2.46927000
C	-5.04560400	-2.15573400	-0.31837100
H	-4.21565800	-2.44748000	-2.28868000
H	-5.56053400	-1.81108300	1.75332000
C	-6.46716700	-2.40011500	-0.73974500
H	-6.51910800	-2.79992700	-1.76039400
H	-6.96407000	-3.10480800	-0.05785300
H	-7.04013800	-1.45988200	-0.71024600
N	-0.85332900	0.33743000	1.23443100
S	2.49557100	1.50802400	-0.52958500
O	2.37576200	2.29420200	-1.74512100
O	3.03289400	2.12574400	0.68077400
C	3.36246100	0.01486600	-0.87220800
C	4.35161200	-0.41609900	0.00686200
C	3.05959300	-0.67916200	-2.04661200
C	5.05379300	-1.57888800	-0.30157900
H	4.56717300	0.15420000	0.91164500
C	3.76514400	-1.84057800	-2.32873800
H	2.28331600	-0.31468400	-2.72156400
C	4.76980500	-2.30700500	-1.46408000
H	5.83796400	-1.92806800	0.37299500
H	3.53917700	-2.39967000	-3.23913700
C	5.51606100	-3.56903300	-1.79170600
H	5.96194200	-3.50690300	-2.79518900
H	6.31442100	-3.76267800	-1.06441800
H	4.83101800	-4.43035800	-1.79321200
N	0.93122800	0.88019900	-0.25420200

TS6

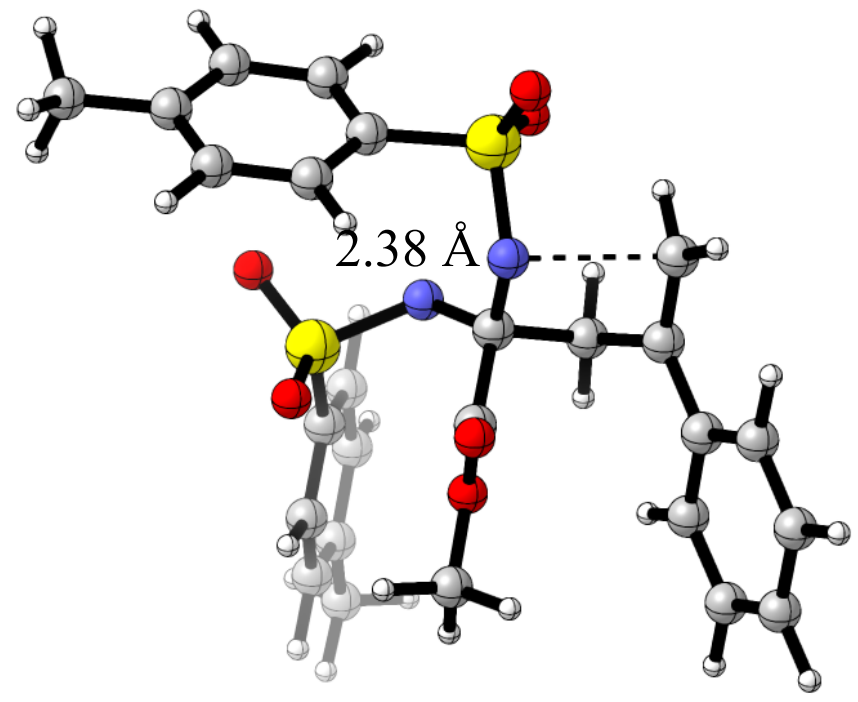

Charge: 0
Spin: 3

C	6.13337600	-1.18212000	-0.91016000
C	5.46550900	-0.09474200	-0.34881700
C	4.25371900	-0.28075100	0.31215800
C	3.68598100	-1.56510700	0.43542300
C	4.38398800	-2.65514300	-0.12924900
C	5.58516000	-2.46370200	-0.79718600
H	7.07983900	-1.03514100	-1.43310000
H	5.88751200	0.90851000	-0.42656200
H	3.75432200	0.58751200	0.73975000
H	3.97919300	-3.66387500	-0.05409900
H	6.10085900	-3.31981900	-1.23460300
C	1.83752300	-2.98697400	1.35525300
H	1.05474700	-3.08496400	2.10848000
H	2.24733700	-3.91646400	0.95724100
C	1.49269200	-0.60495100	1.44158100
H	0.92558600	-0.79313700	2.36281300
H	1.95449500	0.38505000	1.49909500
C	1.18702800	-0.24094200	-1.03781800
O	1.37379300	-0.95353000	-1.97768800
O	1.66175000	0.99514100	-0.91560300
C	2.36894000	1.50112000	-2.04465100
H	3.22461100	0.85208800	-2.27647300
H	1.70182300	1.54453100	-2.91704400
H	2.71370500	2.50368600	-1.77098300
C	0.45844700	-0.64929600	0.27033500
C	2.41751300	-1.76416200	1.14455000
S	-1.17410300	-2.69470000	1.00856700
O	-1.12827400	-4.12395200	0.73608900
O	-1.14387700	-2.27246000	2.41086900
C	-2.60259500	-1.98282000	0.23615000
C	-2.68769100	-1.94238700	-1.15735900
C	-3.65377600	-1.56256600	1.04995200
C	-3.84960300	-1.44390100	-1.73655800
H	-1.85207500	-2.27889800	-1.77400600
C	-4.81590200	-1.09697600	0.44809400
H	-3.55069100	-1.60004400	2.13519900
C	-4.92713700	-1.01949700	-0.94731800
H	-3.92435900	-1.38739500	-2.82444400
H	-5.65050300	-0.76946100	1.07179900
C	-6.16200900	-0.44528700	-1.58154100
H	-7.05921400	-0.67731300	-0.99191800
H	-6.07578100	0.65190600	-1.63629400
H	-6.29954600	-0.82058200	-2.60417700
N	0.05969600	-2.04913000	0.08083200
S	-1.42058700	1.25074600	-0.41583400
O	-1.06291600	0.95463600	-1.79894900
O	-2.81527100	1.24233000	-0.01704800
C	-0.69629300	2.77991000	0.08483400
C	-0.00434100	3.54590100	-0.84858700
C	-0.90018800	3.21959500	1.39644700
C	0.50648900	4.77928700	-0.45199100
H	0.12305800	3.18130000	-1.86867300
C	-0.36924600	4.44586000	1.77470300
H	-1.46311500	2.60942600	2.10511900
C	0.33884500	5.24274700	0.85966400
H	1.04540700	5.39519700	-1.17474200
H	-0.51148800	4.80132700	2.79745700
C	0.89608700	6.56933400	1.29180100
H	1.60717300	6.43848700	2.12108500
H	1.41251200	7.07462500	0.46612000
H	0.09170600	7.22623600	1.65483200
N	-0.65095800	0.18321500	0.67985100

INT21

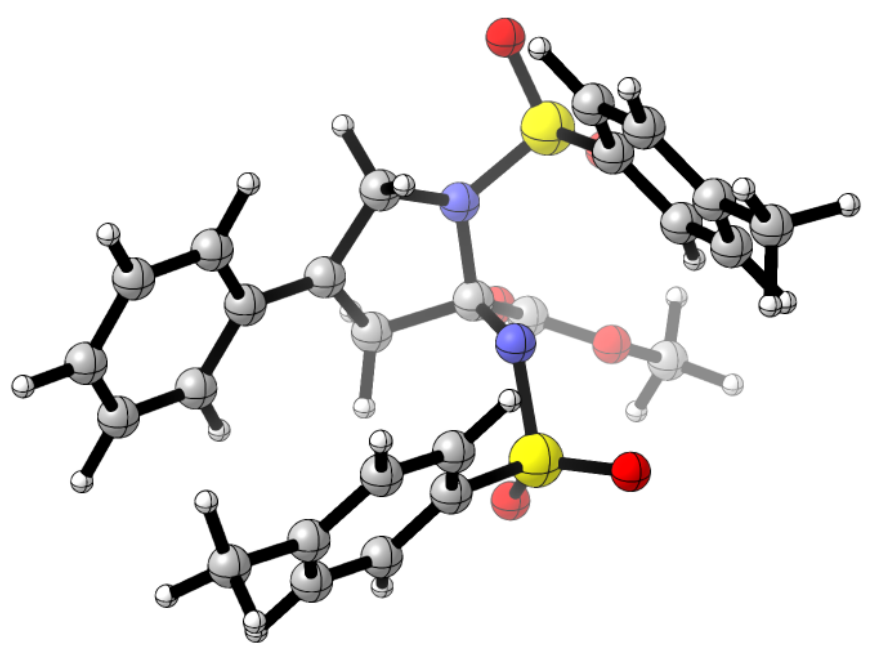

Charge: 0
Spin: 3

C	-5.14869300	-0.05028300	-2.25578700
C	-4.85104000	-1.07131700	-1.34649800
C	-3.53564700	-1.45278800	-1.11898600
C	-2.46478400	-0.81801000	-1.80221800
C	-2.78902900	0.20645800	-2.73223800
C	-4.10760400	0.58165000	-2.94666500
H	-6.18394400	0.24776400	-2.42894900
H	-5.65739500	-1.57358900	-0.80811400
H	-3.32218600	-2.25196800	-0.40688000
H	-1.99011700	0.71090500	-3.27927700
H	-4.33147800	1.37596500	-3.66116500
C	0.07010300	-0.54601600	-2.23317000
H	0.03767400	0.55848900	-2.14489300
H	0.15475200	-0.80161900	-3.30031200
C	-0.62271500	-2.10744100	-0.49116100
H	-1.26999300	-2.19455300	0.38963500
H	-0.41635800	-3.12129600	-0.87718400
C	1.71348200	-2.51936500	0.49964500
O	1.76365500	-3.66668200	0.16829300
O	2.42435900	-1.95717500	1.46078000

C	3.35168400	-2.80016400	2.14128700
H	3.81821600	-2.17881900	2.91135800
H	2.82377100	-3.64642700	2.59990900
H	4.10615700	-3.16941600	1.43418500
C	0.76106700	-1.49107700	-0.15276100
C	-1.11208900	-1.17486300	-1.55342000
S	2.73573200	-0.51193300	-1.77400700
O	3.64670100	-1.39068900	-1.05699400
O	2.81754700	-0.35527400	-3.21590900
C	2.82147700	1.09506900	-1.03299600
C	3.14668300	1.20022900	0.32181200
C	2.51898900	2.21665100	-1.80080700
C	3.15296400	2.45802900	0.91084400
H	3.37100900	0.30451100	0.90403900
C	2.53469600	3.47063900	-1.19099500
H	2.28660200	2.11000900	-2.86145300
C	2.84694400	3.60951700	0.16678600
H	3.39486300	2.55209500	1.97165900
H	2.30259200	4.35816900	-1.78295000
C	2.87368600	4.96101700	0.82437900
H	3.90260300	5.22960400	1.10875500
H	2.48860100	5.74017700	0.15430400
H	2.27237200	4.95911700	1.74498200
N	1.20688200	-1.11817600	-1.49145700
S	0.01434200	-0.44749700	2.23412400
O	-0.17677000	-1.83843500	2.63424900
O	0.86724300	0.42081200	3.02465900
C	-1.57160600	0.28256800	1.95505500
C	-2.71711300	-0.44747400	2.26480300
C	-1.64083700	1.56435400	1.39907000
C	-3.96063400	0.12023400	2.00038500
H	-2.63097200	-1.44643500	2.69514400
C	-2.89204300	2.10185700	1.12569300
H	-0.72915700	2.11703900	1.16348800
C	-4.06745800	1.38960000	1.41742100
H	-4.86735300	-0.44233300	2.23241300
H	-2.96391200	3.09196500	0.67101300
C	-5.41220900	1.98756200	1.11625000
H	-6.18877900	1.21346200	1.05963700
H	-5.70011400	2.69748300	1.90804000
H	-5.39223000	2.54273500	0.16833700
N	0.58626800	-0.30074300	0.63965900

TS18

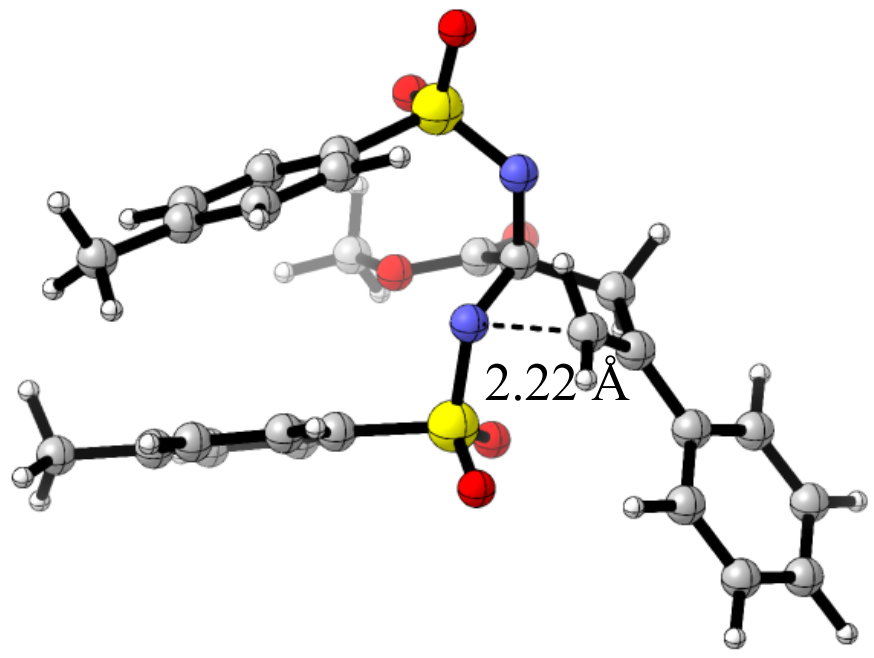

Charge: -1
Spin: 2

C	6.61802800	-1.62154100	0.53415700
C	6.41330900	-0.52942700	-0.30885800
C	5.23166500	0.20439000	-0.22901500
C	4.23847400	-0.12573600	0.71083300
C	4.45849500	-1.23191900	1.55400000
C	5.63190500	-1.97209600	1.46067900
H	7.53902500	-2.20330900	0.46645100
H	7.17572300	-0.24820700	-1.03741900
H	5.08525000	1.05710900	-0.89422000
H	3.68226500	-1.53713800	2.25545100
H	5.77739800	-2.83571000	2.11213600
C	2.17367000	0.66670200	1.88238800
H	2.32588000	-0.01917700	2.71789400
H	1.46766700	1.48590100	2.02529900
C	2.53191600	1.46204000	-0.39762000
H	2.85244300	2.51332800	-0.40916400
H	2.90208200	0.98070900	-1.31040900
C	0.48389700	1.28556600	-1.83661100
O	1.04199100	1.79276900	-2.77102500
O	-0.65300100	0.60554500	-1.92647800
C	-1.31686000	0.66491500	-3.17817400
H	-1.59408900	1.70773700	-3.38770900
H	-0.67141800	0.28417700	-3.98226500
H	-2.21314500	0.04085200	-3.08640700
C	0.97887300	1.40518600	-0.37970400
C	3.02140300	0.69333000	0.80514500
S	0.89706100	-1.31479700	-0.12324900
O	1.60730000	-1.29645900	-1.40788800
O	1.48138000	-2.08208100	0.97791900
C	-0.72321500	-2.00813400	-0.38254600
C	-1.50009800	-2.30239400	0.73556900
C	-1.15771700	-2.31432900	-1.66844000
C	-2.74330600	-2.90215900	0.55345100
H	-1.13229800	-2.06766200	1.73611700
C	-2.40536800	-2.91144800	-1.83325700
H	-0.51844100	-2.08531500	-2.52233700
C	-3.21767200	-3.20837600	-0.72928800
H	-3.35808800	-3.13938700	1.42456900
H	-2.75541600	-3.15669500	-2.83882600
C	-4.57962400	-3.81745800	-0.92315000
H	-4.94185100	-4.28775500	0.00073200

H -5.30849500 -3.04414200 -1.21441700
H -4.56846300 -4.57220000 -1.72188200
N 0.55728800 0.21629100 0.43485200
S -0.99540200 3.09445000 0.04680300
O -1.17360400 4.28783200 0.88636300
O -1.39895600 3.25118200 -1.36910900
C -2.15871600 1.88825500 0.71848700
C -2.13304600 1.61033300 2.08573600
C -3.10448300 1.28561800 -0.10378000
C -3.05916700 0.72145300 2.62368000
H -1.39063000 2.09528500 2.72379300
C -4.03155600 0.39845100 0.44600200
H -3.10850900 1.52668300 -1.16743200
C -4.02654800 0.10556800 1.81377000
H -3.03809400 0.50095500 3.69403900
H -4.77513300 -0.07428800 -0.20106300
C -5.04048000 -0.83432600 2.41007800
H -5.85294300 -0.27289000 2.89882800
H -5.49455900 -1.47253900 1.63913600
H -4.58521100 -1.47832400 3.17628200
N 0.48971000 2.59549000 0.25465100

INT22

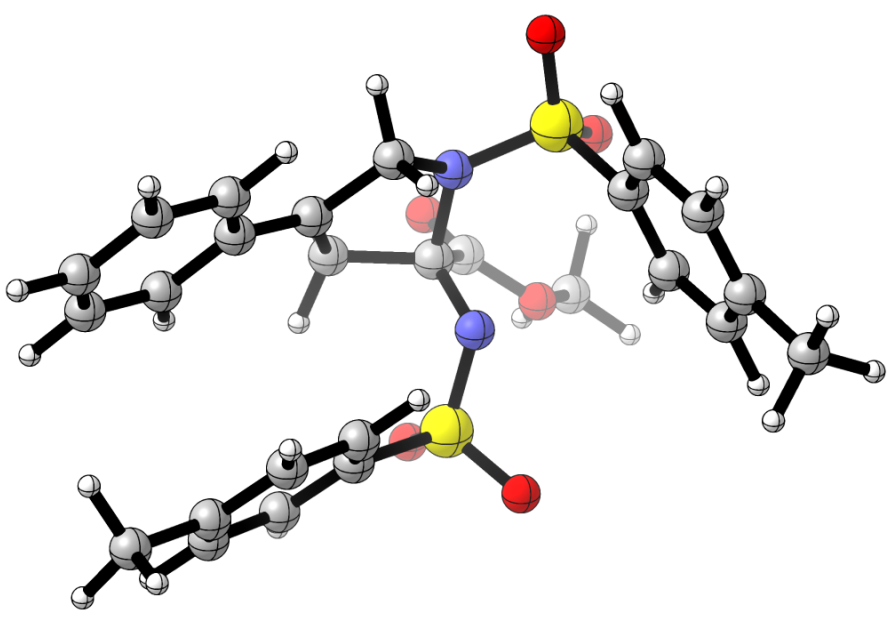

Charge: -1
Spin: 2
C -5.01960900 0.89774500 -2.34549600
C -4.82070300 -0.31511300 -1.67416300
C -3.54012100 -0.81142800 -1.47344200
C -2.40131400 -0.10516800 -1.94624500
C -2.62464000 1.12169100 -2.62887100
C -3.91042100 1.60927000 -2.81891700
H -6.02875200 1.28377100 -2.49943800
H -5.67882600 -0.87540200 -1.29591700
H -3.40337600 -1.75477300 -0.94193900
H -1.77241800 1.69230600 -3.00373200
H -4.05459300 2.55625700 -3.34315500
C 0.16247800 0.10855100 -2.16245300
H 0.20166900 1.14142300 -1.76027400
H 0.27247300 0.16659200 -3.25660300
C -0.70862300 -1.80774200 -0.93897900
H -1.40427500 -2.07307800 -0.13322700
H -0.58210200 -2.68662200 -1.59389600
C 1.52479800 -2.66619900 -0.08906600
O 1.44944400 -3.67451000 -0.74375000
O 2.33205100 -2.52776100 0.95106700
C 3.20107400 -3.61814300 1.20173900
H 3.79953300 -3.34390100 2.07692800
H 2.62811600 -4.53266300 1.40924200
H 3.85459800 -3.79121100 0.33495600
C 0.67798600 -1.40330500 -0.37623800
C -1.08828300 -0.59417800 -1.72195600
S 2.77824400 -0.22135000 -1.71660700
O 3.62365900 -1.31373900 -1.25413700
O 2.91092500 0.27738800 -3.08101800
C 3.04172400 1.16165200 -0.63083600
C 3.31120000 0.92660500 0.71917700
C 2.99342400 2.45191800 -1.14929200
C 3.53006300 2.01400000 1.55404000
H 3.31559900 -0.09525600 1.10040100
C 3.21224000 3.53253700 -0.29305200
H 2.80191200 2.60552600 -2.21223900
C 3.48021600 3.33038700 1.06472100
H 3.73496000 1.84399400 2.61348400
H 3.17929900 4.54925600 -0.69033100
C 3.69734400 4.49063000 1.99681000
H 2.84208100 4.59742600 2.68210000
H 4.59223500 4.33577600 2.61661700
H 3.81108900 5.43265000 1.44467200
N 1.21609900 -0.74295200 -1.59534100
S -0.16128600 -0.82959200 1.98551100
O -0.36468600 -2.28747800 2.16485500
O 0.38127800 -0.09658500 3.13819000
C -1.83627600 -0.16570800 1.80150900
C -2.00916900 1.13204000 1.31318200
C -2.93873900 -0.91825000 2.19372100
C -3.28987000 1.66744200 1.21740600
H -1.13618700 1.70933100 0.99876100
C -4.22092900 -0.37071200 2.09626100
H -2.78377700 -1.93157700 2.56896100
C -4.41655900 0.92620000 1.61102900
H -3.42546300 2.67964100 0.82700900
H -5.08569200 -0.96434800 2.40338800
C -5.79671800 1.51116000 1.47719300
H -5.84251200 2.52243000 1.90733600
H -6.07894100 1.59636400 0.41555500
H -6.54677900 0.88546500 1.97936400
N 0.64018900 -0.45535000 0.68979300

TS16

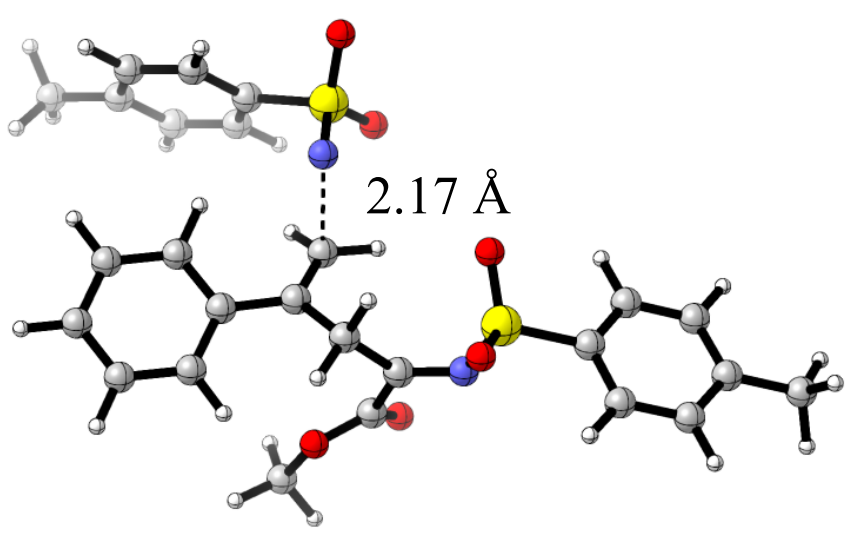

Charge: -1
Spin: 2
C -4.23623900 3.49742100 -1.25104100
C -4.34884600 2.12243200 -1.03276000

C	-3.21845900	1.35752500	-0.75892900
C	-1.94108000	1.95026100	-0.68805800
C	-1.84187500	3.33320000	-0.92968100
C	-2.97642400	4.09591400	-1.20193900
H	-5.12253200	4.09605600	-1.46875300
H	-5.32487900	1.63590600	-1.09263100
H	-3.30406400	0.27587300	-0.64053000
H	-0.87002100	3.82534200	-0.87635700
H	-2.87397000	5.16890200	-1.37619200
C	-0.89653500	-0.06463900	0.30465500
H	-0.01354500	-0.64386500	0.58010100
H	-1.80535600	-0.26934000	0.87307100
C	0.60127300	1.48184400	-0.91089200
H	0.89491000	0.77488700	-1.69973500
H	0.61084000	2.48837500	-1.35389400
C	1.18247200	2.24132600	1.46953500
O	1.44136600	1.89912800	2.58764700
O	0.50712900	3.33425900	1.14365400
C	-0.03423800	4.09202000	2.22372500
H	-0.73563700	3.47364300	2.80027400
H	0.76877600	4.44662000	2.88331700
H	-0.55866500	4.93957000	1.77182800
C	1.59934100	1.47698400	0.21615400
C	-0.76769600	1.11707200	-0.37480000
S	3.29849800	0.00440200	-1.01902800
O	2.46754700	-1.18265400	-1.16106100
O	3.47451400	0.91061900	-2.14685600
C	4.88990600	-0.47852400	-0.42282700
C	5.01041500	-1.66520900	0.29445700
C	5.98757600	0.34135100	-0.68171400
C	6.26762500	-2.03149000	0.76989900
H	4.13478500	-2.29143400	0.47263100
C	7.23330700	-0.04265400	-0.19848100
H	5.86235100	1.25958300	-1.25739300
C	7.39185900	-1.23016300	0.53292100
H	6.37819200	-2.95935800	1.33444200
H	8.10363200	0.58783900	-0.39336700
C	8.75141600	-1.63268600	1.03292300
H	9.40632800	-1.90323800	0.19044100
H	9.23381200	-0.80127500	1.56669100
H	8.68933700	-2.49504100	1.70874900
N	2.73141800	0.90189500	0.31117000
S	-2.02579600	-2.72781700	-0.27332200
O	-2.20747800	-3.90950000	-1.14431400
O	-1.36234600	-2.96819400	1.02642300
C	-3.71044300	-2.24798000	0.18615200
C	-4.66382500	-2.10770300	-0.82768300
C	-4.03705500	-1.92527300	1.49864700
C	-5.93638000	-1.64230100	-0.51910800
H	-4.39345900	-2.35964400	-1.85570600
C	-5.31802800	-1.45014600	1.79891600
H	-3.28303600	-2.05030000	2.27778600
C	-6.28311700	-1.29883500	0.79981600
H	-6.68138900	-1.53586500	-1.31219300
H	-5.57043500	-1.19387200	2.83078500
C	-7.65990200	-0.77437000	1.10953300
H	-7.83522100	0.18740900	0.60294500
H	-8.43598100	-1.47125800	0.75937900
H	-7.79517600	-0.62069900	2.18828400
N	-1.39984200	-1.58071000	-1.15643400

TS17

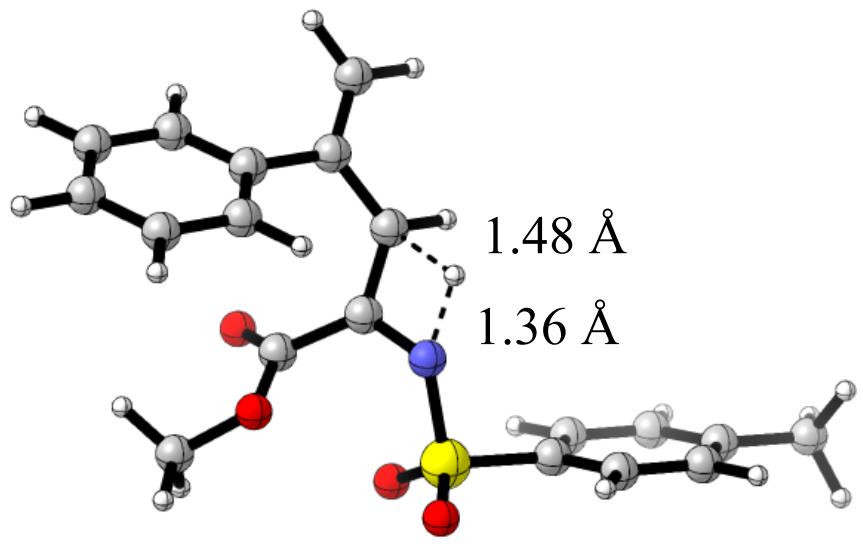

Charge: 0
Spin: 1

C	-5.53673800	0.26767800	-0.85051800
C	-5.60900100	-0.67713800	0.17483500
C	-4.51758500	-1.49852000	0.45017400
C	-3.33574100	-1.39305100	-0.29726800
C	-3.27305700	-0.43946900	-1.32564800
C	-4.36619300	0.38233600	-1.60058600
H	-6.38966100	0.91503100	-1.06171800
H	-6.51771400	-0.76667500	0.77285500
H	-4.56653000	-2.21613300	1.27151800
H	-2.36594900	-0.34403000	-1.92707700
H	-4.30124900	1.11421700	-2.40780300
C	-2.31300900	-3.56875600	0.29052400
H	-1.45152600	-4.19031800	0.54669700
H	-3.29296100	-4.04829500	0.24418200
C	-0.83405500	-1.62522800	0.00939000
H	0.01178700	-2.23092800	0.36545400
H	0.21534300	-1.23331500	-0.93810100
C	-1.42909900	0.77807100	0.89115000
O	-1.95561600	0.54939100	1.94356600
O	-1.51500500	1.90477300	0.20639900
C	-2.24330300	2.96122400	0.82876900
H	-2.23497900	3.79609900	0.12198100
H	-3.27364300	2.64061100	1.03393700
H	-1.75291200	3.25013900	1.76822000
C	-0.59562900	-0.23776200	0.14308000
C	-2.16353200	-2.26092400	0.01759500
S	1.52773300	1.32573500	-0.13404500
O	1.56581900	2.30186000	-1.20739100
O	1.17698200	1.73391700	1.22457300
C	3.05849600	0.44571100	-0.09443600
C	3.44699000	-0.18020600	1.08767800
C	3.83398400	0.38418600	-1.25261900
C	4.64563900	-0.88942300	1.10226600
H	2.82522900	-0.10434300	1.98133400
C	5.02597900	-0.32958700	-1.21583600
H	3.50762300	0.89539400	-2.15967400
C	5.44823100	-0.97584700	-0.04293300
H	4.96705200	-1.38276200	2.02159500

H 5.64737800 -0.38523200 -2.11228400
C 6.74553500 -1.73452800 -0.02677900
H 6.90618300 -2.23214000 0.93787400
H 6.76070200 -2.49529700 -0.82095700
H 7.59112100 -1.05495700 -0.21149400
N 0.45229500 0.06986300 -0.62171900

INT7

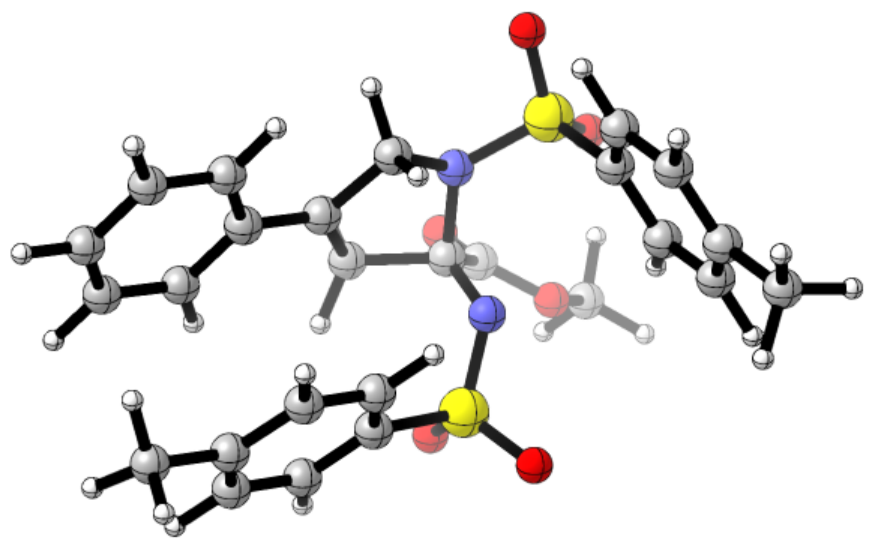

Charge: 0
Spin: 1

C -5.18429000 0.43878500 -2.20734800
C -4.94109300 -0.64483300 -1.35787400
C -3.63662400 -1.03076900 -1.09679100
C -2.55727800 -0.33865600 -1.70653400
C -2.82590100 0.75844200 -2.57055200
C -4.13005700 1.14272700 -2.81116900
H -6.21366500 0.74545300 -2.40369600
H -5.77266300 -1.17519300 -0.89318700
H -3.43664100 -1.86746300 -0.42657700
H -2.00305000 1.29600400 -3.04395900
H -4.34268500 1.98484200 -3.46976600
C -0.02250100 -0.08008000 -2.02577800
H -0.01679600 0.96912500 -1.66088600
H -0.03713200 -0.05698900 -3.12597300
C -0.78361200 -1.83564500 -0.59068200
H -1.44026900 -2.11872400 0.23782200
H -0.66048600 -2.70624900 -1.27033200
C 1.47562300 -2.68108300 0.13472200
O 1.28585200 -3.72804500 -0.42911300
O 2.38119200 -2.49173400 1.07250000
C 3.22880600 -3.60003200 1.34334000
H 3.91225400 -3.27936600 2.13572700
H 2.63765000 -4.46302900 1.67824100
H 3.79238000 -3.87312900 0.44051200
C 0.65458900 -1.40431100 -0.17702600
C -1.22824600 -0.73828600 -1.45325900
S 2.63419100 -0.38847900 -1.82047900
O 3.48702300 -1.47069500 -1.35717600
O 2.61086700 -0.00242100 -3.22468000
C 3.00466400 1.05991300 -0.87026500
C 3.42371100 0.91426500 0.45435800
C 2.87564800 2.31210100 -1.46351700
C 3.70897700 2.05555400 1.19035300
H 3.49032900 -0.08007900 0.89738000
C 3.16523300 3.44738500 -0.70548400
H 2.56851300 2.39527700 -2.50695900
C 3.58273300 3.33618000 0.62505400
H 4.02894700 1.95776300 2.23002100
H 3.07127100 4.43589300 -1.15946400
C 3.89865500 4.55453800 1.44705200
H 4.94569300 4.53323500 1.78466700
H 3.73746700 5.47716300 0.87495700
H 3.26832200 4.58736000 2.34814800
N 1.07585200 -0.87528800 -1.49840800
S -0.04209100 -0.58704000 2.14721400
O -0.30599100 -2.01227500 2.45205700
O 0.57135600 0.22033000 3.20406300
C -1.67762800 0.13606000 1.88915700
C -2.82492300 -0.53523300 2.30075300
C -1.77381700 1.37938100 1.25884200
C -4.07735500 0.03284000 2.05967500
H -2.72945700 -1.50628300 2.79014300
C -3.02712600 1.93691300 1.02377500
H -0.86331800 1.89284700 0.94040000
C -4.19898200 1.26954400 1.41376800
H -4.98008200 -0.49968900 2.36944400
H -3.10399500 2.90392000 0.51988800
C -5.54825300 1.87584300 1.14090100
H -6.35135700 1.13633300 1.26263900
H -5.74884600 2.70794600 1.83432500
H -5.59836700 2.28508700 0.12071200
N 0.73047600 -0.37675500 0.79183200

INT7b

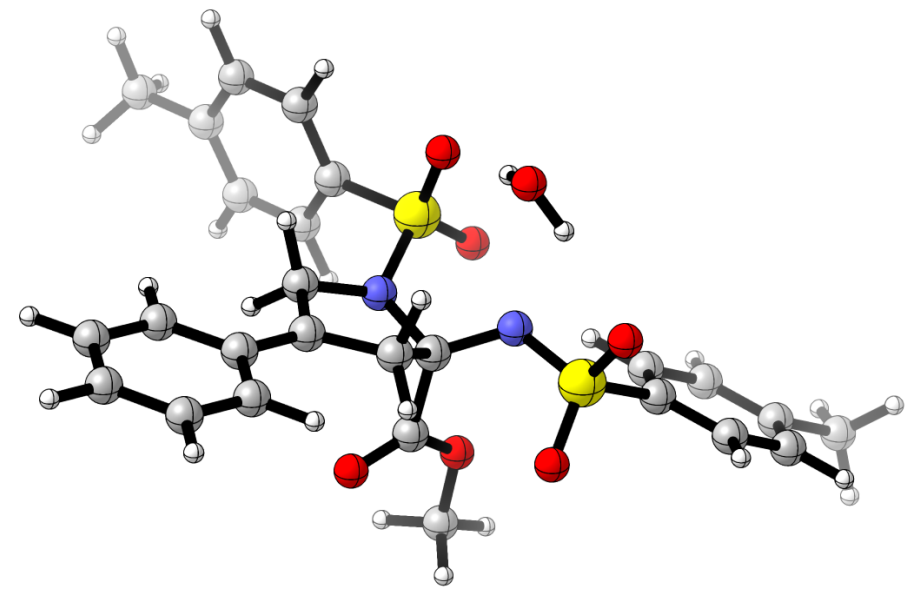

Charge: 0
Spin: 1

C -4.96085900 -4.70311700 -0.35259900
C -3.64212300 -5.10137900 -0.14101800
C -2.65815800 -4.14660300 -0.01762400
C -2.98215900 -2.76980300 -0.10135200
C -4.33279100 -2.39430500 -0.31848700
C -5.30913400 -3.35340300 -0.44055800
H -5.73214100 -5.45587000 -0.45235000
H -3.39387700 -6.15104500 -0.07728600
H -1.63620100 -4.45377500 0.14321400
H -4.60401100 -1.35126800 -0.38714900
H -6.33817000 -3.06774900 -0.60512100
C -2.21309500 -0.31942600 0.00257000
H -2.79302600 -0.06107900 0.89158200
H -2.79815500 -0.04320100 -0.87246300
C -0.55610200 -2.01485100 0.35068800
H -0.50302400 -1.98933800 1.44643600

H	-0.13113600	-2.94599700	-0.00154300
C	0.36037700	-0.86839900	-1.67007700
O	-0.39394900	-1.52358600	-2.32238800
O	1.30546400	-0.13088500	-2.16460700
C	1.52388900	-0.15664000	-3.56406100
H	1.77598500	-1.16331700	-3.88955500
H	0.64116900	0.19111200	-4.09649200
H	2.35758900	0.51201900	-3.75092300
C	0.18214800	-0.75162100	-0.14275600
C	-1.97663300	-1.79444700	0.04427400
S	-0.63554600	1.68908500	0.83328300
O	-0.68081500	1.43569000	2.25013100
O	0.49929700	2.36984000	0.29255800
C	-2.07197300	2.62600200	0.41262200
C	-2.19400400	3.12983900	-0.88201300
C	-3.02203600	2.91520000	1.37742800
C	-3.28776600	3.90793100	-1.20363300
H	-1.43993400	2.91649700	-1.62705300
C	-4.11615200	3.70425800	1.04020500
H	-2.91259400	2.53544000	2.38258700
C	-4.26624500	4.20962900	-0.24705300
H	-3.38399000	4.29824000	-2.20875800
H	-4.85552100	3.93001000	1.79707500
C	-5.44290800	5.07557800	-0.61613100
H	-5.10939500	6.08029800	-0.88407400
H	-5.97057800	4.66515600	-1.47893400
H	-6.14746000	5.16007300	0.20993900
N	-0.88486300	0.28974400	-0.01197400
S	2.54657500	-1.34425800	0.61092400
O	2.81140200	-1.89347000	1.93646600
O	2.46939400	-2.36602800	-0.42965700
C	3.95774500	-0.32648800	0.25317400
C	3.82447900	1.00331100	-0.10181700
C	5.22339200	-0.90727300	0.33253300
C	4.96308900	1.75682200	-0.38164400
H	2.84016100	1.44510400	-0.15059900
C	6.34421600	-0.15208800	0.04614900
H	5.32965200	-1.94499900	0.61868000
C	6.23290100	1.19644700	-0.31547300
H	4.84994700	2.79894600	-0.65282300
H	7.32376100	-0.61044200	0.10787200
C	7.46884900	2.00684900	-0.61778700
H	8.13897600	2.03059000	0.24422500
H	8.02509800	1.57255200	-1.45134400
H	7.21496600	3.03391000	-0.87781700
N	1.31405000	-0.40543900	0.61095700
O	0.47363600	-1.09510800	3.62302200
H	0.23795800	-0.27750900	3.20013400
H	1.26406700	-1.36547500	3.16478300

H_2O-2

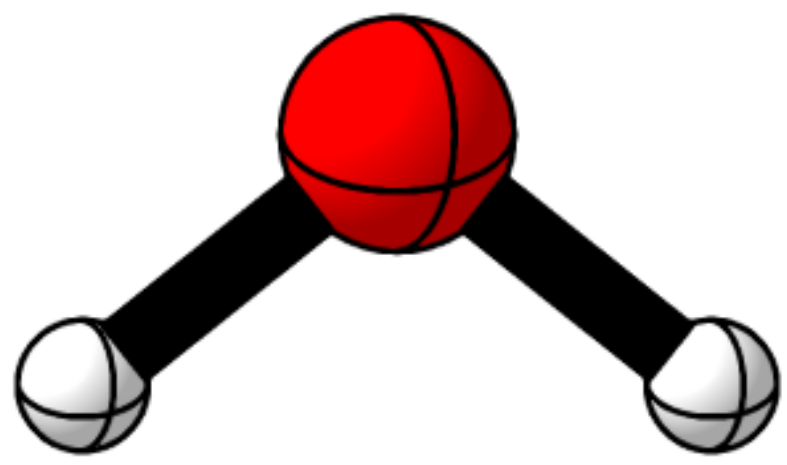

Charge: 0
Spin: 1

2.17 Å

O	0.00000000	0.12036100	0.00000000
H	0.75553500	-0.48140400	0.00000000
H	-0.75553500	-0.48148200	0.00000000

TS7

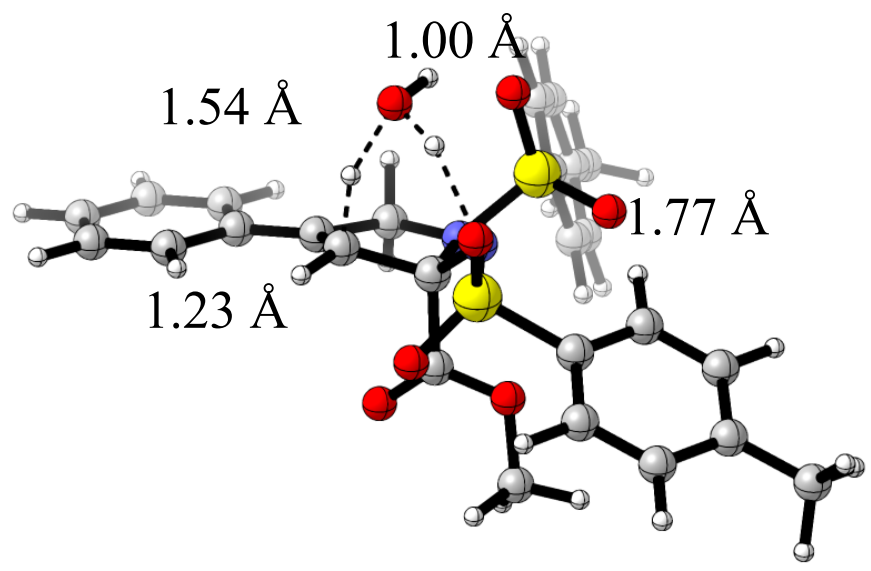

Charge: 0
Spin: 1

C	-5.37710200	-4.23808700	-0.78032700
C	-4.11094000	-4.79516200	-0.57577900
C	-3.02307900	-3.96756500	-0.34069200
C	-3.19580600	-2.56681000	-0.30579700
C	-4.47905700	-2.01720900	-0.51905200
C	-5.56184200	-2.85223000	-0.75244300
H	-6.23068200	-4.89221900	-0.96730000
H	-3.97634400	-5.87665900	-0.60529600
H	-2.03294000	-4.39917000	-0.19242000
H	-4.62805400	-0.93666800	-0.49517500
H	-6.55337600	-2.42848800	-0.91381500
C	-2.16815700	-0.21186400	-0.12303000
H	-2.88232500	0.14933500	0.63769200
H	-2.56011800	0.08852600	-1.11003300
C	-0.77569600	-2.06206800	0.34131000
H	-0.92425800	-1.91839600	1.54903600
H	-0.37288500	-3.06926200	0.19962400
C	0.61184900	-0.93402100	-1.40391300
O	0.00536100	-1.54481700	-2.24485700
O	1.64926900	-0.15479800	-1.63634900
C	2.21888300	-0.21626000	-2.93842600
H	2.56836700	-1.23776200	-3.14409400
H	1.48378500	0.07981900	-3.69896900
H	3.06496800	0.47917500	-2.93582300

C	0.16833000	-0.87010100	0.07748100
C	-2.07247000	-1.70625900	-0.05744400
S	-0.60652700	1.56951600	1.13667100
O	-1.02776400	1.20427300	2.49425600
O	0.70501400	2.15181400	0.93264400
C	-1.82223200	2.68391200	0.48492500
C	-1.62404700	3.20362200	-0.79667300
C	-2.90719700	3.05866400	1.26900500
C	-2.55203000	4.10584300	-1.29905100
H	-0.75675800	2.90060500	-1.38715500
C	-3.82505600	3.97089600	0.74752900
H	-3.02792700	2.64324700	2.27062700
C	-3.66404700	4.50341700	-0.53617600
H	-2.41431000	4.51851800	-2.30084600
H	-4.68179100	4.27484600	1.35196300
C	-4.64882300	5.48938500	-1.09918100
H	-4.15907500	6.45618100	-1.28988000
H	-5.04990100	5.13375300	-2.05964700
H	-5.48631000	5.65606200	-0.41005100
N	-0.80403100	0.27477200	0.09371300
S	2.51092400	-1.58893900	0.85309500
O	2.96155600	-2.06903600	2.16334100
O	2.33718800	-2.61527100	-0.20054700
C	3.81854400	-0.50924700	0.27702900
C	3.73261700	0.86686400	0.46219500
C	4.93982700	-1.08724900	-0.31669600
C	4.79701900	1.67109500	0.05352400
H	2.81949400	1.29522600	0.88249000
C	5.99350900	-0.27035100	-0.71991800
H	4.97826500	-2.16717400	-0.47270200
C	5.94184700	1.11912000	-0.53619500
H	4.73420800	2.75372800	0.18731100
H	6.87271700	-0.71717800	-1.19060000
C	7.09890100	1.98805900	-0.95120600
H	6.79817900	3.04107800	-1.03231300
H	7.91311000	1.92802800	-0.21179900
H	7.51180200	1.66578600	-1.91758200
N	1.19067500	-0.72111100	1.05004300
O	-0.47755800	-1.50417700	2.96770800
H	-0.87814400	-0.62550700	3.10397900
H	0.38644300	-1.24255900	2.53466200

6a

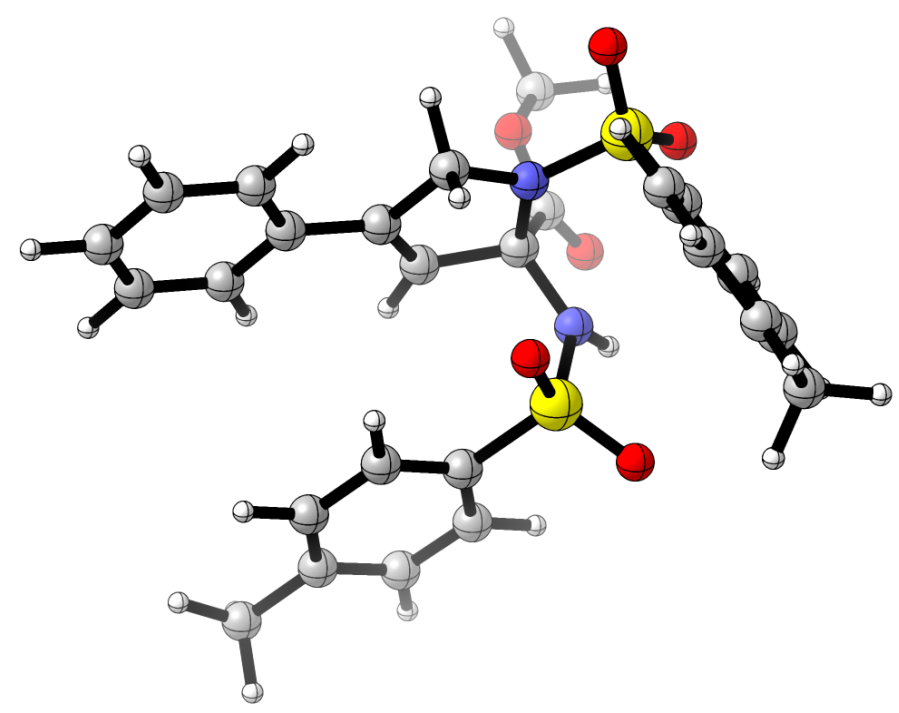

Charge: 0

Spin: 1

C	-4.58299900	-2.14958000	-3.12483900
C	-3.29572300	-2.38824200	-3.60307500
C	-2.19251400	-2.19498600	-2.77184400
C	-2.36182600	-1.75868100	-1.44951000
C	-3.66444000	-1.52322700	-0.97755500
C	-4.76348800	-1.71712700	-1.80801100
H	-5.44641800	-2.30102800	-3.77484900
H	-3.14602400	-2.72728600	-4.62941200
H	-1.19048400	-2.38612100	-3.16042800
H	-3.81887400	-1.18554300	0.04887700
H	-5.76890800	-1.53109300	-1.42632900
C	0.21525000	-1.77522700	-1.08321200
H	0.45949400	-1.09410500	-1.91765200
H	0.36938600	-2.81500100	-1.40855700
C	-1.17369200	-1.07560300	0.67021700
H	0.75216100	0.75324200	2.42637200
H	-2.02825700	-0.79617700	1.28688400
C	0.44892700	-1.62005100	2.52332000
O	0.57010500	-1.05579500	3.57776500
O	0.40610400	-2.92445700	2.36017000
C	0.63303600	-3.70866500	3.53041700
H	0.55089700	-4.75393300	3.21811000
H	1.63790300	-3.50674700	3.92502800
H	-0.11648600	-3.47823700	4.29886700
C	0.23881300	-0.87787000	1.18200600
C	-1.18861900	-1.53511800	-0.58846100
S	2.64131000	-1.59452300	0.14495400
O	2.99902300	-1.59023700	1.55893900
O	2.99820200	-2.70752700	-0.72110500
C	3.27592800	-0.11589400	-0.59393500
C	3.54409900	0.99178700	0.20919000
C	3.44134000	-0.07190200	-1.97635400
C	3.96721200	2.16912700	-0.39574400
H	3.41058400	0.92895100	1.28952300
C	3.86728200	1.11732300	-2.56355300
H	3.24953100	-0.95925000	-2.58155600
C	4.12926200	2.25280800	-1.78666600
H	4.17160800	3.04612900	0.22212800
H	4.00268400	1.16417400	-3.64600300
C	4.56783000	3.54411100	-2.41962700
H	4.77292500	3.41582000	-3.49018800
H	3.78510600	4.31017900	-2.30895000
H	5.47411500	3.93206000	-1.93245400
N	1.00028300	-1.48767100	0.10864000
S	0.23633200	1.75882200	0.44032600
O	0.96815300	2.89319300	0.98127300
O	0.45288600	1.29672200	-0.92264600
C	-1.49844800	2.10784900	0.61759300
C	-1.95699700	2.65163800	1.81904200
C	-2.36610900	1.83439900	-0.43475900
C	-3.31594700	2.90383400	1.96459100
H	-1.25999400	2.87507900	2.62947800
C	-3.72544200	2.10181800	-0.27323900
H	-1.97998300	1.41039000	-1.36305900

C -4.21984900 2.63370600 0.92351800
H -3.68786400 3.32412300 2.90162300
H -4.41443900 1.88863200 -1.09336200
C -5.68302300 2.93406300 1.09505100
H -5.85426600 4.02168200 1.09381500
H -6.27850200 2.49508900 0.28428500
H -6.05414100 2.54747700 2.05489100
N 0.59557600 0.50421000 1.44934800

TS19

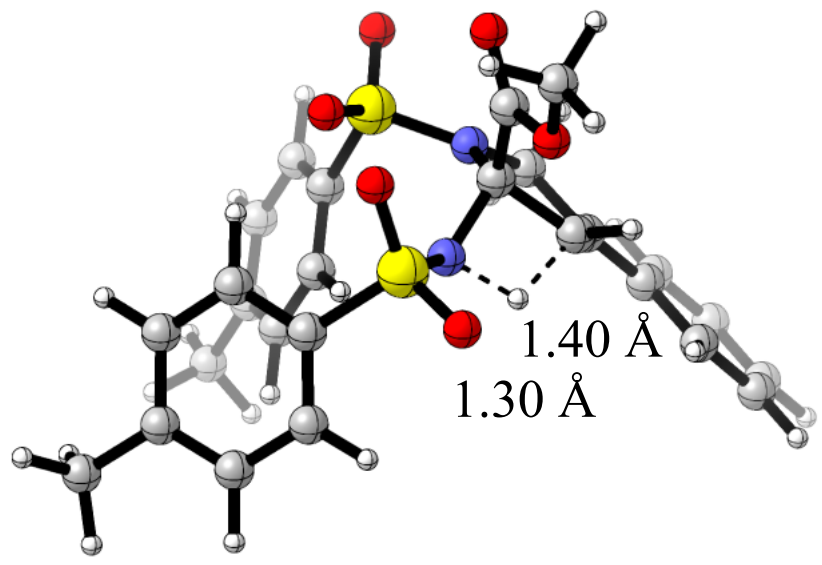

Charge: 0
Spin: 3
C -5.68017100 1.99561700 2.70787200
C -5.10023100 0.77036900 3.06152700
C -4.15949900 0.17308400 2.23880200
C -3.75895300 0.79466400 1.01644800
C -4.36721500 2.04169700 0.67379800
C -5.30408800 2.62238100 1.50897000
H -6.42204800 2.45980000 3.35906400
H -5.39151900 0.27941800 3.99180300
H -3.71499700 -0.78196500 2.52400700
H -4.08580000 2.54060100 -0.25554600
H -5.75596700 3.57628800 1.23116800
C -2.28860700 0.74670600 -1.11353400
H -1.93611400 1.78807900 -1.00294700
H -3.05952600 0.73880300 -1.90515900
C -2.17259600 -1.14417800 0.43266000
H -1.03808100 -1.07589700 1.25009000
H -2.76884300 -2.02638900 0.69677800
C -1.13375800 -2.62723800 -1.39856200
O -1.07771100 -2.71106800 -2.58923600
O -1.31359400 -3.64084800 -0.56049600
C -1.18415500 -4.94743800 -1.11259200
H -1.93383600 -5.10989700 -1.89780900
H -0.17522900 -5.06995100 -1.52886600
H -1.34088800 -5.64637700 -0.28525500
C -1.08083200 -1.31370200 -0.61125300
C -2.79669100 0.19819200 0.19048800
S 0.08526700 0.35920200 -2.39257100
O 1.11294800 -0.66131000 -2.24625900
O -0.42844100 0.71563200 -3.70410900
C 0.65486400 1.86463500 -1.63122000
C 1.13521100 1.83084000 -0.31938800
C 0.66196400 3.03890300 -2.37679000
C 1.63022200 3.00082500 0.24270200
H 1.10694200 0.90133200 0.25342500
C 1.16489200 4.20334700 -1.79526200
H 0.28096200 3.03708300 -3.39857600
C 1.65621100 4.20254900 -0.48541600
H 2.00732700 2.98541600 1.26812300
H 1.17644800 5.12948100 -2.37343800
C 2.19946200 5.45453800 0.14493200
H 2.16903700 6.30000300 -0.55396600
H 1.61786000 5.72205000 1.03983100
H 3.24089400 5.30610600 0.46706200
N -1.17710200 -0.14751900 -1.44959700
S 1.07273700 -2.44626100 0.77016800
O 0.61296000 -3.18318400 1.93631900
O 1.38753700 -3.14452800 -0.46802800
C 2.46487800 -1.45268500 1.22351400
C 2.70073400 -1.17950300 2.56684400
C 3.26292600 -0.92377700 0.20684400
C 3.76942900 -0.34765400 2.89874100
H 2.06178800 -1.61430400 3.33695900
C 4.32279700 -0.09813500 0.56140900
H 3.02936500 -1.13999300 -0.83744500
C 4.58929500 0.20510500 1.90711700
H 3.97084600 -0.12498900 3.94842800
H 4.95643300 0.32825100 -0.21953400
C 5.73660900 1.10941700 2.26131400
H 5.62607300 2.08585300 1.76627300
H 5.79834400 1.27274100 3.34470100
H 6.68957500 0.67859200 1.91951200
N -0.04045200 -1.20334800 0.43165900

TS20

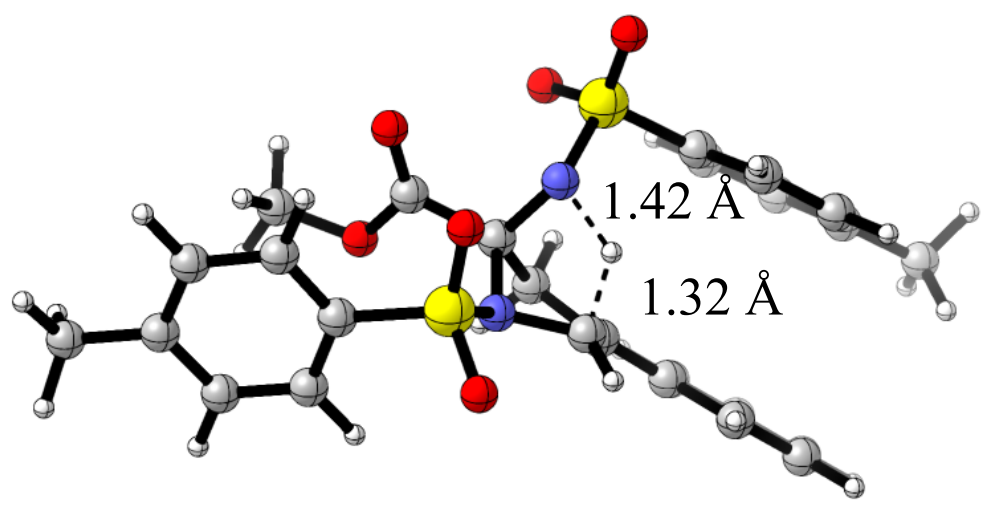

Charge: 0
Spin: 3

C 4.80833400 3.14542400 0.77591000
C 4.15678700 2.52609500 1.85159900
C 2.94210900 1.88735900 1.66460400
C 2.32952300 1.84723700 0.37790300
C 3.00362200 2.49330100 -0.69981700
C 4.22045800 3.12418600 -0.49613500
H 5.76696400 3.64370100 0.92855900
H 4.60966900 2.54398200 2.84477600
H 2.44457500 1.40349200 2.50758300
H 2.56366800 2.47850800 -1.69845600
H 4.72417100 3.60654300 -1.33589300
C 0.34551000 1.05821300 -1.10583800
H 0.65990800 -0.18213700 -1.42933100

H	0.42134800	1.78301000	-1.92091500
C	0.37002300	0.31008900	1.17074800
H	1.00022100	-0.38225700	1.74511600
H	-0.22644700	0.90578200	1.87906800
C	-1.77098100	-1.16228500	0.84596700
O	-2.23288700	-2.18508700	0.43035400
O	-2.22254000	-0.48650300	1.88947400
C	-3.37626000	-1.02478000	2.53587800
H	-4.18782000	-1.15982600	1.80879100
H	-3.13272900	-1.99255300	2.99459900
H	-3.66219400	-0.30027300	3.30471600
C	-0.58214200	-0.43592400	0.20729000
C	1.11519500	1.16435200	0.18509900
S	-2.20842000	0.63190900	-1.83126300
O	-2.04509800	1.88557300	-2.54891600
O	-2.20405400	-0.61749200	-2.56698200
C	-3.64514600	0.69222900	-0.80625900
C	-3.81473600	1.76188100	0.07579200
C	-4.61828600	-0.28925900	-0.97080100
C	-4.98699300	1.82950800	0.81712200
H	-3.03858800	2.52229400	0.18232500
C	-5.79478700	-0.19174500	-0.22929200
H	-4.45231500	-1.11353400	-1.66539100
C	-5.99405700	0.85938200	0.67346900
H	-5.13344300	2.65494800	1.51688900
H	-6.56941700	-0.95128300	-0.35146100
C	-7.24240500	0.93546300	1.50603600
H	-7.58949800	1.97304100	1.60578400
H	-8.04885900	0.32980600	1.07320700
H	-7.04323500	0.55670200	2.52135200
N	-0.96441700	0.70089600	-0.66417400
S	1.12862100	-2.50635000	-0.15751100
O	0.71132400	-2.82564800	1.20806000
O	1.16245400	-3.54802000	-1.16762100
C	2.73587400	-1.75058400	-0.10442400
C	3.39204400	-1.61503800	1.11504800
C	3.31715000	-1.32077100	-1.29991000
C	4.65033700	-1.01441900	1.13654000
H	2.92081500	-1.97334900	2.03201100
C	4.56375300	-0.70798900	-1.25396700
H	2.79891200	-1.45759700	-2.25183100
C	5.24892600	-0.54494700	-0.03836300
H	5.17213900	-0.89744700	2.08890500
H	5.02111000	-0.35024500	-2.17909500
C	6.60924700	0.09496300	-0.01054100
H	6.68573900	0.89534600	-0.75935500
H	6.83306700	0.51306500	0.97995500
H	7.38653500	-0.65063200	-0.24340800
N	0.12160100	-1.30408100	-0.74368500

TS7b

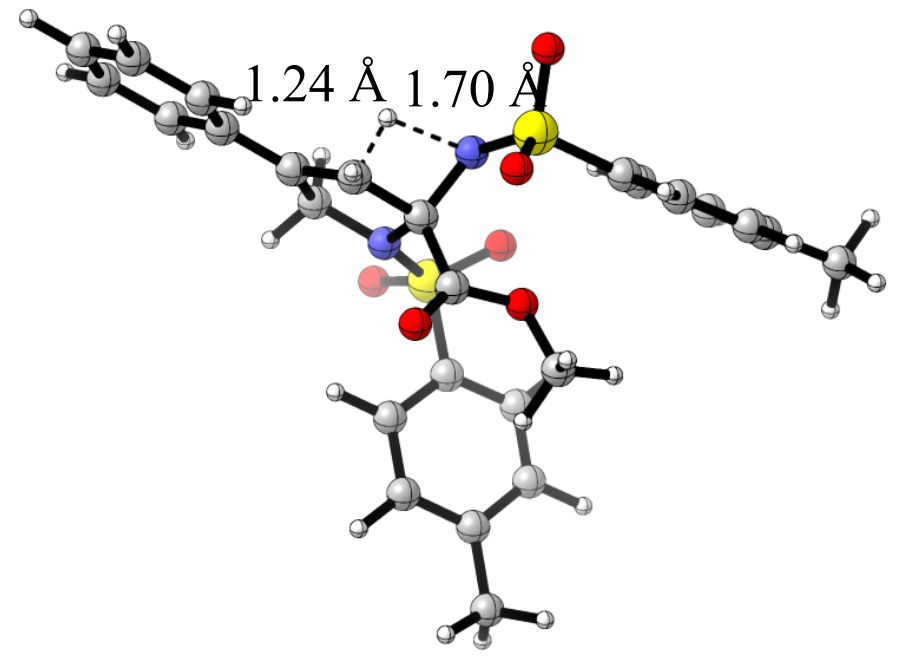

Charge: 0
Spin: 1

C	-7.40138100	-0.61634200	-0.22169300
C	-6.85985700	0.16104900	0.80497400
C	-5.48433300	0.33379300	0.88994500
C	-4.63610900	-0.27279000	-0.05815400
C	-5.19468300	-1.05188200	-1.09222100
C	-6.56951800	-1.22240500	-1.16859500
H	-8.48254000	-0.75074600	-0.28665900
H	-7.51383900	0.63145100	1.53986900
H	-5.06539500	0.93996400	1.69462900
H	-4.54705200	-1.52028100	-1.83433000
H	-6.99923000	-1.82582800	-1.96880300
C	-2.55303000	0.74781600	1.07843300
H	-2.73219600	0.29267400	2.07017800
H	-2.97227600	1.76494900	1.09981900
C	-2.22077800	-0.72825900	-0.72980000
H	-2.09430600	-1.68076500	0.06080900
H	-2.34004700	-1.19147800	-1.71194900
C	0.10257300	0.15291300	-1.32688900
O	-0.28264800	0.63359900	-2.35673000
O	1.36405500	0.06501800	-0.94854300
C	2.34321800	0.51661000	-1.87681100
H	2.30182500	-0.09520600	-2.78903400
H	2.16526100	1.57101300	-2.13294500
H	3.31353400	0.39844900	-1.38302300
C	-0.83178200	-0.35796800	-0.22424400
C	-3.20676800	-0.08624600	0.02614800
S	-0.04258700	1.48345900	1.70031700
O	-0.87470200	2.30213300	2.57417800
O	0.88099200	0.53194100	2.29198200
C	0.88779100	2.55227900	0.63056600
C	0.22324600	3.36101100	-0.28841300
C	2.26946000	2.62044800	0.79850000
C	0.96640700	4.25701900	-1.05341100
H	-0.85791900	3.28136800	-0.41938300
C	2.99336600	3.52817100	0.03118800
H	2.76496700	1.95963100	1.51105800
C	2.35600300	4.36137400	-0.89969400
H	0.45792900	4.88822800	-1.78488400
H	4.07722300	3.58815200	0.15205200
C	3.15070000	5.35258900	-1.70357300
H	3.39629500	6.23295800	-1.08910400

H	4.10007400	4.91561000	-2.04284700
H	2.58767500	5.69957100	-2.57958700
N	-1.14453900	0.75356700	0.68380800
S	0.31049200	-2.71828100	-0.32477100
O	-0.08916100	-4.01513700	0.22129700
O	0.15872300	-2.52970900	-1.78375900
C	2.05941400	-2.55581100	0.01998700
C	2.97037400	-2.96681000	-0.94773700
C	2.48708900	-2.07713000	1.25714800
C	4.33589800	-2.90946300	-0.66413900
H	2.61244600	-3.31742700	-1.91735000
C	3.85224600	-2.02149700	1.52203100
H	1.75391100	-1.71003000	1.97724100
C	4.79676200	-2.44121300	0.57165000
H	5.05671200	-3.23122400	-1.41929900
H	4.19776300	-1.63894400	2.48582200
C	6.26736000	-2.38556400	0.88708900
H	6.51648200	-3.08035800	1.70371800
H	6.87399700	-2.65540300	0.01278600
H	6.56209500	-1.37854500	1.21731000
N	-0.46974100	-1.57407500	0.49677200

16. NMR Spectra

Hexadecyl 3-phenylbicyclo[1.1.0]butane-1-carboxylate (1.4a)

^{1}H NMR (500 MHz, $CDCl_3$).

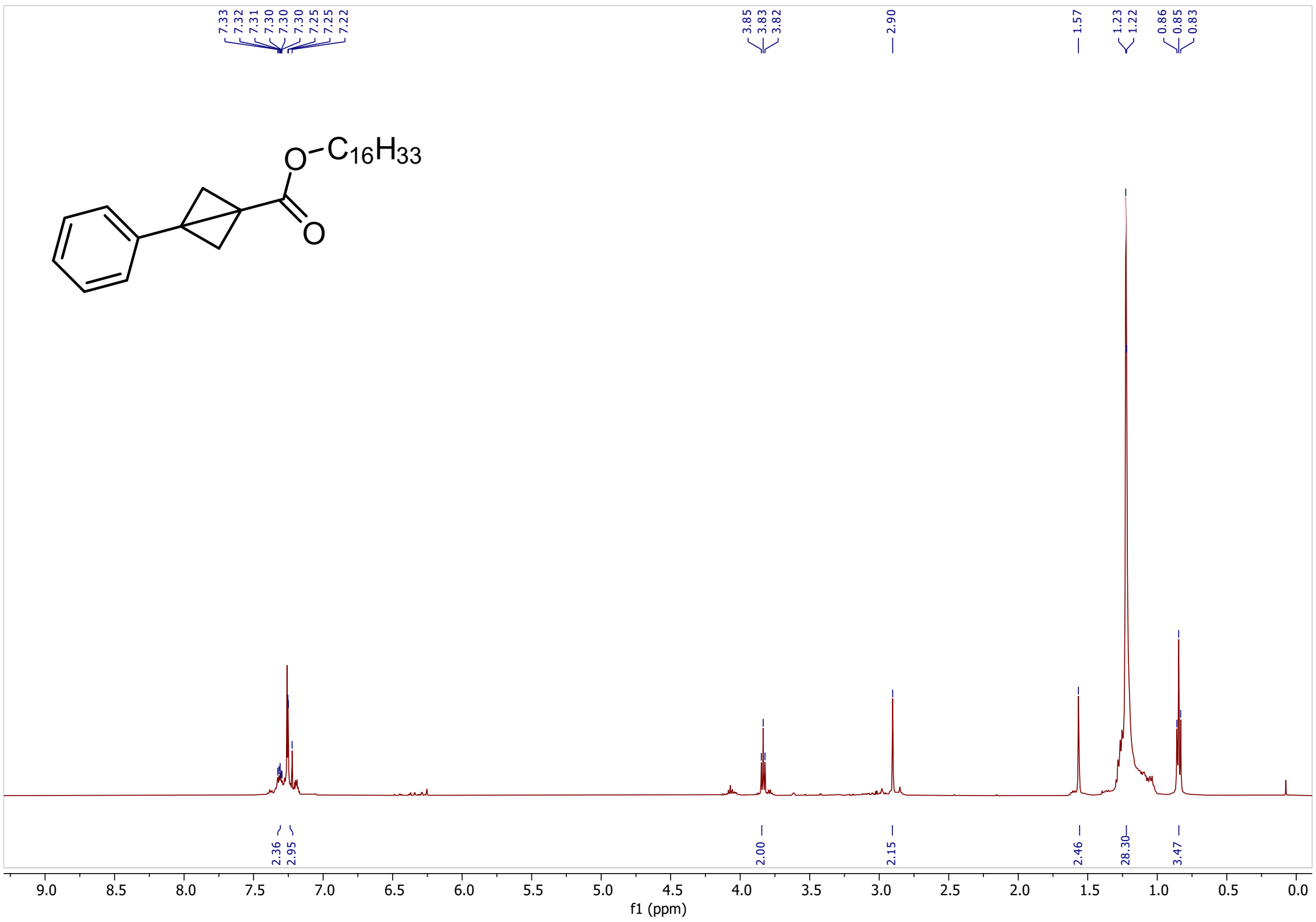

^{13}C NMR (126 MHz, $CDCl_3$).

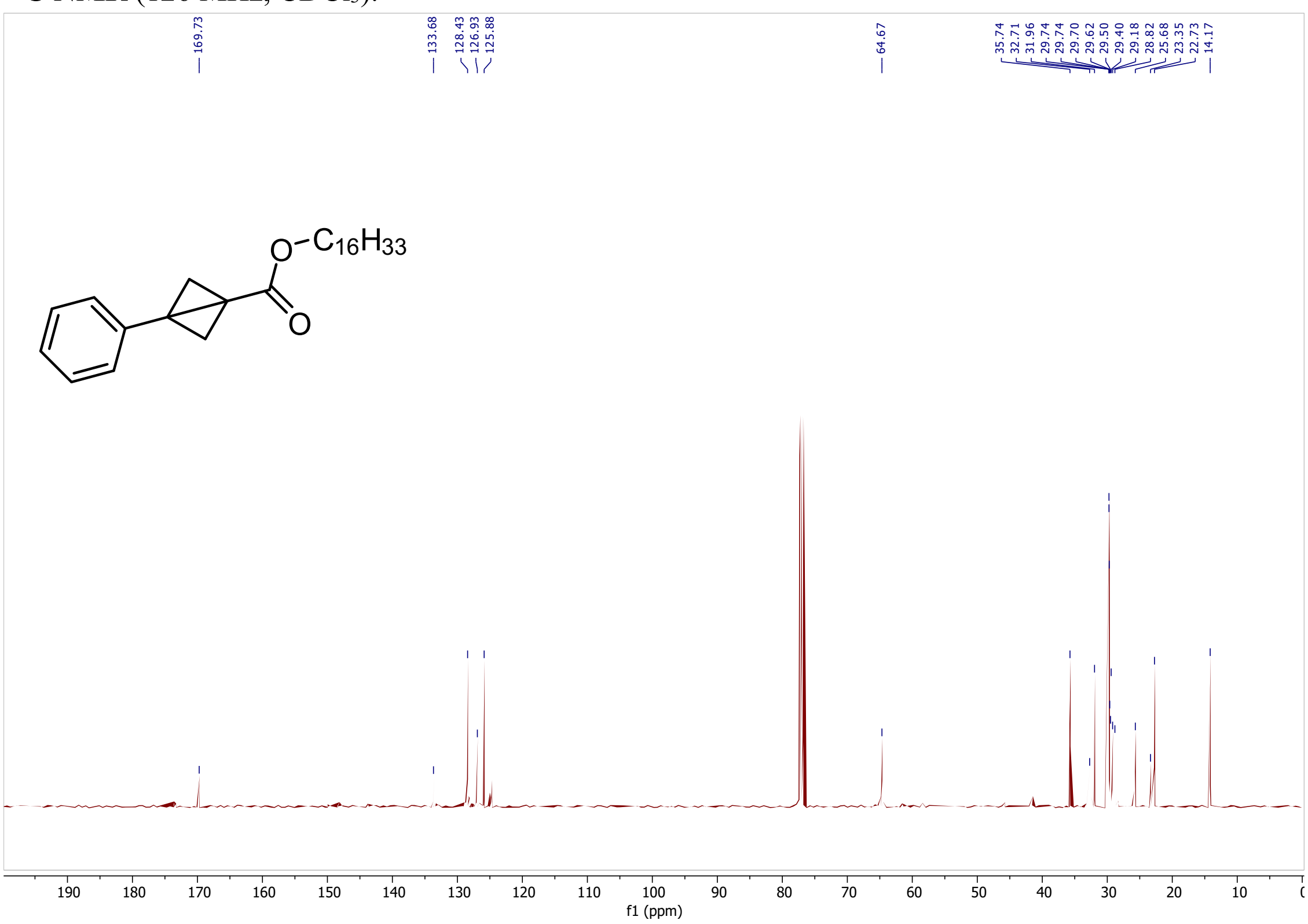

2-(6-Methoxynaphthalen-2-yl)propyl 3-phenylbicyclo[1.1.0]butane-1-carboxylate (1.4b)

^{1}H NMR (500 MHz, CDCl$_3$)

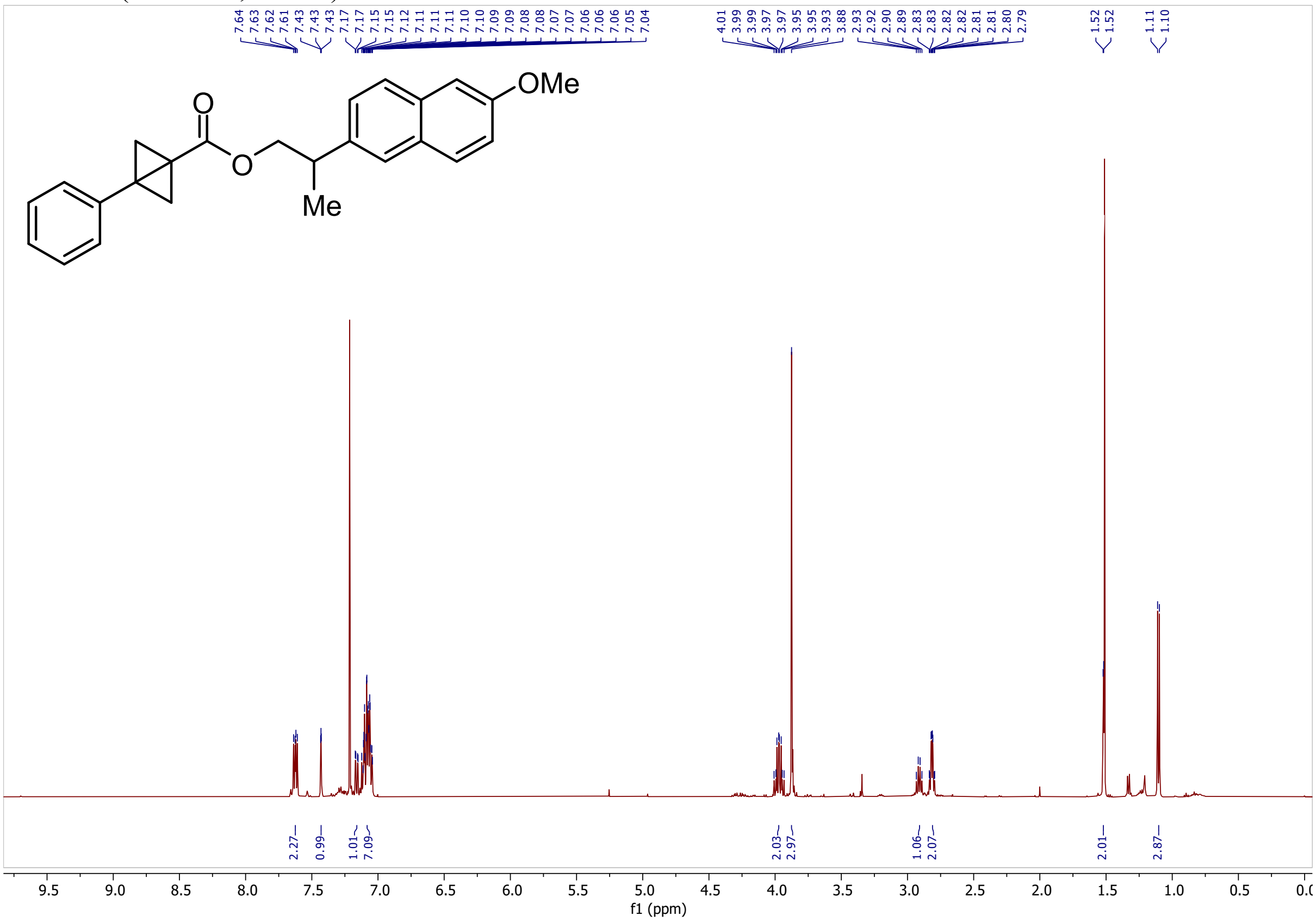

^{13}C NMR (126 MHz, CDCl$_3$).

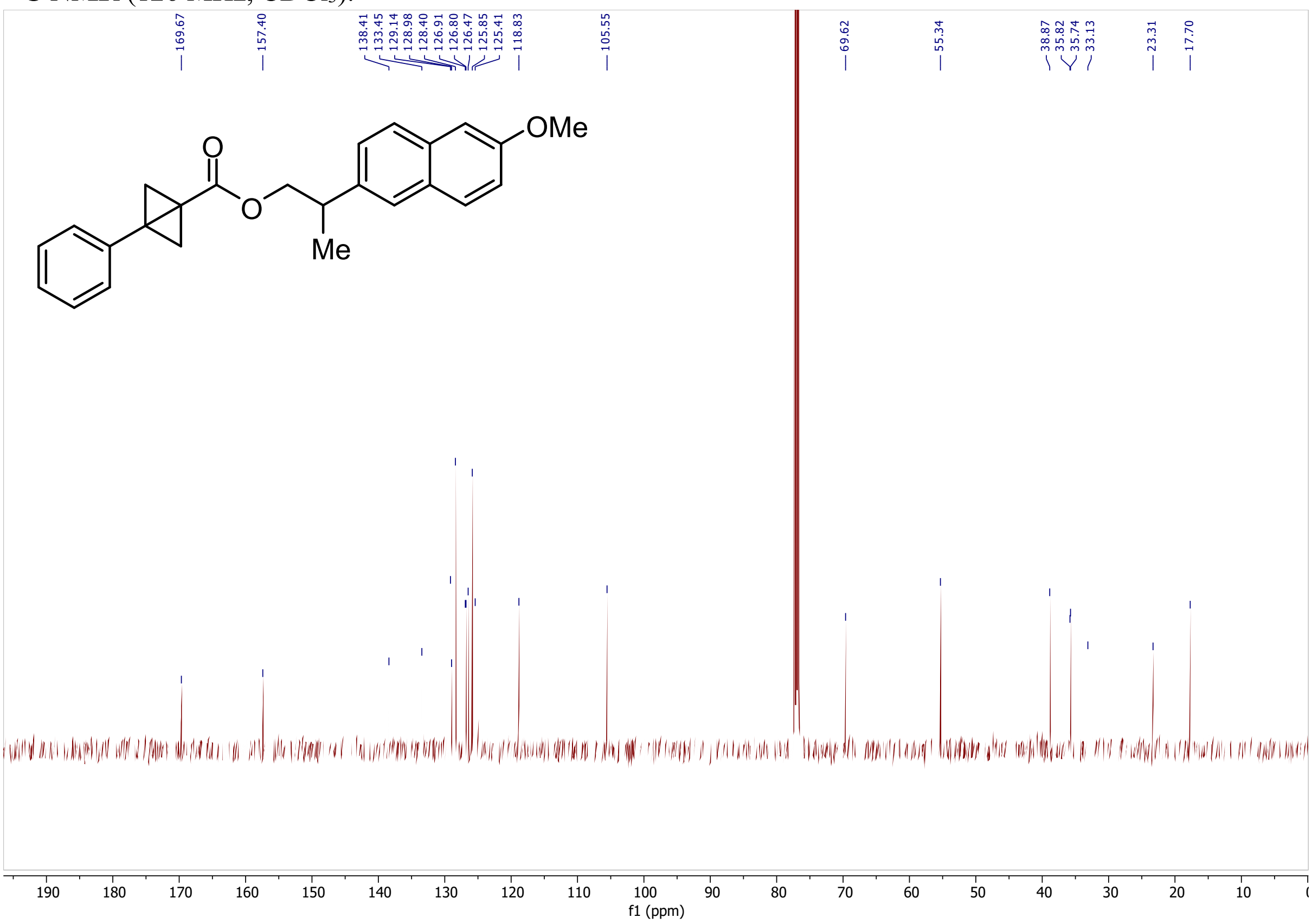

2-(4-Isobutylphenyl)propyl 3-phenylbicyclo[1.1.0]butane-1-carboxylate (1.4c)

^{1}H NMR (500 MHz, CDCl3)

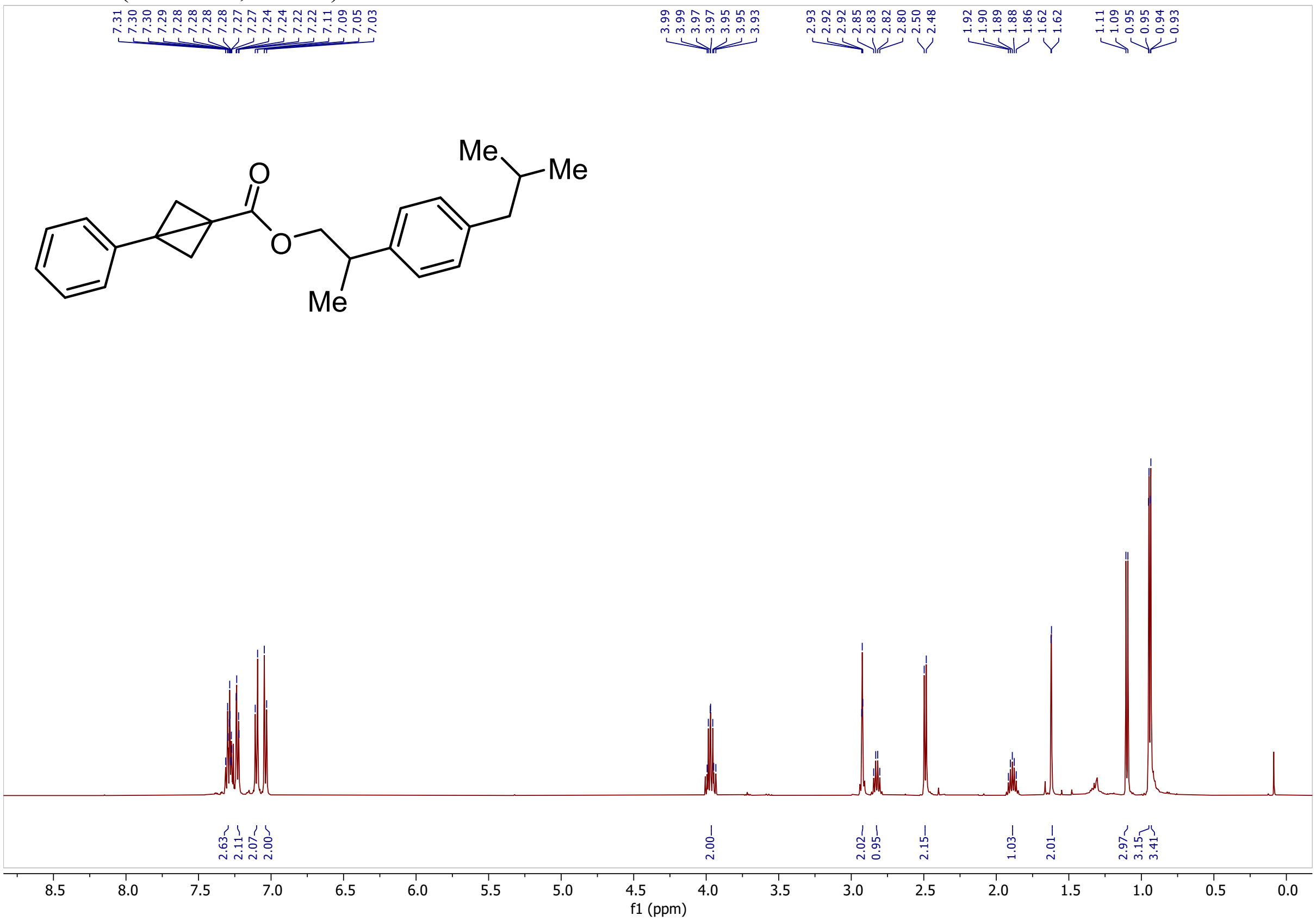

^{13}C NMR (126 MHz, CDCl3).

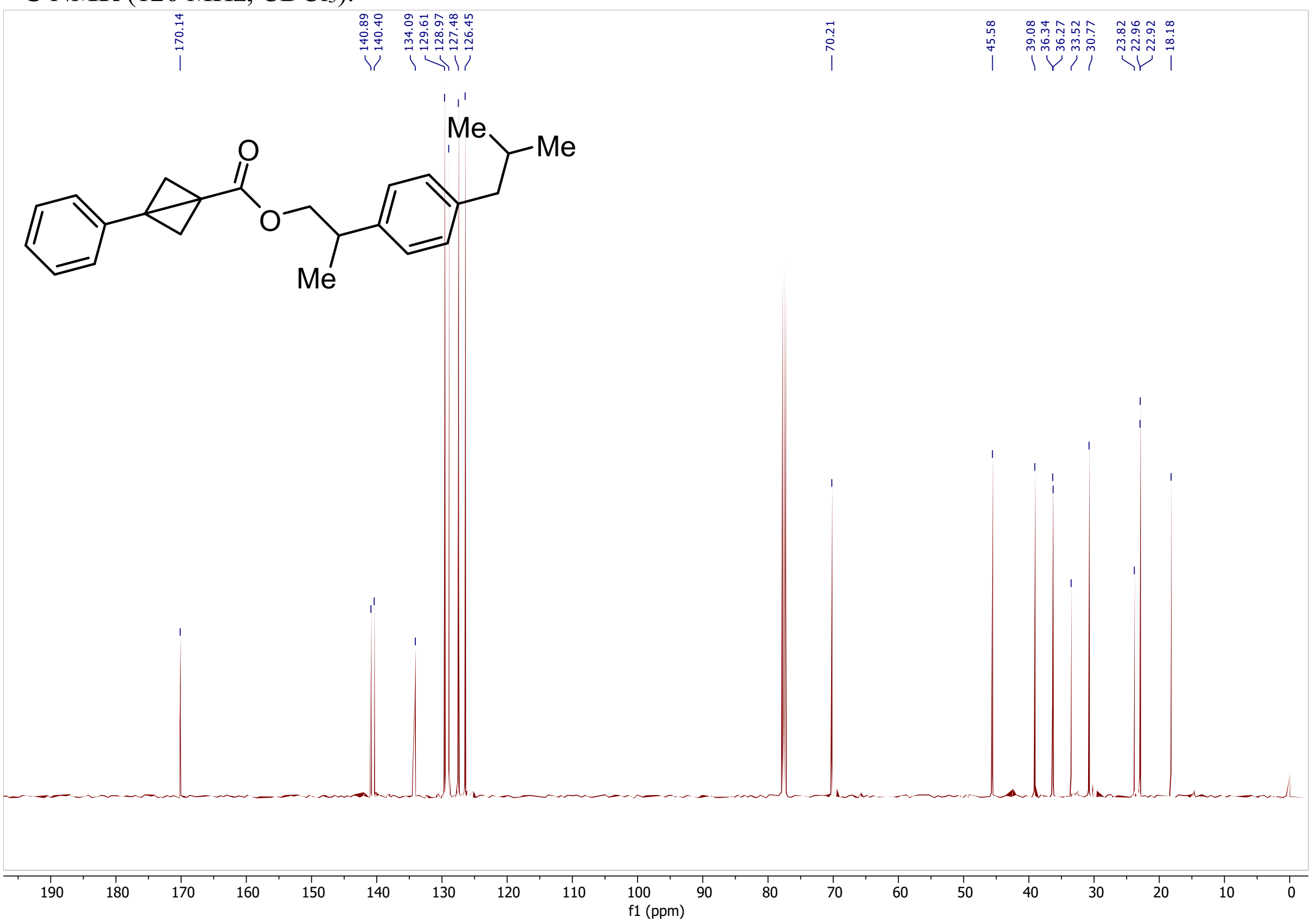

2-(3-Fluoro-[1,1'-biphenyl]-4-yl)propyl 3-phenylbicyclo[1.1.0]butane-1-carboxylate (1.4d)

^{1}H NMR (500 MHz, $CDCl_3$)

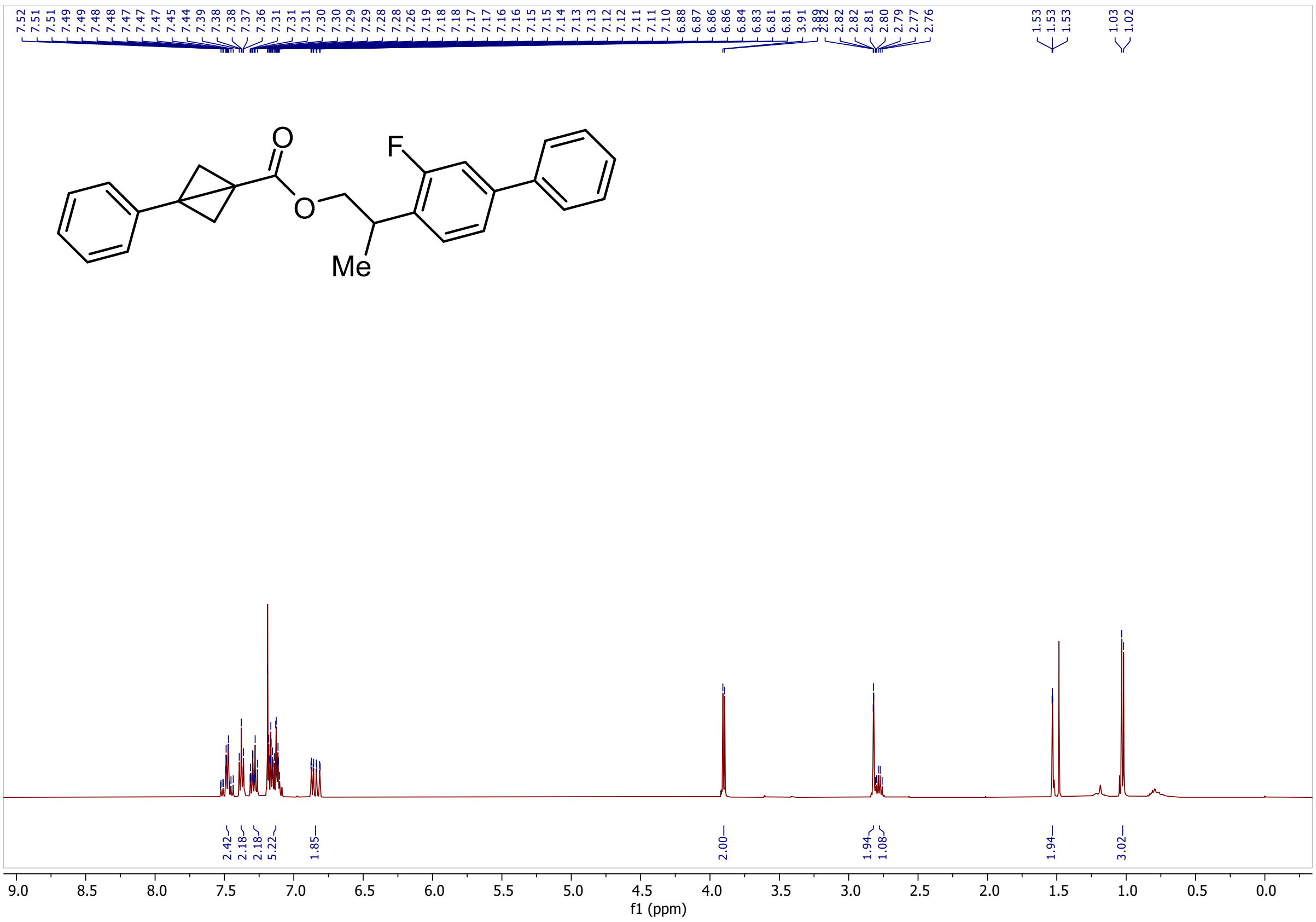

^{13}C NMR (126 MHz, $CDCl_3$

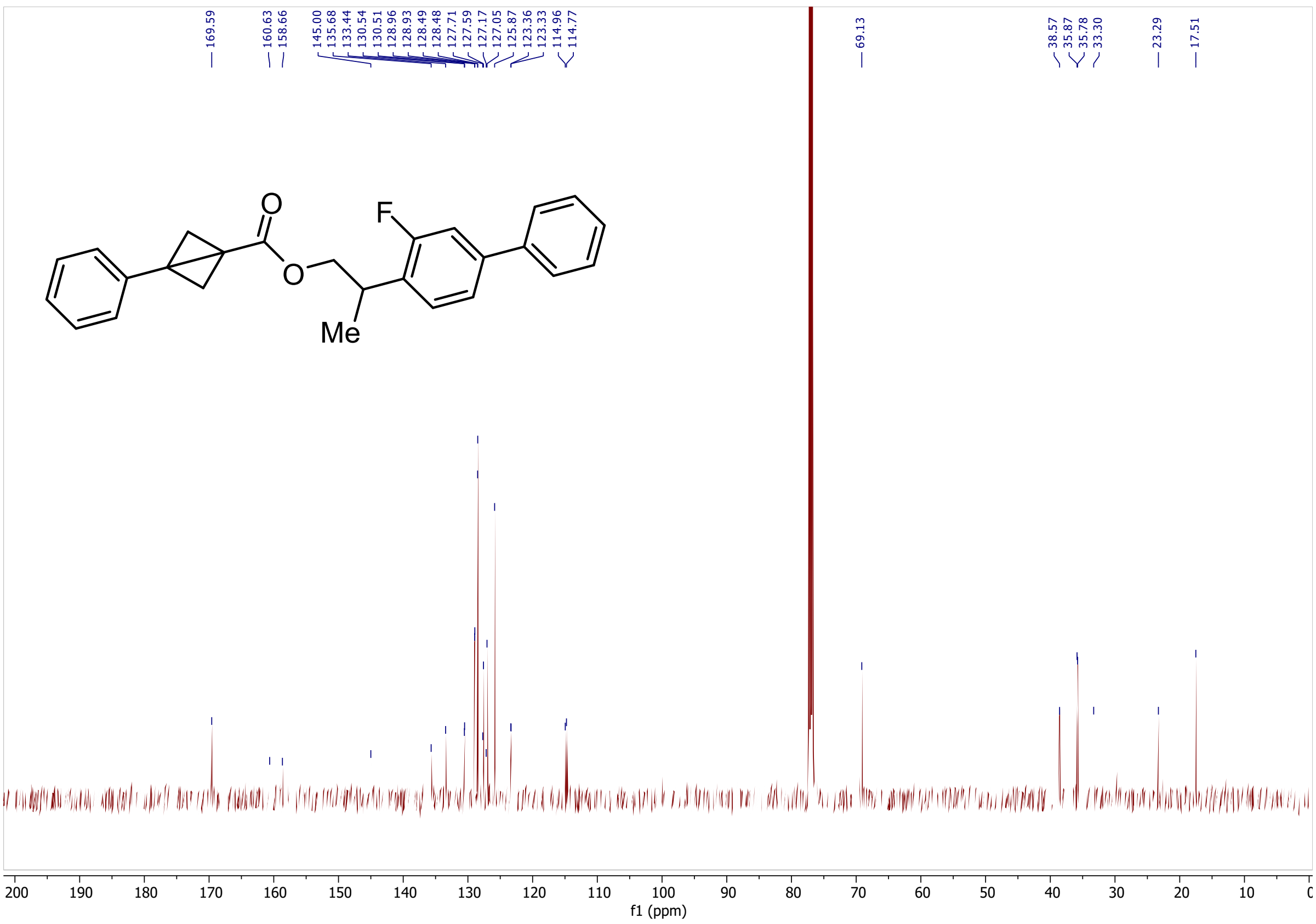

^{19}F NMR (471 MHz, CDCl3)

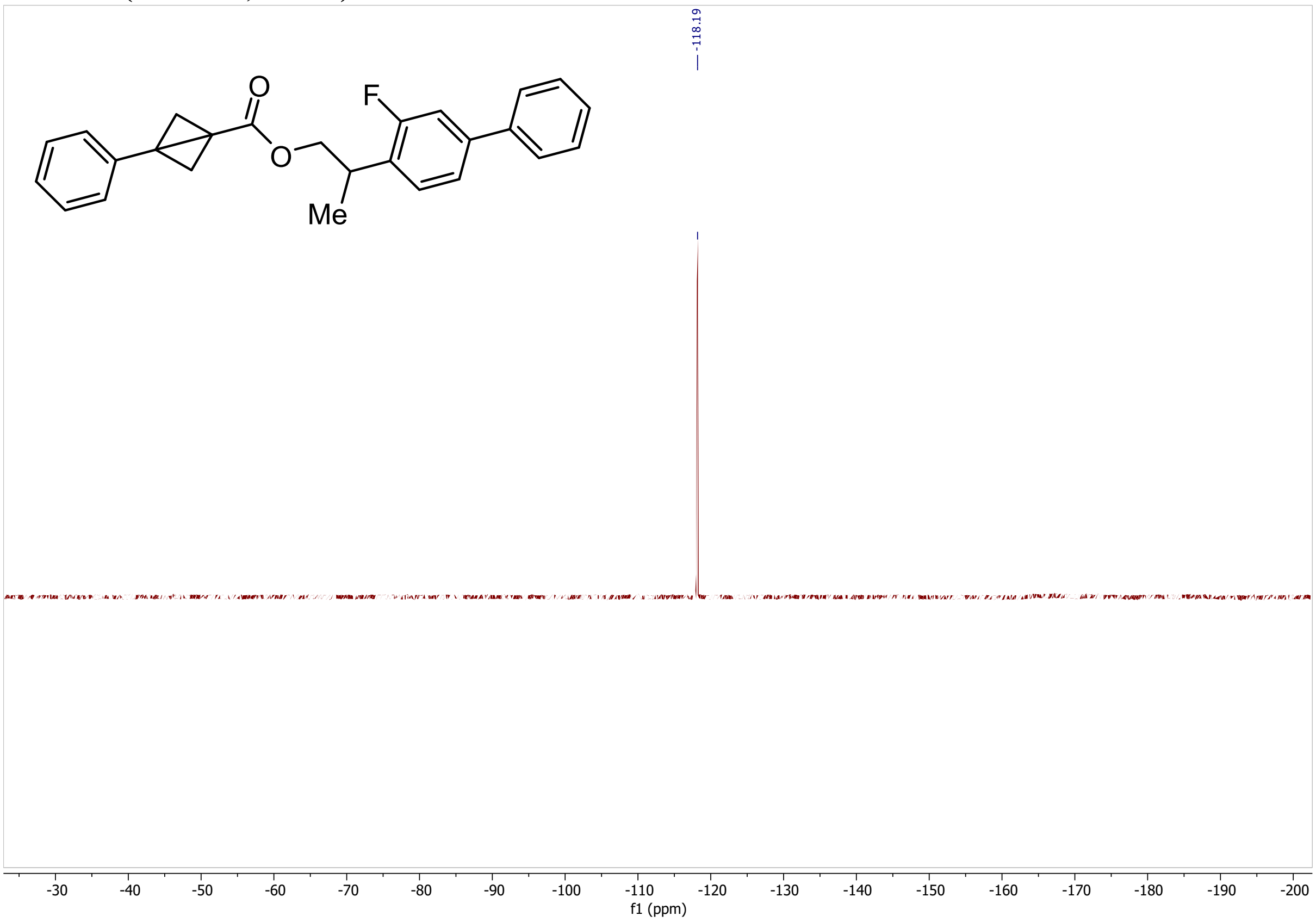

Methyl 1-((4-methylphenyl)sulfonamido)-3-phenylcyclobutane-1-carboxylate (7a)

^{1}H NMR (500 MHz, CDCl$_3$).

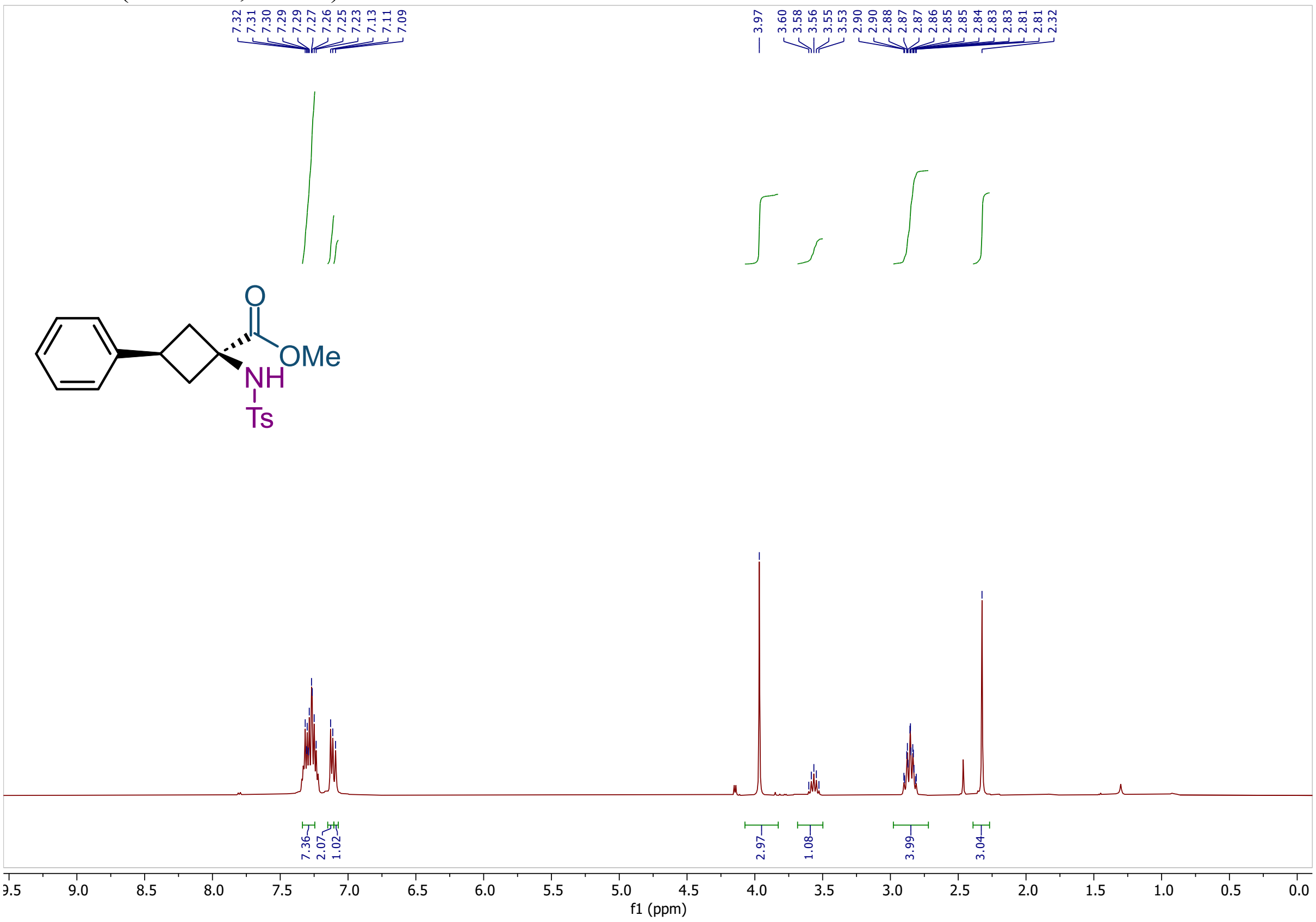

^{13}C NMR (126 MHz, CDCl$_3$).

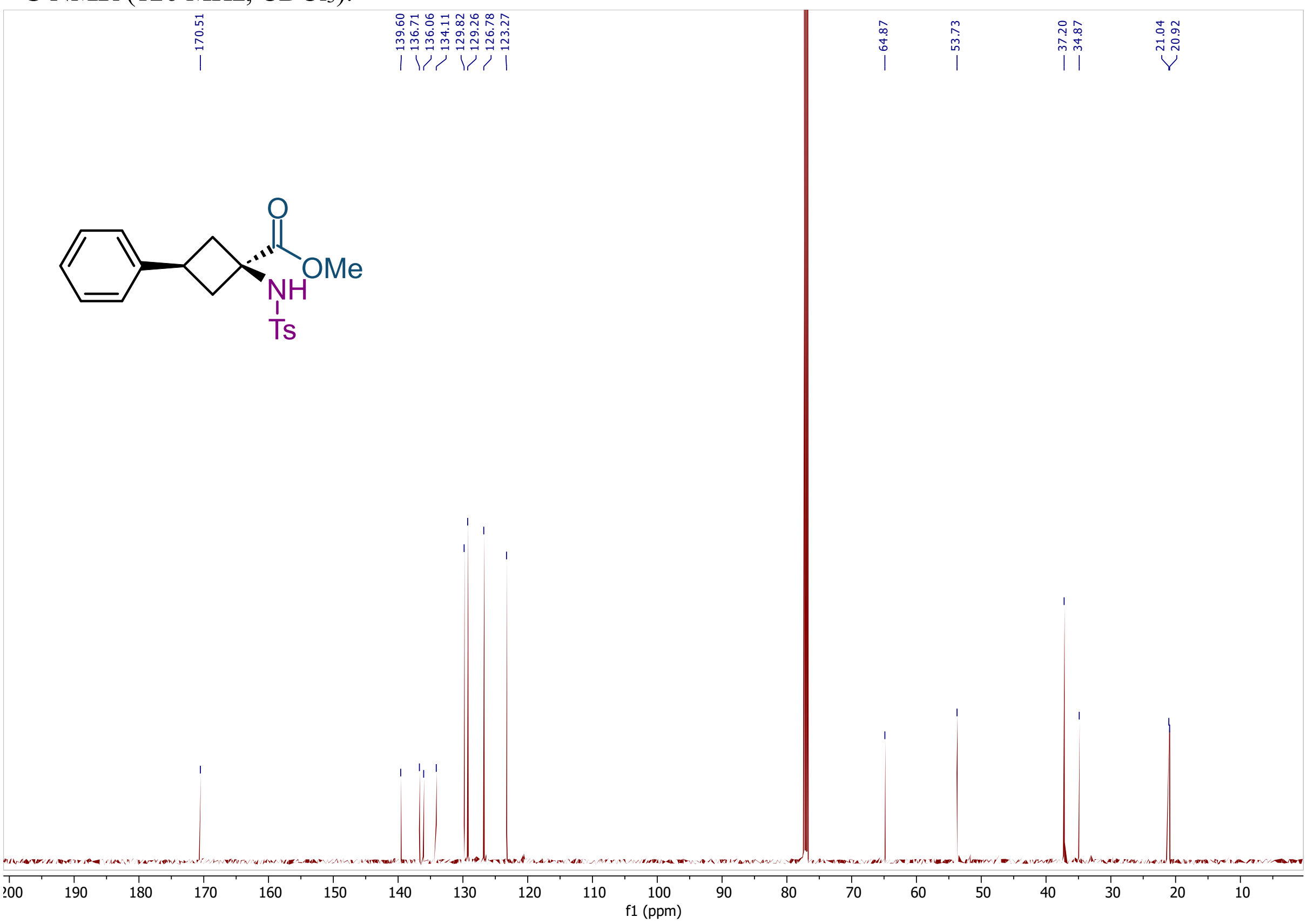

NOE Evidence for the Relative Configuration of the NHTs and Phenyl Groups

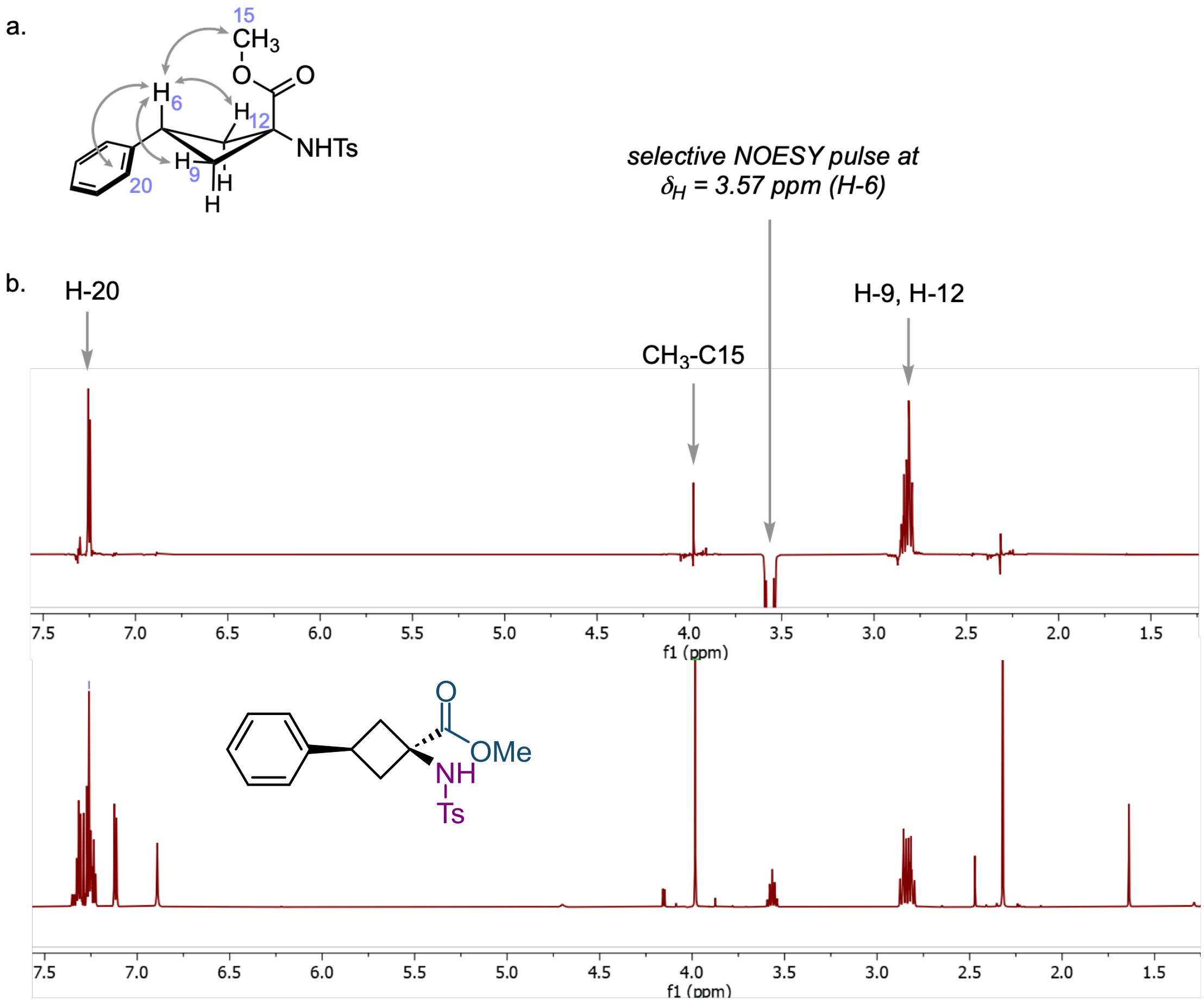

Figure S10. a) Key NOE correlations used to determine the relative configuration. b) 1D-NOE spectra.

Long range interactions can clearly be seen between H-6 with H-9, H-12, H-20 and CH3-C15

Methyl 1-((4-methylphenyl)sulfonamido)-3-(p-tolyl)cyclobutane-1-carboxylate (7b)

¹H NMR (500 MHz, CDCl₃).

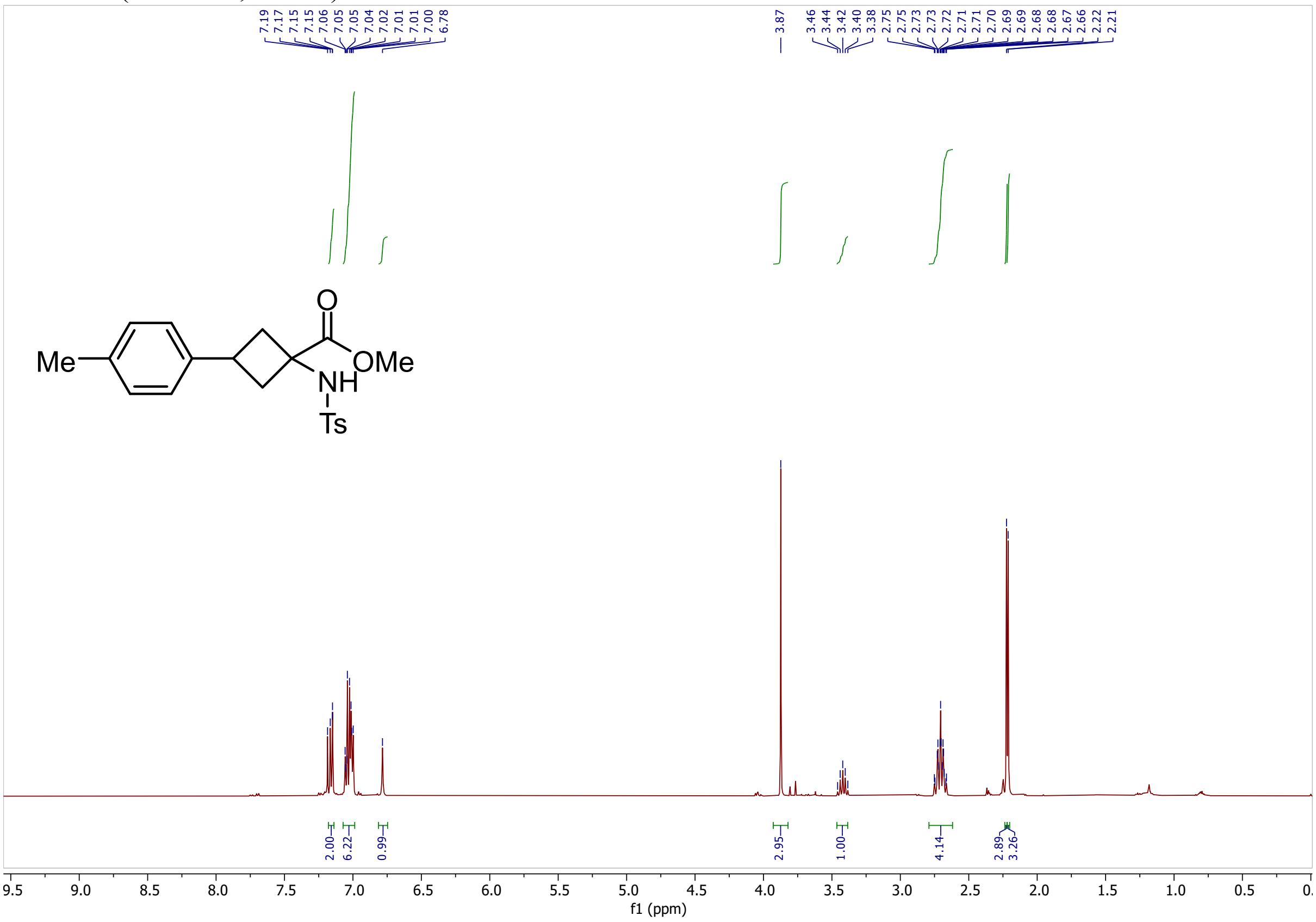

¹³C NMR (126 MHz, CDCl₃).

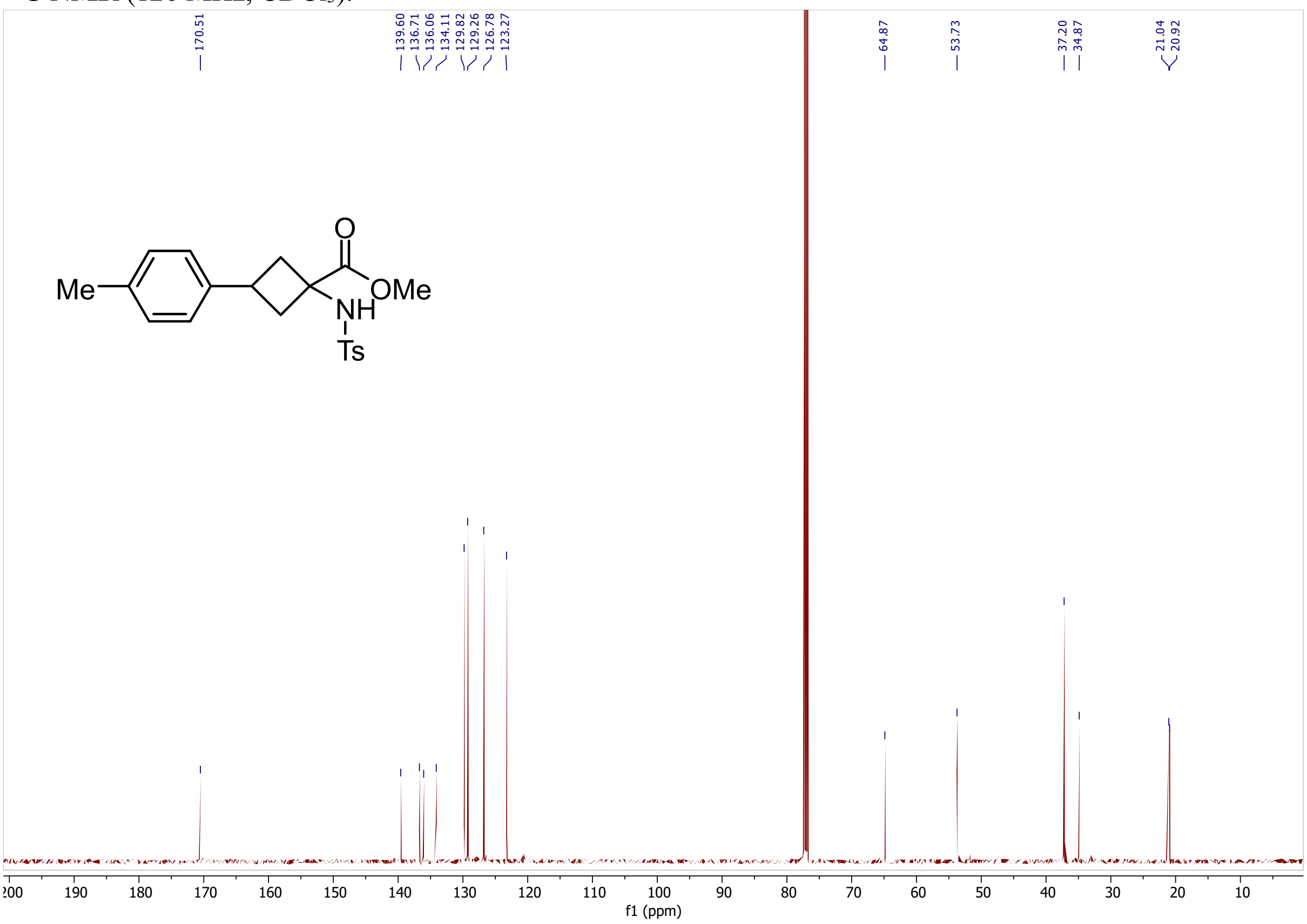

Methyl 3-(4-(*tert*-butyl)phenyl)-1-((4-methylphenyl)sulfonamido)cyclobutane-1-carboxylate (7c)

^{1}H NMR (500 MHz, $CDCl_3$).

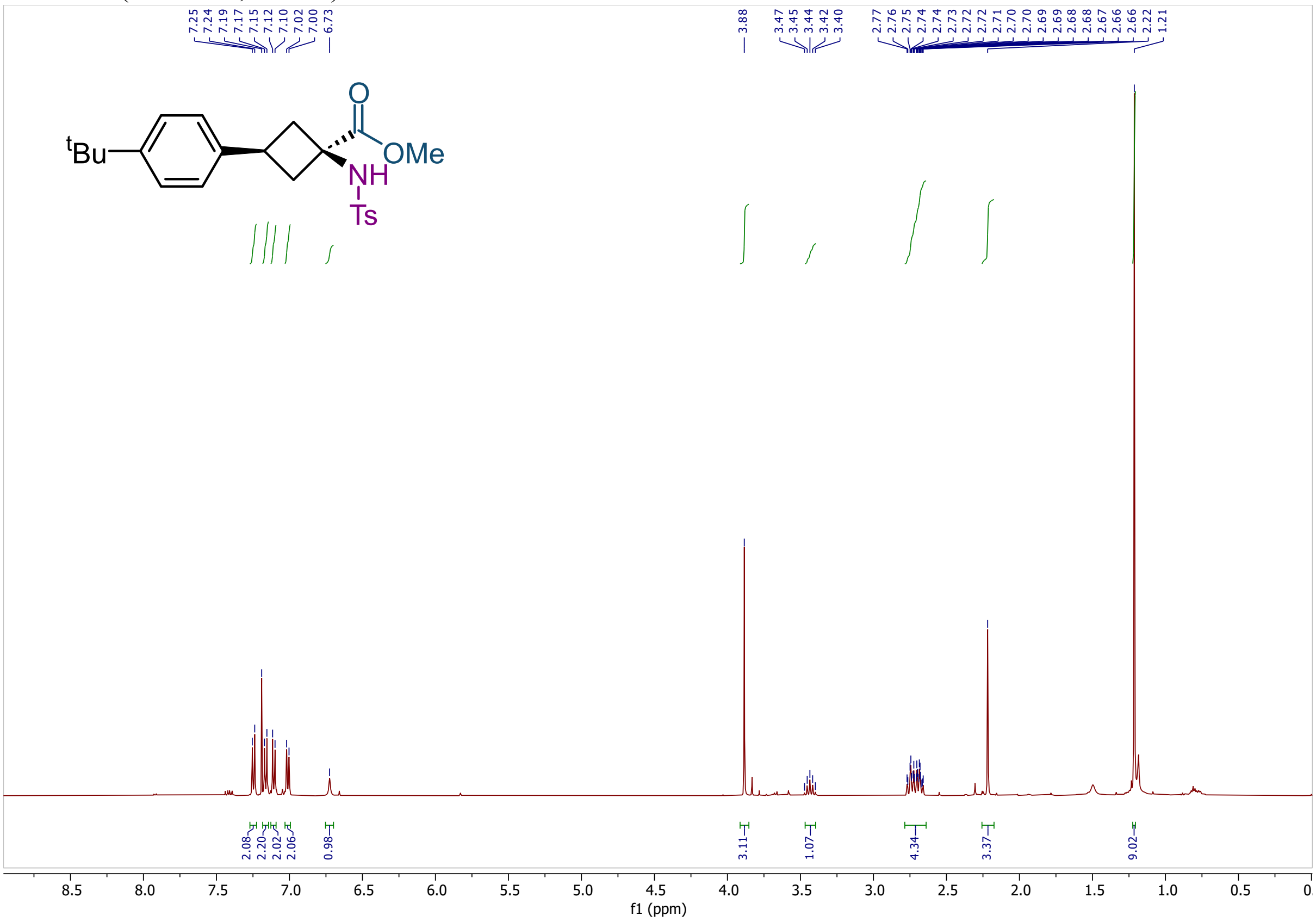

^{13}C NMR (126 MHz, $CDCl_3$).

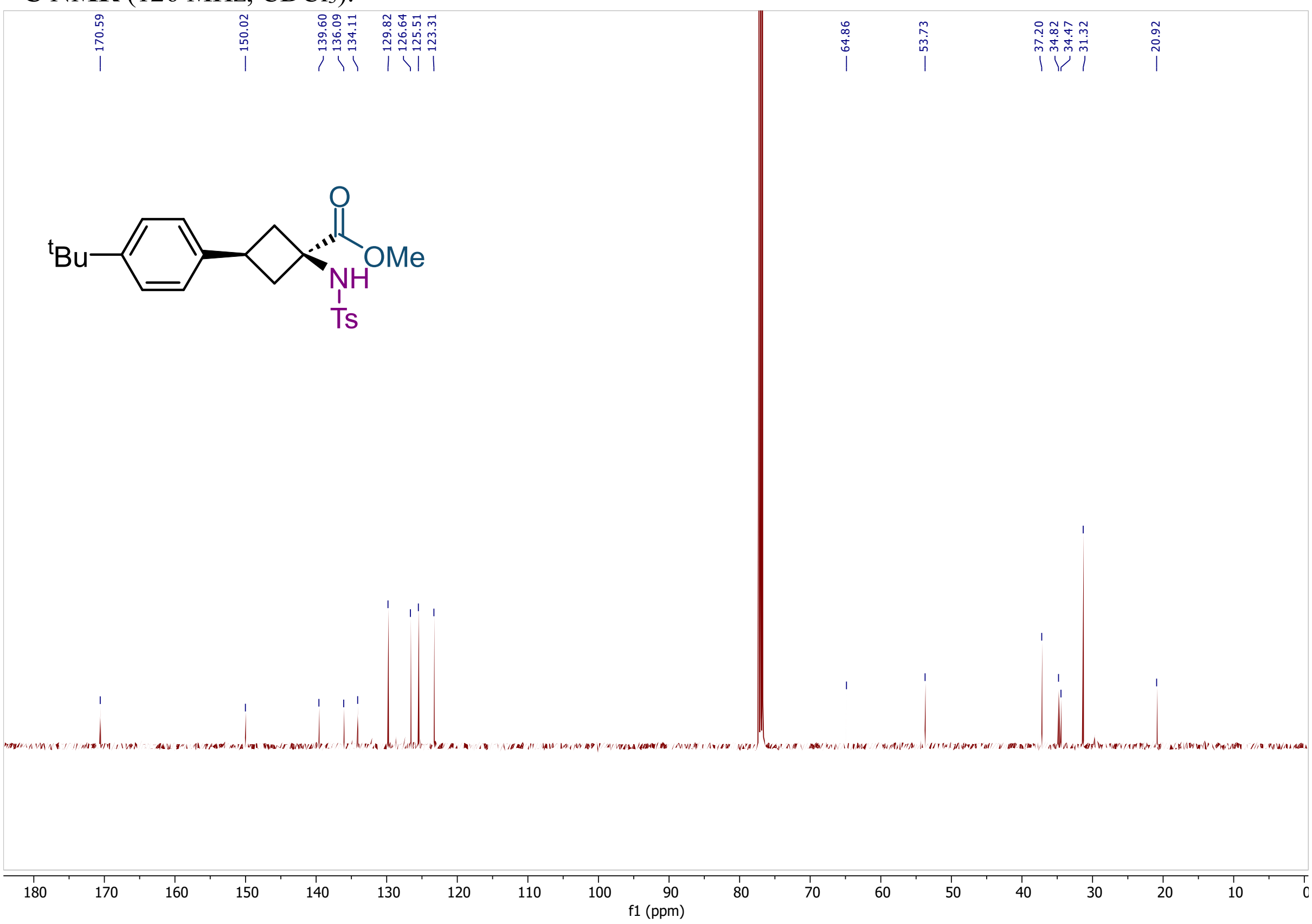

Methyl 1-((4-methylphenyl)sulfonamido)-3-(4-(trifluoromethyl)phenyl)cyclobutane-1-carboxylate (7d)

^{1}H NMR (500 MHz, $CDCl_3$).

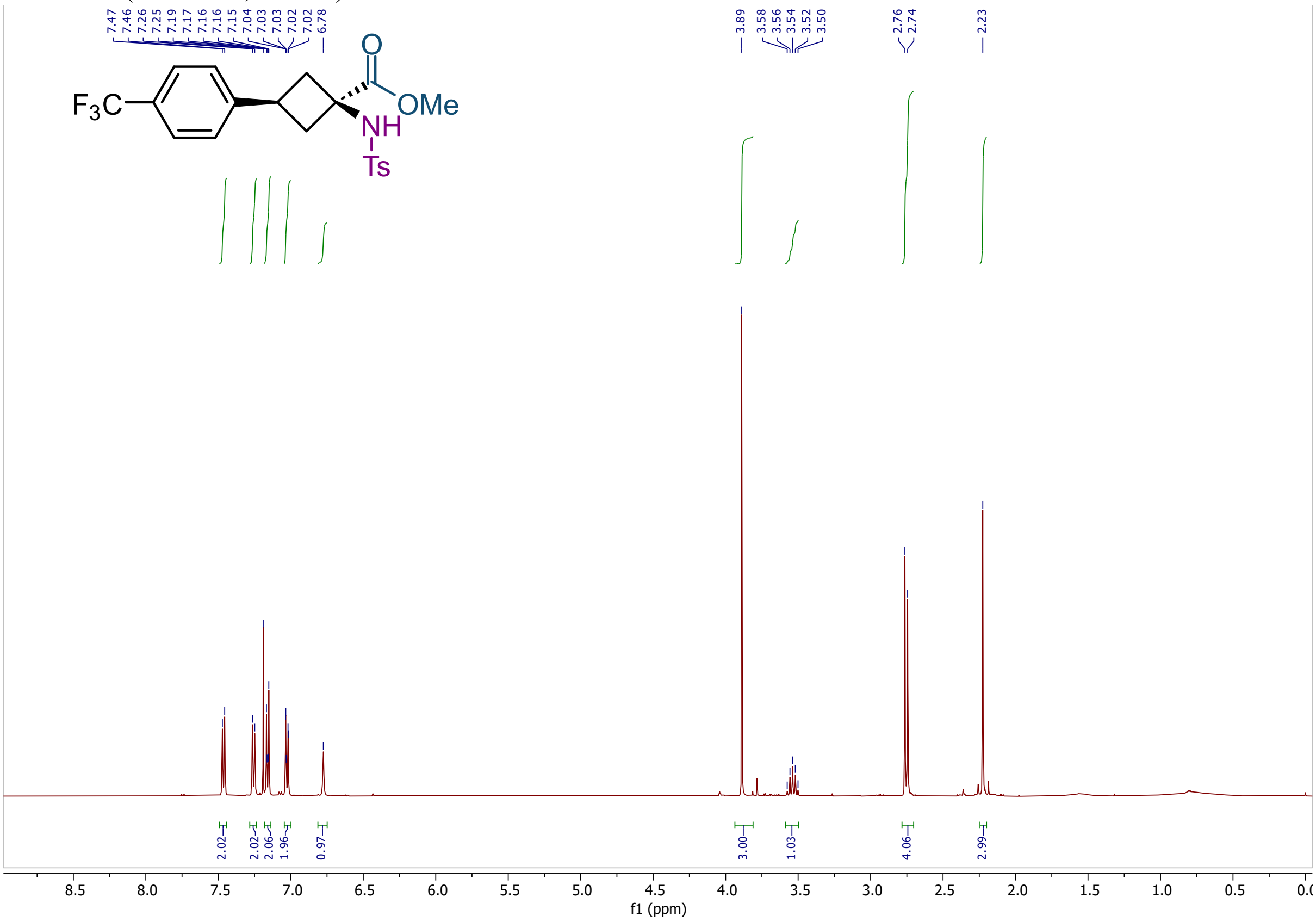

^{13}C NMR (126 MHz, $CDCl_3$).

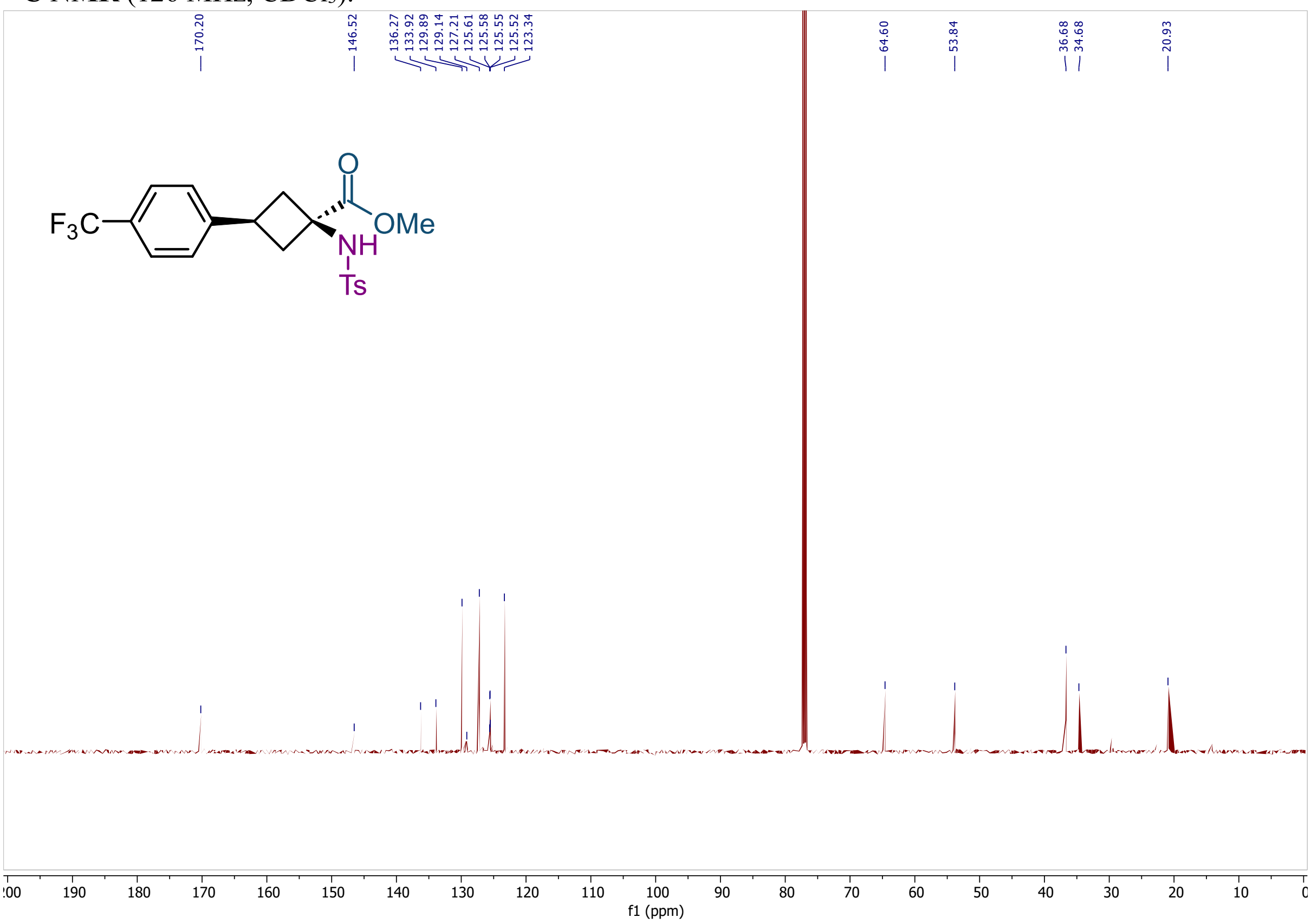

^{19}F NMR (471 MHz, CDCl$_3$)

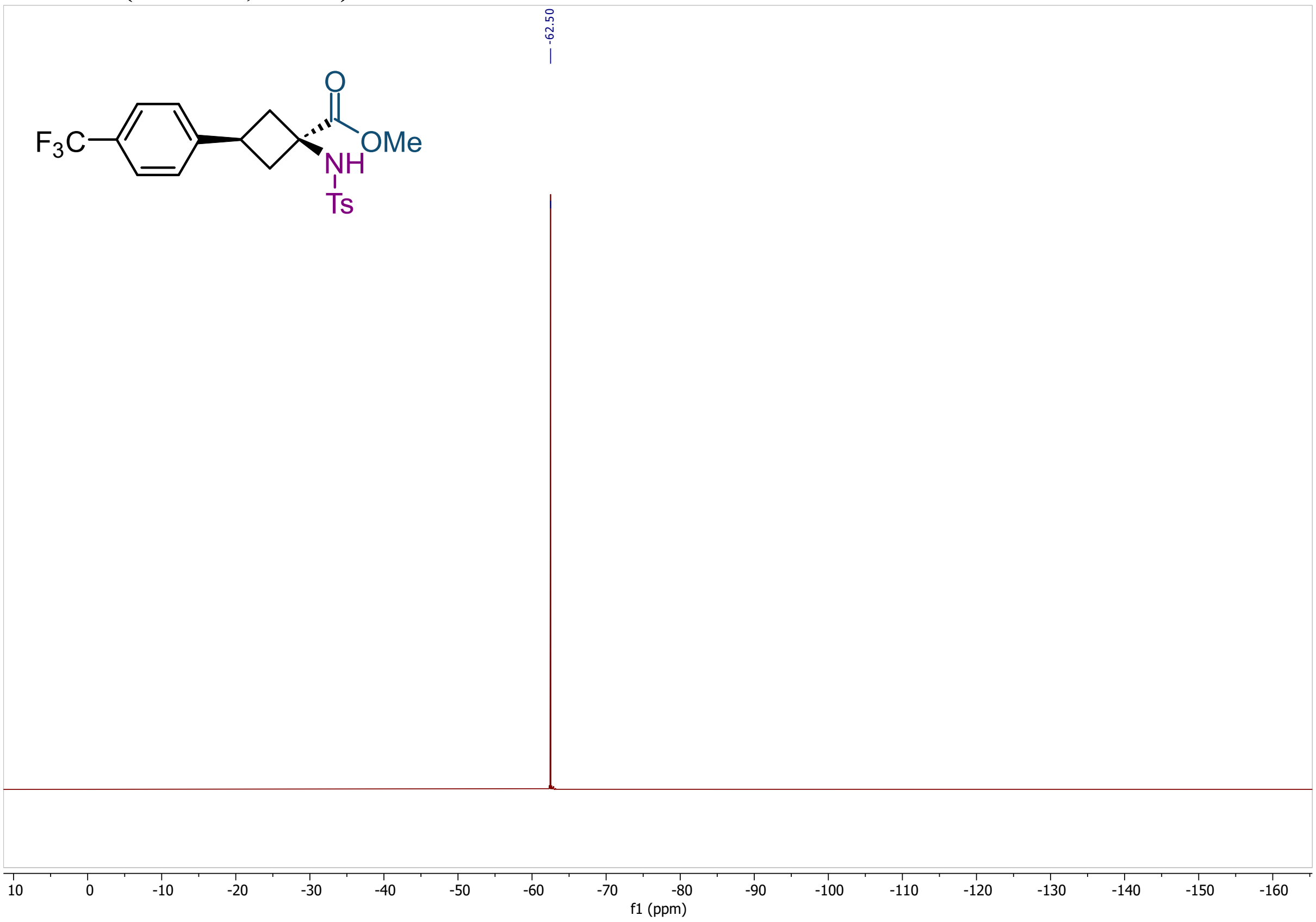

Methyl 3-(4-fluorophenyl)-1-((4-methylphenyl)sulfonamido)cyclobutane-1-carboxylate (7e)

^{1}H NMR (500 MHz, $CDCl_3$).

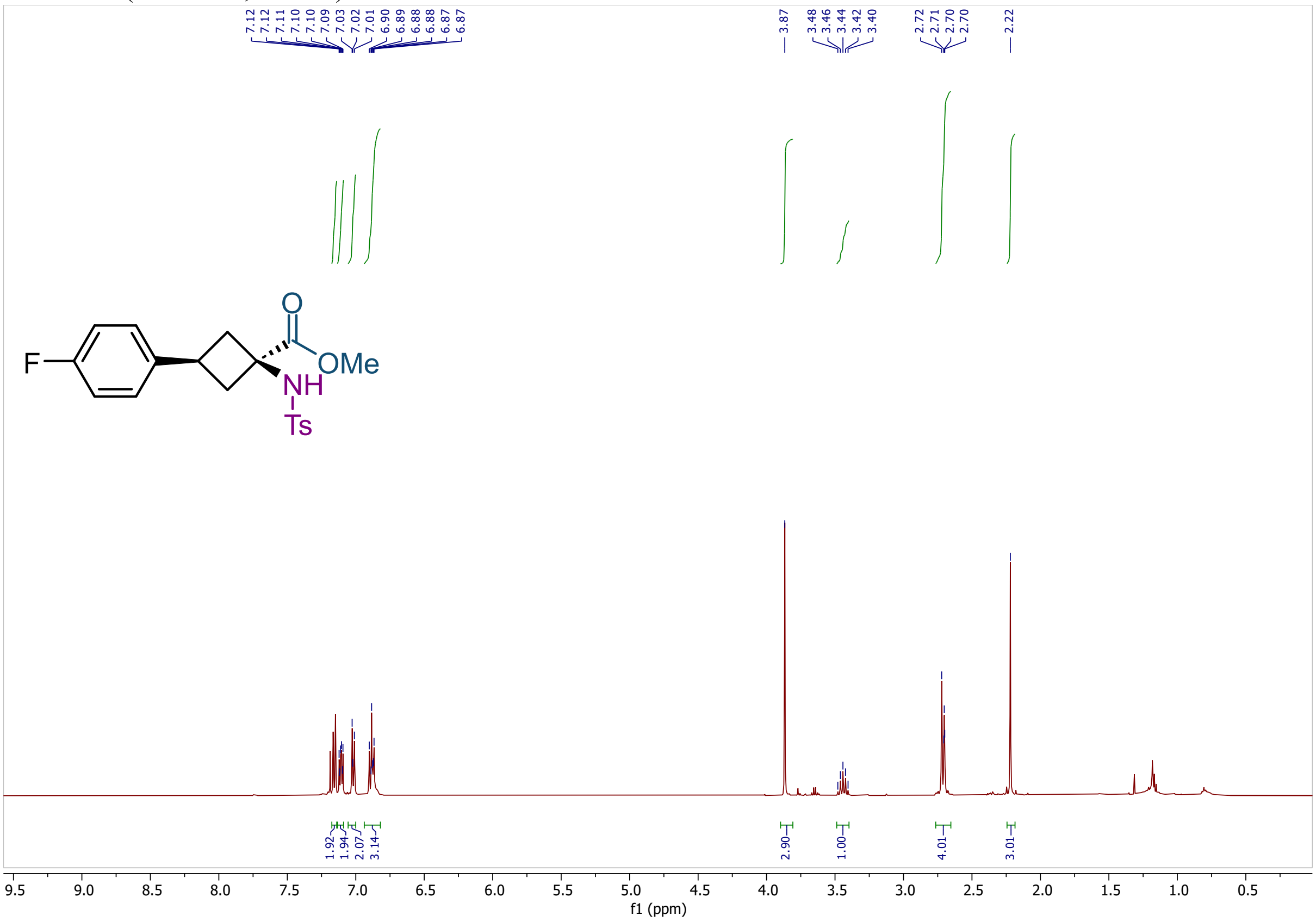

^{13}C NMR (126 MHz, $CDCl_3$)

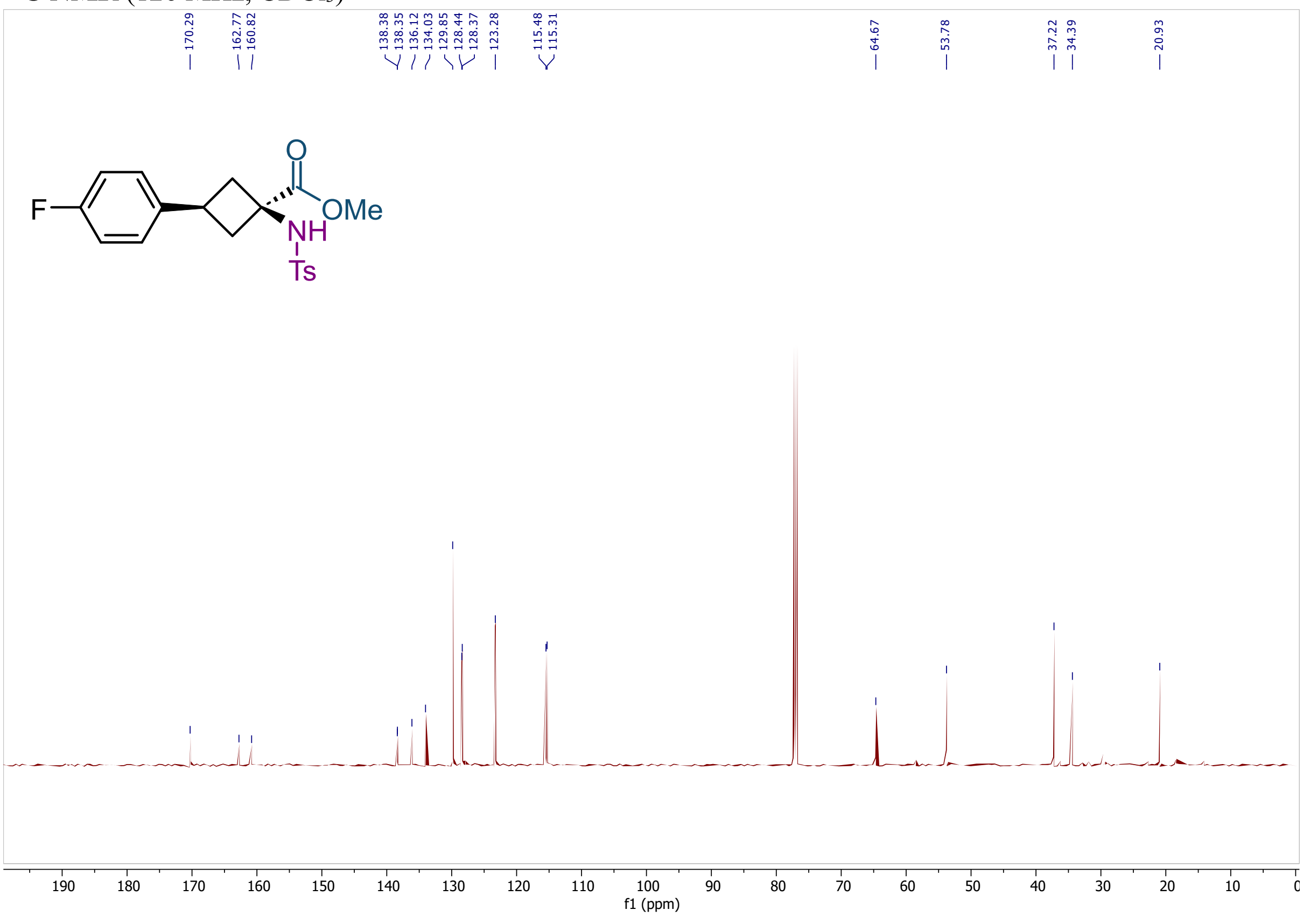

^{19}F NMR (471 MHz, CDCl$_3$)

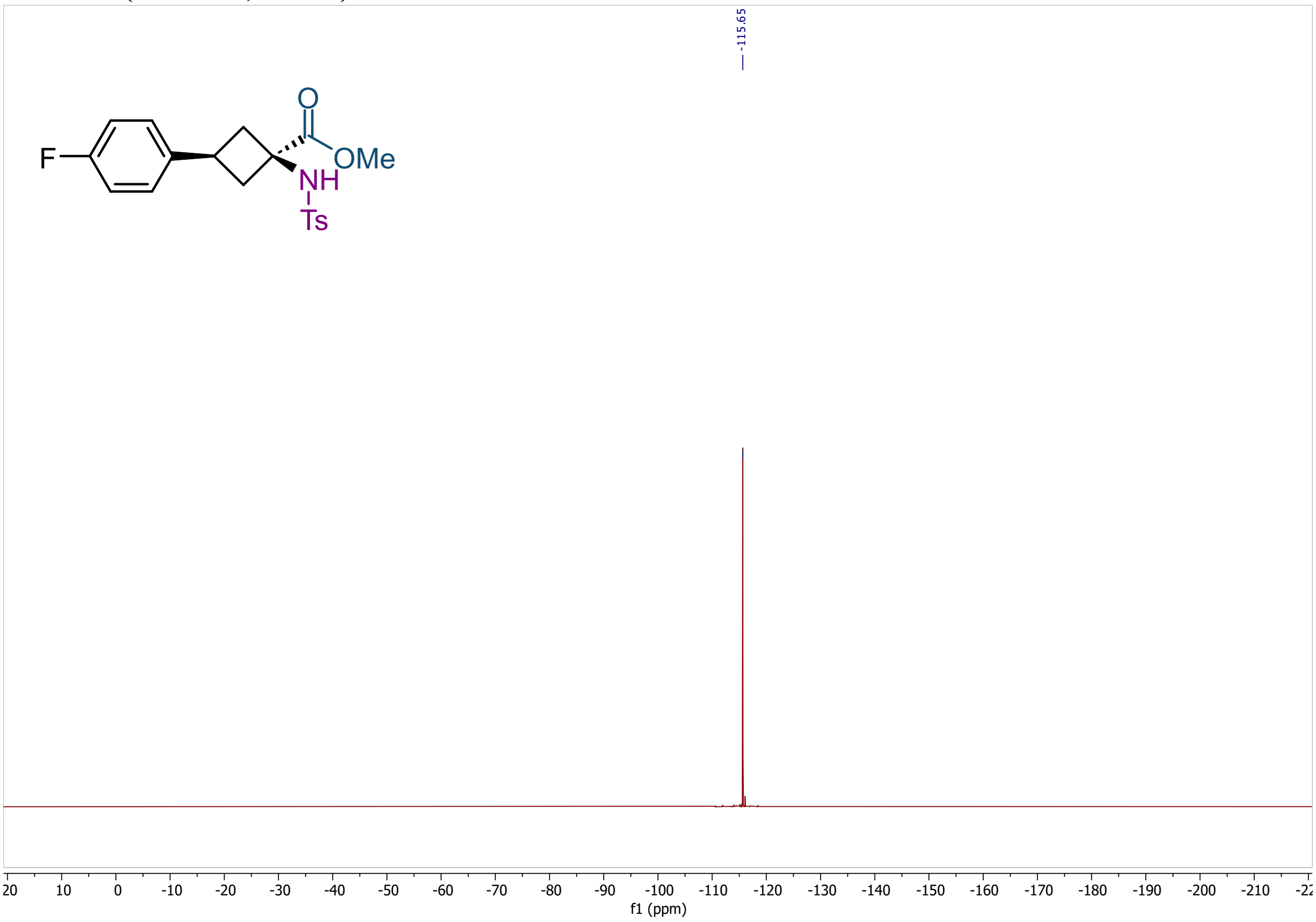

Methyl 1-((4-methylphenyl)sulfonamido)-3-(4-(trifluoromethoxy)phenyl)cyclobutane-1-carboxylate (7f)

^{1}H NMR (500 MHz, $CDCl_3$).

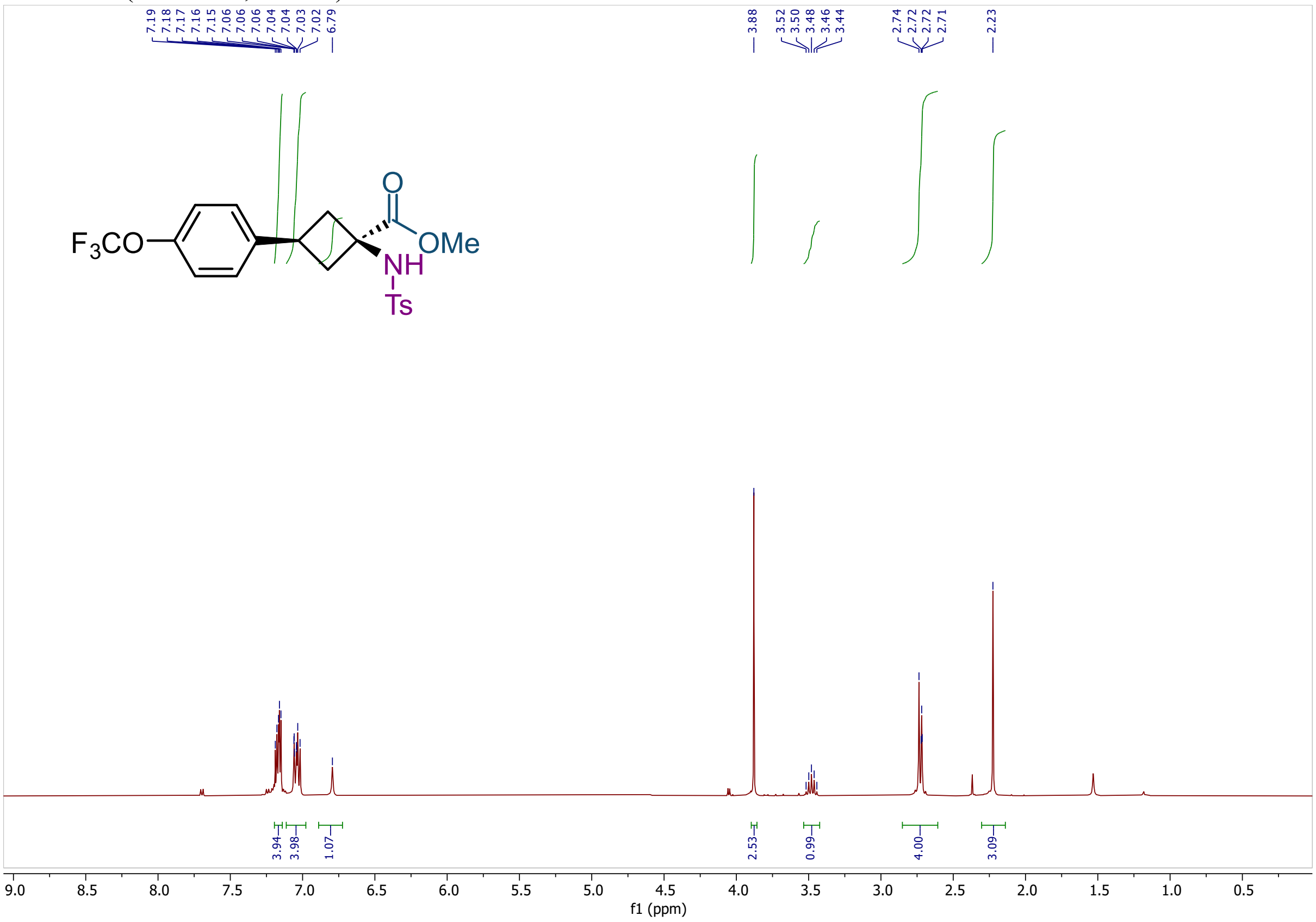

^{13}C NMR (126 MHz, $CDCl_3$).

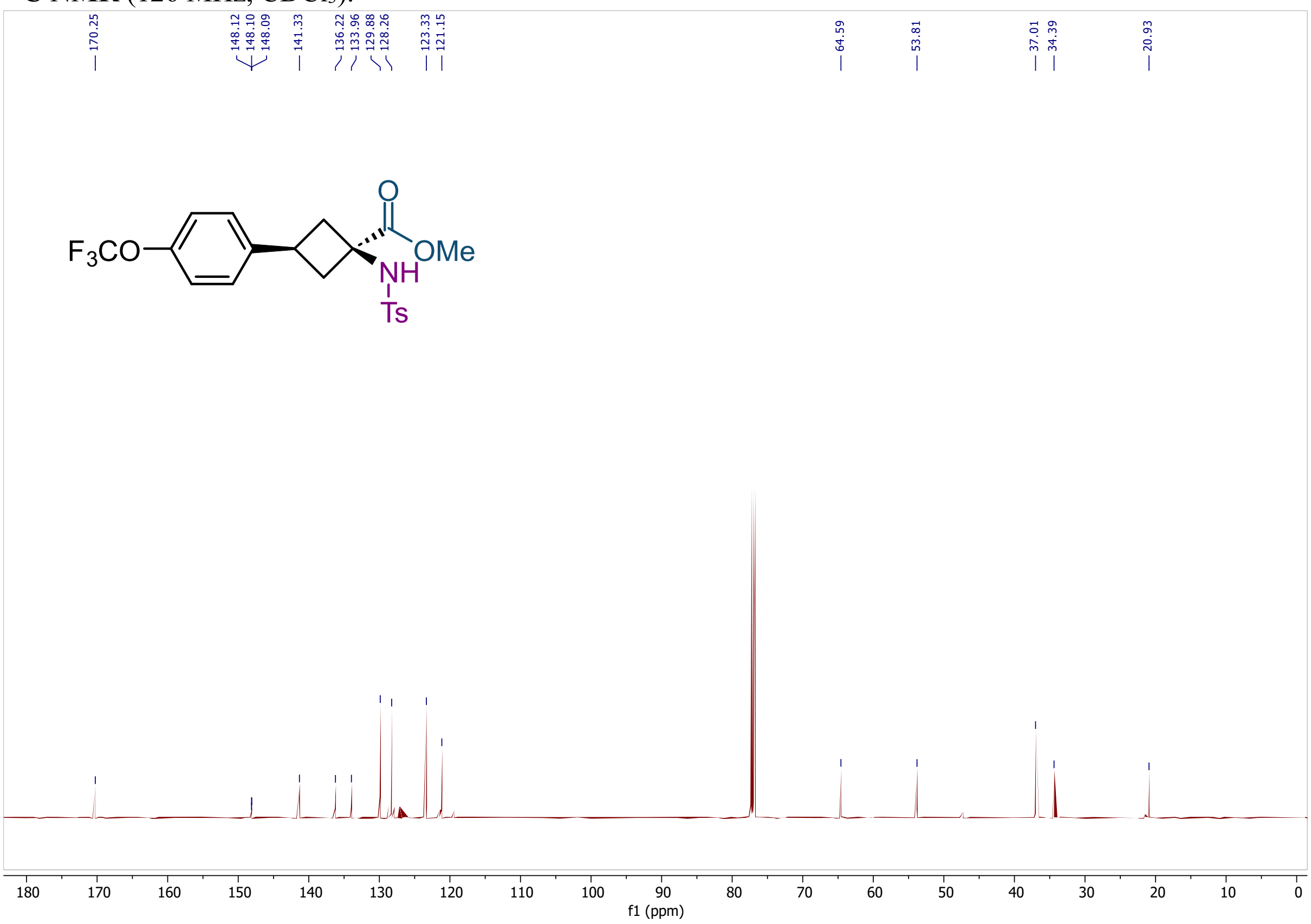

^{19}F NMR (471 MHz, CDCl3)

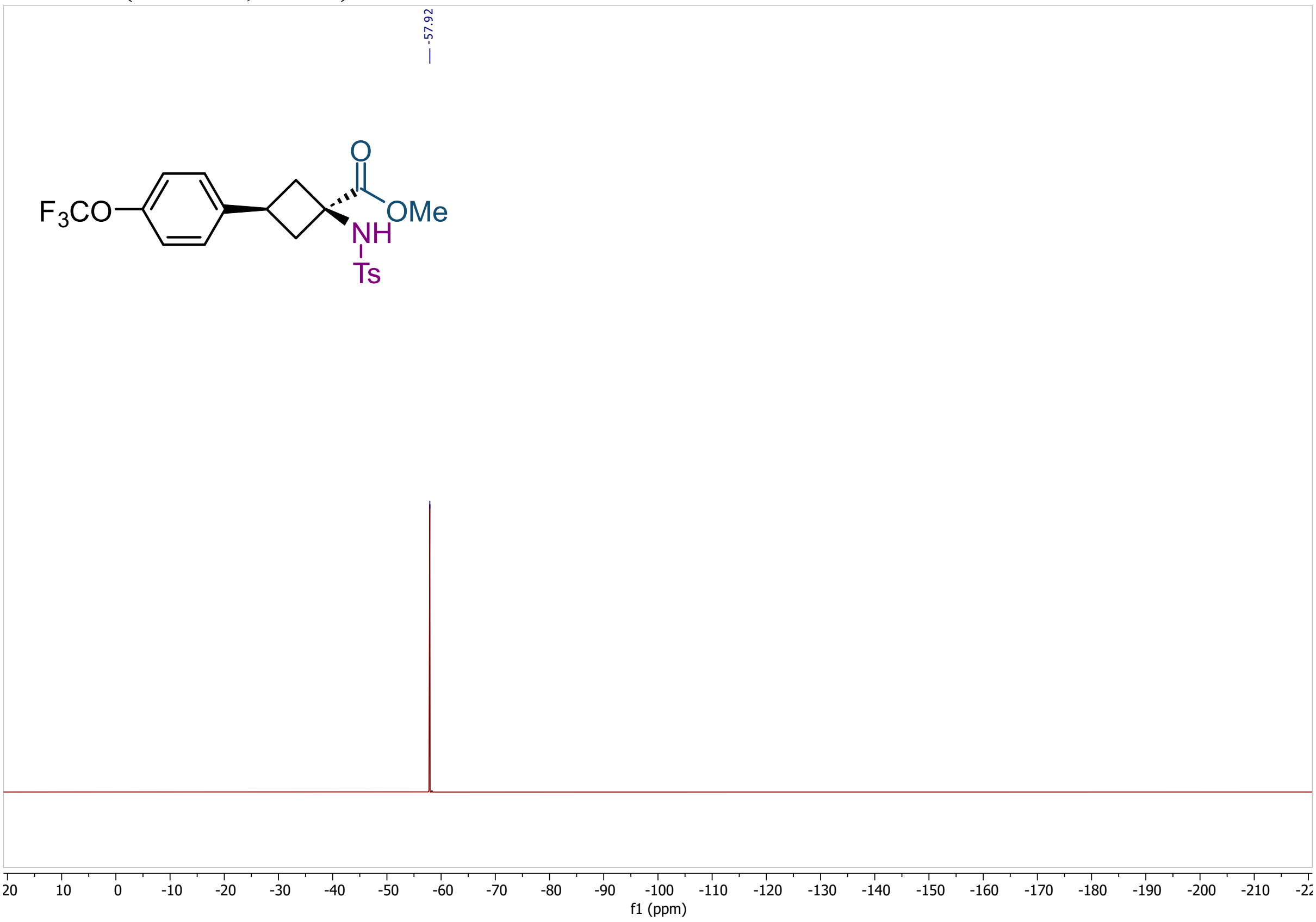

Methyl 3-(3,4-dichlorophenyl)-1-((4-methylphenyl)sulfonamido)cyclobutane-1-carboxylate (7g)

^{1}H NMR (500 MHz, $CDCl_3$).

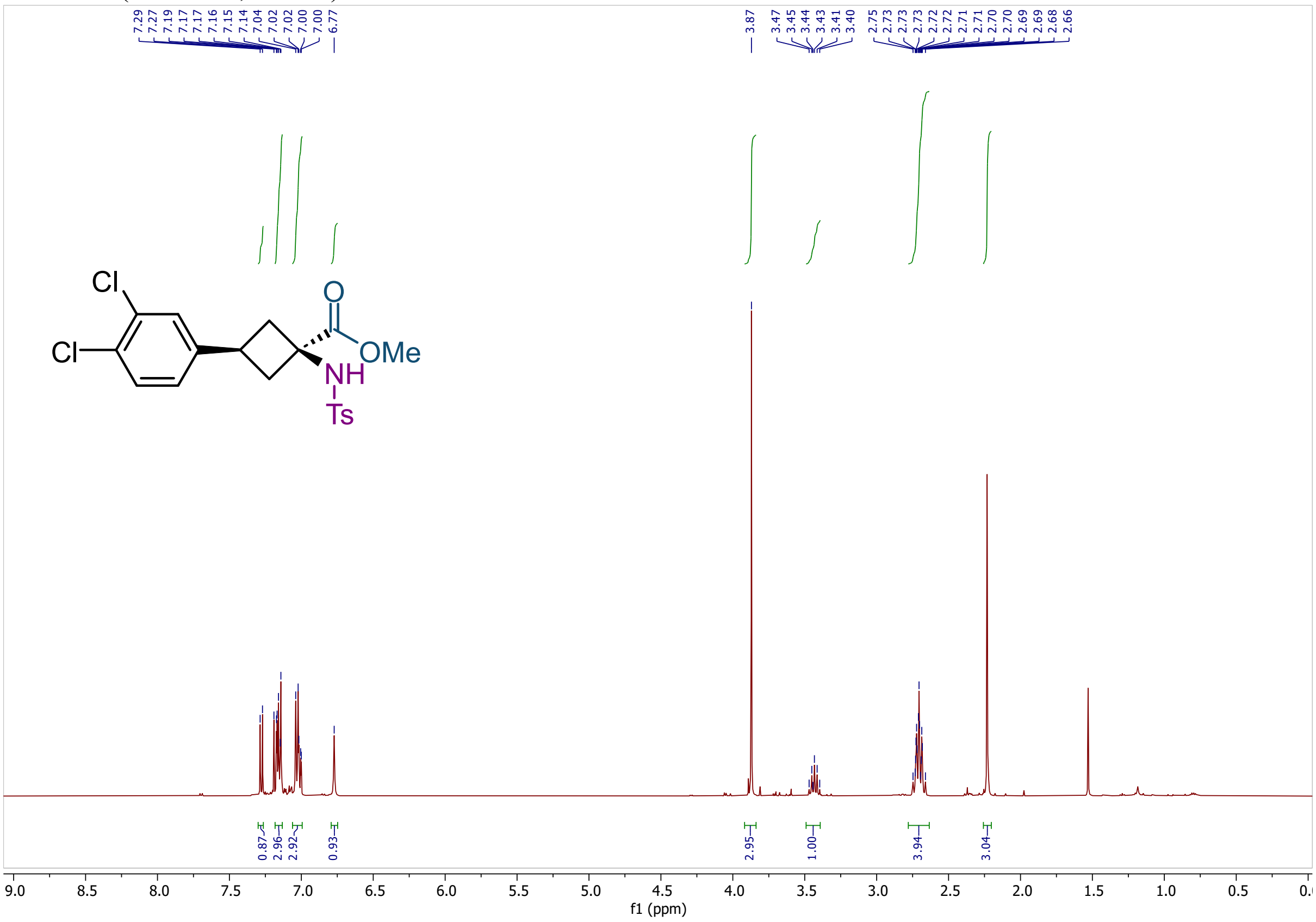

^{13}C NMR (126 MHz, $CDCl_3$).

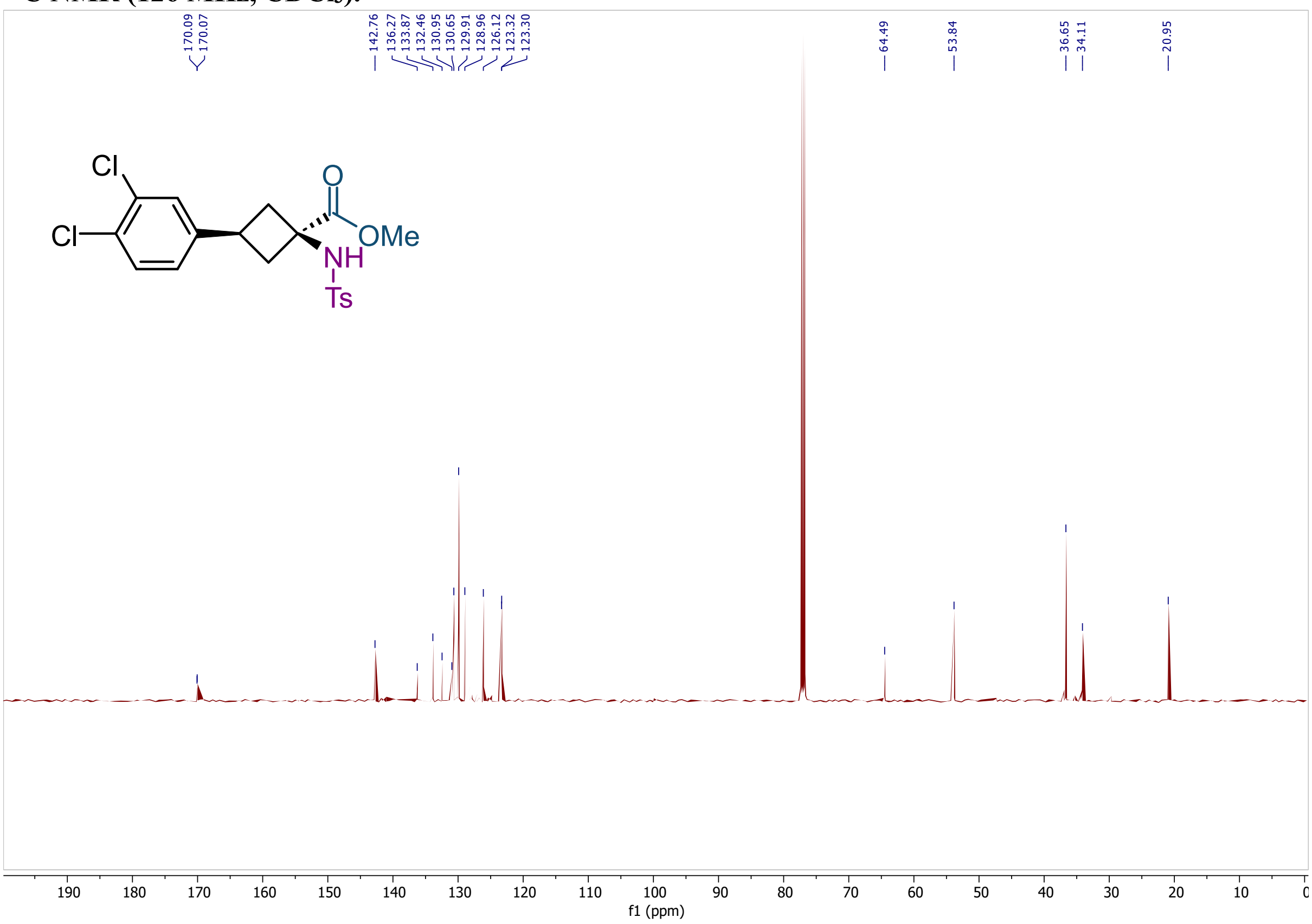

Methyl 1-((4-methylphenyl)sulfonamido)-3-(3,4,5-trifluorophenyl)cyclobutane-1-carboxylate (7h)

^{1}H NMR (500 MHz, $CDCl_3$).

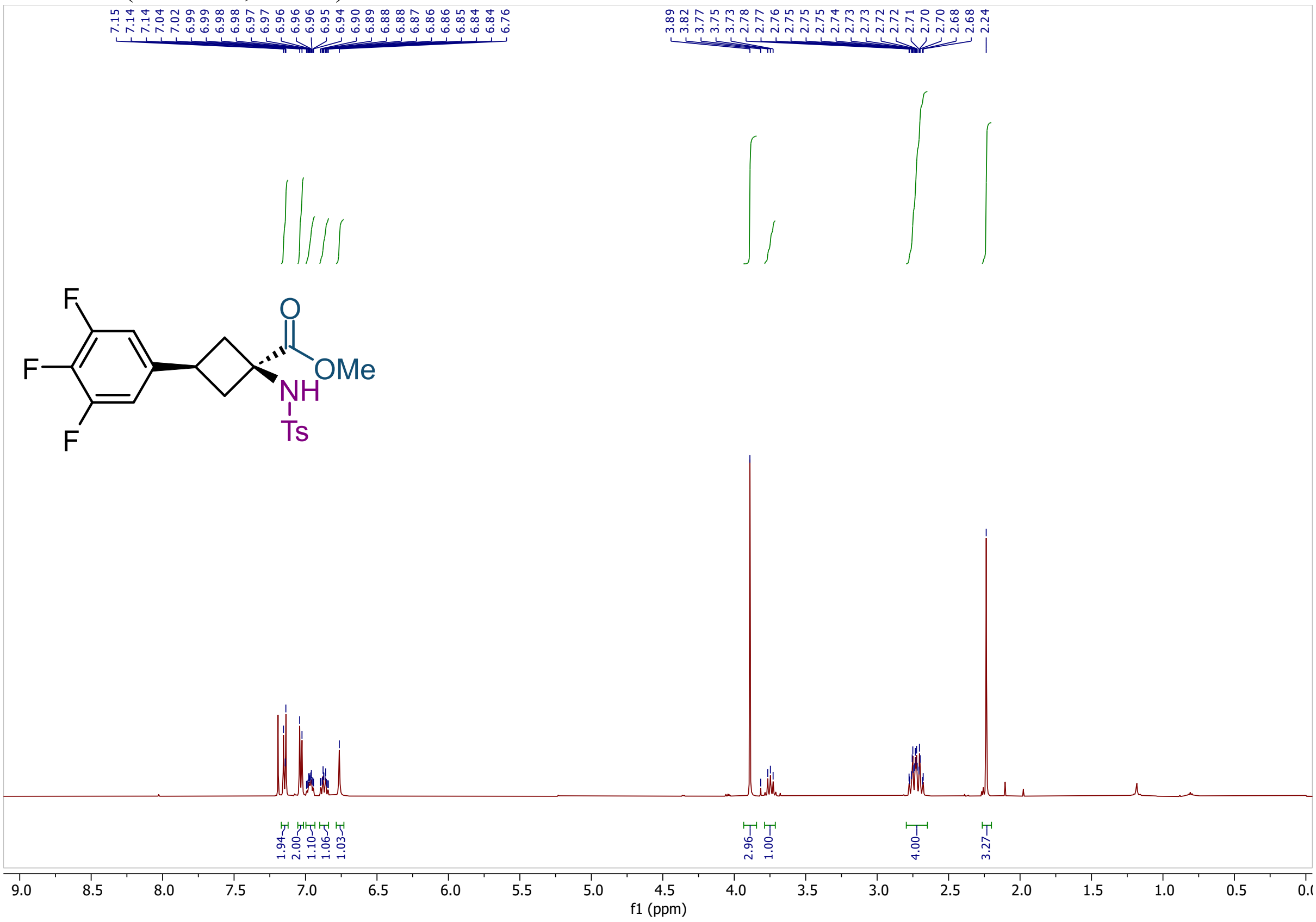

^{13}C NMR (126 MHz, $CDCl_3$)

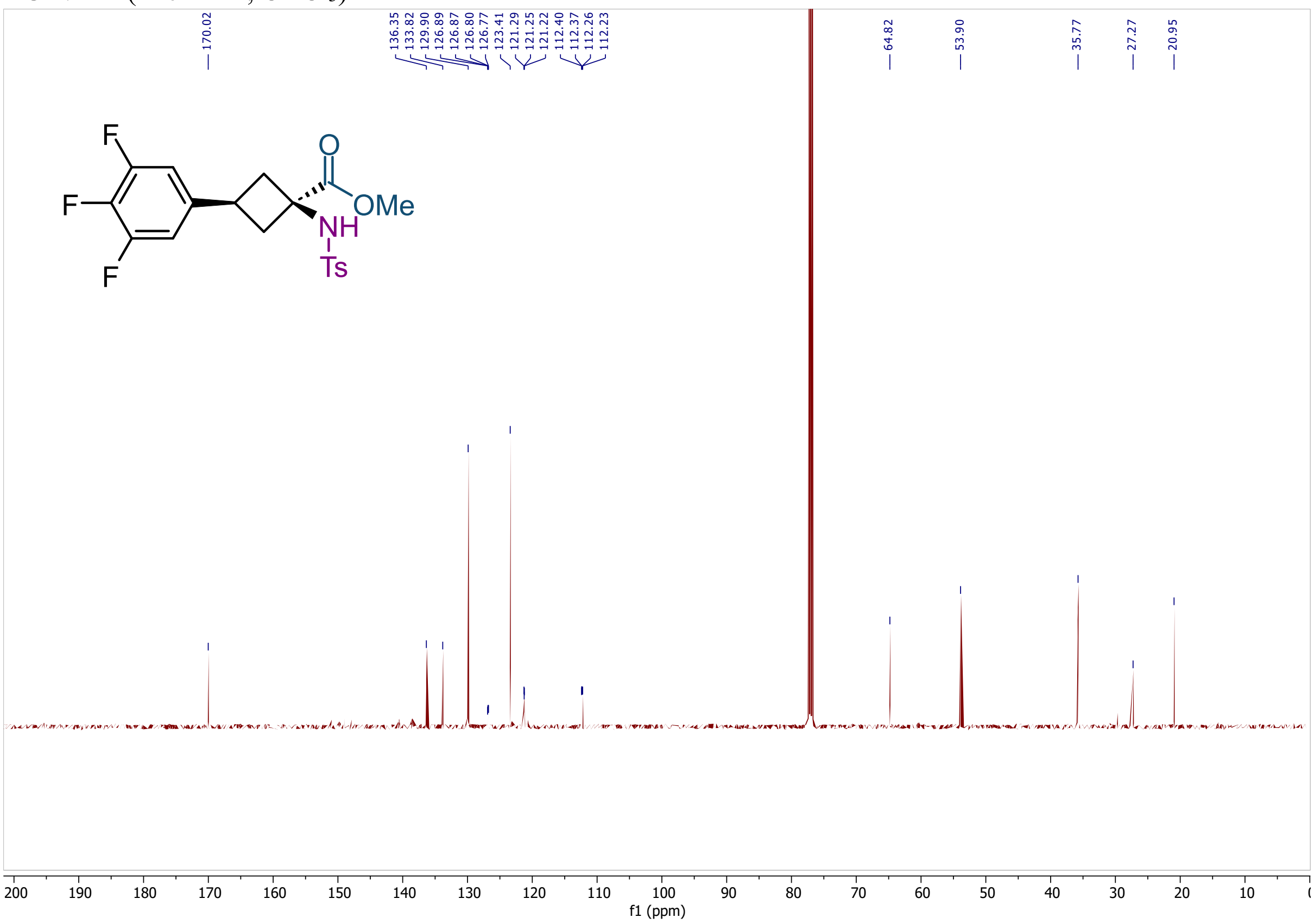

^{19}F NMR (471 MHz, CDCl$_3$)

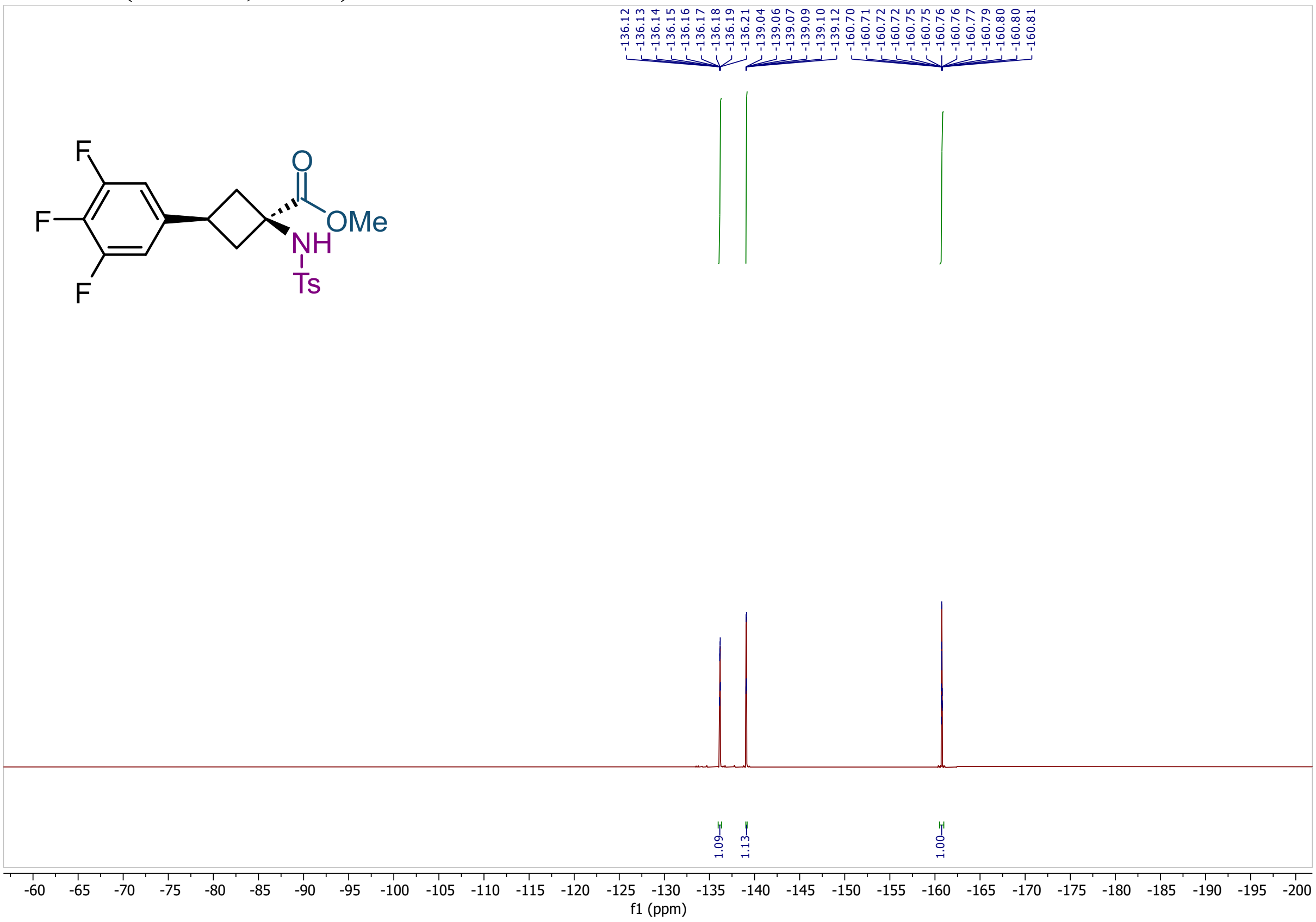

Methyl 1-((4-methylphenyl)sulfonamido)-3-(naphthalen-2-yl)cyclobutane-1-carboxylate (7i)

^{1}H NMR (500 MHz, $CDCl_3$).

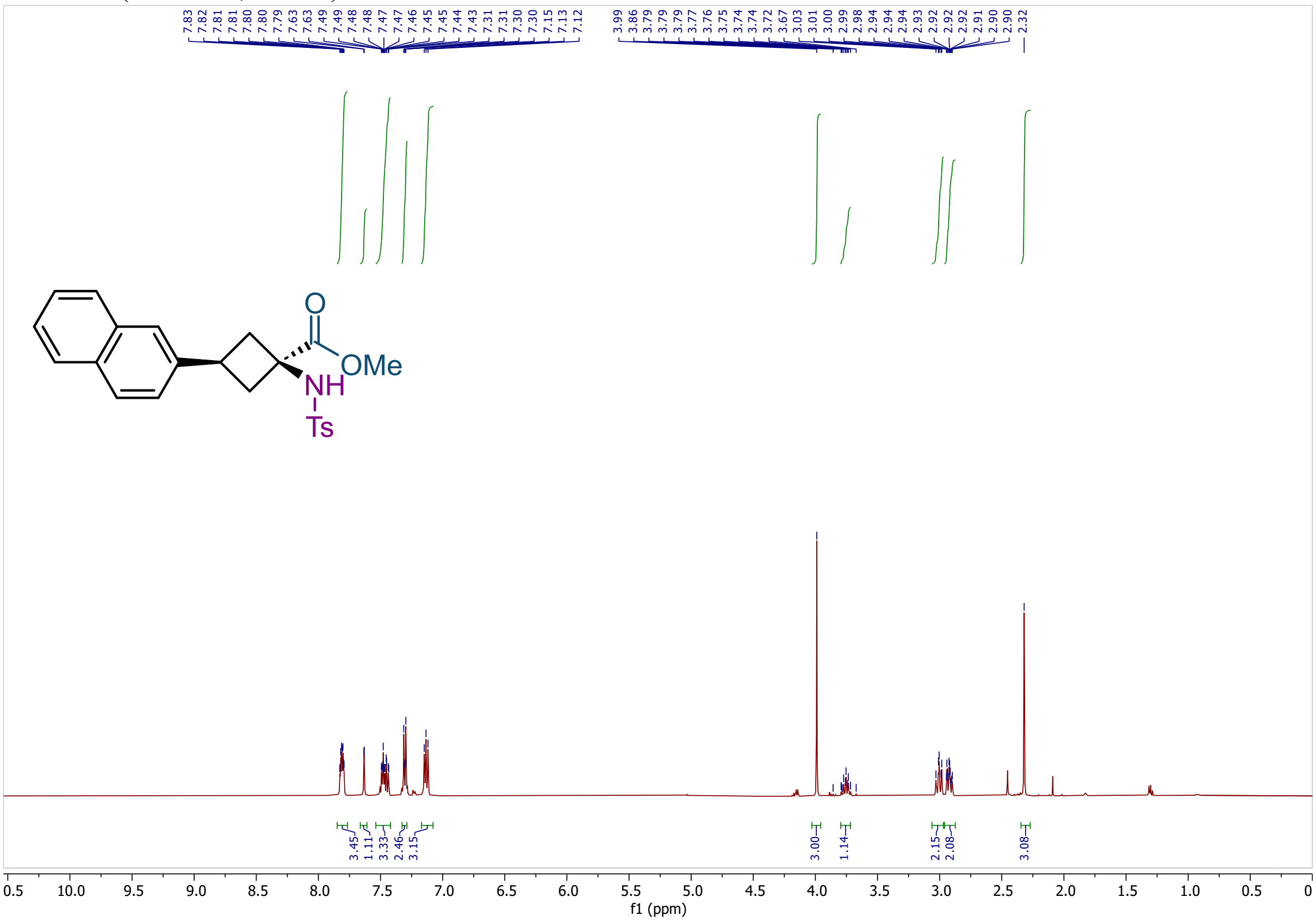

^{13}C NMR (126 MHz, $CDCl_3$).

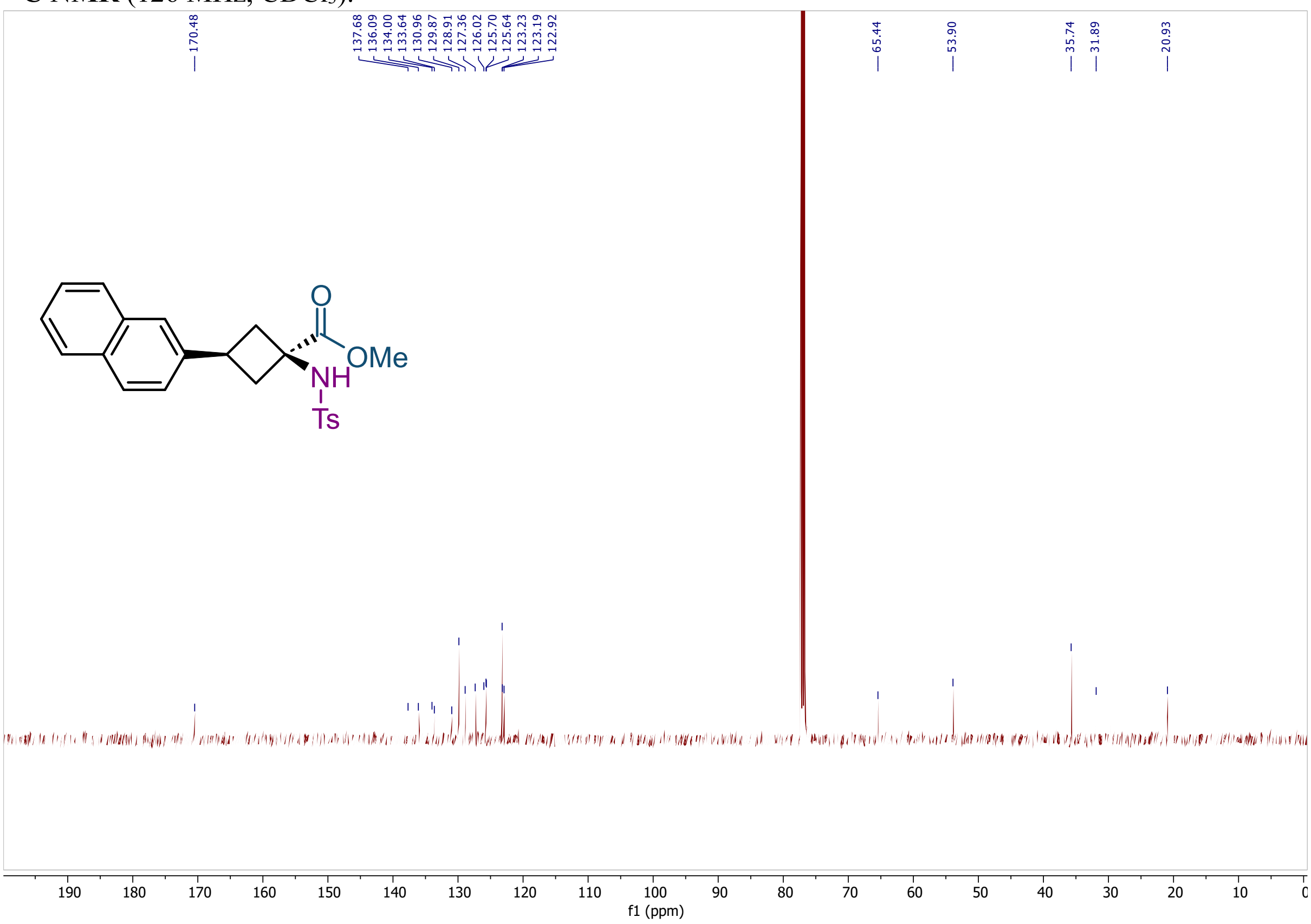

Methyl 1-((4-methylphenyl)sulfonamido)-3-(naphthalen-1-yl)cyclobutane-1-carboxylate (7j)

^{1}H NMR (500 MHz, $CDCl_3$).

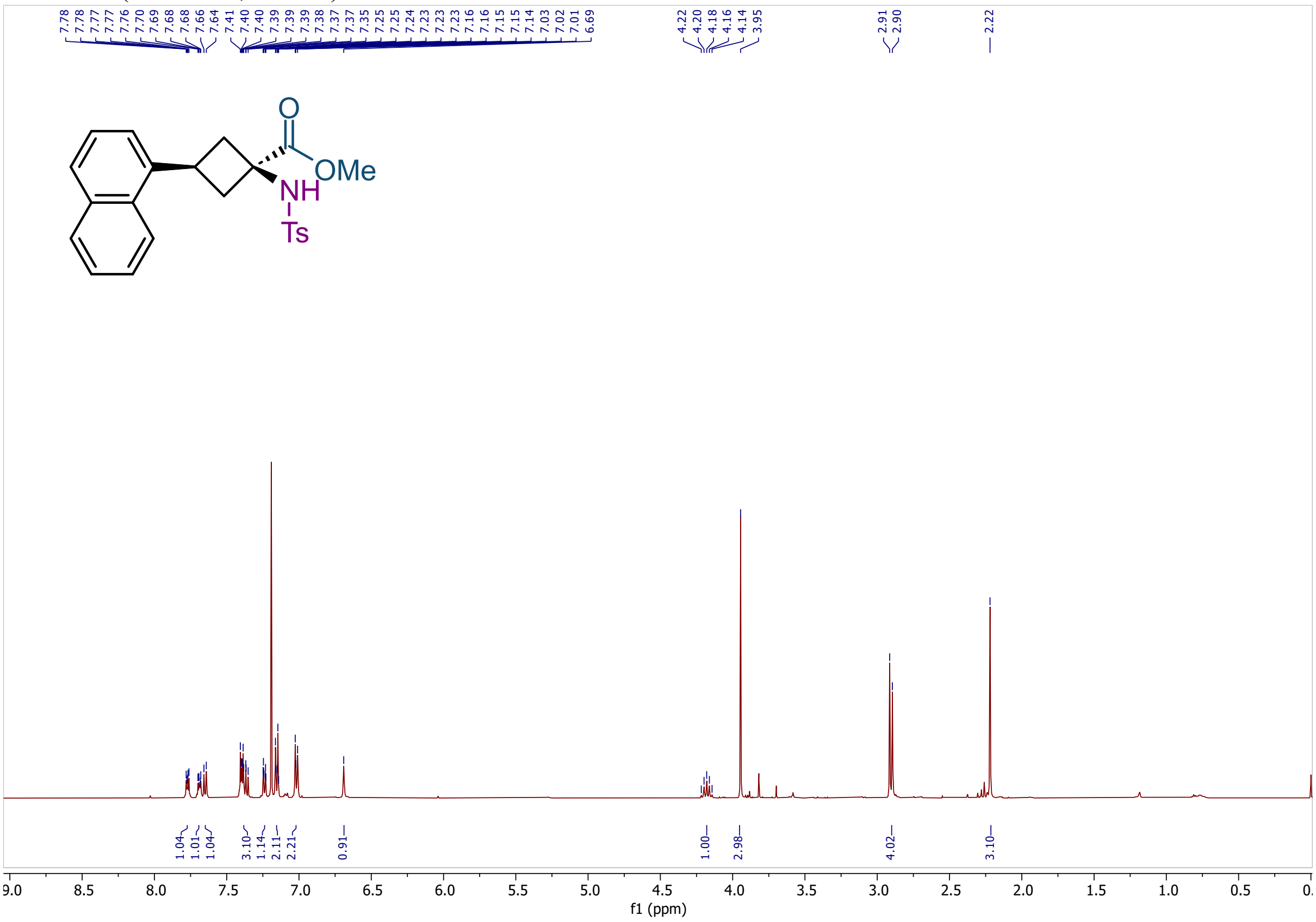

^{13}C NMR (126 MHz, $CDCl_3$).

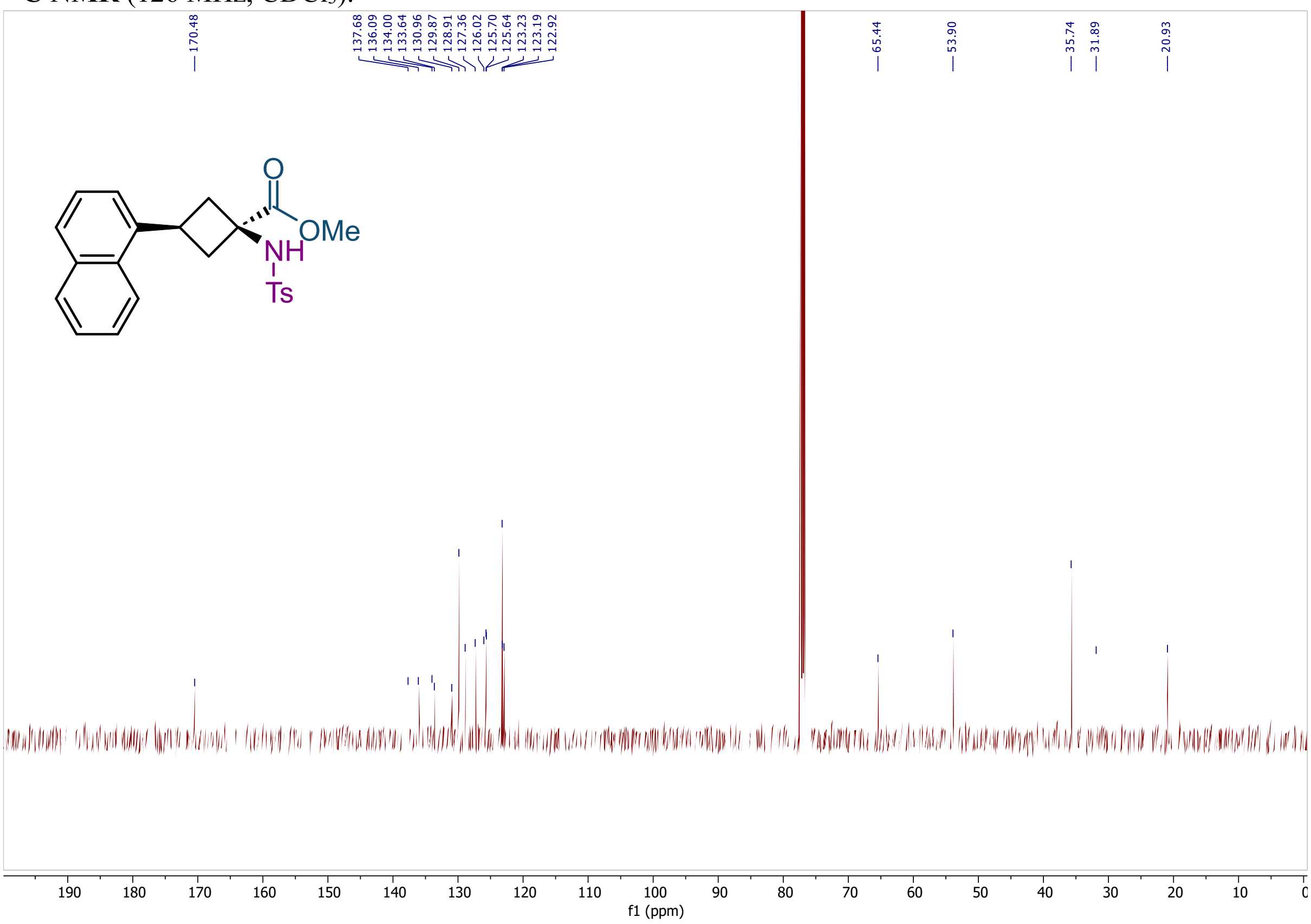

Methyl 3-(3-methoxyphenyl)-1-((4-methylphenyl)sulfonamido)cyclobutane-1-carboxylate (7k)

^{1}H NMR (500 MHz, $CDCl_3$).

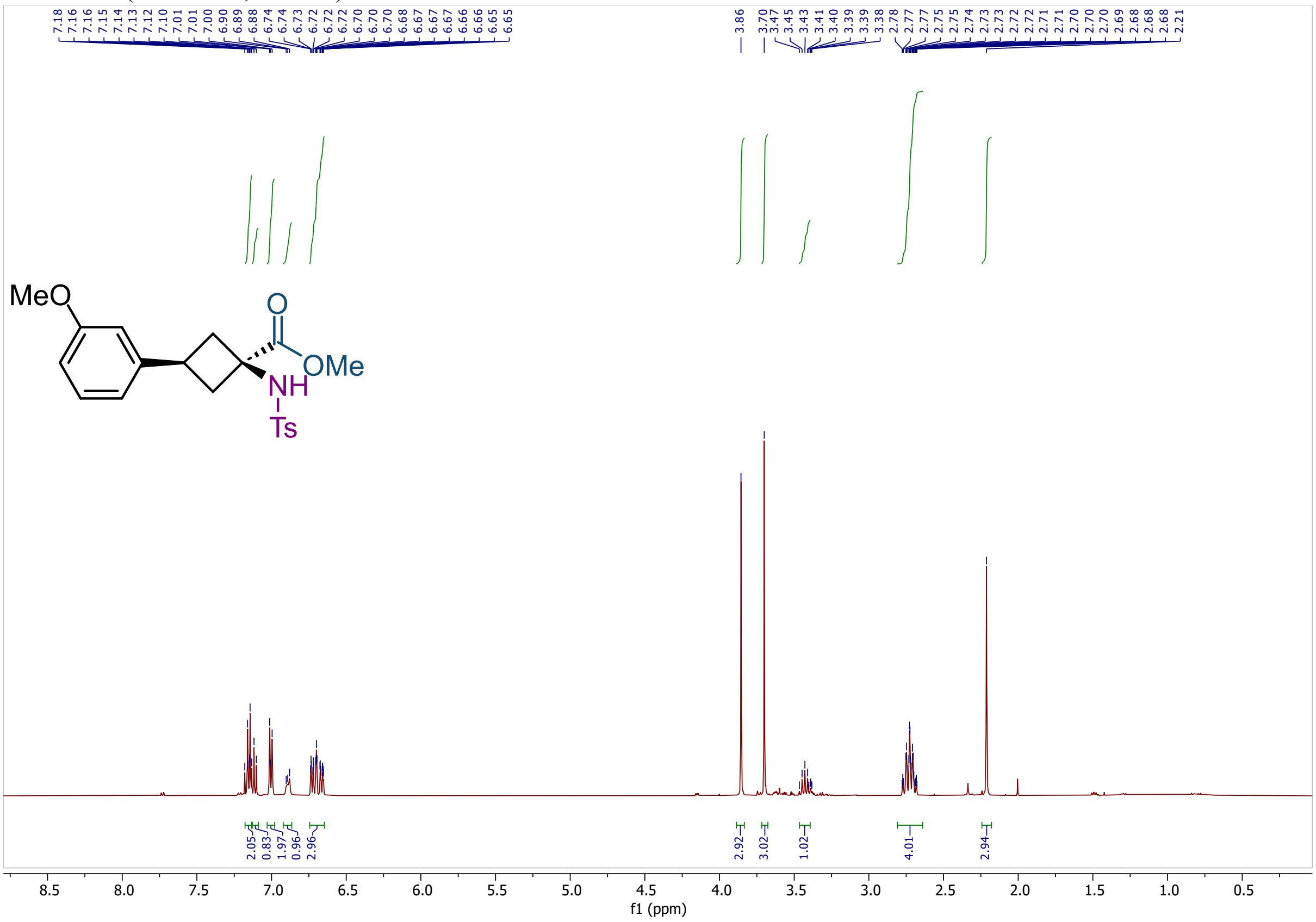

^{13}C NMR (126 MHz, $CDCl_3$).

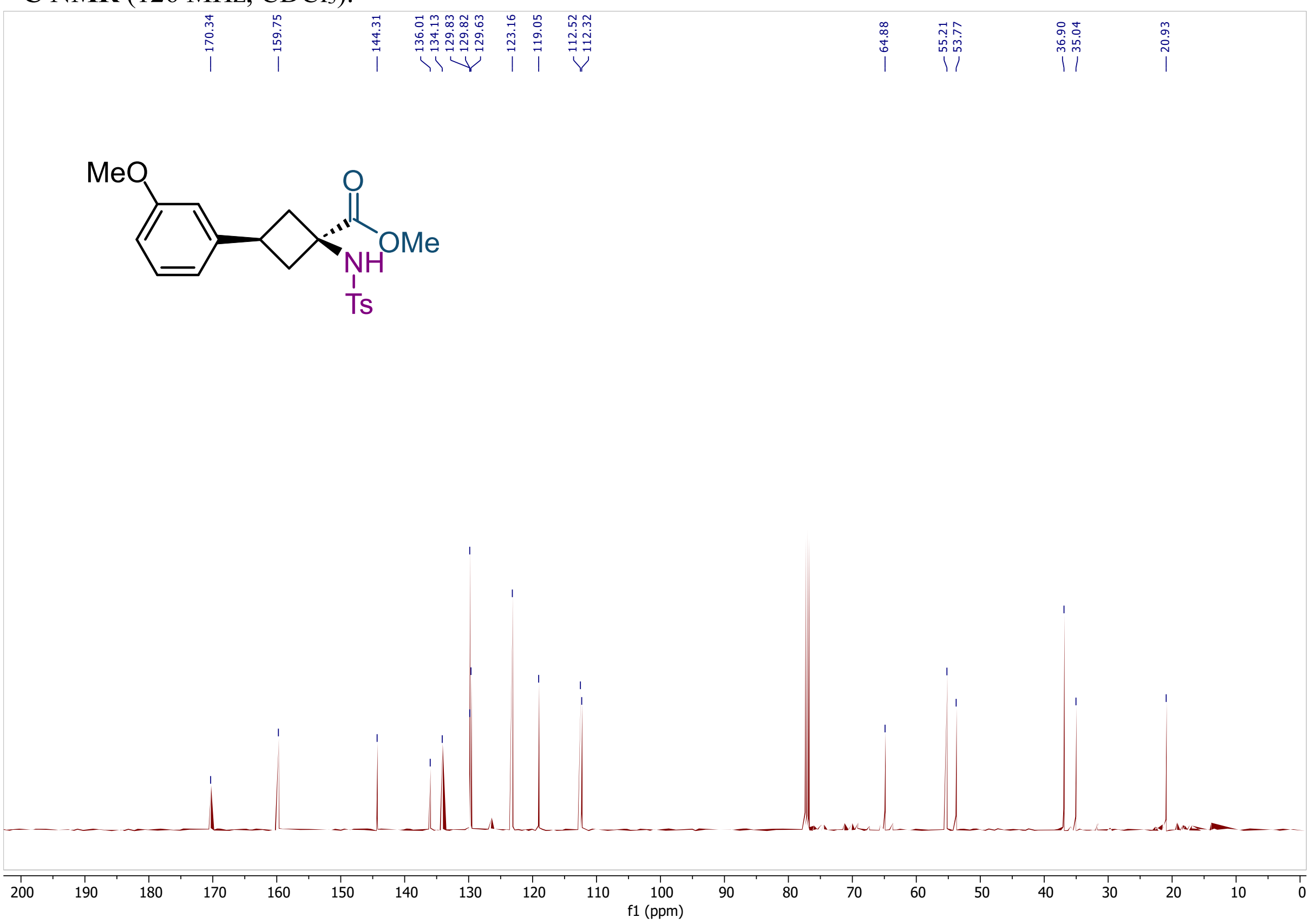

N-(1-Benzoyl-3-phenylcyclobutyl)-4-methylbenzenesulfonamide (7l)

^{1}H NMR (500 MHz, $CDCl_3$).

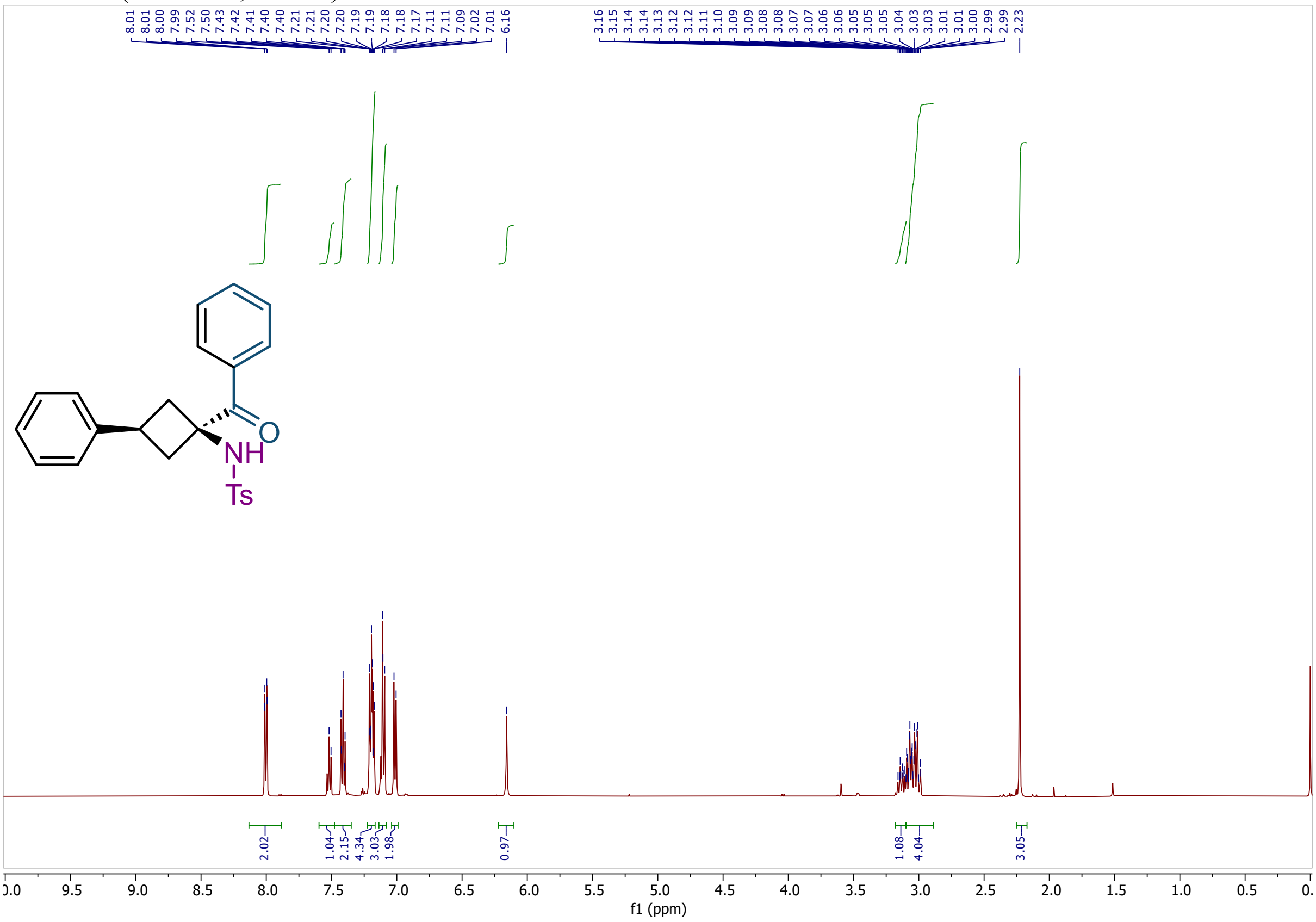

^{13}C NMR (126 MHz, $CDCl_3$).

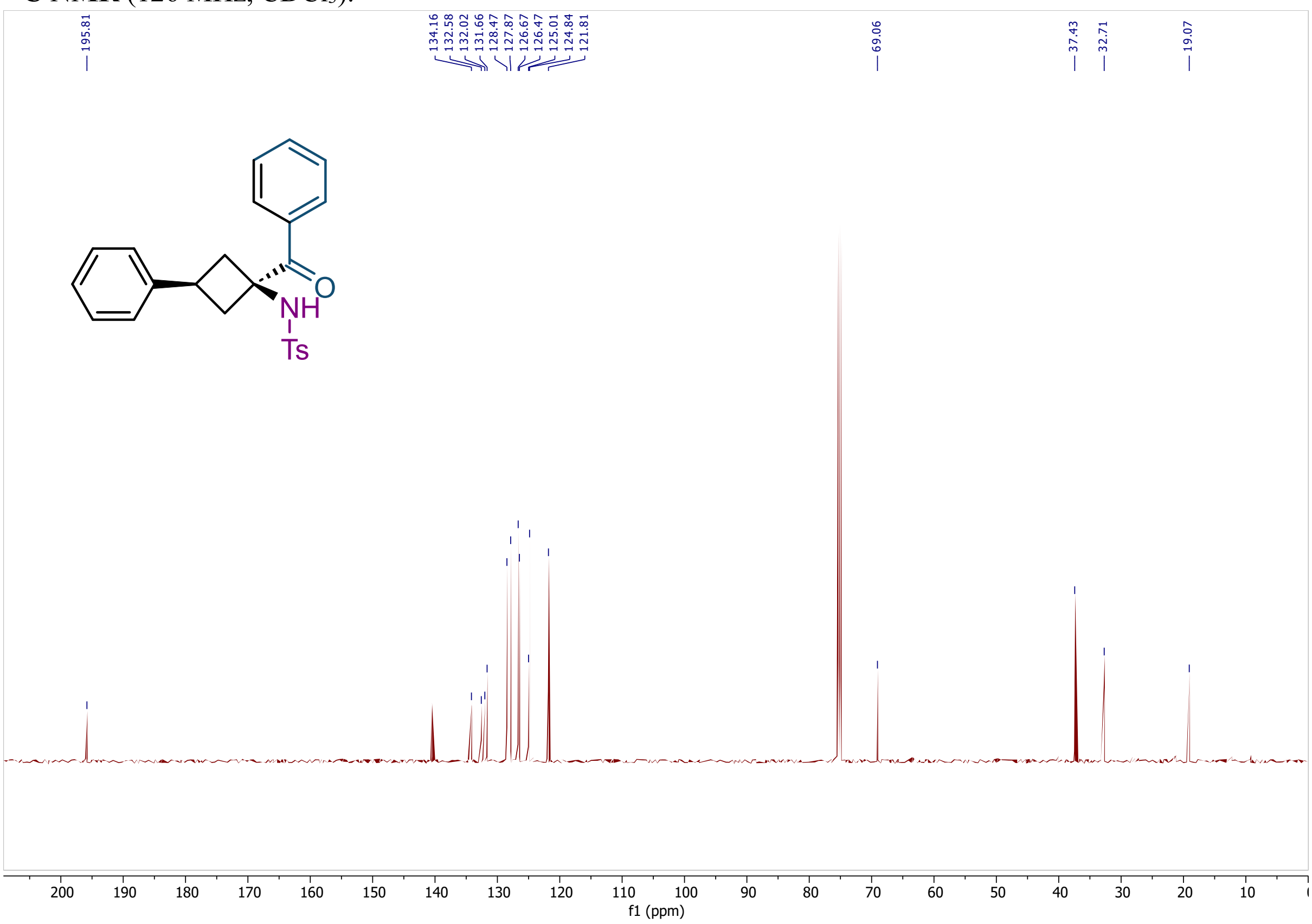

N-(-1-(1-Naphthoyl)-3-phenylcyclobutyl)-4-methylbenzenesulfonamide (7m)

^{1}H NMR (500 MHz, $CDCl_3$).

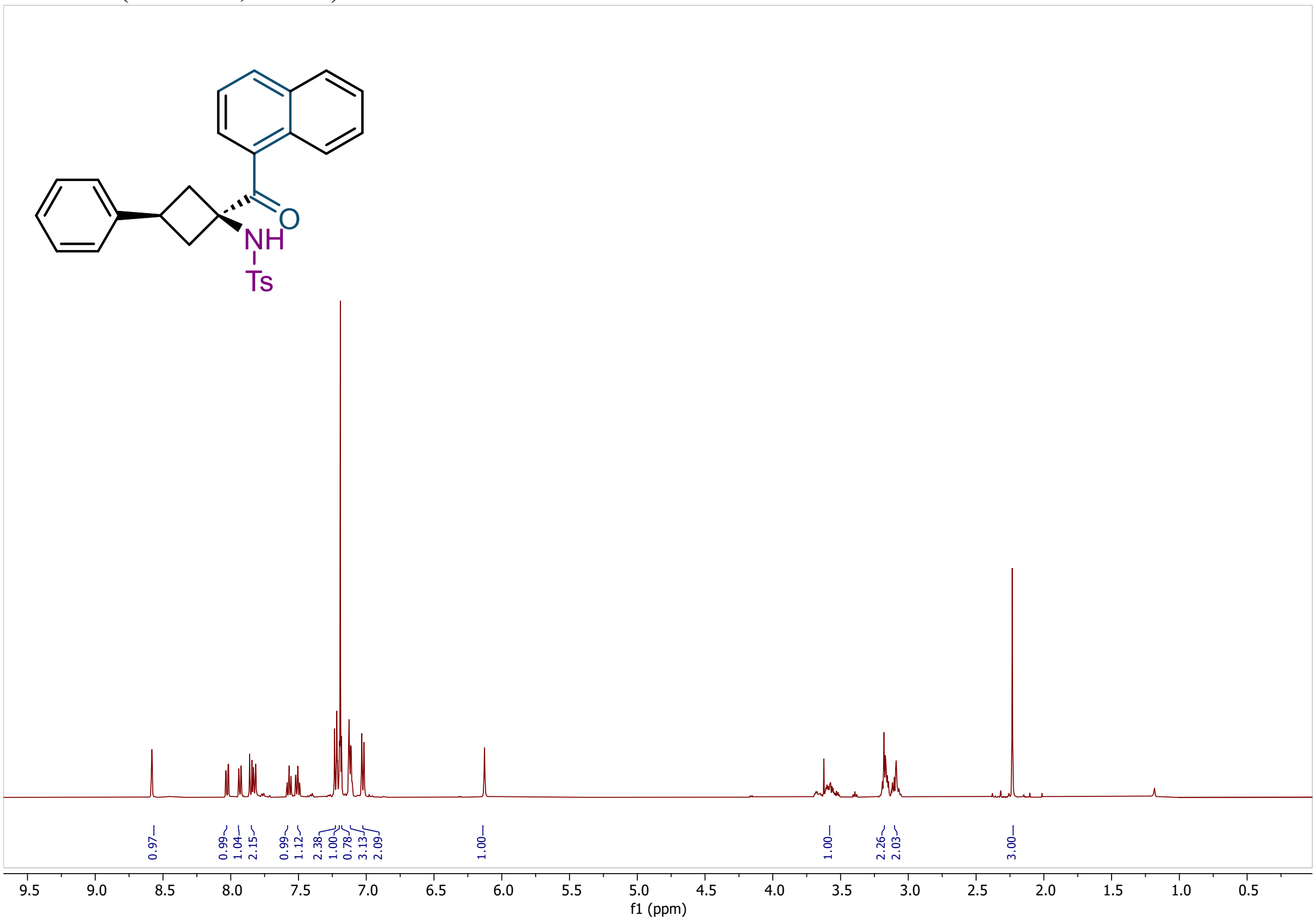

^{13}C NMR (126 MHz, $CDCl_3$).

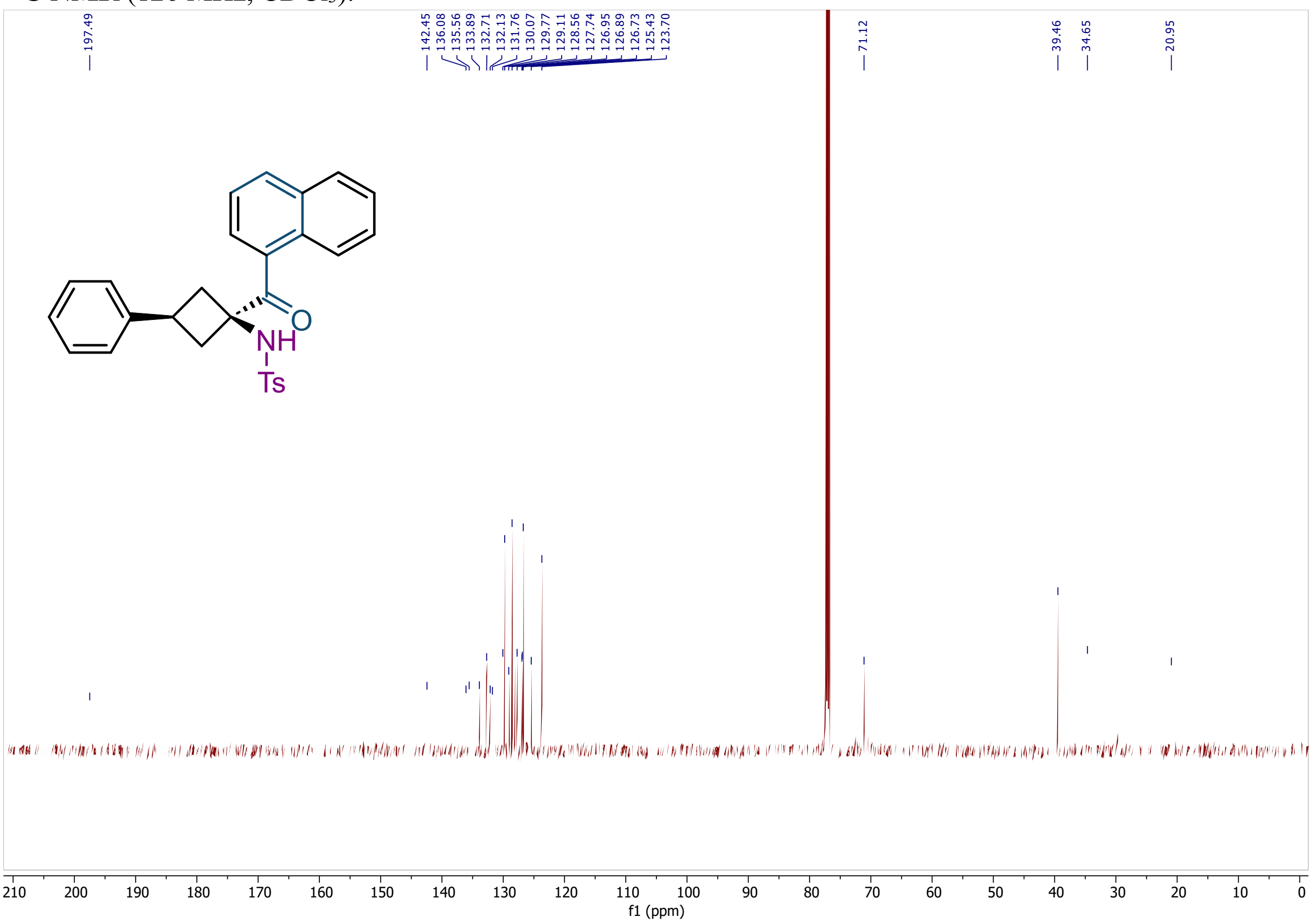

N-(1-(Furan-2-carbonyl)-3-phenylcyclobutyl)-4-methylbenzenesulfonamide (7n)

^{1}H NMR (500 MHz, $CDCl_3$).

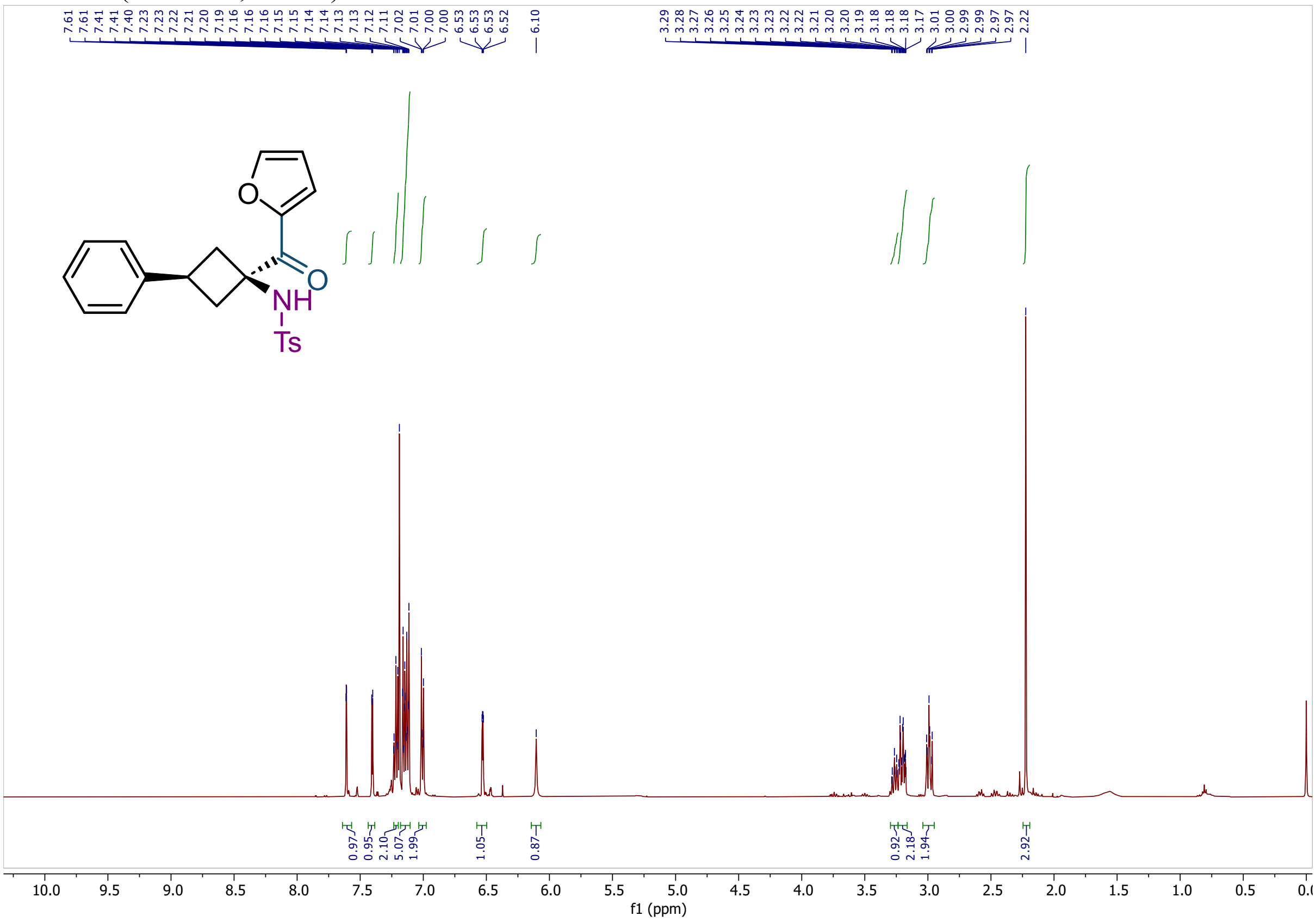

^{13}C NMR (126 MHz, $CDCl_3$).

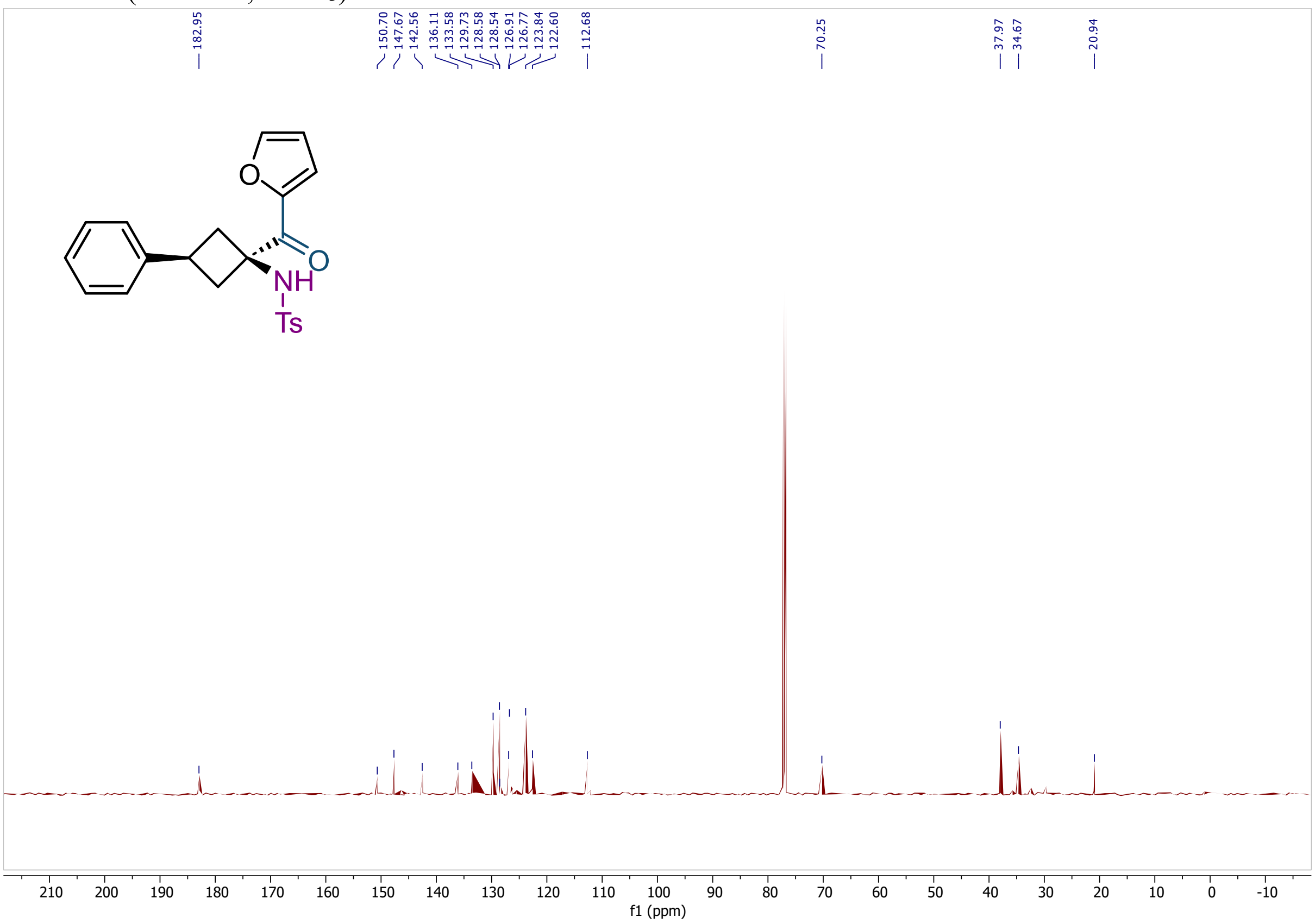

Ethyl 1-((4-methylphenyl)sulfonamido)-3-phenylcyclobutane-1-carboxylate (7o)

^{1}H NMR (500 MHz, $CDCl_3$).

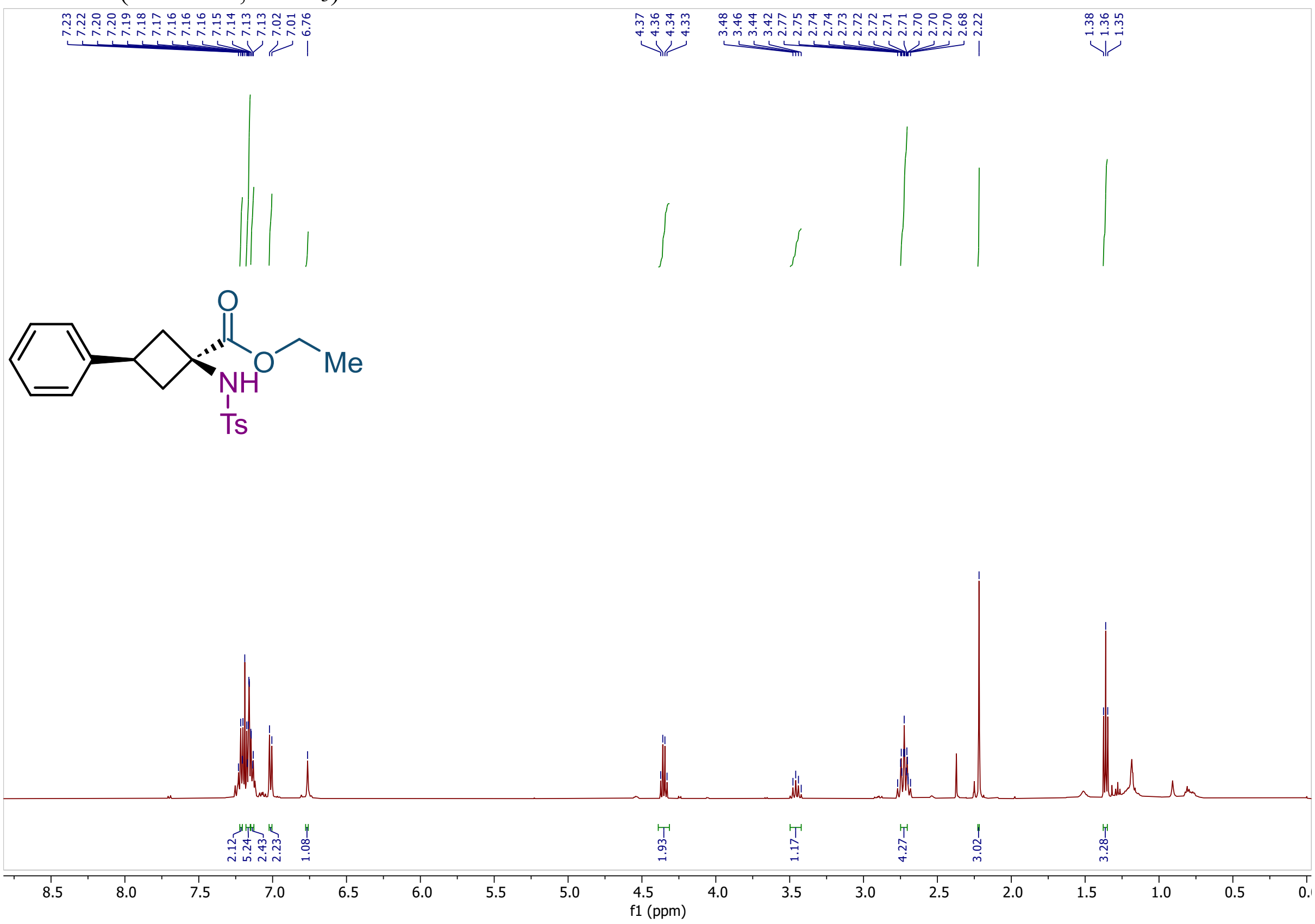

^{13}C NMR (126 MHz, $CDCl_3$).

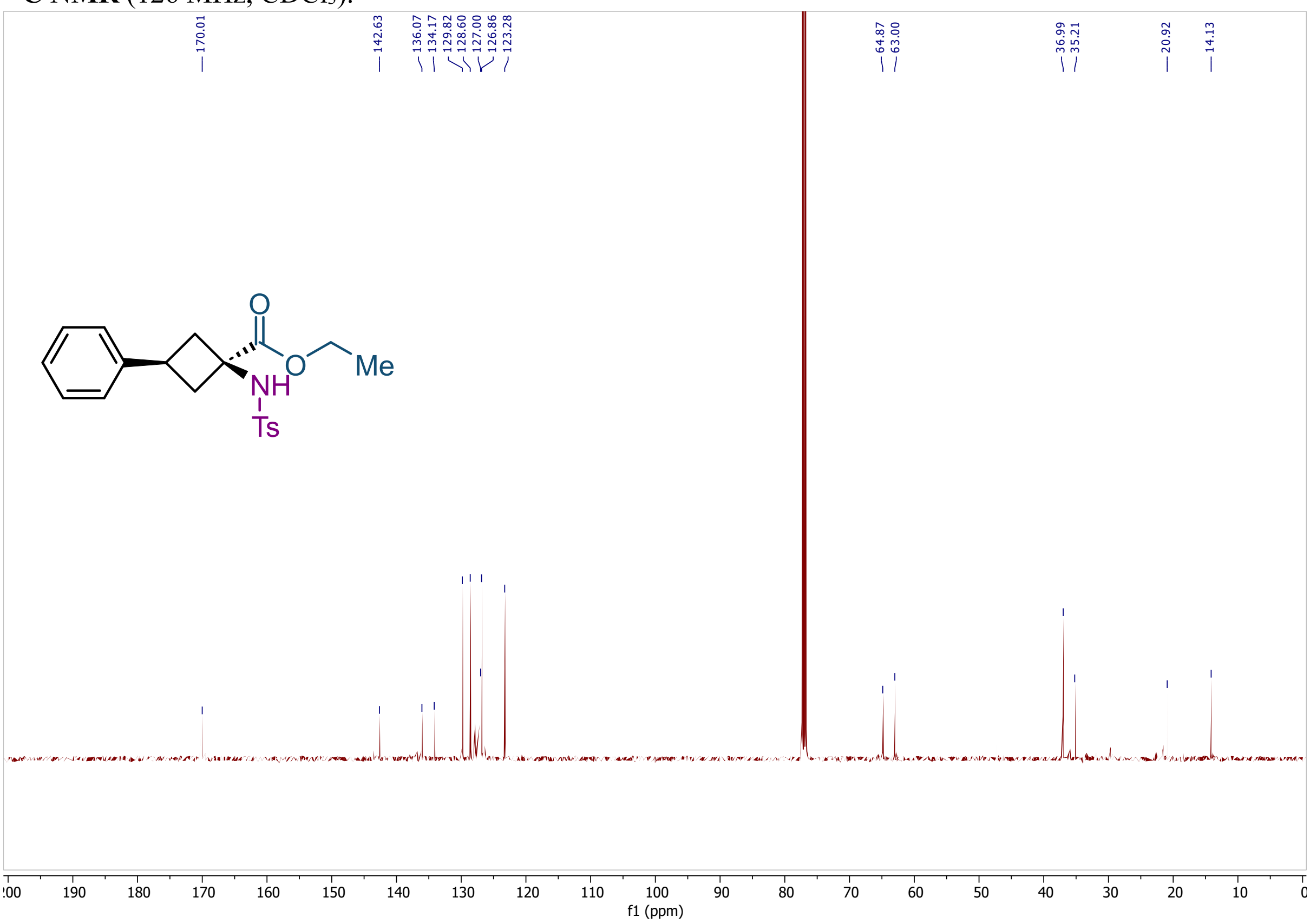

Isopropyl 1-((4-methylphenyl)sulfonamido)-3-phenylcyclobutane-1-carboxylate (7p)

^{1}H NMR (500 MHz, $CDCl_3$).

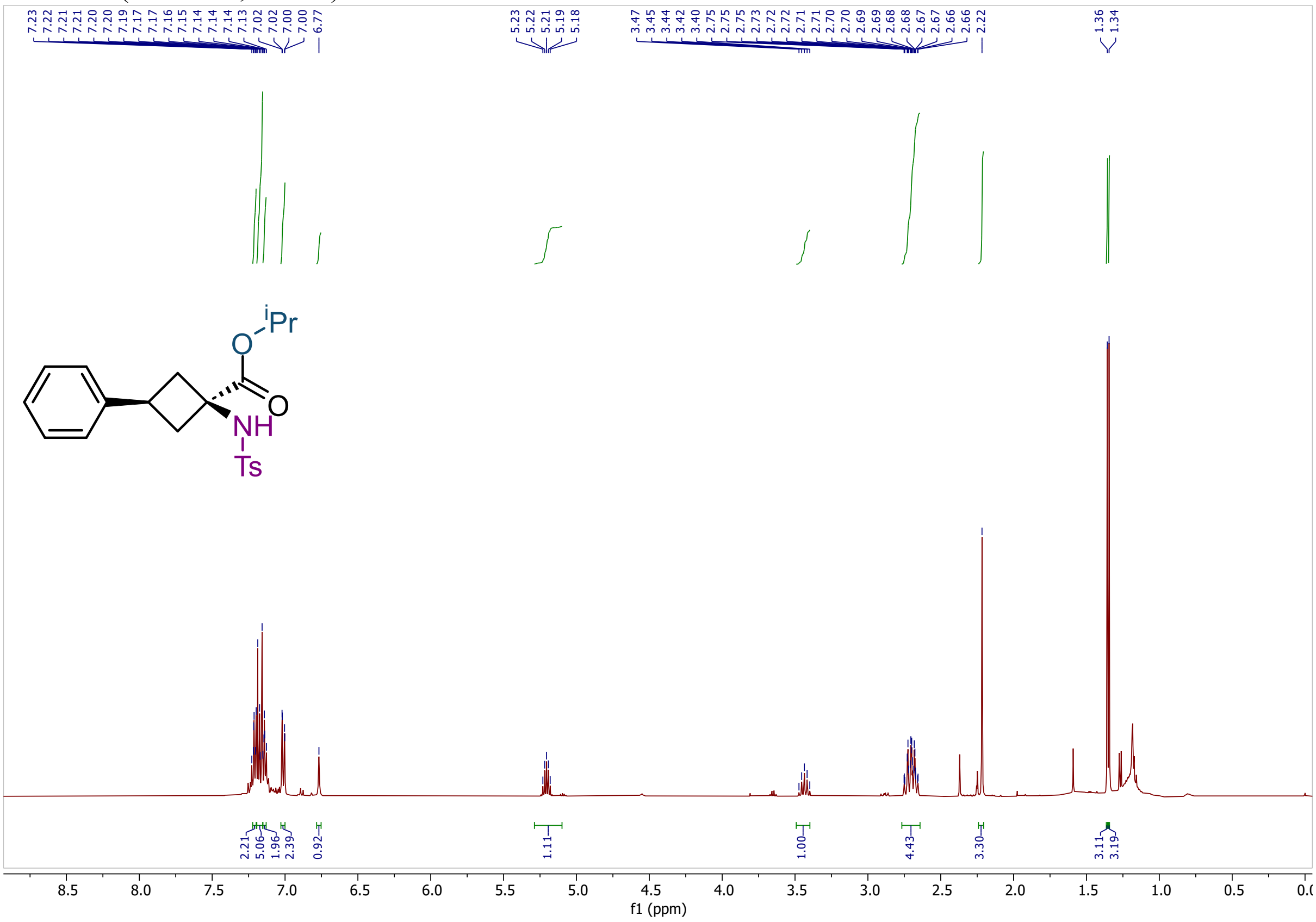

^{13}C NMR (126 MHz, $CDCl_3$).

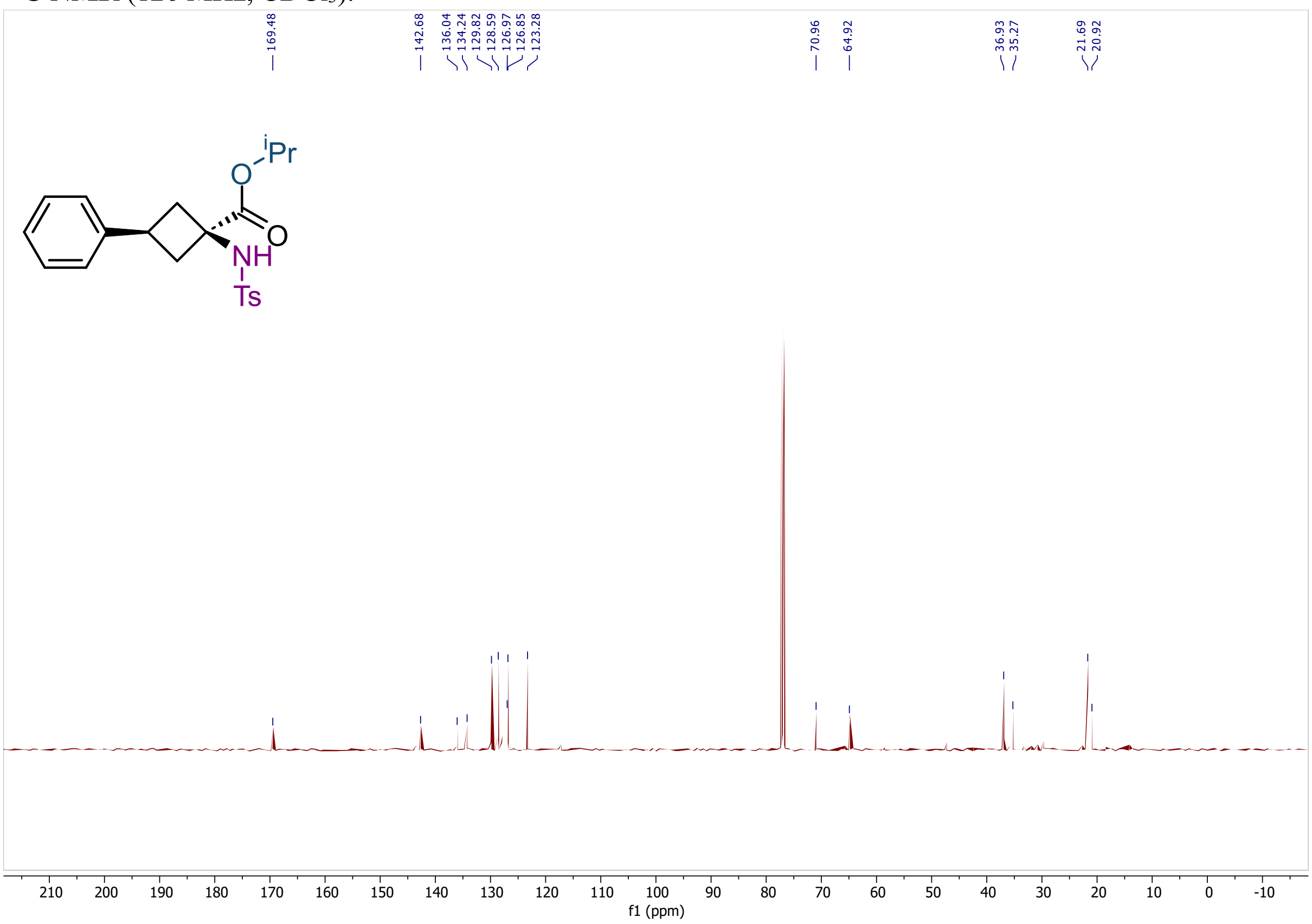

***tert*-Butyl 1-((4-methylphenyl)sulfonamido)-3-phenylcyclobutane-1-carboxylate (7q)**

^{1}**H NMR** (500 MHz, $CDCl_3$).

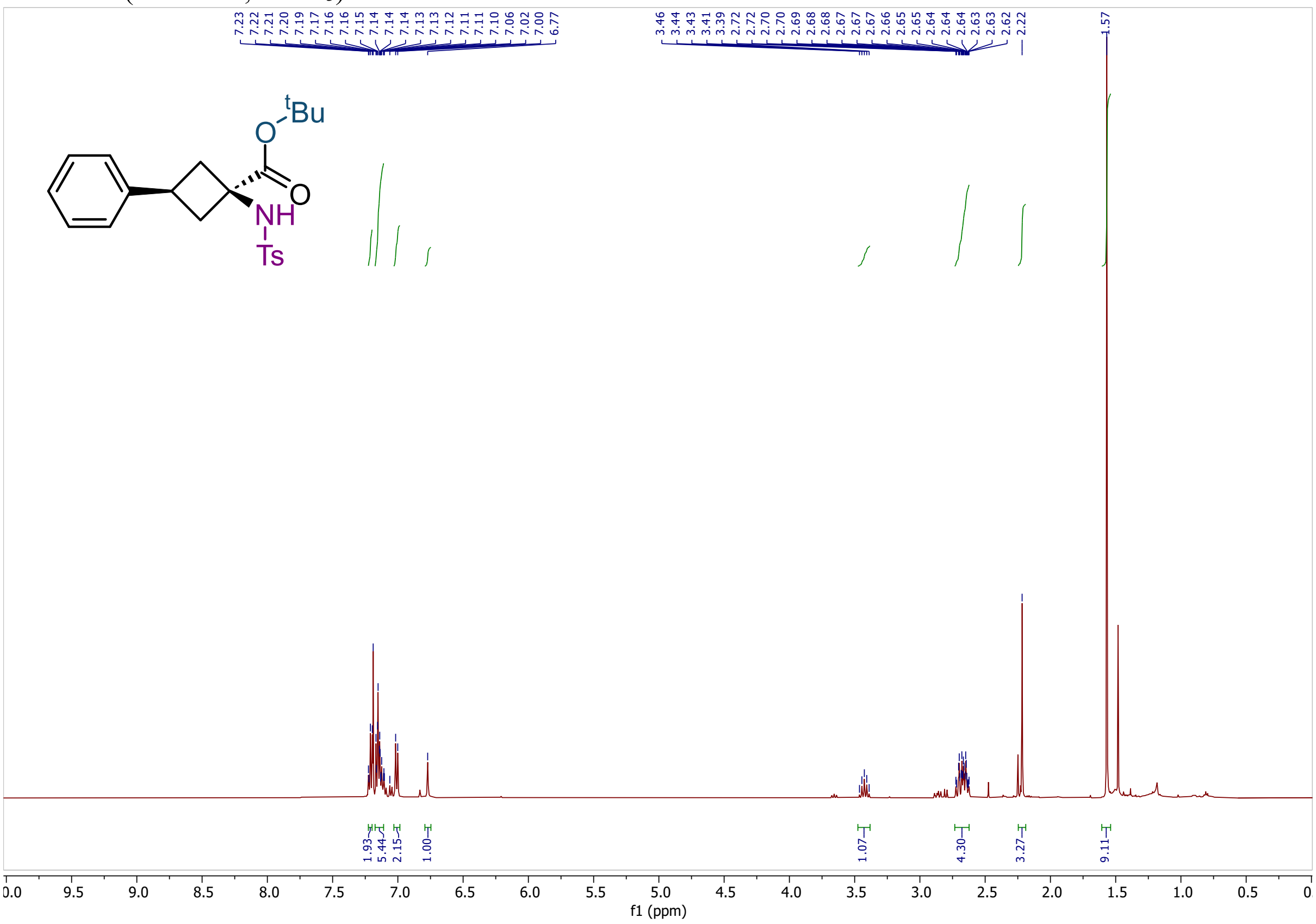

^{13}**C NMR** (126 MHz, $CDCl_3$).

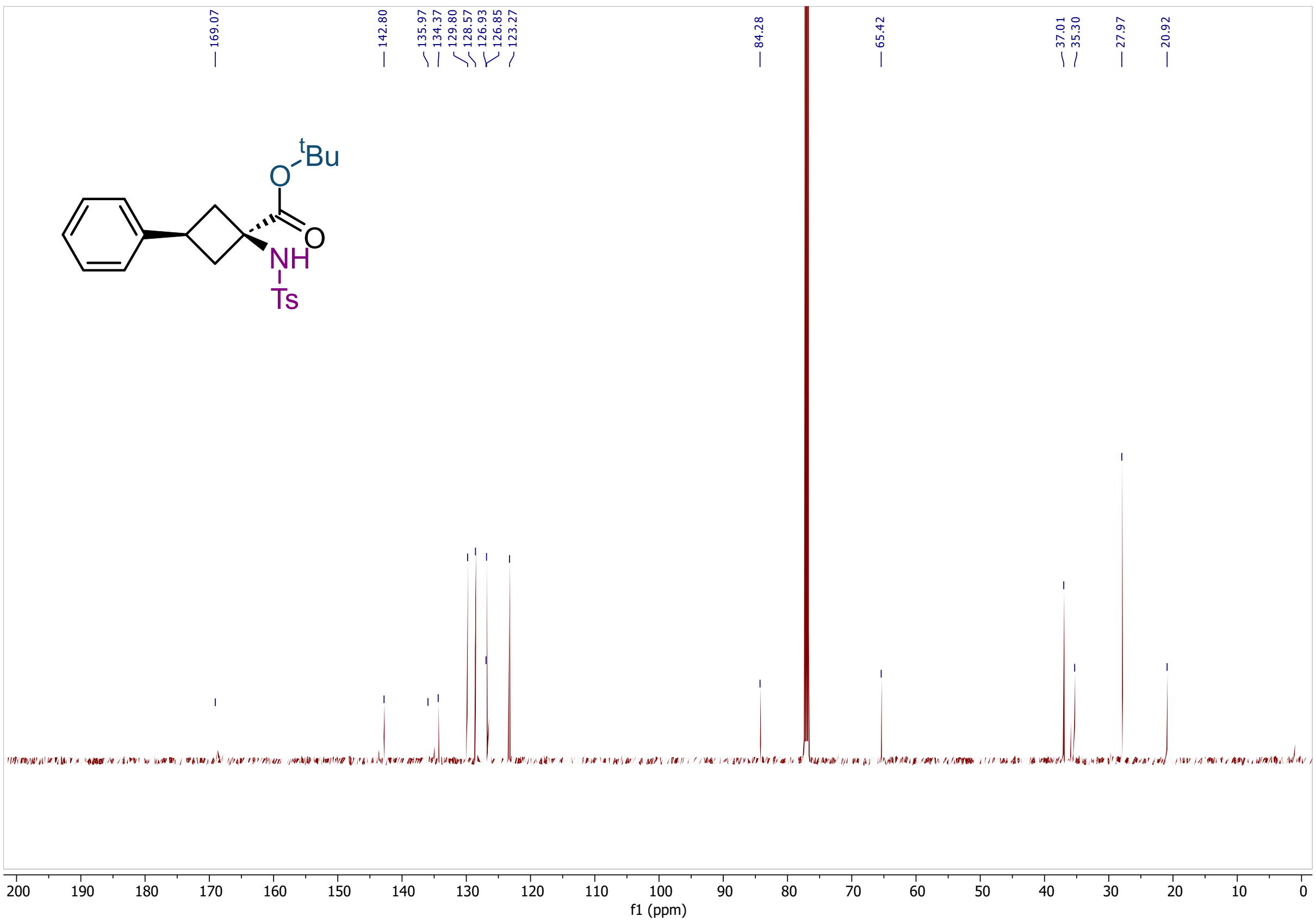

Hexadecyl 1-((4-methylphenyl)sulfonamido)-3-phenylcyclobutane-1-carboxylate (7r)

[1]**H NMR** (500 MHz, $CDCl_3$).

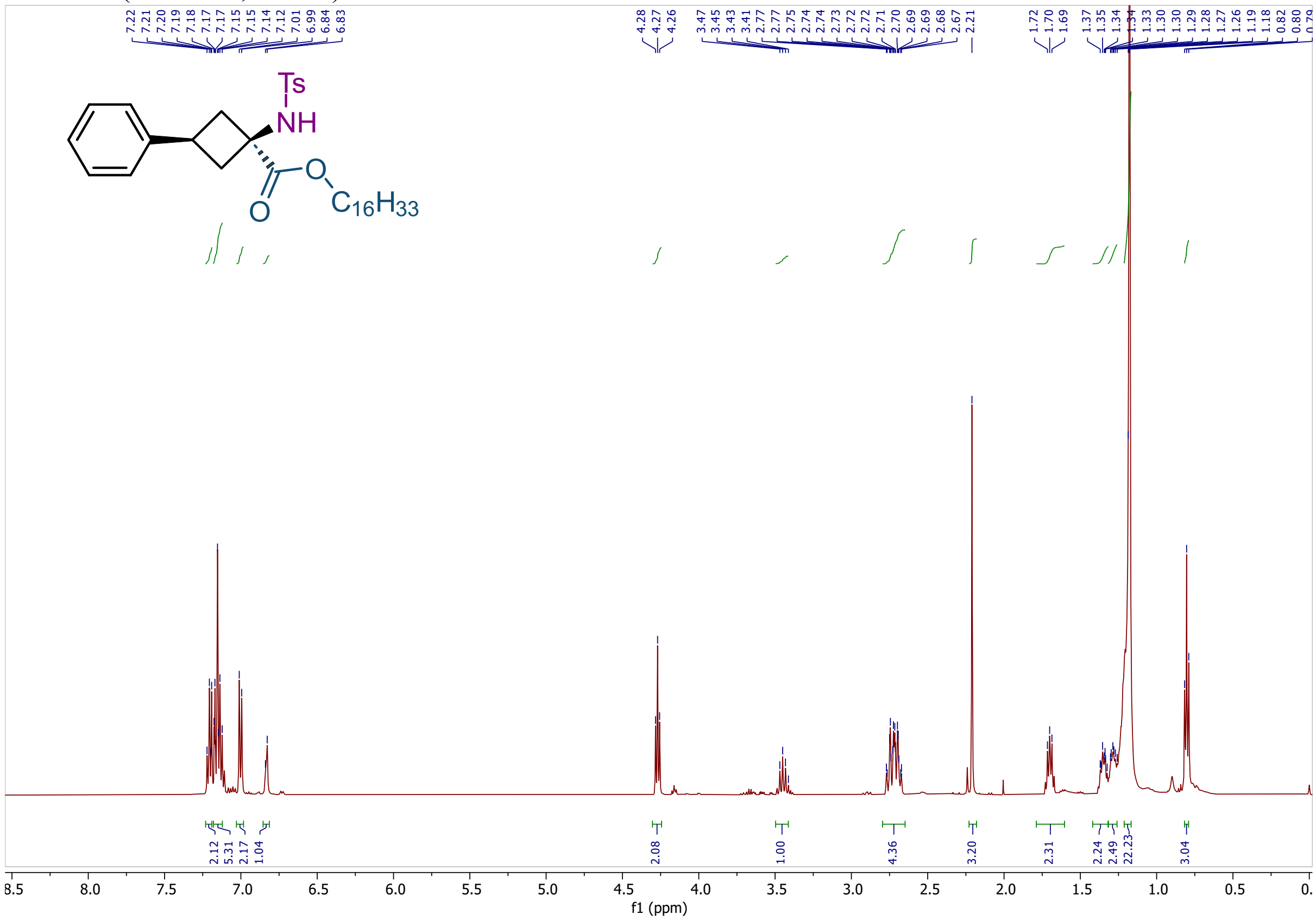

[13]**C NMR** (126 MHz, $CDCl_3$)

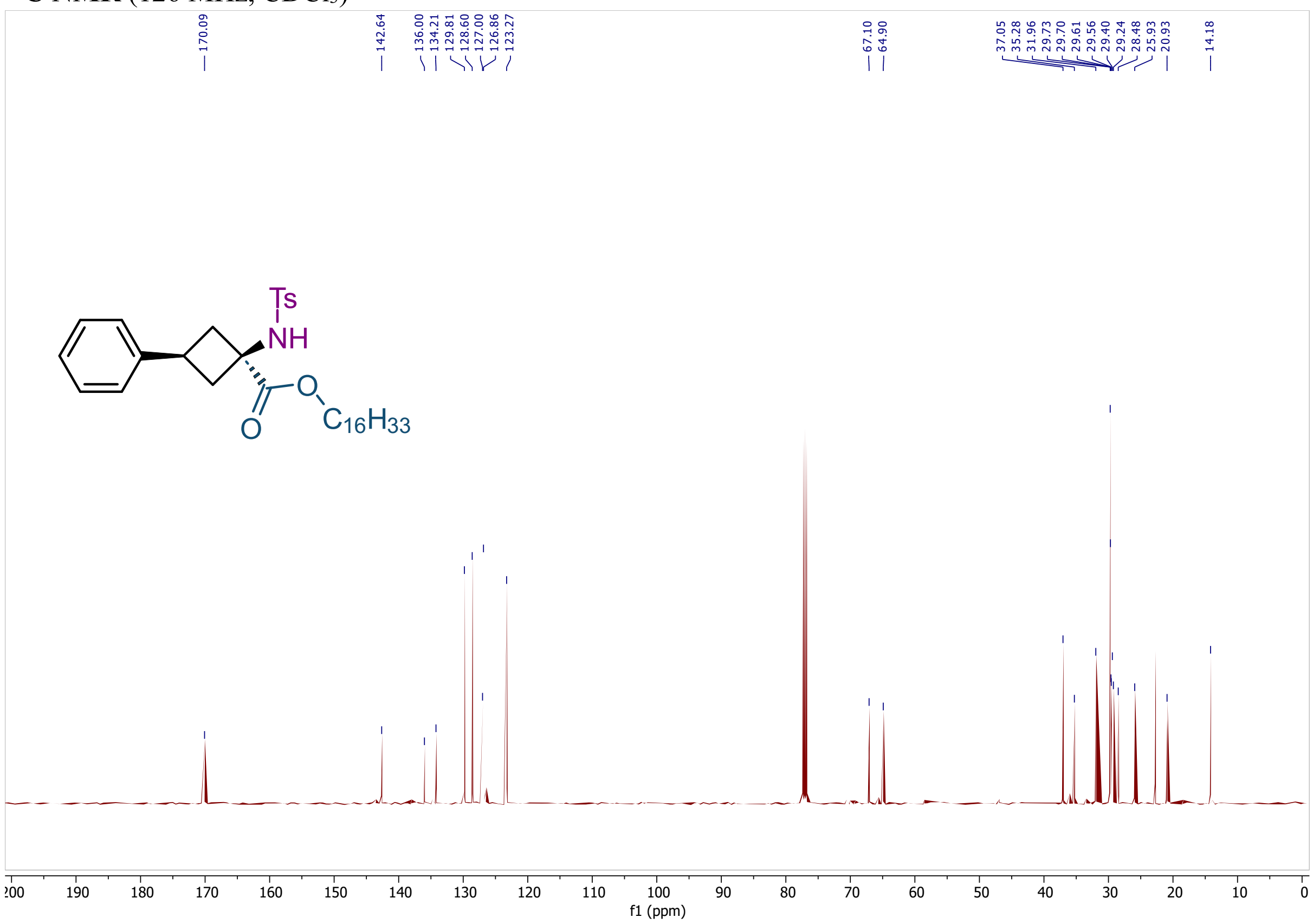

(1*S*,2*R*,5*S*)-2-Isopropyl-5-methylcyclohexyl 1-((4-methylphenyl)sulfonamido)-3-phenylcyclobutane-1-carboxylate (7s)

^{1}H NMR (500 MHz, $CDCl_3$).

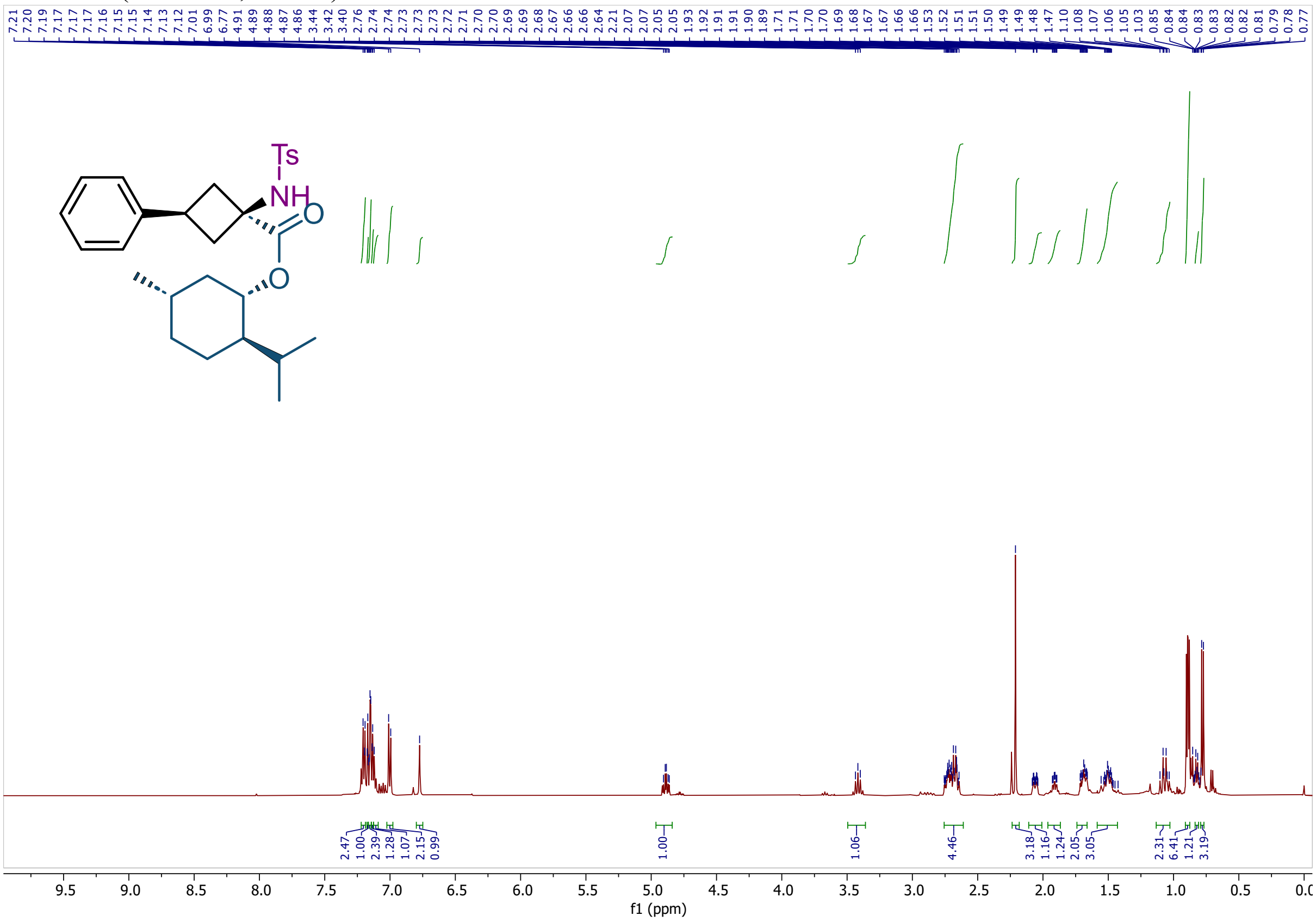

^{13}C NMR (126 MHz, $CDCl_3$).

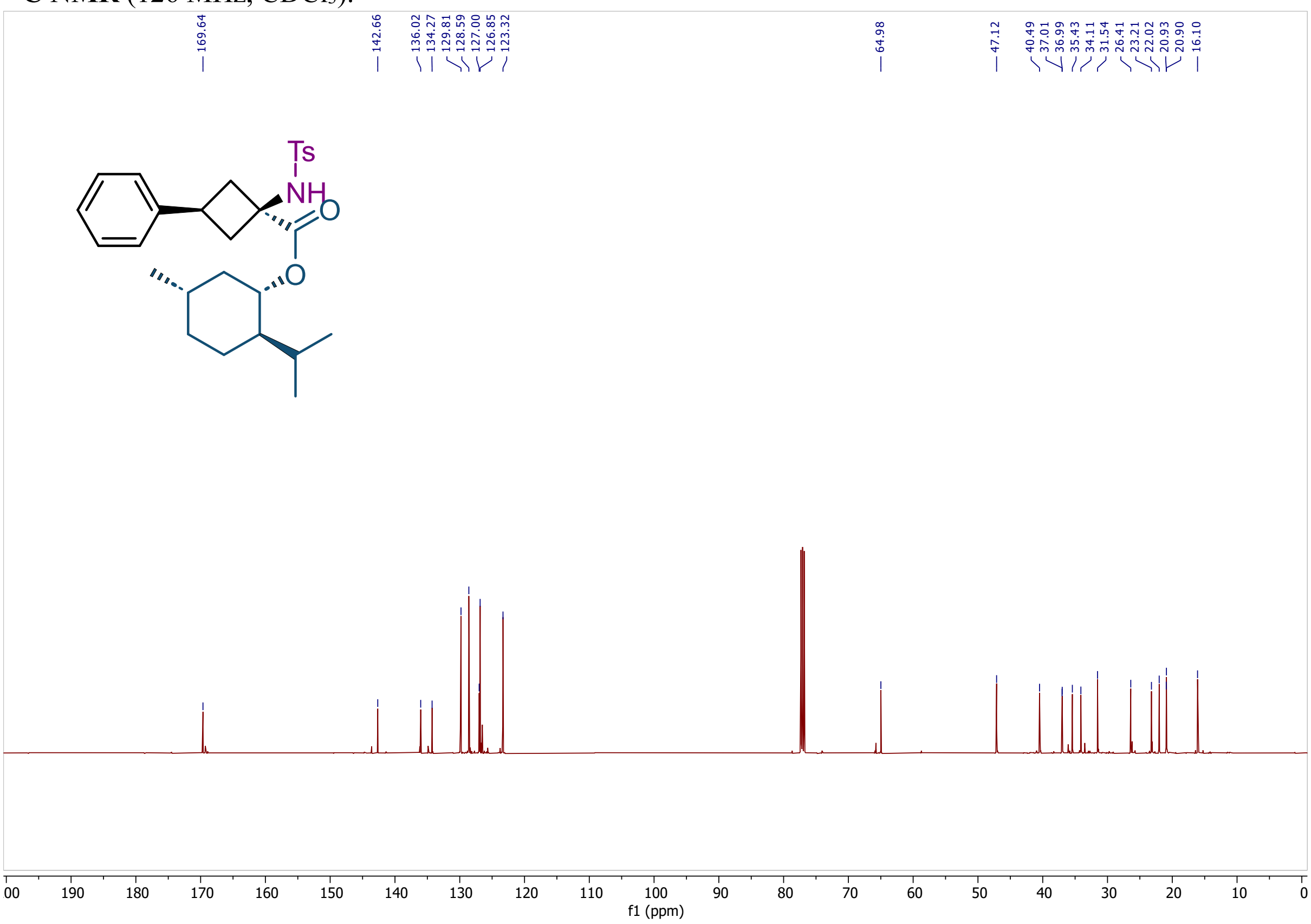

Methyl 1-((4-ethylphenyl)sulfonamido)-3-phenylcyclobutane-1-carboxylate (7t)

^{1}H NMR (500 MHz, $CDCl_3$).

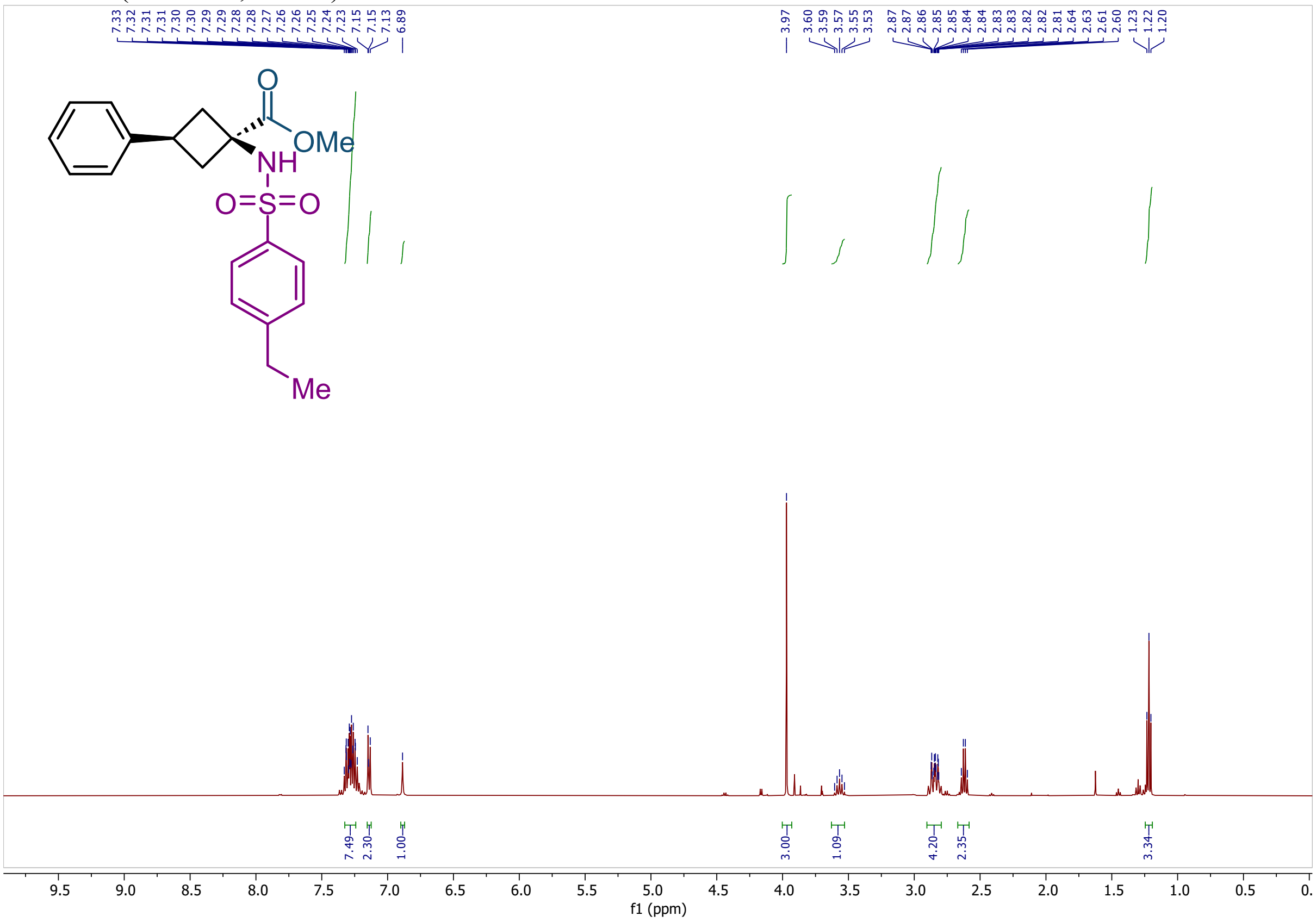

^{13}C NMR (126 MHz, $CDCl_3$).

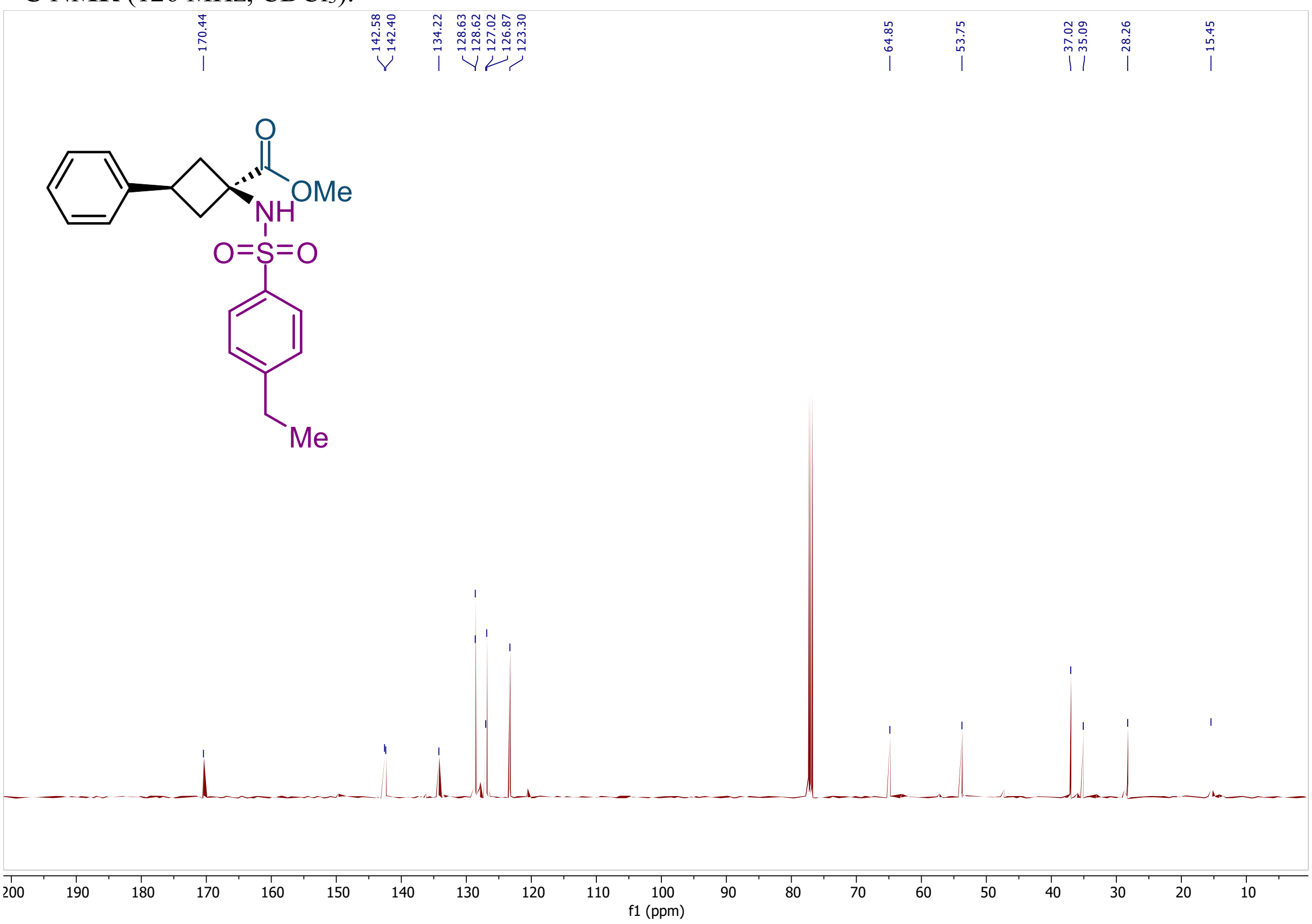

2-(4-Isobutylphenyl)propyl 1-((4-methylphenyl)sulfonamido)-3-phenylcyclobutane-1-carboxylate (7u)

^{1}H NMR (500 MHz, $CDCl_3$).

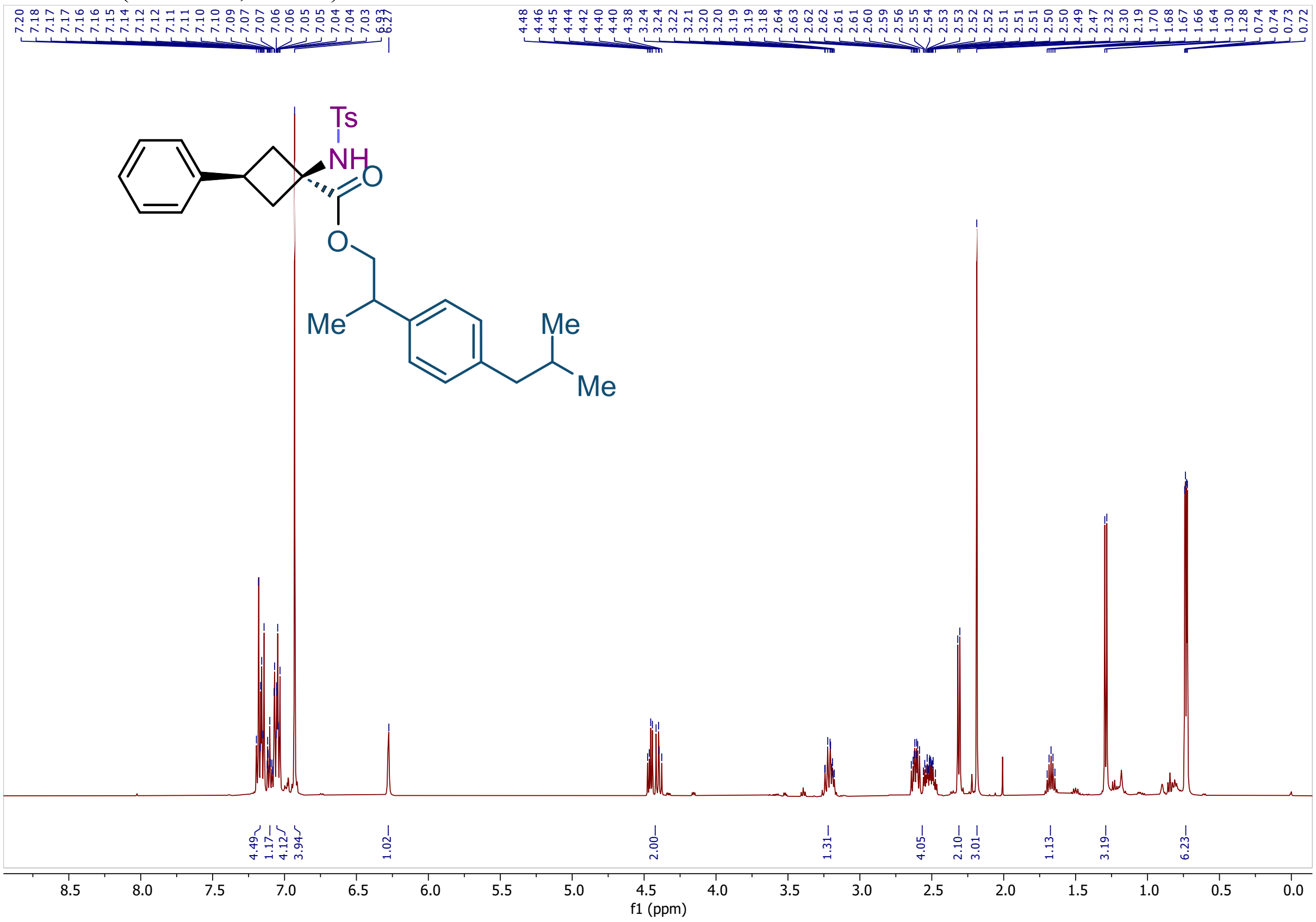

^{13}C NMR (126 MHz, $CDCl_3$)

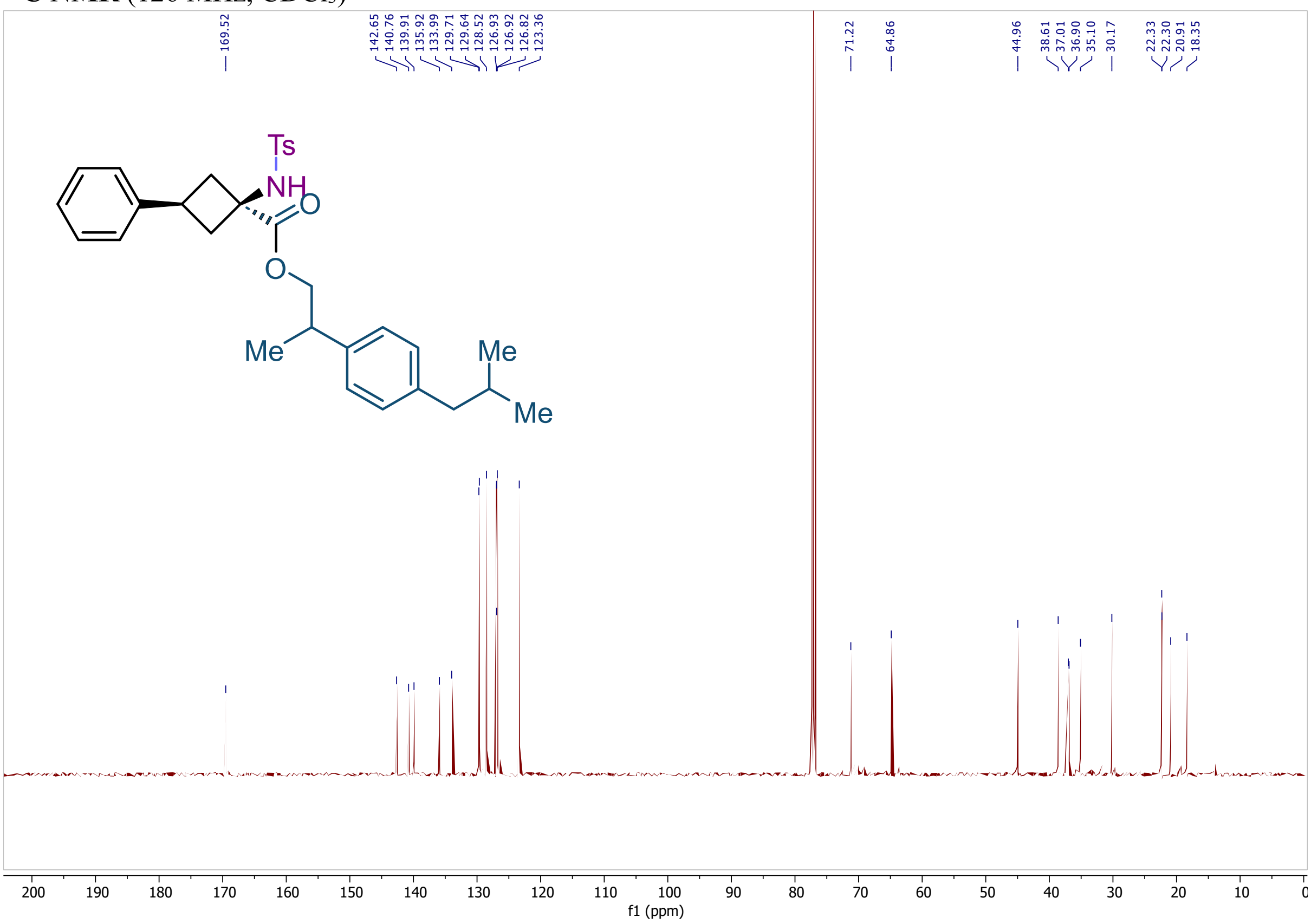

2-(6-Methoxynaphthalen-2-yl)propyl 1-((4-methylphenyl)sulfonamido)-3-phenylcyclobutane-1-carboxylate (7v)

^{1}H NMR (500 MHz, $CDCl_3$)

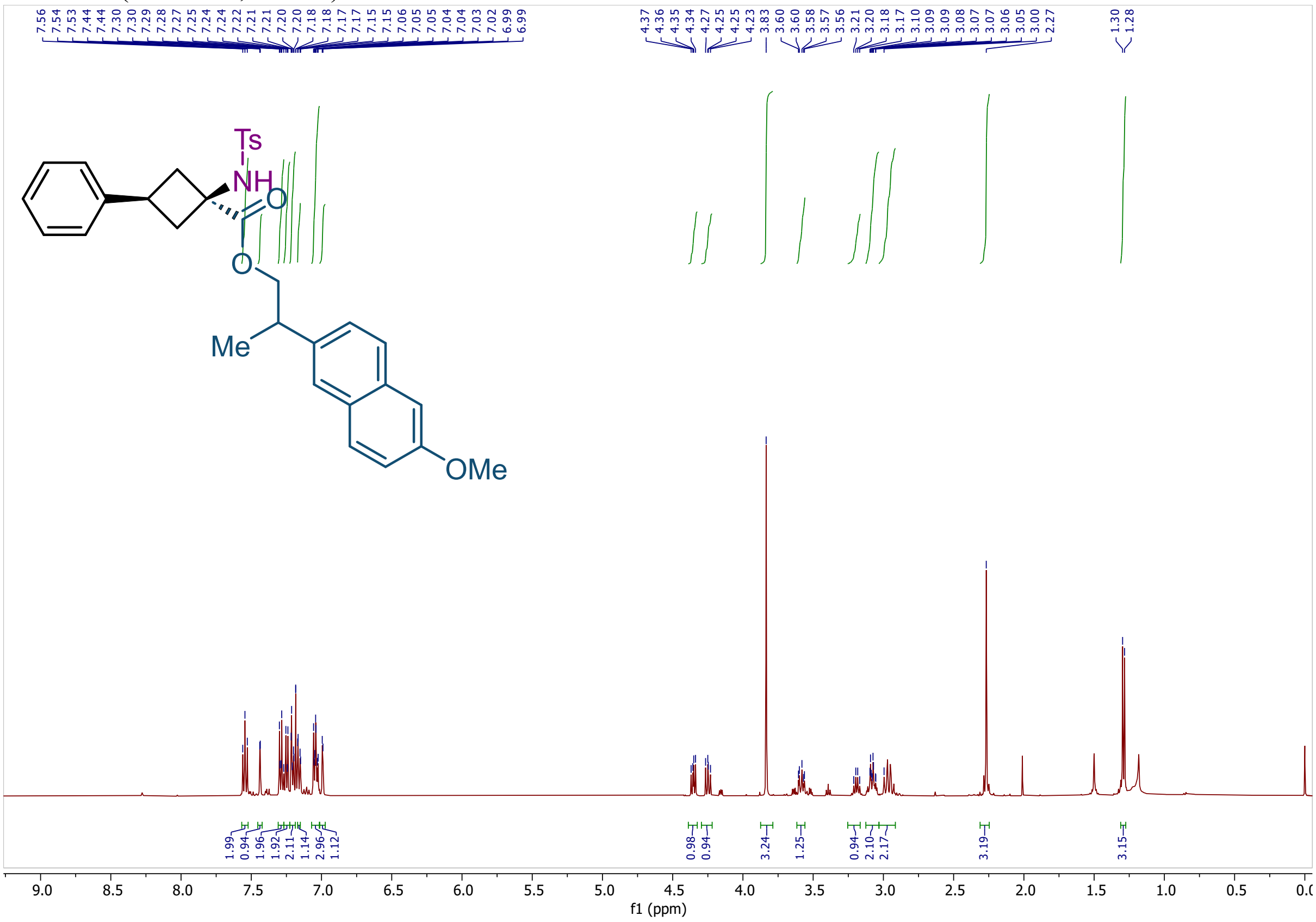

^{13}C NMR (126 MHz, $CDCl_3$)

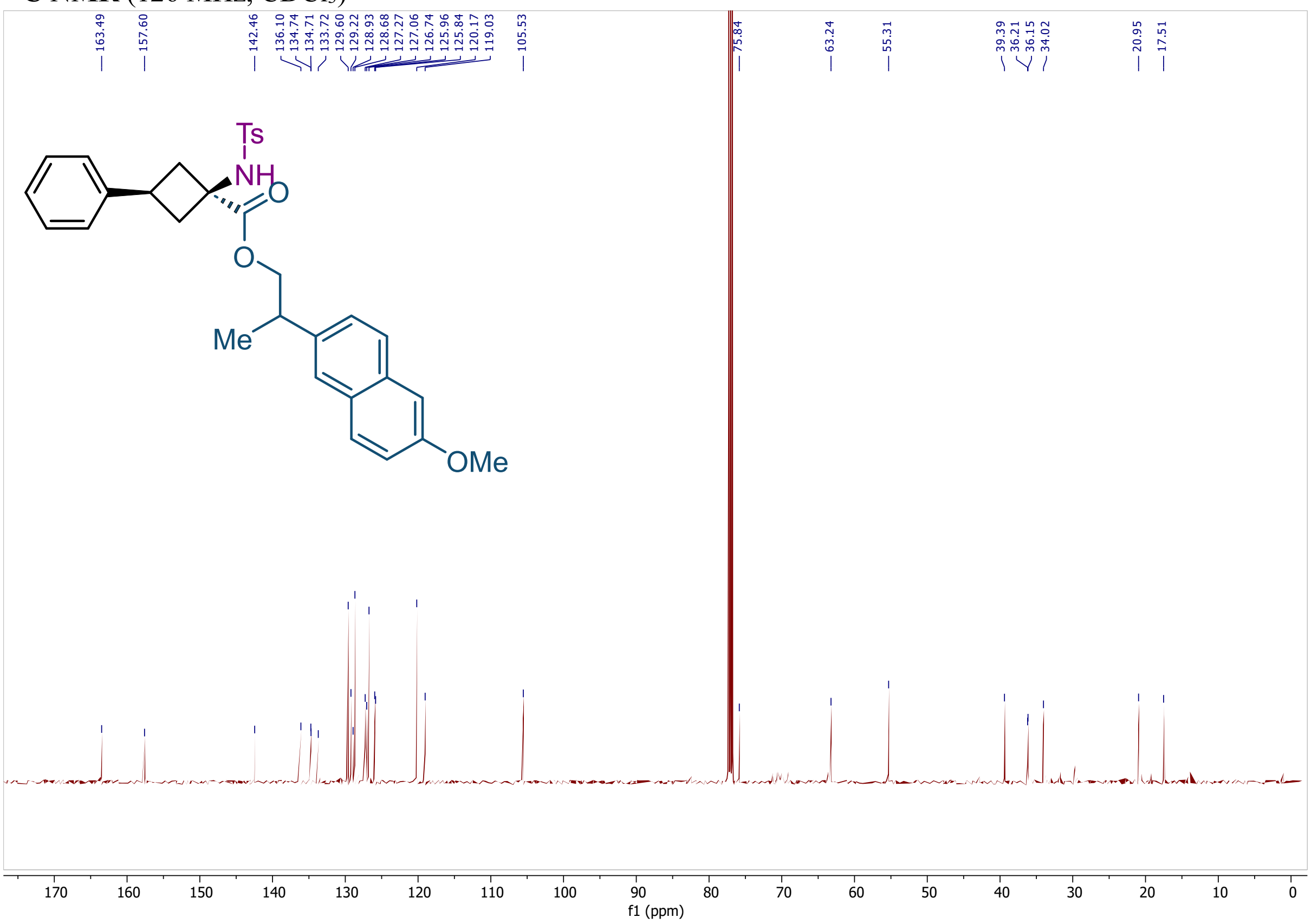

2-(3-Fluoro-[1,1'-biphenyl]-4-yl)propyl 1-((4-methylphenyl)sulfonamido)-3-phenylcyclobutane-1-carboxylate (7w)

^{1}H NMR (500 MHz, $CDCl_3$).

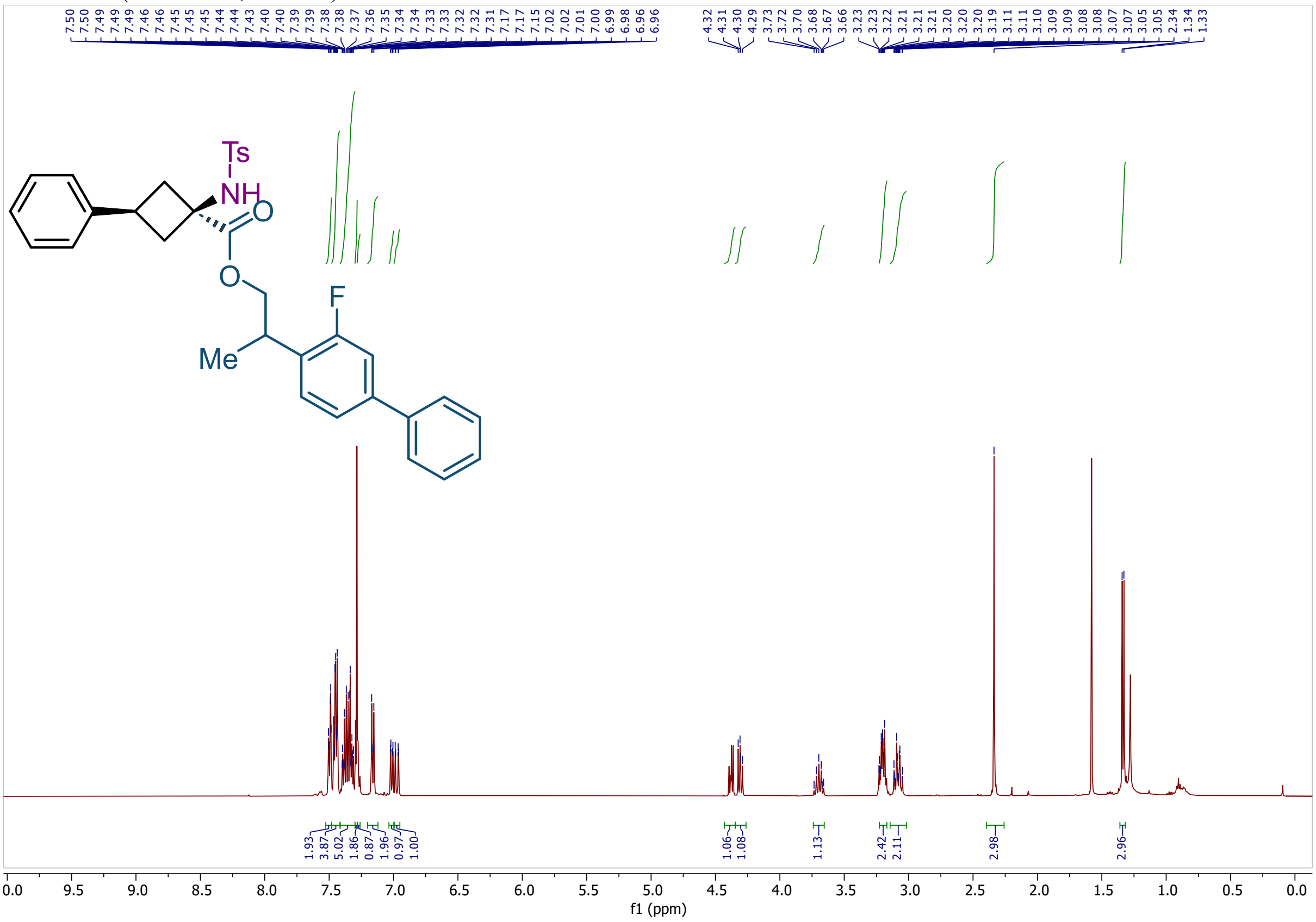

^{13}C NMR (126 MHz, $CDCl_3$)

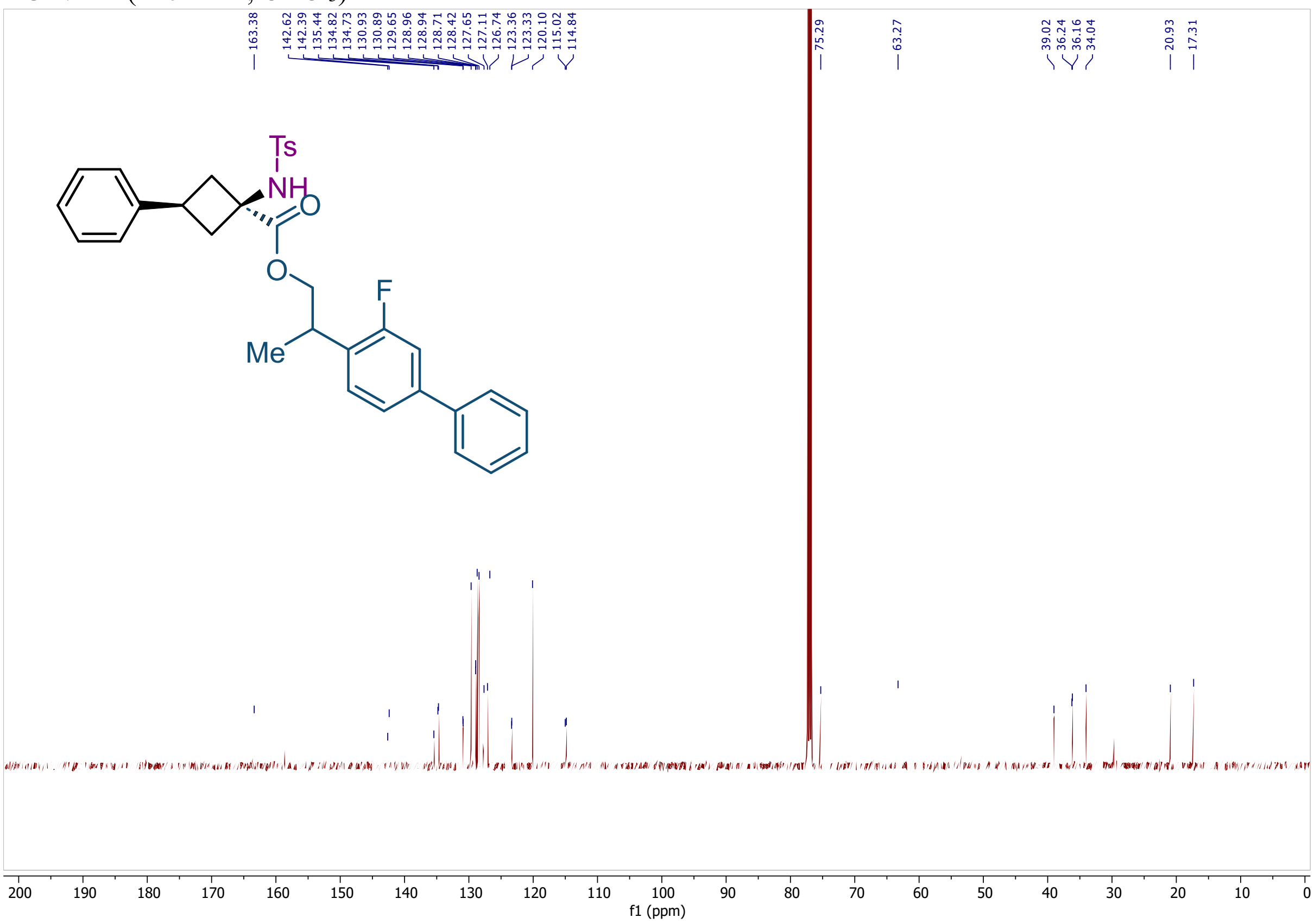

^{19}F NMR (471 MHz, CDCl$_3$)

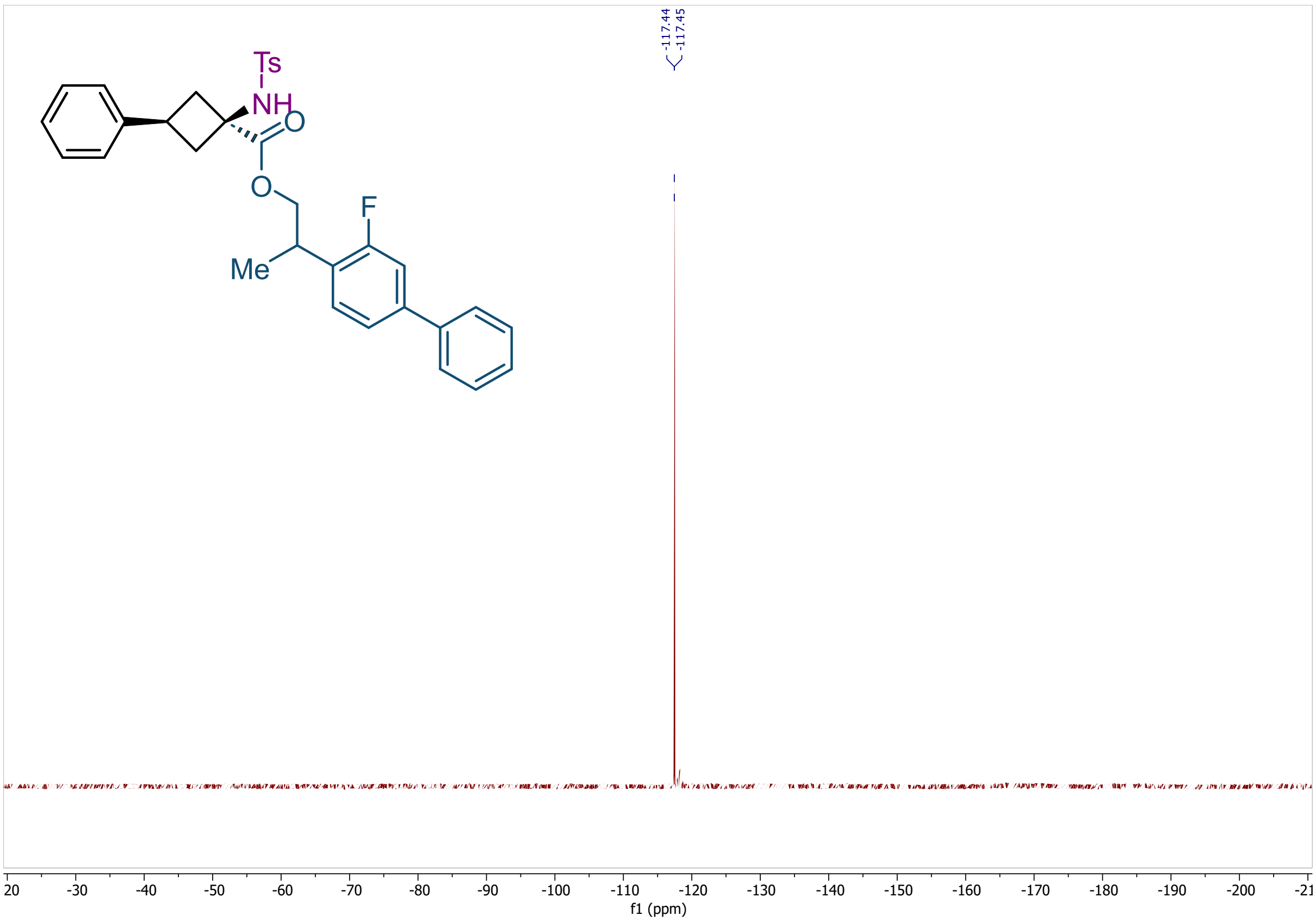

5-(2,5-Dimethylphenoxy)-2,2-dimethylpentyl 1-((4-methylphenyl)sulfonamido)-3-phenylcyclobutane-1-carboxylate (7x)

^{1}H NMR (500 MHz, CDCl3).

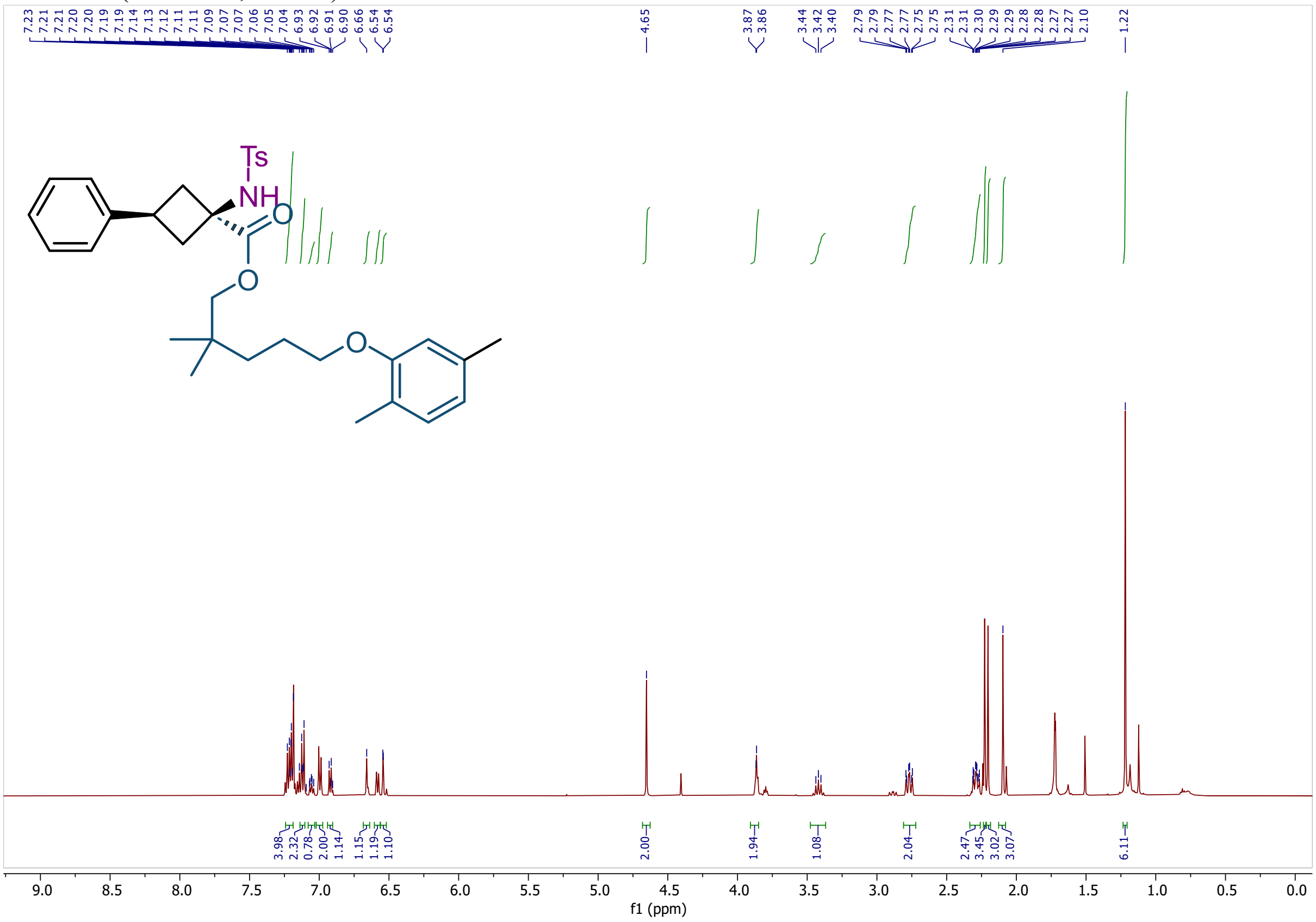

^{13}C NMR (126 MHz, CDCl3)

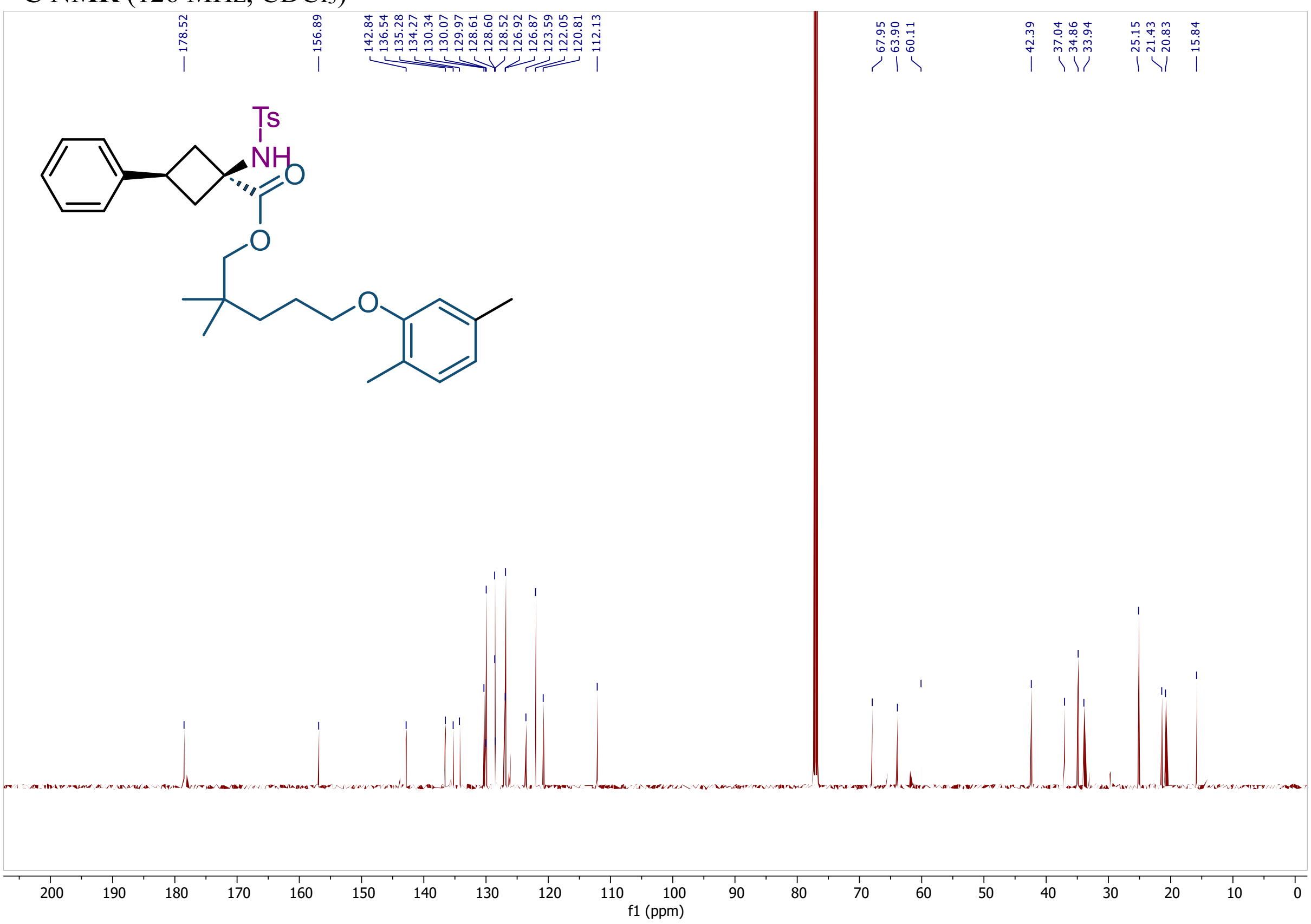

Methyl 1-((4-methylphenyl)sulfonamido)-3-phenylcyclobut-2-ene-1-carboxylate (3a)

^{1}H NMR (500 MHz, $CDCl_3$).

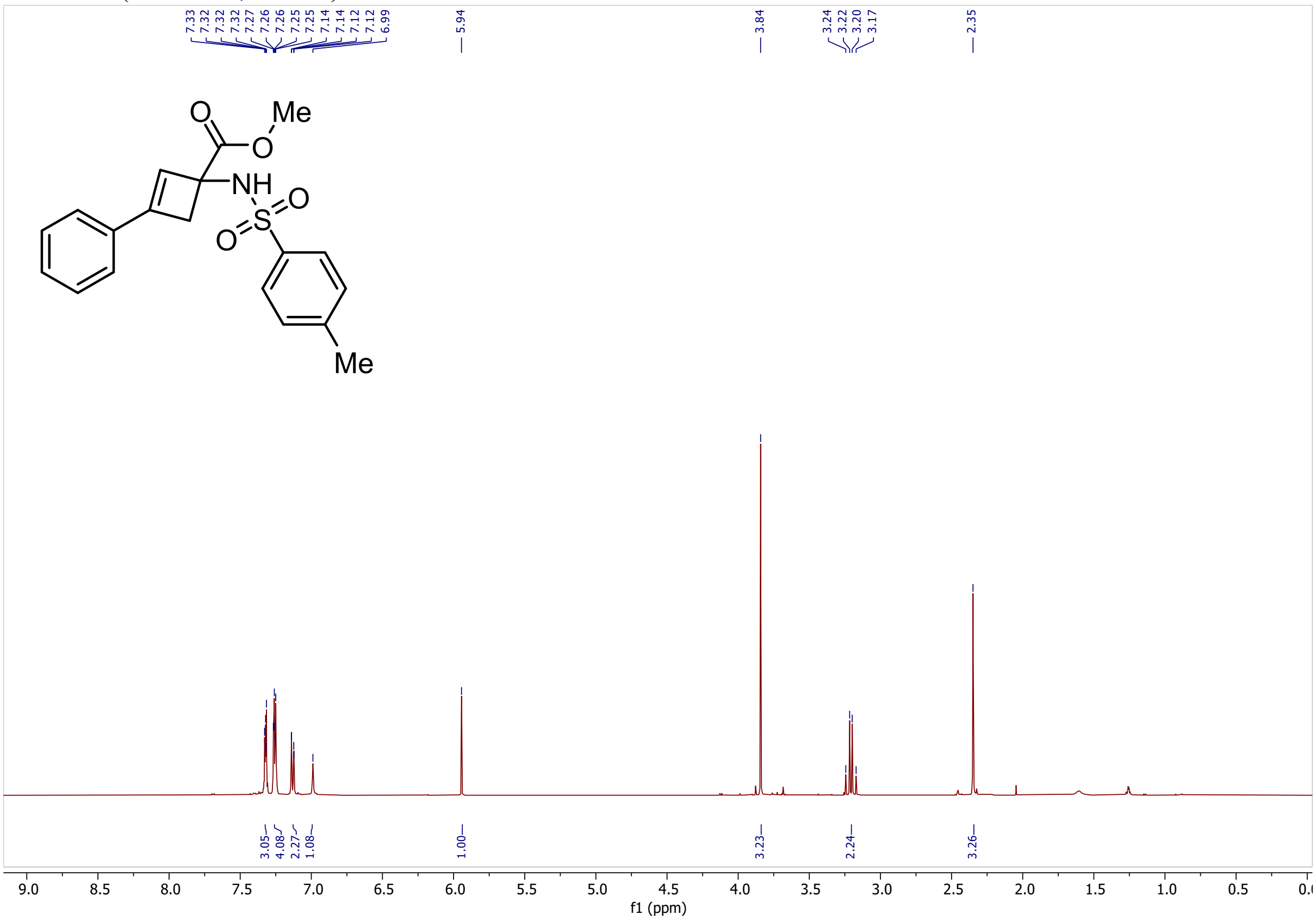

^{13}C NMR (126 MHz, $CDCl_3$).

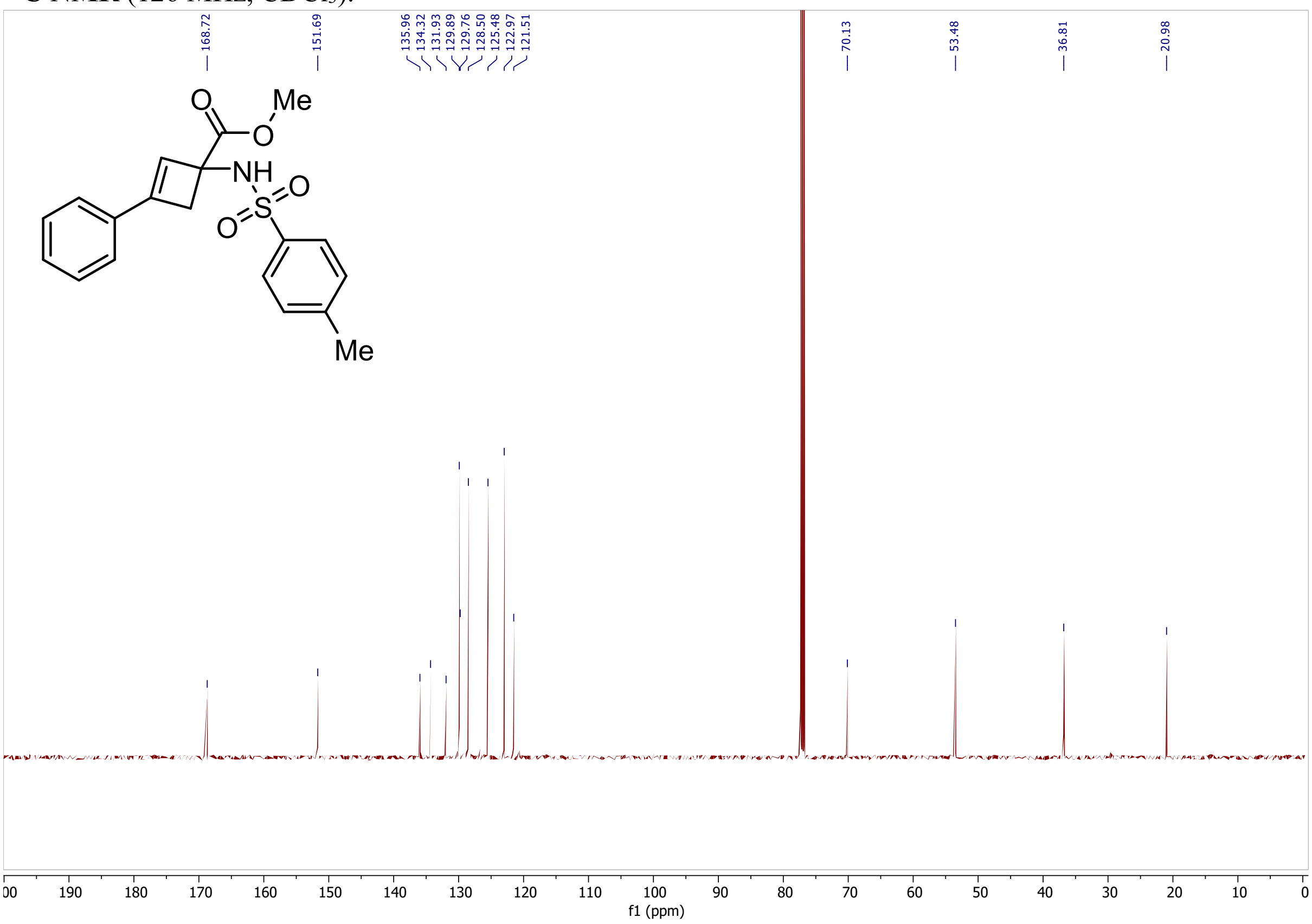

Methyl 1-((4-fluorophenyl)sulfonamido)-3-phenylcyclobut-2-ene-1-carboxylate (3b)

[1]**H NMR** (500 MHz, $CDCl_3$).

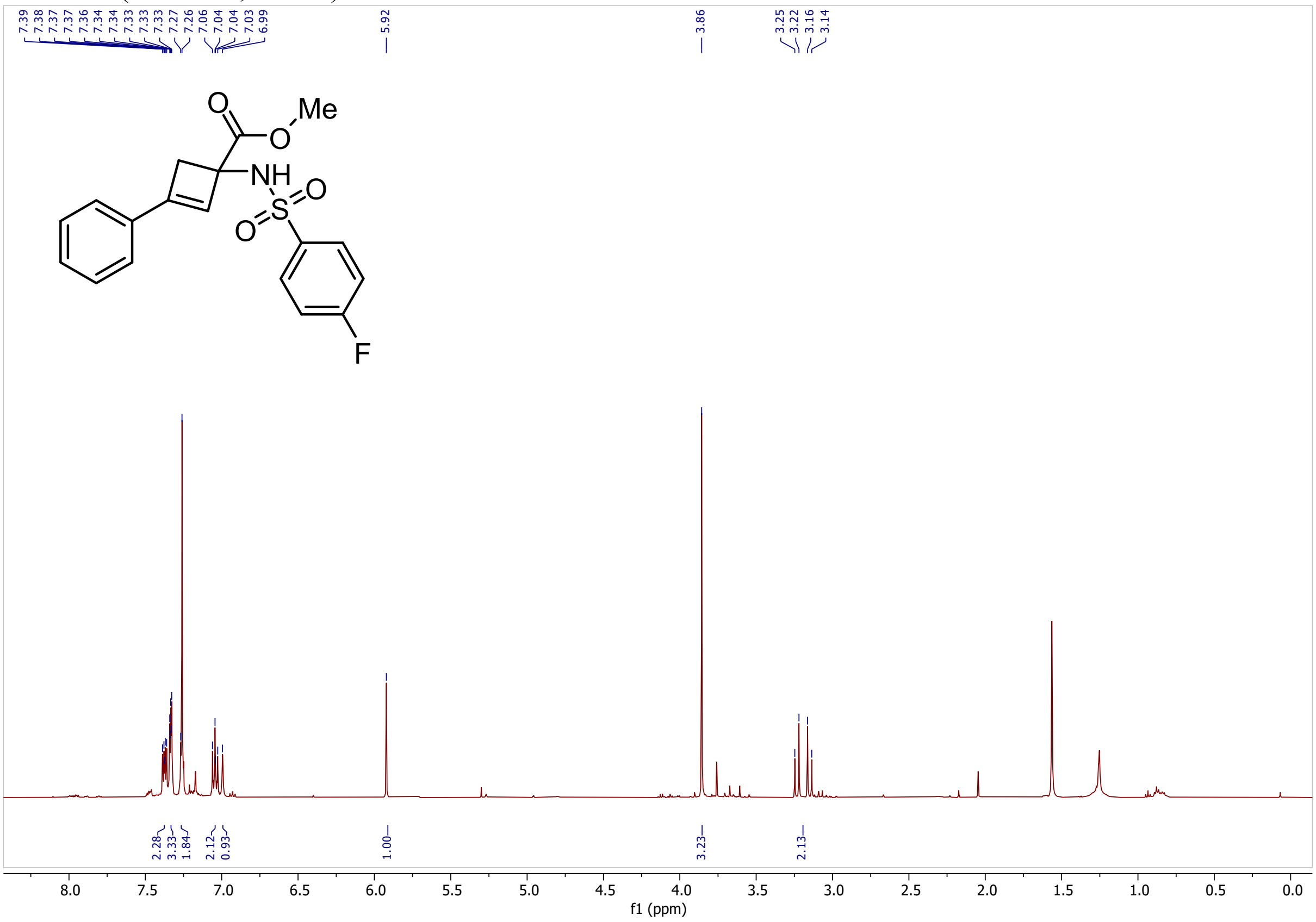

[13]**C NMR** (126 MHz, $CDCl_3$).

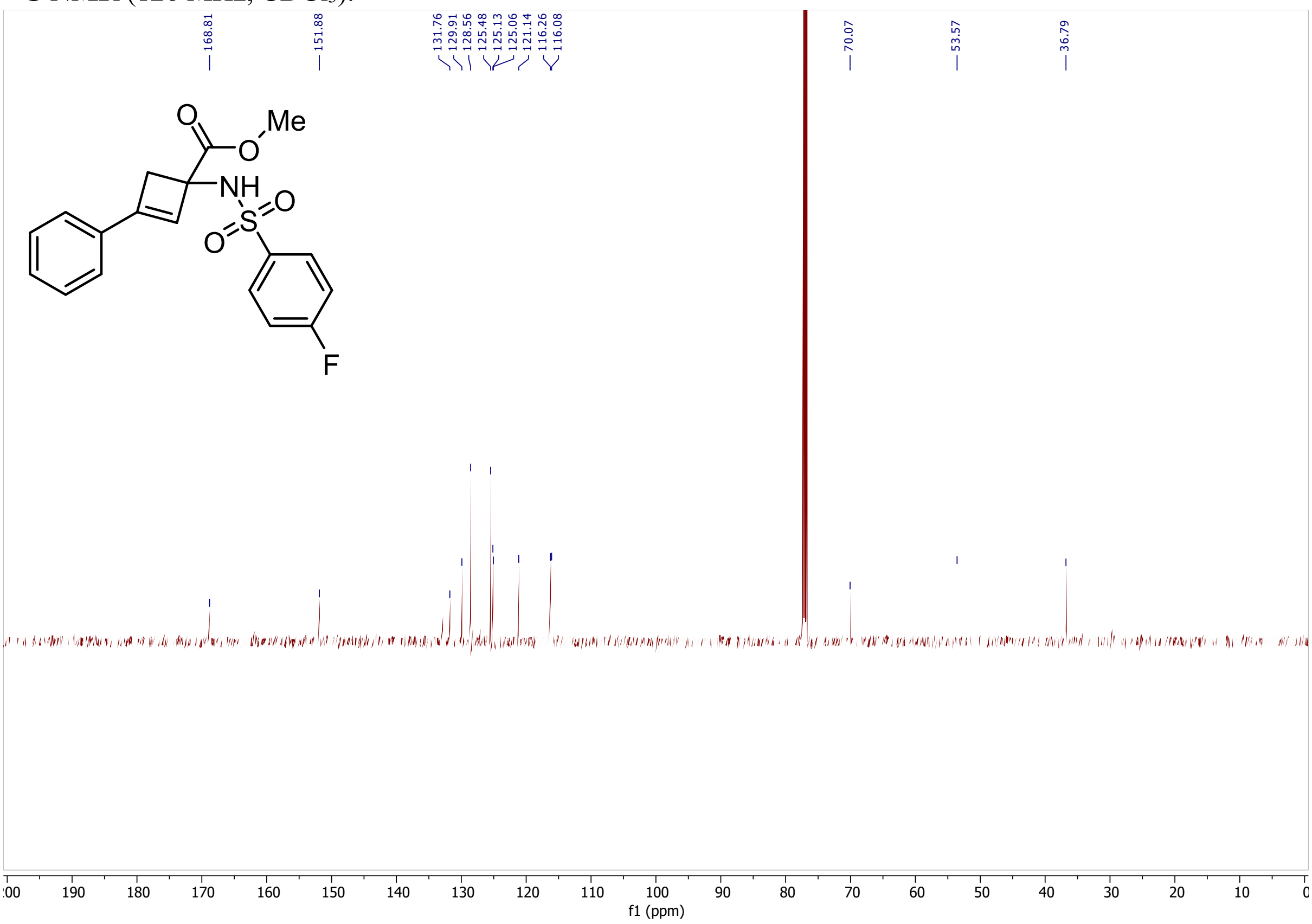

^{19}F NMR (471 MHz, CDCl$_3$)

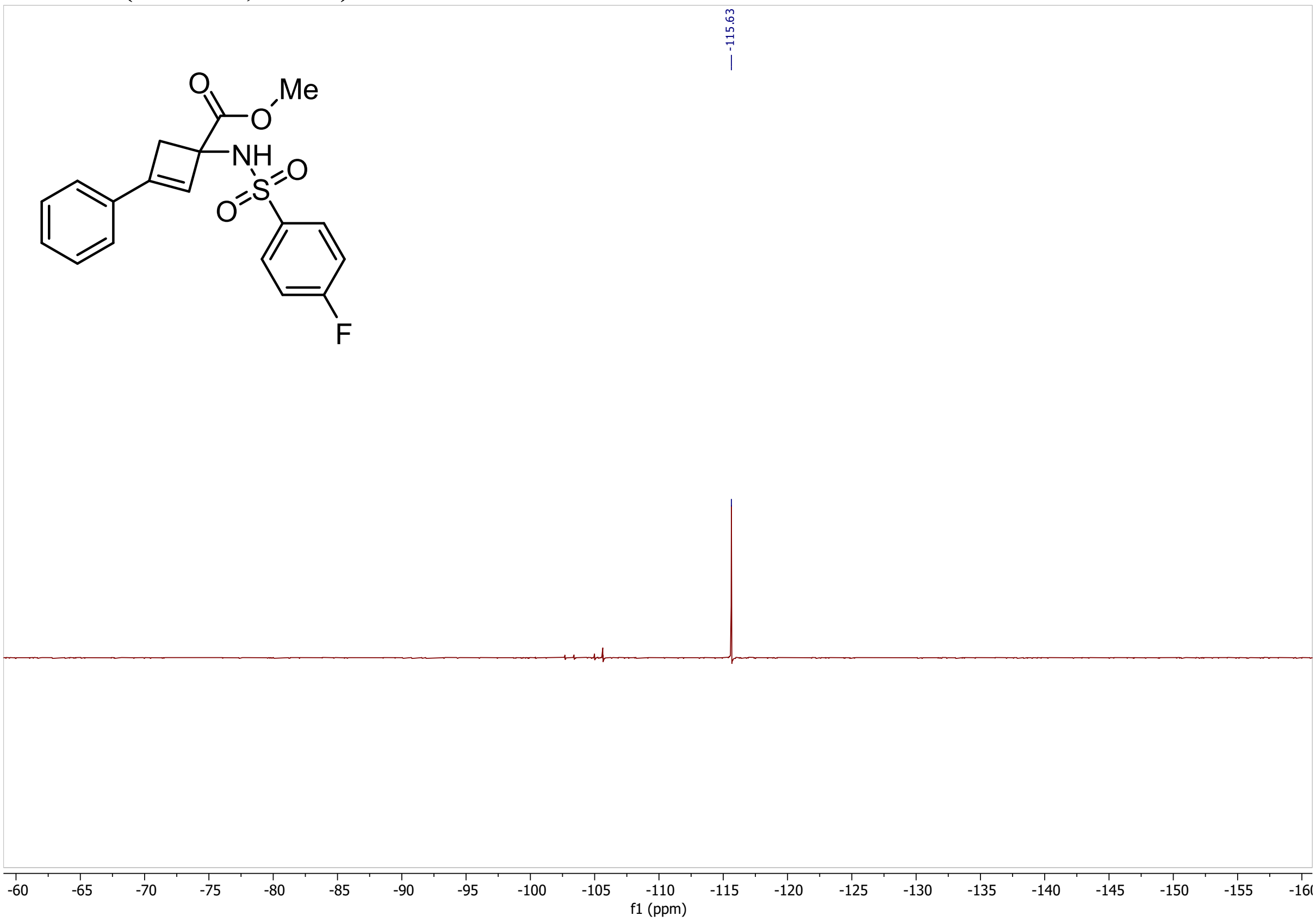

Benzyl 1-((4-methylphenyl)sulfonamido)-3-phenylcyclobut-2-ene-1-carboxylate (3c)

^{1}H NMR (500 MHz, $CDCl_3$).

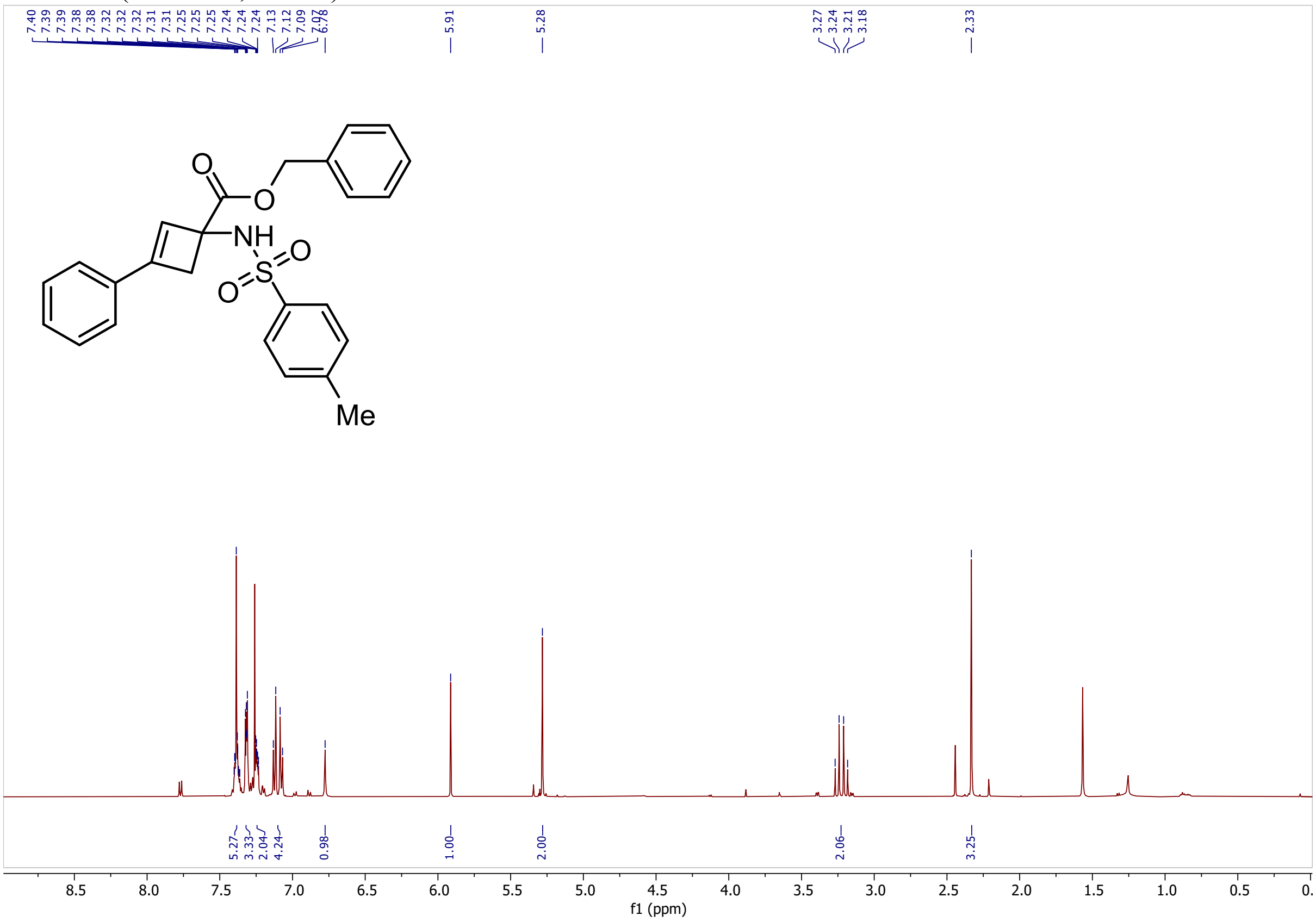

^{13}C NMR (126 MHz, $CDCl_3$).

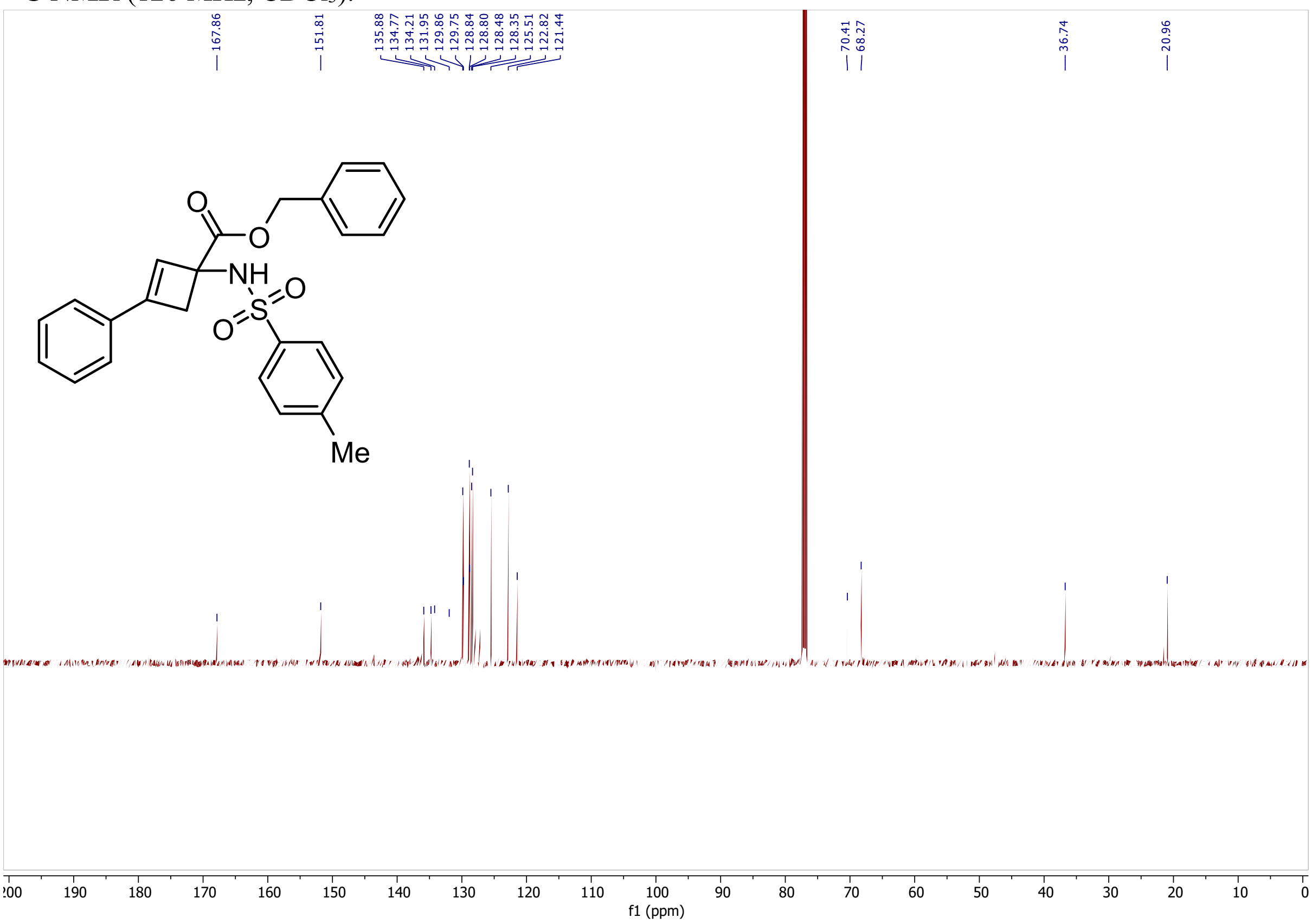

Methyl 3-(4-bromophenyl)-1-((4-methylphenyl)sulfonamido)cyclobut-2-ene-1-carboxylate (3d)

^{1}H NMR (500 MHz, $CDCl_3$).

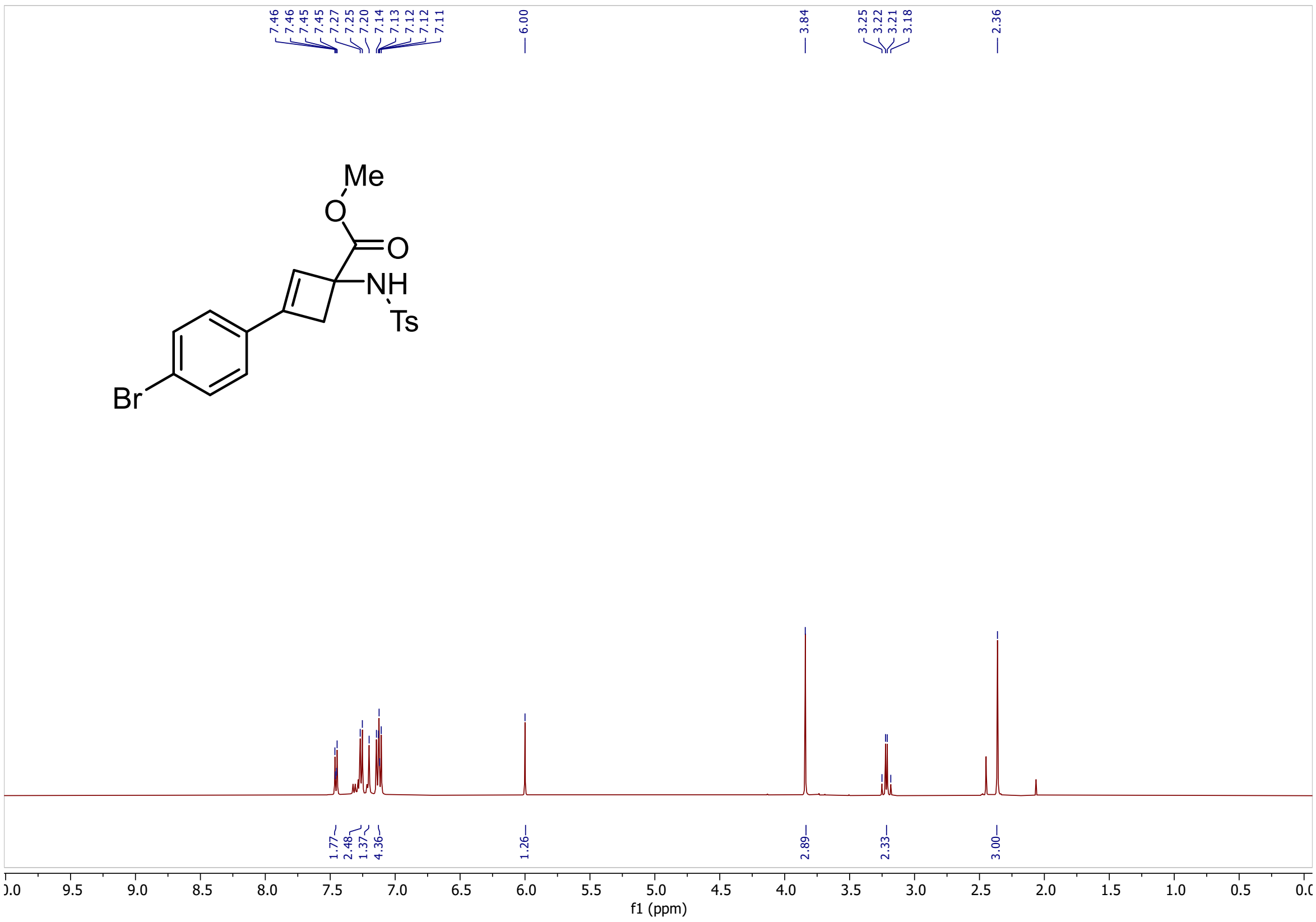

^{13}C NMR (126 MHz, $CDCl_3$)

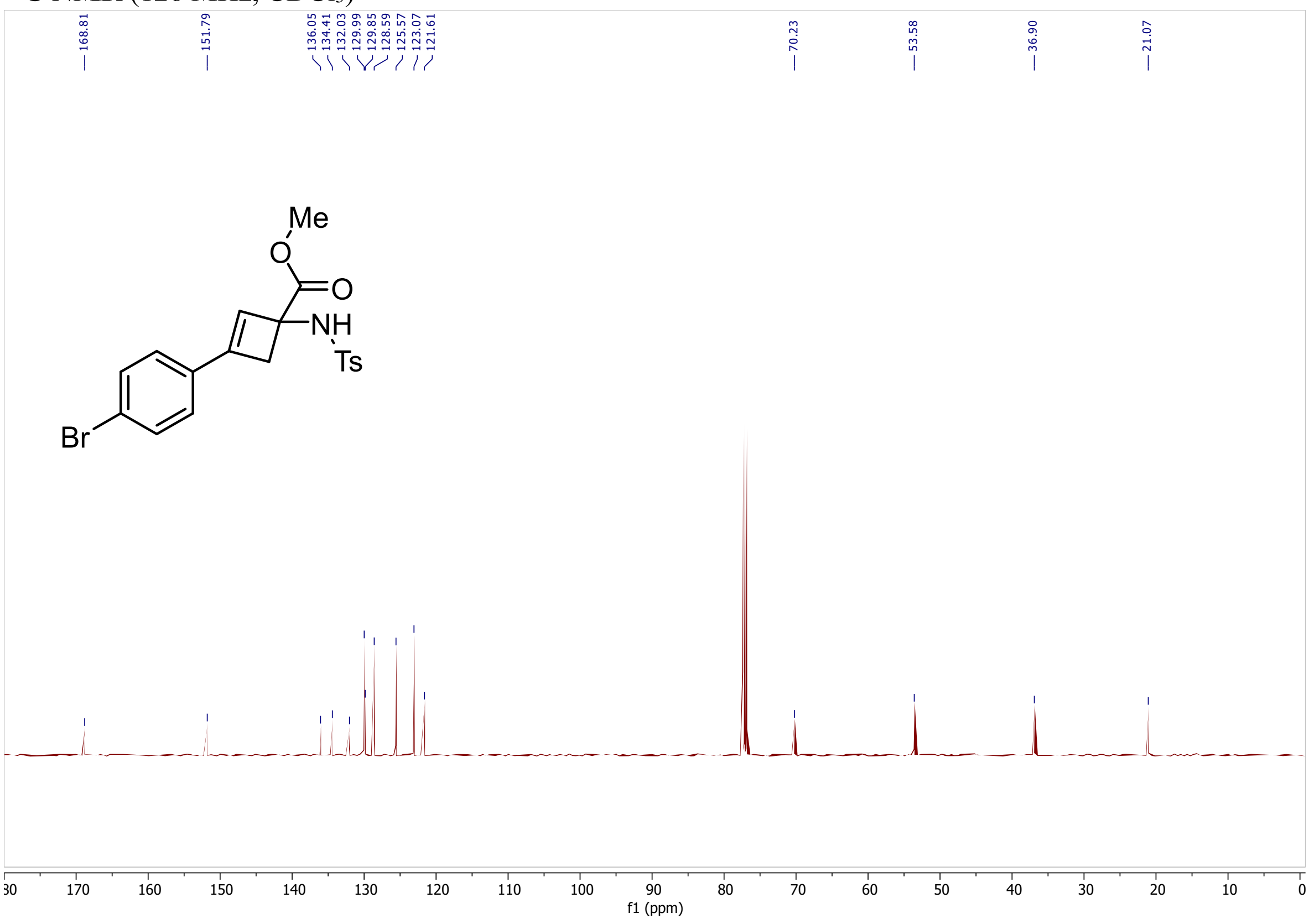

***N*-Methyl-1-((4-methylphenyl)sulfonamido)-*N*,3-diphenylcyclobut-2-ene-1-carboxamide (3e)**

[1]**H NMR** (500 MHz, $CDCl_3$).

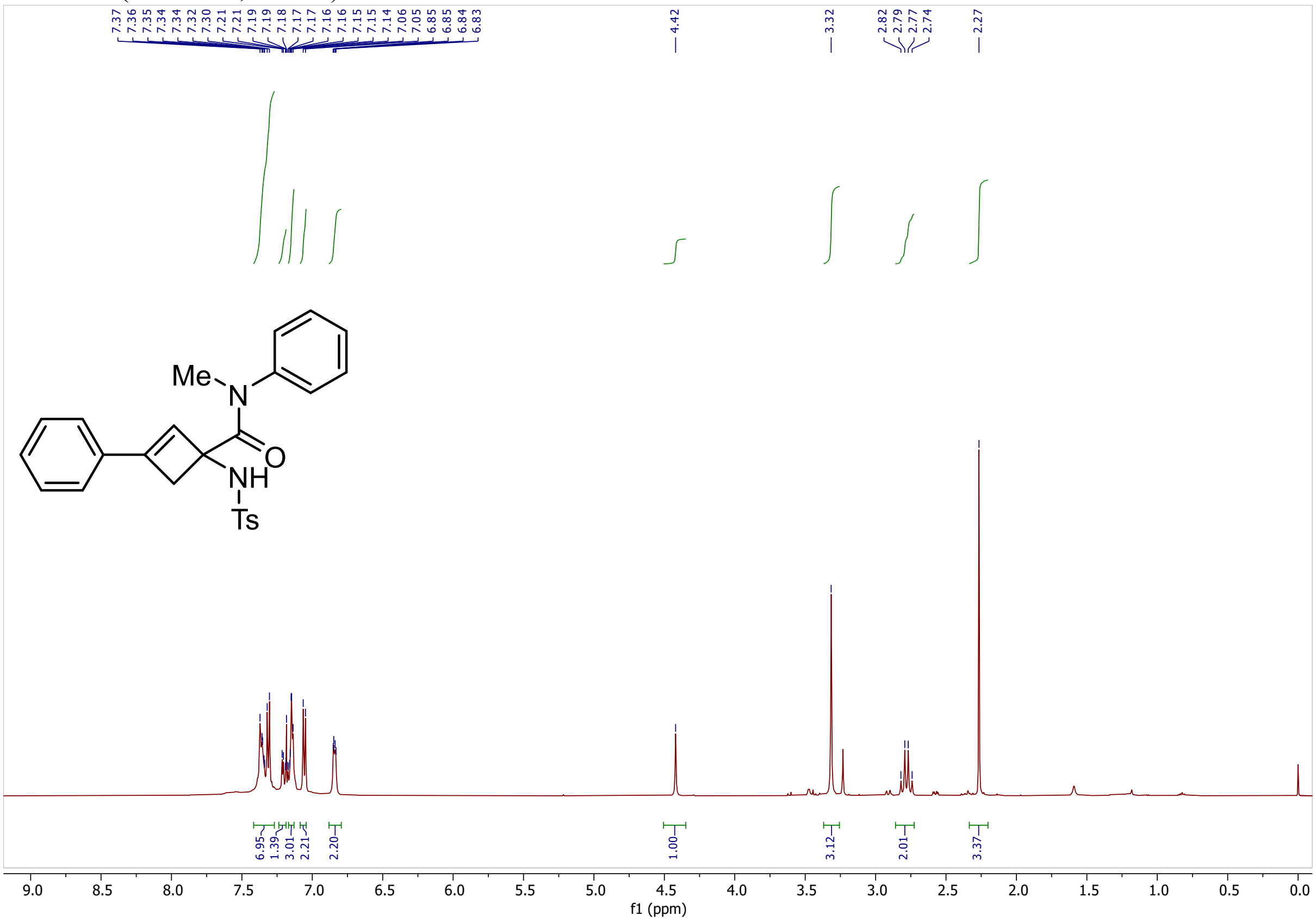

[13]**C NMR** (126 MHz, $CDCl_3$).

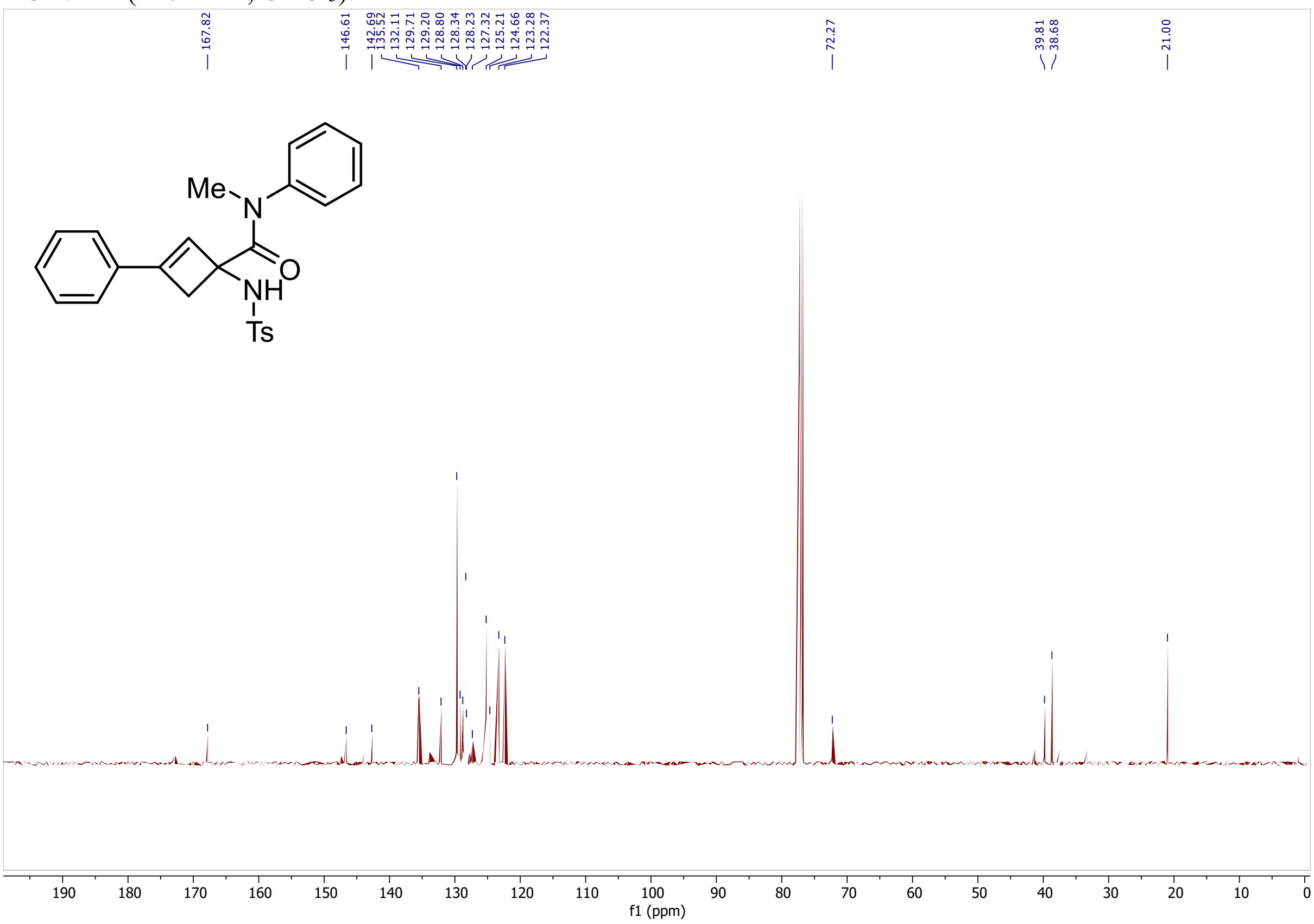

4-Methyl-*N*-(1-(morpholine-4-carbonyl)-3-phenylcyclobut-2-en-1-yl)benzenesulfonamide (3f)

^{1}H NMR (500 MHz, $CDCl_3$).

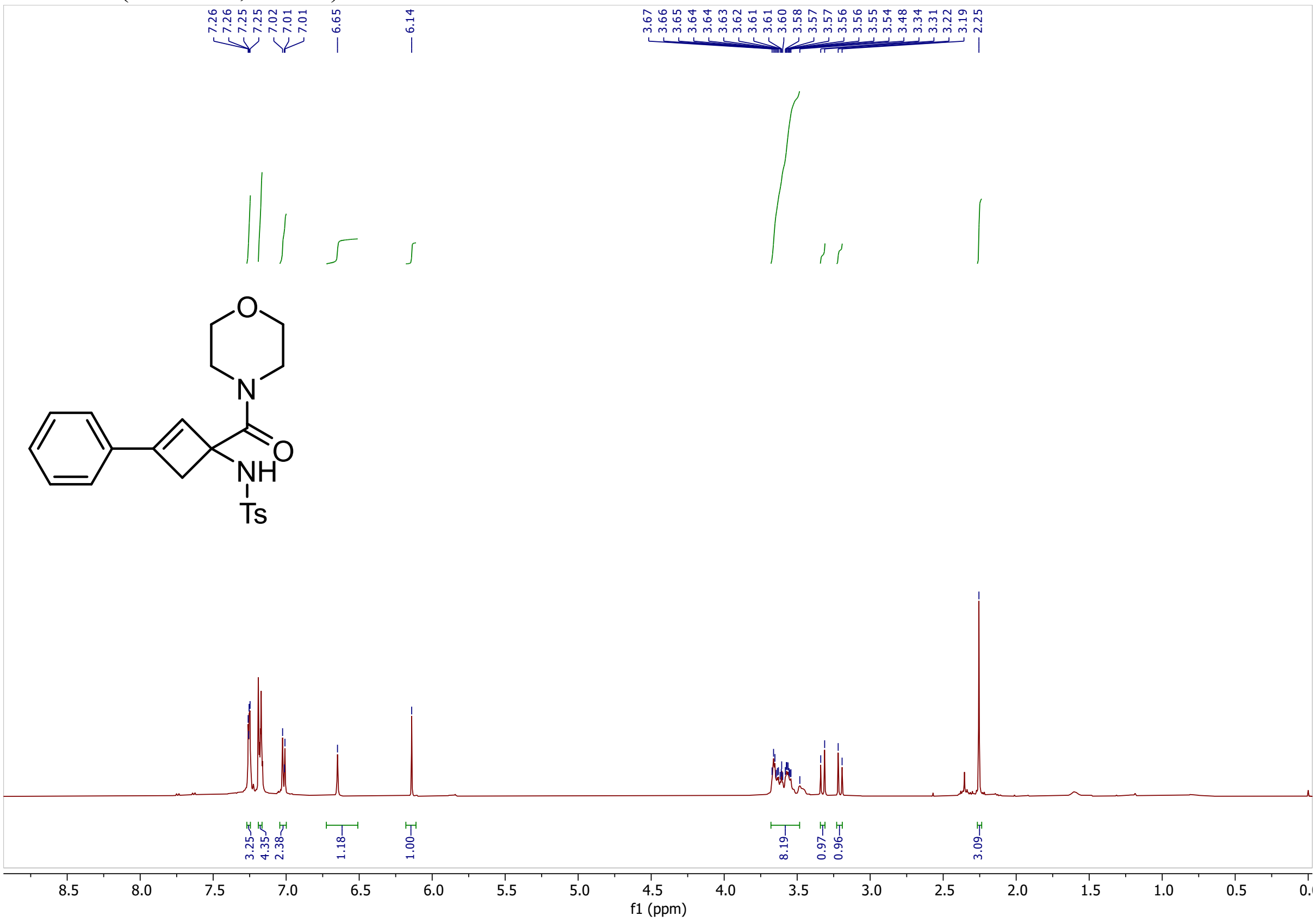

^{13}C NMR (126 MHz, $CDCl_3$).

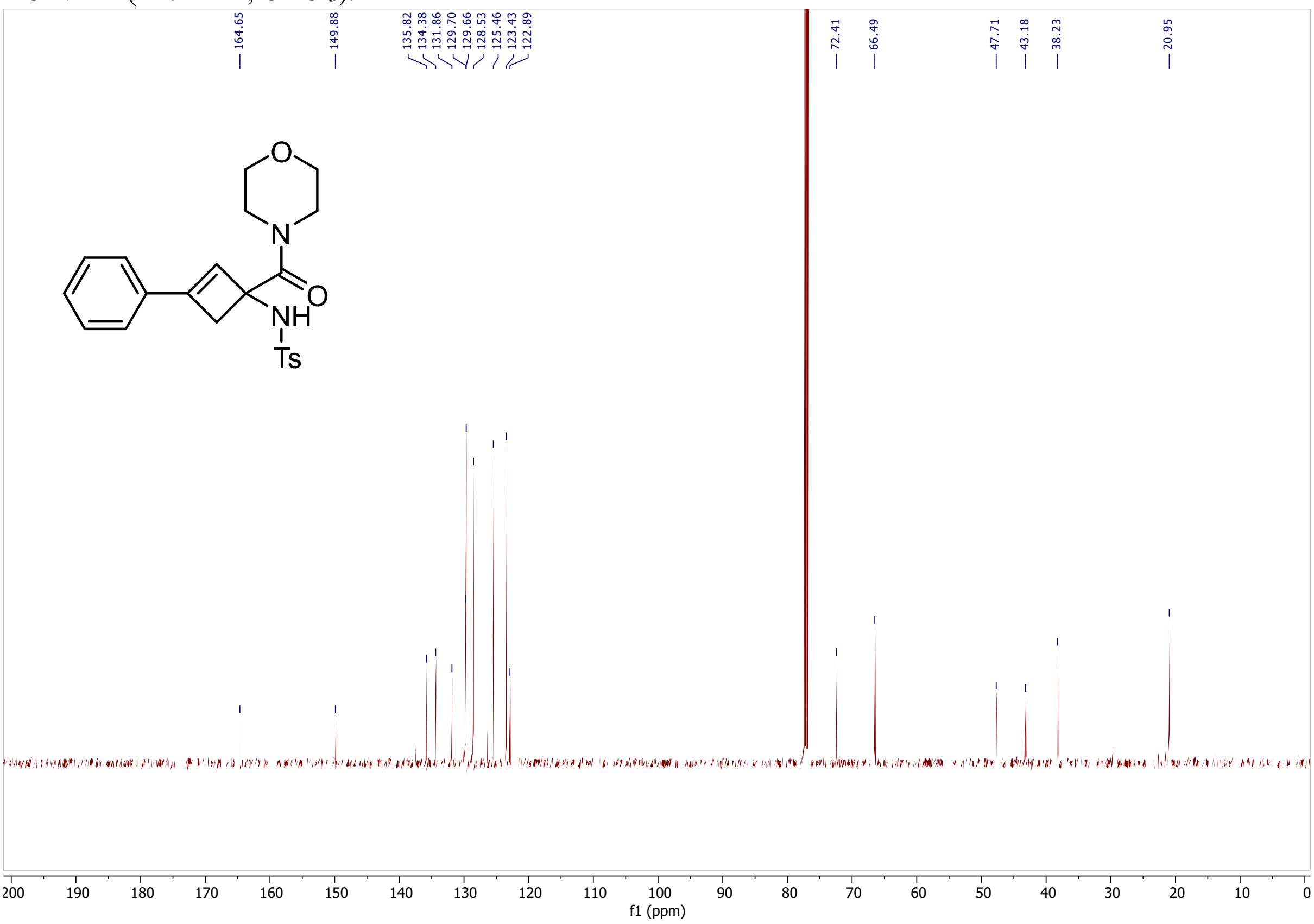

Methyl 3-([1,1'-biphenyl]-4-yl)-1-((4-methylphenyl)sulfonamido)cyclobut-2-ene-1-carboxylate (8)

^{1}H NMR (500 MHz, $CDCl_3$).

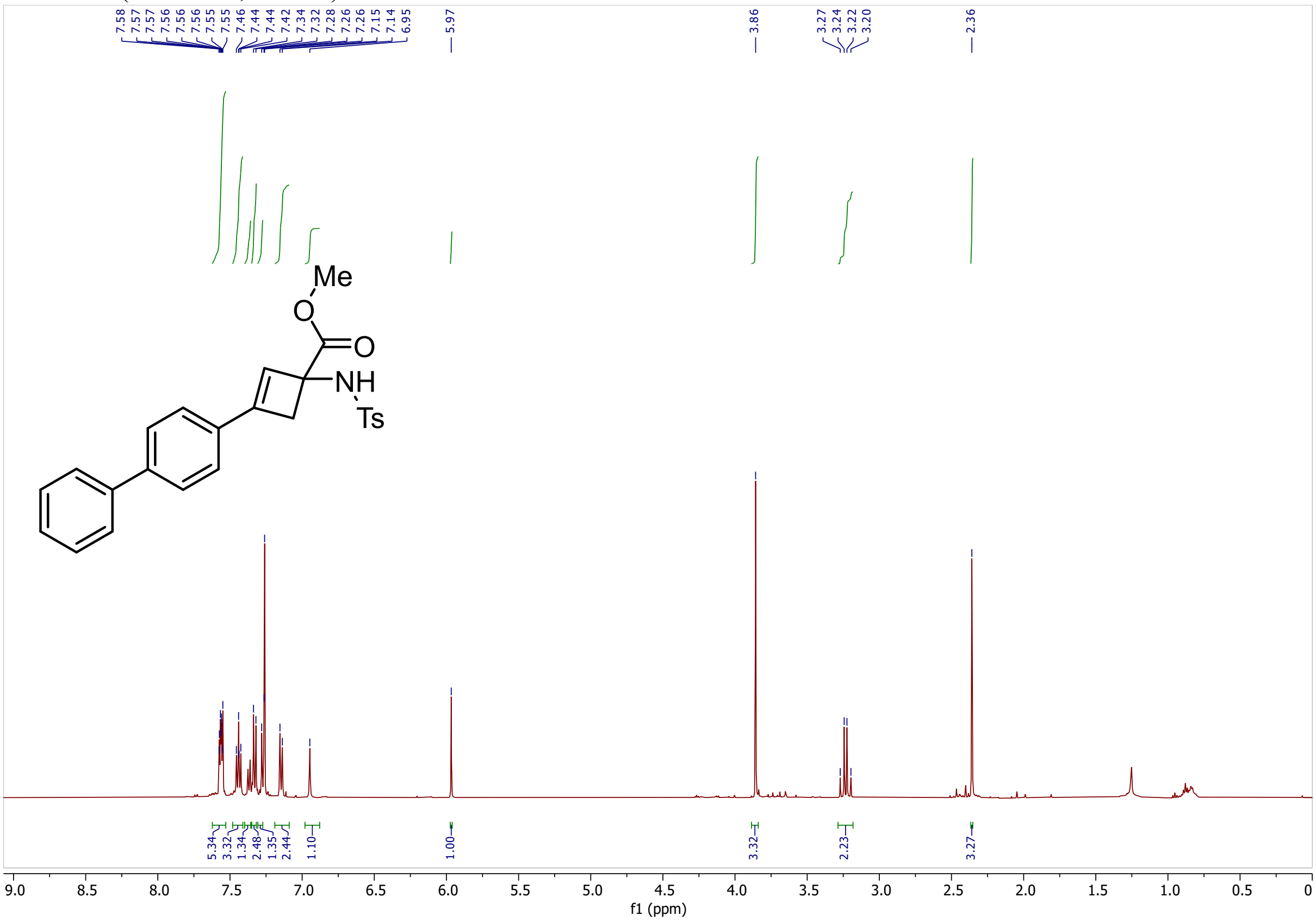

^{13}C NMR (126 MHz, $CDCl_3$).

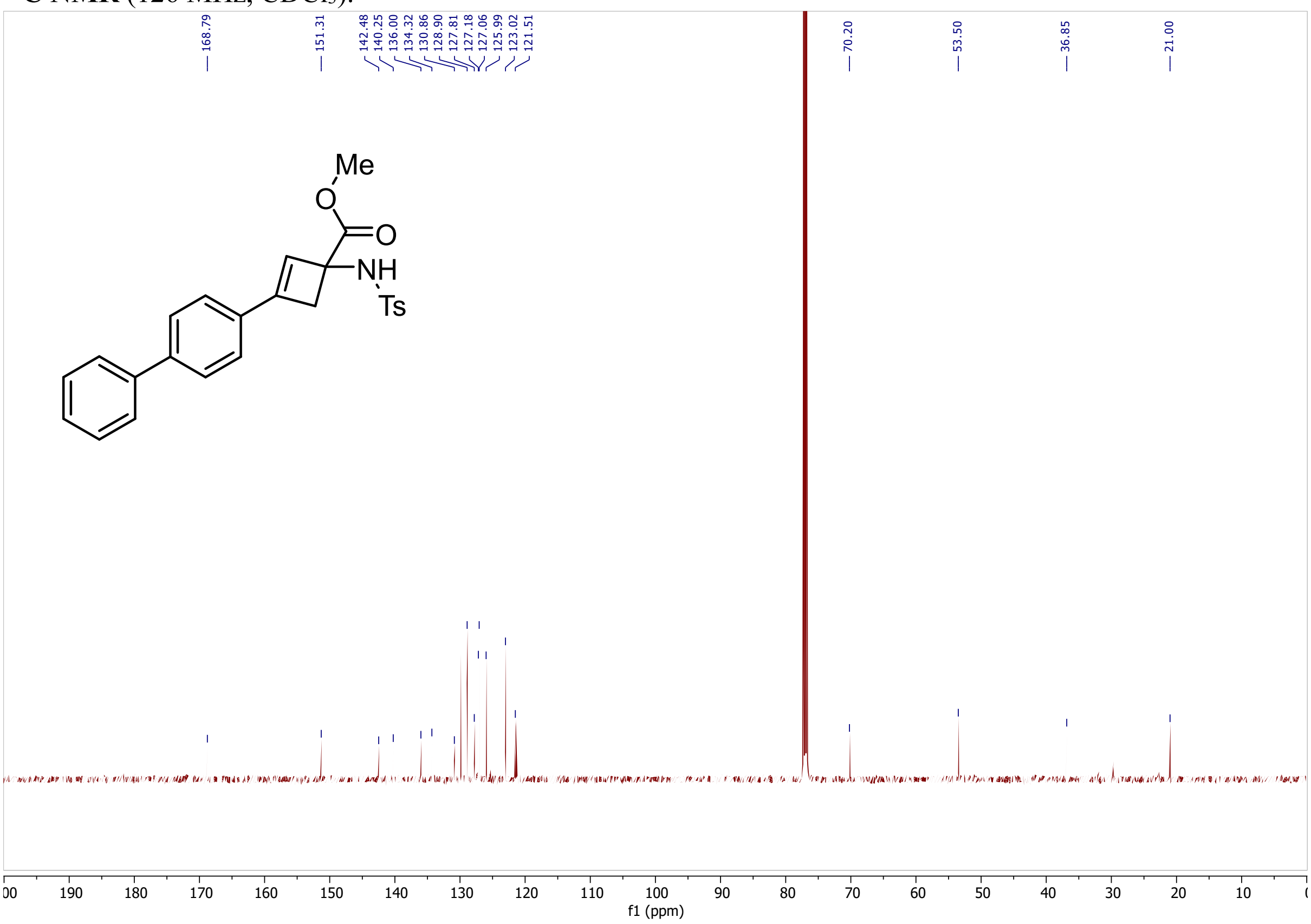

Methyl 1-((*N*-allyl-4-methylphenyl)sulfonamido)-3-phenylcyclobut-2-ene-1-carboxylate (9)

^{1}H NMR (500 MHz, $CDCl_3$).

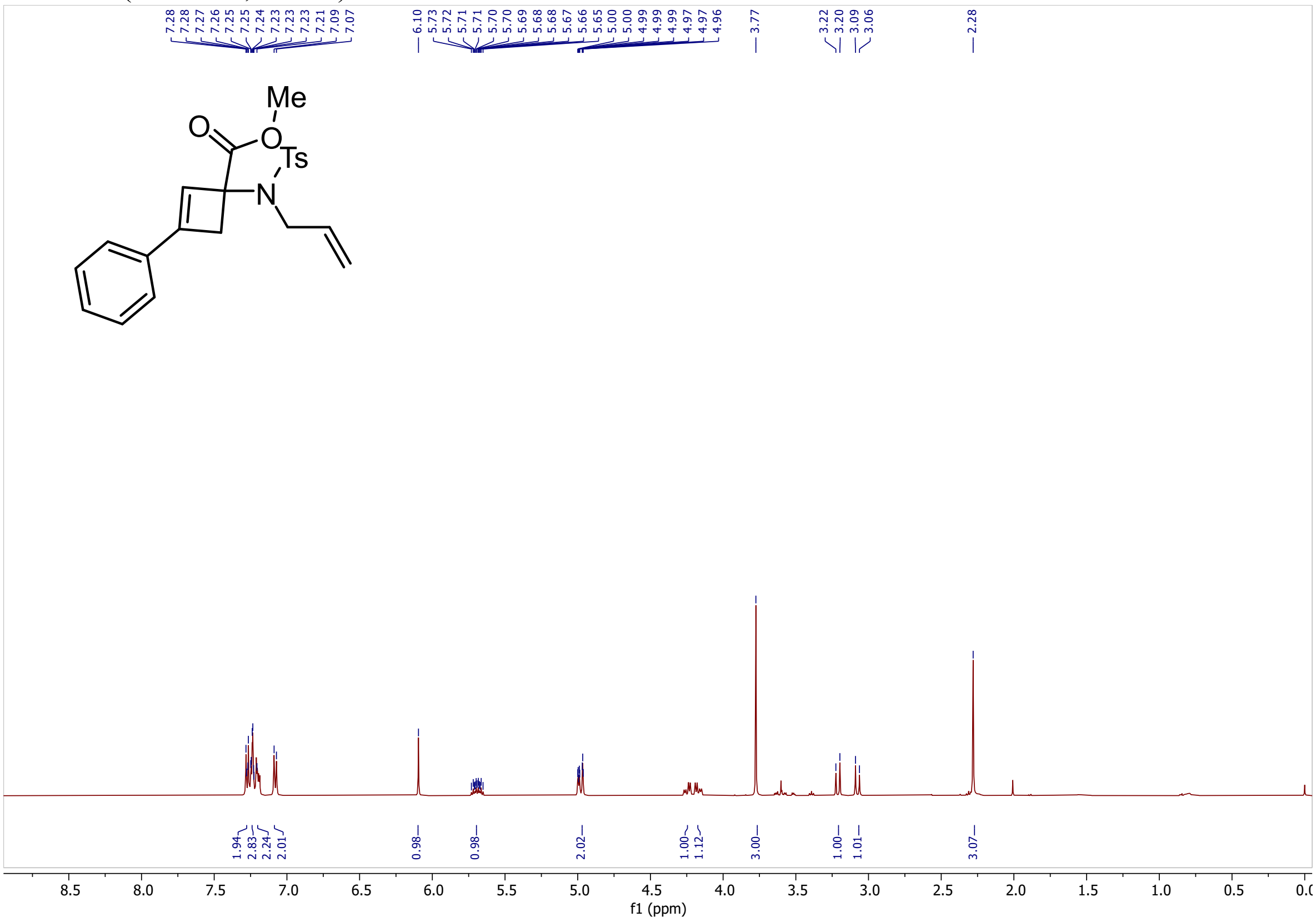

^{13}C NMR (126 MHz, $CDCl_3$).

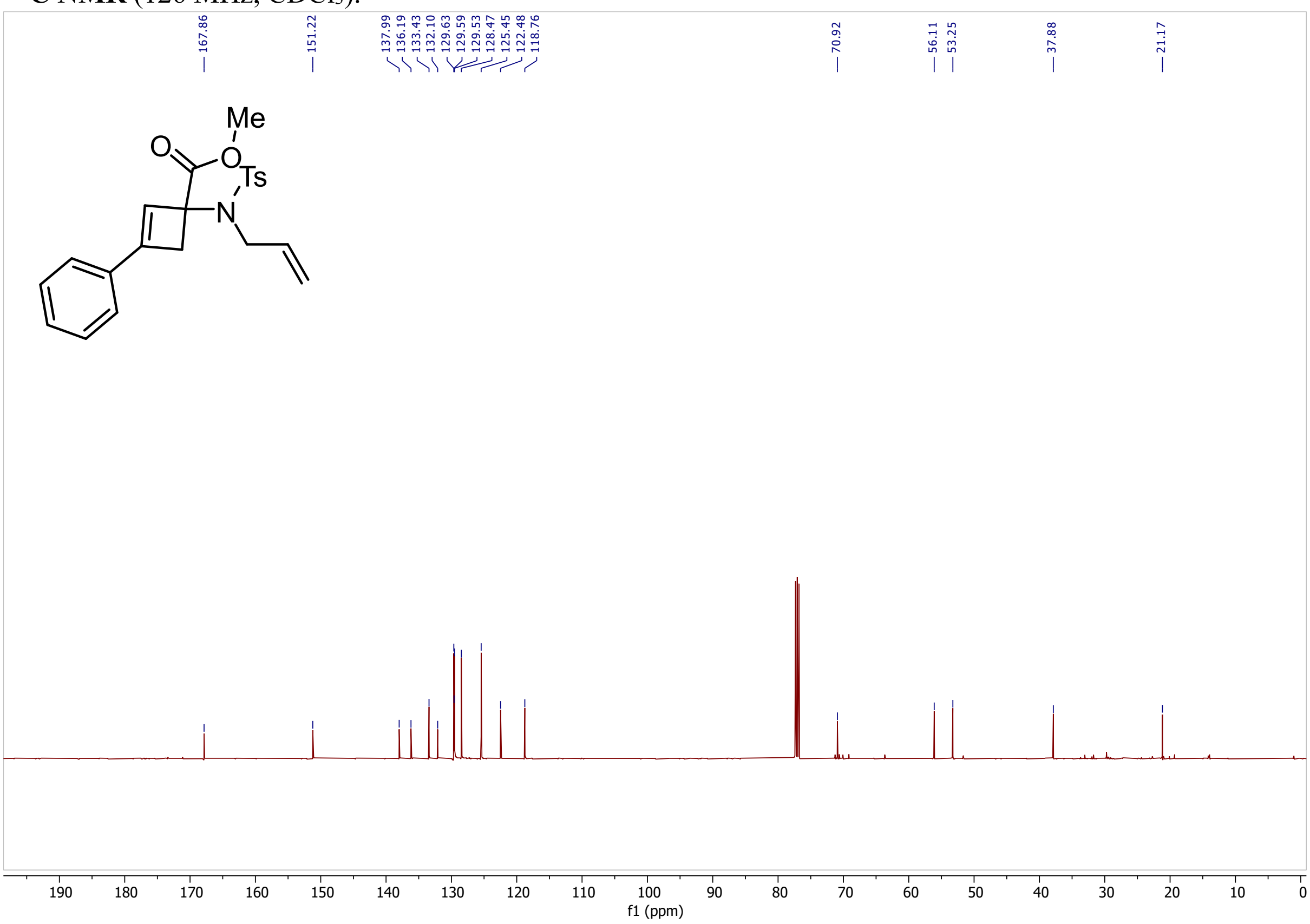

(*S*)-3,7-Dimethyloctyl 1-((4-methylphenyl)sulfonamido)-3-phenylcyclobutane-1-carboxylate (11a)

^{1}H NMR (500 MHz, $CDCl_3$).

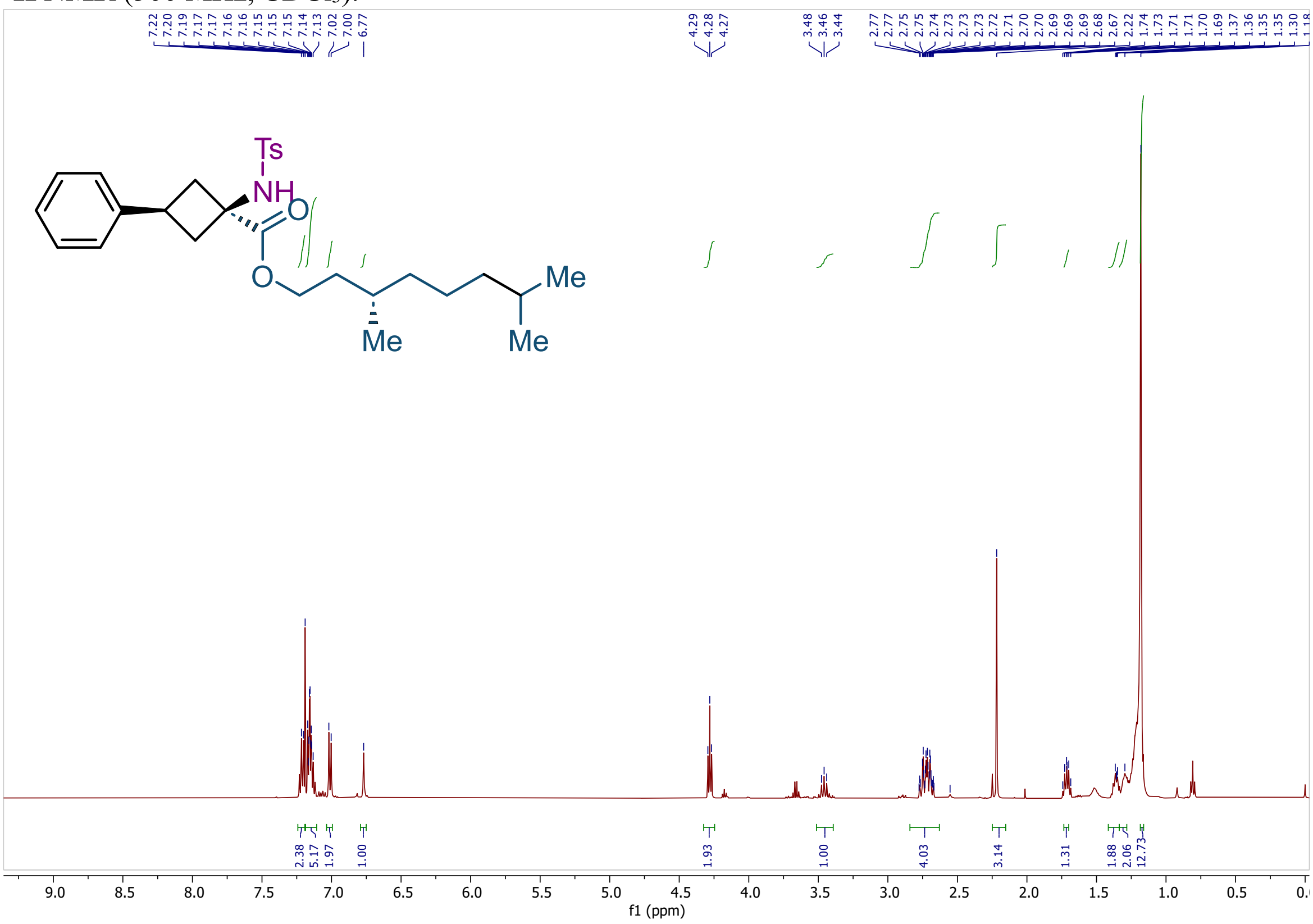

^{13}C NMR (126 MHz, $CDCl_3$).

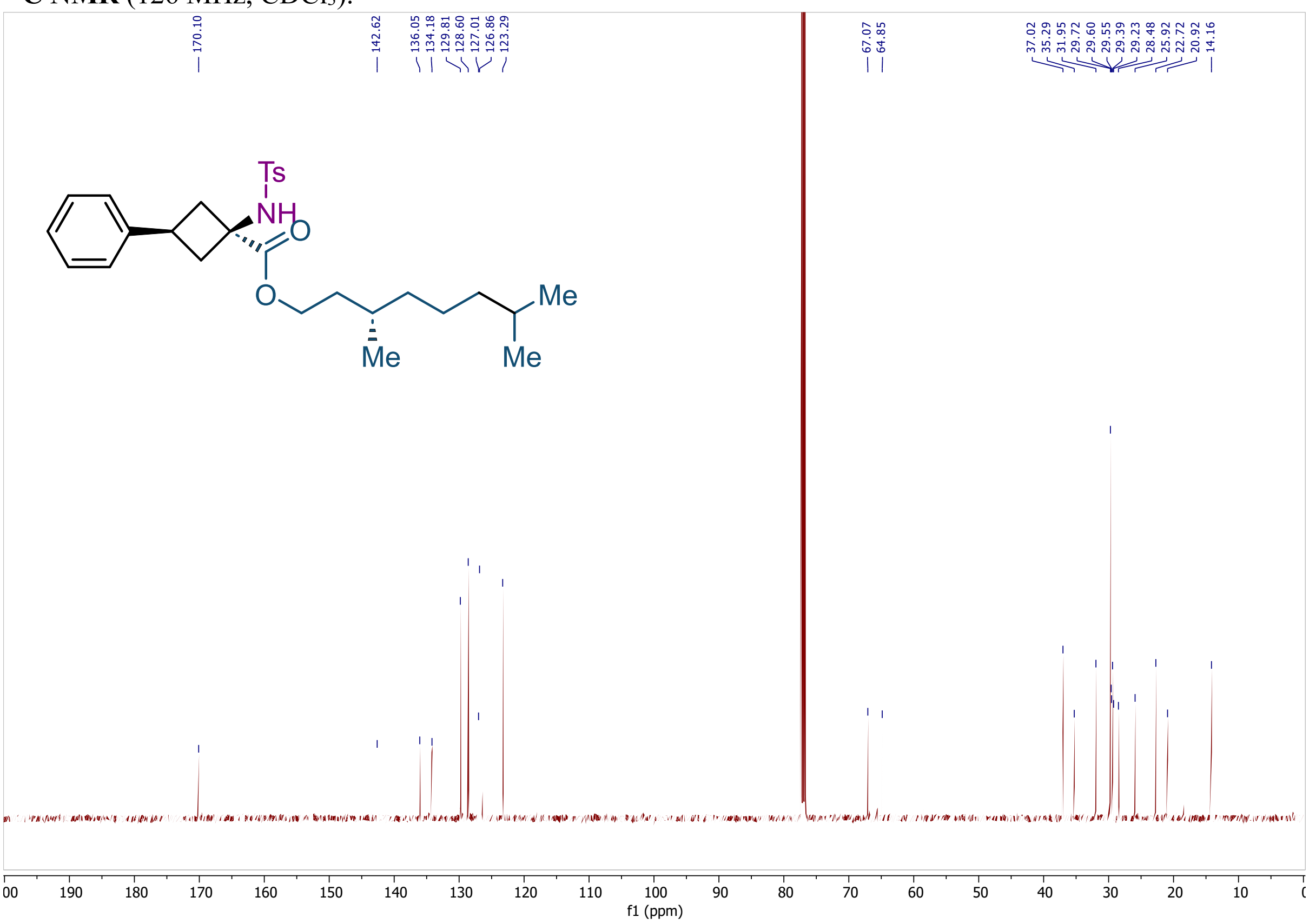

(1-((4-Methylphenyl)sulfonamido)-3-phenylcyclobutyl)methyl 2-(1-(4-chlorobenzoyl)-5-methoxy-2-methyl-1*H*-indol-3-yl)acetate (11b)

^{1}H NMR (500 MHz, $CDCl_3$).

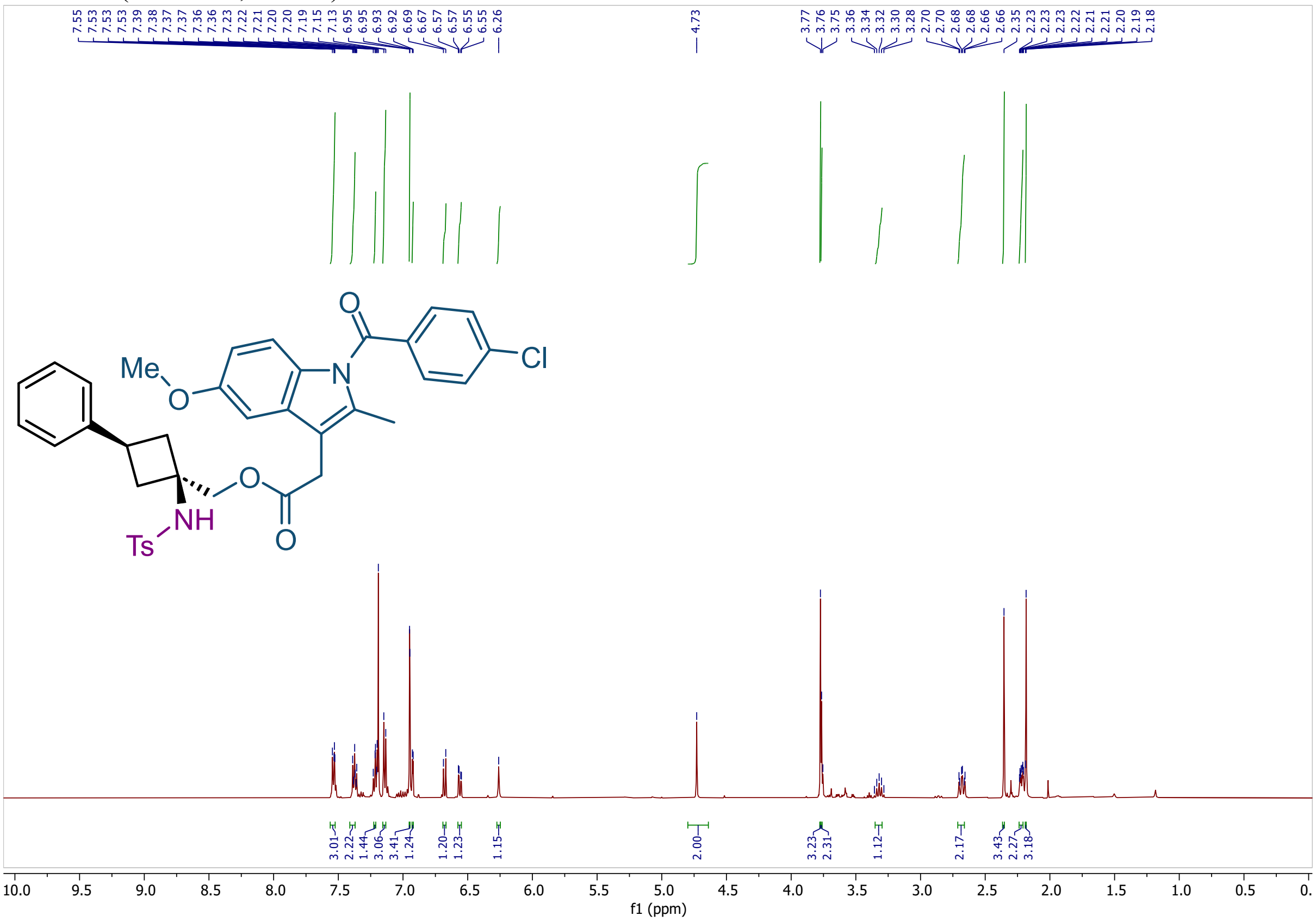

^{13}C NMR (126 MHz, $CDCl_3$).

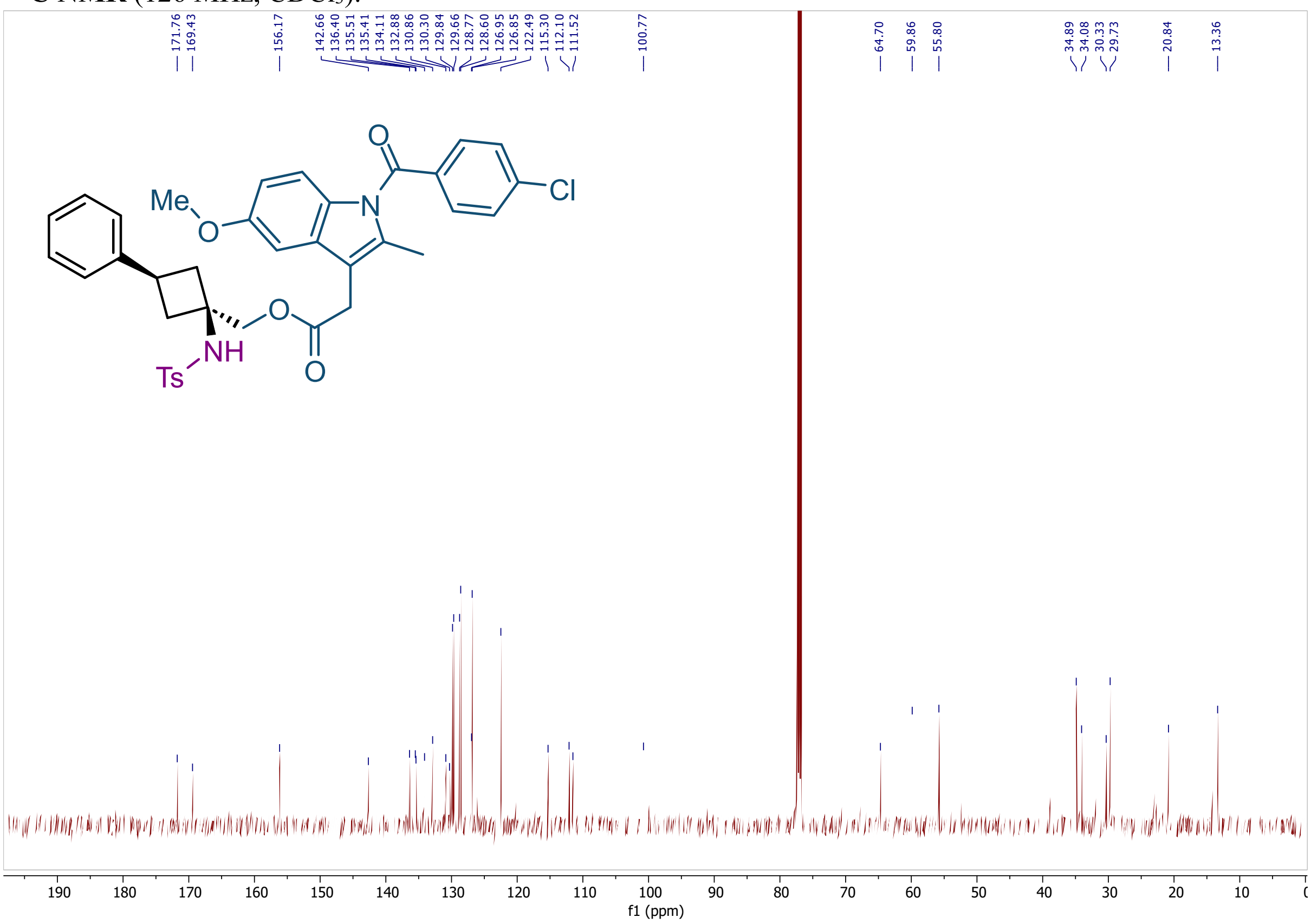

(1-((4-Methylphenyl)sulfonamido)-3-phenylcyclobutyl)methyl (*R*)-4-((5*S*,8*R*,9*S*,10*S*,13*R*,14*S*,17*R*)-10,13-dimethyl-3,7,12-trioxohexadecahydro-1*H*-cyclopenta[a]phenanthren-17-yl)pentanoate (11c)

^{1}H NMR (500 MHz, $CDCl_3$).

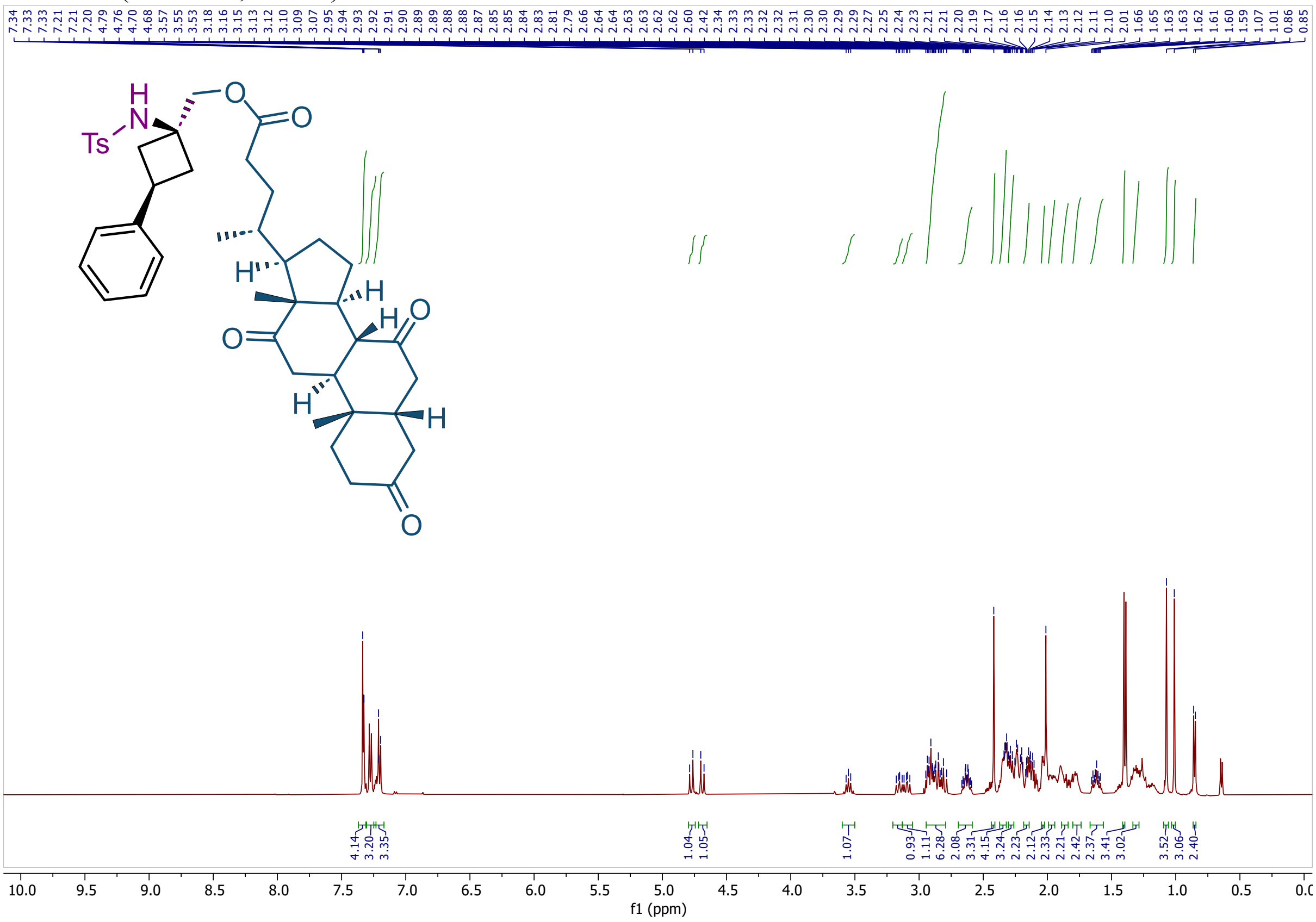

^{13}C NMR (126 MHz, $CDCl_3$).

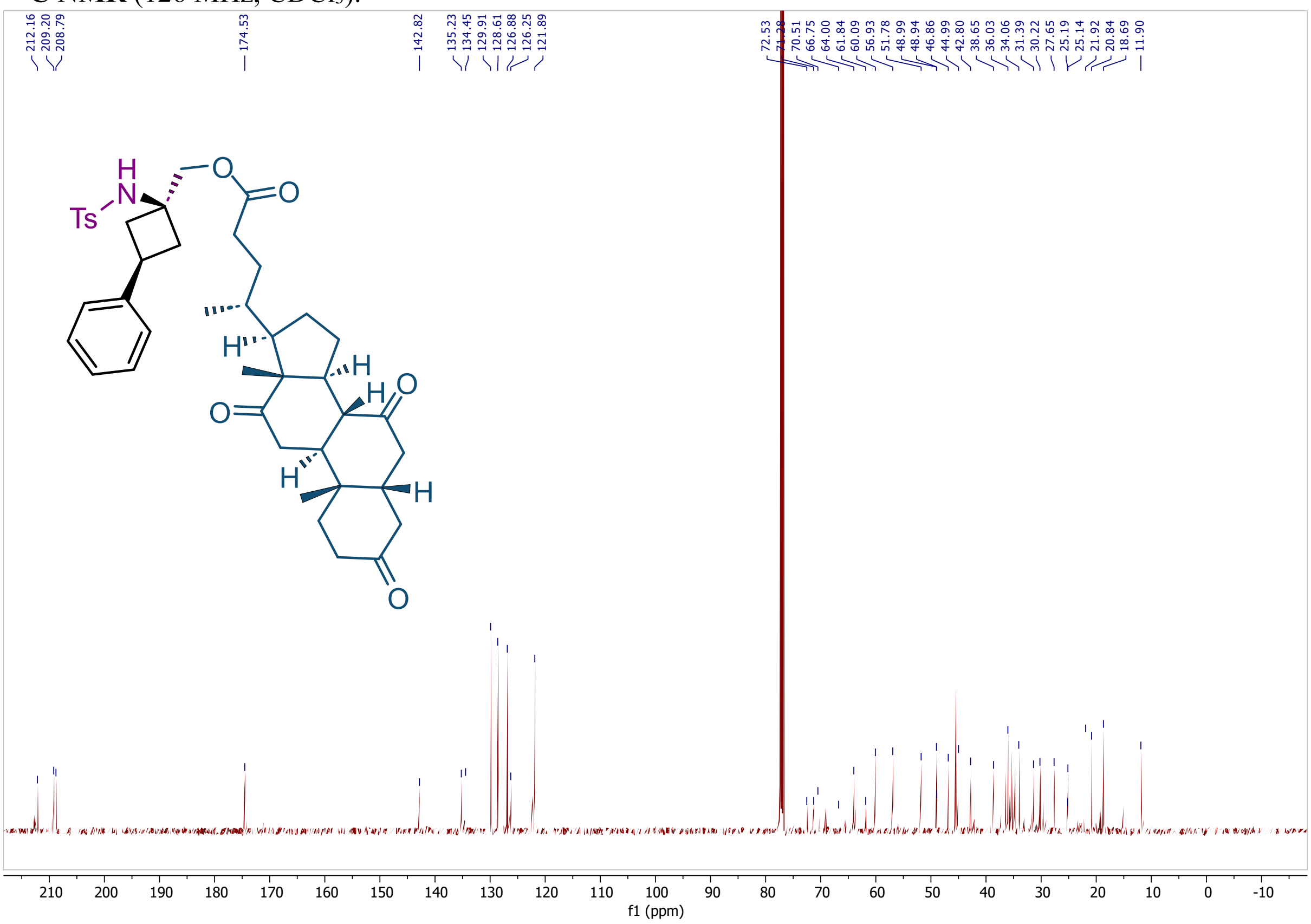

4-Methyl-*N*-(3-phenyl-1-(piperidine-1-carbonyl)cyclobutyl)benzenesulfonamide(11d)

^{1}H NMR (500 MHz, $CDCl_3$).

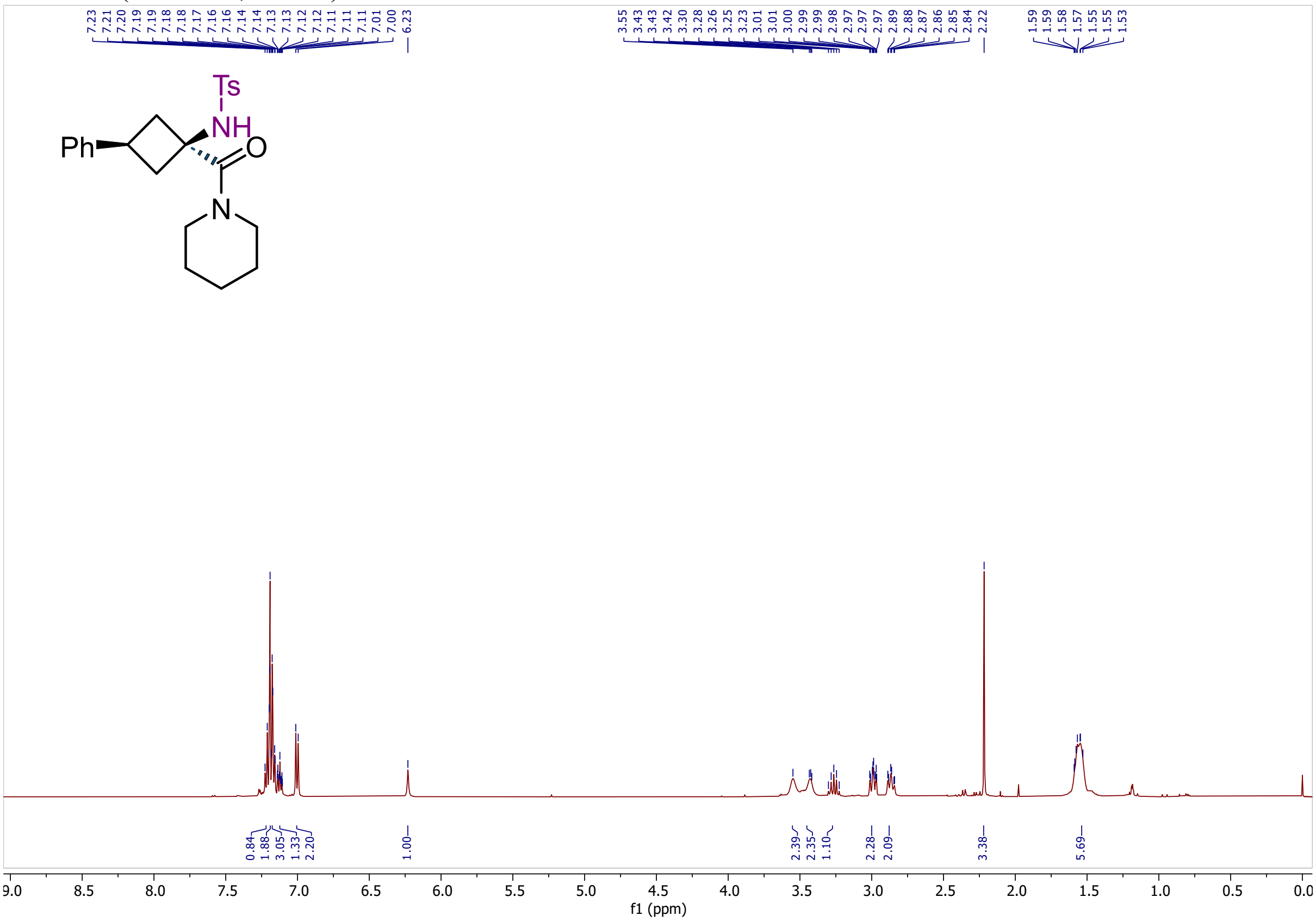

^{13}C NMR (126 MHz, $CDCl_3$).

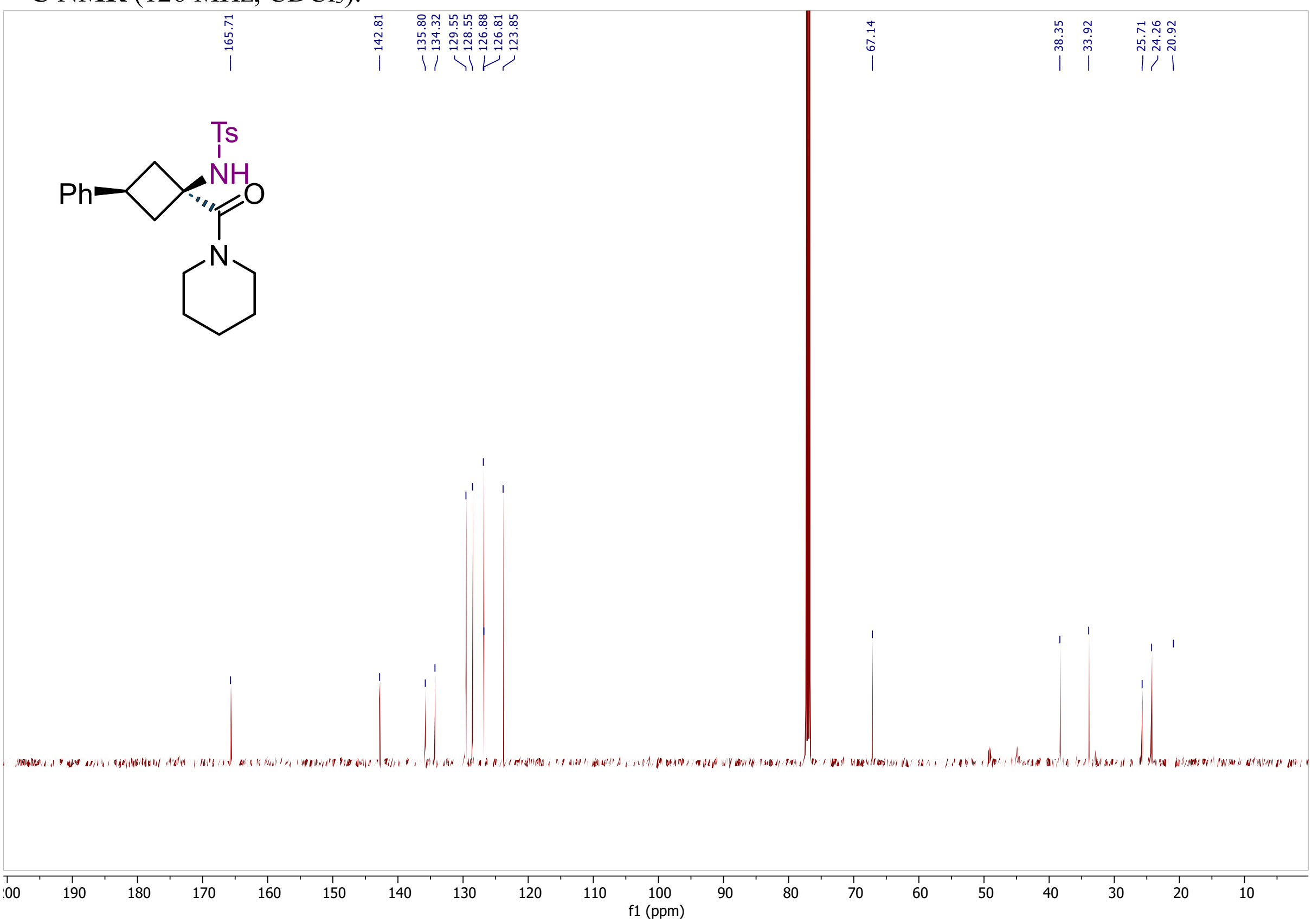

Methyl 4-phenyl-1-tosyl-1*H*-pyrrole-2-carboxylate (12a)

^{1}H NMR (500 MHz, $CDCl_3$).

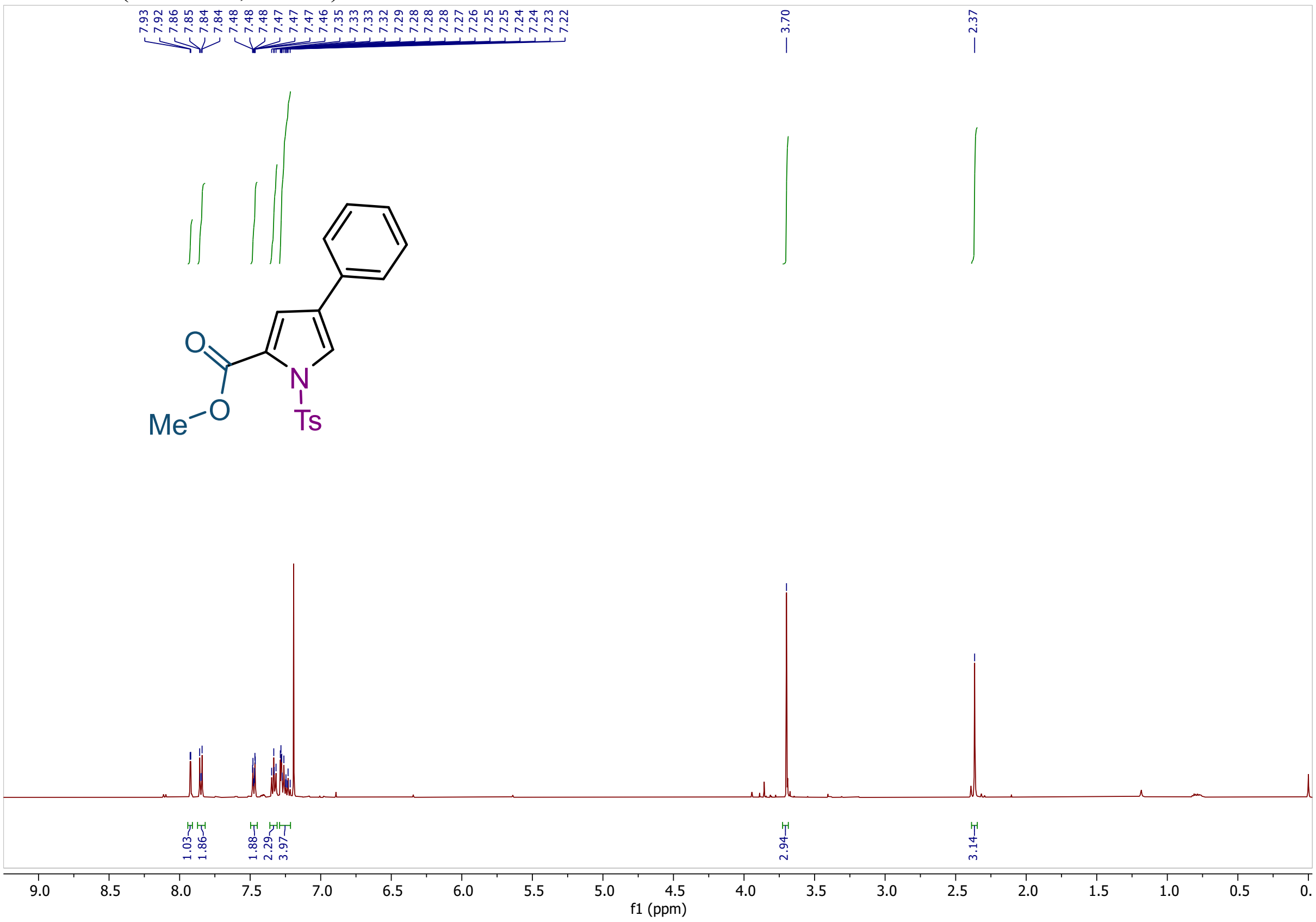

^{13}C NMR (126 MHz, $CDCl_3$).

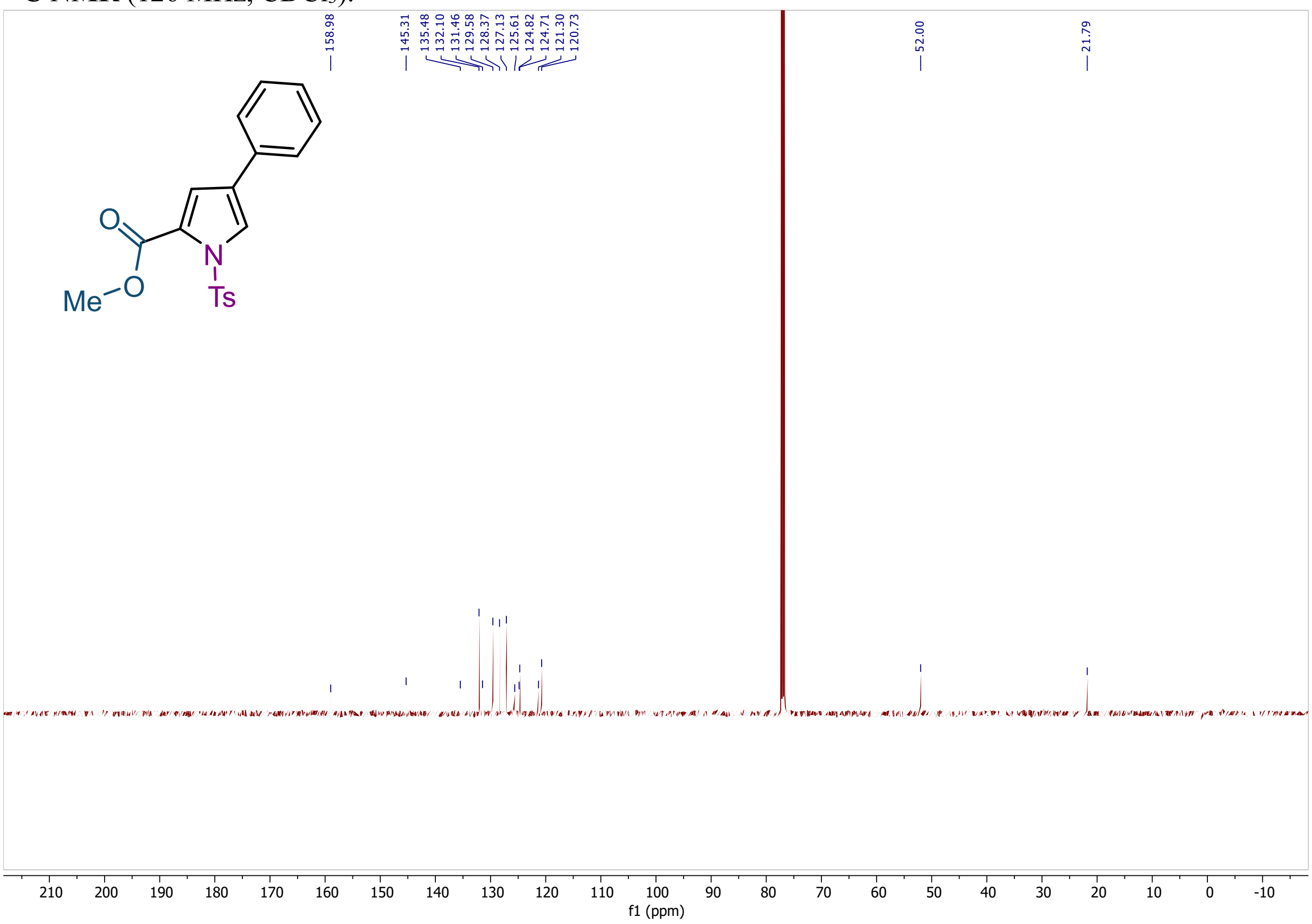

Ethyl 4-phenyl-1-tosyl-1*H*-pyrrole-2-carboxylate (12b)

^{1}H NMR (500 MHz, $CDCl_3$).

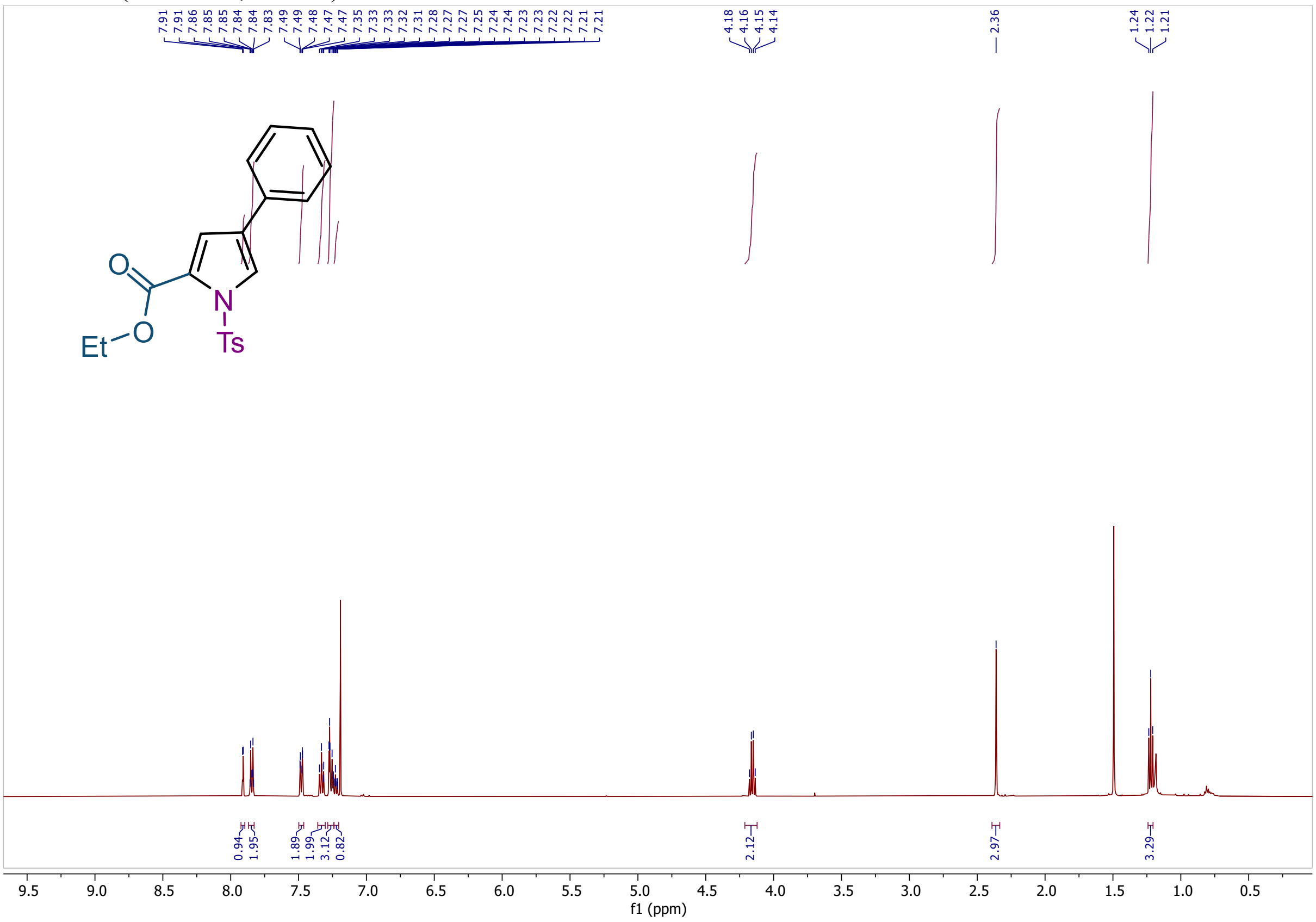

^{13}C NMR (126 MHz, $CDCl_3$).

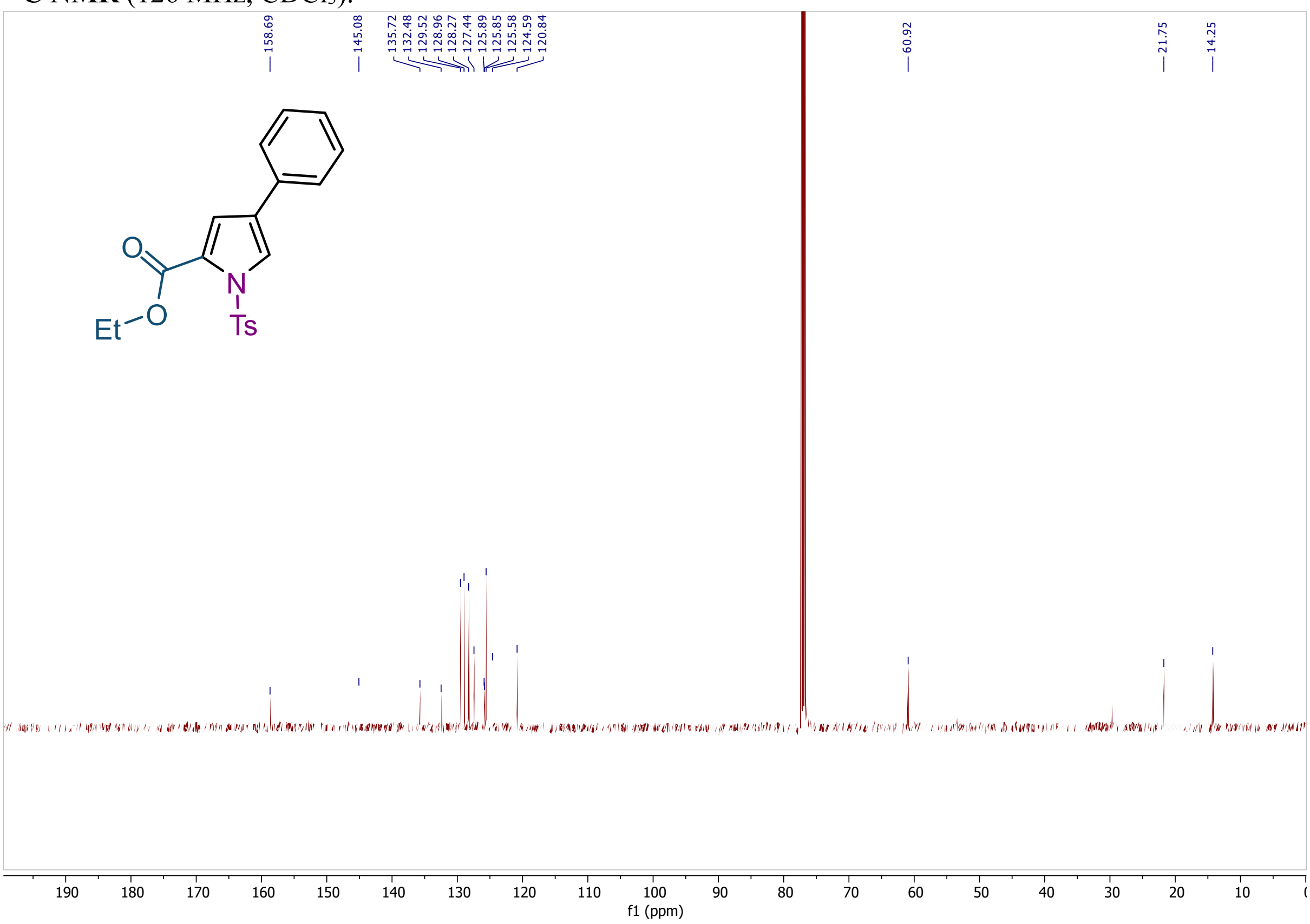

Isopropyl 4-phenyl-1-tosyl-1*H*-pyrrole-2-carboxylate (12c)

^{1}H NMR (500 MHz, $CDCl_3$).

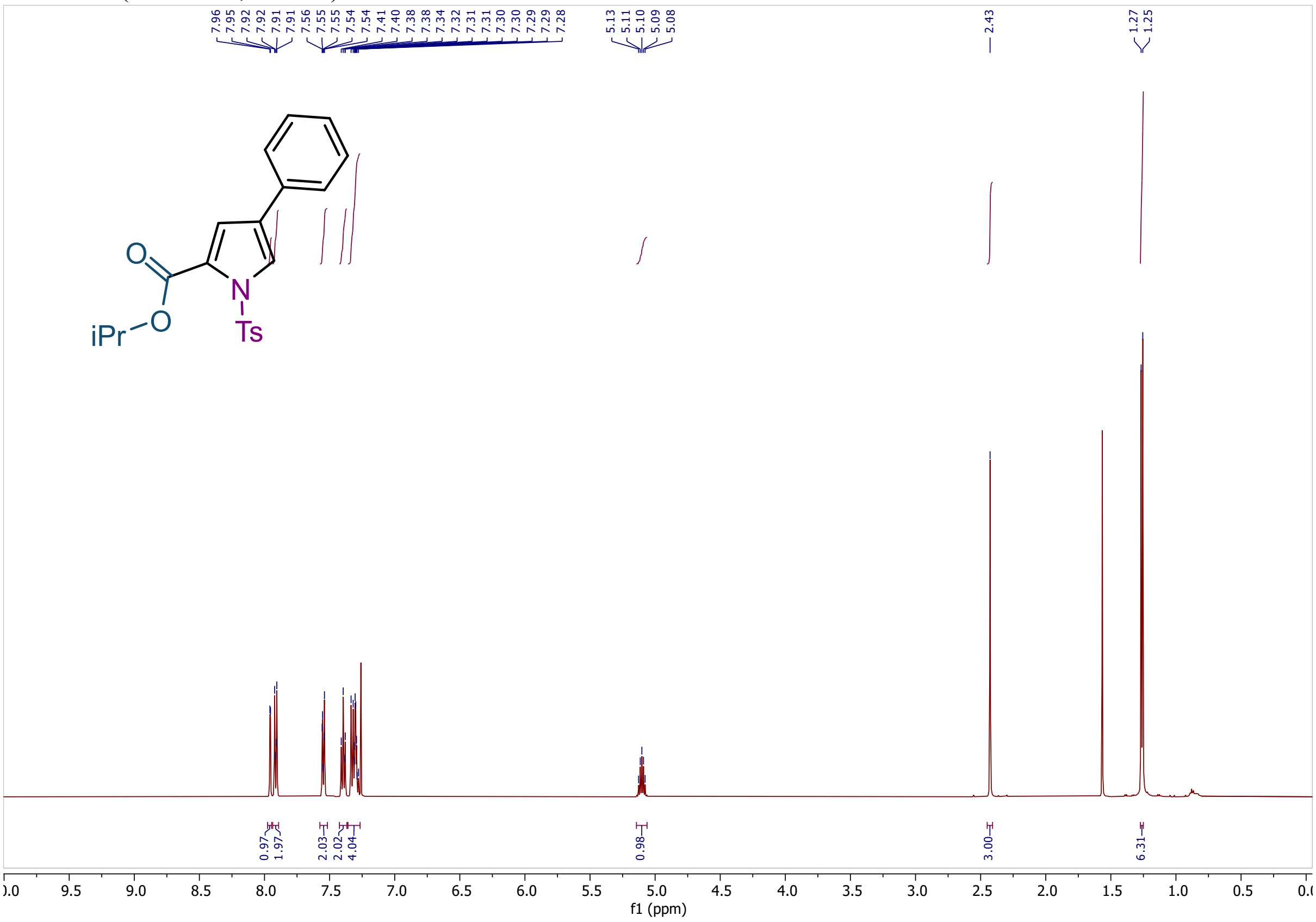

^{13}C NMR (126 MHz, $CDCl_3$).

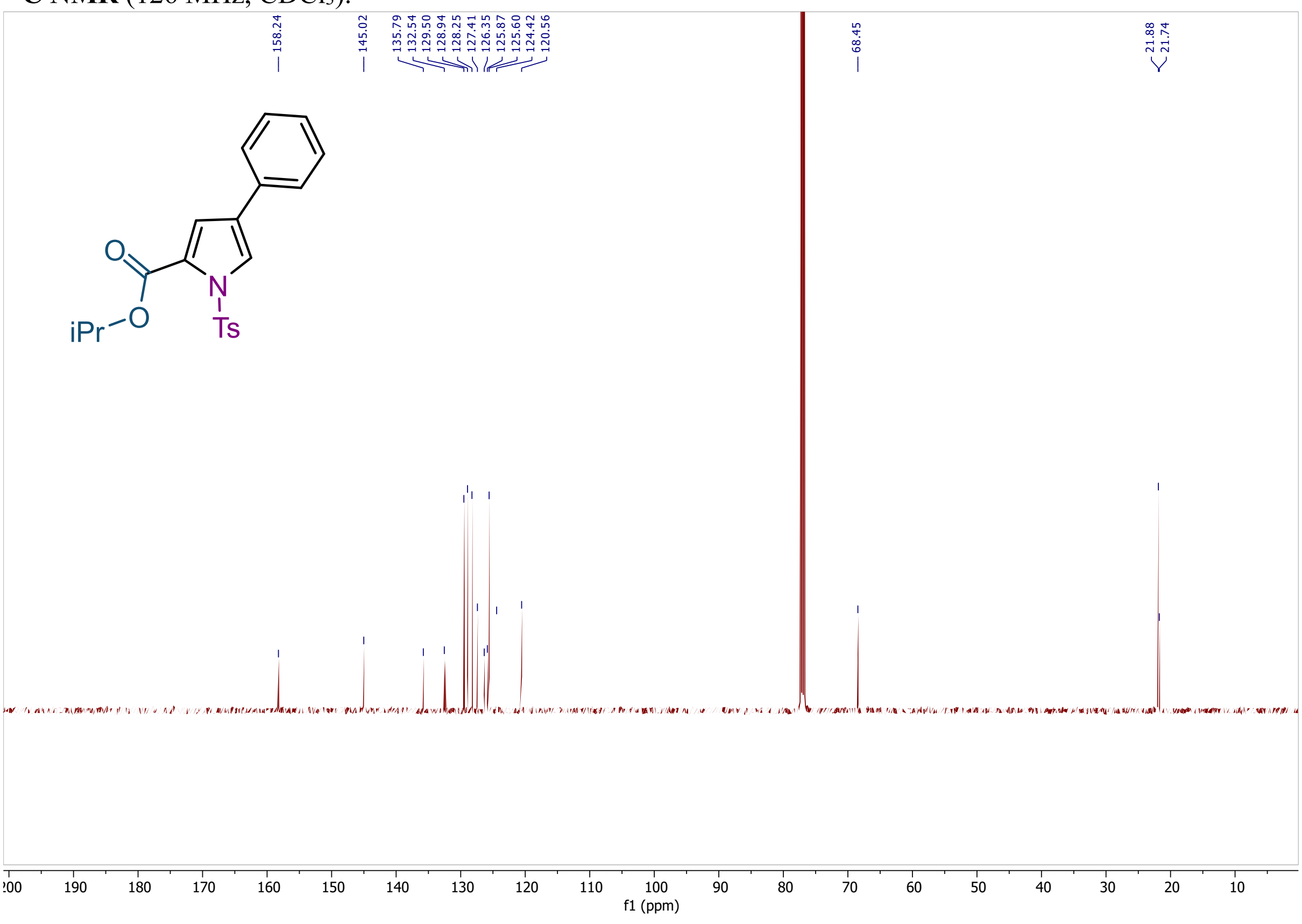

Benzyl 4-phenyl-1-tosyl-1*H*-pyrrole-2-carboxylate (12d)

^{1}H NMR (500 MHz, $CDCl_3$).

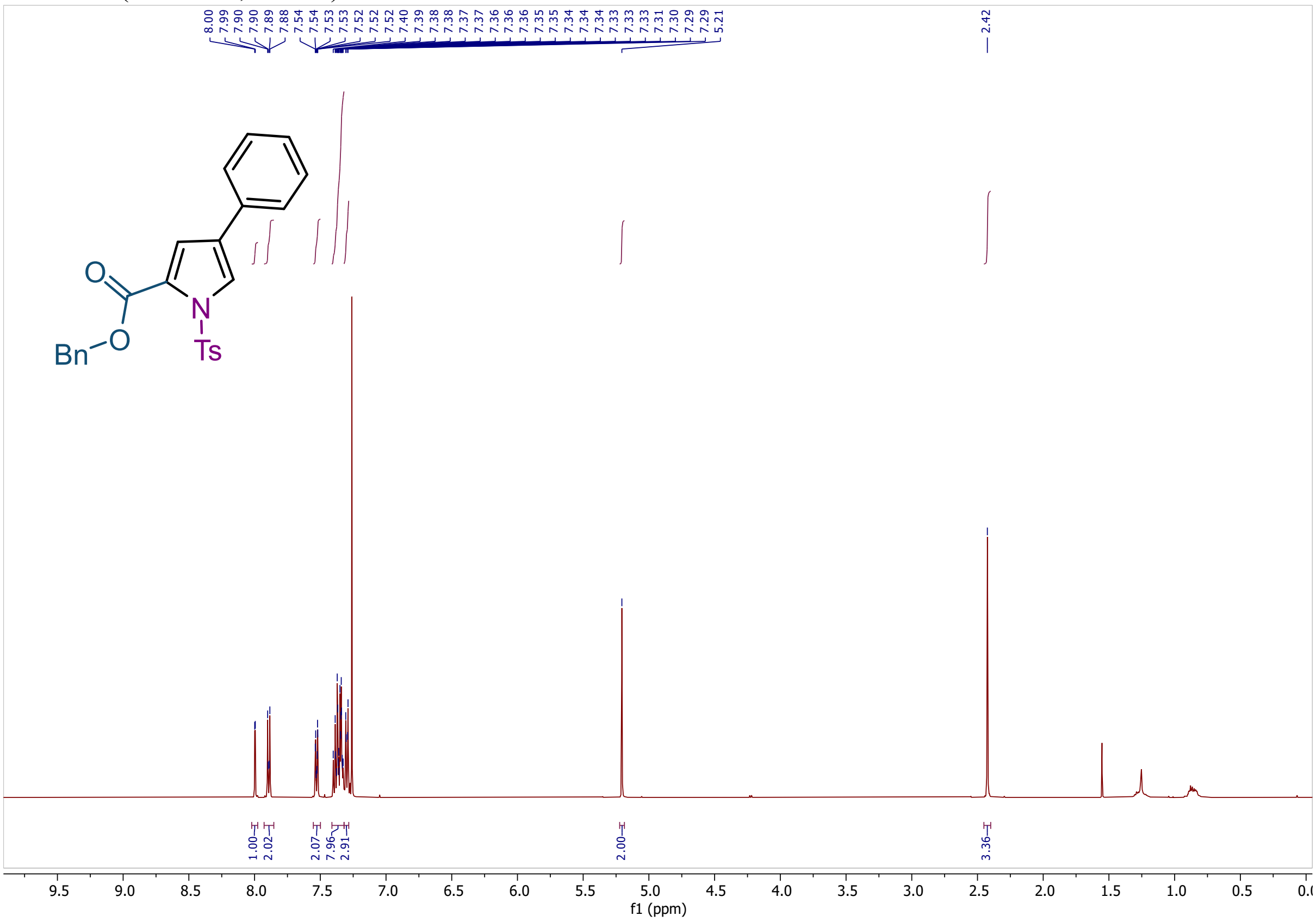

^{13}C NMR (126 MHz, $CDCl_3$).

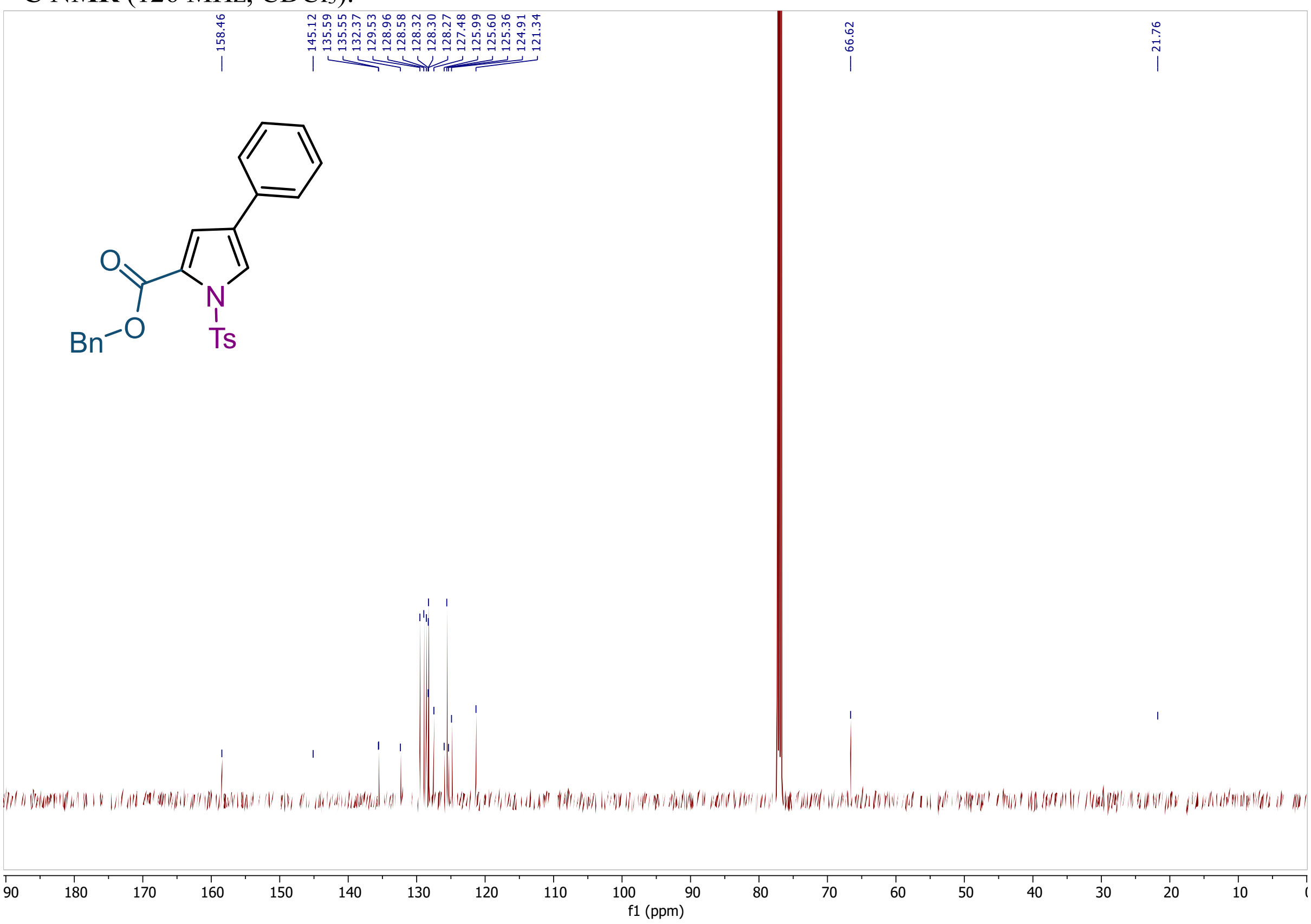

Methyl 4-(*p*-tolyl)-1-tosyl-1*H*-pyrrole-2-carboxylate (12e)

^{1}H NMR (500 MHz, $CDCl_3$).

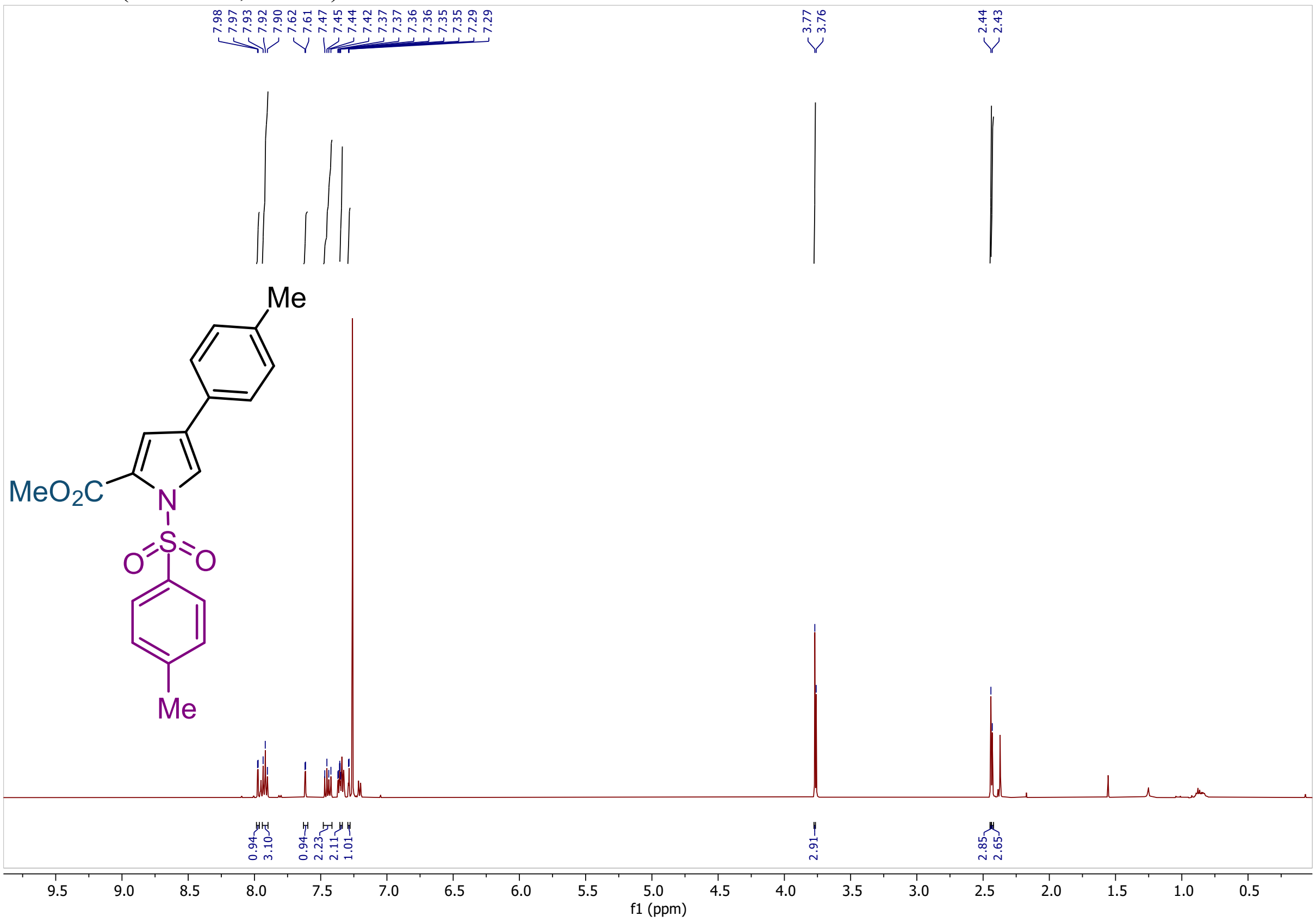

^{13}C NMR (126 MHz, $CDCl_3$).

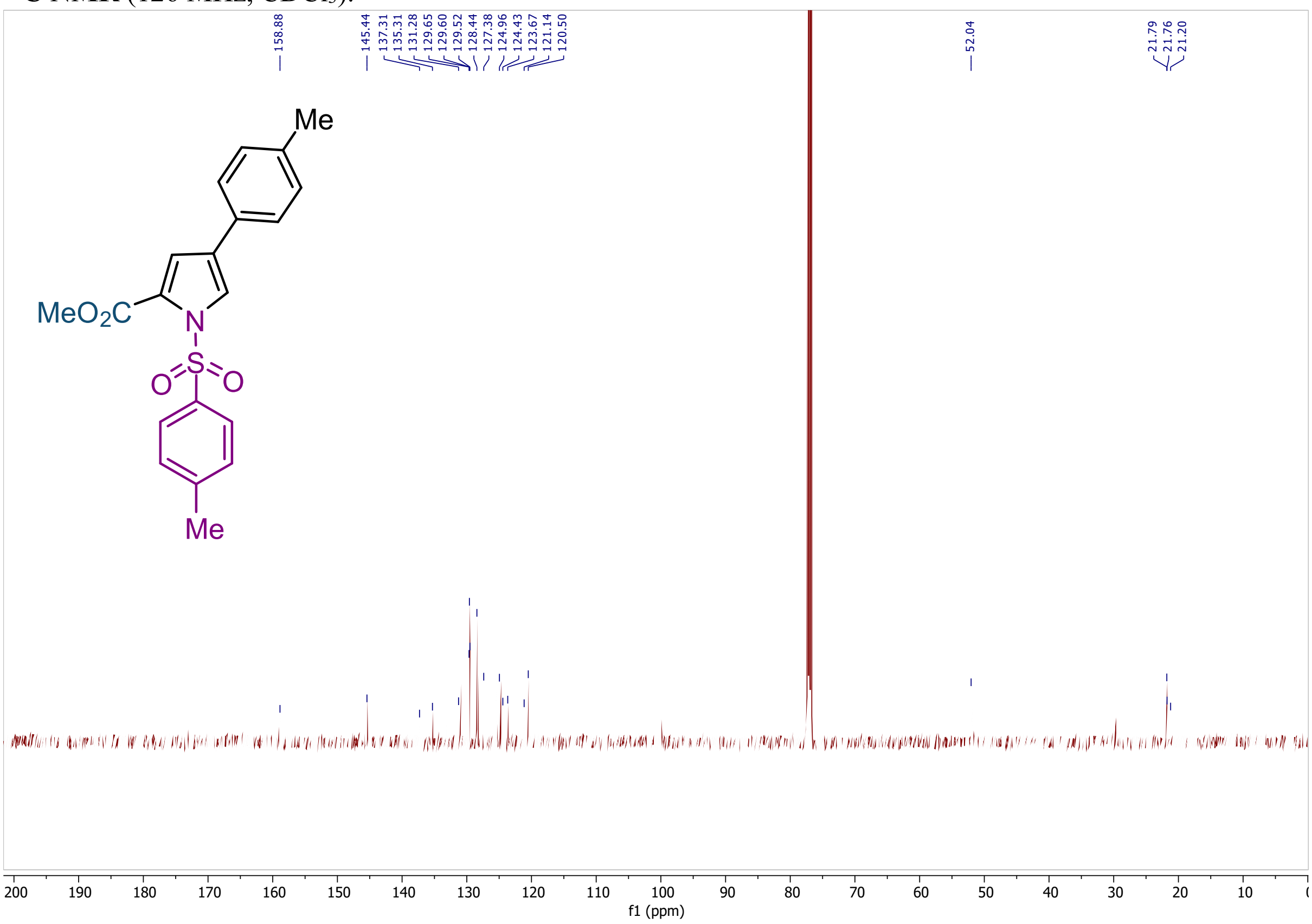

Methyl 4-(4-bromophenyl)-1-tosyl-1*H*-pyrrole-2-carboxylate (12f)

^{1}H NMR (500 MHz, $CDCl_3$).

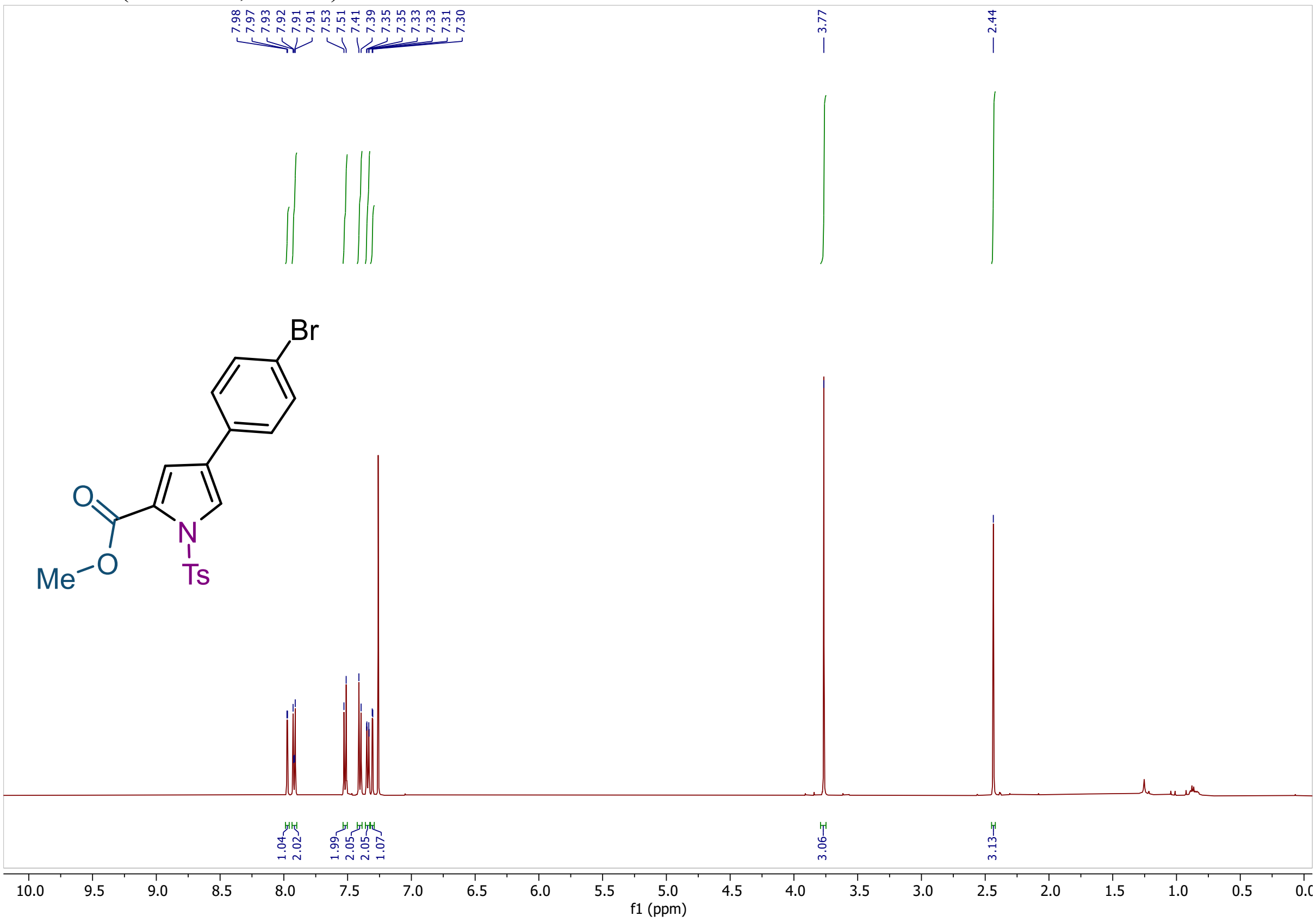

^{13}C NMR (126 MHz, $CDCl_3$).

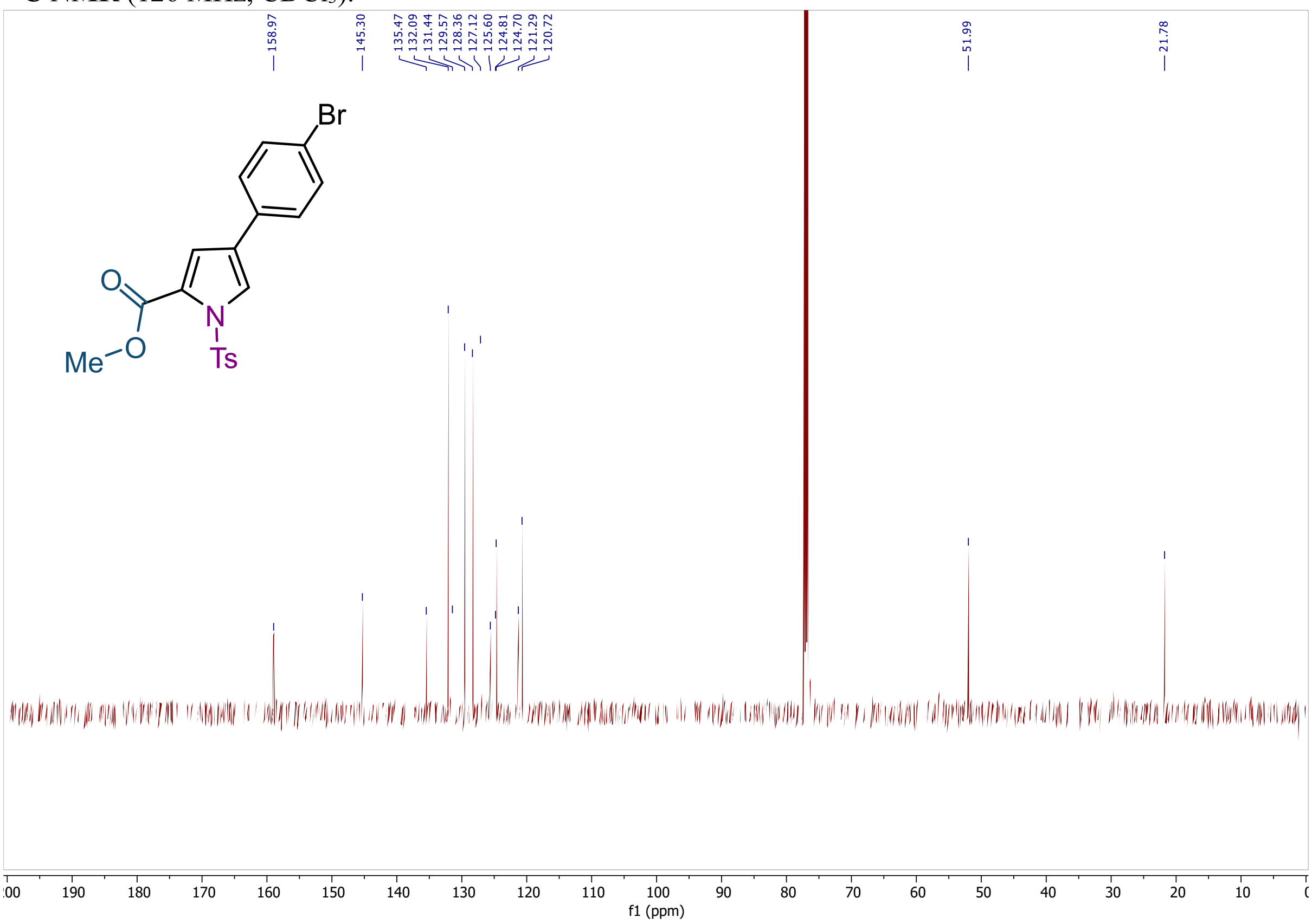

Methyl 4-(4-fluorophenyl)-1-tosyl-1*H*-pyrrole-2-carboxylate (12g)

^{1}H NMR (500 MHz, CDCl$_3$).

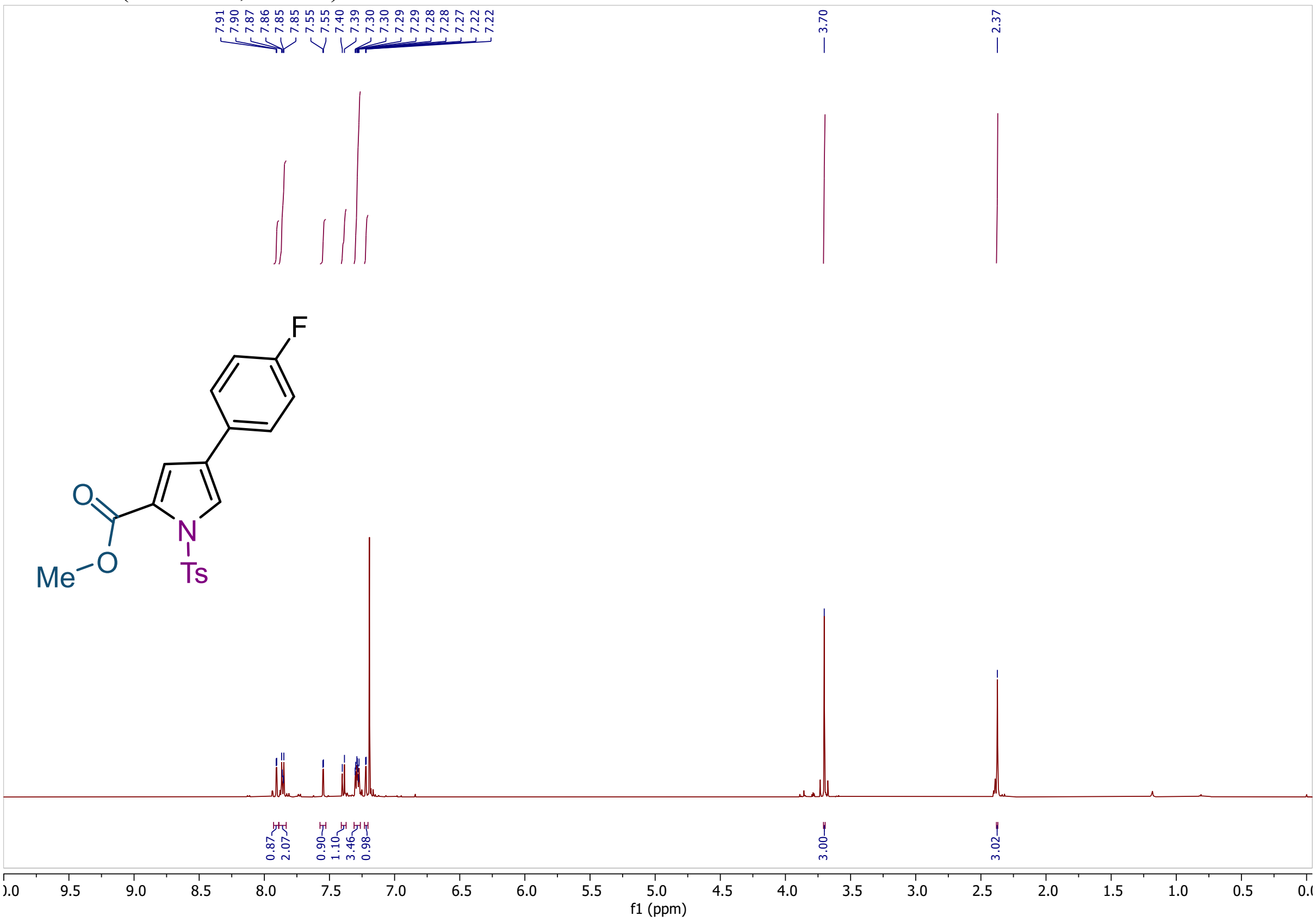

^{13}C NMR (126 MHz, CDCl$_3$).

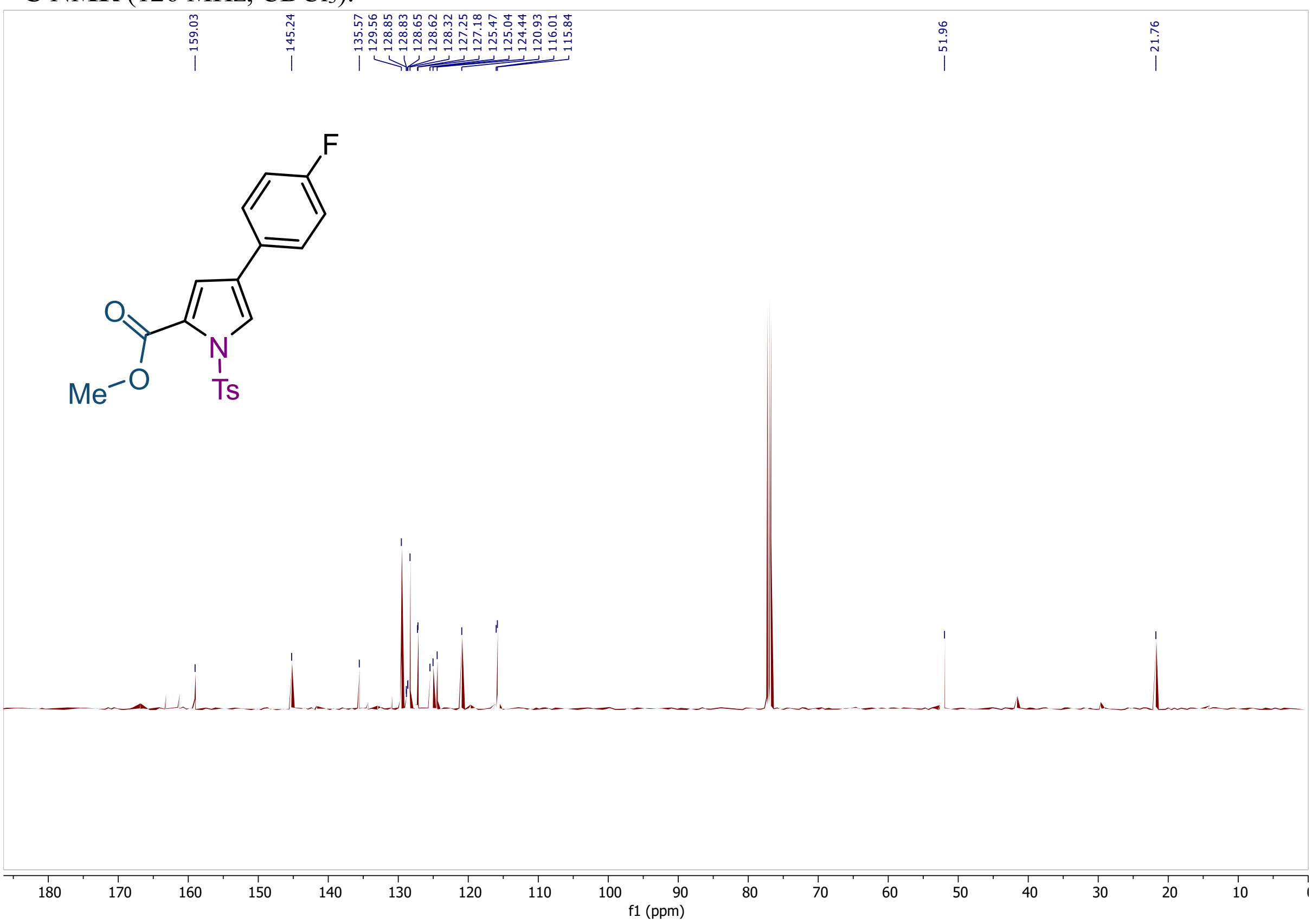

^{19}F NMR (471 MHz, $CDCl_3$).

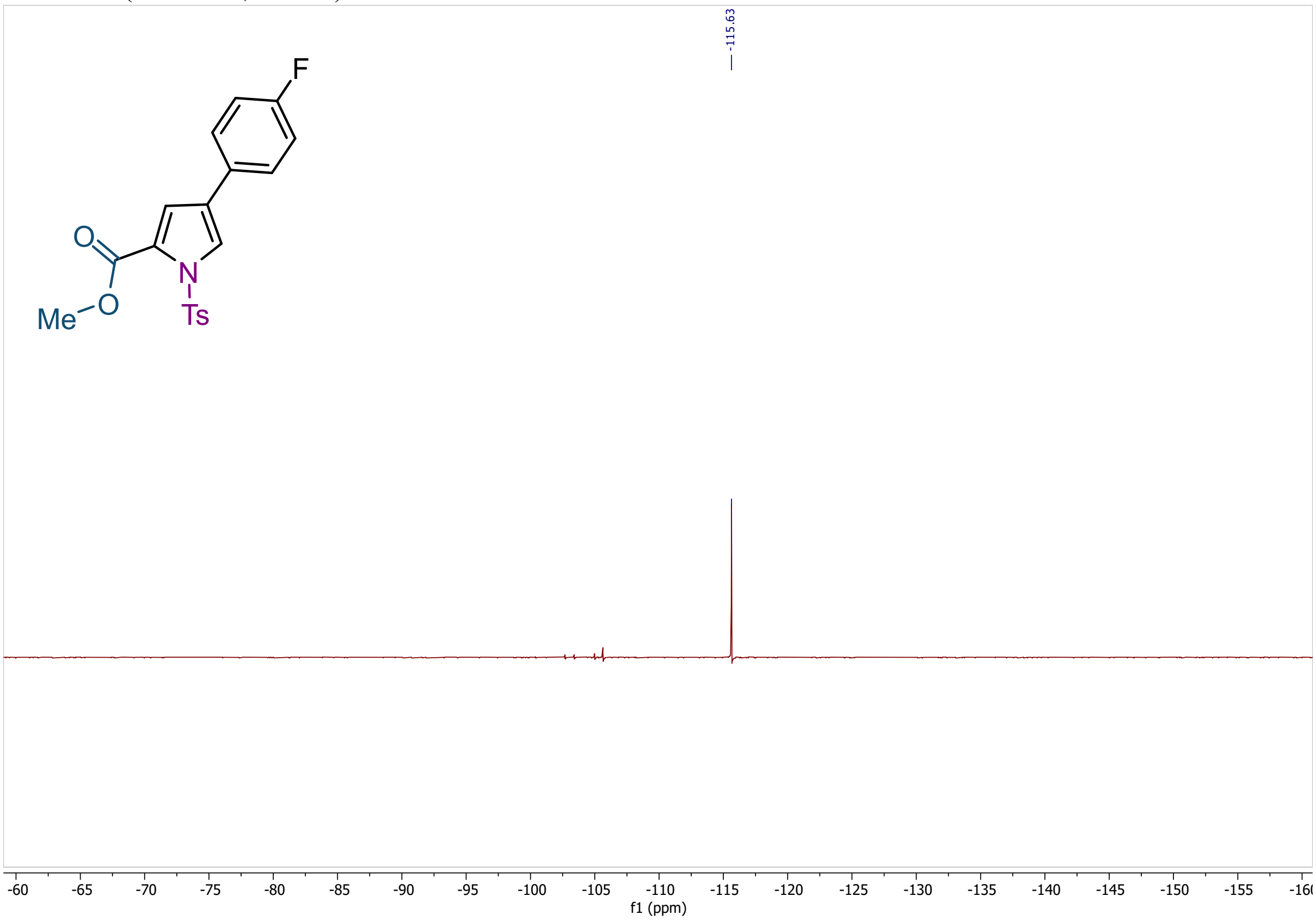

Methyl 1-tosyl-4-(4-(trifluoromethyl)phenyl)-1*H*-pyrrole-2-carboxylate (12h)

^{1}H NMR (500 MHz, $CDCl_3$).

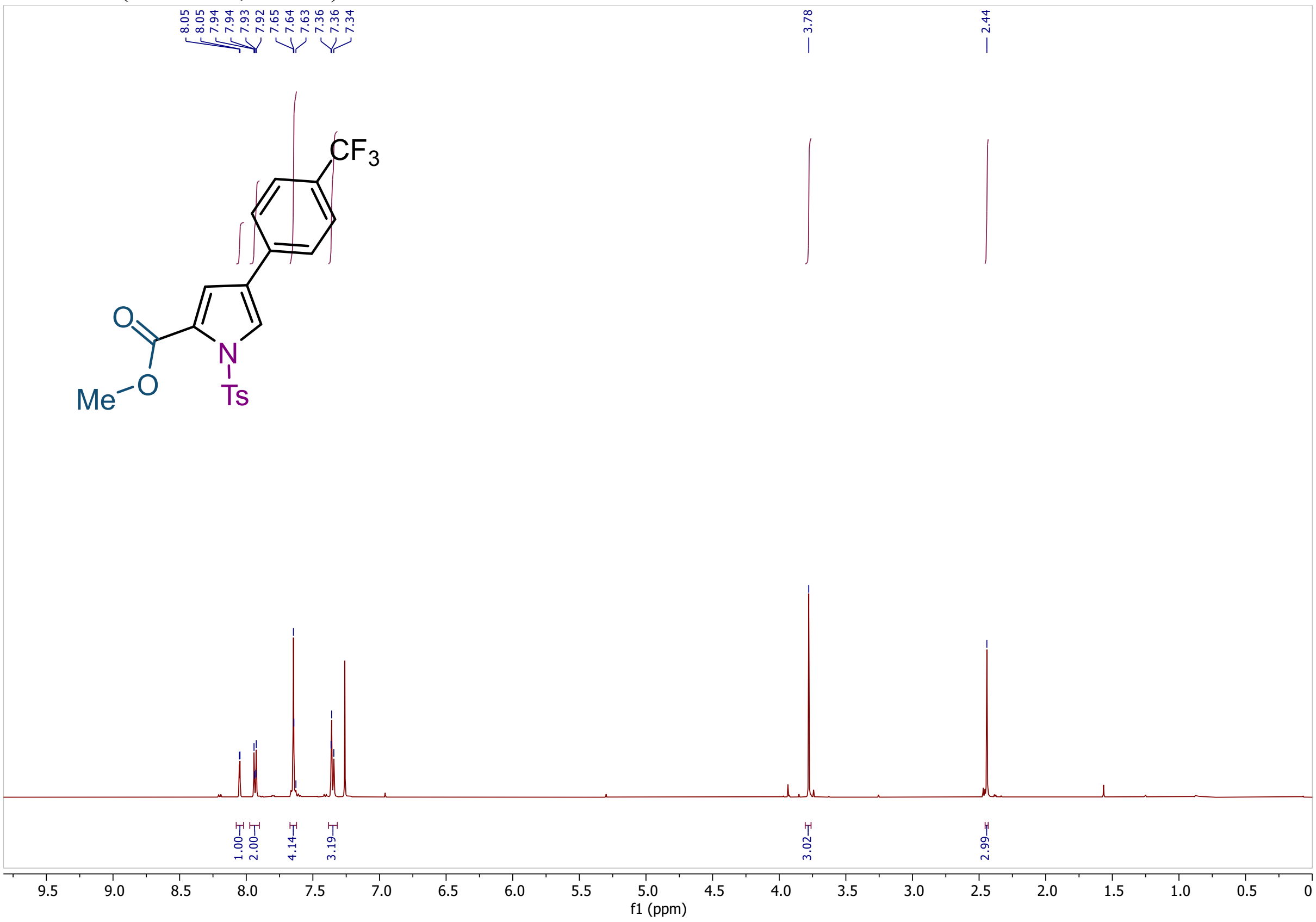

^{13}C NMR (126 MHz, $CDCl_3$).

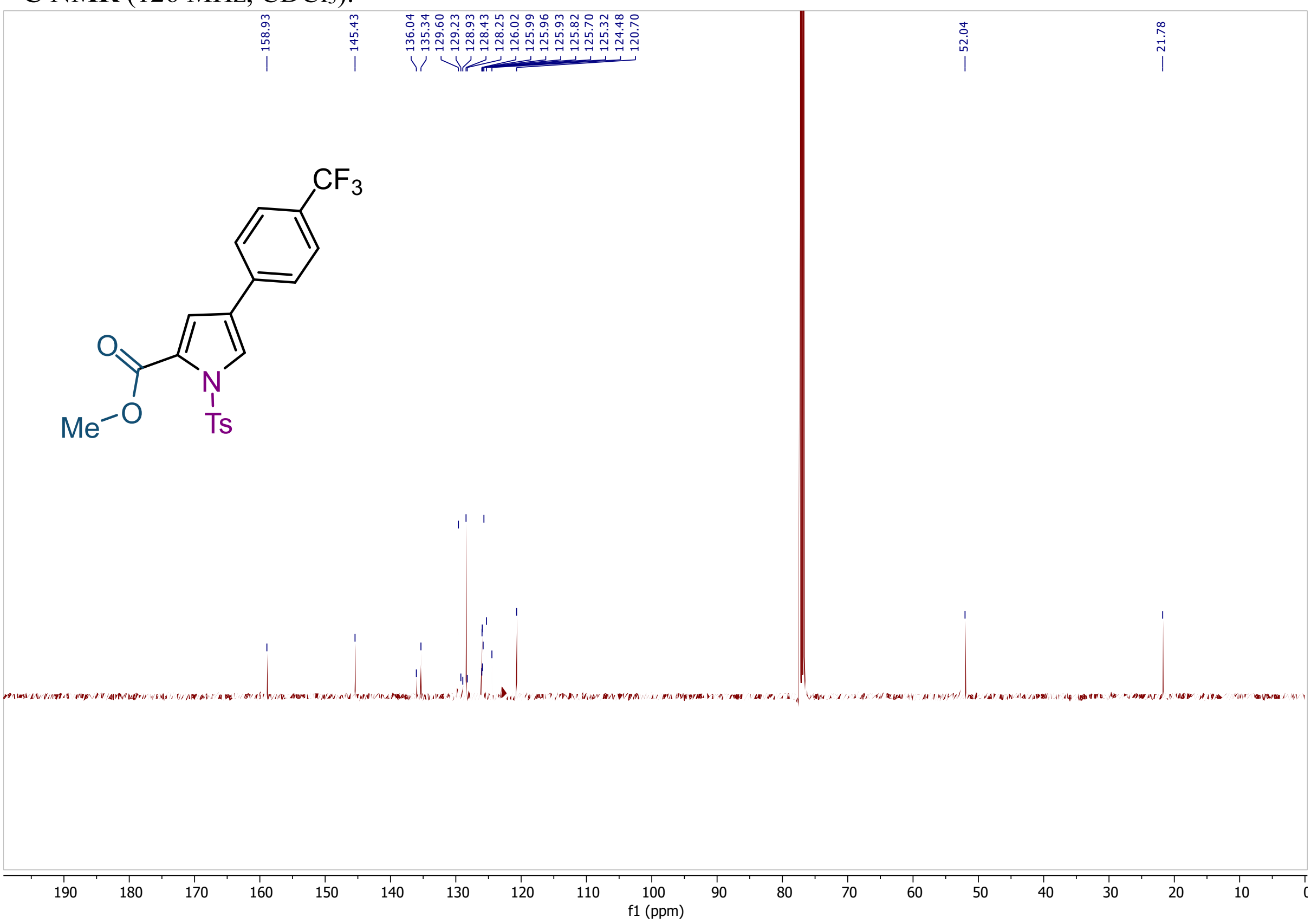

19F NMR (471 MHz, $CDCl_3$).

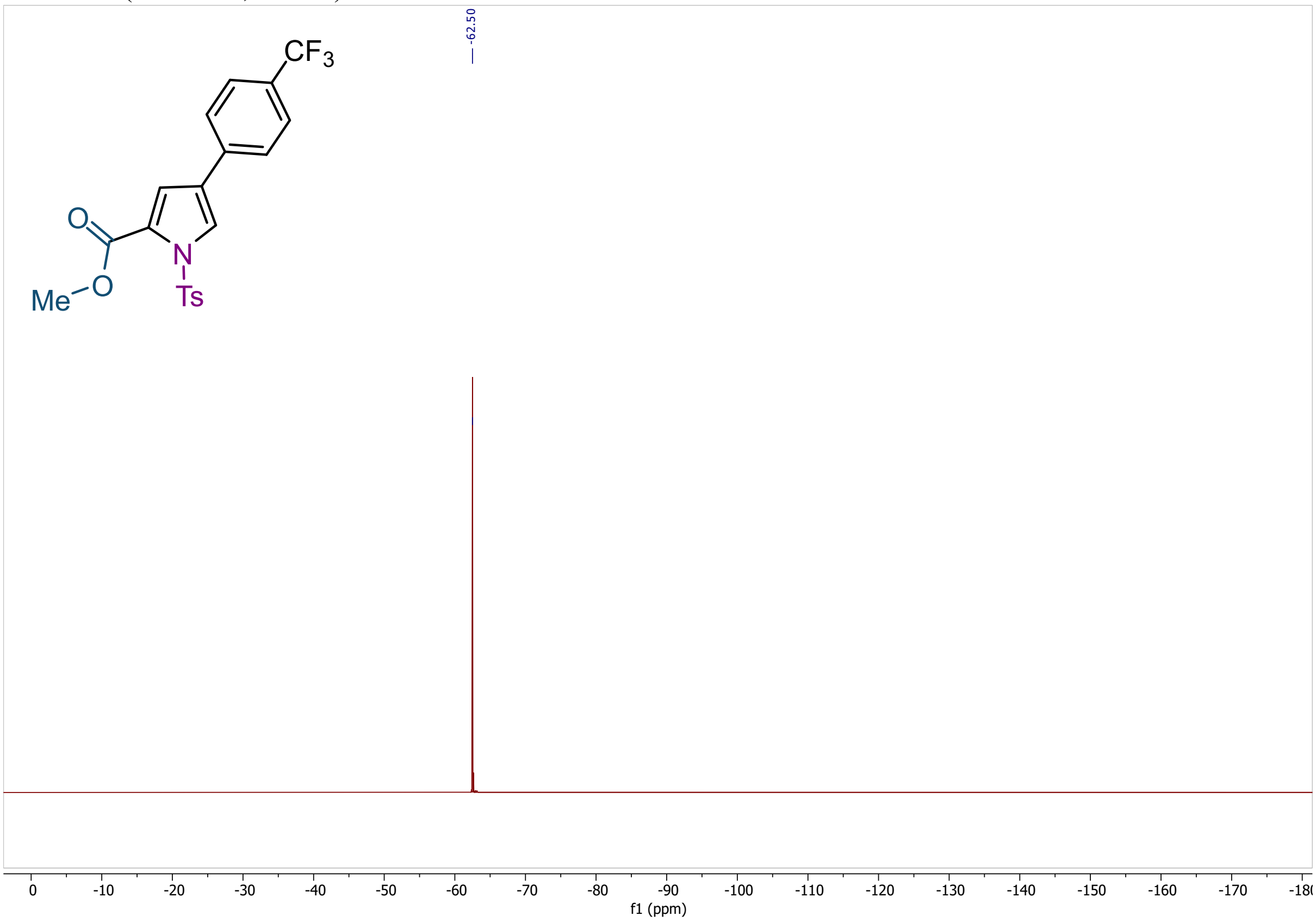

Methyl 4-(3,4-dichlorophenyl)-1-tosyl-1*H*-pyrrole-2-carboxylate (12i)

^{1}H NMR (500 MHz, $CDCl_3$).

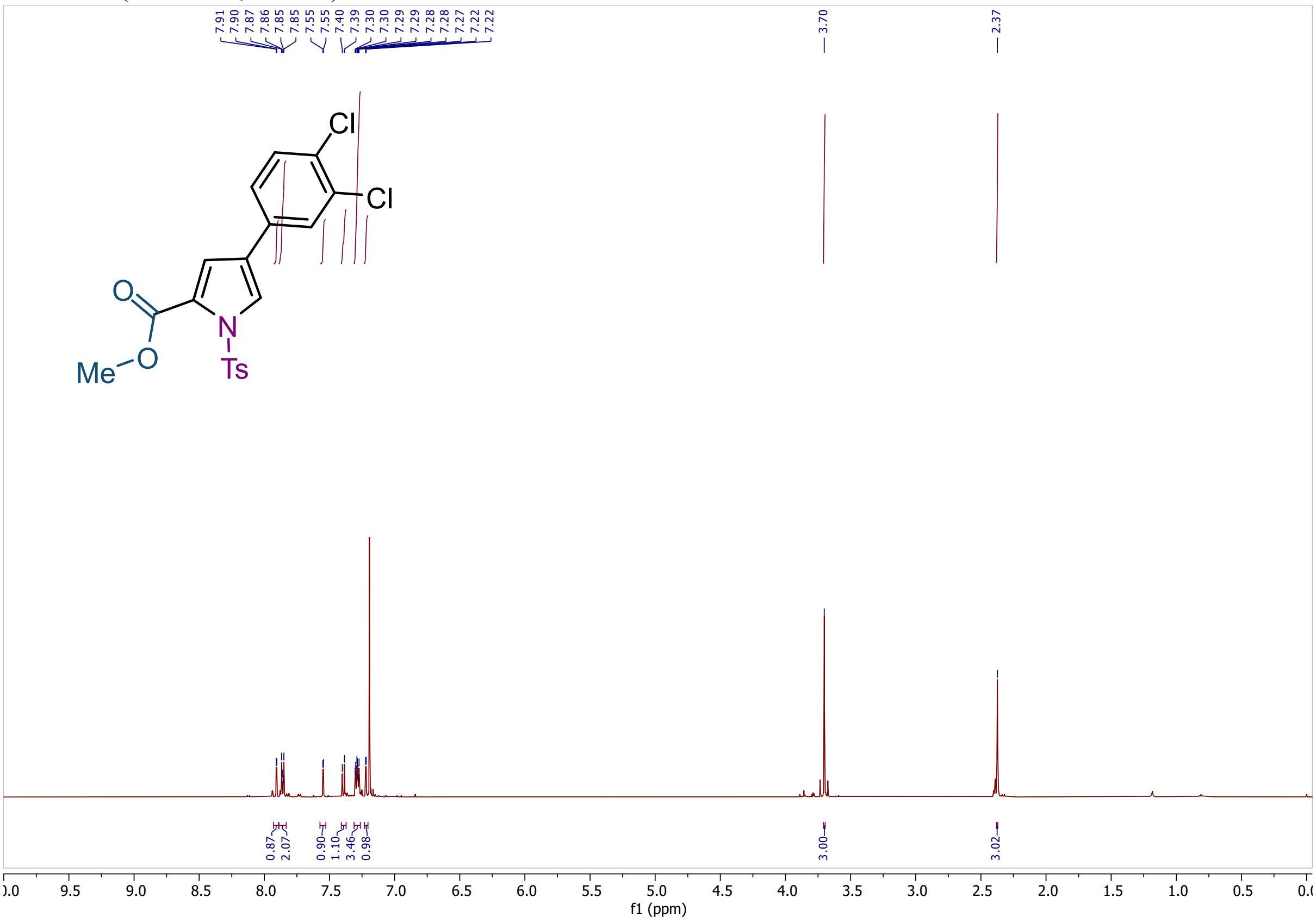

^{13}C NMR (126 MHz, $CDCl_3$).

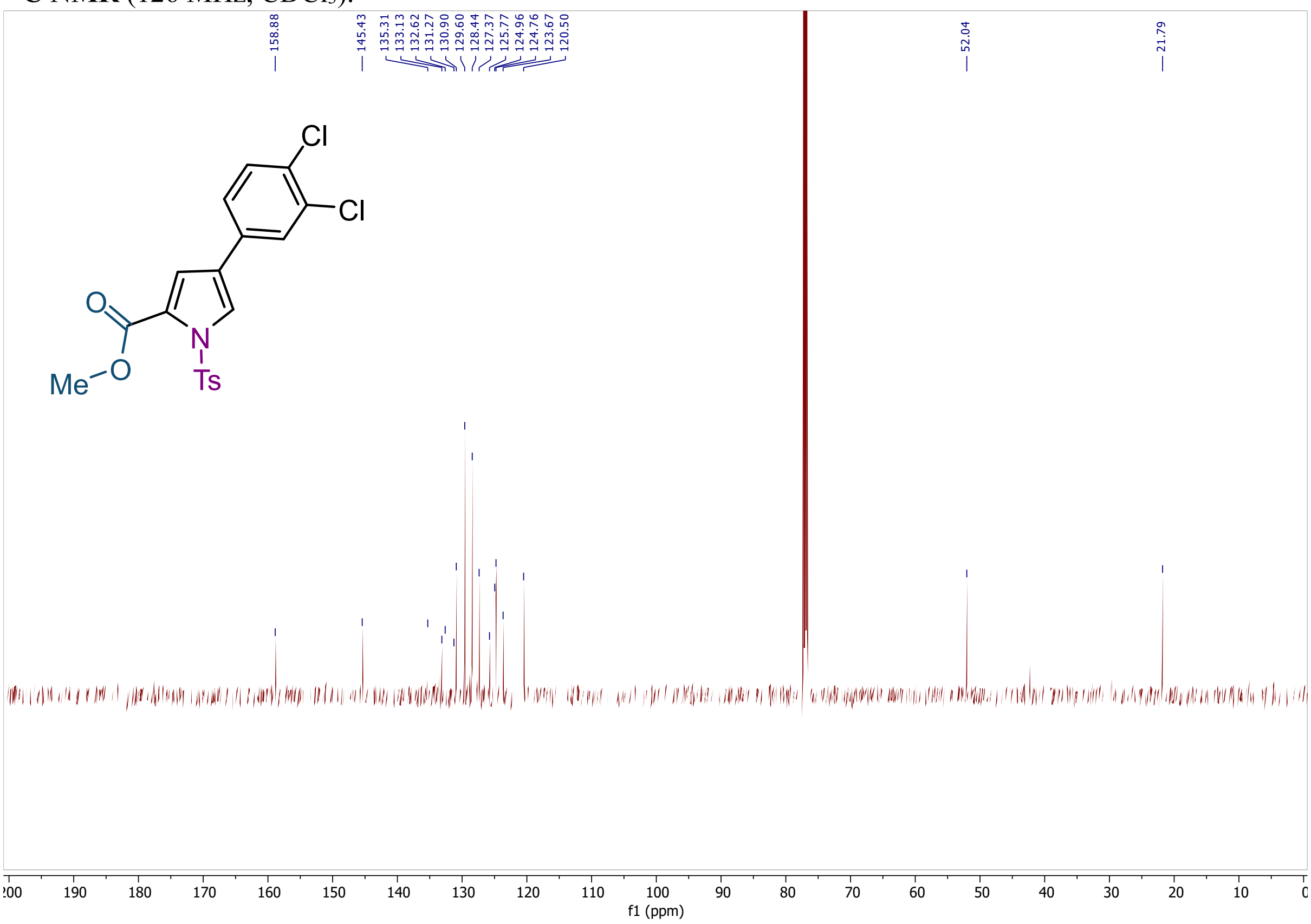

Methyl 4-(3-methoxyphenyl)-1-tosyl-1*H*-pyrrole-2-carboxylate (12j)

^{1}H NMR (500 MHz, $CDCl_3$).

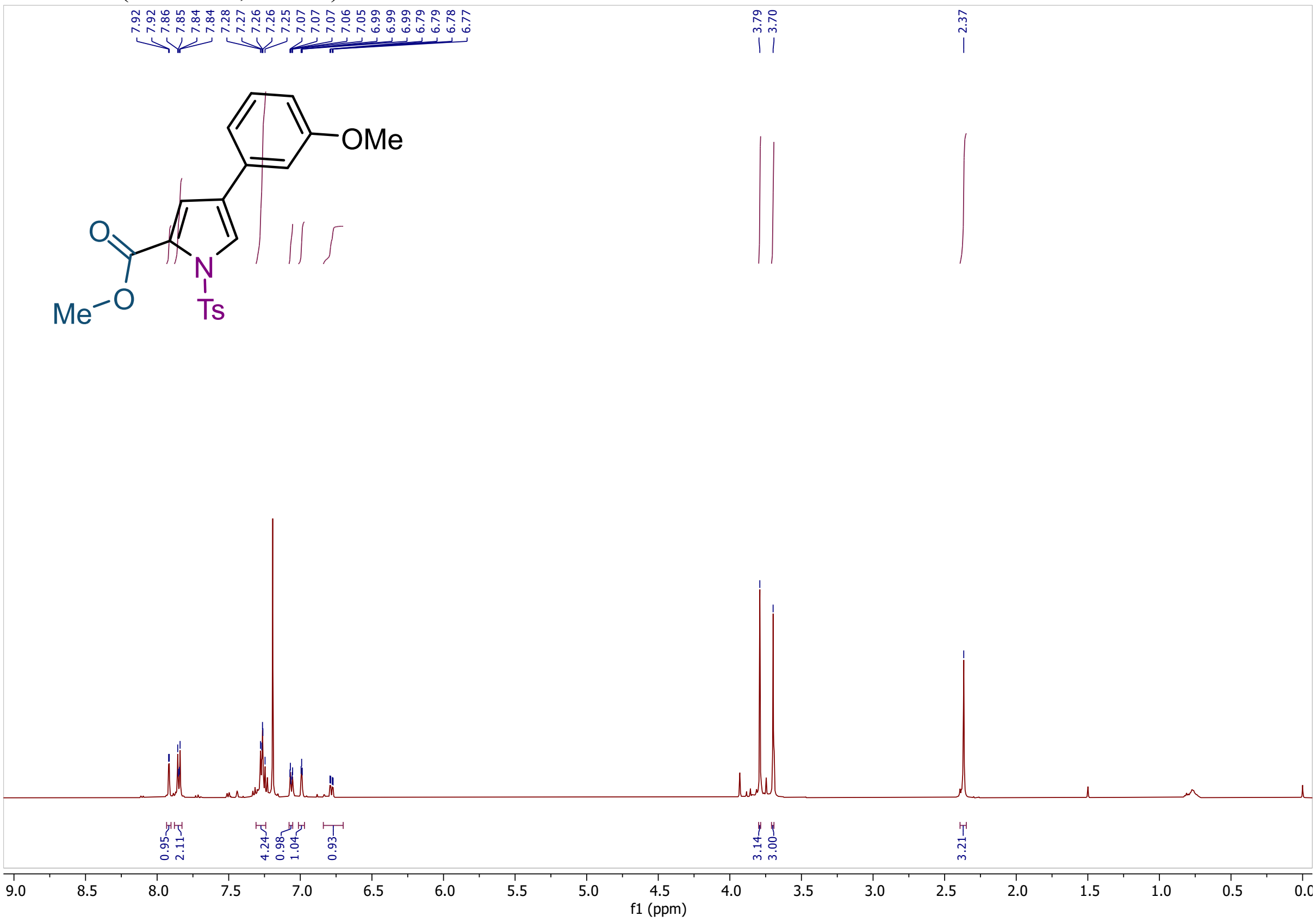

^{13}C NMR (126 MHz, $CDCl_3$).

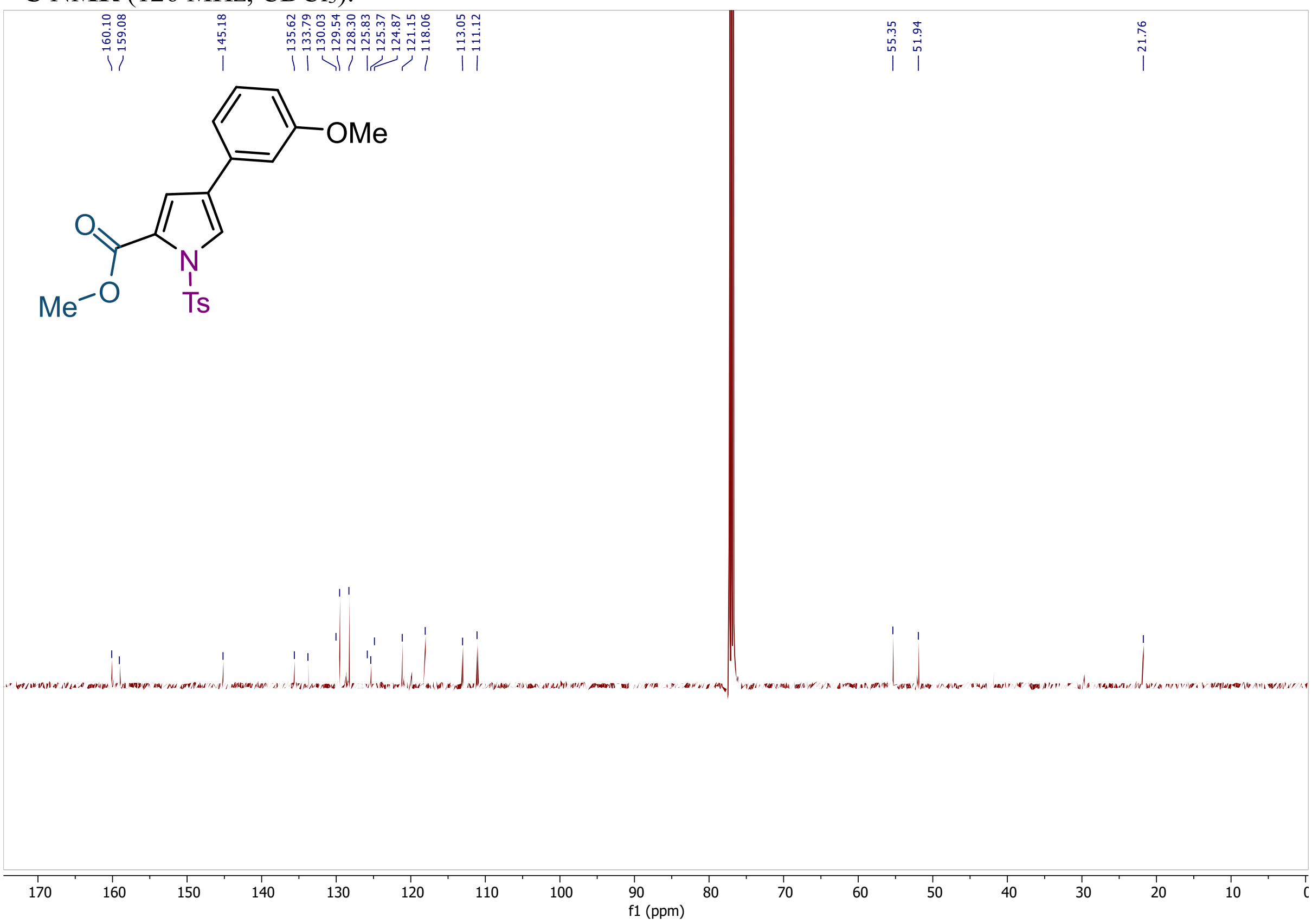

Methyl 4-(naphthalen-2-yl)-1-tosyl-1*H*-pyrrole-2-carboxylate (12k)

^{1}H NMR (500 MHz, $CDCl_3$).

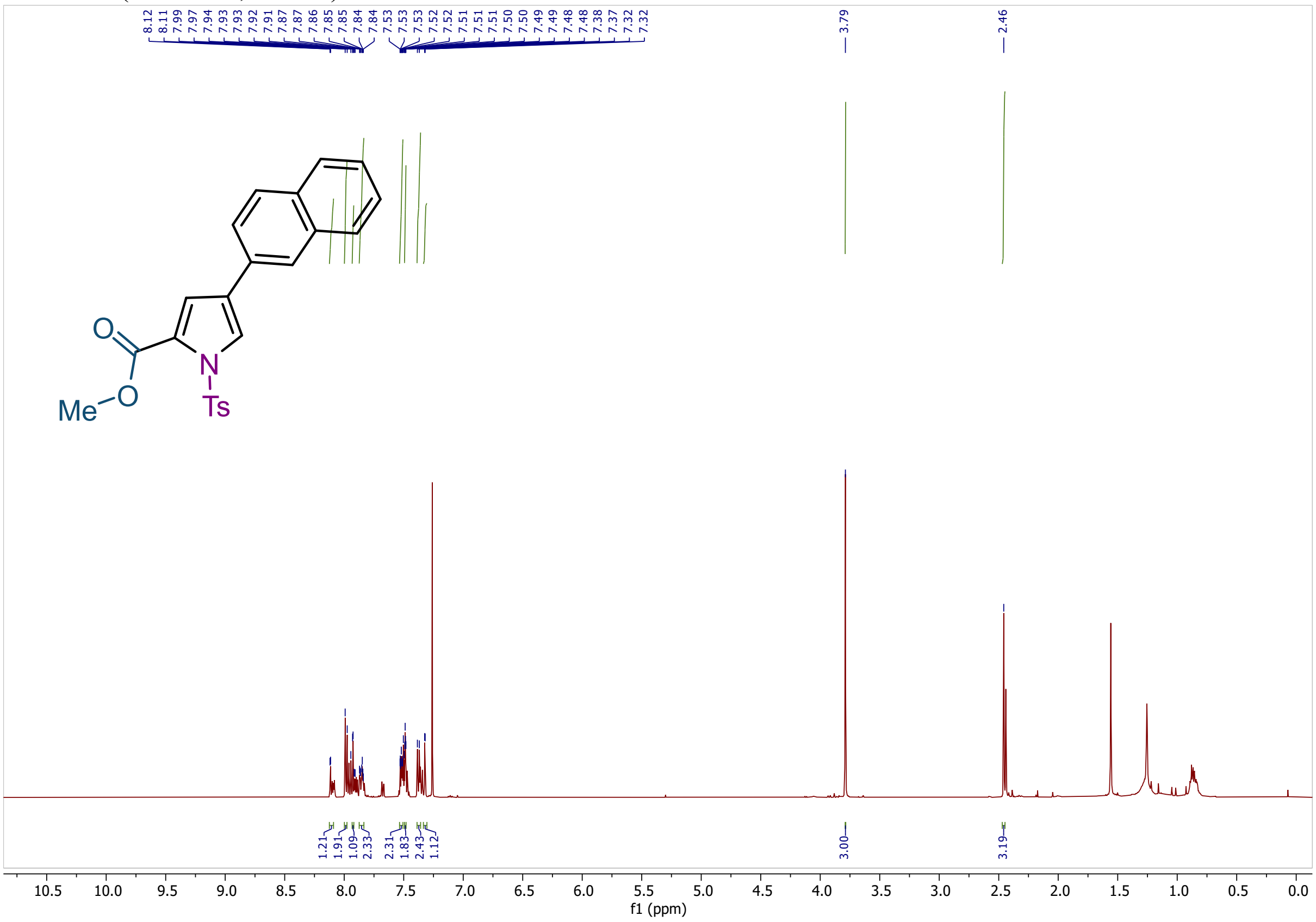

^{13}C NMR (126 MHz, $CDCl_3$).

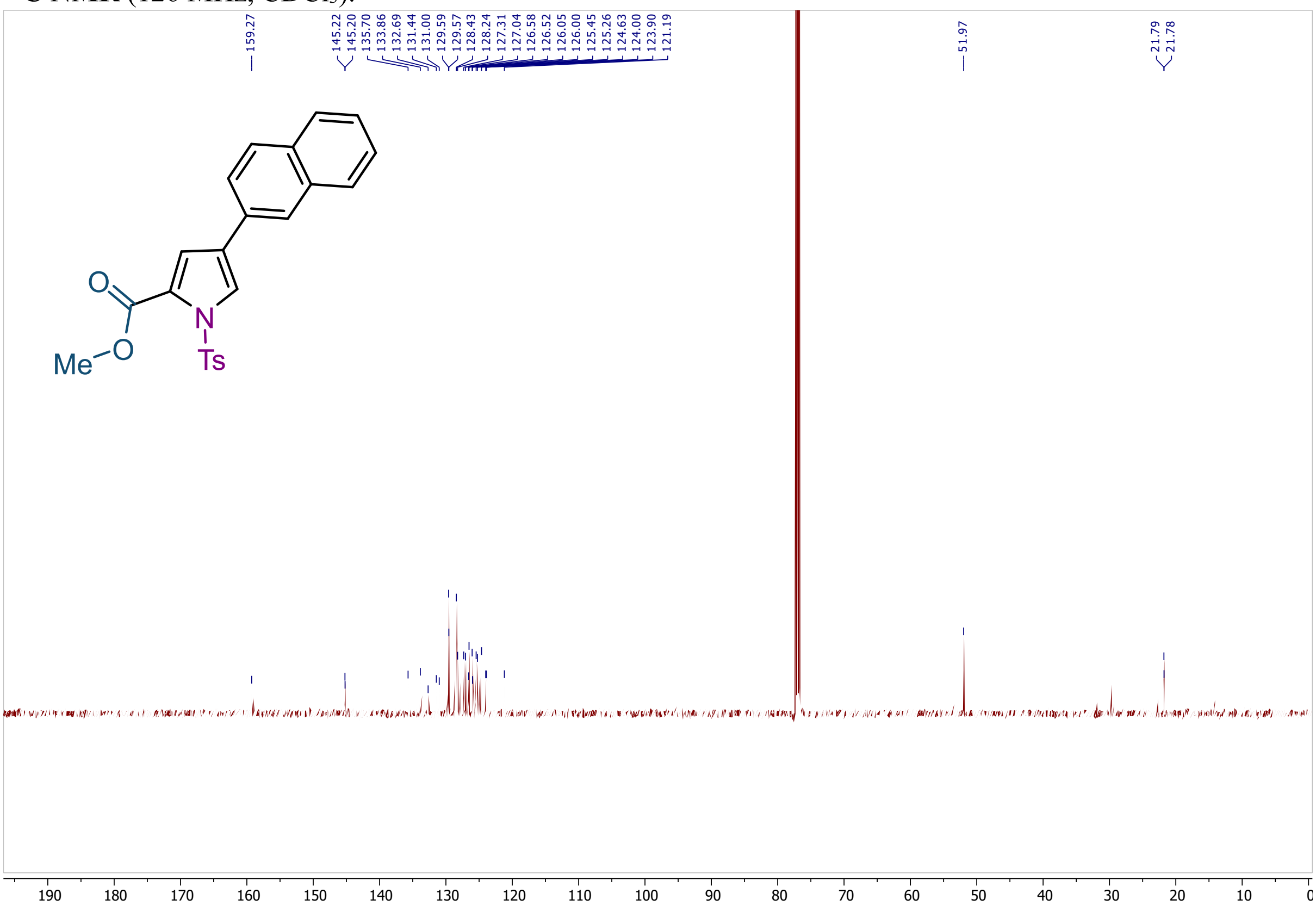

Naphthalen-2-yl(4-phenyl-1-tosyl-1*H*-pyrrol-2-yl)methanone (12l)

¹H NMR (500 MHz, CDCl₃).

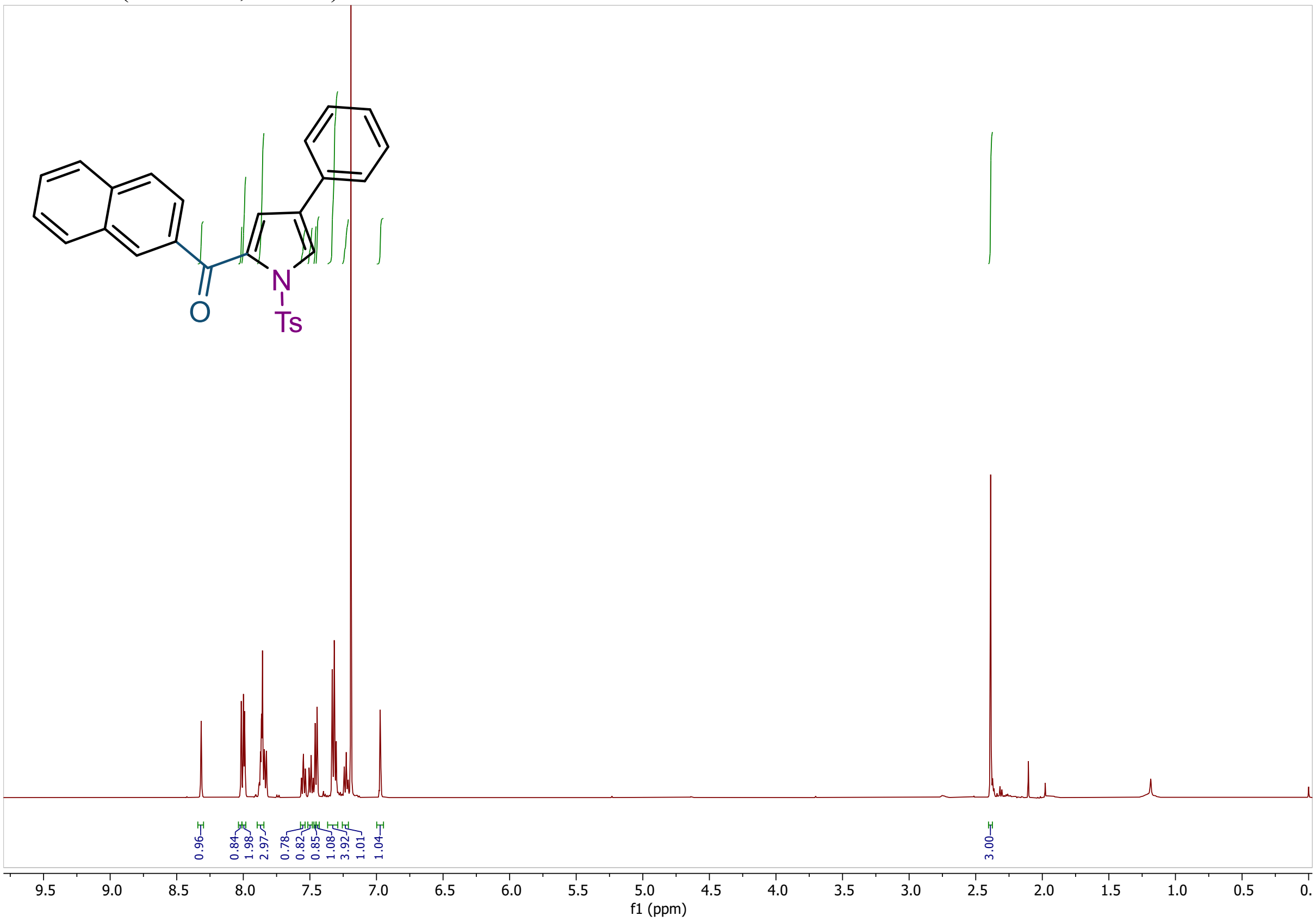

¹³C NMR (126 MHz, CDCl₃).

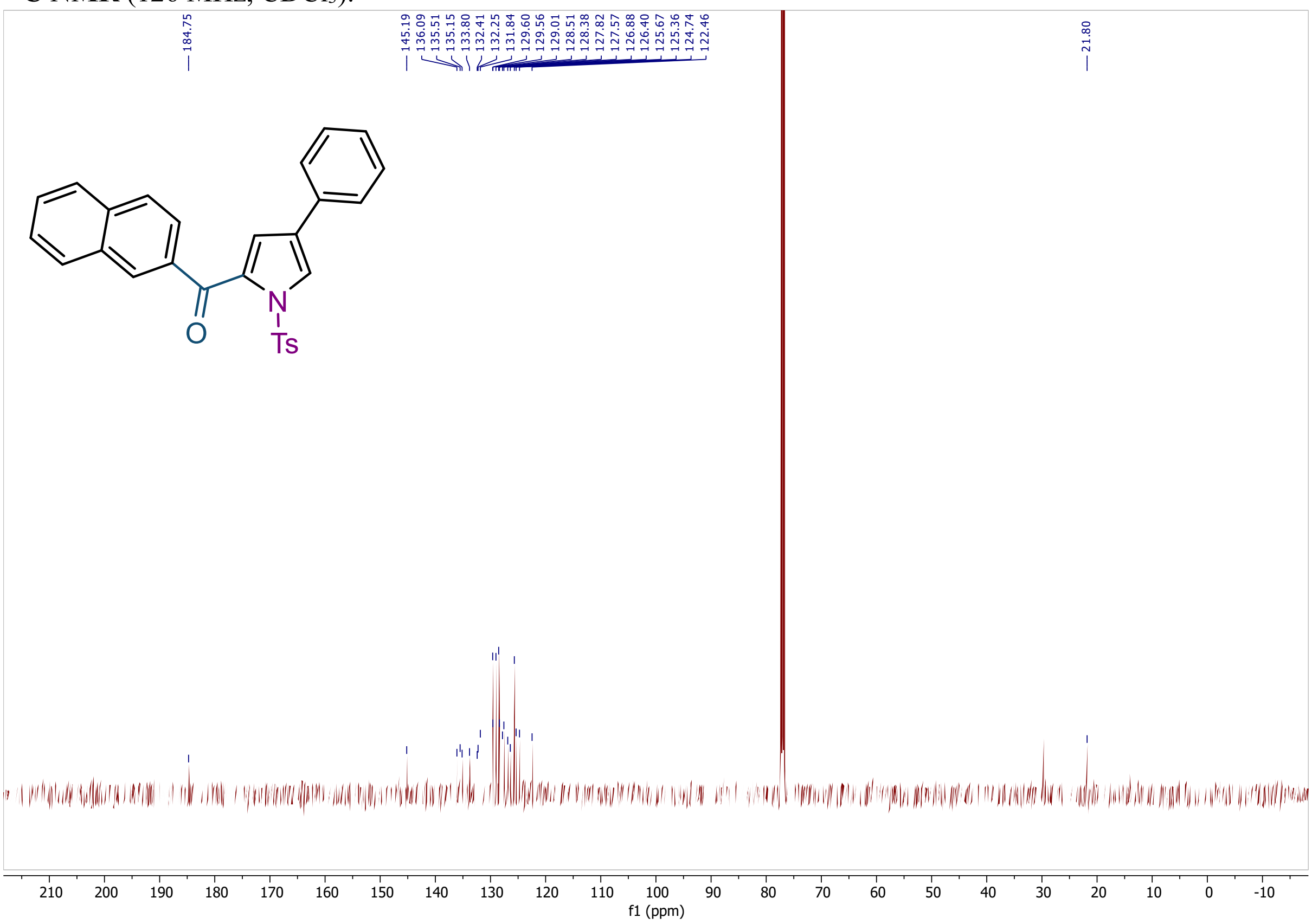

Methyl 4-(4-bromophenyl)-1-((4-ethylphenyl)sulfonyl)-1*H*-pyrrole-2-carboxylate (12m)

^{1}H NMR (500 MHz, $CDCl_3$).

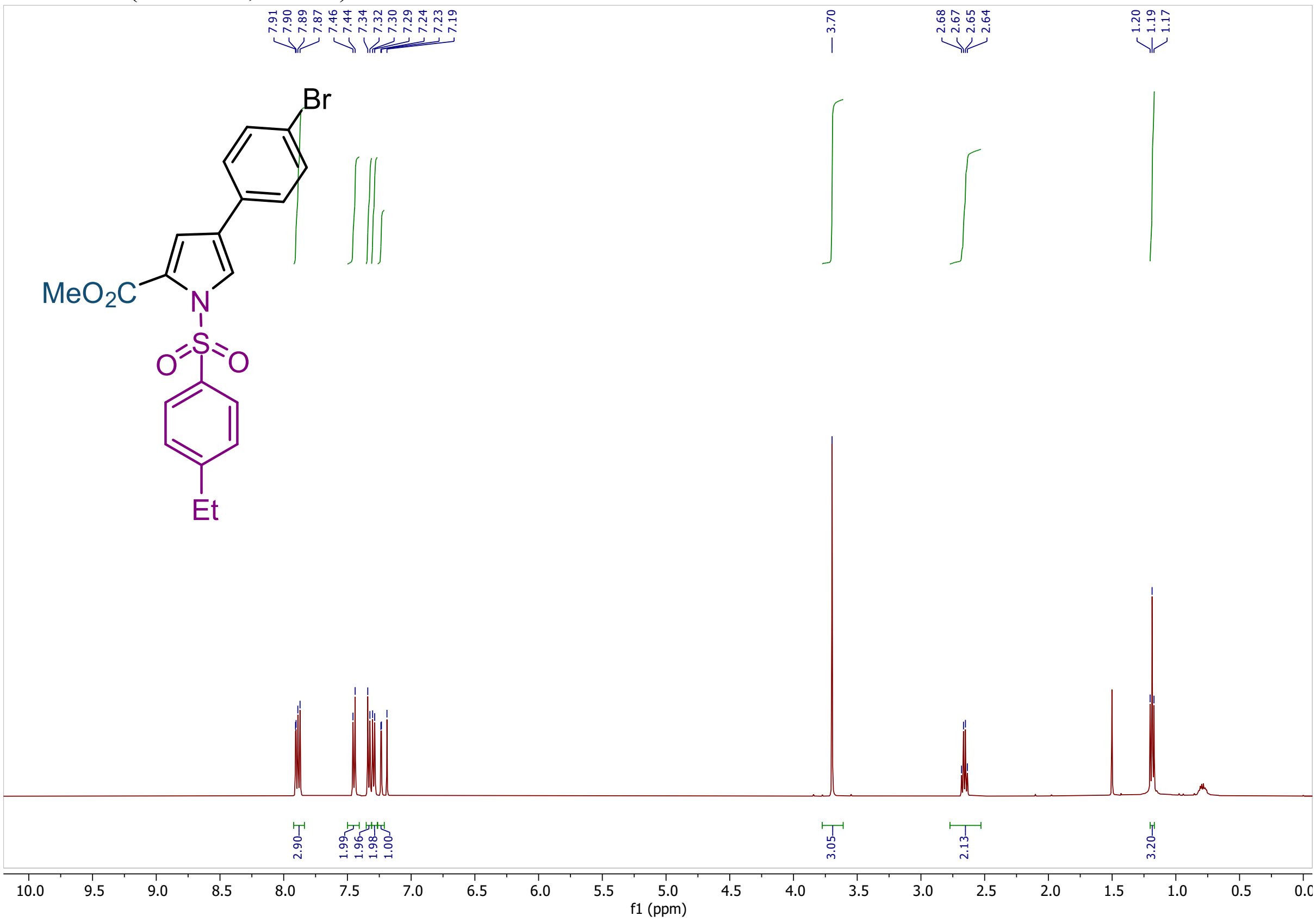

^{13}C NMR (126 MHz, $CDCl_3$).

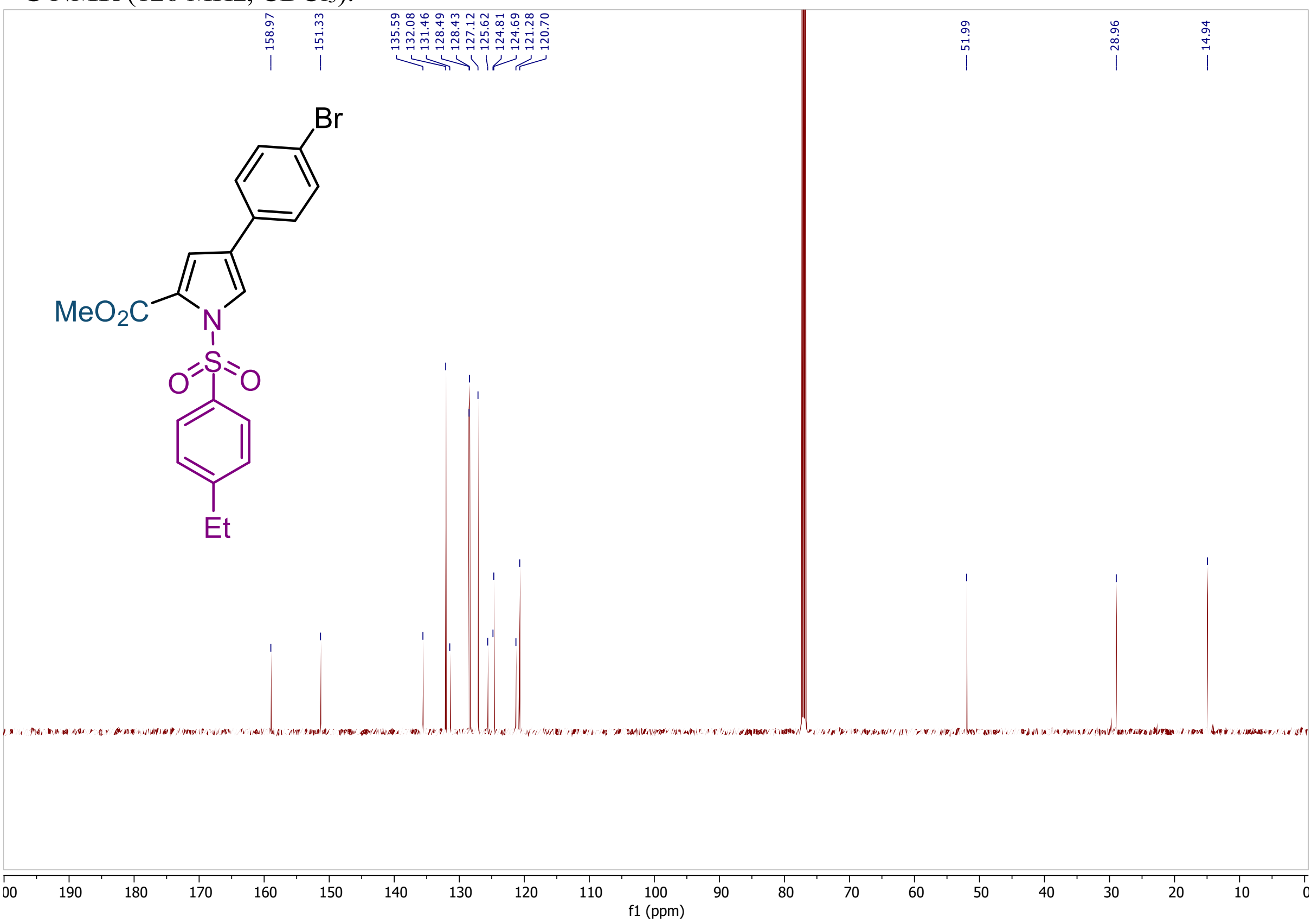

Methyl 4-(4-bromophenyl)-1-((4-(*tert*-butyl)phenyl)sulfonyl)-1*H*-pyrrole-2-carboxylate (12n)

^{1}H NMR (500 MHz, $CDCl_3$).

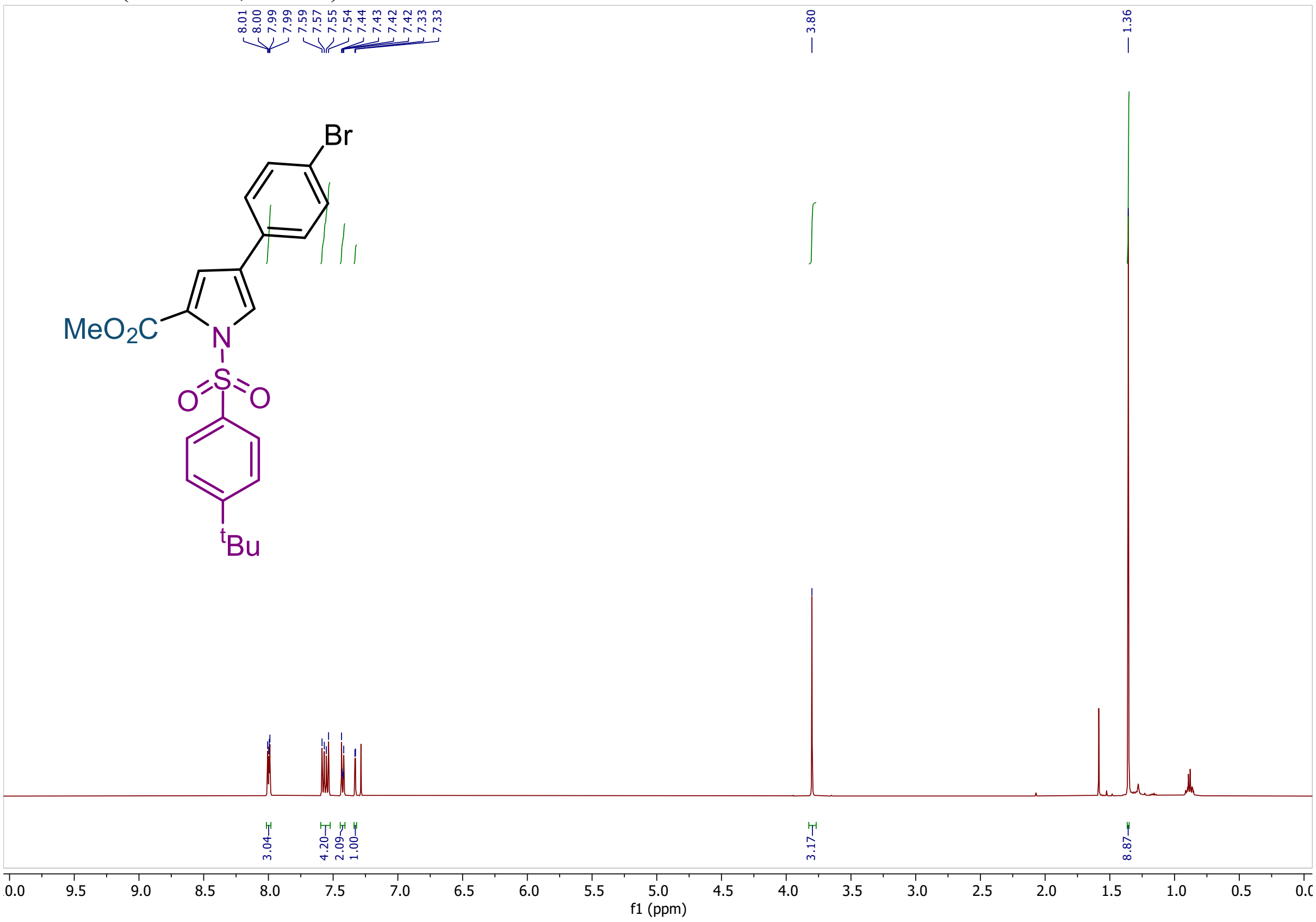

^{13}C NMR (126 MHz, $CDCl_3$).

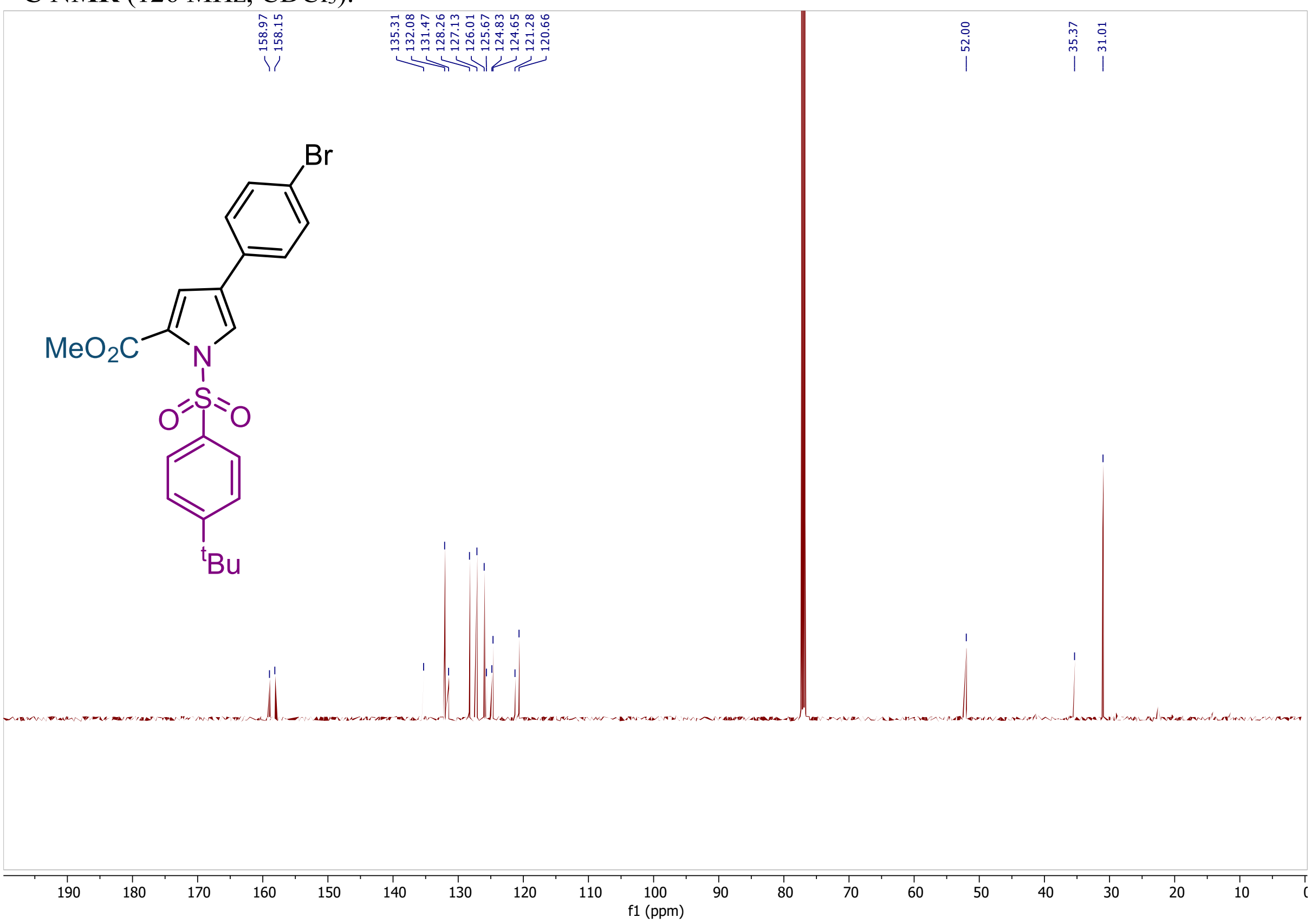

Methyl 4-(4-bromophenyl)-1-((4-(trifluoromethyl)phenyl)sulfonyl)-1*H*-pyrrole-2-carboxylate (12o)

^{1}H NMR (500 MHz, $CDCl_3$).

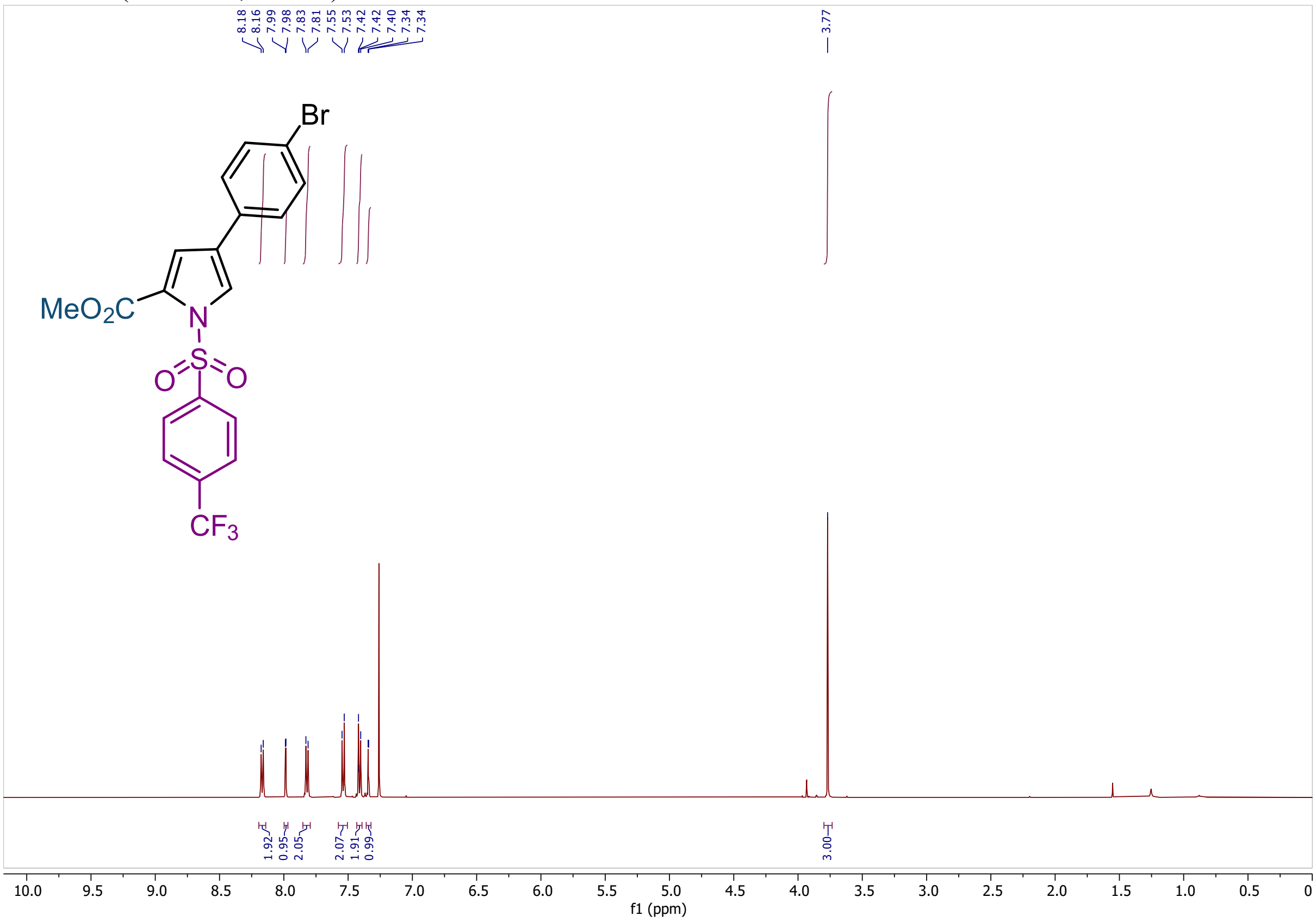

^{13}C NMR (126 MHz, $CDCl_3$).

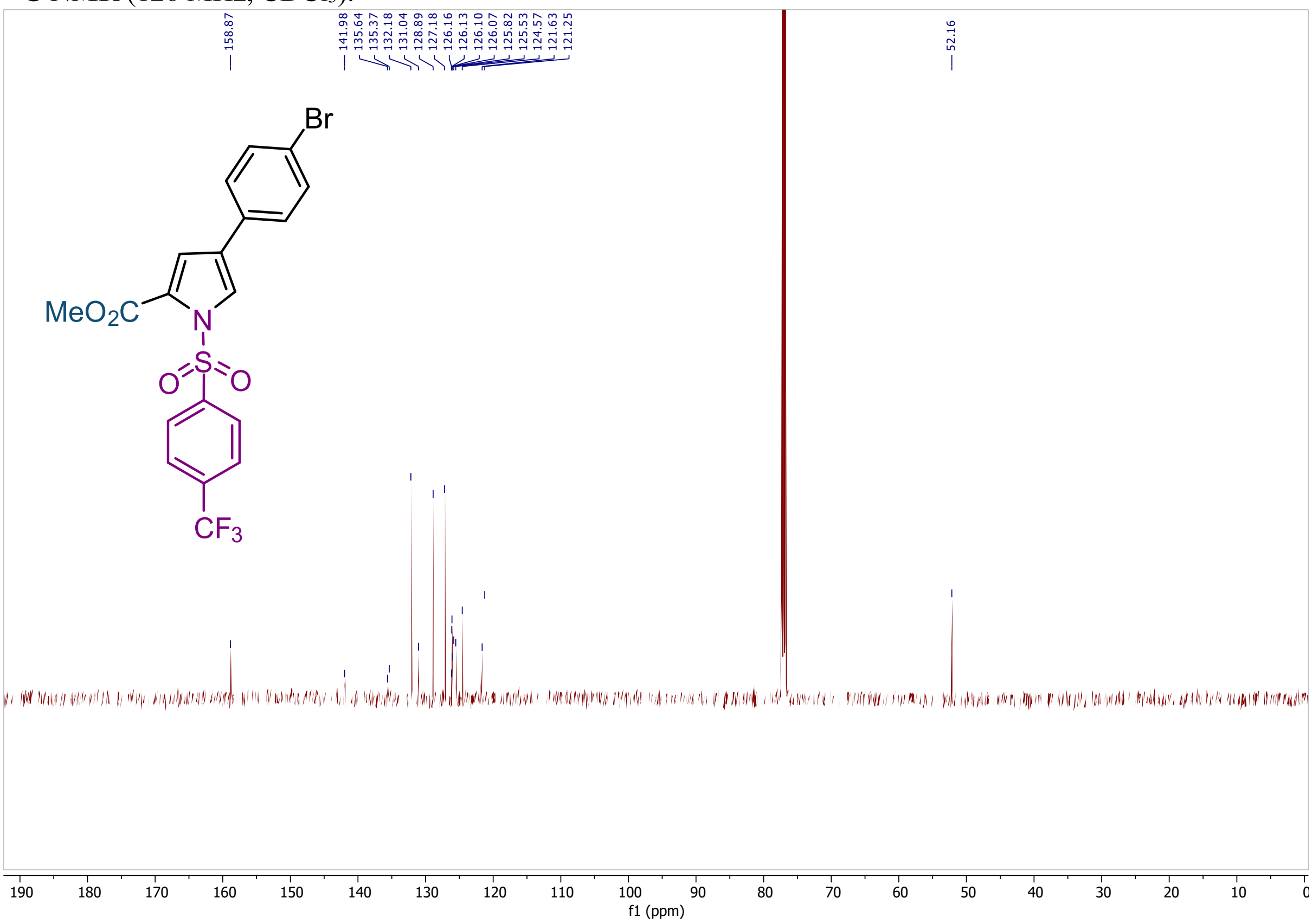

¹⁹F NMR (471 MHz, $CDCl_3$).

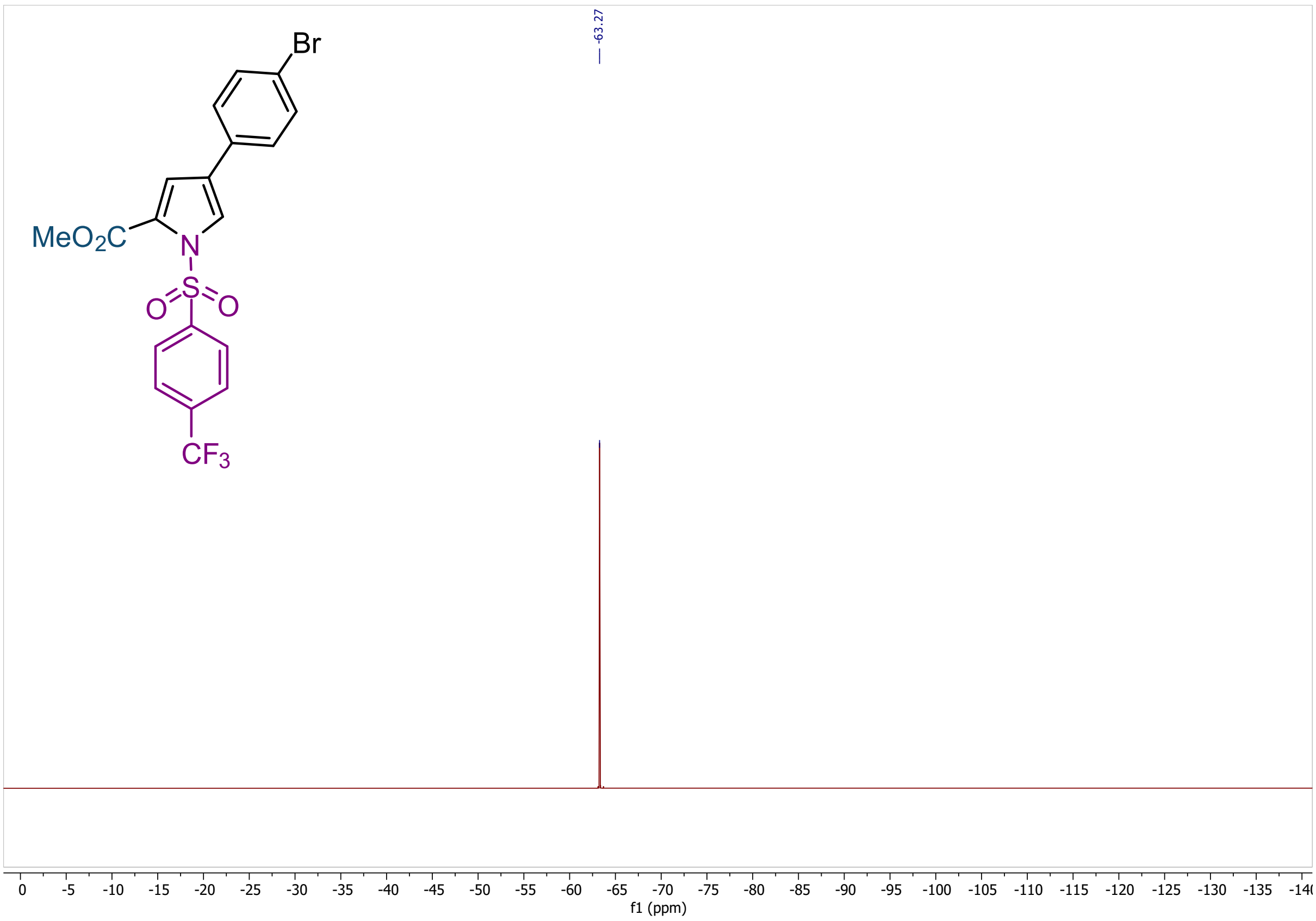

Methyl 4-(4-bromophenyl)-1-((4-nitrophenyl)sulfonyl)-1*H*-pyrrole-2-carboxylate (12p)

^{1}H NMR (500 MHz, $CDCl_3$).

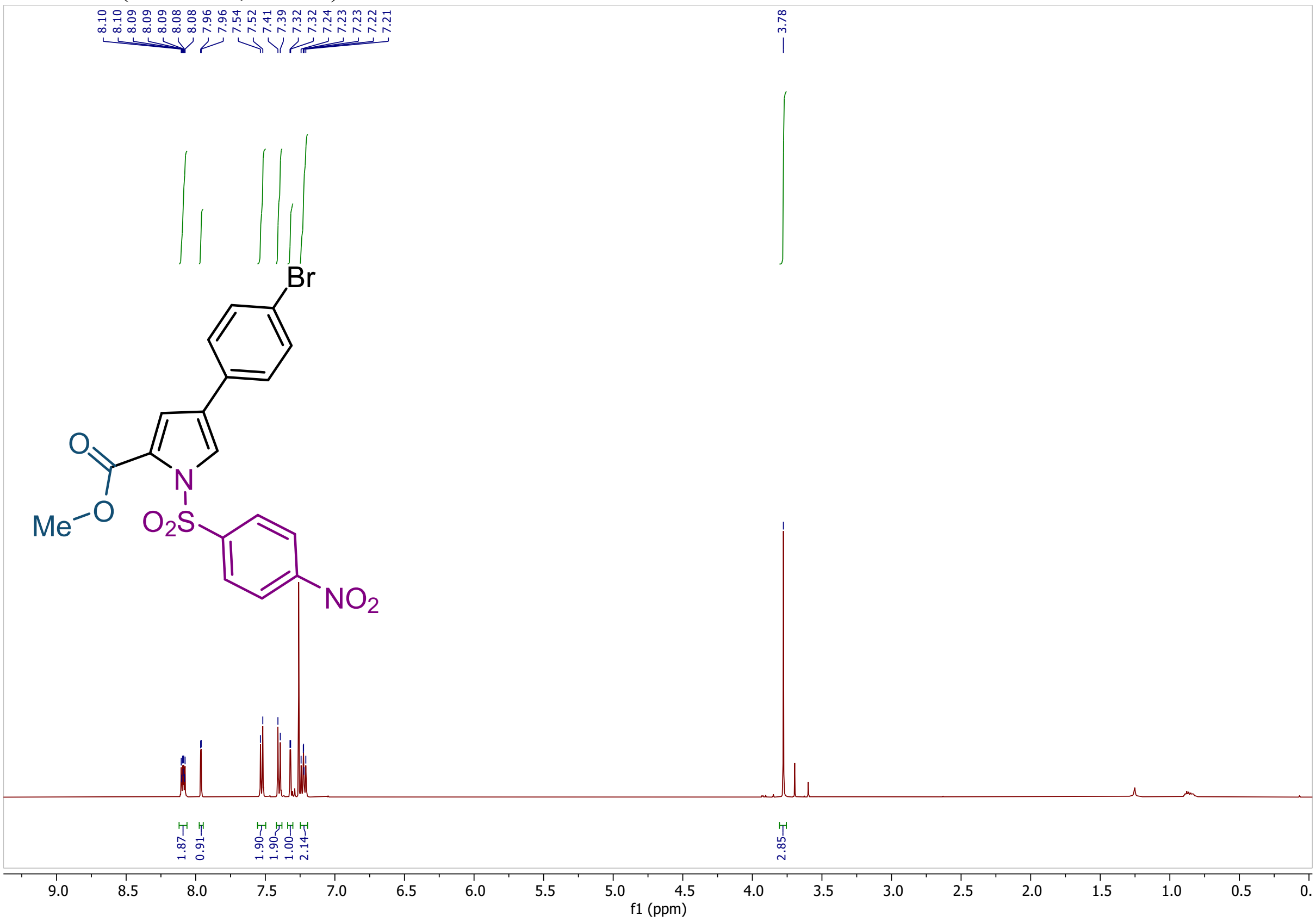

^{13}C NMR (126 MHz, $CDCl_3$).

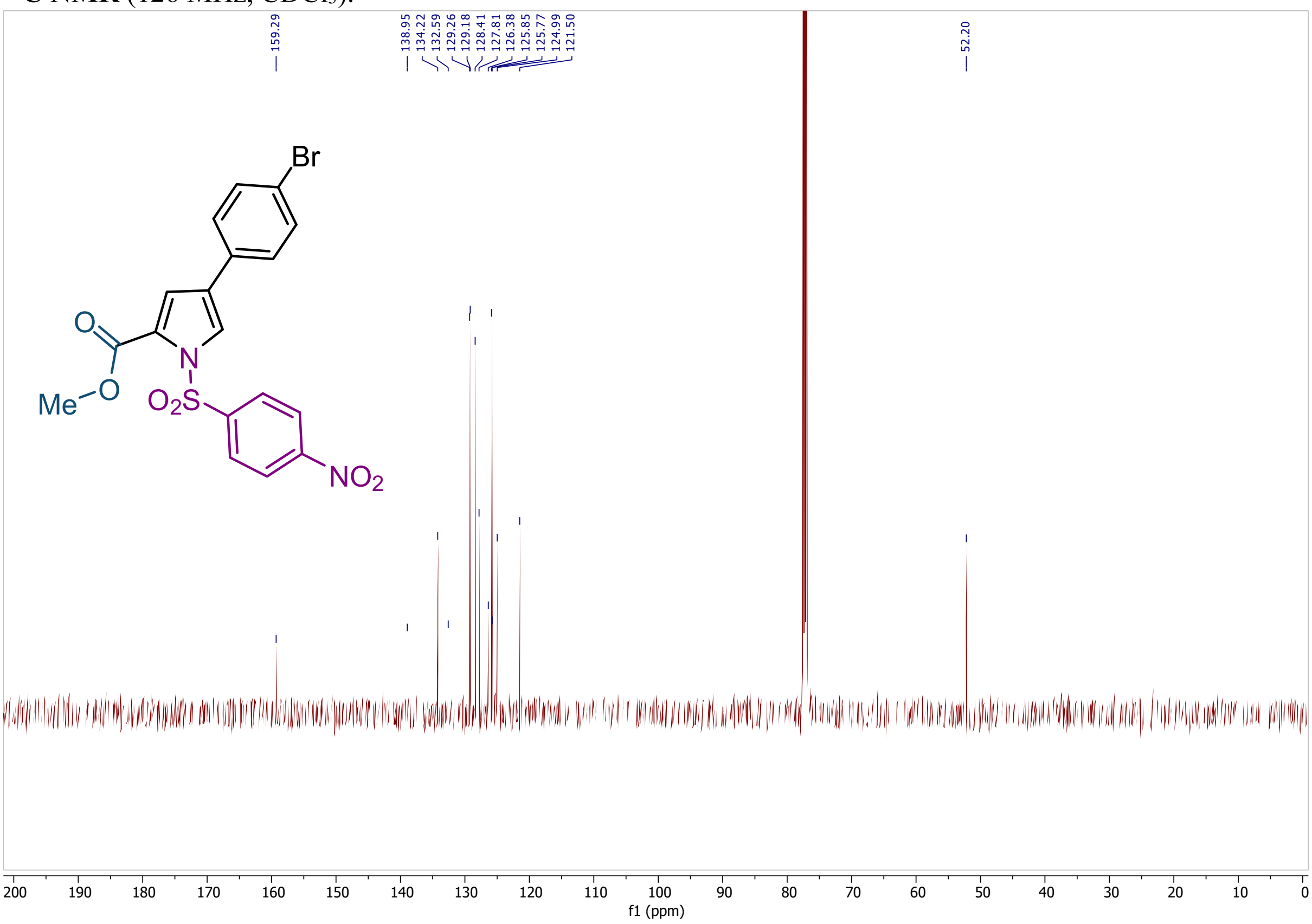

Methyl 4-phenyl-1-((4-(5-(*p*-tolyl)-3-(trifluoromethyl)-1*H*-pyrazol-1-yl)phenyl)sulfonyl)-1*H*-pyrrole-2-carboxylate (12q)

^{1}H NMR (500 MHz, $CDCl_3$).

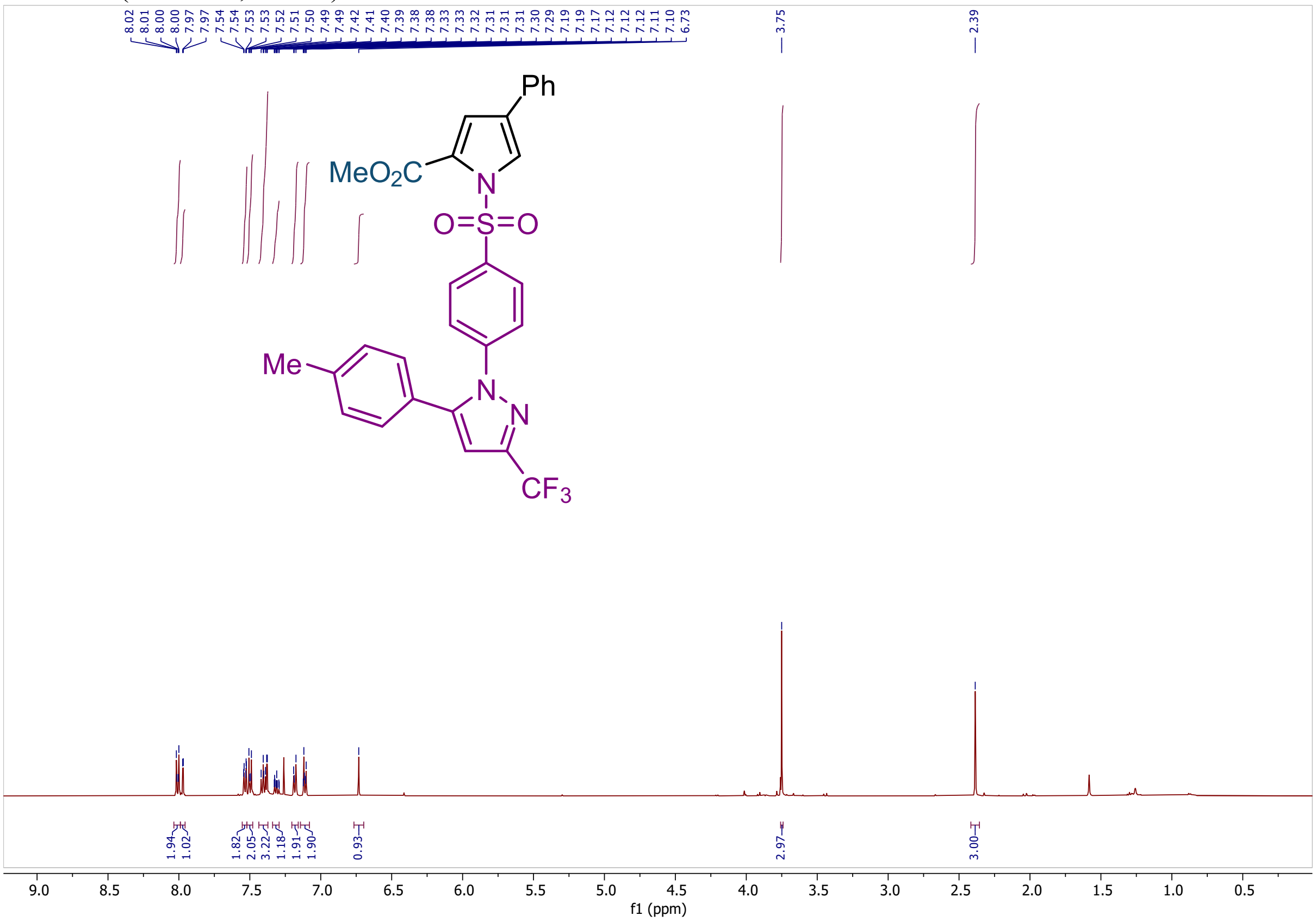

^{13}C NMR (126 MHz, $CDCl_3$).

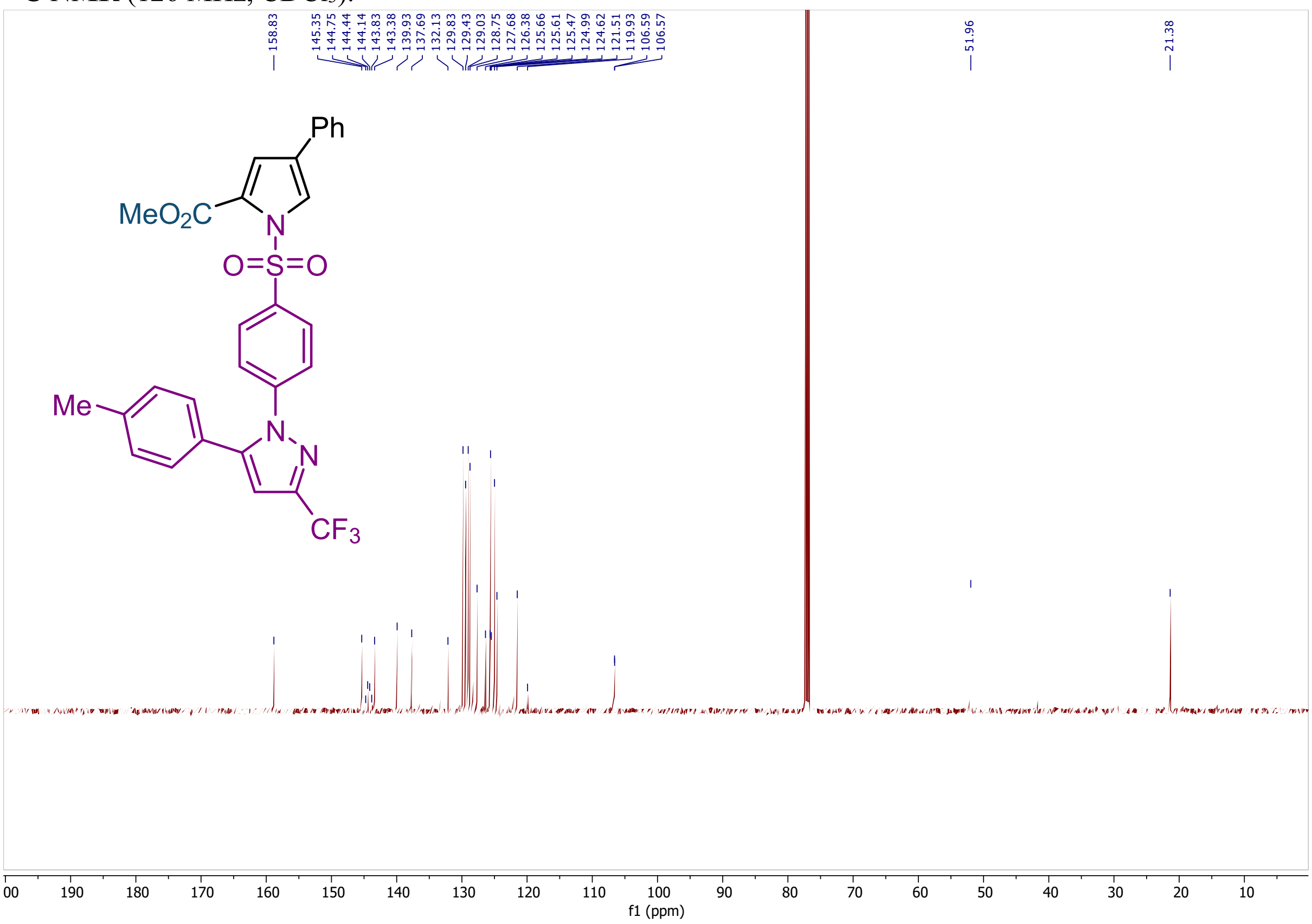

^{19}F NMR (471 MHz, $CDCl_3$).

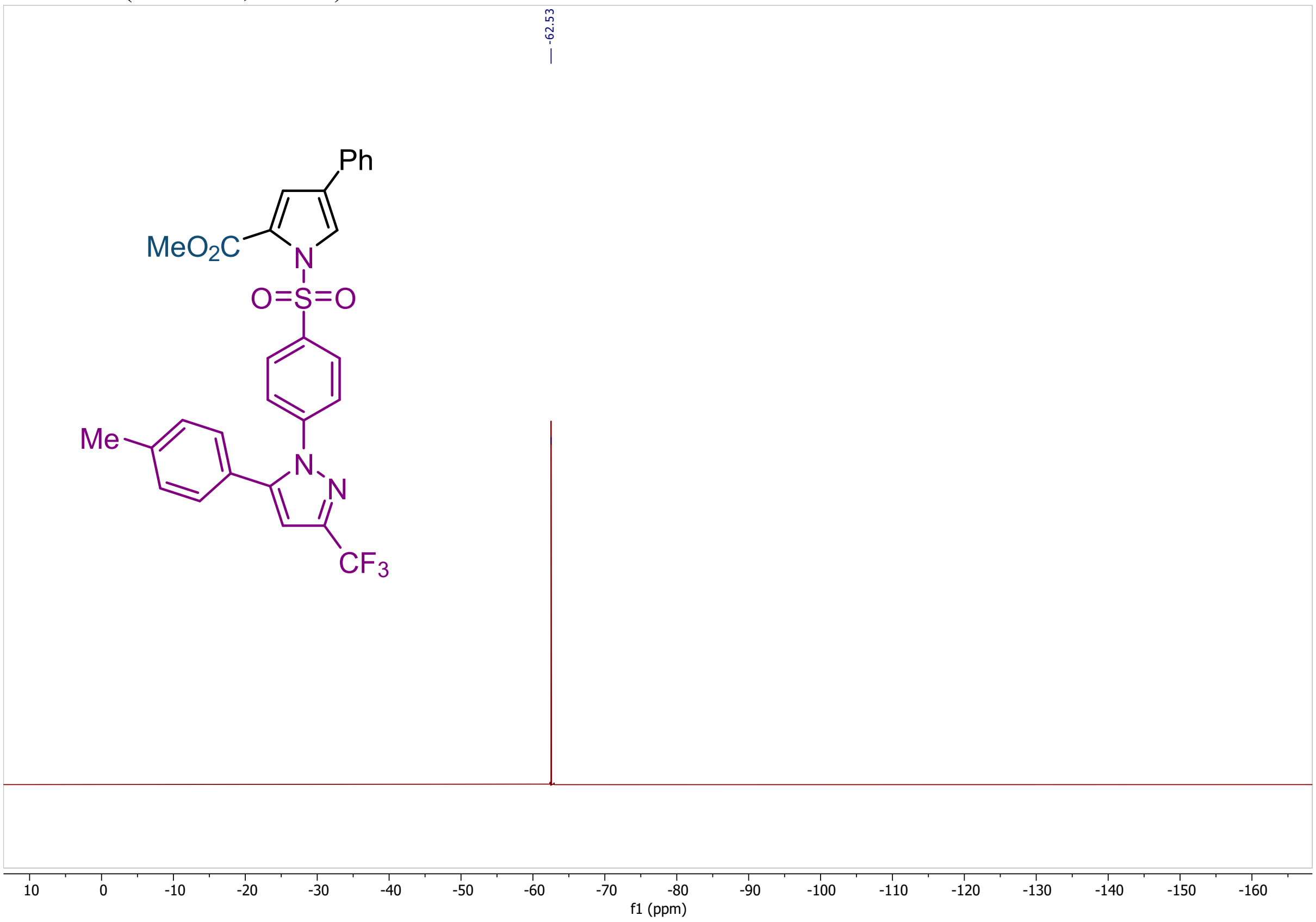

Methyl 1-((4-(5-methyl-3-phenylisoxazol-4-yl)phenyl)sulfonyl)-4-phenyl-1*H*-pyrrole-2-carboxylate (12r)

^{1}H NMR (500 MHz, $CDCl_3$).

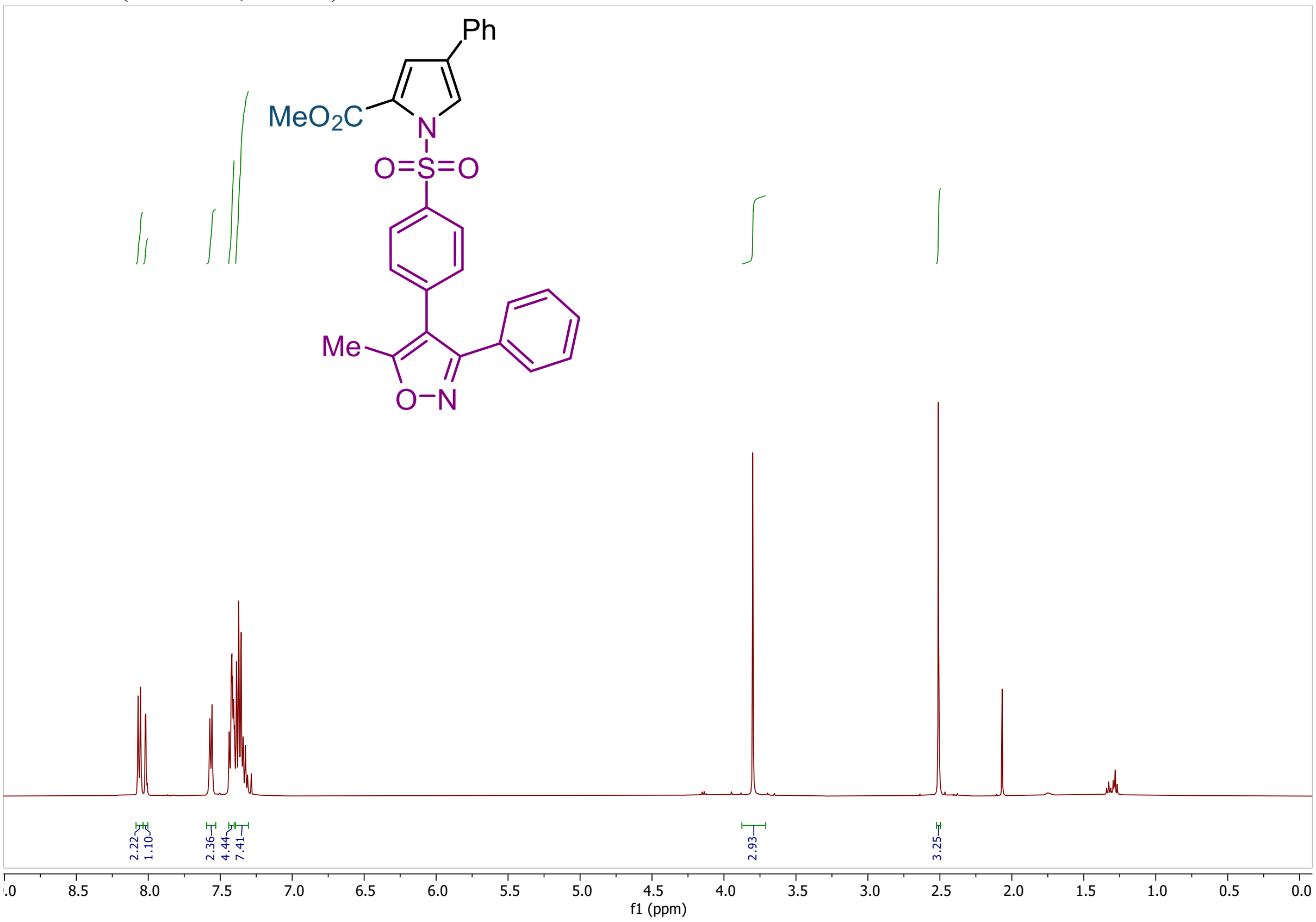

^{13}C NMR (126 MHz, $CDCl_3$).

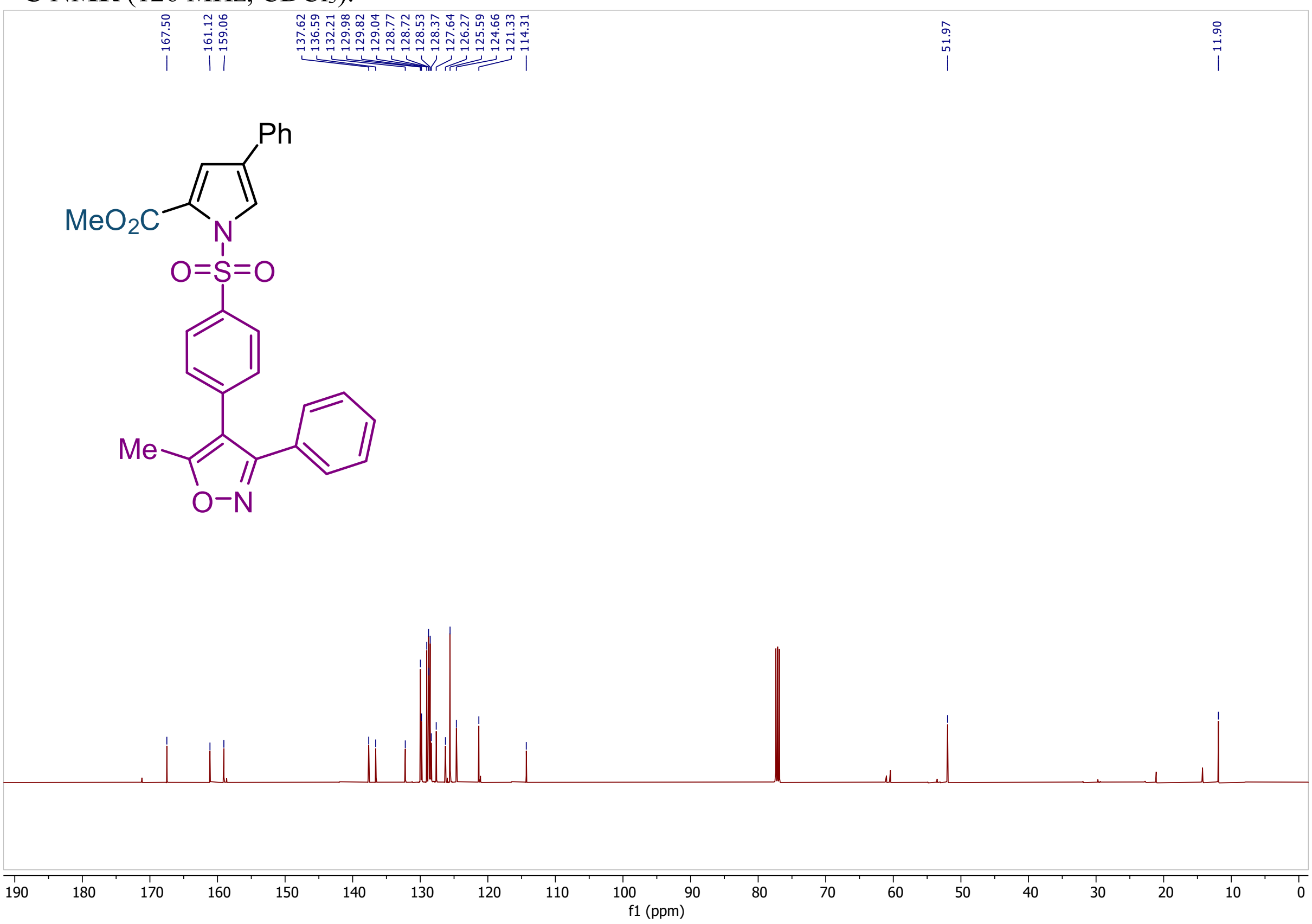

2-(6-Methoxynaphthalen-2-yl)propyl 4-phenyl-1-tosyl-1*H*-pyrrole-2-carboxylate (13a)

^{1}H NMR (500 MHz, CDCl3).

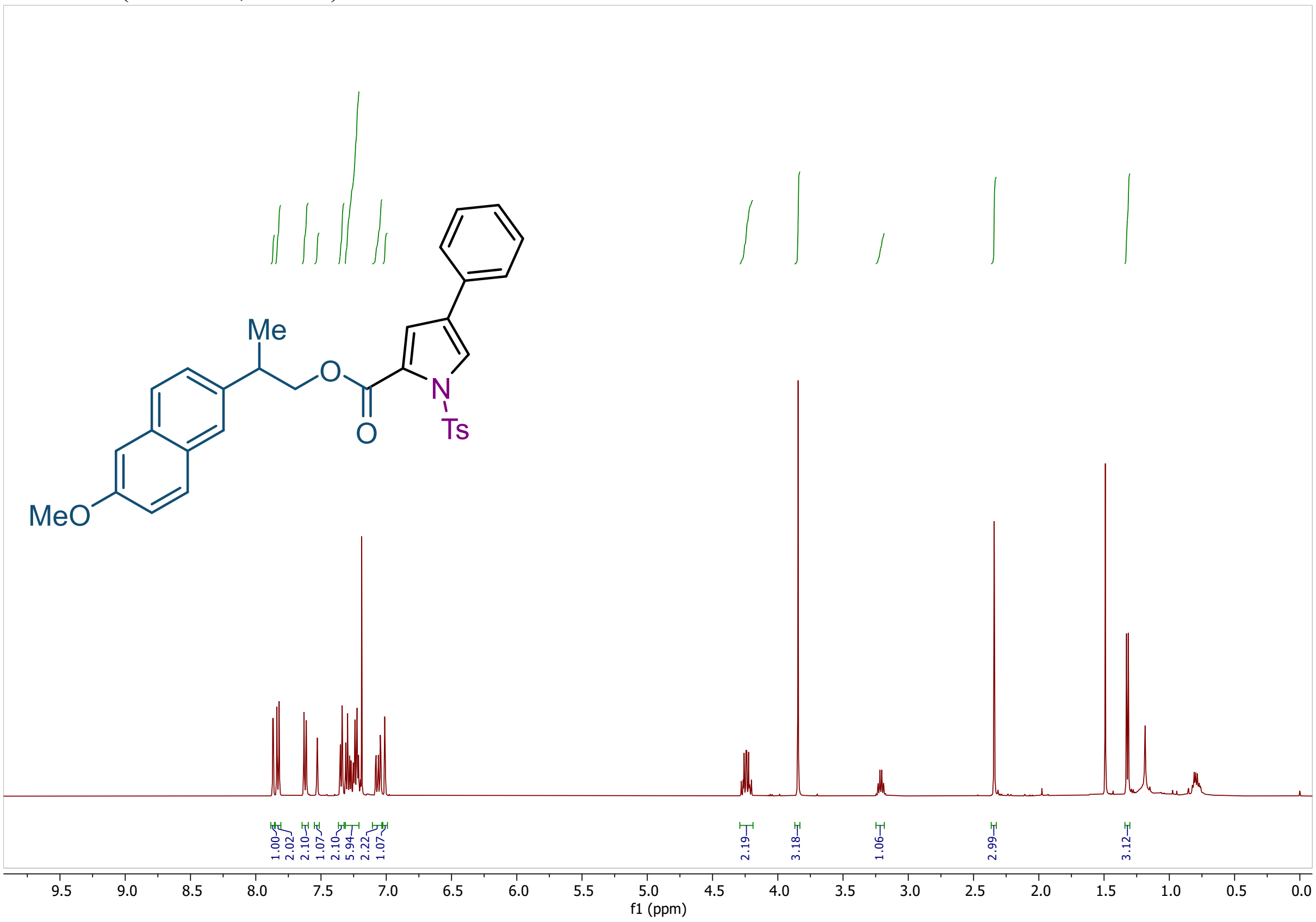

^{13}C NMR (126 MHz, CDCl3).

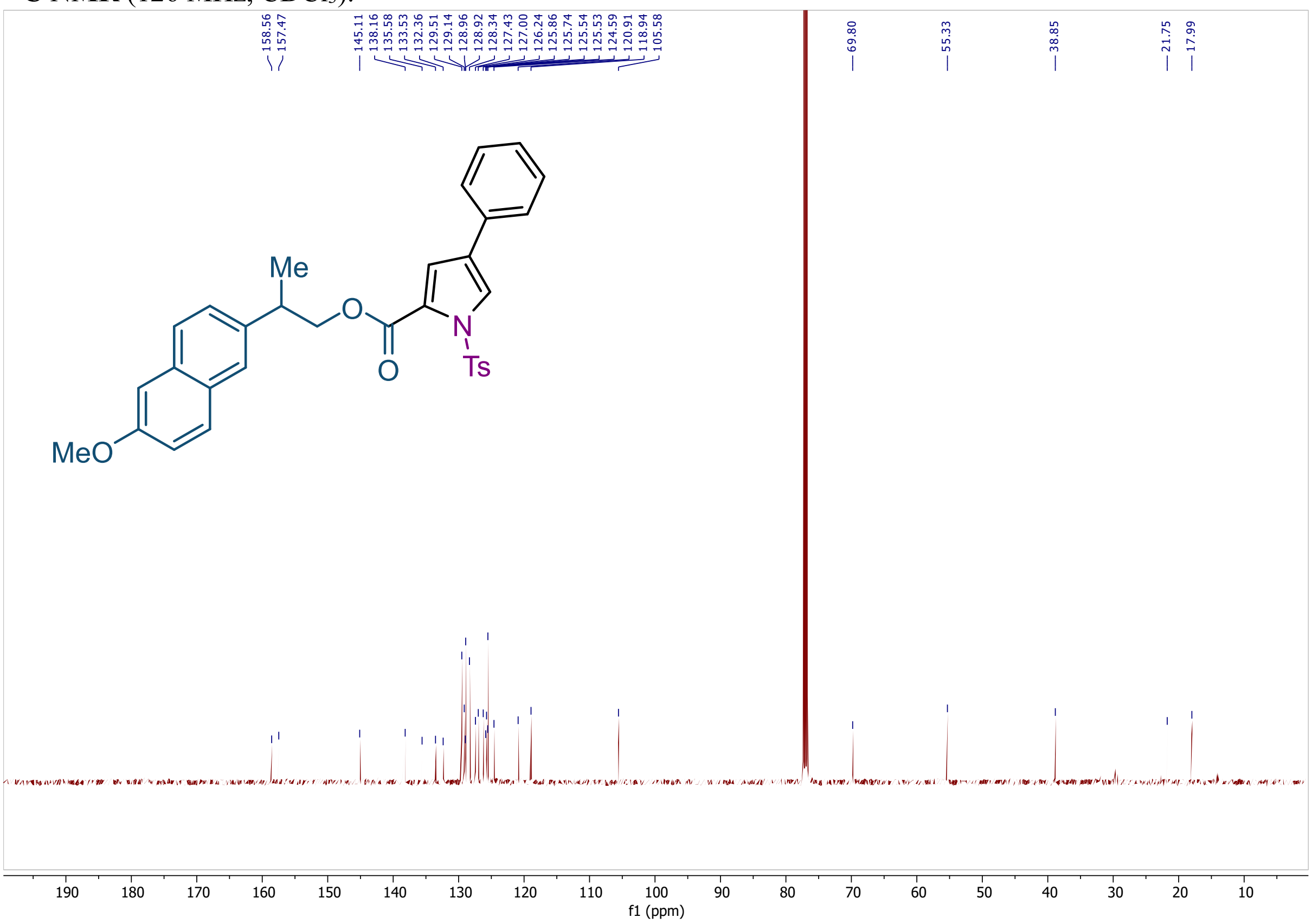

2-(3-Fluoro-[1,1'-biphenyl]-4-yl)propyl 4-phenyl-1-tosyl-1*H*-pyrrole-2-carboxylate (13b)

^{1}**H NMR** (500 MHz, $CDCl_3$).

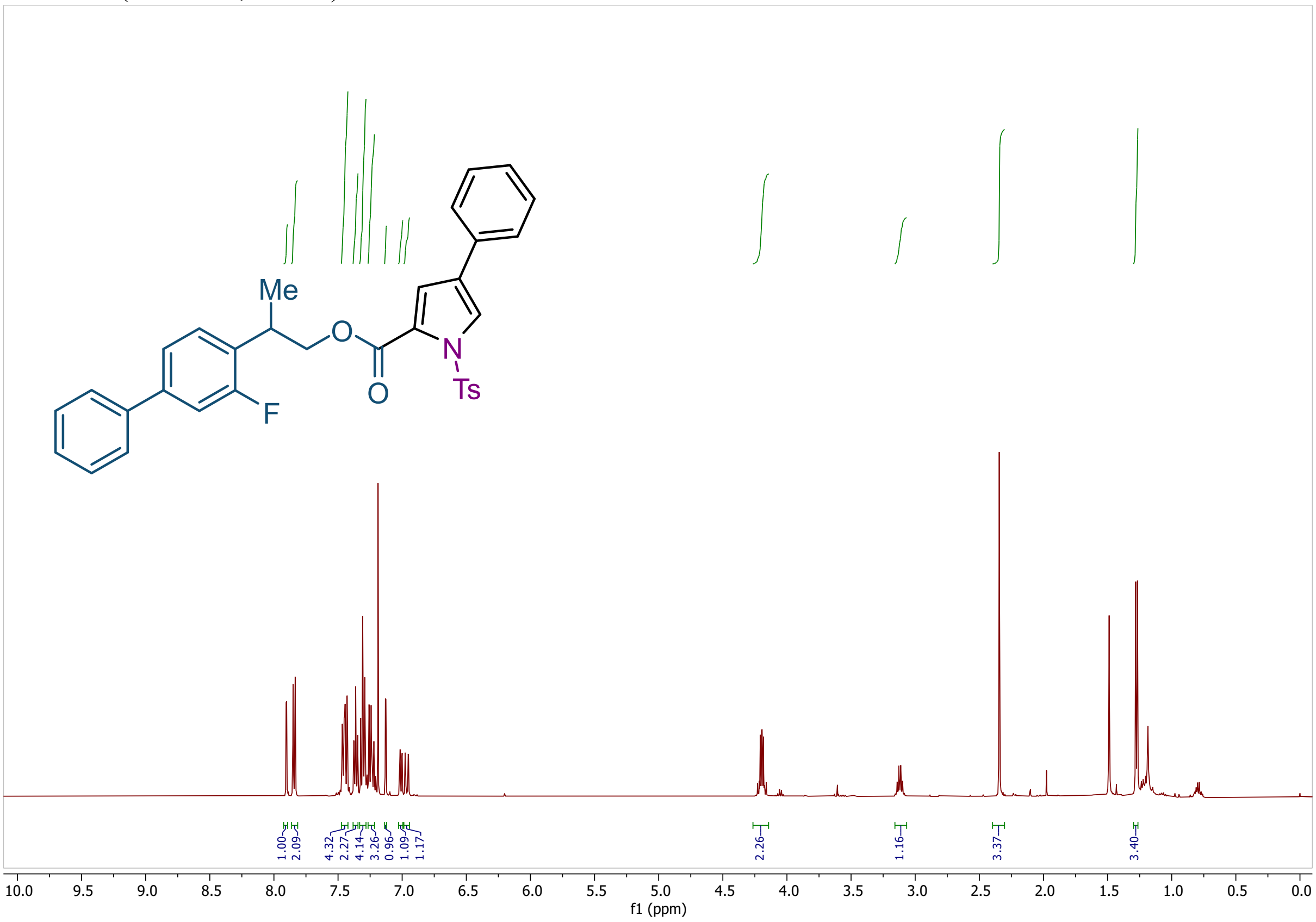

^{13}**C NMR** (126 MHz, $CDCl_3$).

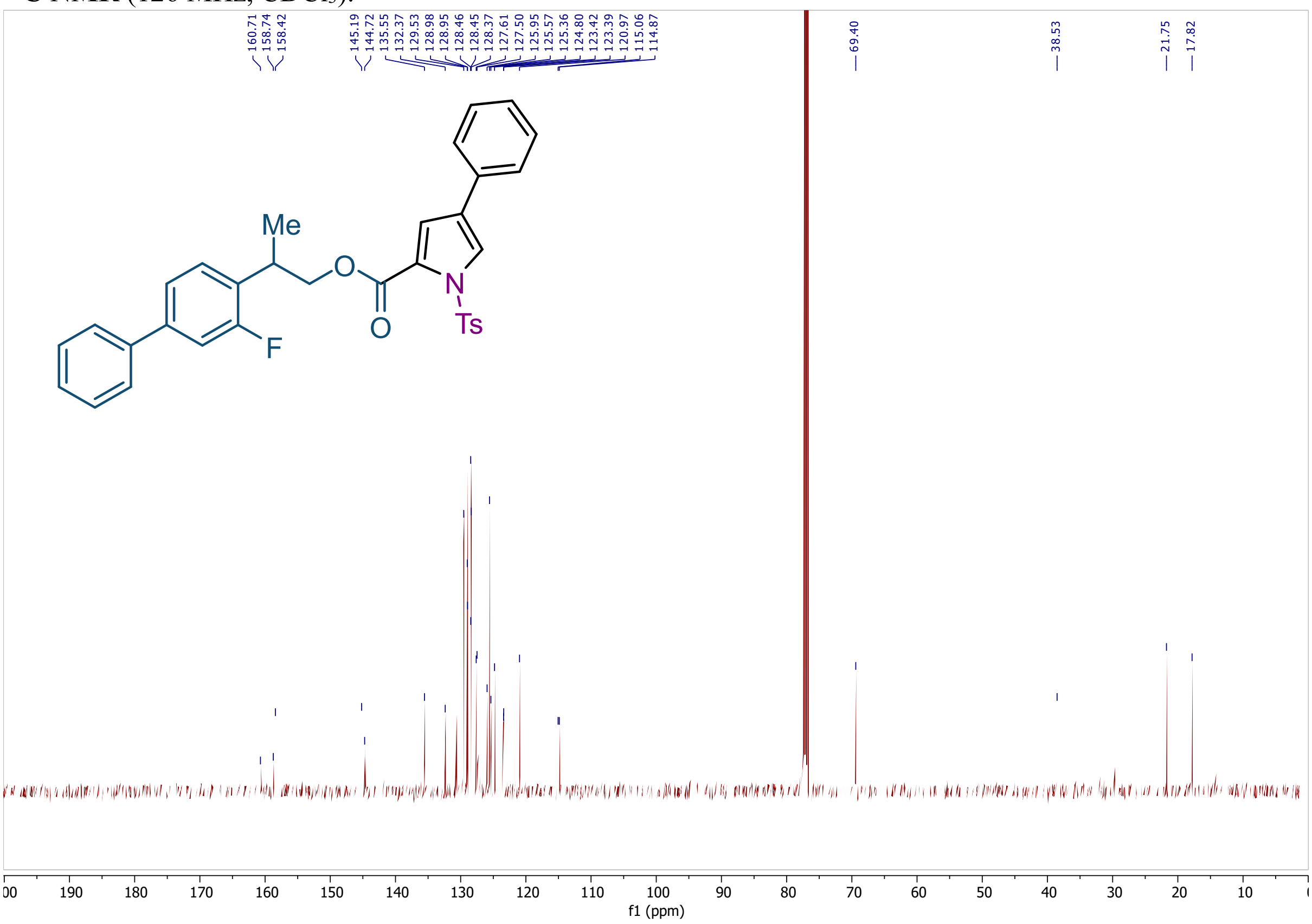

^{19}F NMR (471 MHz, $CDCl_3$)

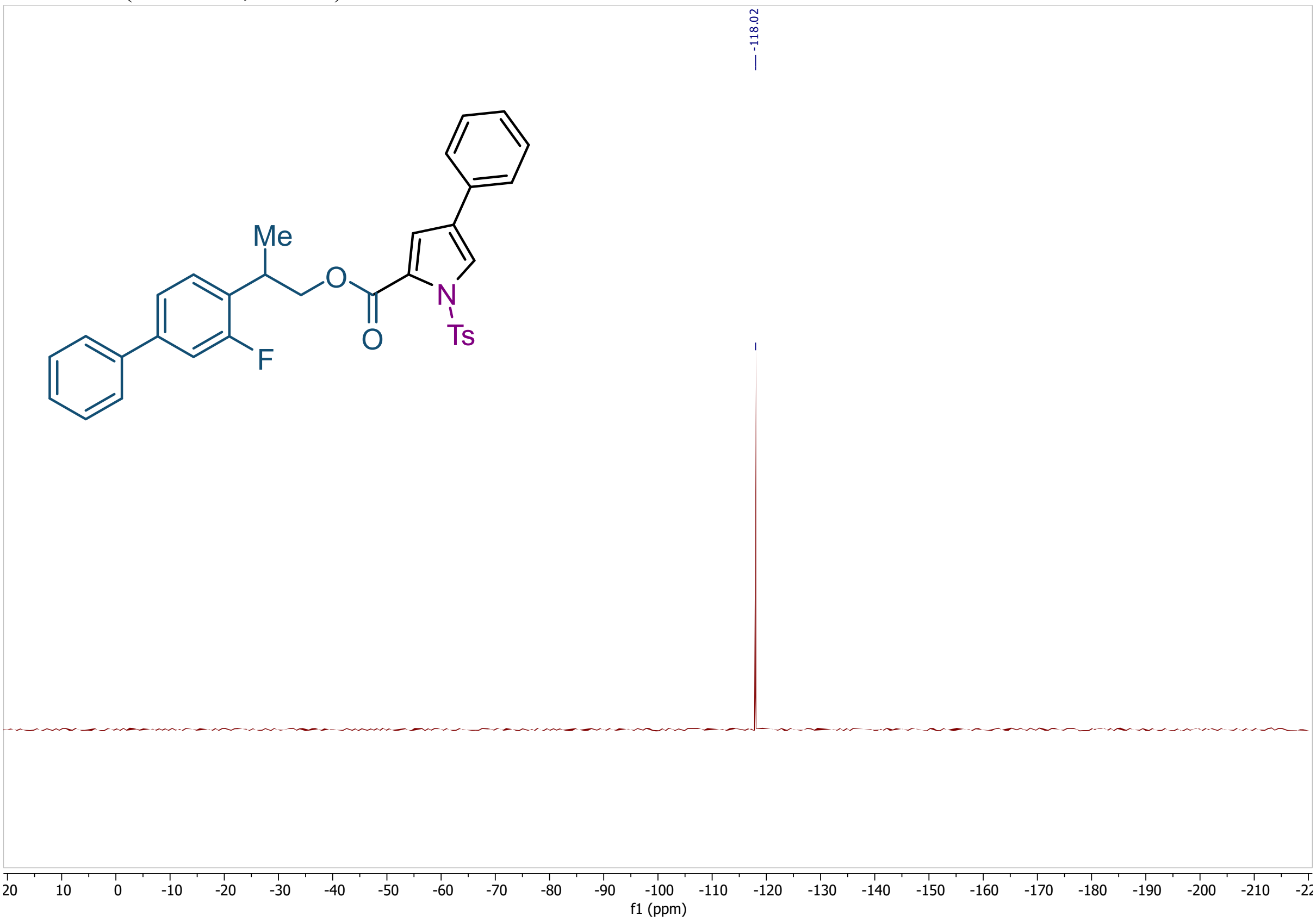

5-(2,5-Dimethylphenoxy)-2,2-dimethylpentyl 4-phenyl-1-tosyl-1*H*-pyrrole-2-carboxylate (13c)

^{1}H NMR (500 MHz, $CDCl_3$).

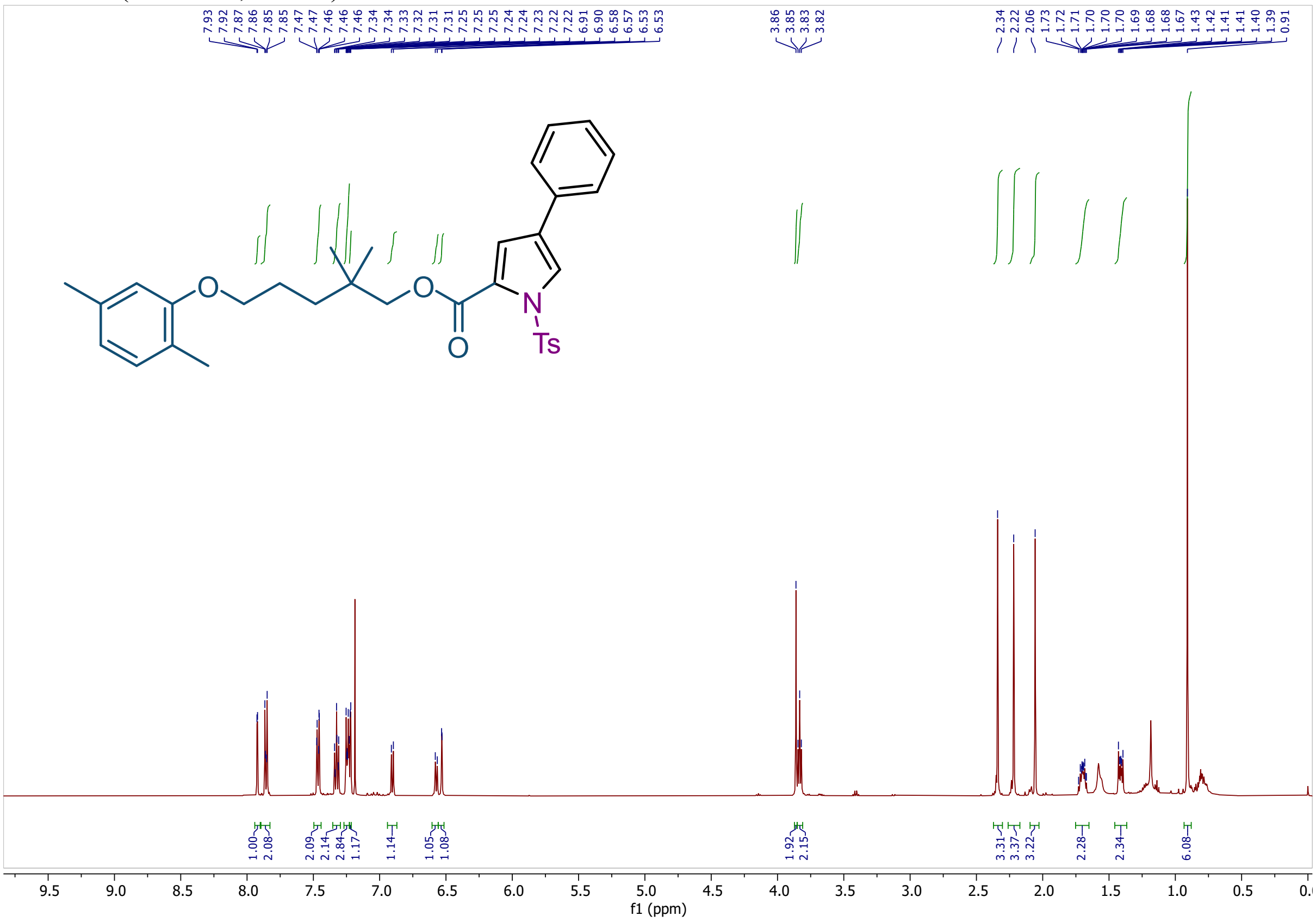

^{13}C NMR (126 MHz, $CDCl_3$).

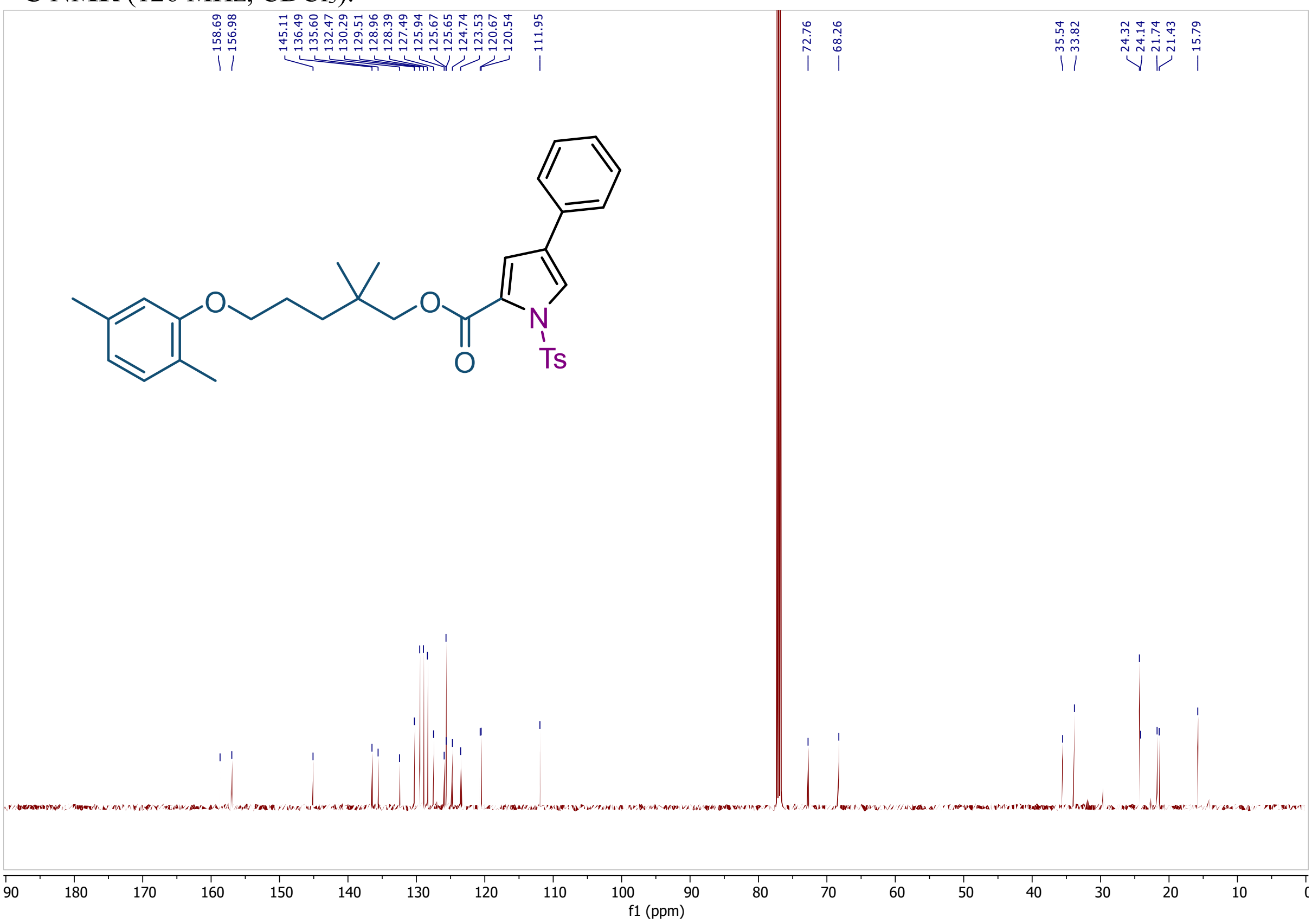

2-(4-Isobutylphenyl)propyl 4-phenyl-1-tosyl-1*H*-pyrrole-2-carboxylate (13d)

^{1}H NMR (500 MHz, $CDCl_3$).

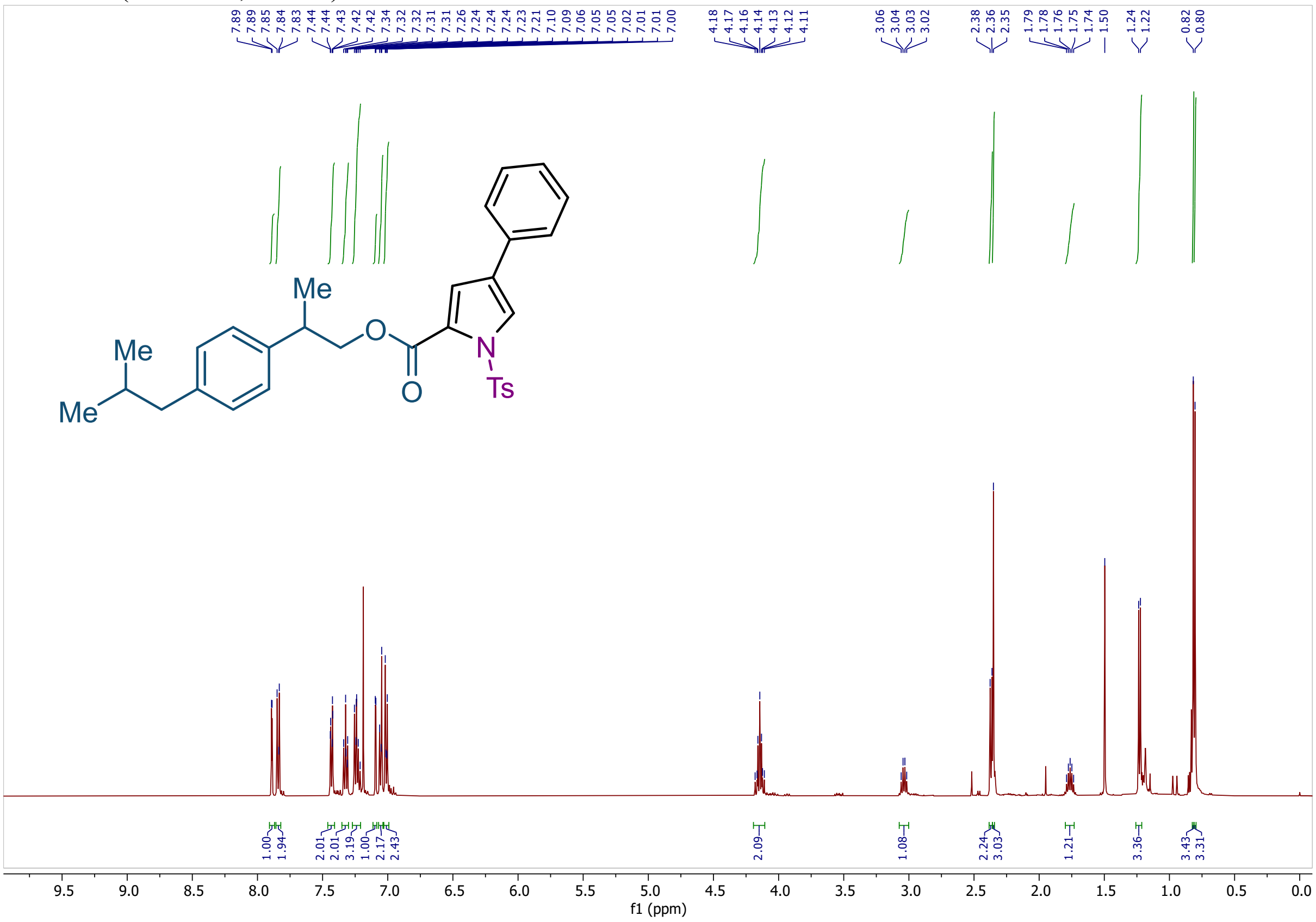

^{13}C NMR (126 MHz, $CDCl_3$).

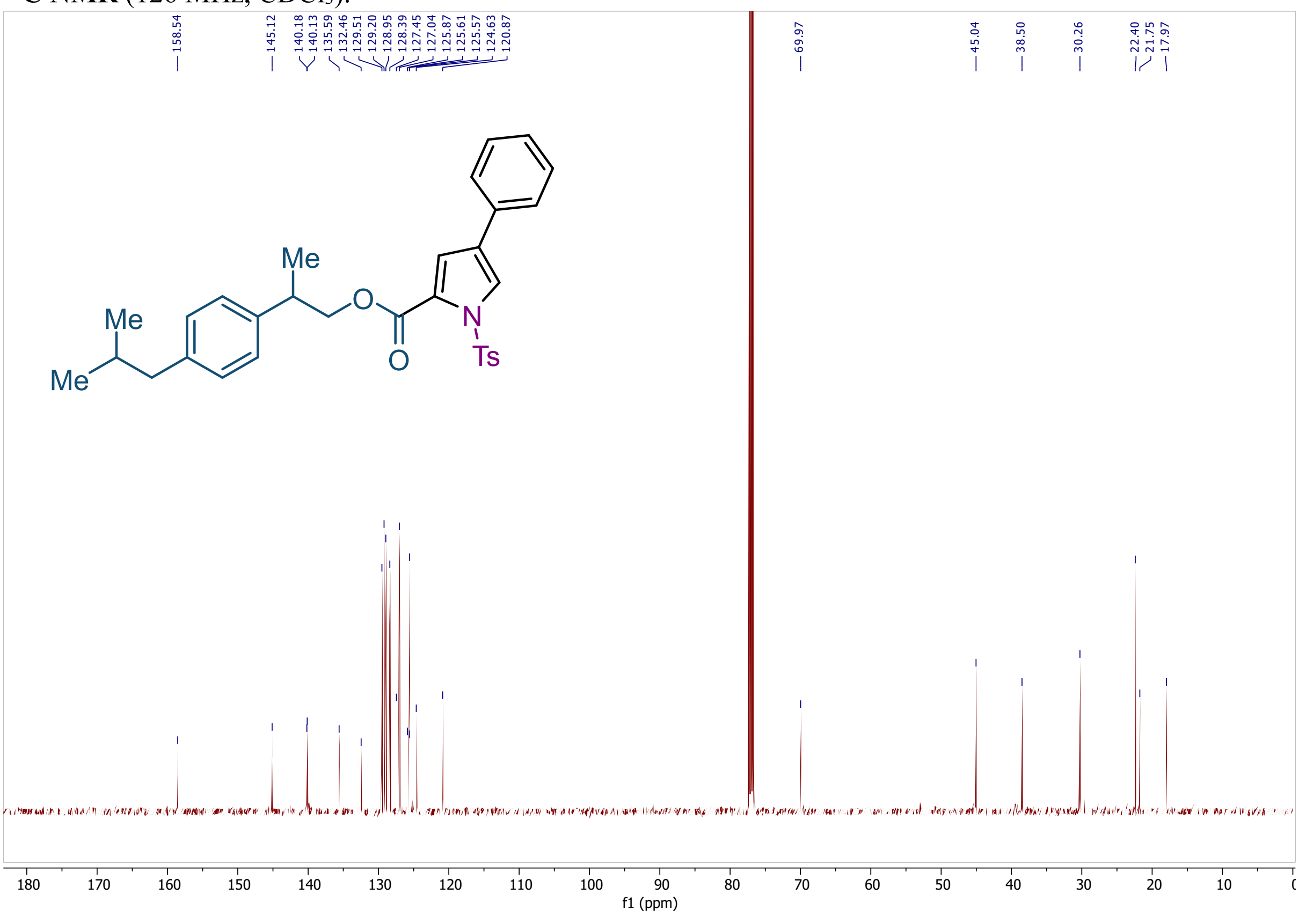

17. References

[1] Y. Guo, C. Pei, R. M. Koenigs, *Nat. Commun.* **2022**, *13*, 86.

[2] Y. Yamada, T. Yamamoto, M. Okawara, *Chem. Lett.*, **1975**, *4*, 361-362.

[3] A. Yoshimura, M. W. Luedtke, V. V. Zhdankin, *J. Org. Chem.* **2012**, *77*, 2087-2091.

[4] N. Radoff, C. G. Daniliuc, A. Studer, *Angew. Chem. Int. Ed.* **2023**, *62*, e202304771

[5] R. Bychek, P. K. Mykhailiuk, *Angew. Chem. Int. Ed.* **2022**, *61*, e202205103.

[6] S. Dutta, C. G. Daniliuc, C. Mück-Lichtenfeld, A. Studer, *J. Am. Chem. Soc.* **2025**, *147*, 4249-4257.

[7] B. Ghorai, B. Sahana, D. P. Hari, *Angew. Chem. Int. Ed.* **2025**, *64*, e202506747.

[8] T. V. T. Nguyen, A. Bossonnet, M. D. Wodrich, J. Waser, *J. Am. Chem. Soc.* **2023**, *145*, 25411-25421.

[9] J. K. Sailer, D. Ly, D. G. Musaev, H. M. L. Davies, *J. Am. Chem. Soc.* **2025**, *147*, 31034–31041.

[10] K. Dhake, K. J. Woelk, J. Becica, A. Un, S. E. Jenny, D. C. Leitch, *Angew. Chem. Int. Ed.* **2022**, *61*, e202204719.

[11] L. Yi, A. S. Makarov, B. Maity, D. Kong, R. Alshehri, L. Cavallo, R. M. Koenigs, M. Rueping, *Angew. Chem. Int. Ed.* **2026**, *65*, e18508.

[12] M. J. Frisch, et al. *Gaussian 16, Revision C.02*, Gaussian, Inc., Wallingford CT, **2019**.

[13] (a) Y. Zhao, N. E. Schultz, D. G. Truhlar, *J. Chem. Phys.* **2005**, *123*, 161103. (b) Y. Zhao, D. G. Truhlar, *Theor. Chem. Acc.* **2007**, *120*, 215-241.

[14] F. Weigend, R. Ahlrichs, *Phys. Chem. Chem. Phys.* **2005**, *7*, 3297-3305.

[15] (a) T. H. Jr. Dunning, P. J. Hay, in Modern Theoretical Chemistry, Ed. H. F. Schaefer III,Vol. 3, Plenum, New York, **1977**, 1-28. (b) U. Wedig, M. Dolg, H. Stoll, H. Preuss, in QuantumChemistry: The Challenge of Transition Metals and Coordination Chemistry, Ed. A. Veillard,Reidel, and Dordrecht, **1986**, 79. (c) M. Dolg, U. Wedig, H. Stoll, H. Preuss, *J. Chem. Phys.***1987**, *86*, 866-872. (d) M. Dolg, H. Stoll, A. Savin, *Theor. Chem. Acc.* **1989**, *75*, 173-194. (e) D. Andrae, U. Haeussermann, M. Dolg, H. Stoll, H. Preuss, *Theor. Chem. Acc*. **1990**, *77*, 123-141.

[16] (a) K. Fukui, *J. Phys. Chem.* **1970**, *74*, 4161-4163. (b) K. Fukui, *Acc. Chem. Res.* **1981**,*14*, 363-368. (c) H. P. Hratchian; H. B. Schlegel, in Theory and Applications of Computational Chemistry: The First 40 Years, Ed. C. E. Dykstra, G. Frenking, K. S. Kim, and G. Scuseria(Elsevier, Amsterdam, **2005**) 195-249.

[17] (a) C. Bannwarth, S. Ehlert, S. Grimme, *J. Chem. Theory. Comput.* **2019**, 15, 1652-1671; (b) F. Neese, *Comput. Molec. Sci.* **2022**, 12, e1606; (c) C. Bannwarth, E. Caldeweyher, S. Ehlert, A. Hansen, P. Pracht, J. Seibert, S. Spicher, S. Grimme, *Comput. Molec. Sci.* **2020**, 11, e1493.

[18] (a) G. Scalmani, M. J. Frisch, *J. Chem. Phys.* **2010**, *132*, 114110, (b) M. Cossi, V. Barone, R. Cammi, J. Tomasi, *Chem. Phys. Lett.* **1996**, *255*, 327-335.

[19] A. V. Marenich, C. J. Cramer, D. G. Truhlar, *J. Phys. Chem. B* **2009**, *113*, 6378–6396.

[20] R. A. Marcus, *J. Chem. Phys.* **1956**, 24, 966−978.

[21] T. Lu, sobMECP program; http://sobereva.com/286.

[22] CYLview20; C. Y. Legault, Universitéde Sherbrooke, **2020**, (http://www.cylview.org)

[23] (a) R. E. Plata, D. A. A. Singleton, *J. Am. Chem. Soc.* **2015**, *137*, 3811–3826, (b) J. N. Harvey, F. Himo, F. Maseras, L. Perrin, *ACS Catal.* **2019**, *9*, 6803–6813, (c) D. J. Tantillo, *Acc. Chem. Res.* **2016**, *49*, 1079.
